# Design of DNA Intercalators Based on 4-Carboranyl-1,8-Naphthalimides: Investigation of Their DNA-Binding Ability and Anticancer Activity

**DOI:** 10.3390/ijms23094598

**Published:** 2022-04-21

**Authors:** Sebastian Rykowski, Dorota Gurda-Woźna, Marta Orlicka-Płocka, Agnieszka Fedoruk-Wyszomirska, Małgorzata Giel-Pietraszuk, Eliza Wyszko, Aleksandra Kowalczyk, Paweł Stączek, Katarzyna Biniek-Antosiak, Wojciech Rypniewski, Agnieszka B. Olejniczak

**Affiliations:** 1 Institute of Medical Biology, Polish Academy of Sciences, 106 Lodowa St., 93-232 Lodz, Poland; srykowski@cbm.pan.pl; 2 Institute of Bioorganic Chemistry, Polish Academy of Sciences, 12/14 Z. Noskowskiego St., 61-704 Poznan, Poland; d_gurda@ibch.poznan.pl (D.G.-W.); mplocka@ibch.poznan.pl (M.O.-P.); agaw@ibch.poznan.pl (A.F.-W.); giel@ibch.poznan.pl (M.G.-P.); wyszkoe@ibch.poznan.pl (E.W.); kbiniek@ibch.poznan.pl (K.B.-A.); wojtekr@ibch.poznan.pl (W.R.); 3 Department of Molecular Microbiology, Faculty of Biology and Environmental Protection, University of Lodz, 12/16 Banacha St., 90-237 Lodz, Poland; aleksandra.strzelczyk@biol.uni.lodz.pl (A.K.); pawel.staczek@biol.uni.lodz.pl (P.S.)

**Keywords:** 1,8-naphthalimides, carborane, anticancer activity, intercalators

## Abstract

In the present study, we continue our work related to the synthesis of 1,8-naphthalimide and carborane conjugates and the investigation of their anticancer activity and DNA-binding ability. For this purpose, a series of 4-carboranyl-1,8-naphthalimide derivatives, mitonafide, and pinafide analogs were synthesized using click chemistry, reductive amination, amidation, and Mitsunobu reactions. The calf thymus DNA (ct-DNA)-binding properties of the synthesized compounds were investigated by circular dichroism (CD), UV–vis spectroscopy, and thermal denaturation experiments. Conjugates **54**–**61** interacted very strongly with ct-DNA (∆*T*_m_ = 7.67–12.33 °C), suggesting their intercalation with DNA. They were also investigated for their in vitro effects on cytotoxicity, cell migration, cell death, cell cycle, and production of reactive oxygen species (ROS) in a HepG2 cancer cell line as well as inhibition of topoisomerase IIα activity (Topo II). The cytotoxicity of these eight conjugates was in the range of 3.12–30.87 µM, with the lowest IC_50_ value determined for compound **57**. The analyses showed that most of the conjugates could induce cell cycle arrest in the G0/G1 phase, inhibit cell migration, and promote apoptosis. Two conjugates, namely **60** and **61**, induced ROS production, which was proven by the increased level of 2′-deoxy-8-oxoguanosine in DNA. They were specifically located in lysosomes, and because of their excellent fluorescent properties, they could be easily detected within the cells. They were also found to be weak Topo II inhibitors.

## 1. Introduction

**Figure 1:**
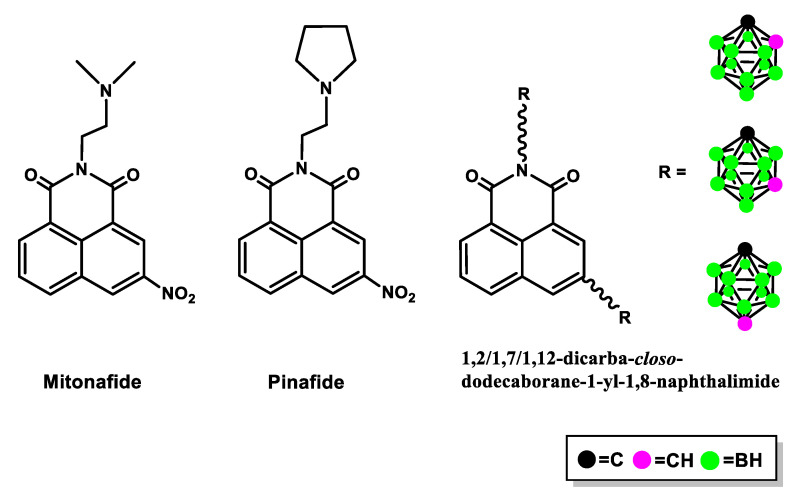
Structures of mitonafide, pinafide, and 1,8-naphthalimides modified with a carborane cluster [8,9].

1,8-Naphthalimide is a class of heterocycles, constituting a π-deficient planar aromatic structure and a versatile pharmacophore with diverse biological applications in pharmaceuticals such as anticancer, antibacterial, antiviral, and analgesic agents [1,2]. Naphthalimides exert antiproliferative activity primarily because of their ability to intercalate into the base pairs of DNA, through DNA-groove binding and topoisomerase inhibition [3,4]. They also exert anticancer activity through inhibition of receptor tyrosine kinases [5], induction of reactive oxygen species production [6], and other mechanisms [7]. The most popular and well-described naphthalimides are mitonafide, pinafide (Figure 1), amonafide, and elinafide. The development of functional 1,8-naphthalimide derivatives as anticancer agents and DNA-targeting agents is a fast-growing area and has resulted in several products. Different strategies and use of an aromatic ring and ring substituents (at the 3-, 4-position or disubstituted) have been attempted to obtain particular naphthalimide products with improved potency and reduced side effects [4].

In our work, we have described thus far the methods used to synthesize naphthalimides modified with carborane or metallacarborane groups through the modification of imide (Figure 1) [8] and bearing the carborane group at position 3 of the ring [9]. We observed that the attachment site of the boron cluster to the naphthalimide moiety, type of the boron cluster, and structure of the linker between the boron cluster and the heteroaromatic ring system influenced various cytotoxic activities against HepG2 cancer cells. The tested conjugates induced cell cycle arrest at the G0/G1 or G2M phase and mainly activated apoptosis. Selected conjugates activated autophagy and ferroptosis. The presence of the carboranyl cluster at position 3 did not promote them as effective topoisomerase II (Topo II) inhibitors. However, the studied compounds were rather weak classical DNA intercalators as compared to mitonafide [8]. Additionally, J. Laskova et al. classically obtained 3-nitro-1,8-naphthalimides bearing the *nido*-carborane and *closo*-dodecaborate modifications by reaction of naphthalic anhydride with ammonium derivatives containing a boron cluster. A mitonafide derivative modified with *nido*-carborane was obtained using the nucleophilic ring-opening reaction of the corresponding cyclic oxonium derivatives [10]. However, physicochemical and biological studies on this group of compounds have not been performed.

In recent years, several studies have described the potential biomedical application of boron clusters, mostly as boron carriers for boron neutron capture therapy, pharmacophores, or modulators of different types of chemical compounds [11,12,13]. The properties of boron clusters that are critical to their use in medicinal chemistry include abiotic origin (these are therefore chemically and biologically orthogonal to native cellular components), the unique interaction properties of boron clusters and their derivatives with biomolecules, lipophilicity, amphiphilicity, hydrophobicity (depending on the chemical structure), chemical stability, susceptibility to functionalization, spherical or ellipsoidal geometry, and rigid three-dimensional arrangement [14,15]. As reported earlier, the introduction of boron clusters into drug molecules could improve the activity toward resistant forms [16,17,18,19] or to obtain hitherto unknown nematicidal activity [20].

Based on the properties of boron clusters useful in medicinal chemistry and the results achieved thus far for 1,8-naphthalimide–boron cluster conjugates, in the present work, we describe a method for synthesizing 1,8-naphthalimide derivatives, analogs of mitonafide and pinafide, modified with 1,2-dicarba-*closo*-dodecaborane (*ortho*-carborane) or 1,7-dicarba-*closo*-dodecaborane (*meta*-carborane) at position 4 of the heteroaromatic skeleton and investigate their interaction with calf thymus DNA (ct-DNA), their anticancer activity, and their ability to induce cell death, cell cycle arrest, ROS production, and inhibition of human topoisomerase IIα.

## 2. Results and Discussion

### 2.1. Chemistry

#### 2.1.1. Synthesis of Mitonafide and Pinafide Analogs Containing Carborane Clusters

**Scheme 1:**
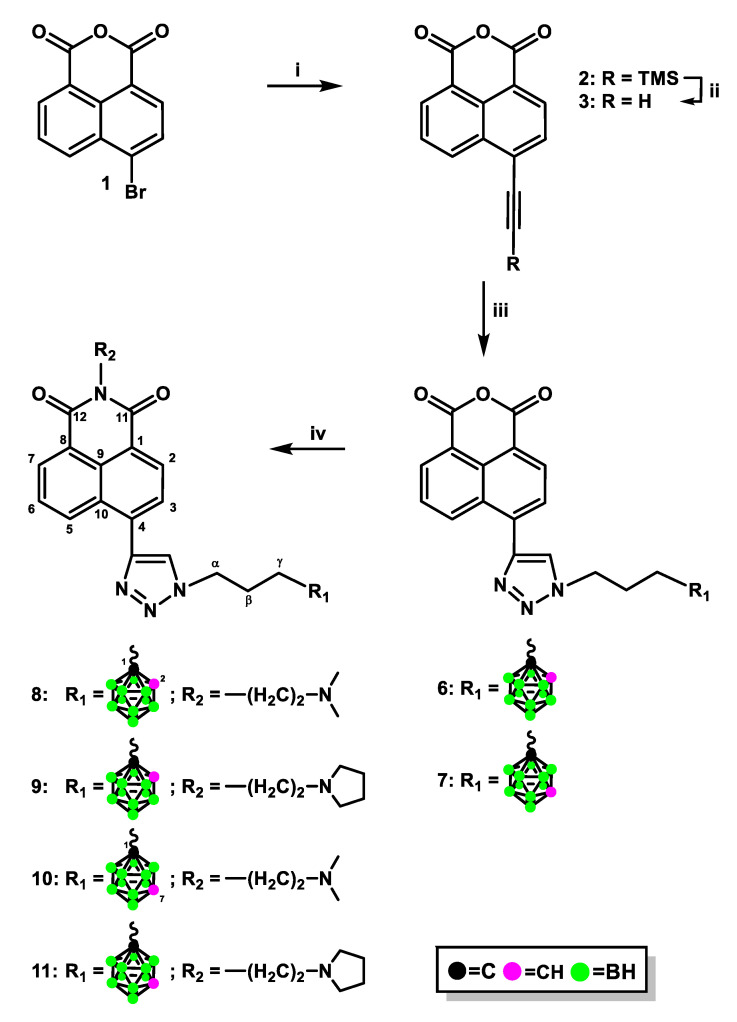
Synthesis of 1,8-naphthalimide-*ortho*/*meta*-carborane conjugates **8**–**11**. (**i**). HCCSi(CH_3_)_3_, Pd(PPh_3_)_4_, CuI, TEA, DMF, 1 h, 60 °C; (**ii**). TFA, 9 h, 50 °C; (**iii**). 1-(3-azidopropyl)-1,2-dicarba-*closo*-dodecaborane (**4**), CuSO_4_•5H_2_O, sodium ascorbate, TBTA, THF/H_2_O, 2 h, 40 °C (for **6**); 1-(3-azidopropyl)-1,7-dicarba-*closo*-dodecaborane (**5**), CuSO_4_•5H_2_O, sodium ascorbate, TBTA, THF/H_2_O, 2 h, 40 °C (for **7**); (**iv**). *N*,*N*-dimethylethylenediamine (for **8** and **10**), *N*-(2-aminoethyl)pyrrolidine (for **9** and **11**), EtOH, 2 h, 45 °C.

**Scheme 2:**
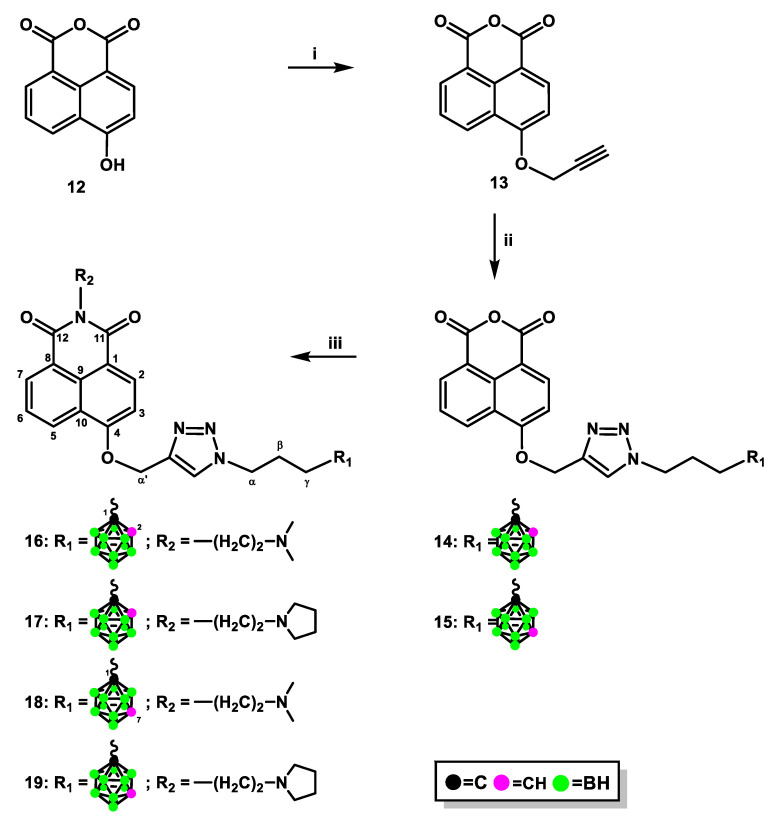
Synthesis of 1,8-naphthalimide-*ortho*/*meta*-carborane conjugates **16**–**19**. (**i**). HCCCH_2_OH, PPh_3_, DIAD, THF, 72 h, RT; (**ii**). 1-(3-azidopropyl)-1,2-dicarba-*closo*-dodecaborane (**4**), CuSO_4_•5H_2_O, sodium ascorbate, THF/H_2_O, 24 h, 40 °C (for **14**); 1-(3-azidopropyl)-1,7-dicarba-*closo*-dodecaborane (**5**), CuSO_4_•5H_2_O, sodium ascorbate, THF/H_2_O, 24 h, 40 °C (for **15**); (**iii**). *N*,*N*-dimethylethylenediamine (for **16** and **18**), *N*-(2-aminoethyl)pyrrolidine (for **17** and **19**), EtOH, 3–4 h, 40 °C.

**Scheme 3:**
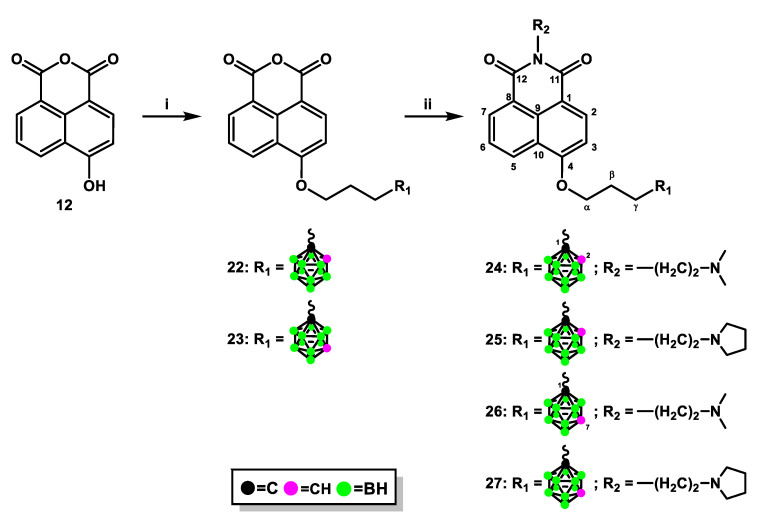
Synthesis of 1,8-naphthalimide derivatives containing *ortho*-/*meta*-carborane using Mitsunobu reaction. (**i**). 1-(3-hydroxy-propyl)-1,2-dicarba-*closo*-dodecaborane (**20**), PPh_3_, DIAD, THF, 48 h, RT (for **22**); 1-(3-hydroxy-propyl)-1,7-dicarba-*closo*-dodecaborane (**21**), PPh_3_, DIAD, THF, 72 h, RT (for **23**); (**ii**). *N*,*N*-dimethylethylenediamine (for **24** and **26**), *N*-(2-aminoethyl)pyrrolidine (for **25** and **27**), EtOH, 4 h, 45 °C.

**Scheme 4:**
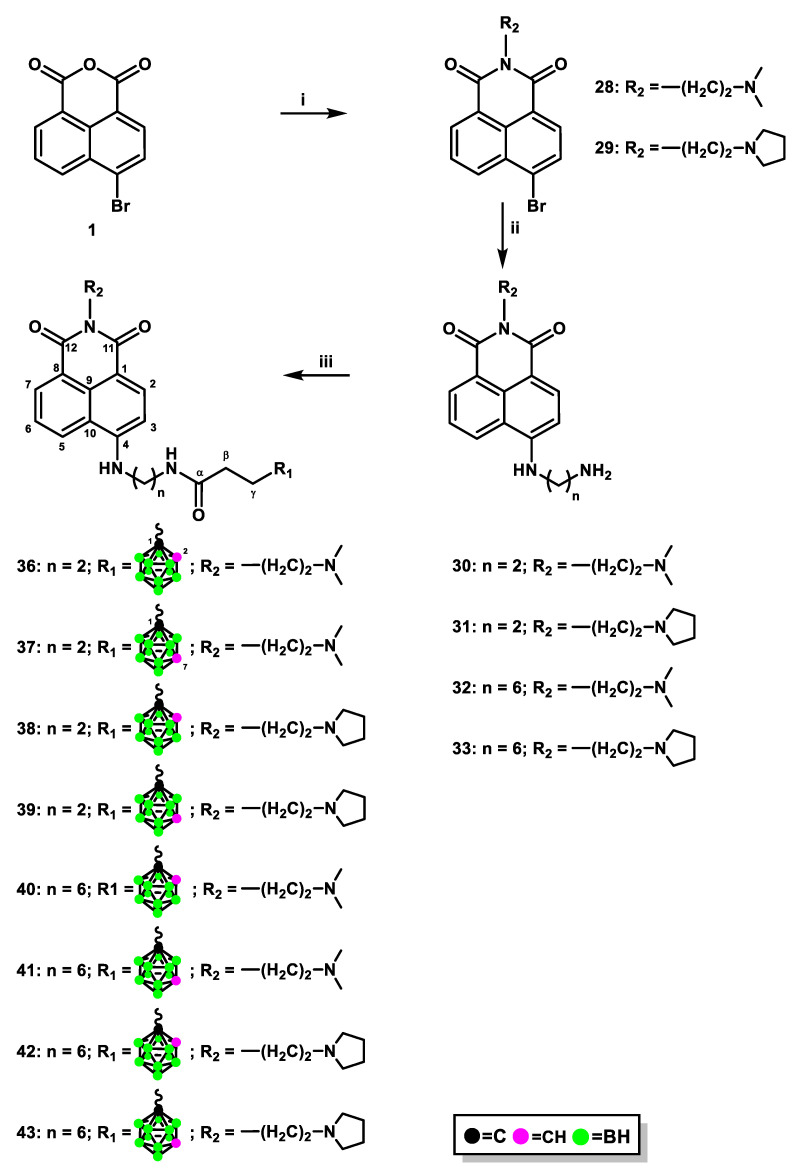
Modification of 1,8-naphthalimide derivatives with *ortho*-/*meta*-carborane through the formation of an amide bond. (**i**). *N*,*N*-dimethylethylenediamine, EtOH, 2 h, 45 °C (for **28**); *N*-(2-aminoethyl)pyrrolidine, EtOH, 2 h, 45 °C (for **29**); (**ii**). ethane-1,2-diamine, 2-methoxyethanol, 7 h, reflux (for **30** and **31**); hexane-1,6-diamine, 2-methoxyethanol, 7 h, reflux (for **32** and **33**); (**iii**). 3-(1,2-dicarba-*closo*-dodecaboran-1-yl)propionic acid *N*-succinimidyl ester (**34**), CH_2_Cl_2_, 5 h, 40 °C (for **36**, **38**, **40**, and **42**); 3-(1,7-dicarba-*closo*-dodecaboran-1-yl)propionic acid *N*-succinimidyl ester (**35**), CH_2_Cl_2_, 1.5–5 h, 40 °C (for **37**, **39**, **41**, and **43**).

**Scheme 5:**
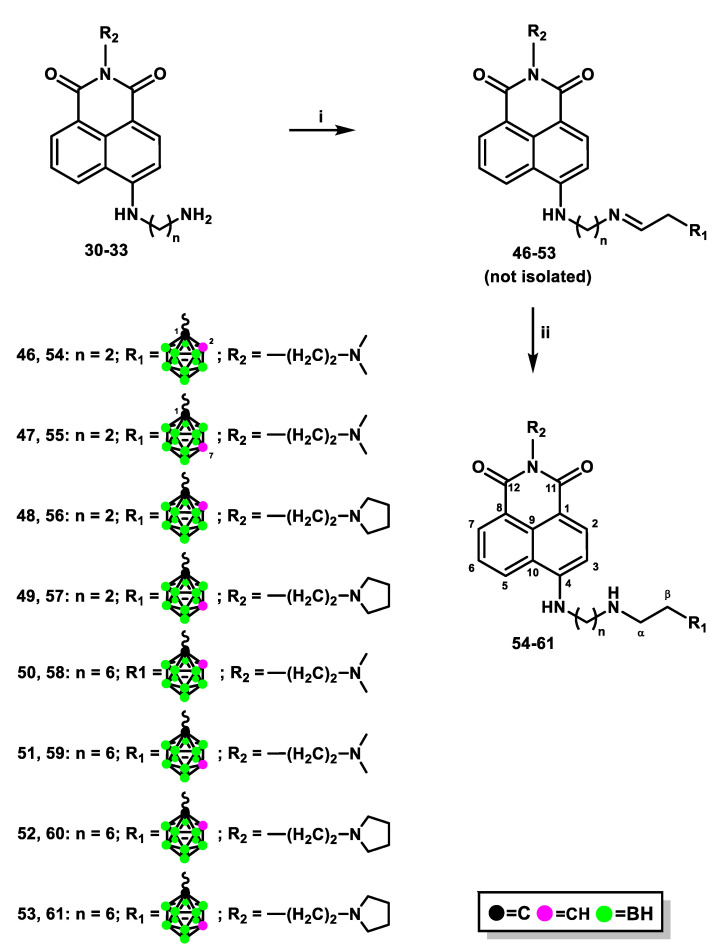
Synthesis of conjugates **54**–**61** through reductive amination. (**i**). 2-(1,2-dicarba-*closo*-dodecaboran-1-yl)ethanal (**44**), THF, 24 h, reflux (for **46**, **48**, **50**, and **52**); 2-(1,7-dicarba-*closo*-dodecaboran-1-yl)ethanal (**45**), AcOEt, 24 h, reflux (for **47**); 2-(1,7-dicarba-*closo*-dodecaboran-1-yl)ethanal (**45**), MeOH, 24 h, reflux (for **49**, **51**, and **53**); (**ii**). NaBH_3_CN, 24 h, RT.

The 1,8-naphthalimide derivatives containing a carborane cluster at position 4 of the heteroaromatic skeleton described in this study (compounds **8**–**11**, **16**–**19**, **24**–**27**, **36**–**43**, and **46**–**53**, Scheme 1, Scheme 2, Scheme 3, Scheme 4 and Scheme 5) were synthesized using the following methods: (1) copper(I)-catalyzed Huisgen–Meldal–Sharpless 1,3-dipolar cycloaddition of azides and alkynes (i.e., “click chemistry”) (Scheme 1 and Scheme 2); (2) Mitsunobu reaction (Scheme 3); (3) amidation reaction (Scheme 4), and (4) reductive amination (Scheme 5).

“Click chemistry” is a very efficient and popular method in pharmaceutical sciences and drug discovery. While several reactions fulfill the criteria, the Huisgen 1,3-dipolar cycloaddition of azides and terminal alkynes has emerged as the frontrunner [21,22,23]. In the first step, suitable boron cluster donors (**4**, **5**) [24] were dissolved in a mixture of THF/H_2_O and naphthalic anhydrides containing a terminal triple bond (**3** and **13**, Scheme 1 and Scheme 2), and a catalytic amount of sodium ascorbate and CuSO_4_•5H_2_O were added. The reactions were performed at 40 °C for 2 h (for compounds **6** and **7**) or 24 h (for compounds **14** and **15**).

After purification, the modified anhydrides **6**, **7**, **14**, and **15** (Scheme 1 and Scheme 2) were obtained in yields ranging between 58% and 88%, with a lower yield for compound **7** modified with *meta*-carborane. In the second step, the modified anhydrides were transformed to mitonafide (**8**, **10**, **16**, and **18**) and pinafide (**9**, **11**, **17**, and **19**) analogs through a condensation reaction with *N*,*N*-dimethylethylenediamine or *N*-(2-aminoethyl)pyrrolidine, respectively (Scheme 1 and Scheme 2).

The yield of products **8**–**11** and **16**–**19** achieved after isolation and purification by column chromatography ranged from 46% to 69% for conjugates containing *ortho*-carborane (**8**, **9**, **16**, and **17**) and from 82% to 91% for conjugates containing *meta*-carborane (**10**, **11**, **18**, and **19**). The modified anhydrides **6**, **7**, **14**, and **15** and the modified naphthalimides **8**–**11** and **16**–**19** were characterized by ^1^H-, ^13^C-, and ^11^B-NMR, FT-IR, MS, RP-HPLC (Figures S1–S86 (Electronic Supplementary Information, ESI)), and TLC.

Products **8**–**11** can also be directly synthesized from mitonafide or pinafide derivatives bearing terminal triple bonds with boron cluster donors **4** and **5**. However, an advantage of the synthetic pathway described in Scheme 1 is that one substrate (anhydride **6** or **7**) produces two target products—mitonafide and pinafide analogs. It should be emphasized that products **8**–**11** can be synthesized with a high yield only in the presence of tris[((1-benzyl)-*1H*-1,2,3-triazol-4-yl)methyl]amine (TBTA). During the reaction without TBTA, after adding copper(I) ions and sodium ascorbate to the reaction mixture containing anhydride or mitonafide/pinafide derivatives with terminal triple bonds, a black precipitate was formed in the reaction mixture, and the desired product was formed with a yield of less than 10%. It should also be noted that the use of TBTA is not required for synthesizing modified anhydrides **14** and **15** with good yields.

The Mitsunobu reaction is often used to synthesize biologically active molecules [25]. Treatment of 4-hydroxy-1,8-naphthalic anhydride (**12**) [26], alcohols containing *ortho*-carborane (**20**) [27] or *meta*-carborane (**21**) [28], and triphenylphosphine with diisopropyl azodicarboxylate (DIAD) in anhydrous THF at room temperature (RT) for 48 h (for compound **22**) or 72 h (for compound **23**) resulted in the corresponding anhydrides **22** and **23** bearing a carborane cluster (Scheme 3).

The modified naphthalic anhydrides were transformed to mitonafide and pinafide analogs with an appropriate amine (Scheme 3). The yield of products **24**–**27** after purification by column chromatography ranged from 51% to 65%. The structure, purity, and homogeneity of compounds **22**, **23**, and **24**–**27** were confirmed by ^1^H-, ^13^C-, and ^11^B-NMR, FT-IR, MS, RP-HPLC (Figures S87–S128, ESI), and TLC.

The synthesis of amides remains one of the most significant transformations, and it is one of the more frequently performed reactions. The amide function is unarguably a critical feature, being the constituent of natural and synthetic polymers and found in a wide variety of small bioactive molecules produced in both nature and laboratory [29].

We developed methods for synthesizing 4-aminonaphthalimide derivatives bearing carborane clusters **36**–**43** by the formation of an amide bond between naphthalimide and carborane using appropriate active esters containing a carborane cluster (Scheme 4). Briefly, *N*-[2-(dimethylamino)ethyl]-4-[(2-aminoethyl)amino]-1,8-naphthalimide (**30**), *N*-[2-(*N*-pyrrolidinyl)ethyl]-4-[(2-aminoethyl)amino]-1,8-naphthalimide (**31**), *N*-[2-(dimethylamino)ethyl]-4-[(6-aminohexyl)amino]-1,8-naphthalimide (**32**), or *N*-[2-(*N*-pyrrolidinyl)ethyl]-4-[(6-aminohexyl)amino]-1,8-naphthalimide (**33**) [30] and 3-(1,2-dicarba-*closo*-dodecaboran-1-yl)propionic acid *N*-succinimidyl ester (**34**) or 3-(1,7-dicarba-*closo*-dodecaboran-1-yl)propionic acid *N*-succinimidyl ester (**35**) [18] were dissolved in anhydrous CH_2_Cl_2_, and the solution was then stirred under anhydrous condition (Ar) for 1.5–5 h at 40 °C. After the reaction, the crude products were purified by column silica gel chromatography, following which conjugates **36**–**43** were obtained as a yellow solid in a moderate or good yield as follows: 71% (**36**), 91% (**37**), 40% (**38**, **40**), 59% (**39**), 78% (**41**), 51% (**42**), and 45% (**43**). The structure, purity, and homogeneity of these compounds were confirmed by ^1^H-, ^13^C-, and ^11^B-NMR, FT-IR, MS, RP-HPLC (Figures S129–S184, ESI), and TLC. It is worth mentioning that the attachment of the carborane cluster directly to 4-amine naphthalic derivatives was abandoned due to the reaction’s low yield (10–20%). Therefore, we prepared suitable 1,8-naphthalimide substrates containing ethylenediamine or hexylenediamine linkers (**30**–**33**). Interestingly, the syntheses of 3-carboranyl-1,8-naphthalimide derivatives through the formation of an amide bond using 3-amino-*N*-[2-(dimethylamino)ethyl]-1,8-naphthalimide or 3-amino-*N*-[2-(*N*-pyrrolidinyl)ethyl]-1,8-naphthalimide with 3-(1,2-dicarba-*closo*-dodecaboran-1-yl)propionic acid or 3-(1,7-dicarba-*closo*-dodecaboran-1-yl)propionic acid in the presence of anhydrous benzotriazol-1-yl-oxytripyrrolidinophosphonium hexafluorophosphate (PyBOP) and triethylamine (TEA) have been successfully developed. Methods for synthesizing 3-aminonaphthalimide derivatives bearing carborane clusters have been developed using the same *N*-succinimidyl active esters. However, the products were obtained after a prolonged synthesis time (4–10 days) at 37 °C with a maximum yield of 39% [9].

Reductive amination plays a vital role in pharmaceutical and medicinal chemistry owing to its synthetic merits and the ubiquitous presence of amines among biologically active compounds [31].

Treatment of *N*-[2-(dimethylamino)ethyl]-4-[(2-aminoethyl)amino]-1,8-naphthalimide (**30**), *N*-[2-(*N*-pyrrolidinyl)ethyl]-4-[(2-aminoethyl)amino]-1,8-naphthalimide (**31**), *N*-[2-(dimethylamino)ethyl]-4-[(6-aminohexyl)amino]-1,8-naphthalimide (**32**), or *N*-[2-(*N*-pyrrolidinyl)ethyl]-4-[(6-aminohexyl)amino]-1,8-naphthalimide (**33**) [28] with an appropriate aldehyde containing *ortho*-carborane **44** or *meta*-carborane **45** [32] in anhydrous THF (for the synthesis of compounds **46**, **48**, **50**, and **52**), anhydrous AcOEt (for the synthesis of compound **47**), or anhydrous MeOH (for the synthesis of compounds **49**, **51**, and **53**) at reflux under an inert (Ar) atmosphere yielded the corresponding Schiff bases and **46**–**53**, but these could not be isolated due to their instability (Scheme 5).

Compounds **54**–**61** were obtained by treating the modified Schiff bases **46**–**53** with NaBH_3_CN, followed by column chromatography. The structure, purity, and homogeneity of compounds **54**–**61** were confirmed by ^1^H-, ^13^C-, and ^11^B-NMR, FT-IR, MS, RP-HPLC (Figures S185–S248, ESI), and TLC.

In contrast to the synthesis of 3-aminonaphthalimide derivatives bearing the carborane group [9], the direct attachment of the aldehyde to the amino group at position 4 of the heterocyclic ring system proceeded with low yield (10%). Therefore, it was necessary to prepare suitable naphthalimide substrates containing ethylenediamine or hexylenediamine linkers.

#### 2.1.2. X-ray Structural Analysis

**Figure 2:**
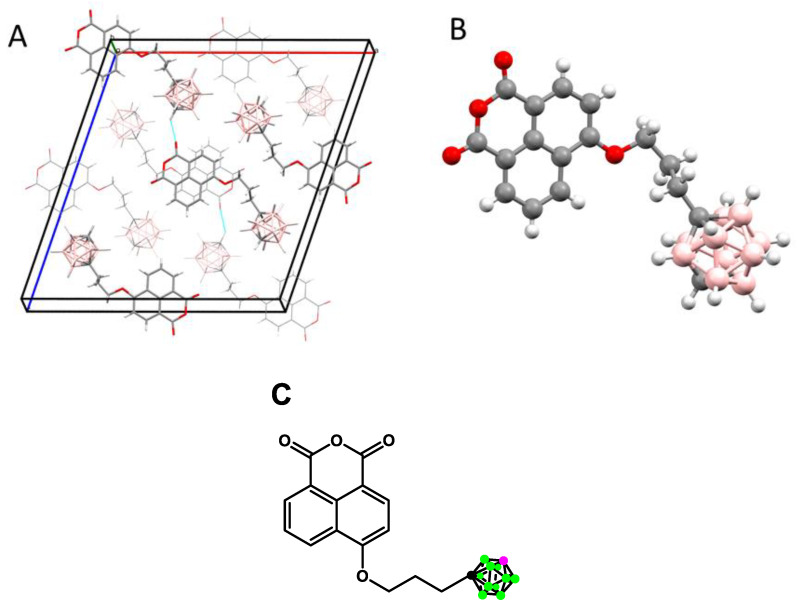
The crystallographic structures of **23**: (**A**) crystal packing within the unit cell and (**B**) the molecular structure. Hydrogen bonds are marked between the CH groups of the carborane clusters and the carbonyl oxygen atoms of the neighboring molecules (blue lines); (**C**) chemical structure of compound **23**.

Figure 2 shows the crystal structure of an asymmetric unit of one molecule of modified 1,8-naphthalic anhydride **23** (Figure 2). The packing of molecules in the crystal lattice involves extensive parallel stacking of the heteroaromatic ring systems, while the carborane clusters form separate zones. Notably, the CH group of the carborane clusters form weak hydrogen bonds with one of the carbonyl oxygen atoms of neighboring molecules, thus forming a crystal lattice network. The donor-acceptor distance is 3.16 Å, indicating a relatively strong bond for a carbon atom acting as a donor. This is another confirmation that the carborane groups can participate in weak H-bonding interactions [9,19], and the acidic nature of the C-H group was previously observed for free carboranes [33].

### 2.2. Physicochemical Investigation with DNA

#### 2.2.1. Melting Temperature Measurements

The melting temperature (*T*_m_) of DNA is defined as the temperature at which 50% of the DNA is in form of single strands forming randomly coiled structures [34]. DNA melting is measured by the absorbance of UV light (260 nm) by the DNA solution, where the amount of UV light absorbed is proportional to the fraction of non-bonded base pairs. The majority of DNA-binding small molecules known thus far stabilize duplex DNA against heat denaturation. A high, drug-induced increase in the *T*_m_ of DNA is generally viewed as an excellent criterion to select DNA ligands and is a common feature of several anticancer drugs such as intercalators (a significant increase in *T*_m_ = 3–8 °C is observed for strong intercalation) and alkylators. The reverse situation (destabilization of DNA to facilitate its denaturation) may be an attractive option to identify therapeutic agents acting on the DNA structure.

Our study conducted *T*_m_ measurement to estimate the impact of 1,8-naphthalimides bearing the carborane cluster on DNA stabilization (Table 1, Figures S249–S253, ESI).

Thermal melting experiments showed that the studied compounds **6**, **22**, **23**, and **41** caused destabilization of ct-DNA. Conjugates **7**–**15**, **17**–**19**, **25**, **27**, **38**–**40**, **42**, and **43** caused negligible stabilization of ct-DNA, while conjugates **16**, **24**, **26**, **36**, and **37** caused better DNA stabilization. It is worth mentioning that substituents in naphthalimide derivatives **8**–**11** and **16**–**19** were the same as those reported in a previous paper [9], with a difference in the position of the substituent (4-substituted vs. 3-substituted). A naphthalimide derivative can stabilize a DNA duplex to a varying degree depending on the substituent attached to the naphthalimide, the position of the substituent, and the sequence of DNA [35]. We observed that 4-substituted derivatives stabilize DNA slightly more than the corresponding 3-substituted naphthalimides. However, naphthalimide derivatives **54** and **61** modified with the carborane cluster through reductive amination revealed a considerable increase in melting temperature. The thermal denaturation experiment conducted for ct-DNA in the absence of the modified naphthalimide revealed a *T*_m_ value of 74 °C. In contrast, the observed *T*_m_ of ct-DNA in the presence of **54**–**61** significantly increased within the range of *T*_m_ values 81.67–86.33 °C (Δ*T*_m_ = 7.67–12.33 °C, Table 1) with the highest value for pinafide derivative **61** bearing *meta*-carborane and a (CH_2_)_6_ linker between modification and a 1,8-naphthalimide residue. In comparison to unmodified mitonafide and pinafide (Δ*T*_m_ = 5.17 and 6.50 °C, respectively) [9], this may confirm classical intercalation of the modified compounds as a dominant binding mode.

#### 2.2.2. Circular Dichroism Spectra Measurements

The rich photophysical properties of the 1,8-naphthalimides (which are highly dependent on the nature and substitution pattern of the aryl ring) make them prime candidates as probes for changes in spectroscopic properties such as dichroism, and absorption can be used to monitor their binding to biomolecules.

To understand the interactions of the modified 1,8-naphthalimides with DNA, circular dichroism (CD) measurement was conducted. CD is a technique to investigate the conformational changes in DNA morphology during its interaction with ligands. The CD spectra of the B-form DNA duplex generally display a positive Cotton effect at 270 nm and a negative effect at approximately 250 nm with nearly equal magnitudes of positive longwave bands and negative shortwave bands [36,37].

The CD spectrum of free ct-DNA showed a negative band at 248 nm due to polynucleotide helicity and a positive band at 276 nm due to base stacking, thus confirming the existence of ct-DNA in the right-handed B-form [38]. Treatment with mitonafide or pinafide decreased the negative peak and increased the positive peak [9]. As illustrated in Figures S254, S256–S262, S265, and S266 (ESI), conjugates bearing carborane clusters **6**, **7**, **11**, **14**–**19**, **22**–**27**, **41**, and **42** did not cause any appreciable change in the CD spectra of ct-DNA with an increase in concentration. For compounds **8**–**10**, **36**–**40**, and **43**, the positive and negative bands were perturbed by the presence of these compounds (Figures S255, S256, and S263–S266, ESI). Thus, simple groove binding and electrostatic interaction of ligands show less or no perturbation on base-stacking and helicity bands, while intercalation enhances the intensities of both the bands, stabilizing the right-handed B conformation of DNA, as observed for the classical intercalator methylene blue [39]. As depicted in Figures S267–S270 (ESI), the CD spectrum of ct-DNA was remarkably perturbed in the presence of conjugates **54**–**61**, resulting in the rapid increase in positive bands without any shift. Negative bands increased slightly with a slight red shift of their maximum. The obtained data for those compounds agree well with their melting curve results. The CD changes are reminiscent of destacking of the DNA base pairs and some degree of local helix destabilization consequent to intercalation of the 1,8-naphthalimide moiety. However, no conformational change from the B-form structure was observed.

#### 2.2.3. UV–Vis Spectra Measurement

The interaction of 1,8-naphthalimides bearing carborane clusters with ct-DNA was also studied by UV–vis absorption titration to better understand the mode of interaction and binding strength. For compounds interacting with DNA by intercalation, bathochromic and hypochromic effects are observed in the absorption spectra [40]. The spectral changes observed in the electronic absorption of **6**–**11**, **16**–**19**, **36**–**43**, and **54**–**61** in the absence and presence of ct-DNA are illustrated in Figures S271–S296 (ESI). Progressive addition of ct-DNA at the concentration of 1.25–15 µM to a fixed amount of modified naphthalic anhydride or naphthalimide (10 µM concentration) decreased absorbance for almost all the tested modified conjugates, except conjugates **14**, **15**, and **22**–**27** for which changes in absorbance were not observed. For all other conjugates, the absorption spectra demonstrated no bathochromic effect (**6**–**10**, **16**, **18**, **36**–**43**, **54**, **56**, **58**, **60**, and **61**) or slight bathochromic shifts (**11**, **17**, **19**, **55**, and **59**) (1–2 nm). It should be noted that mitonafide also caused a small bathochromic shift, which was confirmed in our study [9] and the literature [41]. Hypochromicity (10–56%) was also observed for all conjugates, with the highest value for compound **40**. The isosbestic point within 404–412 nm was observed for **6**, **7**, **11**, **17**, and **19**, indicating that ligand molecules are in two spectrophotometrically distinguished conditions—bound and free.

To compare the DNA-binding strength of the tested conjugates, we calculated the binding constant K*_b_*, as described in the Materials and Methods section. The selected modified compounds (**6**, **7**, **9**, **11**, **16**–**19**, **36**–**39**, **42**, **42**, and **54**–**61**) showed a similar K*_b_* value as compared to mitonafide (2.54 × 10^5^) [9], and some of the conjugates (**8**, **10**, **40**, and **41**) revealed a comparable K*_b_* value (Table 1) to that of pinafide (6.60 × 10^4^) [9]. For conjugates **14**, **15**, and **22**–**27**, the K*_b_* value could not be determined due to the lack of noticeable changes in the UV spectra.

### 2.3. Biological Investigation

#### 2.3.1. In Vitro Cytotoxic Activity

The obtained 1,8-naphthalimide–carborane conjugates were investigated for their in vitro antitumor activity by examining their cytotoxic effects using the MTT tetrazolium dye assay [42,43] against the human cancer cell line HepG2 established from hepatocellular carcinoma. IC_50_ refers to the drug concentration (µM) required to inhibit cell growth by 50%. The IC_50_ values determined for the synthesized compounds are summarized in Table 2.

Generally, 1,8-naphthalimides modified with carboranes (**8**–**11**, **16**–**19**, **24**–**27**, **36**–**43**, and **54**–**61**) exhibited more cytotoxicity than naphthalic anhydrides containing carborane clusters (**6**, **7**, **14**, **15**, **22**, and **23**). In compounds **8**–**11**, **16**–**19**, **24**–**27**, and **36**–**43**, the modified mitonafide derivatives were less cytotoxic than the modified pinafide derivatives, and conjugates modified with *ortho*-carboranes were less active than the corresponding conjugates modified with *meta*-carborane. Molecules synthesized via “click reaction” **8**–**11** (triazole ring attached directly to the heteroaromatic system) and **16**–**19** (triazole ring attached through an oxygen atom to the heteroaromatic system) showed similar cytotoxicity. A comparative analysis of the naphthalimides in the series in terms of their activity showed that these two groups of compounds were the least cytotoxic as compared to the modified 1,8-naphthalimides obtained using Mitsunobu reaction, amidation reaction, and reductive amination. Among the tested compounds, the most cytotoxic were conjugates **54**–**61** obtained by a reductive amination reaction. In this group of compounds, the conjugates containing a shorter linker between the heteroaromatic system and carborane modification (**54**–**57**) were slightly more cytotoxic than those containing a longer linker between the heteroaromatic system and carborane (**58**–**61**). The pinafide analog **57** containing *meta*-carborane was identified to be the most cytotoxic to the tested tumor cell line at a concentration as low as 3.12 µM. The pinafide analog **56** containing *ortho*-carborane was less cytotoxic with an IC_50_ value of 12.07 µM. The mitonafide analogs bearing *meta*-carborane **55** (IC_50_ = 3.43 µM) or *ortho*-carborane **54** (IC_50_ = 5.49 µM) were slightly less cytotoxic as compared to their pinafide analogs. It is worth mentioning that conjugates **54**–**61** were rather more active than the mitonafide and pinafide analogs also obtained through reductive amination but containing carborane clusters at position 3 of the heteroaromatic system (IC_50_ = 4.77–53.09 µM) [9]. Naphthalic anhydrides **6**, **14**, **15**, and **22** showed low cytotoxicity against the HepG2 cell line (IC_50_ = 28.65–71.19 µM), while compounds **7** and **23** were not toxic (IC_50_ > 100 µM).

Considering the physicochemical and cytotoxic properties of conjugates **54**–**61**, these compounds were used for further biological assays.

#### 2.3.2. Cell Migration Inhibition Assay

Cell migration is a vital process for various physiological and pathological conditions such as tissue homeostasis, immune response, cancer, and chronic inflammatory diseases [44].

**Figure 3:**
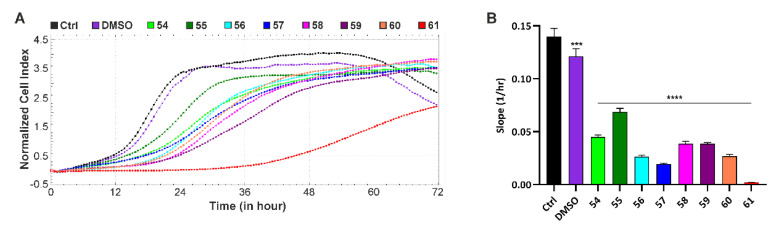
Real-time migration potential of HepG2 cells after 24 h administration of compounds **54**–**61**. The migration potential of the cells was monitored using the xCELLigence system. HepG2 cells were incubated with the analyzed compounds for 24 h before the analysis at the concentration corresponding to one-fourth of the IC_50_ value. The impedance (cell index values) was measured every 30 min for the next 72 h. To eliminate the influence of the solvent, DMSO was added to the cells in a volume that corresponded to the highest compound concentration (**A**). The slope parameter represents the rate of changes in CI values of HepG2 cells within the first 24 h (**B**). Statistical significance is indicated by asterisks: (***) *p* < 0.001, and (****) *p* < 0.0001.

By using the xCELLigence platform, we evaluated migration potential of HepG2 cells after the administration of compounds. We incubated the cells with compounds **54**–**61** at the concentration corresponding to one-fourth of the IC_50_ value for 24 h before the analysis, and the cells were then seeded on a CIM plate. Impedance-based cell index (CI) of HepG2 cells was measured every 30 min for 72 h. Our results indicate that all the analyzed compounds significantly affected the migration potential of HepG2 cells as compared to control. The most significant changes were observed within the first 24 h (Figure 3A). Thus, to obtain more insight into the altered migration profile caused by the analyzed compounds, we additionally calculated the slope representing the rate of change of the cell index within the first 24 h. Among the tested compounds, pinafide analogs bearing *ortho*- and *meta*-carborane clusters attached to heterocyclic rings through a (CH_2_)_2_ (**56**, **57**) or (CH_2_)_6_ linker (**60** and **61**) influenced the migratory activity most. However, the highest impairment was observed after treatment with conjugate **61** as compared to control cells (Figure 3B).

#### 2.3.3. Cell Cycle Analysis by Flow Cytometry

Recent preclinical and clinical studies of highly selective agents that target various regulators of the mammalian cell cycle demonstrate cell cycle arrest, inhibition of transcription, and apoptotic cell death in models of human cancer [45]. To reveal the mechanism underlying the inhibitory effect of the obtained compounds on cellular viability, we examined cell cycle regulation. For this purpose, HepG2 cells were exposed to compounds **54**–**61** (**54** (5.5 µM), **55** (3.4 µM), **56** (12 µM), **57** (3.1 µM), **58** (3.9 µM), **59** (5.4 µM), **60** (31 µM), and **61** (4.8 µM)). The chosen concentration of each of these compounds corresponded to their total IC_50_ value.

**Figure 4:**
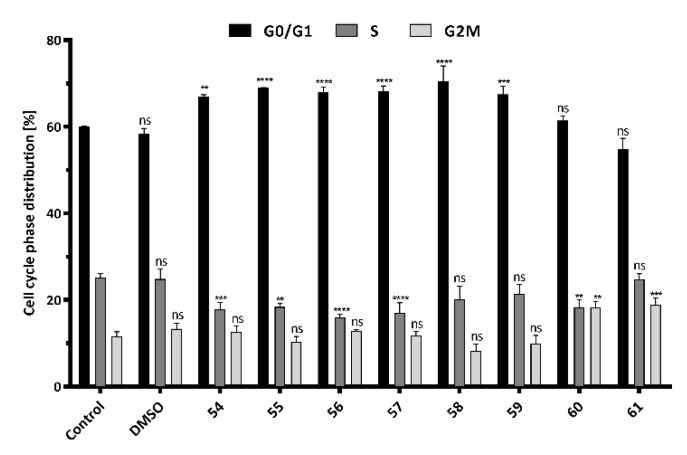
Effect of compounds **54** (5.5 µM), **55** (3.4 µM), **56** (12 µM), **57** (3.1 µM), **58** (3.9 µM), **59** (5.4 µM), **60** (31 µM), and **61** (4.8 µM) on cell cycle distribution in HepG2 cells. The cells were treated with these compounds at the concentration corresponding to the previously estimated IC_50_ value. The graph presents the percentage of cells in the G0/G1, S, and G2M phases, respectively. Data are presented as the mean ± SD of three independent experiments. Statistical significance is indicated by asterisks: (ns) *p* > 0.05, (**) *p* < 0.01, (***) *p* < 0.001, and (****) *p* < 0.0001.

After exposure, HepG2 cells were examined by flow cytometry, and their DNA content was measured by PI staining. Based on DNA content, it was found that all compounds affected the cell cycle, although the observed effect differed depending on the compound. Compounds **54**–**59** caused arrest in the G1/G0 phase since we observed a higher number of cells in that phase as compared to control (Figure 4 and Figure S297, ESI). The most significant effect on cell cycle progression was shown by compound **58**, where the G0/G1 cell fraction increased to 70.4% from 60.1% in control. Compounds **60** and **61** arrested cell cycle in the G2M phase, where the accumulation of cells was observed (>18% vs. 11.5% in control cells) (Figure 4 and Figure S297, ESI). In comparison, mitonafide and pinafide induced cell cycle arrest at the S and G2M phases [8], respectively. Our previous studies showed that 1,8-naphthalimide derivatives with carborane or metallacarborane modification at the *N*-imide position also caused cell cycle arrest at the G0/G1 phase [8]. In contrast, 1,8-naphthalimide products with carborane modification at position 3 caused cell cycle arrest at the G2M phase [9], similar to pinafide and compounds **60** and **61** from this study.

#### 2.3.4. Oxidative Stress Measurement in HepG2 Cells by Flow Cytometry

To reveal the mechanism responsible for the inhibitory effect of compounds **54**–**61** on cell viability, we examined their ability to induce ROS production. A previous study showed that 1,8-naphthalimide derivatives could elevate intracellular and mitochondrial ROS production and a remarkable activation of the p38 MAPK pathway [6].

**Figure 5:**
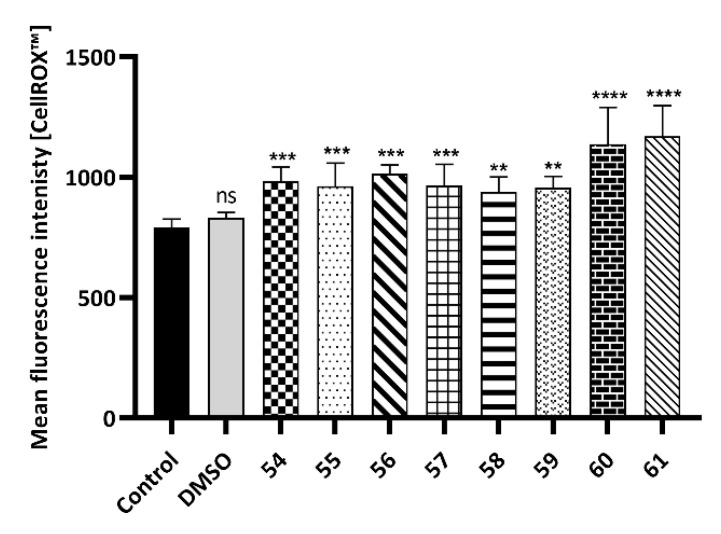
Intracellular ROS production in HepG2 cells after treatment with the analyzed compounds **54** (5.5 µM), **55** (3.4 µM), **56** (12 µM), **57** (3.1 µM), **58** (3.9 µM), **59** (5.4 µM), **60** (31 µM), and **61** (4.8 µM). The concentration chosen for each compound corresponded to the total IC_50_ value. Oxidative stress was evaluated using CellROX Deep Red Reagent by flow cytometry. Data are presented as the mean ± SD of three independent experiments. Statistical significance is indicated with asterisks: (ns) *p*  >  0.05, (**) *p*  <  0.01, (***) *p*  <  0.001, and (****) *p*  <  0.0001.

To determine the effect of the compounds on HepG2 cells, we analyzed oxidative status after 24 h of treatment as the pathological level of ROS affects the proper functioning of proteins, lipids, and nucleic acids. The compounds were added to the growth medium at a concentration that corresponds to the total IC_50_ value. After incubation, the cells were stained with CellROX Deep Red Reagent, which is an oxidative stress indicator. This is a non-fluorescent dye that emits red fluorescent signal upon oxidation by ROS. Flow cytometry analysis indicated that the most potent inductors of ROS were compounds **60** and **61** as the fluorescence intensity in HepG2 treated cells increased by 64% and 65%, respectively, as compared to control (Figure 5 and Figure S298, ESI). Conjugates **54**–**59** showed a moderate effect on ROS generation in HepG2 cells. We observed an increase in fluorescence intensity of approximately 22–32% as compared to that of control cells (Figure 5 and Figure S298, ESI).

#### 2.3.5. Analysis of 8-oxo-dG in HepG2 Cells

We also estimated 8-oxo-dG levels in DNA since 2-deoxyguanosine is most susceptible to oxidation among nucleosides and might serve as an oxidative DNA damage marker within the cells. HPLC-UV-ED analysis of DNA revealed that all compounds significantly increased 8-oxo-dG levels in HepG2 cells after 24 h of incubation. The most effective were compounds **60** and **61** as we observed over 57- and 43-fold increase, respectively, in the number of 8-oxo-dG residues per 10^6^ dG as compared to control (Table 3). Both compounds generate much higher oxidative disturbance in DNA than mitonafide, resulting in approximately 4- and 6-fold more 8-oxo-dG residues, respectively. Taken together, these results confirm that all the analyzed compounds affect oxidative balance in HepG2 cells and contribute significantly to oxidative stress generation.

#### 2.3.6. Apoptosis/Necrosis Assay by Flow Cytometry

Apoptosis and necrosis are two mechanisms involved in cell death in multicellular organisms. Apoptosis is considered as a naturally occurring physiological process, whereas necrosis is a pathological process caused by external agents such as toxins. In our studies on 1,8-naphthalimides modified with a carboranyl group in position 3, it was found that this type of conjugates strongly promoted apoptosis, which might inhibit the growth of HepG2 cells [9].

**Figure 6:**
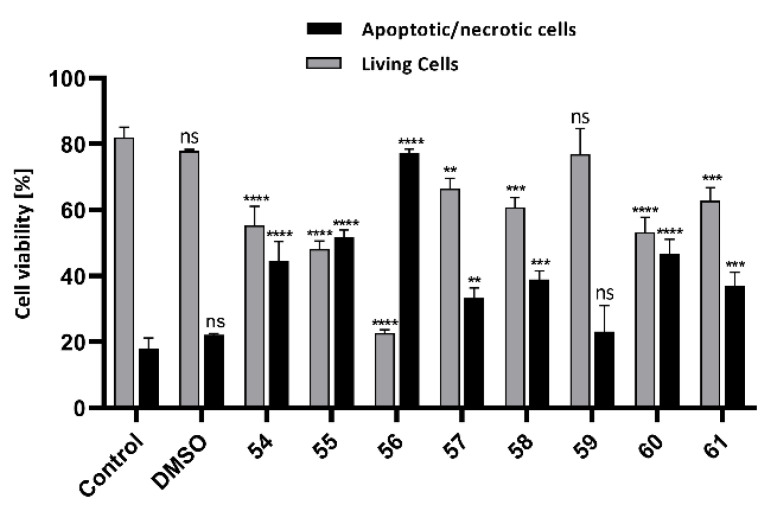
Effect of compounds **54** (5.5 µM), **55** (3.4 µM), **56** (12 µM), **57** (3.1 µM), **58** (3.9 µM), **59** (5.4 µM), **60** (31 µM), and **61** (4.8 µM) on cell death in HepG2 cells. The cells were incubated with the tested compounds for 24 h at the concentration corresponding to the total IC_50_ values. Quantitative flow cytometry analysis was performed to evaluate apoptosis/necrosis induced by compounds after 24 h of treatment. Data are presented as the mean ± SD of three independent experiments. Statistical significance is indicated with asterisks: (ns) *p*  >  0.05, (**) *p*  <  0.01, (***) *p*  <  0.001, and (****) *p*  <  0.0001.

To investigate whether compounds **54**–**61** induced apoptosis in HepG2 cells, we incubated the cells with these compounds for 24 h and performed a flow cytometry analysis. The concentration chosen for each compound corresponded to their total IC_50_ values. After incubation, the cells were stained with Annexin V Alexa Fluor 647 conjugate, which enabled detection of the externalization of phosphatidylserine, one of the earliest indicators of apoptosis. To eliminate the influence of the solvent (DMSO), we also analyzed its ability to induce apoptosis in a volume that corresponded to the highest concentration. All the studied compounds induced apoptosis in HepG2 cells after 24 h of treatment. The apoptosis rate is presented in Figure S299 (ESI). The most potent apoptosis inductor was compound **56**, where we observed 77.4% of apoptotic cells. In the cells treated with compounds **54**, **55**, and **60**, almost half of the test population underwent apoptosis, with the apoptosis rates of 44.3%, 48.2%, and 46.9%, respectively. The other compounds, **57**, **58**, **59**, and **61**, could not induce as high apoptosis as those mentioned above. Approximately one-third of cells targeted apoptotic pathways (33.4%, 39.1%, 23.1%, and 37.1%, respectively) when treated with these compounds (Figure 6).

#### 2.3.7. Fluorescence Imaging of Lysosomes

In recent years, considerable efforts have been made to develop 1,8-naphthalimide derivatives as fluorescent probes and fluorescent dyes. Many excellent examples of 1,8-naphthalimide-based probes have been reported, and some of them have been successfully applied in live-cell imaging research. A series of multifunctional compounds based on 1,8-naphthalimide moiety were designed and synthesized. They could be used as imaging reagents to detect lysosomes in live human cervical cancer cells (HeLa) by using fluorescence microscopy [46].

**Figure 7:**
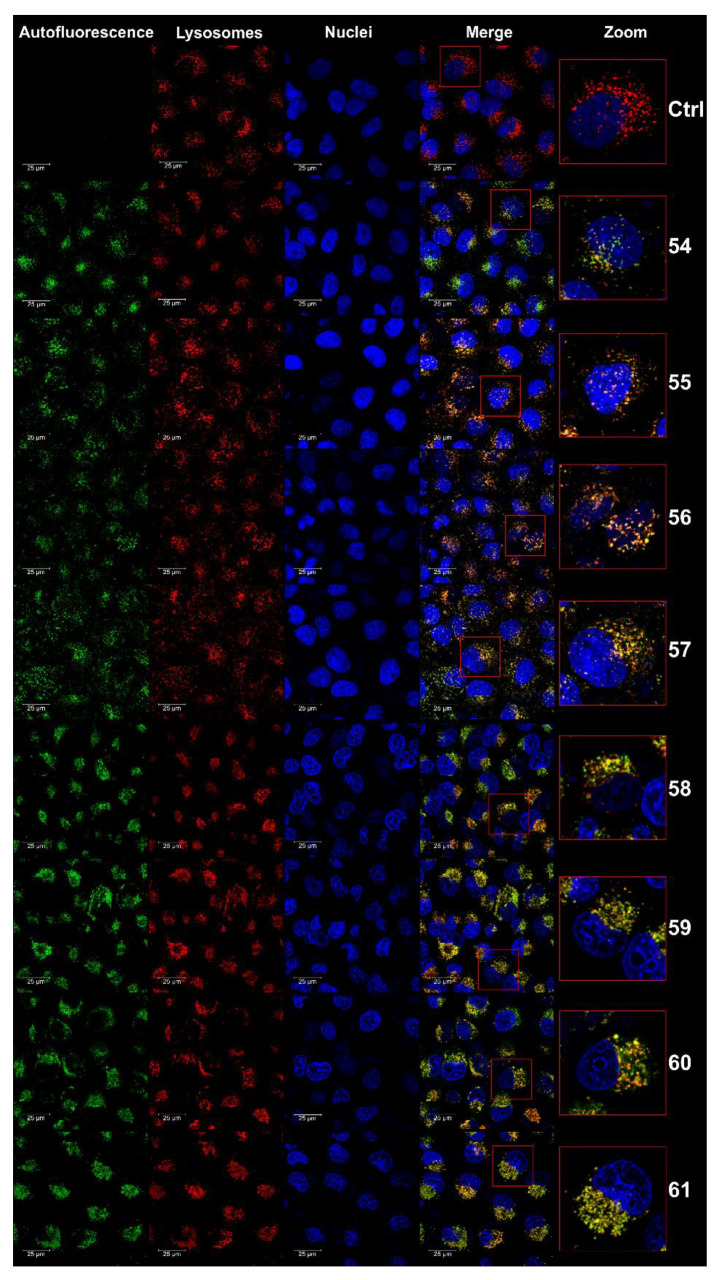
Colocalization analysis of compounds **54**–**61** in HepG2 cells by using confocal microscopy. This study was conducted after 24 h of treatment at the concentration corresponding to the total IC_50_ values. Untreated cells were used as a control. Panels with green fluorescence show the autofluorescence of the investigated compounds (Ex/Em 488/500–600 nm). Lysosomes were stained with LysoTracker Red DND-99 (Ex/Em 561/585–655 nm; red), nuclei were stained with Hoechst 33342 (Ex/Em 405/430–480 nm; blue). Merged images are shown in the right panel. Green/red fluorescence signal reveals the potential colocalization of lysosomes and the analyzed compounds.

Our previous studies showed that the selected 1,8-naphthalimides modified with the carboranyl group at position 3 specifically targeted the lysosomes of living cells with good cell membrane permeability, which enabled the localization of boron/carborane in the cells [9]. The observed autofluorescence of the analyzed compounds **54**–**61** also enabled us to determine intracellular localization of the analyzed compounds. After 24 h of incubation with the compounds at the concentration corresponding to the total IC_50_ value, the cells were stained with the fluorescent dyes that specifically stain cellular organelles. Hoechst 33342 was used to stain nuclei blue, whereas LysoTracker Red DND-99 stained lysosomes. The analyzed compounds emitted strong green fluorescent signals (left panel, Figure 7). Colocalization analysis by confocal microscopy revealed that the autofluorescence of the compounds overlapped with that of DND-99 (red). Because of the observed green and red fluorescence overlap, an orange signal appeared on merged images. These results suggest that the compounds can quickly diffuse through the membranes and can specifically target the lysosomes.

#### 2.3.8. Human Topoisomerase IIα Relaxation Assay

DNA topoisomerases are the enzymes that regulate DNA replication, transcription, and repair. Inhibitors of topoisomerases I and II (Topo I and II, respectively) are effectively used as anticancer agents [47]. Drugs targeting Topo II are divided into two broad classes. The first class, which includes most clinically active agents, leads to an increase in the levels of Topo II-DNA covalent complexes. These drugs have been termed as Topo II poisons. The second class of compounds inhibits Topo II catalytic activity but does not cause an increase in the levels of Topo II covalent complexes. Agents in this second class are thought to kill cells by eliminating the essential enzymatic activity of Topo II and are therefore termed as Topo II catalytic inhibitors. Topo II poisons can be subdivided into intercalating and non-intercalating poisons [48]. Inhibition of topoisomerases, especially Topo II, has been proposed as the primary mechanism by which naphthalimides induce cell cycle arrest and apoptosis in cancer cells [49].

In our earlier study, the presence of the carboranyl cluster at position 3 of 1,8-naphthalimide moieties did not promote them as effective Topo II inhibitors [9].

Based on this information, carborane cluster-modified naphthalic anhydrides (**6**, **7**, **14**, **15**, **22**, and **23**) and 1,8-naphthalimides (**8**–**11**, **16**–**19**, **24**–**27**, **36**–**43**, and **54**–**61**) were tested in the screening assay for human topoisomerase IIα inhibitory activity at the concentration of 200 µM (Figure S300, ESI). The inhibitory activity, which was manifested as the presence of the separated supercoiled fraction of pBR322 plasmid DNA on the agarose gel, was observed for compounds **6**–**8**, **11**–**15**, **22**, **23**, **25**, **26**, and **36**–**38**. Therefore, we subjected these compounds to further detailed analyses of inhibitory potential within the concentration range of 0.1–100 µM (Figure S301, ESI) to calculate IC_50_ values. IC_50_ refers to the compound concentration (µM) required to inhibit the activity of the topoisomerase enzyme by 50%. The IC_50_ values determined for the compounds listed above are summarized in Table 4.

Based on the results obtained, it was observed that compound **8** revealed inhibitory properties against the tested enzyme with the highest potency at the concentration as low as 0.58 µM. Conjugate **38** was almost 5-fold less active with an IC_50_ value of 2.81 µM. It is worth mentioning that compounds **8** and **38** were more active than mitonafide (IC_50_ = 5.13 µM). Promising inhibitory properties were also shown by compounds **6**, **7**, **36**, and **37** relative to the standard used (IC_50_ = 7.73, 8.78, 7.72, and 7.49 µM, respectively). Conjugate **15** was 12-fold less active than mitonafide (Table 4). Compounds **54**–**61**, which were characterized by their ability to stabilize DNA (Table 1), did not inhibit the activity of this enzyme. As described above, intercalation into DNA is not required to inhibit Topo II.

## 3. Materials and Methods

### 3.1. Chemistry

Most of the chemicals were obtained from the Acros Organics (Geel, Belgium) and were used without further purification unless otherwise stated. 4-Bromo-1,8-naphthalic anhydride (**1**) was obtained from the TCI (Tokyo, Japan) and used without further purification. Boron clusters were purchased from KATCHEM spol. S.r.o. (Řež/Prague, Czech Republic). All experiments that involved water-sensitive compounds were conducted under rigorously anhydrous conditions and under an argon atmosphere. Flash column chromatography was performed on silica gel 60 (230–400 mesh, Sigma-Aldrich, Steinheim, Germany). *R*_f_ refers to analytical TLC performed using pre-coated silica gel 60 F254 plates purchased from Sigma-Aldrich (Steinheim, Germany) and developed in the solvent system indicated. Compounds were visualized using UV light (254 nm) or a 0.5% acidic solution of PdCl_2_ in HCl/methanol by heating with a heat gun for boron-containing derivatives. The yields are not optimized.

^1^H NMR, ^13^C NMR, and ^11^B NMR spectra were recorded on a Bruker Avance III 600 MHz spectrometer equipped with a direct ATM probe. The spectra for ^1^H, ^13^C, and ^11^B nuclei were recorded at 600.26, 150.94, and 192.59 MHz, respectively. Deuterated solvents were used as standards. The following abbreviations are used to denote the multiplicities: s = singlet, d = doublet, dd = doublet of doublets, ddd = doublet of doublets of doublets, t = triplet, dt = doublet of triplets, q = quartet, quin = quintet, br s = broad singlet, and m = multiplet. *J* values are given in Hz.

Mass spectra were recorded on a CombiFlash PurIon Model Eurus35 (Teledyne ISCO, Lincoln, NE, USA). The ionization was achieved by Atmospheric-Pressure Chemical Ionization (APCI) ionization in the positive ion mode (APCI+) and negative ion mode (APCI–). The entire flow was directed to the APCI ion source operating in the positive ion mode. Total ion chromatograms were recorded in the *m*/*z* range of 100–700. The vaporization and capillary temperature were set at 250–400 and 200–300 °C, respectively. The capillary voltage of 150 V, corona discharge of 10 µA. High-resolution mass spectra (HRMS) were obtained on a Agilent 6546 LC/Q-TOF with ESI ion source spectrometer (Agilent Technologies, Inc., Santa Clara, CA, USA). The data are presented for the most abundant mass in the boron distribution plot of the base peak (100%) and for the peak corresponding to the highest *m*/*z* value with its relative abundance (%).

The theoretical molecular mass peaks of the compounds were calculated using the “Show Analysis Window” option in the ChemDraw Ultra 12.0 program. The calculated *m*/*z* corresponds to the average mass of the compounds consisting of natural isotopes.

Infrared absorption spectra (IR) were recorded using a Nicolet 6700 Fourier-transform infrared spectrometer from Thermo Scientific equipped with a ETC EverGlo* source for the IR range, a Geon-KBr beam splitter, and a DLaTGS/KBr detector with a smart orbit sampling compartment and diamond window. The samples were placed directly on the diamond crystal, and pressure was added to make the surface of the sample conform to the surface of the diamond crystal.

UV measurements were performed using a GBC Cintra10 UV–vis spectrometer (Dandenong, Australia). The samples used for the UV experiment were dissolved in 99.8% C_2_H_5_OH. The measurement was performed at ambient temperature.

RP-HPLC analysis was performed on a Hewlett-Packard 1050 system equipped with a UV detector and Hypersil Gold C18 column (4.6 × 250 mm, 5 μm particle size, Thermo Scientific, Runcorn, UK). UV detection was conducted at λ = 340 nm for compounds **6**–**11**, **14**–**19**, and **22**–**27** and at 380 nm for compounds **36**–**43** and **54**–**61**. The flow rate was 1 mL min^–1^. All analyses were run at ambient temperature. The gradient elution was as follows: gradient A—10 min from 30% to 55% A, 10 min from 55% to 90% A, and 10 min from 90% to 30% A. Buffer A contained 0.1% HCOOH in CH_3_CN, and buffer B contained 0.1% HCOOH in H_2_O; gradient B—10 min from 0% to 25% A, 10 min from 25% to 60% A and 10 min from 60% to 0% A. Buffer A—CH_3_CN contained 0.1% HCOOH, and buffer B—H_2_O contained 0.1% HCOOH. Crystals of **23** were obtained by slow evaporation from MeOH. X-ray diffraction measurements were carried out under cryogenic conditions on an XtaLab Synergy four-circle diffractometer (Oxford Diffraction) equipped with a Cu rotating anode PhotonJet X-ray source and HyPis-6000HE CCD detector. The data were processed with CRYSALISPRO software (Rigaku Oxford Diffraction), the structure was solved with SHELXT and refined with SHELXL programs, as above, via the Olex2 interface [50]. The refinement of atomic positions was unrestrained except for hydrogen atoms which were maintained at riding positions. Table S1 (ESI) summarizes the crystallographic data.

1-(3-Azidopropyl)-1,2-dicarba-*closo*-dodecaborane (**4**) 1-(3-azidopropyl)-1,7-dicarba-*closo*-dodecaborane (**5**) were synthesized as described in [24]. 4-Hydroxy-1,8-naphthalic anhydride (**12**) was synthesized as described in [26]. 1-(3-hydroxy-propyl)-1,2-dicarba-*closo*-dodecaborane (**20**) was obtained according to [27], and 1-(3-hydroxy-propyl)-1,7-dicarba-*closo*-dodecaborane (**21**) was obtained according to [28]. 4-Bromo-*N*-[2-(dimethylamino)ethyl]-1,8-naphthalimide (**28**) and 4-bromo-*N*-[2-(*N*-pyrrolidinyl)ethyl]-1,8-naphthalimide (**29**) were obtained by reaction of 4-bromo-1,8-naphthalic anhydride (**1**) with appropriate amine *N*,*N*-dimethylethylenediamine (for compound **28**) or *N*-(2-aminoethyl)pyrrolidine (for compound **29**) [51]. *N*-[2-(Dimethylamino)ethyl]-4-[(2-aminoethyl)amino]-1,8-naphthalimide (**30**), *N*-[2-(*N*-pyrrolidinyl)ethyl]-4-[(2-aminoethyl)amino]-1,8-naphthalimide (**31**), *N*-[2-(dimethylamino)ethyl]-4-[(6-aminohexyl)amino]-1,8-naphthalimide (**32**) and *N*-[2-(*N*-pyrrolidinyl)ethyl]-4-[(6-aminohexyl)amino]-1,8-naphthalimide (**33**) were synthesized as described in [30]. Active esters 3-(1,2-dicarba-*closo*-dodecaboran-1-yl)propionic acid *N*-succinimidyl ester (**34**), 3-(1,7-dicarba-*closo*-dodecaboran-1-yl)propionic acid *N*-succinimidyl ester (**35**) were prepared according to [18]. 2-(1,2-Dicarba-*closo*-dodecaboran-1-yl)ethanal (**44**) and 2-(1,7-dicarba-*closo*-dodecaboran-1-yl)ethanal (**45**) were synthesized as described in [32].

#### 3.1.1. Synthesis of 4-Ethynyl-1,8-Naphthalic Anhydride (**3**)

4-Bromo-1,8-naphthalic anhydride (**1**) (150 mg, 541.5 μmol), CuI (20.6 mg, 108 μmol), and Pd(PPh_3_)_4_ (62.4 mg, 54 μmol) were dried under vacuum overnight. Substrate **1** was dissolved in anhydrous DMF (7.5 mL), previously bubbled with dry argon. The obtained solution was added under argon to catalysts. Next, anhydrous TEA (150.5 μL, 1.08 mmol) and trimethylsilylacetylene (309 μL, 2.17 mmol) were added. The reaction mixture was stirred at 60 °C for 1 h, and the solvent was evaporated. 4-Trimethylsilylethynyl-1,8-naphthalic anhydride (**2**) was purified by column chromatography on silica gel (230–400 mesh) using CH_2_Cl_2_ as an eluent. Obtained product **2** (154 mg, 523.8 μmol) was dissolved in TFA (7 mL). The reaction mixture was stirred at 50 °C for 9 h, and solvents were evaporated. The residue was dissolved in CH_2_Cl_2_ (5 mL), and the solvent was evaporated; this procedure was repeated twice. Product **3** was purified by column chromatography on silica gel (230–400 mesh) using CH_2_Cl_2_ (100%) as an eluent to afford the product as a white solid. Yield: 99 mg (85%). TLC (CH_2_Cl_2_): *R*_f_ = 0.35; ^1^H NMR (CDCl_3_, 600.26 MHz): *δ* (ppm) = 8.78 (dd, *J* = 8.4, 1.2 Hz, 1H, H_arom_), 8.68 (dd, *J* = 7.3, 1.2 Hz, 1H, H_arom_), 8.58 (d, *J* = 7.6 Hz, 1H, H_arom_), 8.00 (d, *J* = 7.5 Hz, 1H, H_arom_), 7.91 (dd, *J* = 8.4, 7.2 Hz, 1H, H_arom_), and 3.83 (s, 1H, C-CH).

#### 3.1.2. 4-(Prop-2-yn-1-yloxy)-1,8-Naphthalic Anhydride (**13**)

4-Hydroxy-1,8-naphthalic anhydride (**12**) (82 mg, 383.2 μmol), propargyl alcohol (24.6 μL, 426.2 μmol), and triphenylphosphine (119.2 mg, 454.4 μmol) were suspended in anhydrous THF (1.1 mL). The suspension was cooled under dry argon to 0 °C, and a solution of DIAD (90.2 μL, 458.1 μmol) in anhydrous THF (5.5 mL) was added dropwise. The reaction mixture was stirred at RT for 72 h. Water (10 mL) was added, and THF was evaporated. Crude product **13** was extracted to CH_2_Cl_2_ (3 × 15 mL) and dried over MgSO_4_. The solvent was evaporated, and the product was purified twice by column chromatography on silica gel (230–400 mesh) using CHCl_3_ (100%) as an eluent. Next, the product was washed 3 times with cold ethanol (1 mL), centrifuged, and dried to afford the product as a white solid. Yield: 60 mg (62%). TLC (MeOH/CHCl_3_, 1:99, *v*/*v*): *R*_f_ = 0.50; ^1^H NMR (CDCl_3_, 600.26 MHz): *δ* (ppm) = 8.67 (dd, *J* = 8.5, 1.1 Hz, 1H, H_arom_), 8.63 (dd, *J* = 7.3, 1.1 Hz, 1H, H_arom_), 8.60 (d, *J* = 8.3 Hz, 1H, H_arom_), 7.78 (dd, *J* = 8.5, 7.3 Hz, 1H, H_arom_), 7.26–7.24 (m, 1H, H_arom_ overlapped with signal from CDCl_3_), 5.06 (d, *J* = 2.4 Hz, 2H, O-CH_2_), 2.66 (t, *J* = 2.4 Hz, 1H, C-CH).

#### 3.1.3. Synthesis of 1,8-Naphthalic Anhydride Derivatives **6** and **7** and 1,8-Naphthalimide Derivatives **8** and **11** Modified with Carborane Cluster via “Click Reaction”

1-(3-Azidopropyl)-carborane (*ortho*-carborane (**4**) or *meta*-carborane (**5**)) (1 equiv.) was dissolved in mixture THF/H_2_O (2.6:1, *v*/*v*, 3.9 mL per 0.1 mmol). 4-Ethynyl-1,8-naphthalic anhydride (**3**) (1 equiv.), CuSO_4_•5H_2_O (0.05 eqiuv.), sodium ascorbate (0.1 equiv.) and tris[((1-benzyl)-1*H*-1,2,3-triazol-4-yl)methyl]amine (0.05 equiv.) were added. The reaction mixture was stirred under dry argon for 2 h at 40 °C. The reaction was quenched by evaporation of solvents. The crude product was purified by column chromatography on silica gel (230–400 mesh) with a gradient of MeOH (0–10%) in CHCl_3_ as the eluent to afford the product. Additionally, product **6** was suspended in CHCl_3_ (4 mL) and poured into hexane (10 mL).

*4-{1-[3-(1,2-Dicarba-closo-dodecaborane-1-yl)propyl]-1H-1,2,3-triazol-4-yl}-1,8-naphthalic anhydride* (**6**): white solid, yield 64.3 mg (66%). TLC (MeOH/CH_2_Cl_2_, 1:99, *v/v*): *R*_f_ = 0.24; ^1^H NMR (DMSO-d_6_, 600.26 MHz): *δ* (ppm) = 9.24 (dd, *J* = 8.7, 0.9 Hz, 1H, H_arom_), 8.94 (s, 1H, CH_triazole_), 8.61–8.57 (m, 2H, 2H_arom_), 8.17 (d, *J* = 7.5 Hz, 1H, H_arom_), 7.98 (dd, *J* = 8.5, 7.3 Hz, 1H, H_arom_), 5.22 (br s, 1H, CH_carborane_), 4.52 (t, *J* = 7.0 Hz, 2H, CH_2_-triazole), 2.44–2.39 (m, 2H, CH_2_-carborane), 2.14 (dd, *J* = 16.6, 7.5 Hz, 2H, CH_2_-C*H*_2_-CH_2_), and 3.0–1.5 (m, 10H, B_10_H_10_); ^13^C NMR (DMSO-d_6_, 150.95 MHz): *δ* (ppm) = 160.84 (1C, C11), 160.51 (1C, C12), 144.36 (1C, C_triazole_), 135.04–118.50 (11C, 10C_arom_, CH_triazole_), 75.62 (1C, C_carborane_), 63.13 (1C, CH_carborane_), 48.73 (1C, CH_2_-triazole), 33.60 (1C, CH_2_-carborane), and 29.39 (1C, CH_2_-*C*H_2_-CH_2_); ^11^B NMR {^1^H BB} (DMSO-d_6_, 192.59 MHz): *δ* (ppm) = −3.25 (s, 1B, B9), −6.16 (s, 1B, B12), −9.79 (s, 2B, B8, 10), and −11.68 to −12.97 (m, 6B, B3, 4, 5, 6, 7, 11); UV (99.8% EtOH): λ_max_ (nm) = 241, 348, and 357, λ_min_ = 227 and 292, and λ_sh_ = 273; FT-IR: *ν*_max_ (cm^−1^) = 3063 (C-H_aliphat_), 2587 (B-H), 1769 (C=O), 1731 (C=O), and 727 (B-B); RP-HPLC (gradient A): *t*_R_ = 17.17 min; APCI-MS: *m/z*: 450 [M + H]^+^ and 482 [M+H+MeOH]^+^, calcd for C_19_H_23_B_10_N_3_O_3_: 449.

*4-{1-[3-(1,7-Dicarba-closo-dodecaborane-1-yl)propyl]-1H-1,2,3-triazol-4-yl}-1,8-naphthalic anhydride* (**7**): white solid, yield 67.5 mg (58%). TLC (MeOH/CH_2_Cl_2_, 1:99, *v/v*): *R*_f_ = 0.27; ^1^H NMR (CDCl_3_, 600.26 MHz): *δ* (ppm) = 9.22 (dd, *J* = 8.6, 1.0 Hz, 1H, H_arom_), 8.65 (dd, *J* = 7.3, 0.9 Hz, 1H, H_arom_), 8.62 (d, *J* = 7.5 Hz, 1H, H_arom_), 8.02 (s, 1H, CH_triazole_), 7.98 (d, *J* = 7.7 Hz, 1H, H_arom_), 7.86 (dd, *J* = 8.6, 7.2 Hz, 1H, H_arom_), 4.47 (t, *J* = 6.5 Hz, 2H, CH_2_-triazole), 2.96 (br s, 1H, CH_carborane_), 2.15–2.11 (m, 4H, CH_2_-C*H*_2_-CH_2_ overlapped with CH_2_-carborane), and 3.0–1.5 (m, 10H, B_10_H_10_); ^13^C NMR (CDCl_3_, 150.95 MHz): *δ* (ppm) = 160.72 (1C, C11), 160.49 (1C, C12), 145.83 (1C, C_triazole_), 135.71–118.53 (11C, 10C_arom_, CH_triazole_), 74.52 (1C, C_carborane_), 55.25 (1C, CH_carborane_), 49.90 (1C, CH_2_-triazole), 33.78 (1C, CH_2_-carborane), and 30.54 (1C, CH_2_-*C*H_2_-CH_2_); ^11^B NMR {^1^H BB} (CDCl_3_, 192.59 MHz): *δ* (ppm) = −4.19 (s, 1B, B5), −9.53 to −11.00 (m, 5B, B4, 6, 9, 10, 12), −13.40 (s, 2B, B8, 11), and −15.26 (s, 2B, B2, 3); UV (99.8% EtOH): λ_max_ (nm) = 241, 348, and 357, λ_min_ = 227 and 292, and λ_sh_ = 273; FT-IR: *ν*_max_ (cm^−1^) = 3065 (C-H_aliphat_), 2590 (B-H), 1769 (C=O), 1732 (C=O), and 727 (B-B); RP-HPLC (gradient A): *t*_R_ = 17.44 min; APCI-MS: *m/z*: 450 [M + H]^+^ and 482 [M+H+MeOH]^+^, calcd for C_19_H_23_B_10_N_3_O_3_: 449.

4-{1-[3-(1,2-Dicarba-*closo*-dodecaborane-1-yl)propyl]-1*H*-1,2,3-triazol-4-yl}-1,8-naphthalic anhydride (**6**) or 4-{1-[3-(1,7-dicarba-*closo*-dodecaborane-1-yl)propyl]-1*H*-1,2,3-triazol-4-yl}-1,8-naphthalic anhydride (**7**) was suspended in absolute EtOH (5 mL per 0.13 mmol) and *N*,*N*-dimethylethylenediamine (1.7 equiv.) or *N*-(2-aminoethyl)pyrrolidine (1.7 equiv.) was added. The reaction mixture was stirred for 2 h in 45 °C under an inert (Ar) atmosphere. Subsequently, the solvent was evaporated to dryness under vacuum and crude product was purified by column chromatography on silica gel (230–400 mesh) with a gradient of MeOH (3–13%) in CHCl_3_.

*N-[2-(Dimethylamino)ethyl]-4-{1-[3-(1,2-dicarba-closo-dodecaborane-1-yl)propyl]-1H-1,2,3-triazol-4-yl}-1,8-naphthalimide* (**8**): beige solid, yield 16.0 mg (46%). TLC (MeOH/CH_2_Cl_2_, 1:9, *v/v*): *R*_f_ = 0.36. ^1^H NMR (CDCl_3_, 600.26 MHz): *δ* (ppm) = 8.98 (d, *J* = 8.5 Hz, 1H, H_arom_), 8.62 (d, *J* = 7.2 Hz, 1H, H_arom_), 8.59 (d, *J* = 7.5 Hz, 1H, H_arom_), 7.97 (s, 1H, CH_triazole_), 7.90 (d, *J* = 7.5 Hz, 1H, H_arom_), 7.78 (t, *J* = 7.9 Hz, 1H, H_arom_), 4.51 (t, *J* = 6.7 Hz, 2H, CH_2_-triazole), 4.34 (t, *J* = 7.1 Hz, 2H, CH_2_-N(CO)_2_), 3.66 (br s, 1H, CH_carborane_), 2.70 (t, *J* = 7.0 Hz, 2H C*H*_2_-N(CH_3_)_2_), 2.41–2.39 (m, 8H, N(CH_3_)_2_ overlapped with CH_2_-carborane), 2.27 (dd, *J* = 10.4, 5.9 Hz, 2H, CH_2_-C*H*_2_-CH_2_), and 3.0–1.5 (m, 10H, B_10_H_10_); ^13^C NMR (CDCl_3_, 150.95 MHz): *δ* (ppm) = 164.30 (1C, C11), 164.02 (1C, C12), 146.42 (1C, C_triazole_), 133.89–122.72 (10C_arom_ + CH_triazole_), 73.67 (1C, C_carborane_), 61.87 (1C, CH_carborane_), 57.02 (1C, *C*H_2_-N(CH_3_)_2_), 49.45 (1C, CH_2_-triazole), 45.80 (2C, 2 × CH_3_), 38.25 (1C, *C*H_2_-N(CO)_2_), 35.18 (1C, CH_2_-carborane), and 29.93 (1C, CH_2_-*C*H_2_-CH_2_); ^11^B NMR {^1^H BB} (CDCl_3_, 192.59 MHz): *δ* (ppm) = −2.10 (s, 1B, B9), −5.41 (s, 1B, B12), −9.11 (s, 2B, B8, 10), and −11.91 to −12.91 (m, 6B, B3, 4, 5, 6, 7, 11); UV (99.8% EtOH): λ_max_ (nm) = 237 and 359, and λ_min_ = 221 and 286; FT-IR: *ν*_max_ (cm^−1^) = 2925 (C-H_aliphat_), 2573 (B-H), 1696 (C=O), 1651 (C=O), and 722 (B-B); RP-HPLC (gradient B): *t*_R_ = 21.50 min; APCI-MS: *m/z*: 520 [M + H]^+^, calcd for C_23_H_33_B_10_N_5_O_2_: 519.

*N-[2-(N-Pyrrolidinyl)ethyl]-4-{1-[3-(1,2-dicarba-closo-dodecaborane-1-yl)propyl]-1H-1,2,3-triazol-4-yl}-1,8-naphthalimide* (**9**): beige solid, yield 15 mg (41%). TLC (MeOH/CH_2_Cl_2_, 1:9, *v*/*v*): *R*_f_ = 0.34. ^1^H NMR (DMSO-d_6_, 600.26 MHz): δ (ppm) = 9.12 (dd, *J* = 8.5, 0.9 Hz, 1H, H_arom_), 8.88 (s, 1H, CH_triazole_), 8.58–8.51 (m, 2H, 2H_arom_), 8.11 (d, *J* = 7.7 Hz, 1H, H_arom_), 7.91 (dd, *J* = 8.5, 7.3 Hz, 1H, H_arom_), 5.23 (br s, 1H, CH_carborane_), 4.51 (t, *J* = 7.0 Hz, 2H, CH_2_-triazole), 4.18 (t, *J* = 7.2 Hz, 2H, CH_2_-N(CO)_2_), 2.71 (br s, 2H, CH_2_-pyrrolidine), 2.56 (br s, 4H, N-C*H*_2pyrrolidine_-CH_2_), 2.43–2.40 (m, 2H, CH_2_-carborane), 2.17–2.10 (m, 2H, CH_2_-C*H*_2_-CH_2_), 1.67 (br s, 4H, CH_2_-C*H*_2pyrrolidine_-CH_2_), and 3.0–1.5 (m, 10H, B_10_H_10_); ^13^C NMR (DMSO-d_6_, 150.95 MHz): δ (ppm) = 163.39 (1C, C11), 163.07 (1C, C12), 144.60 (1C, C_triazole_), 134.07–121.54 (11C, 10C_arom_ + CH_triazole_), 75.63 (1C, C_carborane_), 63.13 (1C, CH_carborane_), 53.79 (2C, N-*C*H_2pyrrolidine_-CH_2_), 53.00 (1C, CH_2_-pyrrolidine), 48.69 (1C, CH_2_-triazole), 38.64 (1C, *C*H_2_-N(CO)_2_), 33.61 (1C, CH_2_-carborane), 29.43 (1C, CH_2_-*C*H_2_-CH_2_), and 23.20 (2C, CH_2_-*C*H_2pyrrolidine_-CH_2_); ^11^B NMR {1H BB} (DMSO-d_6_, 192.59 MHz): δ (ppm) = −3.27 (s, 1B, B9), −6.24 (s, 1B, B12), and −9.85 to −13.01 (m, 8B, B3, 4, 5, 6, 7, 8, 10, 11); UV (99.8% EtOH): λ_max_ (nm) = 237 and 358, and λ_min_ = 221 and 288; FT-IR: ν_max_ (cm^−1^) = 2951 (C-H_aliphat_.), 2580 (B-H), 1697 (C=O), 1655 (C=O), and 725 (B-B); RP-HPLC (gradient B): t_R_ = 23.07 min; APCI-MS: *m*/*z*: 547 [M + H]^+^, calcd for C_25_H_35_B_10_N_5_O_2_: 546.

*N-[2-(Dimethylamino)ethyl]-4-{1-[3-(1,7-dicarba-closo-dodecaborane-1-yl)propyl]-1H-1,2,3-triazol-4-yl}-1,8-naphthalimide* (**10**): beige solid, yield 33.8 mg (87%). TLC (MeOH/CH_2_Cl_2_, 1:9, *v/v*): *R*_f_ = 0.37. ^1^H NMR (CDCl_3_, 600.26 MHz): *δ* (ppm) = 9.00 (dd, *J* = 8.7, 0.9 Hz, 1H, H_arom_), 8.64 (dd, *J* = 7.2, 0.9 Hz, 1H, H_arom_), 8.62 (d, *J* = 7.7 Hz, 1H, H_arom_), 7.95–7.91 (m, 2H, CH_triazole_ overlapped with H_arom_), 7.79 (dd, *J* = 8.5, 7.2 Hz, 1H, H_arom_), 4.45 (t, *J* = 6.4 Hz, 2H, CH_2_-triazole), 4.35 (t, *J* = 7.1 Hz, 2H, CH_2_-N(CO)_2_), 2.95 (s, 1H, CH_carborane_), 2.71–2.67 (m, 2H, C*H*_2_-N(CH_3_)_2_), 2.37 (s, 6H, N(CH_3_)_2_), 2.14–2.09 (m, 4H, CH_2_-C*H*_2_-CH_2_ overlapped with CH_2_-carborane), and 3.0–1.5 (m, 10H, B_10_H_10_); ^13^C NMR (CDCl_3_, 150.95 MHz): *δ* (ppm) = 164.36 (1C, C11), 164.08 (1C, C12), 146.30 (1C, C_triazole_), 134.13–122.69 (11C, 10C_arom_ + CH_triazole_), 74.55 (1C, C_carborane_), 57.07 (1C, *C*H_2_-N(CH_3_)_2_), 55.24 (1C, CH_carborane_), 49.79 (1C, CH_2_-triazole), 45.89 (2C, 2 × CH_3_), 38.33 (*C*H_2_-N(CO)_2_), 33.79 (CH_2_-carborane), and 30.57 (CH_2_-*C*H_2_-CH_2_); ^11^B NMR {^1^H BB} (CDCl_3_, 192.59 MHz): *δ* (ppm) = −4.22 (s, 1B, B5), −9.60 to −11.02 (m, 5B, B4, 6, 9, 10, 12), −13.42 (s, 2B, B8, 11), and −15.29 (s, 2B, B2, 3); UV (99.8% EtOH): λ_max_ (nm) = 237 and 358, and λ_min_ = 285; FT-IR: *ν*_max_ (cm^−1^) = 2944 (C-H_aliphat_), 2592 (B-H), 1697 (C=O), 1654 (C=O), and 729 (B-B); RP-HPLC (gradient B): *t*_R_ = 21.45 min; APCI-MS: *m/z*: 520 [M + H]^+^, calcd for C_23_H_33_B_10_N_5_O_2_: 519.

*N-[2-(N-Pyrrolidinyl)ethyl]-4-{1-[3-(1,7-dicarba-closo-dodecaborane-1-yl)propyl]-1H-1,2,3-triazol-4-yl}-1,8-naphthalimide* (**11**): beige solid, yield 36.7 mg (91%). TLC (MeOH/CH_2_Cl_2_, 1:9, *v*/*v*): *R*_f_ = 0.36. ^1^H NMR (CDCl_3_, 600.26 MHz): δ (ppm) = 9.00 (dd, *J* = 8.7, 0.9 Hz, 1H, H_arom_), 8.63 (dd, *J* = 7.2, 0.9 Hz, 1H, H_arom_), 8.61 (d, *J* = 7.5 Hz, 1H, H_arom_), 7.96–7.90 (m, 2H, CH_triazole_ overlapped with H_arom_), 7.79 (dd, *J* = 8.5, 7.3 Hz, 1H, H_arom_), 4.45 (t, *J* = 6.5 Hz, 2H, CH_2_-triazole), 4.38 (t, *J* = 7.2 Hz, 2H, CH_2_-N(CO)_2_), 2.95 (br s, 1H, CH_carborane_), 2.87–2.83 (m, 2H, CH_2_-pyrrolidine), 2.70 (br s, 4H, N-C*H*_2pyrrolidine_-CH_2_), 2.13–2.11 (m, 4H, CH_2_-C*H*_2_-CH_2_ overlapped with CH_2_-carborane), 1.83–1.78 (m, 4H, CH_2_-C*H*_2pyrrolidine_-CH_2_), and 3.0–1.5 (m, 10H, B_10_H_10_); ^13^C NMR (CDCl_3_, 150.95 MHz): δ (ppm) = 164.29 (1C, C11), 164.01 (1C, C12), 146.29 (1C, C_triazole_), 134.13–122.70 (11C, 10C_arom_ + CH_triazole_), 74.56 (1C, C_carborane_), 55.23 (1C, CH_carborane_), 54.50 (2C, N-*C*H_2pyrrolidine_-CH_2_), 53.73 (1C, CH_2_-pyrrolidine), 49.78 (1C, CH_2_-triazole), 39.32 (1C, *C*H_2_-N(CO)_2_), 33.78 (1C, CH_2_-carborane), 30.56 (1C, CH_2_-*C*H_2_-CH_2_), and 23.73 (2C, CH_2_-*C*H_2pyrrolidine_-CH_2_); ^11^B NMR {^1^H BB} (CDCl_3_, 192.59 MHz): δ (ppm) = −4.23 (s, 1B, B5), −9.59 to −11.04 (m, 5B, B4, 6, 9,10, 12), −13.44 (s, 2B, B8, 11), and −15.30 (s, 2B, B2, 3); UV (99.8% EtOH): λ_max_ (nm) = 237 and 359, and λ_min_ = 221 and 288; FT-IR: ν_max_ (cm^−1^) = 2948 (C-H_aliphat_), 2592 (B-H), 1697 (C=O), 1655 (C=O), and 729 (B-B); RP-HPLC (gradient B): t_R_ = 22.65 min; APCI-MS: *m*/*z*: 547 [M + H]^+^, calcd for C_25_H_35_B_10_N_5_O_2_: 546.

#### 3.1.4. Synthesis of 1,8-Naphthalic Anhydride Derivatives **14** and **15** and 1,8-Naphthalimide Derivatives **16** and **19** Modified with Carborane Cluster via “Click Reaction”

1-(3-Azidopropyl)-*ortho*-carborane (**4**) or 1-(3-azidopropyl)-*meta*-carborane (**5**) (1 equiv.) was dissolved in THF/H_2_O (2.4;1, *v/v*, 2.6 mL per 0.1 mmol). 4-(Prop-2-yn-1-yloxy)-1,8-naphthalic anhydride (**13**) (1 equiv.), copper (II) sulfate pentahydrate (0.05 equiv.) and sodium ascorbate (0.1 equiv.) were added. The reaction mixture was stirred under dry argon for 24 h at 40 °C. The reaction was quenched by evaporation of the solvents. The crude compound was purified by column chromatography on silica gel (230–400 mesh) with CHCl_3_ (100%) as an eluent to afford the product.

*4-{1-[3-(1,2-Dicarba-closo-dodecaborane-1-yl)propyl]-1H-1,2,3-triazol-4-yl}methoxy-1,8-naphthalic anhydride* (**14**): white solid, yield 57 mg (75%). TLC (MeOH/CH_2_Cl_2_, 1:99, *v/v*): *R*_f_ = 0.13. ^1^H NMR (acetone-d_6_, 600.26 MHz): *δ* (ppm) = 8.61 (dd, *J* = 8.4, 1.2 Hz, 1H, H_arom_), 8.58–8.53 (m, 2H, 2H_arom_), 8.33 (s, 1H, CH_triazole_), 7.85 (dd, *J* = 8.4, 7.2 Hz, 1H, H_arom_), 7.62 (d, *J* = 8.3 Hz, 1H, H_arom_), 5.64 (s, 2H, O-CH_2_-triazole), 4.70 (br s, 1H, CH_carborane_), 4.53 (t, *J* = 6.9 Hz, 2H, CH_2_-triazole), 2.47–2.43 (m, 2H, CH_2_-carborane), 2.23–2.17 (m, 2H, CH_2_-C*H*_2_-CH_2_), and 3.0–1.5 (m, 10H, B_10_H_10_); ^13^C NMR (acetone-d_6_, 150.95 MHz): *δ* (ppm) = 161.95–161.01 (3C, C11 + C12 + C4), 143.17 (C_triazole_), 136.13–108.57 (10C, 9C_arom_ + CH_triazole_), 76.20 (1C, C_carborane_), 63.87 (1C, O-CH_2_-triazole), 63.46 (1C, CH_carborane_), 49.64 (1C, CH_2_-triazole), 35.21 (1C, CH_2_-carborane), and 30.70 (1C, CH_2_-*C*H_2_-CH_2_); ^11^B NMR {^1^H BB} (acetone-d_6_, 192.59 MHz): *δ* (ppm) = −2.88 (s, 1B, B9), −5.98 (s, 1B, B12), −9.64 (s, 2B, B8, 10), and −11.64 to −13.03 (m, 6B, B3, 4, 5, 6, 7, 11); UV (99.8% EtOH): λ_max_ (nm) = 248 and 358, λ_min_ = 229 and 290, and λ_sh_ = 272; FT-IR: *ν*_max_ (cm^−1^) = 3073 (C-H_aliphat_), 2576 (B-H), 1764 (C=O), 1732 (C=O), and 722 (B-B); RP-HPLC (gradient A): *t*_R_ = 18.25 min; APCI-MS: *m/z*: 480 [M + H]^+^, 513 [M+H+MeOH]^+^, and 522 [M+H+CH_3_CN]^+^, calcd for C_20_H_25_B_10_N_3_O_4_: 479.

*4-{1-[3-(1,7-Dicarba-closo-dodecaborane-1-yl)propyl]-1H-1,2,3-triazol-4-yl}methoxy-1,8-naphthalic anhydride* (**15**): white solid, yield 42 mg (88%). TLC (MeOH/CH_2_Cl_2_, 1:99, *v/v*): *R*_f_ = 0.17. ^1^H NMR (acetone-d_6_, 600.26 MHz): *δ* (ppm) = 8.60 (dd, *J* = 8.5, 1.2 Hz, 1H, H_arom_), 8.58–8.52 (m, 2H, 2H_arom_), 8.32 (s, 1H, CH_triazole_), 7.84 (dd, *J* = 8.4, 7.3 Hz, 1H, H_arom_), 7.61 (d, *J* = 8.4 Hz, 1H, H_arom_), 5.64 (s, 2H, O-CH_2_-triazole), 4.48 (t, *J* = 6.5 Hz, 2H, CH_2_-triazole), 3.66 (br s, 1H, CH_carborane_), 2.09–2.06 (m, 4H, CH_2_-carborane and CH_2_-C*H*_2_-CH_2_ overlapped with signal from acetone-d_6_), and 3.0–1.5 (m, 10H, B_10_H_10_); ^13^C NMR (acetone-d_6_, 150.95 MHz): *δ* (ppm) = 161.94–160.99 (3C, C11 + C12 + C4), 143.09 (1C, C_triazole_), 136.12–108.56 (10C, 9C_arom_ + CH_triazole_), 76.53 (1C, C_carborane_), 63.85 (1C, O-CH_2_-triazole), 56.79 (1C, CH_carborane_), 49.80 (1C, CH_2_-triazole), 34.24 (1C, CH_2_-carborane), and 31.36 (1C, CH_2_-*C*H_2_-CH_2_); ^11^B NMR {^1^H BB} (acetone-d_6_, 192.59 MHz): *δ* (ppm) = −4.40 (s, 1B, B5), −9.90 to −11.02 (m, 5B, B4, 6, 9, 10, 12), −13.54 (s, 2B, B8, 11), and −15.10 (s, 2B, B2, 3); UV (99.8% EtOH): λ_max_ (nm) = 248 and 358, λ_min_ = 228 and 290, and λ_sh_ = 272; FT-IR: *ν*_max_ (cm^−1^) = 3065 (C-H_aliphat_), 2598 (B-H), 1766 (C=O), 1716 (C=O), and 727 (B-B); RP-HPLC (gradient A): *t*_R_ = 18.01 min; APCI-MS: *m/z*: 480 [M + H]^+^ and 513 [M+H+MeOH]^+^, calcd for C_20_H_25_B_10_N_3_O_4_: 479.

4-{1-[3-(1,2-Dicarba-*closo-*dodecaborane-1-yl)propyl]-*1H*-1,2,3-triazol-4-yl}methoxy-1,8-naphthalic anhydride (**14**) or 4-{1-[3-(1,7-dicarba-*closo*-dodecaborane-1-yl)propyl]-1*H*-1,2,3-triazol-4-yl}methoxy-1,8-naphthalic anhydride (**15**) was suspended in absolute EtOH (6 mL per 0.1 mmol) and *N*,*N*-dimethylethylenediamine (1.7 equiv.) or *N*-(2-aminoethyl)pyrrolidine (1.7 equiv.) was added. The reaction mixture was stirred for 3–4 h in 40 °C under an inert (Ar) atmosphere. Subsequently, the solvent was evaporated to dryness under vacuum and crude product was purified by column chromatography on silica gel (230–400 mesh) with a gradient of MeOH (0–14%) in CHCl_3_.

*N-[2-(Dimethylamino)ethyl]-4-{1-[3-(1,2-dicarba-closo-dodecaborane-1-yl)propyl]-1H-1,2,3-triazol-4-yl}methoxy-1,8-naphthalimide* (**16**): yellow solid, yield 15 mg (69%). TLC (MeOH/CH_2_Cl_2_, 1:9, *v*/*v*): *R*_f_ = 0.26. ^1^H NMR (acetone-d_6_, 600.26 MHz): δ (ppm) = 8.56–8.50 (m, 3H, 3H_arom_), 8.30 (s, 1H, CH_triazole_), 7.79 (dd, *J* = 8.4, 7.2 Hz, 1H, H_arom_), 7.54 (d, *J* = 8.3 Hz, 1H, H_arom_), 5.60 (s, 2H, O-CH_2_-triazole), 4.69 (br s, 1H, CH_carborane_), 4.52 (t, *J* = 6.8 Hz, 2H, CH_2_-triazole), 4.24 (t, *J* = 7.0 Hz, 2H, CH_2_-N(CO)_2_), 2.57 (t, *J* = 7.0 Hz, 2H, C*H*_2_-N(CH_3_)_2_), 2.46–2.41 (m, 2H, CH_2_-carborane), 2.26 (s, 6H, N(CH_3_)_2_), 2.22–2.16 (m, 2H, CH_2_-C*H*_2_-CH_2_), and 3.0–1.5 (m, 10H, B_10_H_10_); ^13^C NMR (acetone-d_6_, 150.95 MHz): δ (ppm) = 164.69–160.20 (3C, C11 + C12 + C4), 143.45 (1C, C_triazole_), 133.82–108.00 (10C, 9C_arom_ + CH_triazole_), 76.21 (1C, C_carborane_), 63.60 (1C, O-CH_2_-triazole), 63.46 (1C, CH_carborane_), 57.77 (1C, *C*H_2_-N(CH_3_)_2_), 49.62 (1C, CH_2_-triazole), 46.01 (2C, 2 × CH_3_), 38.57 (1C, *C*H_2_-N(CO)_2_), 35.21 (1C, CH_2_-carborane), and 30.69 (1C, CH_2_-*C*H_2_-CH_2_); ^11^B NMR {^1^H BB} (acetone-d_6_, 192.59 MHz): δ (ppm) = −2.87 (s, 1B, B9), −6.03 (s, 1B, B12), −9.66 (s, 2B, B8, 10), and −11.61 to −13.03 (m, 6B, B3, 4, 5, 6, 7, 11); UV (99.8%EtOH): λ_max_ (nm) = 243 and 363, λ_min_ = 226 and 283, and λ_sh_ = 298; FT-IR: ν_max_ (cm^−1^) = 2948 (C-H_aliphat_), 2565 (B-H), 1692 (C=O), 1647 (C=O), and 722 (B-B); RP-HPLC (gradient B): t_R_ = 21.11 min; APCI-MS: *m*/*z*: 550 [M + H]^+^, calcd for C_24_H_35_B_10_N_5_O_3_: 549.

*N-[2-(N-Pyrrolidinyl)ethyl]-4-{1-[3-(1,2-dicarba-closo-dodecaborane-1-yl)propyl]-1H-1,2,3-triazol-4-yl}methoxy-1,8-naphthalimide* (**17**): yellow solid, yield 10 mg (53%). TLC (MeOH/CH_2_Cl_2_, 1:9, *v*/*v*): *R*_f_ = 0.30. ^1^H NMR (acetone-d_6_, 600.26 MHz): δ (ppm) = 8.52–8.45 (m, 3H, 3H_arom_), 8.31 (s, 1H, CH_triazole_), 7.75 (dd, *J* = 8.4, 7.2 Hz, 1H, H_arom_), 7.49 (d, *J* = 8.4 Hz, 1H, H_arom_), 5.58 (s, 2H, O-CH_2_-triazole), 4.69 (br s, 1H, CH_carborane_), 4.52 (t, *J* = 6.9 Hz, 2H, CH_2_-triazole), 4.25 (t, *J* = 7.1 Hz, 2H, CH_2_-N(CO)_2_), 2.75 (t, *J* = 7.2 Hz, 2H, CH_2_-pyrrolidine), 2.59 (br s, 4H, N-C*H*_2pyrrolidine_-CH_2_), 2.46–2.42 (m, 2H, CH_2_-carborane), 2.23–2.17 (m, 2H, CH_2_-C*H*_2_-CH_2_), 1.72–1.68 (m, 4H, CH_2_-C*H*_2pyrrolidine_-CH_2_), and 3.0–1.5 (m, 10H, B_10_H_10_); ^13^C NMR (acetone-d_6_, 150.95 MHz): δ (ppm) = 164.62–160.12 (3C, C11 + C12 + C4), 143.44 (1C, C_triazole_), 133.76–107.91 (10C, 9C_arom_ + CH_triazole_), 76.19 (1C, C_carborane_), 63.55 (1C, O-CH_2_-triazole), 63.46 (1C, CH_carborane_), 54.86 (2C, N-*C*H_2pyrrolidine_-CH_2_), 54.32 (1C, CH_2_-pyrrolidine), 49.62 (1C, CH_2_-triazole), 39.57 (1C, *C*H_2_-N(CO)_2_), 35.19 (1C, CH_2_-carborane), 30.69 (1C, CH_2_-*C*H_2_-CH_2_), and 24.26 (2C, CH_2_-*C*H_2pyrrolidine_-CH_2_); ^11^B NMR {^1^H BB} (acetone-d_6_, 192.59 MHz): δ (ppm) = −2.87 (s, 1B, B9), −6.02 (s, 1B, B12), −9.65 (s, 2B, B8, 10), and −11.64 to −13.04 (m, 6B, B3, 4, 5,6, 7, 11); UV (99.8% EtOH): λ_max_ (nm) = 243 and 363, λ_min_ = 226 and 284, and λ_sh_ = 298; FT-IR: ν_max_ (cm^−1^) = 2960 (C-H_aliphat_), 2581 (B-H), 1693 (C=O), 1651 (C=O), and 722 (B-B); RP-HPLC (gradient B): t_R_ = 21.44 min; APCI-MS: *m*/*z*: 577 [M + H]^+^, calcd for C_26_H_37_B_10_N_5_O_3_: 576.

*N-[2-(Dimethylamino)ethyl]-4-{1-[3-(1,7-dicarba-closo-dodecaborane-1-yl)propyl]-1H-1,2,3-triazol-4-yl}methoxy-1,8-naphthalimide* (**18**): yellow solid, yield 20 mg (87%). TLC (MeOH/CH_2_Cl_2_, 1:9, *v*/*v*): *R*_f_ = 0.30. ^1^H NMR (acetone-d_6_, 600.26 MHz): δ (ppm) = 8.52–8.44 (m, 3H, 3H_arom_), 8.30 (s, 1H, CH_triazole_), 7.75 (dd, *J* = 8.4, 7.2 Hz, 1H, H_arom_), 7.49 (d, *J* = 8.3 Hz, 1H, H_arom_), 5.58 (s, 2H, O-CH_2_-triazole), 4.47 (t, *J* = 6.5 Hz, 2H, CH_2_-triazole), 4.22 (t, *J* = 7.0 Hz, 2H, CH_2_-N(CO)_2_), 3.65 (br s, 1H, CH_carborane_), 2.56 (t, *J* = 7.0 Hz, 2H, C*H*_2_-N(CH_3_)_2_), 2.25 (s, 6H, N(CH_3_)_2_), 2.09–2.05 (m, 4H, CH_2_-carborane and CH_2_-C*H*_2_-CH_2_ overlapped with signal from acetone-d_6_), and 3.0–1.5 (m, 10H, B_10_H_10_); ^13^C NMR (acetone-d_6_, 150.95 MHz): δ (ppm) = 164.64–160.13 (3C, C11 + C12 + C4), 143.38 (1C, C_triazole_), 133.77–107.92 (10C, 9C_arom_ + CH_triazole_), 76.53 (1C, C_carborane_), 63.57 (1C, O-CH_2_-triazole), 57.76 (1C, *C*H_2_-N(CH_3_)_2_), 56.80 (1C, CH_carborane_), 49.78 (1C, CH_2_-triazole), 46.01 (2C, 2 × CH_3_), 38.57 (1C, *C*H_2_-N(CO)_2_), 34.25 (1C, CH_2_-carborane), and 31.36 (1C, CH_2_-*C*H_2_-CH_2_); ^11^B NMR {^1^H BB} (acetone-d_6_, 192.59 MHz): δ (ppm) = −4.42 (s, 1B, B5), −9.91 to −11.06 (m, 5B, B4, 6, 9, 10, 12), −13.54 (s, 2B, B8, 11), and −15.07 (s, 2B, B2, 3); UV (99.8% EtOH): λ_max_ (nm) = 243 and 363, λ_min_ = 226 and 284, and λ_sh_ = 298; FT-IR: ν_max_ (cm^−1^) = 2944 (C-H_aliphat_), 2594 (B-H), 1694 (C=O), 1651 (C=O), and 730 (B-B); RP-HPLC (gradient B): t_R_ = 20.98 min; APCI-MS: *m*/*z*: 550 [M + H]^+^, calcd for C_24_H_35_B_10_N_5_O_3_: 549.

*N-[2-(N-Pyrrolidinyl)ethyl]-4-{1-[3-(1,7-dicarba-closo-dodecaborane-1-yl)propyl]-1H-1,2,3-triazol-4-yl}methoxy-1,8-naphthalimide* (**19**): yellow solid, yield 15 mg (82%). TLC (MeOH/CH_2_Cl_2_, 1:9, *v*/*v*): R_f_ = 0.36. ^1^H NMR (acetone-d_6_, 600.26 MHz): δ (ppm) = 8.49–8.42 (m, 3H, 3H_arom_), 8.31 (s, 1H, CH_triazole_), 7.72 (dd, *J* = 8.4, 7.2 Hz, 1H, H_arom_), 7.46 (d, *J* = 8.3 Hz, 1H, H_arom_), 5.57 (s, 2H, O-CH_2_-triazole), 4.47 (t, *J* = 6.2, 2H, CH_2_-triazole), 4.26 (t, *J* = 7.1 Hz, 2H, CH_2_-N(CO)_2_), 3.65 (br s, 1H, CH_carborane_), 2.77 (t, *J* = 7.1 Hz, 2H, CH_2_-pyrrolidine), 2.62 (br s, 4H, N-C*H*_2pyrrolidine_-CH_2_), 2.10–2.06 (m, 4H, CH_2_-carborane and CH_2_-C*H*_2_-CH_2_ overlapped with signal from acetone-d_6_), 1.74–1.69 (m, 4H, CH_2_-C*H*_2pyrrolidine_-CH_2_), and 3.0–1.5 (m, 10H, B_10_H_10_); ^13^C NMR (acetone-d_6_, 150.95 MHz): δ (ppm) = 164.62–160.10 (3C, C11 + C12 + C4), 143.38 (1C, C_triazole_), 133.74–107.88 (10C, 9C_arom_ + CH_triazole_), 76.54 (1C, C_carborane_), 63.55 (1C, O-CH_2_-triazole), 56.80 (1C, CH_carborane_), 54.82 (2C, N-*C*H_2pyrrolidine_-CH_2_), 54.26 (1C, CH_2_-pyrrolidine), 49.79 (1C, CH_2_-triazole), 39.46 (1C, *C*H_2_-N(CO)_2_), 34.25 (1C, CH_2_-carborane), 31.36 (1C, CH_2_-*C*H_2_-CH_2_), and 24.26 (2C, CH_2_-*C*H_2pyrrolidine_-CH_2_); ^11^B NMR {^1^H BB} (acetone-d_6_, 192.59 MHz): δ (ppm) = −4.41 (s, 1B, B5), −9.92 to −11.05 (m, 5B, B4, 6, 9, 10, 12), −13.54 (s, 2B, B8, 11), and −15.09 (s, 2B, B2, 3); UV (99.8% EtOH): λ_max_ (nm) = 243 and 363, λ_min_ = 227 and 284, and λ_sh_ = 298; FT-IR: ν_max_ (cm^−1^) = 2960 (C-H_aliphat_), 2595 (B-H), 1694 (C=O), 1651 (C=O), and 730 (B-B); RP-HPLC (gradient B): t_R_ = 21.40 min; APCI-MS: *m*/*z*: 577 [M + H]^+^, calcd for C_26_H_37_B_10_N_5_O_3_: 576.

#### 3.1.5. Synthesis of 1,8-Naphthalic Anhydride Derivatives **22** and **23** and 1,8-Naphthalimide Derivatives **24** and **27** Modified with Carborane Cluster via Mitsunobu Reaction

4-Hydroxy-1,8-naphthalic anhydride (**12**) (1 equiv.), 1-(3-hydroxy-propyl)-1,2-dicarba-*closo*-dodecaborane (**20**) or 1-(3-hydroxy-propyl)-1,7-dicarba-*closo*-dodecaborane (**21**) (1.1 equiv.) and triphenylphosphine (1.2 equiv.) were suspended in anhydrous THF (0.3 mL per 0.1 mmol). The suspension was cooled to 0 °C under an inert atmosphere (Ar) and DIAD (1.2 equiv.) dissolved in anhydrous THF (0.7 mL per 0.1 mmol) was added dropwise. The reaction mixture was stirred for 48–72 h at RT. Next, water (3 mL) was added to the reaction, and solvents were evaporated. The crude product was extracted with CHCl_3_ (3 × 5 mL). The organic phase was separated, dried over MgSO_4_, filtered, and evaporated to dryness. The residue was purified by column chromatography twice on silica gel (230–400 mesh) using CHCl_3_ (100%) as an eluent to afford the product.

*4-[3-(1,2-Dicarba-closo-dodecaborane-1-yl)propoxy]-1,8-naphthalic anhydride* (**22**): white solid, yield 53 mg (95%). TLC (MeOH/CH_2_Cl_2_, 1:99, *v/v*): *R*_f_ = 0.26. ^1^H NMR (DMSO-d_6_, 600.26 MHz): *δ* (ppm) = 8.60 (dd, *J* = 8.3, 1.1 Hz, 1H, H_arom_), 8.51 (dd, *J* = 7.3, 1.1 Hz, 1H, H_arom_), 8.46 (d, *J* = 8.3 Hz, 1H, H_arom_), 7.86 (dd, *J* = 8.4, 7.2 Hz, 1H, H_arom_), 7.31 (d, *J* = 8.4 Hz, 1H, H_arom_), 5.30 (br s, 1H, CH_carborane_), 4.33 (t, *J* = 6.0 Hz, 2H, CH_2_-O), 2.63–2.58 (m, 2H, CH_2_-carborane), 2.10–2.05 (m, 2H, CH_2_-C*H*_2_-CH_2_), and 3.00–1.50 (m, 10H, B_10_H_10_); ^13^C NMR (DMSO-d_6_, 150.95 MHz): *δ* (ppm) = 161.18–160.26 (3C, C11 + C12 + C4), 135.40–107.37 (9C, 9C_arom_), 76.04 (1C, C_carborane_), 67.91 (1C, CH_2_-O), 63.35 (1C, CH_carborane_), 33.42 (1C, CH_2_-carborane), and 28.48 (1C, CH_2_-*C*H_2_-CH_2_); ^11^B NMR {^1^H BB} (DMSO-d_6_, 192.59 MHz): *δ* (ppm) = −3.23 (s, 1B, B9), −6.23 (s, 1B, B12), and −9.76 to −12.89 (m, 6B, B3, 4, 5, 6, 7, 8, 10, 11); UV (99.8% EtOH): λ_max_ (nm) = 249 and 359, λ_min_ = 227 and 291, and λ_sh_ = 271; FT-IR: *ν*_max_ (cm^−1^) = 3058 (C-H_aliphat_), 2579 (B-H), 1769 (C=O), 1740 (C=O), and 722 (B-B); RP-HPLC (gradient A): *t*_R_ = 20.74 min; APCI-MS: *m/z*: 399 [M + H]^+^, 431 [M+H+MeOH]^+^, and 440 [M+H+CH_3_CN]^+^, calcd for C_17_H_22_B_10_O_4_: 398.

*4-[3-(1,7-Dicarba-closo-dodecaborane-1-yl)propoxy]-1,8-naphthalic anhydride* (**23**): white solid, yield 43 mg (78%). TLC (MeOH/CH_2_Cl_2_, 1:99, *v/v*): *R*_f_ = 0.30. ^1^H NMR (CDCl_3_, 600.26 MHz): *δ* (ppm) = 8.60 (dd, *J* = 7.3, 1.2 Hz, 1H, H_arom_), 8.56 (dd, *J* = 8.4, 1.3 Hz, 1H, H_arom_), 8.52 (d, *J* = 8.3 Hz, 1H, H_arom_), 7.76 (dd, *J* = 8.4, 7.2 Hz, 1H, H_arom_), 7.02 (d, *J* = 8.3 Hz, 1H, H_arom_), 4.23 (t, *J* = 6.0 Hz, 2H, CH_2_-O), 2.98 (br s, 1H, CH_carborane_), 2.31–2.26 (m, 2H, CH_2_-carborane), 2.12 -2.05 (m, 2H, CH_2_-C*H*_2_-CH_2_), and 3.00–1.50 (m, 10H, B_10_H_10_); ^13^C NMR (CDCl_3_, 150.95 MHz): *δ* (ppm) = 161.27–160.44 (3C, C11 + C12 + C4), 135.95–106.52 (9C, 9C_arom_), 75.23 (1C, C_carborane_), 68.24 (1C, CH_2_-O), 55.21 (1C, CH_carborane_), 33.68 (1C, CH_2_-carborane), and 29.40 (1C, CH_2_-*C*H_2_-CH_2_); ^11^B NMR {^1^H BB} (CDCl_3_, 192.59 MHz): *δ* (ppm) = −4.05 (s, 1B, B5), −9.70 to −10.93 (m, 5B, B4, 6, 9, 10, 12), −13.42 (s, 2B, B8, 11), and −15.22 (s, 2B, B2, 3); UV (99.8% EtOH): λ_max_ (nm) = 249 and 360, λ_min_ = 226 and 291, and λ_sh_ = 271; FT-IR: *ν*_max_ (cm^−1^) = 3066 (C-H_aliphat_), 2594 (B-H), 1772 (C=O), and 1732 (C=O), 724 (B-B); RP-HPLC (gradient A): *t*_R_ = 21.55 min; APCI-MS: *m/z*: 399 [M + H]^+^ and 431 [M+H+MeOH]^+^, calcd for C_17_H_22_B_10_O_4_: 398.

4-[3-(1,2-Dicarba-*closo*-dodecaborane-1-yl)propoxy]-1,8-naphthalic anhydride (**22**) or 4-{14-[3-(1,7-dicarba-*closo*-dodecaborane-1-yl)propoxy]-1,8-naphthalic anhydride (**23**) was suspended in absolute EtOH (7–8 mL per 0.1 mmol) and *N*,*N*-dimethylethylenediamine (1.7 equiv.) or *N*-(2-aminoethyl)pyrrolidine (1.7 equiv.) was added. The reaction mixture was stirred for 4 h at 45 °C under an inert (Ar) atmosphere. Subsequently, the solvent was evaporated to dryness under vacuum, and the crude product was purified by column chromatography on silica gel (230–400 mesh) with a gradient of MeOH (0–10%) in CHCl_3_.

*N-[2-(Dimethylamino)ethyl]-4-[3-(1,2-dicarba-closo-dodecaborane-1-yl)propoxy]-1,8-naphthalimide* (**24**): white solid, yield 12.2 mg (65%). TLC (MeOH/CH_2_Cl_2_, 1:9, *v/v*): *R*_f_ = 0.33. ^1^H NMR (acetone-d_6_, 600.26 MHz): *δ* (ppm) = 8.54 (dd, *J* = 8.4, 1.3 Hz, 1H, H_arom_), 8.50 (dd, *J* = 7.3, 1.3 Hz, 1H, H_arom_), 8.43 (d, *J* = 8.2 Hz, 1H, H_arom_), 7.78 (dd, *J* = 8.4, 7.2 Hz, 1H, H_arom_), 7.23 (d, *J* = 8.3 Hz, 1H, H_arom_), 4.81 (br s, 1H, CH_carborane_), 4.41 (t, *J* = 6.0 Hz, 2H, CH_2_-O), 4.22 (t, *J* = 7.0 Hz, 2H, CH_2_-N(CO)_2_), 2.80–2.75 (m, 2H, CH_2_-carborane), 2.56 (t, *J* = 7.0 Hz, 2H, C*H*_2_-N(CH_3_)_2_), 2.29–2.24 (m, 8H, N(CH_3_)_2_ overlapped with CH_2_-C*H*_2_-CH_2_), and 3.0–1.5 (m, 10H, B_10_H_10_); ^13^C NMR (acetone-d_6_, 150.95 MHz): *δ* (ppm) = 164.64–160.50 (3C, C11 + C12 + C4), 133.88–107.24 (9C, 9C_arom_), 76.73 (1C, C_carborane_), 68.54 (1C, CH_2_-O), 63.73, (1C, CH_carborane_), 57.76 (1C, *C*H_2_-N(CH_3_)_2_), 46.01 (2C, 2 × CH_3_), 38.56 (1C, *C*H_2_-N(CO)_2_), 35.22 (1C, CH_2_-carborane), and 30.35 (1C, CH_2_-*C*H_2_-CH_2_); ^11^B NMR {^1^H BB} (acetone-d_6_, 192.59 MHz): *δ* (ppm) = −2.82 (s, 1B, B9), −6.04 (s, 1B, B12), −9.57 (s, 2B, B8, 10), and −11.49 to −12.98 (m, 6B, B3, 4, 5, 6, 7, 11); UV (99.8% EtOH): λ_max_ (nm) = 243 and 364, and λ_min_ = 224 and 283; FT-IR: *ν*_max_ (cm^−1^) = 2940 (C-H_aliphat_), 2579 (B-H), 1694 (C=O), and 1647 (C=O), 721 (B-B); RP-HPLC (gradient B): *t*_R_ = 22.78 min; APCI-MS: *m/z*: 470 [M + H]^+^, calcd for C_21_H_32_B_10_N_2_O_3_: 469.

*N-[2-(N-Pyrrolidinyl)ethyl]-4-[3-(1,2-dicarba-closo-dodecaborane-1-yl)propoxy]-1,8-naphthalimide* (**25**): white solid, 11.4 mg (57%). TLC (MeOH/CH_2_Cl_2_, 1:9, *v/v*): *R*_f_ = 0.37. ^1^H NMR (acetone-d_6_, 600.26 MHz): *δ* (ppm) = 8.54 (dd, *J* = 8.3, 1.3 Hz, 1H, H_arom_), 8.50 (dd, *J* = 7.2, 1.3 Hz, 1H, H_arom_), 8.43 (d, *J* = 8.2 Hz, 1H, H_arom_), 7.77 (dd, *J* = 8.4, 7.2 Hz, 1H, H_arom_), 7.23 (d, *J* = 8.3 Hz, 1H, H_arom_), 4.81 (br s, 1H, CH_carborane_), 4.41 (t, *J* = 6.0 Hz, 2H, CH_2_-O), 4.25 (t, *J* = 7.1 Hz, 2H, CH_2_-N(CO)_2_), 2.80–2.73 (m, 4H, CH_2_-pyrrolidine overlapped with CH_2_-C*H*_2_-CH_2_), 2.64–2.57 (m, 4H, N-C*H*_2pyrrolidine_-CH_2_), 2.30–2.22 (m, 2H, CH_2_-carborane), 1.74–1.68 (m, 4H, CH_2_-C*H*_2pyrrolidine_-CH_2_), and 3.0–1.5 (m, 10H, B_10_H_10_); ^13^C NMR (acetone-d_6_, 150.95 MHz): *δ* (ppm) = 163.75–159.63 (3C, C11 + C12 + C4), 133.00–106.36 (9C, 9C_arom_), 75.87 (1C, C_carborane_), 67.67 (1C, CH_2_-O), 62.86 (1C, CH_carborane_), 53.96 (2C, N-*C*H_2pyrrolidine_-CH_2_), 53.39 (1C, CH_2_-pyrrolidine), 38.62 (1C, *C*H_2_-N(CO)_2_), 34.34 (1C, CH_2_-carborane), 29.47 (1C, CH_2_-*C*H_2_-CH_2_), and 23.38 (2C, CH_2_-*C*H_2pyrrolidine_-CH_2_); ^11^B NMR {^1^H BB} (acetone-d_6_, 192.59 MHz): *δ* (ppm) = −2.81 (s, 1B, B9), −6.01 (s, 1B, B12), −9.57 (s, 2B, B8, 10), and −11.50 to −12.97 (m, 6B, B3, 4, 5, 6, 7, 11); UV (99.8% EtOH): λ_max_ (nm) = 243 and 364, and λ_min_ = 224 and 284; FT-IR: *ν*_max_ (cm^−1^) = 2953 (C-H_aliphat_), 2577 (B-H), 1693 (C=O), and 1647 (C=O), 721 (B-B); RP-HPLC (gradient B): *t*_R_ = 23.17 min; APCI-MS: *m/z*: 495 [M + H]^+^, calcd for C_23_H_34_B_10_N_2_O_3_: 494.

*N-[2-(Dimethylamino)ethyl]-4-[3-(1,7-dicarba-closo-dodecaborane-1-yl)propoxy]-1,8-naphthalimide* (**26**): white solid, yield 14 mg (51%). TLC (MeOH/CH_2_Cl_2_, 1:9, *v/v*): *R*_f_ = 0.35. ^1^H NMR (acetone-d_6_, 600.26 MHz): *δ* (ppm) = 8.56 (dd, *J* = 8.3, 1.3 Hz, 1H, H_arom_), 8.53 (dd, *J* = 7.2, 1.2 Hz, 1H, H_arom_), 8.45 (d, *J* = 8.2 Hz, 1H, H_arom_), 7.81 (dd, *J* = 8.3, 7.3 Hz, 1H, H_arom_), 7.24 (d, *J* = 8.3 Hz, 1H, H_arom_), 4.36 (t, *J* = 6.1 Hz, 2H, CH_2_-O), 4.22 (t, *J* = 7.0 Hz, 2H, CH_2_-N(CO)_2_), 3.72 (br s, 1H, CH_carborane_), 2.56 (t, *J* = 7.0 Hz, 2H, C*H*_2_-N(CH_3_)_2_), 2.43–2.37 (m, 2H, CH_2_-carborane), 2.26 (s, 6H, N(CH_3_)_2_), 2.13–2.07 (m, 2H, CH_2_-C*H*_2_-CH_2_), and 3.0–1.5 (m, 10H, B_10_H_10_); ^13^C NMR (acetone-d_6_, 150.95 MHz): *δ* (ppm) = 164.67–160.60 (3C, C11 + C12 + C4), 133.92–107.25 (9C, 9C_arom_), 77.11 (1C, C_carborane_), 68.76 (1C, CH_2_-O), 57.75 (1C, *C*H_2_-N(CH_3_)_2_), 56.84, (1C, CH_carborane_), 45.99 (2C, 2 × CH_3_), 38.54 (1C, *C*H_2_-N(CO)_2_), 34.20 (1C, CH_2_-carborane), and 30.29 (1C, CH_2_-*C*H_2_-CH_2_); ^11^B NMR {^1^H BB} (acetone-d_6_, 192.59 MHz): *δ* (ppm) = −4.26 (s, 1B, B5), −9.93 to −10.94 (m, 5B, B4, 6, 9, 10, 12), −13.48 (s, 2B, B8, 11), and −14.96 (s, 2B, B2, 3); UV (99.8% EtOH): λ_max_ (nm) = 243 and 365, and λ_min_ = 224 and 284; FT-IR: *ν*_max_ (cm^−1^) = 2939 (C-H_aliphat_), 2590 (B-H), 1691 (C=O), and 1652 (C=O), 731 (B-B); RP-HPLC (gradient B): *t*_R_ = 22.83 min; APCI-MS: *m/z*: 470 [M + H]^+^, calcd for C_21_H_32_B_10_N_2_O_3_: 469.

*N-[2-(N-Pyrrolidinyl)ethyl]-4-[3-(1,7-dicarba-closo-dodecaborane-1-yl)propoxy]-1,8-naphthalimide* (**27**): white solid, yield 13.5 mg (54%). TLC (MeOH/CH_2_Cl_2_, 1:9, *v/v*): *R*_f_ = 0.40. ^1^H NMR (DMSO-d_6_, 600.26 MHz): *δ* (ppm) = 8.47–8.46 (m, 2H, 2H_arom_), 8.40 (d, *J* = 8.3 Hz, 1H, H_arom_), 7.81 (t, *J* = 7.9 Hz, 1H, H_arom_), 7.23 (d, *J* = 8.4 Hz, 1H, H_arom_), 4.26 (t, *J* = 6.1 Hz, 2H, CH_2_-O), 4.14 (t, *J* = 7.2 Hz, 2H, CH_2_-N(CO)_2_), 4.10 (br s, 1H, CH_carborane_), 2.67 (t, *J* = 7.5 Hz, 2H, CH_2_-pyrrolidine), 2.54 (br s, 4H, N-C*H*_2pyrrolidine_-CH_2_), 2.31–2.25 (m, 2H, CH_2_-carborane), 1.99–1.90 (m, 2H, CH_2_-C*H*_2_-CH_2_), 1.67–1.65 (m, 4H, CH_2_-C*H*_2pyrrolidine_-CH_2_), and 3.0–1.5 (m, 10H, B_10_H_10_); ^13^C NMR (DMSO-d_6_, 150.95 MHz): *δ* (ppm) = 163.56–159.37 (3C, C11 + C12 + C4), 133.32–106.88 (9C, 9C_arom_), 76.24 (1C, C_carborane_), 67.72 (1C, CH_2_-O), 56.43 (1C, CH_carborane_), 53.99 (2C, N-*C*H_2pyrrolidine_-CH_2_), 53.12 (1C, CH_2_-pyrrolidine), 38.47 (1C, *C*H_2_-N(CO)_2_), 32.66 (1C, CH_2_-carborane), 29.08 (1C, CH_2_-*C*H_2_-CH_2_), and 23.20 (2C, CH_2_-*C*H_2pyrrolidine_-CH_2_); ^11^B NMR {^1^H BB} (DMSO-d_6_, 192.59 MHz): *δ* (ppm) = −4.62 (s, 1B, B5), −11.11 (s, 5B, B4, 6, 9, 10, 12), −13.55 (s, 2B, B8, 11), and −14.94 (s, 2B, B2, 3); UV (99.8% EtOH): λ_max_ (nm) = 243 and 365, and λ_min_ = 224 and 284; FT-IR: *ν*_max_ (cm^−1^) = 2949 (C-H_aliphat_), 2589 (B-H), 1693 (C=O), 1652 (C=O), and 734 (B-B); RP-HPLC (gradient B): *t*_R_ = 23.19 min; APCI-MS: *m/z*: 495 [M + H]^+^, calcd for C_23_H_34_B_10_N_2_O_3_: 494.

#### 3.1.6. Synthesis of 1,8-Naphthalimide Derivatives **36** and **43** Modified with Carborane Cluster via Amidation Reaction

*N*-[2-(Dimethylamino)ethyl]-4-[(2-aminoethyl)amino]-1,8-naphthalimide (**30**), *N*-[2-(*N*-pyrrolidinyl)ethyl]-4-[(2-aminoethyl)amino]-1,8-naphthalimide (**31**), *N*-[2-(dimethylamino)ethyl]-4-[(6-aminohexyl)amino]-1,8-naphthalimide (**32**) or *N*-[2-(*N*-pyrrolidinyl)ethyl]-4-[(6-aminohexyl)amino]-1,8-naphthalimide (**33**) (1 equiv.) and 3-(1,2-dicarba-*closo*-dodecaboran-1-yl)propionic acid *N*-succinimidyl ester (**34**) or 3-(1,7-dicarba-*closo*-dodecaboran-1-yl)propionic acid *N*-succinimidyl ester (**35**) (1 equiv.) were dissolved in anhydrous CH_2_Cl_2_ (1 mL per 0.1 mmol). Reaction mixture was stirred under dry argon for 1.5–5 h at 40 °C. Subsequently, the solvent was evaporated to dryness and crude product was purified by column chromatography on silica gel (230–400 mesh) using CH_3_CN as an eluent, then a gradient of MeOH (3–20%) in CH_2_Cl_2_. Compounds **36**, **38**, **40** and **42** were purified additionally by column chromatography on silica gel (230–400 mesh) with a gradient of MeOH (3–20%) in CH_2_Cl_2_.

*N-[2-(Dimethylamino)ethyl]-4-[(2-(3-(1,2-dicarba-closo-dodecaborane-1-yl)propanamido)ethyl)amino]-1,8-naphthalimide* (**36**): yellow solid, yield 19.6 mg (71%). TLC (MeOH/CH_2_Cl_2_, 3:17, *v/v*): *R*_f_ = 0.29. ^1^H NMR (DMSO-d_6_, 600.26 MHz): *δ* (ppm) = 8.58 (d, *J* = 7.9 Hz, 1H, H_arom_), 8.43–8.42 (m, 1H, H_arom_), 8.25–8.24 (m, 2H, H_arom_ + NH), 7.79 (t, *J* = 5.6 Hz, 1H, NH), 7.70–7.67 (m, 1H, H_arom_), 6.82 (d, *J* = 8.7 Hz, 1H, H_arom_), 5.15 (br s, 1H, CH_carborane_), 4.11 (t, *J* = 7.1 Hz, 2H, CH_2_-N(CO)_2_), 3.45 (q, *J* = 6.0 Hz, 2H, C*H*_2_-NH), 3.38 (q, *J* = 6.1 Hz, 2H, C*H*_2_-NH), 2.49–2.46 (m, 4H, CH_2_-carborane overlapped with C*H*_2_-N(CH_3_)_2_), 2.30 (dd, *J* = 9.5, 6.7 Hz, 2H, NH-C(O)-C*H*_2_), 2.20 (s, 6H, N(CH_3_)_2_), and 3.0–1.5 (m, 10H, B_10_H_10_); ^13^C NMR (DMSO-d_6_, 150.95 MHz): *δ* (ppm) = 170.28 (1C, C(O)-NH), 163.74–162.86 (2C, C11 + C12), 150.61–103.72 (10C, 10C_arom_), 75.78 (1C, C_carborane_), 63.35 (1C, CH_carborane_), 56.67 (1C, *C*H_2_-N(CH_3_)_2_), 45.41 (2C, 2 × CH_3_), 42.47 (1C, CH_2_-NH), 37.57 (1C, CH_2_-NH), 37.20 (1C, *C*H_2_-N(CO)_2_), 34.57 (1C, CH_2_-carborane), and 32.31 (1C, NH-C(O)-*C*H_2_); ^11^B NMR {^1^H BB} (DMSO-d_6_, 192.59 MHz): *δ* (ppm) = −3.18 (s, 1B, B9), −6.18 (s, 1B, B12), and −9.84 to −13.08 (m, 8B, B3, 4, 5, 6, 7, 8, 10, 11); UV (99.8% EtOH): λ_max_ (nm) = 261, 281, 322, and 438, and λ_min_ = 241, 267, 316, and 349; FT-IR: *ν*_max_ (cm^−1^) = 2927 (C-H_aliphat_), 2587 (B-H), 1671 (C=O), and 1632 (C=O), 723 (B-B); RP-HPLC (gradient B): *t*_R_ = 19.89 min; APCI-MS: *m/z*: 526 [M + H]^+^, calcd for C_23_H_36_B_10_N_4_O_3_: 525.

*N-[2-(Dimethylamino)ethyl]-4-[(2-(3-(1,7-dicarba-closo-dodecaborane-1-yl)propanamido)ethyl)amino]-1,8-naphthalimide* (**37**): yellow solid, yield 25.1 mg, 91%. TLC (MeOH/CH_2_Cl_2_, 3:17, *v/v*): *R*_f_ = 0.29. ^1^H NMR (MeOH-d_4_, 600.26 MHz): *δ* (ppm) = 8.40 (d, *J* = 6.8 Hz, 1H, H_arom_), 8.32 (d, *J* = 8.3 Hz, 1H, H_arom_), 8.23 (d, *J* = 8.5 Hz, 1H, H_arom_), 7.57 (dd, *J* = 8.5, 7.3 Hz, 1H, H_arom_), 6.73 (d, *J* = 8.7 Hz, 1H, H_arom_), 4.25 (t, *J* = 7.2 Hz, 2H, CH_2_-N(CO)_2_), 3.54 (br s, 4H, 2C*H*_2_-NH), 3.44 (br s, 1H, CH_carborane_), 2.69 (t, *J* = 7.2 Hz, 2H, C*H*_2_-N(CH_3_)_2_), 2.40 (s, 6H, N(CH_3_)_2_), 2.27–2.24 (m, 2H, CH_2_-carborane), 2.16–2.14 (m, 2H, NH-C(O)-C*H*_2_), and 3.0–1.5 (m, 10H, B_10_H_10_); ^13^C NMR (MeOH-d_4_, 150.95 MHz): *δ* (ppm) = 174.42 (1C, C(O)-NH), 166.11–165.50 (2C, C11 + C12), 152.46–104.91 (10C, 10C_arom_), 76.60 (1C, C_carborane_), 57.83 (1C, *C*H_2_-N(CH_3_)_2_), 57.00 (1C, CH_carborane_), 45.65 (2C, 2 × CH_3_), 44.39 (1C, CH_2_-NH), 39.54 (1C, CH_2_-NH), 38.30 (1C, *C*H_2_-N(CO)_2_), 36.75 (1C, CH_2_-carborane), and 33.30 (1C, NH-C(O)-*C*H_2_); ^11^B NMR {^1^H BB} (MeOH-d_4_, 192.59 MHz): *δ* (ppm) = −4.49 (s, 1B, B5), −9.90 to −11.03 (m, 5B, B4, 6, 9, 10, 12), −13.59 (s, 2B, B8, 11), and −15.25 (s, 2B, B2, 3); UV (99.8% EtOH): λ_max_ (nm) = 261, 281, 323, and 438, and λ_min_ = 241, 267, 317, and 349; FT-IR: *ν*_max_ (cm^−1^) = 2945 (C-H_aliphat_), 2596 (B-H), 1680 (C=O), 1632 (C=O), and 730 (B-B); RP-HPLC (gradient B): *t*_R_ = 19.73 min; APCI-MS: *m/z*: 526 [M + H]^+^, calcd for C_23_H_36_B_10_N_4_O_3_: 525.

*N-[2-(N-Pyrrolidinyl)ethyl]-4-[(2-(3-(1,2-dicarba-closo-dodecaborane-1-yl)propanamido)ethyl)amino]-1,8-naphthalimide* (**38**): yellow solid, yield 11.4 mg (40%). TLC (MeOH/CH_2_Cl_2_, 3:17, *v/v*): *R*_f_ = 0.26. ^1^H NMR (MeOH-d_4_, 600.26 MHz): *δ* (ppm) = 8.39 (dd, *J* = 7.3, 1.3 Hz, 1H, H_arom_), 8.32 (dd, *J* = 8.4, 1.2 Hz, 1H, H_arom_), 8.22 (d, *J* = 8.5 Hz, 1H, H_arom_), 7.56 (dd, *J* = 8.5, 7.3 Hz, 1H, H_arom_), 6.72 (d, *J* = 8.5 Hz, 1H, H_arom_), 4.49 (s, 1H, CH_carborane_), 4.28 (t, *J* = 7.2 Hz, 2H, CH_2_-N(CO)_2_), 3.58–3.55 (m, 2H, C*H*_2_-NH), 3.55–3.50 (m, 2H, C*H*_2_-NH), 2.87 (t, *J* = 7.2 Hz, 2H, CH_2_-pyrrolidine), 2.79–2.78 (m, 4H, N-C*H*_2pyrrolidine_-CH_2_), 2.51–2.49 (m, 2H, CH_2_-carborane), 2.42–2.41 (m, 2H, NH-C(O)-C*H*_2_), 1.86 (br s, 4H, CH_2_-C*H*_2pyrrolidine_-CH_2_), and 3.0–1.5 (m, 10H, B_10_H_10_); ^13^C NMR (MeOH-d_4_, 150.95 MHz): *δ* (ppm) = 173.96 (1C, C(O)-NH), 166.10–165.48 (2C, C11 + C12), 152.40–104.89 (10C, 10C_arom_), 76.26 (1C, C_carborane_), 63.91 (1C, CH_carborane_), 55.35 (2C, N-*C*H_2pyrrolidine_-CH_2_), 54.83 (1C, CH_2_-pyrrolidine), 44.49 (1C, CH_2_-NH), 39.56 (1C, CH_2_-NH), 39.17 (1C, *C*H_2_-N(CO)_2_), 35.91 (1C, CH_2_-carborane), 34.03 (1C, NH-C(O)-*C*H_2_), and 24.21 (2C, CH_2_-*C*H_2pyrrolidine_-CH_2_);^11^B NMR {^1^H BB} (MeOH-d_4_, 192.59 MHz): *δ* (ppm) = −2.74 (s, 1B, B9), −5.97 (s, 1B, B12), −9.68 (s, 2B, B8, 10), and −11.86 to −13.06 (m, 6B, B3, 4, 5, 6, 7, 11); UV (99.8% EtOH): λ_max_ (nm) = 261, 280, 325, 438, and λ_min_ = 242, 269, 316, and 348; FT-IR: *ν*_max_ (cm^−1^) = 2923 (C-H_aliphat_), 2578 (B-H), 1680 (C=O), 1636 (C=O), and 722 (B-B); RP-HPLC (gradient B): *t*_R_ = 19.79 min; APCI-MS: *m/z*: 551 [M + H]^+^, calcd for C_25_H_38_B_10_N_4_O_3_: 550.

*N-[2-(N-Pyrrolidinyl)ethyl]-4-[(2-(3-(1,7-dicarba-closo-dodecaborane-1-yl)propanamido)ethyl)amino]-1,8-naphthalimide* (**39**): yellow solid, yield 17 mg (59%). TLC (MeOH/CH_2_Cl_2_, 3:17, *v/v*): *R*_f_ = 0.30. ^1^H NMR (MeOH-d_4_, 600.26 MHz): *δ* (ppm) = 8.41 (dd, *J* = 7.3, 1.3 Hz, 1H, H_arom_), 8.33 (dd, *J* = 8.4, 1.2 Hz, 1H, H_arom_), 8.23 (d, *J* = 8.5 Hz, 1H, H_arom_), 7.57 (dd, *J* = 8.5, 7.2 Hz, 1H, H_arom_), 6.74 (d, *J* = 8.7 Hz, 1H, H_arom_), 4.29 (dd, *J* = 8.3, 6.0 Hz, 2H, CH_2_-N(CO)_2_), 3.55–3.54 (m, 4H, 2C*H*_2_-NH), 3.45 (br s, 1H, CH_carborane_), 2.90 (t, *J* = 7.2 Hz, 2H, CH_2_-pyrrolidine), 2.83–2.80 (m, 4H, N-C*H*_2pyrrolidine_-CH_2_), 2.27–2.24 (m, 2H, CH_2_-carborane), 2.17–2.14 (m, 2H, NH-C(O)-C*H*_2_), 1.86 (t, *J* = 3.7 Hz, 4H, CH_2_-C*H*_2pyrrolidine_-CH_2_), and 3.0–1.5 (m, 10H, B_10_H_10_); ^13^C NMR (MeOH-d_4_, 150.95 MHz): *δ* (ppm) = 174.43 (1C, C(O)-NH), 166.13–165.50 (2C, C11 + C12), 152.49–104.92 (10C, 10C_arom_), 76.60 (1C, C_carborane_), 57.01 (1C, CH_carborane_), 55.32 (2C, N-*C*H_2pyrrolidine_-CH_2_), 54.82 (1C, CH_2_-pyrrolidine), 44.40 (1C, CH_2_-NH), 39.54 (1C, CH_2_-NH), 39.21 (1C, *C*H_2_-N(CO)_2_), 36.75 (1C, CH_2_-carborane), 33.30 (1C, NH-C(O)-*C*H_2_), and 24.22 (2C, CH_2_-*C*H_2pyrrolidine_-CH_2_); ^11^B NMR {^1^H BB} (MeOH-d_4_, 192.59 MHz): *δ* (ppm) = −4.50 (s, 1B, B5), −9.95 to −11.03 (m, 5B, B4, 6, 9, 10, 12), −13.59 (s, 2B, B8, 11), and −15.25 (s, 2B, B2, 3); UV (99.8% EtOH): λ_max_ (nm) = 261, 281, 325, and 438, and λ_min_ = 241, 267, 314, and 348; FT-IR: *ν*_max_ (cm^−1^) = 2923 (C-H_aliphat_), 2593 (B-H), 1681 (C=O), 1637 (C=O), and 728 (B-B); RP-HPLC (gradient B): *t*_R_ = 20.09 min; APCI-MS: *m/z*: 551 [M + H]^+^, calcd for C_25_H_38_B_10_N_4_O_3:_550.

*N-[2-(Dimethylamino)ethyl]-4-[(6-(3-(1,2-dicarba-closo-dodecaborane-1-yl)propanamido)hexyl)amino]-1,8-naphthalimide* (**40**): yellow solid, yield 12.2 mg, (40%). TLC (MeOH/CH_2_Cl_2_, 3:17, *v/v*): *R*_f_ = 0.28. ^1^H NMR (CDCl_3_, 600.26 MHz): *δ* (ppm) = 8.51 (dd, *J* = 7.3, 1.1 Hz, 1H, H_arom_), 8.37 (d, *J* = 8.5 Hz, 1H, H_arom_), 8.08 (dd, *J* = 8.6, 1.2 Hz, 1H, H_arom_), 7.56 (dd, *J* = 8.5, 7.2 Hz, 1H, H_arom_), 6.63 (d, *J* = 8.7 Hz, 1H, H_arom_), 5.77 (t, *J* = 5.9 Hz, 1H, NH), 5.48 (t, *J* = 5.3 Hz, 1H, NH), 4.31 (t, *J* = 7.2 Hz, 2H, CH_2_-N(CO)_2_), 3.84 (br s, 1H, CH_carborane_), 3.37 (td, *J* = 7.2, 5.1 Hz, 2H, C*H*_2_-NH), 3.28–3.25 (m, 2H, C*H*_2_-NH), 2.67 (dd, *J* = 7.8, 6.5 Hz, 2H, C*H*_2_-N(CH_3_)_2_), 2.60 (t, *J* = 7.2 Hz, 2H, CH_2_-carborane), 2.39–2.36 (m, 8H, N(CH_3_)_2_ overlapped with NH-C(O)-C*H*_2_), 1.81–1.86 (m, 2H, CH_2_-C*H*_2_-CH_2_), 1.56–1.50 (m, 4H, 2(CH_2_-C*H*_2_-CH_2_)), 1.44–1.41 (m, 2H, CH_2_-C*H*_2_-CH_2_), and 3.0–1.5 (m, 10H, B_10_H_10_); ^13^C NMR (CDCl_3_, 150.95 MHz): *δ* (ppm) = 170.18 (1C, C(O)-NH), 164.83–164.27 (2C, C11 + C12), 149.65–104.36 (10C, 10C_arom_), 74.71 (1C, C_carborane_), 61.91 (1C, CH_carborane_), 57.25 (1C, *C*H_2_-N(CH_3_)_2_), 45.89 (2C, 2 × CH_3_), 43.48 (1C, CH_2_-NH), 39.55 (1C, CH_2_-NH), 37.94 (1C, *C*H_2_-N(CO)_2_), 35.21 (1C, CH_2_-carborane), 32.80 (1C, NH-C(O)-*C*H_2_), 29.62 (1C, CH_2_-*C*H_2_-CH_2_), 28.81 (1C, CH_2_-*C*H_2_-CH_2_), 26.62 (1C, CH_2_-*C*H_2_-CH_2_), and 26.46 (1C, CH_2_-*C*H_2_-CH_2_); ^11^B NMR {^1^H BB} (CDCl_3_, 192.59 MHz): *δ* (ppm) = −2.17 (s, 1B, B9), −5.64 (s, 1B, B12), and −9.62 to −12.91 (m, 8B, B3, 4, 5, 6, 7, 8, 10, 11); UV (99.8% EtOH): λ_max_ (nm) = 261, 282, 325, and 442, and λ_min_ = 242, 267, 317, and 350; FT-IR: *ν*_max_ (cm^−1^) = 2923 (C-H_aliphat_), 2580 (B-H), 1679 (C=O), 1636 (C=O), and 722 (B-B); RP-HPLC (gradient B): *t*_R_ = 21.02 min; APCI-MS: *m/z*: 581 [M + H]^+^, calcd for C_27_H_44_B_10_N_4_O_3_: 580.

*N-[2-(Dimethylamino)ethyl]-4-[(6-(3-(1,7-dicarba-closo-dodecaborane-1-yl)propanamido)hexyl)amino]-1,8-naphthalimide* (**41**): yellow solid, yield 23.6 mg (78%). TLC (MeOH/CH_2_Cl_2_, 3:17, *v/v*): *R*_f_ = 0.30. ^1^H NMR (CDCl_3_, 600.26 MHz): *δ* (ppm) = 8.50 (dd, *J* = 7.3, 1.1 Hz, 1H, H_arom_), 8.36 (d, *J* = 8.5 Hz, 1H, H_arom_), 8.13 (dd, *J* = 8.6, 1.2 Hz, 1H, H_arom_), 7.54 (dd, *J* = 8.5, 7.3 Hz, 1H, H_arom_), 6.61 (d, *J* = 8.5 Hz, 1H, H_arom_), 5.75 (t, *J* = 5.9 Hz, 1H, NH), 5.62 (t, *J* = 5.3 Hz, 1H, NH), 4.30 (t, *J* = 7.2 Hz, 2H, CH_2_-N(CO)_2_), 3.35 (td, *J* = 7.2, 5.1 Hz, 2H, C*H*_2_-NH), 3.26–3.22 (m, 2H, C*H*_2_-NH), 2.90 (br s, 1H, CH_carborane_), 2.66 (t, *J* = 7.2 Hz, 2H, C*H*_2_-NH), 2.37 (s, 6H, N(CH_3_)_2_), 2.35–2.31 (m, 2H, CH_2_-C*H*_2_-CH_2_), 2.22–2.18 (m, 2H, NH-C(O)-C*H*_2_), 1.79–1.74 (m, 2H, CH_2_-C*H*_2_-CH_2_), 1.55–1.46 (m, 4H, 2(CH_2_-C*H*_2_-CH_2_)), 1.41–1.37 (m, 2H, CH_2_-C*H*_2_-CH_2_), and 3.0–1.5 (m, 10H, B_10_H_10_); ^13^C NMR (CDCl_3_, 150.95 MHz): *δ* (ppm) = 170.58 (1C, C(O)-NH), 164.85–164.25 (2C, C11 + C12), 149.78–104.28 (10C, 10C_arom_), 75.10 (1C, C_carborane_), 57.23 (1C, *C*H_2_-N(CH_3_)_2_), 55.15 (1C, CH_carborane_), 45.86 (2C, 2 × CH_3_), 43.39 (1C, CH_2_-NH), 39.28 (1C, CH_2_-NH), 37.88 (1C, *C*H_2_-N(CO)_2_), 36.28 (1C, CH_2_-carborane), 32.07 (1C, NH-C(O)-*C*H_2_), 29.64 (1C, CH_2_-*C*H_2_-CH_2_), 28.71 (1C, CH_2_-*C*H_2_-CH_2_), 26.48 (1C, CH_2_-*C*H_2_-CH_2_), and 26.32 (1C, CH_2_-*C*H_2_-CH_2_); ^11^B NMR {^1^H BB} (CDCl_3_, 192.59 MHz): *δ* (ppm) = −4.44 (s, 1B, B5), −9.79 to −11.05 (m, 5B, B4, 6, 9, 10, 12), −13.53 (s, 2B, B8, 11), and −15.23 (s, 2B, B2, 3); UV (99.8% EtOH): λ_max_ (nm) = 261, 282, 325, and 442, and λ_min_ = 241, 267, 316, and 350; FT-IR: *ν*_max_ (cm^−1^) = 2931 (C-H_aliphat_), 2595 (B-H), 1681 (C=O), 1636 (C=O), and 729 (B-B); RP-HPLC (gradient B): *t*_R_ = 20.88 min; APCI-MS: *m/z*: 581 [M + H]^+^, calcd for C_27_H_44_B_10_N_4_O_3_: 580.

*N-[2-(N-Pyrrolidinyl)ethyl]-4-[(6-(3-(1,2-dicarba-closo-dodecaborane-1-yl)propanamido)hexyl)amino]-1,8-naphthalimide* (**42**): yellow solid, yield 16.1 mg (51%). TLC (MeOH/CH_2_Cl_2_, 3:17, *v/v*): *R*_f_ = 0.31. ^1^H NMR (CDCl_3_, 600.26 MHz): *δ* (ppm) = 8.48 (d, *J* = 7.2 Hz, 1H, H_arom_), 8.33 (d, *J* = 8.3 Hz, 1H, H_arom_), 8.10 (d, *J* = 8.3 Hz, 1H, H_arom_), 7.53 (t, *J* = 7.8 Hz, 1H, H_arom_), 6.59 (d, *J* = 8.5 Hz, 1H, H_arom_), 5.95 (t, *J* = 6.0 Hz, 1H, NH), 5.58 (t, *J* = 5.4 Hz, 1H, NH), 4.36 (t, *J* = 7.3 Hz, 2H, CH_2_-N(CO)_2_), 3.86 (br s, 1H, CH_carborane_), 3.34 (q, *J* = 7.2 Hz, 2H, C*H*_2_-NH), 3.25 (q, *J* = 6.8 Hz, 2H, C*H*_2_-NH), 2.91 (t, *J* = 7.3 Hz, 2H, CH_2_-pyrrolidine), 2.81 (t, *J* = 6.4 Hz, 4H, N-C*H*_2pyrrolidine_-CH_2_), 2.60 (t, *J* = 7.2 Hz, 2H, CH_2_-carborane), 2.39 (t, *J* = 7.2 Hz, 2H, NH-C(O)-C*H*_2_), 1.85 (*p*, *J* = 3.4 Hz, 4H, CH_2_-C*H*_2pyrrolidine_-CH_2_), 1.76 (q, *J* = 7.2 Hz, 2H CH_2_-C*H*_2_-CH_2_), 1.56–1.48 (m, 4H, 2(CH_2_-C*H*_2_-CH_2_)), 1.45–1.36 (m, 2H, CH_2_-C*H*_2_-CH_2_), and 3.0–1.5 (m, 10H, B_10_H_10_); ^13^C NMR (CDCl_3_, 150.95 MHz): *δ* (ppm) = 170.26 (1C, C(O)-NH), 164.79–164.17 (2C, C11 + C12), 149.80–104.32 (10C, 10C_arom_), 74.78 (1C, C_carborane_), 61.90 (1C, CH_carborane_), 54.40 (2C, N-*C*H_2pyrrolidine_-CH_2_), 53.77 (1C, CH_2_-pyrrolidine), 43.47 (1C, CH_2_-NH), 39.53 (1C, CH_2_-NH), 38.50 (1C, *C*H_2_-N(CO)_2_), 35.23 (1C, CH_2_-carborane), 32.87 (1C, NH-C(O)-*C*H_2_), 29.56 (1C, CH_2_-*C*H_2_-CH_2_), 28.74 (1C, CH_2_-*C*H_2_-CH_2_), 26.60 (1C, CH_2_-*C*H_2_-CH_2_), 26.45 (1C, CH_2_-*C*H_2_-CH_2_), and 23.70 (2C, CH_2_-*C*H_2pyrrolidine_-CH_2_); ^11^B NMR {^1^H BB} (CDCl_3_, 192.59 MHz): *δ* (ppm) = −2.18 (s, 1B, B9), −5.64 (s, 1B, B12), and −9.56 to −12.88 (m, 8B, B3, 4, 5, 6, 7, 8, 10, 11); UV (99.8% EtOH): λ_max_ (nm) = 261, 282, 326, and 442, and λ_min_ = 242, 267, 317, and 350; FT-IR: *ν*_max_ (cm^−1^) = 2923 (C-H_aliphat_), 2577 (B-H), 1680 (C=O), 1636 (C=O), and 722 (B-B); RP-HPLC (gradient B): *t*_R_ = 21.70 min; APCI-MS: *m/z*: 607 [M + H]^+^, calcd for C_29_H_46_B_10_N_4_O_3_: 606.

*N-[2-(N-Pyrrolidinyl)ethyl]-4-[(6-(3-(1,2-dicarba-closo-dodecaborane-1-yl)propanamido)hexyl)amino]-1,8-naphthalimide* (**43**): yellow solid, yield 14.4 mg (45%). TLC (MeOH/CH_2_Cl_2_, 3:17, *v/v*): *R*_f_ = 0.27. ^1^H NMR (CDCl_3_, 600.26 MHz): *δ* (ppm) = 8.51 (d, *J* = 7.3 Hz, 1H, H_arom_), 8.38 (d, *J* = 8.5 Hz, 1H, H_arom_), 8.15 (d, *J* = 8.5 Hz, 1H, H_arom_), 7.55 (t, *J* = 7.8 Hz, 1H, H_arom_), 6.63 (d, *J* = 8.5 Hz, 1H, H_arom_), 5.68–5.63 (m, 2H, 2NH), 4.37 (t, *J* = 7.3 Hz, 2H, CH_2_-N(CO)_2_), 3.36 (q, *J* = 7.2 Hz, 2H, C*H*_2_-NH), 3.25 (q, *J* = 7.0 Hz, 2H, C*H*_2_-NH), 2.92–2.90 (m, 3H, CH_2_-pyrrolidine overlapped with CH_carborane_), 2.80 (br s, 4H, N-C*H*_2pyrrolidine_-CH_2_), 2.35–2.32 (m, 2H, CH_2_-carborane), 2.23–2.20 (m, 2H, NH-C(O)-C*H*_2_), 1.85–1.83 (m, 4H, CH_2_-C*H*_2pyrrolidine_-CH_2_), 1.80–1.75 (m, *J* = 7.3 Hz, 2H, CH_2_-C*H*_2_-CH_2_), 1.55–1.48 (m, 4H, CH_2_-C*H*_2_-CH_2_), 1.42–1.39 (m, 2H, CH_2_-C*H*_2_-CH_2_), and 3.0–1.5 (m, 10H, B_10_H_10_); ^13^C NMR (CDCl_3_, 150.95 MHz): *δ* (ppm) = 170.60 (1C, C(O)-NH), 164.84–164.20 (2C, C11 + C12), 149.83–104.31 (10C, 10C_arom_), 75.14 (1C, C_carborane_), 55.18 (1C, CH_carborane_), 54.41 (2C, N-*C*H_2pyrrolidine_-CH_2_), 53.78 (1C, CH_2_-pyrrolidine), 43.39 (1C, CH_2_-NH), 39.27 (1C, CH_2_-NH), 38.55 (1C, *C*H_2_-N(CO)_2_), 36.31 (1C, CH_2_-carborane), 32.09 (1C, NH-C(O)-*C*H_2_), 29.64 (1C, CH_2_-*C*H_2_-CH_2_), 28.70 (1C, CH_2_-*C*H_2_-CH_2_), 26.48 (1C, CH_2_-*C*H_2_-CH_2_), 26.31 (1C, CH_2_-*C*H_2_-CH_2_), and 23.73 (2C, CH_2_-*C*H_2pyrrolidine_-CH_2_); ^11^B NMR {^1^H BB} (CDCl_3_, 192.59 MHz): *δ* (ppm) = −4.35 (s, 1B, B5), −9.81 to −11.02 (m, 5B, B4, 6, 9, 10, 12), −13.52 (s, 2B, B8, 11), and −15.20 (s, 2B, B2, 3); UV (99.8% EtOH): λ_max_ (nm) = 261, 282, 326, and 442, and λ_min_ = 242, 267, 317, and 350; FT-IR: *ν*_max_ (cm^−1^) = 2921 (C-H_aliphat_), 2593 (B-H), 1681 (C=O), 1637 (C=O), and 728 (B-B); RP-HPLC (gradient B): *t*_R_ = 21.49 min; APCI-MS: *m/z*: 607 [M + H]^+^, calcd for C_29_H_46_B_10_N_4_O_3_: 606.

#### 3.1.7. Synthesis of 1,8-Naphthalimide Derivatives **54** and **61** Modified with Carborane Cluster via Reductive Amination

*N*-[2-(Dimethylamino)ethyl]-4-[(2-aminoethyl)amino]-1,8-naphthalimide (**30**), *N*-[2-(*N*-pyrrolidinyl)ethyl]-4-[(2-aminoethyl)amino]-1,8-naphthalimide (**31**), *N*-[2-(dimethylamino)ethyl]-4-[(6-aminohexyl)amino]-1,8-naphthalimide (**32**) or *N*-[2-(*N*-pyrrolidinyl)ethyl]-4-[(6-aminohexyl)amino]-1,8-naphthalimide (**33**) (1 equiv.) was dissolved in dry THF, dry AcOEt (for synthesis of compound **55**) or dry MeOH (for synthesis of compounds **57**, **59**, and **61**) (2.4 mL per 0.1 mmol) and 2-(1,2-dicarba-*closo*-dodecaborane-1-yl)ethanal (**44**) or 2-(1,7-dicarba-*closo*-dodecaborane-1-yl)ethanal (**45**) (1.2 equiv.) was added. The reaction mixture was stirred for 24 h at reflux under an inert (Ar) atmosphere. Next, to the Schiff base, **46**–**53** NaBH_3_CN (3 equiv.) was added, and the reaction mixture was stirred for 24 h at RT under an inert (Ar) atmosphere. Subsequently, the solvent was evaporated to dryness under vacuum, and the crude product was purified by column chromatography on silica gel (230–400 mesh) with a gradient using MeOH (5–20%) in CH_2_Cl_2_. Additionally, the purified product was dissolved in MeOH/CH_2_Cl_2_ (1:39, *v/v*) and washed with the same volume of NaHCO_3_ (2.5%) and three times with water. The organic phase was separated, dried over MgSO_4_, filtered, and evaporated to dryness. Then, the product was purified again by column chromatography using the same condition as described above.

*N-[2-(Dimethylamino)ethyl]-4-[(2-(2-(1,2-dicarba-closo-dodecaborane-1-yl)-ethylamino)ethyl)amino]-1,8-naphthalimide* (**54**): yellow solid. Yield 10.3 mg (45%). TLC (MeOH/CH_2_Cl_2_, 1:4, *v/v*): *R*_f_ = 0.26. ^1^H NMR (MeOH-d_4_, 600.26 MHz): *δ* (ppm) = 8.46 (m, 2H, 2H_arom_), 8.30 (d, *J* = 8.5 Hz, 1H, H_arom_), 7.63–7.60 (m, 1H, H_arom_), 6.79 (d, *J* = 8.7 Hz, 1H, H_arom_), 4.66 (br s, 1H, CH_carborane_), 4.31 (t, *J* = 6.9 Hz, 2H, CH_2_-N(CO)_2_), 3.56 (t, *J* = 6.2 Hz, 2H, C*H*_2_-NH), 2.96 (t, *J* = 6.2 Hz, 2H, C*H*_2_-NH), 2.83–2.81 (m, 4H, C*H*_2_-CH_2_-carborane overlapped with C*H*_2_-N(CH_3_)_2_), 2.50–2.46 (m, 8H, CH_2_-carborane overlapped with N(CH_3_)_2_), and 3.0–1.5 (m, 10H, B_10_H_10_); ^13^C NMR (MeOH-d_4_, 150.95 MHz): *δ* (ppm) = 166.27–165.66 (2C, C11 + C12), 152.52–105.17 (10C, 10C_arom_), 75.28 (1C, C_carborane_), 63.45 (1C, CH_carborane_), 57.89 (1C, *C*H_2_-N(CH_3_)_2_), 50.76 (1C, CH_2_-NH), 49.57 (1C, CH_2_-NH overlapped with signal from MeOD), 45.41 (2C, 2 x CH_3_), 43.78 (1C, *C*H_2_-CH_2_-carborane), and 37.97–37.91 (2C, *C*H_2_-N(CO)_2_ and CH_2_-carborane); ^11^B NMR {^1^H BB} (MeOH-d_4_, 192.59 MHz): *δ* (ppm) = −2.78 (s, 1B, B9), −5.79 (s, 1B, B12), −9.71 (s, 2B, B8, 10), and −11.42 to −13.06 (m, 6B, B3, 4, 5, 6, 7, 11); UV (99.8% EtOH): λ_max_ (nm) = 261, 280, and 437, λ_min_ = 241, 266, and 349, and λ_sh_ = 326; FT-IR: *ν*_max_ (cm^−1^) = 2943 (C-H_aliphat_), 2577 (B-H), 1681 (C=O), 1637 (C=O), and 723 (B-B); RP-HPLC (gradient B): *t*_R_ = 16.95 min; APCI-MS: *m/z*: 497 [M + H]^+^, calcd for C_22_H_36_B_10_N_4_O_2_: 496; HRMS (ESI+) 497.3926 [M + H]^+^, calcd for C_22_H_36_B_10_N_4_O_2_ = 496.3841.

*N-[2-(Dimethylamino)ethyl]-4-[(2-(2-(1,7-dicarba-closo-dodecaborane-1-yl)-ethylamino)ethyl)amino]-1,8-naphthalimide* (**55**): yellow solid, yield 9.5 mg (48%). TLC (MeOH/CH_2_Cl_2_, 1:4, *v/v*): *R*_f_ = 0.27. ^1^H NMR (DMSO-d_6_, 600.26 MHz): *δ* (ppm) = 8.65 (d, *J* = 8.5 Hz, 1H, H_arom_), 8.42 (d, *J* = 7.3 Hz, 1H, H_arom_), 8.24 (d, *J* = 8.7 Hz, 1H, H_arom_), 7.68–7.65 (m, 2H, H_arom_ overlapped with NH), 6.78 (d, *J* = 8.7 Hz, 1H, H_arom_), 6.55 (br s, 1H, NH), 4.12 (t, *J* = 6.9 Hz, 2H, CH_2_-N(CO)_2_), 4.01 (br s, 1H, CH_carborane_), 3.42–3.40 (m, 2H, C*H*_2_-NH overlapped with signal from H_2_O), 2.81 (t, *J* = 6.4, 2H, C*H*_2_-NH), 2.56–2.54 (m, 2H, C*H*_2_-CH_2_-carborane), 2.50–2.48 (m, 2H, C*H*_2_-N(CH_3_)_2_ overlapped with signal from DMSO), 2.21 (s, 6H, N(CH_3_)_2_), 2.10–2.07 (m, 2H, CH_2_-carborane), and 3.0–1.5 (m, 10H, B_10_H_10_); ^13^C NMR (DMSO-d_6_, 150.95 MHz): *δ* (ppm) = 164.23–163.36 (2C, C11 + C12), 151.16–104.35 (10C, 10C_arom_), 75.31 (1C, C_carborane_), 57.11 (1C, *C*H_2_-N(CH_3_)_2_), 56.75 (1C, CH_carborane_), 49.30 (1C, CH_2_-NH), 47.48 (1C, CH_2_-NH), 45.81 (2C, 2 x CH_3_), 43.39 (1C, *C*H_2_-CH_2_-carborane), 37.60 (1C, *C*H_2_-N(CO)_2_), and 36.46 (1C, CH_2_-carborane); ^11^B NMR {^1^H BB} (DMSO-d_6_, 192.59 MHz): *δ* (ppm) = −4.64 (s, 1B, B5), −11.29 (br s, 5B, B4, 6, 9, 10, 12), and −13.79 to −15.18 (m, 4B, B2, 3, 8, 11); UV (99.8% EtOH): λ_max_ (nm) = 261, 280, and 437, λ_min_ = 241, 266 and 349, and λ_sh_ = 325; FT-IR: *ν*_max_ (cm^−1^) = 2942 (C-H_aliphat_), 2596 (B-H), 1679 (C=O), 1638 (C=O), and 729 (B-B); RP-HPLC (gradient B): *t*_R_ = 16.71 min; APCI-MS: *m/z*: 497 [M + H]^+^, calcd for C_22_H_36_B_10_N_4_O_2_: 496; HRMS (ESI+) 497.3928 [M + H]^+^, calcd for C_22_H_36_B_10_N_4_O_2_ = 496.3841.

*N-[2-(Pyrrolidin-1-yl)ethyl]-4-[(2-(2-(1,2-dicarba-closo-dodecaborane-1-yl)-ethylamino)ethyl)amino]-1,8-naphthalimide* (**56**): yellow solid, yield 11.7 mg (49%). TLC (MeOH/CH_2_Cl_2_, 1:4, *v/v*): *R*_f_ = 0.27. ^1^H NMR (MeOH-d_4_, 600.26 MHz): *δ* (ppm) = 8.52–8.49 (m, 2H, 2H_arom_), 8.34 (d, *J* = 8.7 Hz, 1H, H_arom_), 7.64 (dd, *J* = 8.5, 7.3 Hz, 1H, H_arom_), 6.81 (d, *J* = 8.7 Hz, 1H, H_arom_), 4.67 (br s, 1H, CH_carborane_), 4.43 (t, *J* = 6.3 Hz, 2H, CH_2_-N(CO)_2_), 3.57 (t, *J* = 6.2 Hz, 2H, C*H*_2_-NH), 3.33 (t, *J* = 6.2 Hz, 2H, CH_2_-pyrrolidine), 3.27 (br s, 4H, N-C*H*_2pyrrolidine_-CH_2_), 2.98 (t, *J* = 6.2 Hz, 2H, C*H*_2_-NH), 2.83–2.80 (m, 2H, C*H*_2_-CH_2_-carborane), 2.49–2.47 (m, 2H, CH_2_-carborane), 2.01 (t, *J* = 7.0 Hz, 4H, CH_2_-C*H*_2pyrrolidine_-CH_2_), and 3.0–1.5 (m, 10H, B_10_H_10_); ^13^C NMR (MeOH-d_4_, 150.95 MHz): *δ* (ppm) = 166.46–165.72 (2C, C11 + C12), 152.71–105.23 (10C, 10C_arom_), 75.25 (1C, C_carborane_), 63.46 (1C, CH_carborane_), 55.73 (2C, N-*C*H_2pyrrolidine_-CH_2_), 54.97 (1C, CH_2_-pyrrolidine), 49.28–48.86 (2C, 2CH_2_-NH overlapped with signal from MeOD), 43.74 (1C, *C*H_2_-CH_2_-carborane), 38.15 (1C, CH_2_-carborane), 37.84 (1C, *C*H_2_-N(CO)_2_), and 24.08 (2C, CH_2_-*C*H_2pyrrolidine_-CH_2_); ^11^B NMR {^1^H BB} (MeOH-d_4_, 192.59 MHz): *δ* (ppm) = −2.81 (s, 1B, B9), −5.90 (s, 1B, B12), −9.78 (s, 2B, B8, 10), and −11.59 to −13.13 (m, 6B, B3, 4, 5, 6, 7, 11); UV (99.8% EtOH): λ_max_ (nm) = 254, 280, and 437, λ_min_ = 239, 267, and 335, and λ_sh_ = 323; FT-IR: *ν*_max_ (cm^−1^) = 2956 (C-H_aliphat_), 2577 (B-H), 1681 (C=O), 1637 (C=O), and 723 (B-B); RP-HPLC (gradient B): *t*_R_ = 17.29 min; APCI-MS: *m/z*: 524 [M + H]^+^, calcd for C_24_H_38_B_10_N_4_O_2_: 523; HRMS (ESI+) 523.4081 [M + H]^+^, calcd for C_24_H_38_B_10_N_4_O_2_ = 522.3998.

*N-[2-(Pyrrolidin-1-yl)ethyl]-4-[(2-(2-(1,7-dicarba-closo-dodecaborane-1-yl)-ethylamino)ethyl)amino]-1,8-naphthalimide* (**57**): yellow solid, yield 15.4 mg (47%). TLC (MeOH/CH_2_Cl_2_, 1:4, *v/v*): *R*_f_ = 0.26. ^1^H NMR (MeOH-d_4_, 600.26 MHz): *δ* (ppm) = 8.38–8.35 (m, 2H, 2H_arom_), 8.20 (d, *J* = 8.7 Hz, 1H, H_arom_), 7.53 (dd, *J* = 8.4, 7.4 Hz, 1H, H_arom_), 6.70 (d, *J* = 8.7 Hz, 1H, H_arom_), 4.28 (t, *J* = 7.1 Hz, 2H, CH_2_-N(CO)_2_), 3.53–3.51 (m, 3H, CH_carborane_ overlapped C*H*_2_-NH), 2.95–2.94 (m, 4H, CH_2_-pyrrolidine overlapped with C*H*_2_-NH), 2.87 (br s, 4H, N-C*H*_2pyrrolidine_-CH_2_), 2.71–2.68 (m, 2H, C*H*_2_-CH_2_-carborane), 2.23–2.20 (m, 2H, CH_2_-carborane), 1.89–1.87 (m, 4H, CH_2_-C*H*_2pyrrolidine_-CH_2_), and 3.0–1.5 (m, 10H, B_10_H_10_); ^13^C NMR (MeOH-d_4_, 150.95 MHz): *δ* (ppm) = 166.10–165.47 (2C, C11 + C12), 152.33–105.06 (10C, 10C_arom_), 75.38 (1C, C_carborane_), 57.04 (1C, CH_carborane_), 55.39 (2C, N-*C*H_2pyrrolidine_-CH_2_), 54.84 (1C, CH_2_-pyrrolidine), 50.21 (1C, CH_2_-NH), 48.43 (1C, CH_2_-NH overlapped with signal from MeOD), 43.75 (1C, *C*H_2_-CH_2_-carborane), 39.06 (1C, CH_2_-carborane), 37.16 (2C, *C*H_2_-N(CO)_2_), and 24.20 (2C, CH_2_-*C*H_2pyrrolidine_-CH_2_); ^11^B NMR {^1^H BB} (MeOH-d_4_, 192.59 MHz): *δ* (ppm) = −4.43 (s, 1B, B5), −9.73 to −10.97 (m, 5B, B4, 6, 9, 10, 12), −13.57 (s, 2B, B8, 11), and −15.16 (s, 2B, B2, 3); UV (99.8% EtOH): λ_max_ (nm) = 260, 280, and 437, λ_min_ = 241, 267, and 349, and λ_sh_ = 325; FT-IR: *ν*_max_ (cm^−1^) = 2954 (C-H_aliphat_), 2592 (B-H), 1681 (C=O), 1639 (C=O), and 729 (B-B); RP-HPLC (gradient B): *t*_R_ = 16.78 min; APCI-MS: *m/z*: 524 [M + H]^+^, calcd for C_24_H_38_B_10_N_4_O_2_: 523; HRMS (ESI+) 523.4084 [M + H]^+^, calcd for C_24_H_38_B_10_N_4_O_2_ = 522.3998.

*N-[2-(Dimethylamino)ethyl]-4-[(6-(2-(1,2-dicarba-closo-dodecaborane-1-yl)-ethylamino)hexyl)amino]-1,8-naphthalimide* (**58**): yellow solid, yield 6.8 mg (24%). TLC (MeOH/CH_2_Cl_2_, 1:4, *v/v*): *R*_f_ = 0.17. ^1^H NMR (MeOH-d_4_, 600.26 MHz): *δ* (ppm) = 8.47–8.43 (m, 2H, 2H_arom_), 8.28 (d, *J* = 8.5 Hz, 1H, H_arom_), 7.58 (dd, *J* = 8.3, 7.3 Hz, 1H, H_arom_), 6.71 (d, *J* = 8.7, 1H, H_arom_), 4.63 (br s, 1H, CH_carborane_), 4.28 (t, *J* = 7.0 Hz, 2H, CH_2_-N(CO)_2_), 3.42 (td, *J* = 12.4, 5.3 Hz, 2H, C*H*_2_-NH), 2.73–2.70 (m, 4H, C*H*_2_-N(CH_3_)_2_ overlapped with C*H*_2_-CH_2_-carborane), 2.60–2.58 (m, 2H, C*H*_2_-NH), 2.45–2.41 (m, 8H, CH_2_-carborane overlapped with N(CH_3_)_2_), 1.81–1.78 (m, 2H, CH_2_-C*H*_2_-CH_2_), 1.55–1.50 (m, 4H, 2(CH_2_-C*H*_2_-CH_2_)), 1.45–1.43 (m, 2H, CH_2_-C*H*_2_-CH_2_), and 3.0–1.5 (m, 10H, B_10_H_10_); ^13^C NMR (MeOH-d_4_, 150.95 MHz): *δ* (ppm) = 166.25–165.64 (2C, C11 + C12), 152.69–105.00 (10C, 10C_arom_), 74.95 (1C, C_carborane_), 63.67 (1C, CH_carborane_), 57.86 (1C, *C*H_2_-N(CH_3_)_2_), 50.22 (1C, CH_2_-NH), 49.43–48.57 (1C, CH_2_-NH overlapped with signal from MeOD), 45.61 (2C, 2 x CH_3_), 44.31 (1C, *C*H_2_-CH_2_-carborane), 38.23 (1C, *C*H_2_-N(CO)_2_), 37.43 (1C, CH_2_-carborane), 30.22 (1C, CH_2_-*C*H_2_-CH_2_), 29.41 (1C, CH_2_-*C*H_2_-CH_2_), 28.07 (1C, CH_2_-*C*H_2_-CH_2_), and 28.04 (1C, CH_2_-*C*H_2_-CH_2_); ^11^B NMR {^1^H BB} (MeOH-d_4_, 192.59 MHz): *δ* (ppm) = −2.75 (s, 1B, B9), −5.69 (s, 1B, B12), −9.63 (s, 2B, B8, 10), and −11.60 to −13.04 (m, 6B, B3, 4, 5, 6, 7, 11); UV (99.8% EtOH): λ_max_ (nm) = 262, 281, and 442, λ_min_ = 242, 267, and 349, and λ_sh_ = 325; FT-IR: *ν*_max_ (cm^−1^) = 2919 (C-H_aliphat_), 2578 (B-H), 1681 (C=O), 1638 (C=O), and 722 (B-B); RP-HPLC (gradient B): *t*_R_ = 17.70 min; APCI-MS: *m/z*: 553 [M + H]^+^, calcd for C_26_H_44_B_10_N_4_O_2_: 552; HRMS (ESI+) 553.4558 [M + H]^+^, calcd for C_26_H_44_B_10_N_4_O_2_ = 552.4467.

*N-[2-(Dimethylamino)ethyl]-4-[(6-(2-(1,7-dicarba-closo-dodecaborane-1-yl)-ethylamino)hexyl)amino]-1,8-naphthalimide* (**59**): yellow solid; yield 11.6 mg (45%). TLC (MeOH/CH_2_Cl_2_, 1:4, *v/v*): *R*_f_ = 0.18. ^1^H NMR (DMSO-d_6_, 600.26 MHz): *δ* (ppm) = 8.70 (d, *J* = 8.5 Hz, 1H, H_arom_), 8.42 (d, *J* = 7.2 Hz, 1H, H_arom_), 8.26 (d, *J* = 7.3 Hz, 1H, H_arom_), 7.77–7.76 (m, 1H, NH), 7.68–7.66 (m, 1H, H_arom_), 6.76 (d, *J* = 8.0, 1H, H_arom_), 6.55 (br s, 1H, NH), 4.12 (t, *J* = 7.0 Hz, 2H, CH_2_-N(CO)_2_), 4.02 (br s, 1H, CH_carborane_), 3.35 (br s, 2H, C*H*_2_-NH overlapped with signal from H_2_O), 2.52–2.44 (m, 6H, C*H*_2_-N(CH_3_)_2,_ overlapped with signal from C*H*_2_-CH_2_-carborane, C*H*_2_-NH and DMSO), 2.20 (s, 6H, N(CH_3_)_2_), 2.07–2.04 (m, 2H, CH_2_-carborane), 1.70–1.68 (m, 2H, CH_2_-C*H*_2_-CH_2_), 1.39–1.32 (m, 6H, 3(CH_2_-C*H*_2_-CH_2_)), and 3.0–1.5 (m, 10H, B_10_H_10_); ^13^C NMR (DMSO-d_6_, 150.95 MHz): *δ* (ppm) = 164.24–163.36 (2C, C11 + C12), 151.17–104.24 (10C, 10C_arom_), 75.27 (1C, C_carborane_), 57.14 (1C, *C*H_2_-N(CH_3_)_2_), 56.75 (1C, CH_carborane_), 49.37 (1C, CH_2_-NH), 49.25 (1C, CH_2_-NH), 45.86 (2C, 2 × CH_3_), 43.28 (1C, *C*H_2_-CH_2_-carborane), 37.63 (1C, *C*H_2_-N(CO)_2_), 36.10 (1C, CH_2_-carborane), 29.62 (1C, CH_2_-*C*H_2_-CH_2_), 28.27 (1C, CH_2_-*C*H_2_-CH_2_), 26.99 (1C, CH_2_-*C*H_2_-CH_2_), and 26.97 (1C, CH_2_-*C*H_2_-CH_2_); ^11^B NMR {^1^H BB} (DMSO-d_6_, 192.59 MHz): *δ* (ppm) = −4.44 (s, 1B, B5), −11.18 (br s, 5B, B4, 6, 9, 10, 12), −13.66 (s, 2B, B8, 11), and −15.02 (s, 2B, B2, 3); UV (99.8% EtOH): λ_max_ (nm) = 261, 282, and 442, λ_min_ = 241, 267, and 351, and λ_sh_ = 326; FT-IR: *ν*_max_ (cm^−1^) = 2930 (C-H_aliphat_), 2591 (B-H), 1681 (C=O), 1640 (C=O), and 729 (B-B); RP-HPLC (gradient B): *t*_R_ = 17.50 min; APCI-MS: *m/z*: 553 [M + H]^+^, calcd for C_26_H_44_B_10_N_4_O_2_: 552; HRMS (ESI+) 553.4555 [M + H]^+^, calcd for C_26_H_44_B_10_N_4_O_2_ = 552.4467.

*N-[2-N-Pyrrolidinyl)ethyl]-4-[(6-(2-(1,2-dicarba-closo-dodecaborane-1-yl)-ethylamino)hexyl)amino]-1,8-naphthalimide* (**60**): yellow solid, yield 13.7 mg (39%). TLC (MeOH/CH_2_Cl_2_, 1:4, *v/v*): *R*_f_ = 0.27. ^1^H NMR (MeOH-d_4_, 600.26 MHz): *δ* (ppm) = 8.57–8.51 (m, 2H, 2H_arom_), 8.35 (d, *J* = 8.7 Hz, 1H, H_arom_), 7.64 (dd, *J* = 8.4, 7.4 Hz, 1H, H_arom_), 6.78 (d, *J* = 8.7, 1H, H_arom_), 4.69 (br s, 1H, CH_carborane_), 4.45 (t, *J* = 6.1 Hz, 2H, CH_2_-N(CO)_2_), 3.47 (d, *J* = 7.2 Hz, 2H, C*H*_2_-NH), 3.41–3.40 (m, *J* = 7.2 Hz, 2H, CH_2_-pyrrolidine), 3.33 (br s, 4H, N-C*H*_2pyrrolidine_-CH_2_), 2.94–2.91 (m, 2H, C*H*_2_-CH_2_-carborane), 2.81–2.78 (m, 2H, C*H*_2_-NH), 2.58–2.55 (m, 2H, CH_2_-carborane), 2.04 (br s, 4H, CH_2_-C*H*_2pyrrolidine_-CH_2_), 1.82–1.80 (m, 2H, CH_2_-C*H*_2_-CH_2_), 1.64–1.61 (m, 2H, CH_2_-C*H*_2_-CH_2_), 1.55–1.47 (m, 4H, 2(CH_2_-C*H*_2_-CH_2_)), and 3.0–1.5 (m, 10H, B_10_H_10_); ^13^C NMR (MeOH-d_4_, 150.95 MHz): *δ* (ppm) = 166.57–165.73 (2C, C11 + C12), 153.00–105.13 (10C, 10C_arom_), 77.92 (1C, C_carborane_), 63.81 (1C, CH_carborane_), 55.79 (2C, N-*C*H_2pyrrolidine_-CH_2_), 55.01 (1C, CH_2_-pyrrolidine), 49.57 (2C, 2CH_2_-NH overlapped with signal from MeOD), 44.26 (1C, *C*H_2_-CH_2_-carborane), 37.34–37.91 (2C, *C*H_2_-N(CO)_2_ and CH_2_-carborane), 29.31 (1C, CH_2_-*C*H_2_-CH_2_), 28.80 (1C, CH_2_-*C*H_2_-CH_2_), 27.86 (1C, CH_2_-*C*H_2_-CH_2_), 27.65 (1C, CH_2_-*C*H_2_-CH_2_), and 24.06 (2C, CH_2_-*C*H_2pyrrolidine_-CH_2_); ^11^B NMR {^1^H BB} (MeOH-d_4_, 192.59 MHz): *δ* (ppm) = −2.73 (s, 1B, B9), −5.72 (s, 1B, B12), −9.56 (s, 2B, B8, 10), and −11.99 to −13.03 (m, 6B, B3, 4, 5, 6, 7, 11); UV (99.8% EtOH): λ_max_ (nm) = 260, 282, and 442, λ_min_ = 241, 267, and 349, and λ_sh_ = 326; FT-IR: *ν*_max_ (cm^−1^) = 2930 (C-H_aliphat_), 2566 (B-H), 1681 (C=O), 1638 (C=O), and 722 (B-B); RP-HPLC (gradient B): *t*_R_ = 17.99 min; APCI-MS: *m/z*: 579 [M + H]^+^, calcd for C_28_H_46_B_10_N_4_O_2_: 578; HRMS (ESI+) 579.4711 [M + H]^+^, calcd for C_28_H_46_B_10_N_4_O_2_ = 578.4624.

*N-[2-N-Pyrrolidinyl)ethyl]-4-[(6-(2-(1,7-dicarba-closo-dodecaborane-1-yl)-ethylamino)hexyl)amino]-1,8-naphthalimide* (**61**): yellow solid, yield 13.9 mg (49%). TLC (MeOH/CH_2_Cl_2_, 1:4, *v/v*): *R*_f_ = 0.30. ^1^H NMR (MeOH-d_4_, 600.26 MHz): *δ* (ppm) = 8.54–8.49 (m, 2H, 2H_arom_), 8.33 (d, *J* = 8.7 Hz, 1H, H_arom_), 7.63 (dd, *J* = 8.4, 7.4 Hz 1H, H_arom_), 6.78 (d, *J* = 8.7, 1H, H_arom_), 4.39 (t, *J* = 6.6 Hz, 2H, CH_2_-N(CO)_2_), 3.56 (br s, 1H, CH_carborane_), 3.46 (t, *J* = 7.2 Hz, 2H, C*H*_2_-NH), 3.15 (t, *J* = 6.6 Hz, 2H, CH_2_-pyrrolidine), 3.06 (br s, 4H, N-C*H*_2pyrrolidine_-CH_2_), 2.75–2.71 (m, 4H, C*H*_2_-CH_2_-carborane overlapped with signal from C*H*_2_-NH), 2.26–2.23 (m, 2H, CH_2_-carborane), 1.95 (br s, 4H, CH_2_-C*H*_2pyrrolidine_-CH_2_), 1.82–1.80 (m, 2H, CH_2_-C*H*_2_-CH_2_), 1.60–1.58 (m, 2H, CH_2_-C*H*_2_-CH_2_), 1.52–1.45 (m, 4H, 2(CH_2_-C*H*_2_-CH_2_)), and 3.0–1.5 (m, 10H, B_10_H_10_); ^13^C NMR (MeOH-d_4_, 150.95 MHz): *δ* (ppm) = 166.44–165.75 (2C, C11 + C12), 152.88–105.08 (10C, 10C_arom_), 74.37 (1C, CH_carborane_), 57.19 (1C, CH_carborane_), 55.55 (2C, N-*C*H_2pyrrolidine_-CH_2_), 54.93 (1C, CH_2_-pyrrolidine), 49.83 (2C, 2CH_2_-NH overlapped with signal from MeOD), 44.26 (1C, *C*H_2_-CH_2_-carborane), 38.62 (1C, *C*H_2_-N(CO)_2_), 35.49 (1C, CH_2_-carborane), 29.32 (1C, CH_2_-*C*H_2_-CH_2_), 29.21 (1C, CH_2_-*C*H_2_-CH_2_), 27.91 (1C, CH_2_-*C*H_2_-CH_2_), 27.75 (1C, CH_2_-*C*H_2_-CH_2_), and 24.14 (2C, CH_2_-*C*H_2pyrrolidine_-CH_2_); ^11^B NMR {^1^H BB} (MeOH-d_4_, 192.59 MHz): *δ* (ppm) = −4.55 (s, 1B, B5), −9.72 to −10.96 (m, 5B, B4, 6, 9, 10, 12), −13.49 (s, 2B, B8, 11), and −15.18 (s, 2B, B2, 3); UV (99.8% EtOH): λ_max_ (nm) = 261, 282, and 442, λ_min_ = 241, 269, and 350, and λ_sh_ = 323; FT-IR: *ν*_max_ (cm^−1^) = 2929 (C-H_aliphat_), 2590 (B-H), 1681 (C=O), 1637 (C=O), and 727 (B-B); RP-HPLC (gradient B): *t*_R_ = 17.79 min; APCI-MS: *m/z*: 579 [M + H]^+^, calcd for C_28_H_46_B_10_N_4_O_2_: 578; HRMS (ESI+) 579.4711 [M + H]^+^, calcd for C_28_H_46_B_10_N_4_O_2_ = 578.4624.

### 3.2. Physicochemical Investigation with DNA

#### 3.2.1. Materials

Calf thymus (ct-DNA) was purchased from Sigma (St. Louis, MO, USA) and used without purification. Sodium cacodylate (for the preparation of cacodylate buffer) was purchased from Acros Organics (Geel, Belgium). Water was obtained from a Milli-Q purification system. All experiments were performed with freshly prepared solutions.

#### 3.2.2. Preparation of ct-DNA

ct-DNA was dissolved in H_2_O reconstituted overnight at 4 °C to dissolve all the material. The molar concentration of ct-DNA was determined from UV–visible spectra using a molar absorption coefficient (ε) of 6600 M^−1^ cm^−1^ at 260 nm [52]. The purity of ct-DNA was confirmed by UV–visible spectroscopy by measuring the absorbance ratio at 260 to 280 nm and was found to be ≥1.8, indicating that DNA was sufficiently free of proteins.

#### 3.2.3. Melting Temperature (*T*_m_) Measurements

The measurements were performed by adding aliquots of DMSO stock solution of the tested compounds to the buffer solution (pH 7.0, 20 mM, cacodylate buffer, DMSO content of the final solution = 0.24–0.36%). The *T*_m_ curves were collected at r = 0.3 (r = [compound]/[ct-DNA]) to assure the dominant binding mode. Thermal melting curves were determined by following the absorption change at 260 nm as a function of temperature by using a GBC Cintra10 UV–vis spectrometer (Dandenong, Australia) equipped with a GBC Thermocell Peltier Power Supply (Dandenong, Australia) using a 1 cm path length cell. The absorbance of the samples was monitored at 260 nm from 30 to 90 °C (95 °C for compounds **54**–**61**) with a heating rate of 1 °C/min. *T*_m_ values are the midpoints or the transition curves determined from the maximum of the first derivative. The Δ*T*_m_ values were calculated by subtracting the *T*_m_ of the free nucleic acid from the *T*_m_ of the sample. Every *T*_m_ value reported in this study was average at least three measurements. The error in *T*_m_ was 0.5 °C.

#### 3.2.4. Circular Dichroism (CD) Measurements

The measurements were performed by adding aliquots of DMSO stock solution of the tested compounds to the buffer solution (pH 7.0, 20 mM, cacodylate buffer, DMSO content of the final solutions = 0.15–1.22%). Changes in the CD spectrum of ct-DNA upon the addition of compound were measured at different molar ratios r = [compound]/[ct-DNA]. Circular dichroism spectra were recorded on a JASCO J-815 CD spectrometer with JASCO PFD-425S Peltier thermostated cell holder (Tokyo, Japan) using a rectangular quartz cuvette of path length 0.5 cm (1 mL) in the 230–400 nm region. The reported CD profiles are an average of three successive scans with 200 nm per minute scan time and an appropriately corrected baseline. The temperature was maintained at 20 °C during the experiment.

#### 3.2.5. Ultraviolet-Visible (UV–Vis) Spectra Titration

The measurements were performed by adding aliquots of DMSO stock solution of the tested compounds to the buffer solution (pH 7.4, 20 mM, 50 mM NaCl, Tris-HCl buffer, DMSO content of the final solutions = 0.40–0.61%) to the final concentration of 10 µM. The tested compounds were incubated 5 min, with increasing concentrations ranging from 0 to 15 µM of ct-DNA at 37 °C, then the UV–vis absorption spectra, between 310 and 500 nm (310–550 nm for compounds **36**–**43** and **54**–**61**), were recorded using a GBC Cintra10 UV–vis spectrometer (Dandenong, Australia) equipped with a GBC Thermocell Peltier Power Supply (Dandenong, Australia) using a 1 cm path length cell. The binding constant was calculated according to the following equation [53]:

$$\frac{A_{0}}{A-A_{0}}=\frac{\varepsilon_{G}}{\varepsilon_{H-G}-\varepsilon_{G}\;}+\frac{\varepsilon_{G}}{\varepsilon_{H-G}-\varepsilon_{G}\;}\times \frac{1}{K_{b}\left[\mathrm{DNA}\right]}$$

where K*_b_* is the binding constant, *A*_0_ and *A* are the absorbance of the free tested compound, and the apparent one, ε_G_ and ε_H−G_ are their coefficient, respectively, and [DNA] is the concentration of [DNA] in the base pair. The slope to intercept ratio of the plot between *A*_0_/*A* − *A*_0_ versus 1/[DNA] yielded the binding constant.

### 3.3. Biological Investigation

#### 3.3.1. Cytotoxicity Assay

The cytotoxic properties of the synthesized compounds were evaluated using the human hepatocellular carcinoma cell line HepG2 established from hepatocellular carcinoma. The HepG2 cell line was purchased from ECACC (Salisbury, UK). Cells were grown in EMEM medium (Corning^®^) supplemented with 10% heat-inactivated fetal bovine serum (FBS) (Corning^®^) and antibiotics (Corning^®^), at 37 °C in a 5% CO_2_ atmosphere. Upon reaching 80–90% confluency, cells were detached with the trypsin (Corning^®^) and transferred into 96-well microplates at a density of 12 × 10^3^. After overnight incubation at 37 °C in a humidified atmosphere containing 5% CO_2_, the cells were treated for the next 24 h with compounds. The stock solution of each compound was prepared in DMSO at 10 mM. The cytotoxicity was evaluated by the MTT assay. The final content of DMSO in solutions did not exceed 0.2%, and an additional control group with 0.2% DMSO was included to rule out the effect of solvent. Subsequently, the medium was aspirated and replaced with 3-(4,5-dimethylthiazol-2-yl)-2,5-diphenyltetrazolium bromide (MTT) dye solution (50 µL, 0.5 mg/mL). After 3 h incubation, the resulting MTT formazan crystals were dissolved in DMSO (100 µL). To ensure the complete dissolution of formazan, the plates were shaken on an orbital microplate shaker at 1000 rpm for 15 min (Thermoshaker NeoLab 7–0055, Bionovo, Legnica, Poland). The optical density of each well was then measured on a VICTOR Nivo multimode plate reader at a wavelength of 570 nm. Each experiment consisted of 6 replications of each concentration, and each experiment was repeated three times independently. The results were calculated as a percentage of control group viability. The IC_50_ values were determined using a non-linear regression from the plot of % viability against log dose of compounds by using GraphPad Prism 6.0 software (GraphPad, San Diego, CA, USA).

#### 3.3.2. Cell Migration Inhibition Assay

Transwell cell migration experiments were performed using xCELLigence RTCA Analyzer (Roche) in Cell Invasion and Migration (CIM) plates (ACEA Biosciences, San Diego, CA, USA). Each well consisted of an upper and a lower chamber separated by a microporous polyethylene terephthalate (PET) membrane containing randomly distributed 8 μm-pores. Prior to the migration assay, HepG2 cells (15 × 10^3^) were seeded onto 48-well plates containing the growth medium (EMEM) and incubated until 70–80% confluency and incubated at 37 °C in a humidified atmosphere containing 5% CO_2_. Subsequently, the cells were treated for 24 h with the tested compounds at the final concentration corresponding to the one-fourth of IC_50_ value. Next, 160 μL of complete growth medium (supplemented with 10% FBS) was added to the lower chamber and 50 μL of serum-free growth medium to the upper chambers of CIM plate. The plates were incubated at 37 °C and 5% CO_2_ saturation for 1 h prior to insertion into the xCELLigence platform. To initiate a transwell migration experiment, cells were detached, resuspended in the serum-free growth medium, and seeded in the upper chamber at a density of 1 × 10^4^ cells in 100 μL per well, previously treated with the compounds. The impedance of the microelectrode array was monitored and converted to the cell index (CI). Normalization values were recorded using the RTCA Software 1.2.1. Readouts were performed every 15 min for 72 h.

#### 3.3.3. Cell Cycle Analysis by Flow Cytometry

HepG2 (5 × 10^5^) cells were seeded onto 6-well cell culture plates and incubated for 24 h in a fully supplemented EMEM medium (Corning^®^) with addition of the analyzed compounds at a concentration corresponding to their total IC_50_ values. DNA content was determined by flow cytometry with PI (Sigma-Aldrich, Steinheim, Germany) staining. After incubation, the cells were trypsinized and washed twice with PBS (1 mL). In the next step, the cells were fixed with an ice-cold 80% ethanol. After 1 h incubation at 4 °C, the cells were stored at −20 °C for further analysis. After double wash with PBS, the fixed cells were stained with PI (50 μg/mL) with RNAse A (100 μg/mL) for 30 min at 37 °C in the dark. PI fluorescence was measured by FACSCalibur (Becton Dickinson), and data were analyzed by FlowJo software.

#### 3.3.4. Oxidative Stress Measurement in HepG2 Cells by Flow Cytometry

HepG2 cells (3.85 × 10^5^) were seeded onto 6-well plates, cultured with EMEM media at 37 °C and 5% CO_2_ saturation, and incubated until 60–70% confluence. Subsequently, the cells were treated for 24 h with the tested compounds at a concentration corresponding to their total IC_50_ values. Next, the cells were detached with trypsin (Corning^®^) and washed twice with DPBS (1 mL) (Thermo Fisher Scientific, Waltham, MA, USA). The level of intracellular ROS generation was analyzed by staining with CellROX™ Deep Red Reagent (ex/em: ~644/665 nm) according to the manufacturer’s protocol (Thermo Fisher Scientific, Waltham, MA, USA), in which fluorescence was triggered in the presence of ROS. The pelleted cells were suspended in DPBS (0.5 mL) containing CellROX™ Deep Red Reagent at the final concentration of 2.5 µM and then incubated at 37 °C for 30 min in the dark. The cells were analyzed after incubation with the dye, with excitation at 488 nm by the FACSCalibur flow cytometer (Becton Dickinson), and data were analyzed by FlowJo software.

#### 3.3.5. Analysis of 8-oxo-dG in HepG2 Cells

HepG2 cells (1 × 10^6^) were seeded onto flasks (25 cm^2^) and cultured for 16 h in the EMEM medium (Corning^®^) supplemented with 10% FBS (Corning^®^) and antibiotics (Sigma).Subsequently, the cells were treated for 24 h with the tested compounds at a concentration corresponding to their total IC_50_ values. Next, the cells were trypsinized, rinsed twice with PBS, and pelleted by centrifugation (200× *g*, 3 min). Total DNA was isolated from the treated cells and untreated control cells using a Genomic mini DNA isolation kit (A&A Biotechnology, Gdynia, Poland) according to the manufacturer′s protocol. The quality of the total DNA was assessed spectrophotometrically.

DNA (1 μg) was dissolved in sodium acetate buffer (10 μL, 40 mM, pH 5.3) containing ZnCl_2_ (0.1 mM) and digested with nuclease P1 (3 μg). Samples were incubated for 3 h at 37 °C. Subsequently, Tris–HCl (2 μL, 1 M, pH 8.0), alkaline phosphatase (1 U) were added and incubated for 2 h at 37 °C. All DNA hydrolysates were ultrafiltered using cut-off 10,000 Da filter units.

8-Oxo-dG and dG in hydrolysates were determined using HPLC-UV followed by electrochemical detection and ED (Coulochem III Electrochemical Detector, ESA, Inc., Chelmsford, MA, USA). DNA hydrolysates were chromatographed isocratically using ammonium acetate (50 mM pH 5.3)/methanol (93:7, *v*/*v*). Detection of dG was performed at 254 nm. Detection of 8-oxo-dG was performed by the electrochemical detector: guard cell + 400 mV, detector 1: +130 mV (as a screening electrode), detector 2: + 350 mV (as a measuring electrode set to sensitivity of 500 nA/V). All results are expressed as the mean ± SD.

To determine 8-oxo-dG levels, guanosine amount was necessary. The total number of 8-oxo-dG residues in the genome was calculated using the unique formula [54].

#### 3.3.6. Apoptosis/Necrosis Assay by Flow Cytometry

HepG2 cells (3.85 × 10^5^) were seeded onto 6-well plates, cultured in supplemented EMEM medium (Corning^®^) at 37 °C and 5% CO_2_ saturation, and incubated until 70–80% cell confluency was achieved. Subsequently, the cells were treated for 24 h, with the tested compounds **54**–**61** were added at a concentration corresponding to their total IC_50_ values. Next, the cells were detached with trypsin (Corning^®^) and washed twice with DPBS (1 mL) (Thermo Fisher Scientific, Waltham, MA, USA). In the meantime, annexin-binding buffer (10 mM HEPES, 140 mM NaCl, and 2.5 mM CaCl_2_, pH 7.4) was prepared. The cells were resuspended in annexin-binding buffer (100 μL per assay) and stained with Alexa Fluor™ 680 annexin V conjugate (ex/em: ~679/702 nm, Thermo Fisher Scientific, Waltham, MA, USA) according to the manufacturer’s protocol (5 μL of the annexin V conjugate was added to each 100 μL of cell suspension) and incubated for 15 min at RT, in the dark. After the incubation period, annexin-binding buffer (400 μL) was added, mixed gently, and the samples were kept on ice during the analysis. The cells were analyzed immediately after staining with 488 nm excitation by Accuri C6 flow cytometer (Becton Dickinson, Franklin Lake, NJ, USA) with excitation at 635 nm. The rate of apoptosis was evaluated immediately by FlowJo software.

#### 3.3.7. Fluorescence Imaging Experiment

HepG2 cells were seeded on a glass-bottom 4-well CELLview cell culture dish (Greiner Bio-One GmbH, Kremsmünster, Austria) at a density of 1.2 × 10^4^/well and cultured in supplemented EMEM medium, at 37 °C and 5% CO_2_ saturation, until 80–90% confluency. Then, cells were treated with the tested compounds at the final concentration corresponding to their total IC_50_ value for 24 h. In the next step, lysosomes were labeled with 100 nM of LysoTracker Red DND-99 (Thermo Fisher Scientific (Waltham, MA, USA)) for 15 min, and nuclei were stained with 3 µg/mL of Hoechst 33342 (Thermo Fisher Scientific (Waltham, MA, USA)) for 5 min. After incubation, the cells were gently rinsed in PBS to remove free dyes placed in the FluoroBrite DMEM (Thermo Fisher Scientific, Waltham, MA, USA). Live-cell imaging was performed using a Leica TCS SP5 II confocal laser scanning microscope equipped with a White Light Laser (470–670 nm), a 405 laser, and an environmental cell culture chamber that provided controlled conditions of temperature, CO_2_ saturation, and humidity. Fluorescence images were collected using a Plan Apo 63x 1.4 NA oil-immersion objective at Ex/Em 488/500–600 nm for autofluorescence of the tested compounds, 561/585–655 nm for lysosomes labeling, and 405/430–480 nm for nuclei staining. LAS AF (Leica, Wetzlar, Germany) and Leica LAS X software with a deconvolution module were used for image processing and fluorescence analysis, respectively.

#### 3.3.8. Human Topoisomerase IIα Relaxation Assay

The Human Topoisomerase II-alpha Relaxation Assay Kit was purchased from Inspiralis (Norwich, UK). The Topo IIα inhibition assay was performed as described by the manufacturer. Briefly, the reaction mixture (30 μL) containing the tested compound (200 µM) dissolved in DMSO (which had a final concentration of 2% that did not influence the Topo IIα activity), supercoiled pBR322 (0.5 μg) in 1X Assay Buffer and ATP (1 mM) was incubated at 37 °C for 15 min. Next, TopoIIα (1 U) was added, and the reaction mixture was incubated at 37 °C for an additional 45 min. The reaction was terminated by addition of the STEB buffer (40 % (*w*/*v*) sucrose, Tris-HCl (100 mM, pH 8), EDTA (1 mM), bromophenol blue (0.5 mg/mL)). The products were analyzed by electrophoresis using agarose gel (1%) in TAE buffer at 70 V for 2 h, followed by ethidium bromide staining. For the most active compounds, **6**–**8**, **15**, **22**, and **36**–**38**, the reaction was repeated at concentrations optimized to their activity, within the range of 0.1–100 µM, depending on the compound. The percentage of Topo-II alpha activity inhibition was calculated by densitometric quantification (Quantity One software, Bio-Rad). The occurrence of a band representing supercoiled DNA on an agarose gel indicated the inhibition of enzyme activity.

## 4. Conclusions

In conclusion, we designed, synthesized with a good yield, and characterized 1,8-naphthalic anhydride or 1,8-naphthalimide derivatives containing *ortho*- or *meta*-carborane clusters at position 4 of the heteroaromatic skeleton as analogs of mitonafide and pinafide. The X-ray structure of the naphthalic anhydride bearing *meta*-carborane **23** was established. The DNA-binding properties of the synthesized compounds were investigated by thermal denaturation experiments, CD, and UV–vis spectroscopy. Conjugates **54**–**61**, with an amine linker between the carborane group and the naphthalimide moiety, were found to interact strongly with ct-DNA, which indicates an intercalative binding mode. Furthermore, the same compounds were the most cytotoxic against HepG2 cells among all the modified conjugates tested. Our study also showed that they could effectively induce cell cycle arrest at G0/G1 (**54**–**59**) or G2M (**60** and **61**) phase, induce ROS production, mainly activate apoptosis, and target lysosomes in living cells. However, these compounds did not show inhibitory activity against Topo II. We selected compound **61** as the most promising among this group of compounds. It interacts most strongly with DNA, is most effective in inhibiting cell migration, and induces ROS production, resulting in a 4-fold higher number of 8-oxo-dG residues as compared to mitonafide. These properties could play an important role in anticancer activities through apoptosis induction.

The present work demonstrated that 4-carborane-1,8-naphthalimide derivatives can interact with DNA and inhibit cancer cell proliferation stronger than 3-carborane–1,8-naphthlimide conjugates. These new inorganic (carborane)–organic (1,8-naphthalimide) hybrids can be considered as a novel group of lead compounds with promising anticancer properties. Studies on the new carborane-naphthalimides are ongoing in our laboratory and will be published soon.

## Figures and Tables

**Table 1:** Δ*T*_m_ and binding constant (K*_b_*) based on UV–vis of ct-DNA in the presence of 1,8-naphthalimide–carborane conjugates. nd: no data.

Compound	Δ*T*_m_ [°C]	K*_b_* [M^−1^]
	ct-DNA	
**6**	−0.33	1.05 ± 0.43 × 10^5^
**7**	0.00	2.36 ± 0.81 × 10^5^
**8**	0.33	6.87 ± 0.30 × 10^4^
**9**	0.67	2.04 ± 0.86 × 10^5^
**10**	0.67	9.05 ± 4.44 × 10^4^
**11**	0.33	2.15 ± 1.02 × 10^5^
**14**	0.33	nd
**15**	0.00	nd
**16**	2.00	1.65 ± 0.80 × 10^5^
**17**	0.67	1.66 ± 1.00 × 10^5^
**18**	0.37	1.36 ± 0.31 × 10^5^
**19**	0.67	1.50 ± 0.48 × 10^5^
**22**	−1.00	nd
**23**	−0.33	nd
**24**	1.33	nd
**25**	0.33	nd
**26**	1.33	nd
**27**	0.33	nd
**36**	1.33	4.00 ± 1.22 × 10^5^
**37**	2.00	2.48 ± 0.34 × 10^5^
**38**	1.00	2.80 ± 1.24 × 10^5^
**39**	1.00	3.84 ± 0.87 × 10^5^
**40**	0.67	9.45 ± 4.13 × 10^4^
**41**	−1.33	8.46 ± 3.65 × 10^4^
**42**	0.67	2.34 ± 1.48 × 10^5^
**43**	1.00	1.66 ± 0.62 × 10^5^
**54**	7.67	3.24 ± 0.56 × 10^5^
**55**	9.67	3.61 ± 1.23 × 10^5^
**56**	7.67	2.86 ± 1.42 × 10^5^
**57**	11.00	3.67 ± 1.20 × 10^5^
**58**	11.00	2.99 ± 0.48 × 10^5^
**59**	8.33	2.03 ± 0.13 × 10^5^
**60**	7.67	3.15 ± 1.02 × 10^5^
**61**	12.33	3.52 ± 1.13 × 10^5^

**Table 2:** Cellular cytotoxic activity of compounds **6**–**11**, **14**–**19**, **22**–**27**, **36**–**42**, and **54**–**61**.

Compound	IC_50_ ^a^ [µM]
**6**	71.19 ± 1.78
**7**	136.00 ± 4.24
**8**	9.65 ± 1.02
**9**	29.55 ± 1.89
**10**	5.35 ± 1.90
**11**	14.58 ± 0.43
**14**	34.13 ± 2.33
**15**	38.12 ± 2.85
**16**	8.17 ± 3.72
**17**	36.85 ± 3.72
**18**	5.80 ± 0.23
**19**	15.02 ± 0.57
**22**	28.65 ± 3.84
**23**	108.71 ± 3.78
**24**	10.99 ± 0.66
**25**	15.22 ± 2.02
**26**	4.89 ± 2.59
**27**	4.07 ± 1.19
**36**	6.03 ± 0.91
**37**	10.50 ± 0.53
**38**	10.11 ± 1.85
**39**	9.85 ± 0.59
**40**	12.24 ± 0.44
**41**	11.90 ± 0.92
**42**	21.09 ± 3.34
**43**	40.01 ± 3.89
**54**	5.49 ± 0.38
**55**	3.43 ± 0.28
**56**	12.07 ± 1.03
**57**	3.12 ± 0.04
**58**	3.87 ± 1.03
**59**	5.35 ± 1.36
**60**	30.87 ± 2.66
**61**	4.79 ± 0.06

^a^ compound concentration required to inhibit cell growth by 50%; mitonafide IC_50_ < 1 [8]; pinafide IC_50_ = 1.23 ± 0.15 [8].

**Table 3:** 8-Oxo-dG level in cellular DNA after treatment with compounds **54**–**61**.

Compound	[8-oxo-dG ± SD] × 10^6^
Control	2.88 ± 0.17
**54**	43.17 ± 0.75
**55**	74.85 ± 1.38
**56**	52.31 ± 0.51
**57**	57.54 ± 0.73
**58**	96.58 ± 0.56
**59**	81.64 ± 0.67
**60**	164.35 ± 6.39
**61**	123.92 ± 2.59

Mitonafide—28.24 ± 0.18 per 10^6^ dG [8].

**Table 4:** Inhibitory properties of the tested 1,8-naphthalimide–carborane conjugates and mitonafide for human DNA topoisomerase IIα.

Compound	IC_50_ ^a^ (µM)
**6**	7.73
**7**	8.78
**8**	0.58
**15**	61.59
**36**	7.72
**37**	7.49
**38**	2.81
Mitonafide	5.13

^a^ compound concentration (µM) required to inhibit the activity of this enzyme by 50%.

## Data Availability

Not applicable.

## Supplementary Materials

The following are available online at https://www.mdpi.com/article/10.3390/ijms23094598/s1: ^1^H, ^13^C, ^11^B{H BB} NMR, FTIR, RP-HPLC, and MS spectra of **3**, **6**–**11**, **13**–**19**, **22**–**27**, **36**–**43**, and **54**–**61**; figures connected with biological and physicochemical studies.
