# Supplementary material for: Design of DNA Intercalators Based on 4-Carboranyl-1,8-Naphthalimides: Investigation of Their DNA-Binding Ability and Anticancer Activity
Source: Int J Mol Sci. 2022 Apr 21;23(9):4598. doi: 10.3390/ijms23094598 (PMC9101373; doi:10.3390/ijms23094598)
Supplement: Supplementary file 1 [file ijms-23-04598-s001.zip › ijms-1674820-supplementary.pdf]

Design of DNA Intercalators Based on 4-Carboranyl-1,8-Naphthalimides: Investigation of Their DNA-Binding Ability and Anticancer Activity

Sebastian Rykowski [1], Dorota Gurda-Woźna [2], Marta Orlicka-Płocka [2], Agnieszka Fedoruk-Wyszomirska [2], Małgorzata Giel-Pietraszuk [2], Eliza Wyszko [2], Aleksandra Kowalczyk [3], Paweł Stączek [3], Katarzyna Biniek-Antosiak [2], Wojciech Rypniewski [2] and Agnieszka B. Olejniczak [1,*]

[1] Institute of Medical Biology, Polish Academy of Sciences, 106 Lodowa St., 93-232 Lodz, Poland; srykowski@cbm.pan.pl
[2] Institute of Bioorganic Chemistry, Polish Academy of Sciences, 12/14 Z. Noskowskiego St., 61-704 Poznan, Poland; d_gurda@ibch.poznan.pl (D.G.-W.); mplocka@ibch.poznan.pl (M.O.-P.); agaw@ibch.poznan.pl (A.F.-W.); giel@ibch.poznan.pl (M.G.-P.); wyszkoe@ibch.poznan.pl (E.W.); kbiniek@ibch.poznan.pl (K.B.-A.); wojtekr@ibch.poznan.pl (W.R.)
[3] Department of Molecular Microbiology, Faculty of Biology and Environmental Protection, University of Lodz, 12/16 Banacha St., 90-237 Lodz, Poland; aleksandra.strzelczyk@biol.uni.lodz.pl (A.K.); pawel.staczek@biol.uni.lodz.pl (P.S.)
* Correspondence: aolejniczak@cbm.pan.pl; Tel.: +48-42-272-36-37

Table of contents

Figure S1. ^{1}H NMR spectrum of **3**.
Figure S2. ^{1}H NMR spectrum of **13**.
Figure S3. ^{1}H NMR spectrum of **6**.
Figure S4. ^{13}C NMR spectrum of **6**.
Figure S5. ^{11}B NMR spectrum of **6**.
Figure S6. UV spectrum of **6**.
Figure S7. IR spectrum of **6**.
Figure S8. HPLC analysis of **6**.
Figure S9. MS spectrum of **6**.
Figure S10. ^{1}H NMR spectrum of **7**.
Figure S11. ^{13}C NMR spectrum of **7**.
Figure S12. ^{11}B NMR spectrum of **7**.
Figure S13. UV spectrum of **7**.
Figure S14. IR spectrum of **7**.
Figure S15. HPLC analysis of **7**.
Figure S16. MS spectrum of **7**.
Figure S17. ^{1}H NMR spectrum of **8**.
Figure S18. ^{13}C NMR spectrum of **8**.
Figure S19. ^{11}B NMR spectrum of **8**.
Figure S20. UV spectrum of **8**.
Figure S21. IR spectrum of **8**.
Figure S22. HPLC analysis of **8**.
Figure S23. MS spectrum of **8**.
Figure S24. ^{1}H NMR spectrum of **9**.
Figure S25. ^{13}C NMR spectrum of **9**.
Figure S26. ^{11}B NMR spectrum of **9**.
Figure S27. UV spectrum of **9**.
Figure S28. IR spectrum of **9**.
Figure S29. HPLC analysis of **9**.
Figure S30. MS spectrum of **9**.
Figure S31. ^{1}H NMR spectrum of **10**.
Figure S32. ^{13}C NMR spectrum of **10**.
Figure S33. ^{11}B NMR spectrum of **10**.
Figure S34. UV spectrum of **10**.
Figure S35. IR spectrum of **10**.
Figure S36. HPLC analysis of **10**.
Figure S37. MS spectrum of **10**.
Figure S38. ^{1}H NMR spectrum of **11**.
Figure S39. ^{13}C NMR spectrum of **11**.
Figure S40. ^{11}B NMR spectrum of **11**.
Figure S41. UV spectrum of **11**.
Figure S42. IR spectrum of **11**.
Figure S43. HPLC analysis of **11**.
Figure S44. MS spectrum of **11**.
Figure S45. ^{1}H NMR spectrum of **14**.
Figure S46. ^{13}C NMR spectrum of **14**.
Figure S47. ^{11}B NMR spectrum of **14**.

Figure S48. UV spectrum of **14**.
Figure S49. IR spectrum of **14**.
Figure S50. HPLC analysis of **14**.
Figure S51. MS spectrum of **14**.
Figure S52. ^{1}H NMR spectrum of **15**.
Figure S53. ^{13}C NMR spectrum of **15**.
Figure S54. ^{11}B NMR spectrum of **15**.
Figure S55. UV spectrum of **15**.
Figure S56. IR spectrum of **15**.
Figure S57. HPLC analysis of **15**.
Figure S58. MS spectrum of **15**.
Figure S59. ^{1}H NMR spectrum of **16**.
Figure S60. ^{13}C NMR spectrum of **16**.
Figure S61. ^{11}B NMR spectrum of **16**.
Figure S62. UV spectrum of **16**.
Figure S63. IR spectrum of **16**.
Figure S64. HPLC chromatogram of 1**6**.
Figure S65. MS spectrum of **16**.
Figure S66. ^{1}H NMR spectrum of **17**.
Figure S67. ^{13}C NMR spectrum of **17**.
Figure S68. ^{11}B NMR spectrum of **17**.
Figure S69. UV spectrum of **17**.
Figure S70. IR spectrum of **17**.
Figure S71. HPLC chromatogram of **17**.
Figure S72. MS spectrum of **17**.
Figure S73. ^{1}H NMR spectrum of **18**.
Figure S74. ^{13}C NMR spectrum of **18**.
Figure S75. ^{11}B NMR spectrum of **18**.
Figure S76. UV spectrum of **18**.
Figure S77. IR spectrum of **18**.
Figure S78. HPLC chromatogram of **18**.
Figure S79. MS spectrum of **18**.
Figure S80. ^{1}H NMR spectrum of **19**.
Figure S81. ^{13}C NMR spectrum of **19**.
Figure S82. ^{11}B NMR spectrum of **19**.
Figure S83. UV spectrum of **19**.
Figure S84. IR spectrum of **19**.
Figure S85. HPLC chromatogram of **19**.
Figure S86. MS spectrum of **19**.
Figure S87. ^{1}H NMR spectrum of **22**.
Figure S88. ^{13}C NMR spectrum of **22**.
Figure S89. ^{11}B NMR spectrum of **22**.
Figure S90. UV spectrum of **22**.
Figure S91. IR spectrum of **22**.
Figure S92. HPLC chromatogram of **22**.
Figure S93. MS spectrum of **22**.
Figure S94. ^{1}H NMR spectrum of **23**.
Figure S95. ^{13}C NMR spectrum of **23**.
Figure S96. ^{11}B NMR spectrum of **23**.
Figure S97. UV spectrum of **23.**

Figure S98. IR spectrum of **23**.
Figure S99. HPLC chromatogram of **23**.
Figure S100. MS spectrum of **23**.
Figure S101. ^{1}H NMR spectrum of **24**.
Figure S102. ^{13}C NMR spectrum of **24**.
Figure S103. ^{11}B NMR spectrum of **24**.
Figure S104. UV spectrum of **24**.
Figure S105. IR spectrum of **24**.
Figure S106. HPLC chromatogram of **24**.
Figure S107. MS spectrum of **24**.
Figure S108. ^{1}H NMR spectrum of **25**.
Figure S109. ^{13}C NMR spectrum of **25**.
Figure S110. ^{11}B NMR spectrum of **25**.
Figure S111. UV spectrum of **25**.
Figure S112. IR spectrum of **25**.
Figure S113. HPLC chromatogram of **25**.
Figure S114. MS spectrum of **25**.
Figure S115. ^{1}H NMR spectrum of **26**.
Figure S116. ^{13}C NMR spectrum of **26**.
Figure S117. ^{11}B NMR spectrum of **26**.
Figure S118. UV spectrum of **26**.
Figure S119. IR spectrum of **26**.
Figure S120. HPLC chromatogram of **26**.
Figure S121. MS spectrum of **26**.
Figure S122. ^{1}H NMR spectrum of **27**.
Figure S123. ^{13}C NMR spectrum of **27**.
Figure S124. ^{11}B NMR spectrum of **27**.
Figure S125. UV spectrum of **27**.
Figure S126. IR spectrum of **27**.
Figure S127. HPLC chromatogram of **27**.
Figure S128. MS spectrum of **27**.
Figure S129. ^{1}H NMR spectrum of **36**.
Figure S130. ^{13}C NMR spectrum of **36**.
Figure S131. ^{11}B NMR spectrum of **36**.
Figure S132. UV spectrum of **36**.
Figure S133. IR spectrum of **36**.
Figure S134. HPLC chromatogram of **36**.
Figure S135. MS spectrum of **36**.
Figure S136. ^{1}H NMR spectrum of **37**.
Figure S137. ^{13}C NMR spectrum of **37**.
Figure S138. ^{11}B NMR spectrum of **37**.
Figure S139. UV spectrum of **37.**
Figure S140. IR spectrum of **37**.
Figure S141. HPLC chromatogram of **37**.
Figure S142. MS spectrum of **37**.
Figure S143. ^{1}H NMR spectrum of **38**.
Figure S144. ^{13}C NMR spectrum of **38**.
Figure S145. ^{11}B NMR spectrum of **38**.
Figure S146. UV spectrum of **38**.
Figure S147. IR spectrum of **38**.

Figure S148. HPLC chromatogram of **38**.
Figure S149. MS spectrum of **38**.
Figure S150. ^{1}H NMR spectrum of **39**.
Figure S151. ^{13}C NMR spectrum of **39**.
Figure S152. ^{11}B NMR spectrum of **39**.
Figure S153. UV spectrum of **39**.
Figure S154. IR spectrum of **39**.
Figure S155. HPLC chromatogram of **39**.
Figure S156. MS spectrum of **39**.
Figure S157. ^{1}H NMR spectrum of **40**.
Figure S158. ^{13}C NMR spectrum of **40**.
Figure S159. ^{11}B NMR spectrum of **40**.
Figure S160. UV spectrum of **40**.
Figure S161. IR spectrum of **40**.
Figure S162. HPLC chromatogram of **40**.
Figure S163. MS spectrum of **40**.
Figure S164. ^{1}H NMR spectrum of **41**.
Figure S165. ^{13}C NMR spectrum of **41**.
Figure S166. ^{11}B NMR spectrum of **41**.
Figure S167. UV spectrum of **41**.
Figure S168. IR spectrum of **41**.
Figure S169. HPLC chromatogram of **41**.
Figure S170. MS spectrum of **41**.
Figure S171. ^{1}H NMR spectrum of **42**.
Figure S172. ^{13}C NMR spectrum of **42**.
Figure S173. ^{11}B NMR spectrum of **42**.
Figure S174. UV spectrum of **42**.
Figure S175. IR spectrum of **42**.
Figure S176. HPLC chromatogram of **42**.
Figure S177. MS spectrum of **42**.
Figure S178. ^{1}H NMR spectrum of **43**.
Figure S179. ^{13}C NMR spectrum of **43**.
Figure S180. ^{11}B NMR spectrum of **43**.
Figure S181. UV spectrum of **43**.
Figure S182. IR spectrum of **43**.
Figure S183. HPLC chromatogram of **43**.
Figure S184. MS spectrum of **43**.
Figure S185. ^{1}H NMR spectrum of **54**.
Figure S186. ^{13}C NMR spectrum of **54**.
Figure S187. ^{11}B NMR spectrum of **54**.
Figure S188. UV spectrum of **54**.
Figure S189. IR spectrum of **54**.
Figure S190. HPLC chromatogram of **54**.
Figure S191. MS spectrum of **54**.
Figure S192. HRMS spectrum of **54**.
Figure S193. ^{1}H NMR spectrum of **55**.
Figure S194. ^{13}C NMR spectrum of **55**.
Figure S195. ^{11}B NMR spectrum of **55**.
Figure S196. UV spectrum of **55**.
Figure S197. IR spectrum of **55**.

Figure S198. HPLC chromatogram of **55**.
Figure S199. MS spectrum of **55**.
Figure S200. HRMS spectrum of **55**.
Figure S201. ^{1}H NMR spectrum of **56**.
Figure S202. ^{13}C NMR spectrum of **56**.
Figure S203. ^{11}B NMR spectrum of **56**.
Figure S204. UV spectrum of **56**.
Figure S205. IR spectrum of **56**.
Figure S206. HPLC chromatogram of **56**.
Figure S207. MS spectrum of **56**.
Figure S208. HRMS spectrum of **56**.
Figure S209. ^{1}H NMR spectrum of **57**.
Figure S210. ^{13}C NMR spectrum of **57**.
Figure S211. ^{11}B NMR spectrum of **57**.
Figure S212. UV spectrum of **57**.
Figure S213. IR spectrum of **57**.
Figure S214. HPLC chromatogram of **57**.
Figure S215. MS spectrum of **57**.
Figure S216. HRMS spectrum of **57**.
Figure S217. ^{1}H NMR spectrum of **58**.
Figure S218. ^{13}C NMR spectrum of **58**.
Figure S219. ^{11}B NMR spectrum of **58**.
Figure S220. UV spectrum of **58**.
Figure S221. IR spectrum of **58**.
Figure S222. HPLC chromatogram of **58**.
Figure S223. MS spectrum of **58**.
Figure S224. HRMS spectrum of **58**.
Figure S225. ^{1}H NMR spectrum of **59**.
Figure S226. ^{13}C NMR spectrum of **59**.
Figure S227. ^{11}B NMR spectrum of **59**.
Figure S228. UV spectrum of **59**.
Figure S229. IR spectrum of **59**.
Figure S230. HPLC chromatogram of **59**.
Figure S231. MS spectrum of **59**.
Figure S232. HRMS spectrum of **59**.
Figure S233. ^{1}H NMR spectrum of **60**.
Figure S234. ^{13}C NMR spectrum of **60**.
Figure S235. ^{11}B NMR spectrum of **60**.
Figure S236. UV spectrum of **60**.
Figure S237. IR spectrum of **60**.
Figure S238. HPLC chromatogram of **60**.
Figure S239. MS spectrum of **60**.
Figure S240. HRMS spectrum of **60**.
Figure S241. ^{1}H NMR spectrum of **61**.
Figure S242. ^{13}C NMR spectrum of **61**.
Figure S243. ^{11}B NMR spectrum of **61**.
Figure S244. UV spectrum of **61**.
Figure S245. IR spectrum of **61**.
Figure S246. HPLC chromatogram of **61**.
Figure S247. MS spectrum of **61**.

Figure S248. HRMS spectrum of **61**.
Figure S249. Melting curves of ct-DNA upon addition of **6-11**.
Figure S250. Melting curves of ct-DNA upon addition of **14-19**.
Figure S251. Melting curves of ct-DNA upon addition of **22-27**.
Figure S252. Melting curves of ct-DNA upon addition of **36-43**.
Figure S253. Melting curves of ct-DNA upon addition of **54-61**.
Figure S254. Changes in the CD spectrum of ct-DNA upon addition of **6** and **7**.
Figure S255. Changes in the CD spectrum of ct-DNA upon addition of **8** and **9**.
Figure S256. Changes in the CD spectrum of ct-DNA upon addition of **10** and **11**.
Figure S257. Changes in the CD spectrum of ct-DNA upon addition of **14** and **15**.
Figure S258. Changes in the CD spectrum of ct-DNA upon addition of **16** and **17**.
Figure S259. Changes in the CD spectrum of ct-DNA upon addition of **18** and **19**.
Figure S260. Changes in the CD spectrum of ct-DNA upon addition of **22** and **23**.
Figure S261. Changes in the CD spectrum of ct-DNA upon addition of **24** and **25**.
Figure S262. Changes in the CD spectrum of ct-DNA upon addition of **26** and **27**.
Figure S263. Changes in the CD spectrum of ct-DNA upon addition of **36** and **37**.
Figure S264. Changes in the CD spectrum of ct-DNA upon addition of **38** and **39**.
Figure S265. Changes in the CD spectrum of ct-DNA upon addition of **40** and **41**.
Figure S266. Changes in the CD spectrum of ct-DNA upon addition of **42** and **43**.
Figure S267. Changes in the CD spectrum of ct-DNA upon addition of **54** and **55**.
Figure S268. Changes in the CD spectrum of ct-DNA upon addition of **56** and **57**.
Figure S269. Changes in the CD spectrum of ct-DNA upon addition of **58** and **59**.
Figure S270. Changes in the CD spectrum of ct-DNA upon addition of **60** and **61**.
Figure S271. UV-vis absorption spectra of compound **6**.
Figure S272. UV-vis absorption spectra of compound **7**.
Figure S273. UV-vis absorption spectra of compound **8**.
Figure S274. UV-vis absorption spectra of compound **9**.
Figure S275. UV-vis absorption spectra of compound **10**.
Figure S276. UV-vis absorption spectra of compound **11**.
Figure S277. UV-vis absorption spectra of compound **16**.
Figure S278. UV-vis absorption spectra of compound **17**.
Figure S279. UV-vis absorption spectra of compound **18**.
Figure S280. UV-vis absorption spectra of compound **19**.
Figure S281. UV-vis absorption spectra of compound **36**.
Figure S282. UV-vis absorption spectra of compound **37**.
Figure S283. UV-vis absorption spectra of compound **38**.
Figure S284. UV-vis absorption spectra of compound **39**.
Figure S285. UV-vis absorption spectra of compound **40**.
Figure S286. UV-vis absorption spectra of compound **41**.
Figure S287. UV-vis absorption spectra of compound **42**.
Figure S288. UV-vis absorption spectra of compound **43**.
Figure S289. UV-vis absorption spectra of compound **54**.
Figure S290. UV-vis absorption spectra of compound **55**.
Figure S291. UV-vis absorption spectra of compound **56**.
Figure S292. UV-vis absorption spectra of compound **57**.
Figure S293. UV-vis absorption spectra of compound **58**.
Figure S294. UV-vis absorption spectra of compound **59**.
Figure S295. UV-vis absorption spectra of compound **60**.
Figure S296. UV-vis absorption spectra of compound **61**.

Figure S297. Influence of compound **54**, **55**, **56**, **57**, **58**, **59**, **60**, and **61** on cell cycle distribution in HepG2 cells.
Figure S298. ROS production in HepG2 cells after 24 h incubation with compound **54**, **55**, **56**, **57**, **58**, **59**, **60**, and **61**.
Figure S299. Flow cytometry analysis of apoptosis/necrosis in HepG2 cells after cell toxicity induction with compound **54**, **55**, **56**, **57**, **58**, **59**, **60**, and **61**.
Figure S300. Human Topoisomerase IIα relaxation assay in the presence of modified with carborane cluster naphthalic anhydrides naphthalic anhydrides (**6**, **7**, **14**, **15**, **22**, **23**) and 1,8-naphthalimides (**8-11**, **16-19**, **24-27**, **36-43**, **54-61**).
Figure S301. Inhibition of the relaxation activity of human topoisomerase IIα in the presence of compounds **6**, **8**, **36**, **38**, and mitonafide.
Table S1. Crystallographic data.

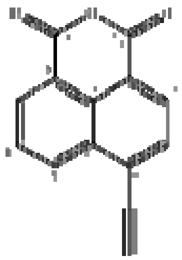

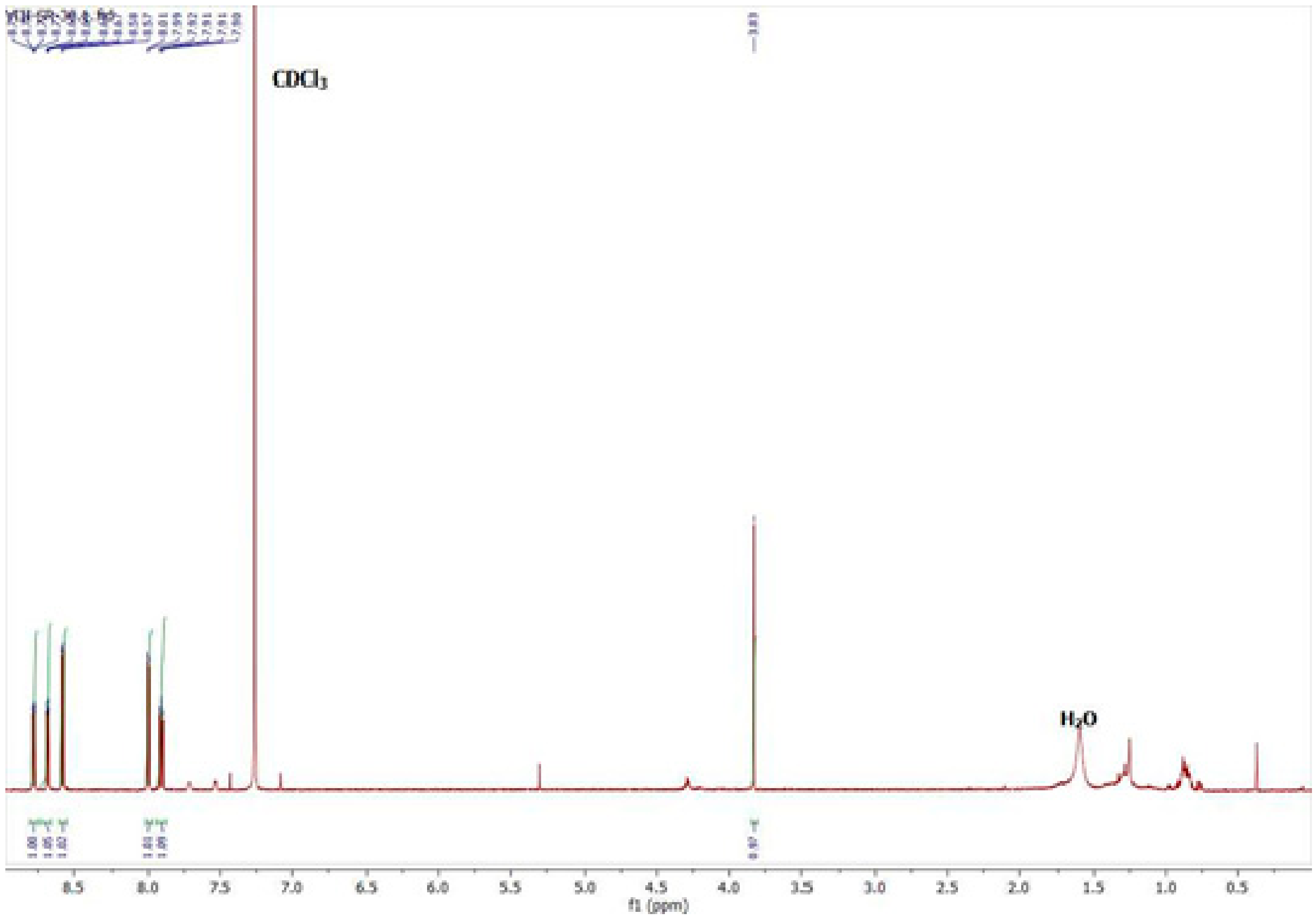

Figure S1. ^{1}H NMR spectrum of **3**.

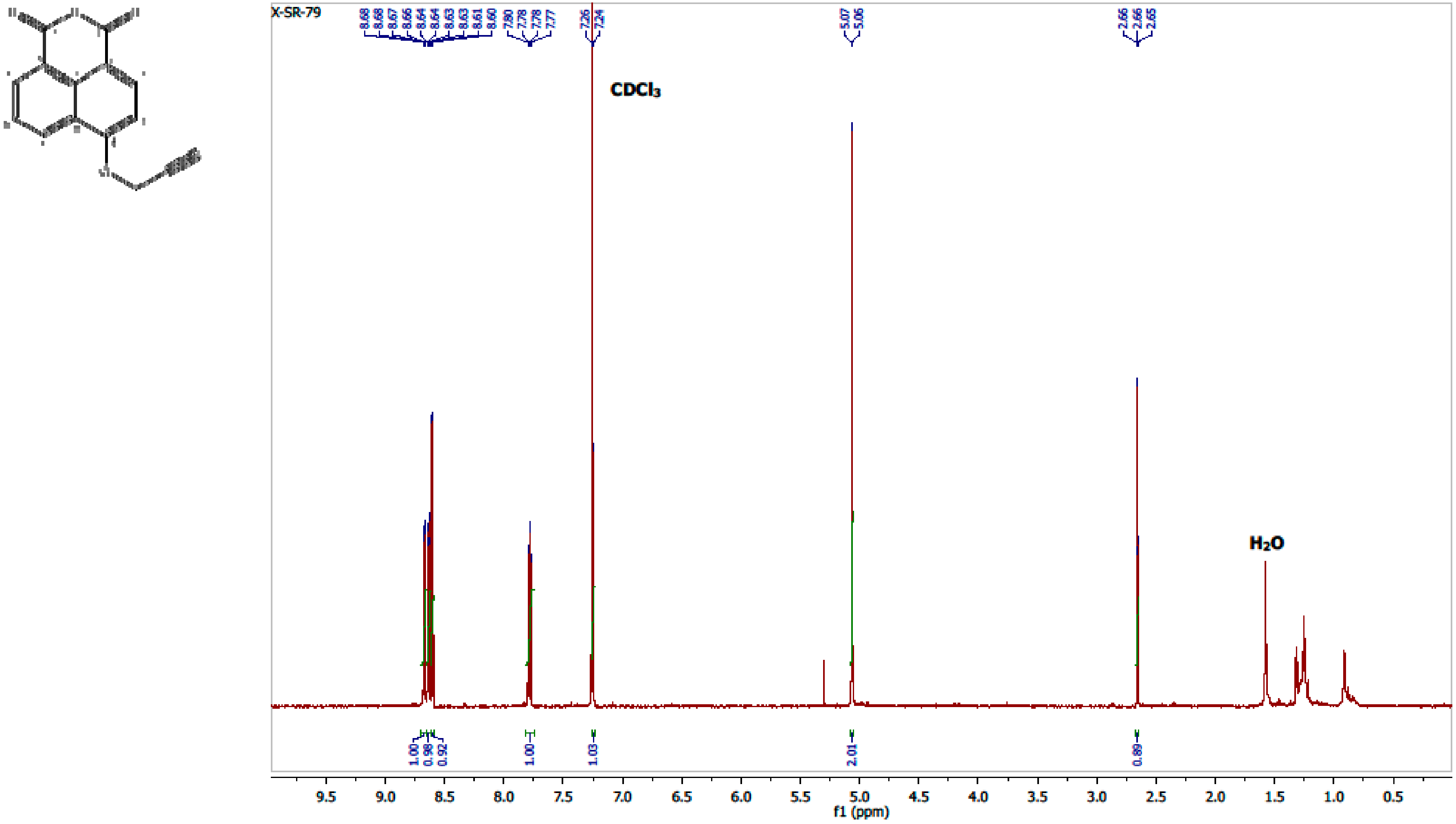

Figure S2. ^{1}H NMR spectrum of **13**.

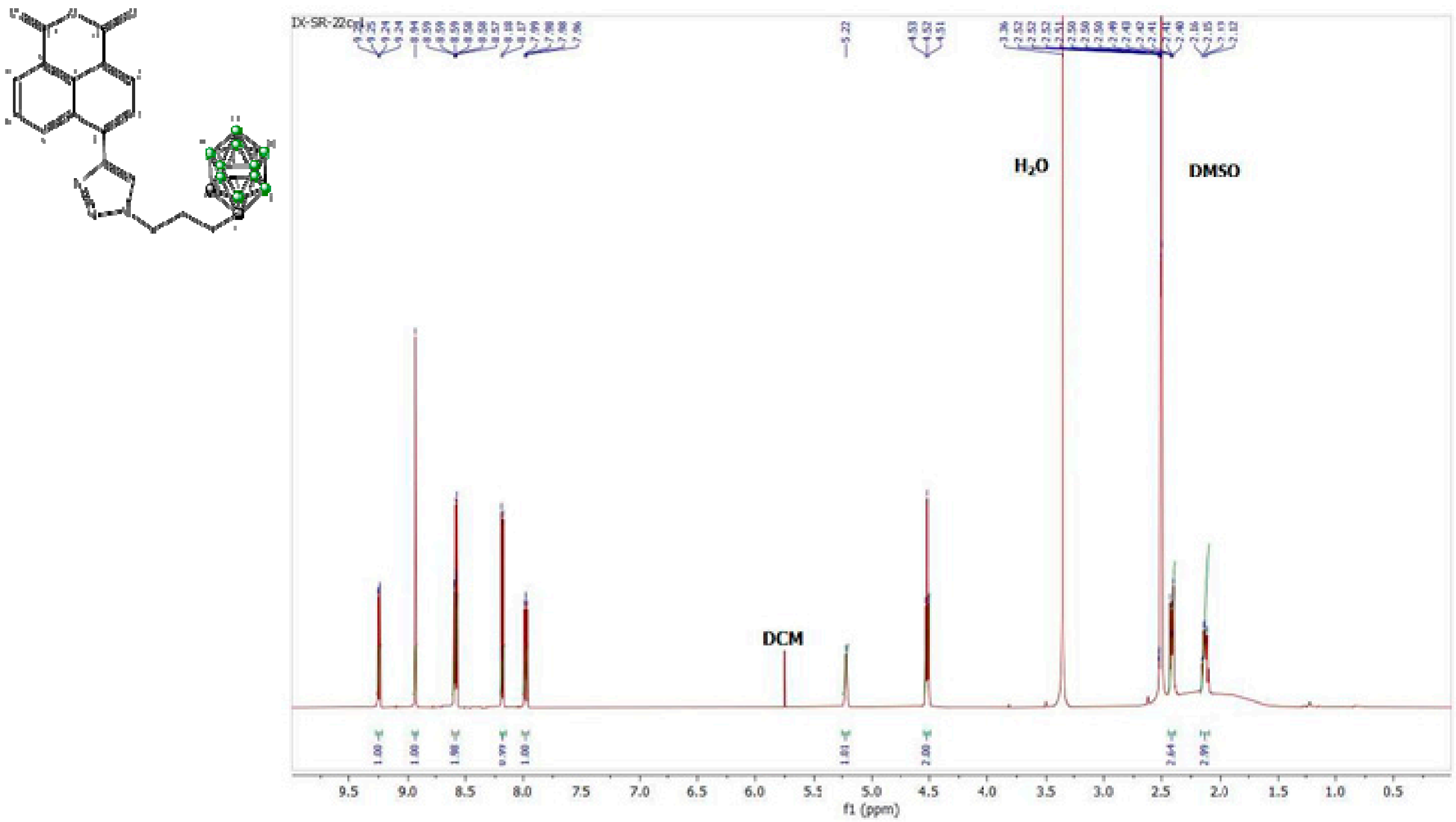

Figure S3. ^{1}H NMR spectrum of **6**.

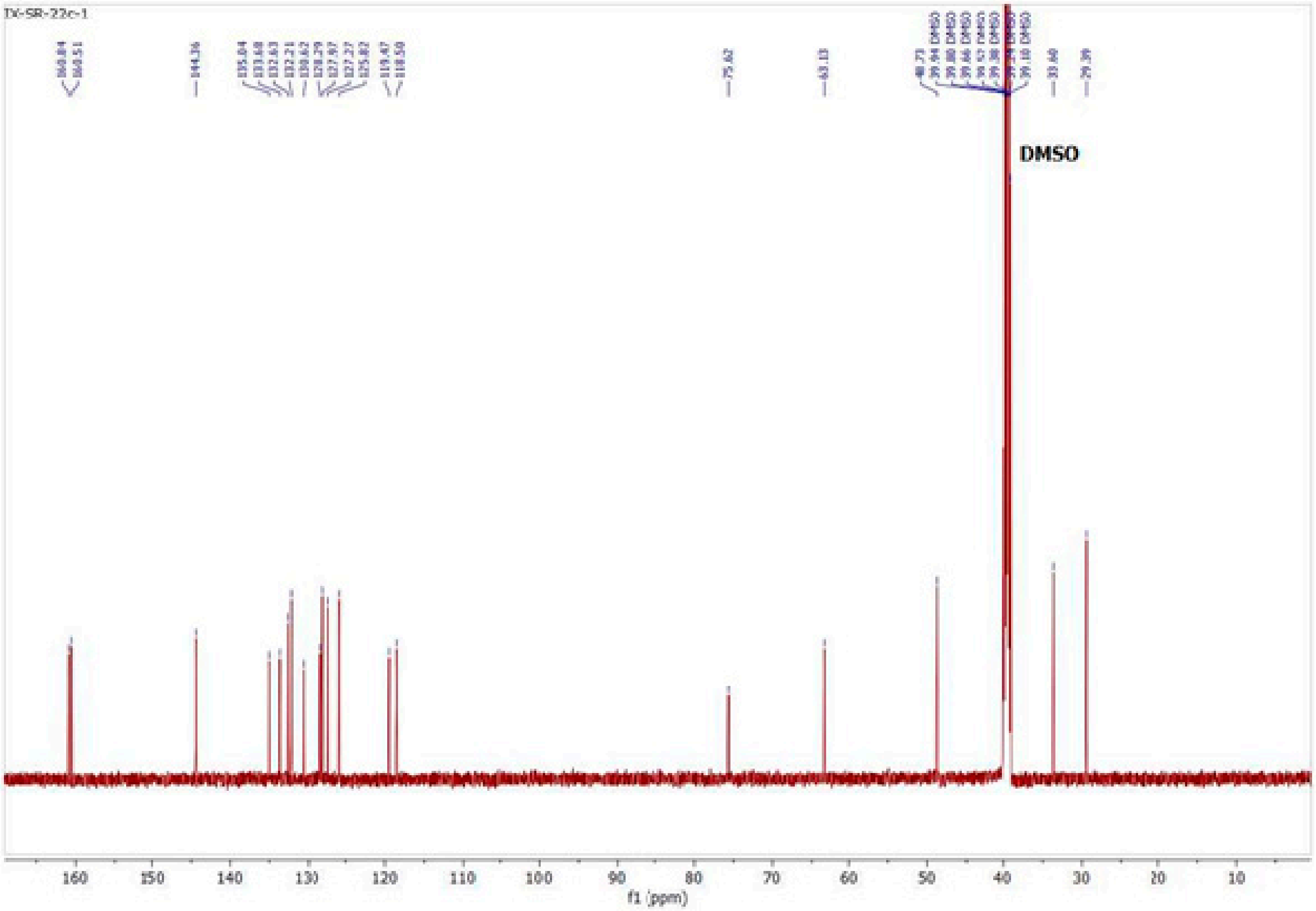

Figure S4. ^{13}C NMR spectrum of **6**.

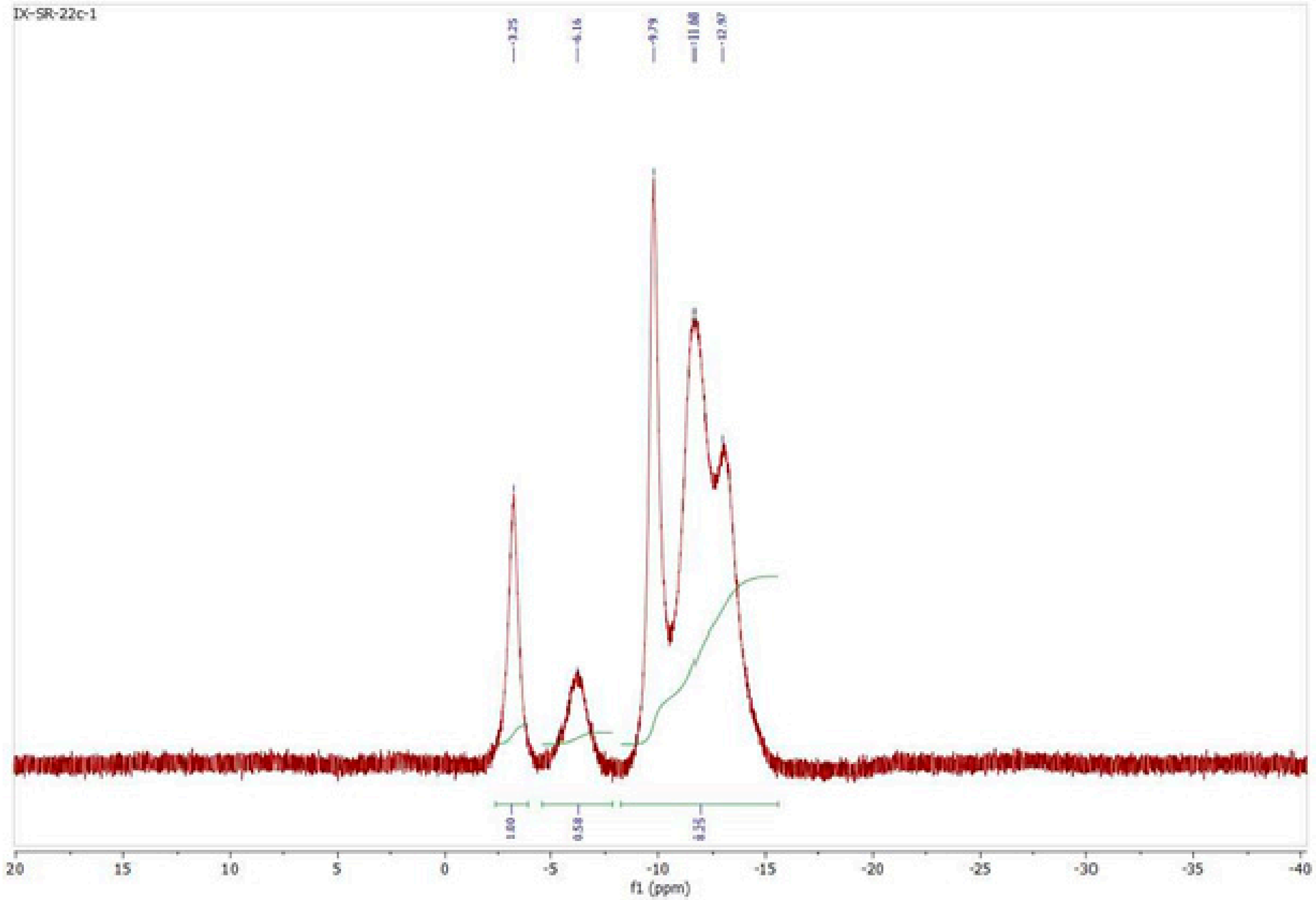

Figure S5. ^{11}B NMR spectrum of **6**.

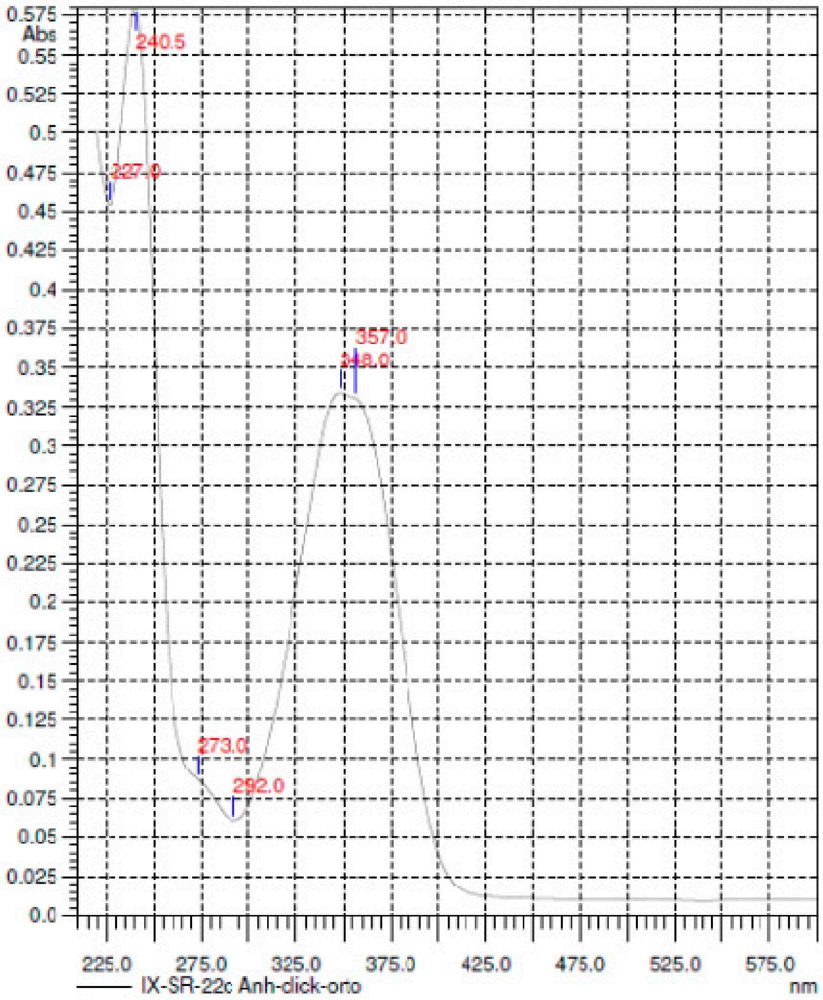

Figure S6. UV spectrum of **6**.

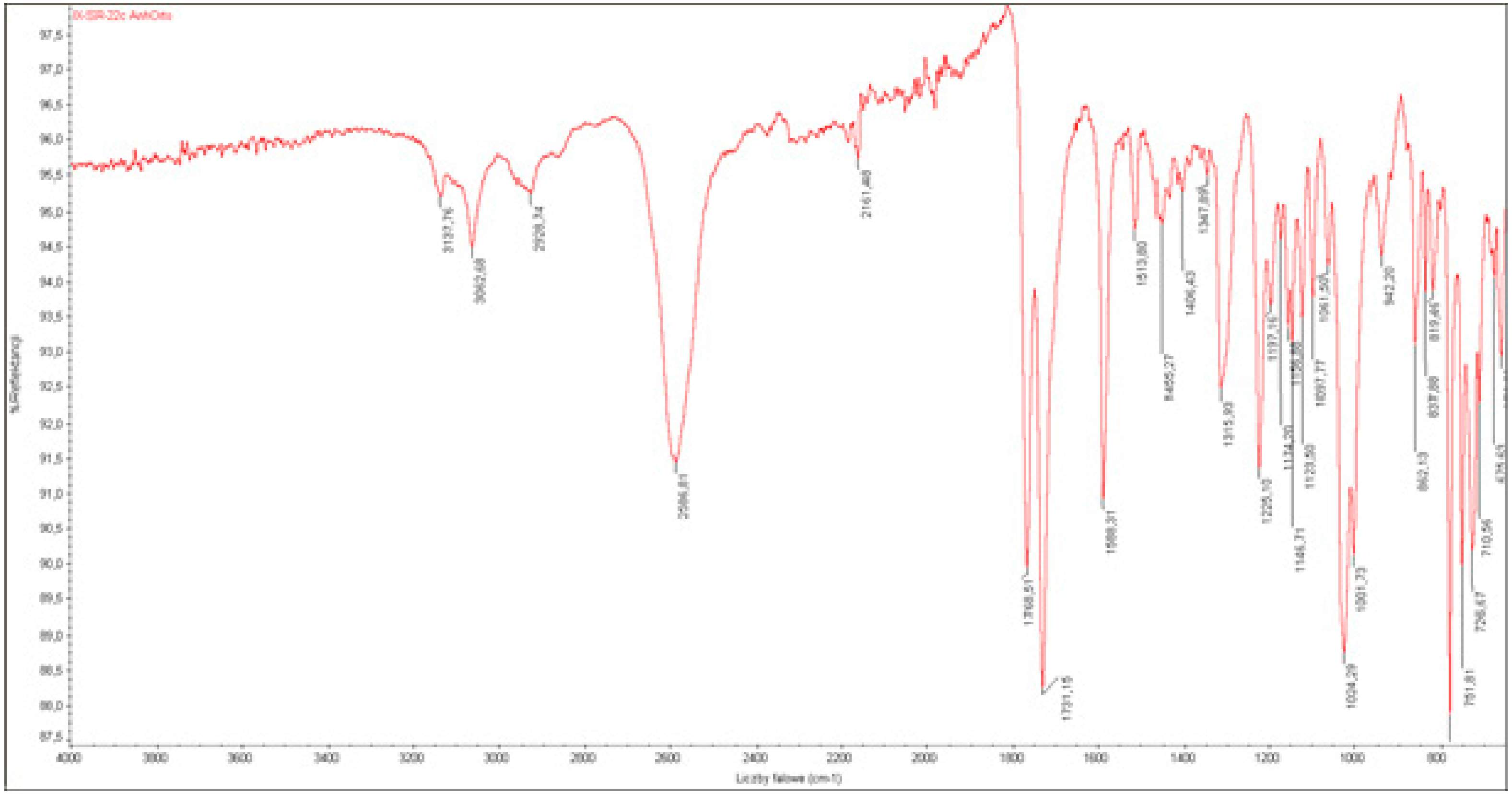

Figure S7. IR spectrum of **6**.

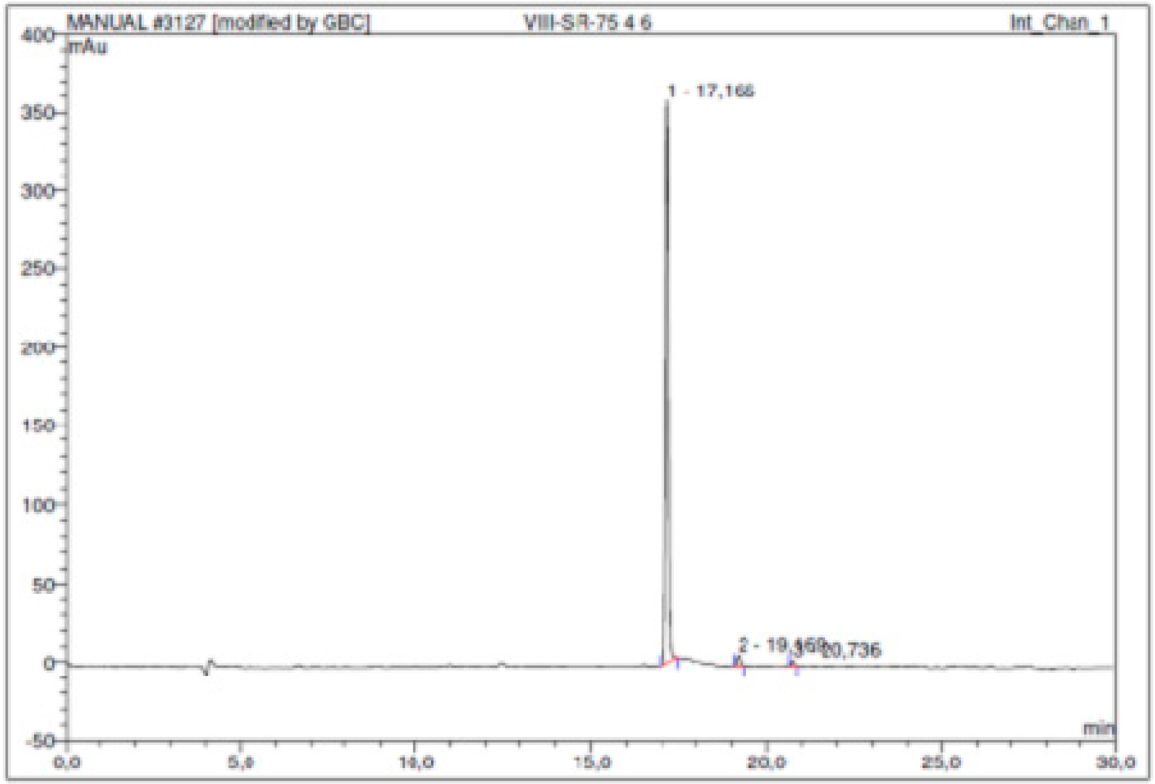

No.	Ret.Time min	Peak Name	Height mAu	Area mAu*min	Rel.Area %	Amount	Type
1	17,17	n.a.	357,675	35,707	97,39	n.a.	BMB*
2	19,17	n.a.	6,579	0,664	1,81	n.a.	BMB*
3	20,74	n.a.	3,304	0,292	0,80	n.a.	BMB*
Total:			367,559	36,663	100,00	0,000	

Figure S8. HPLC analysis of **6**.

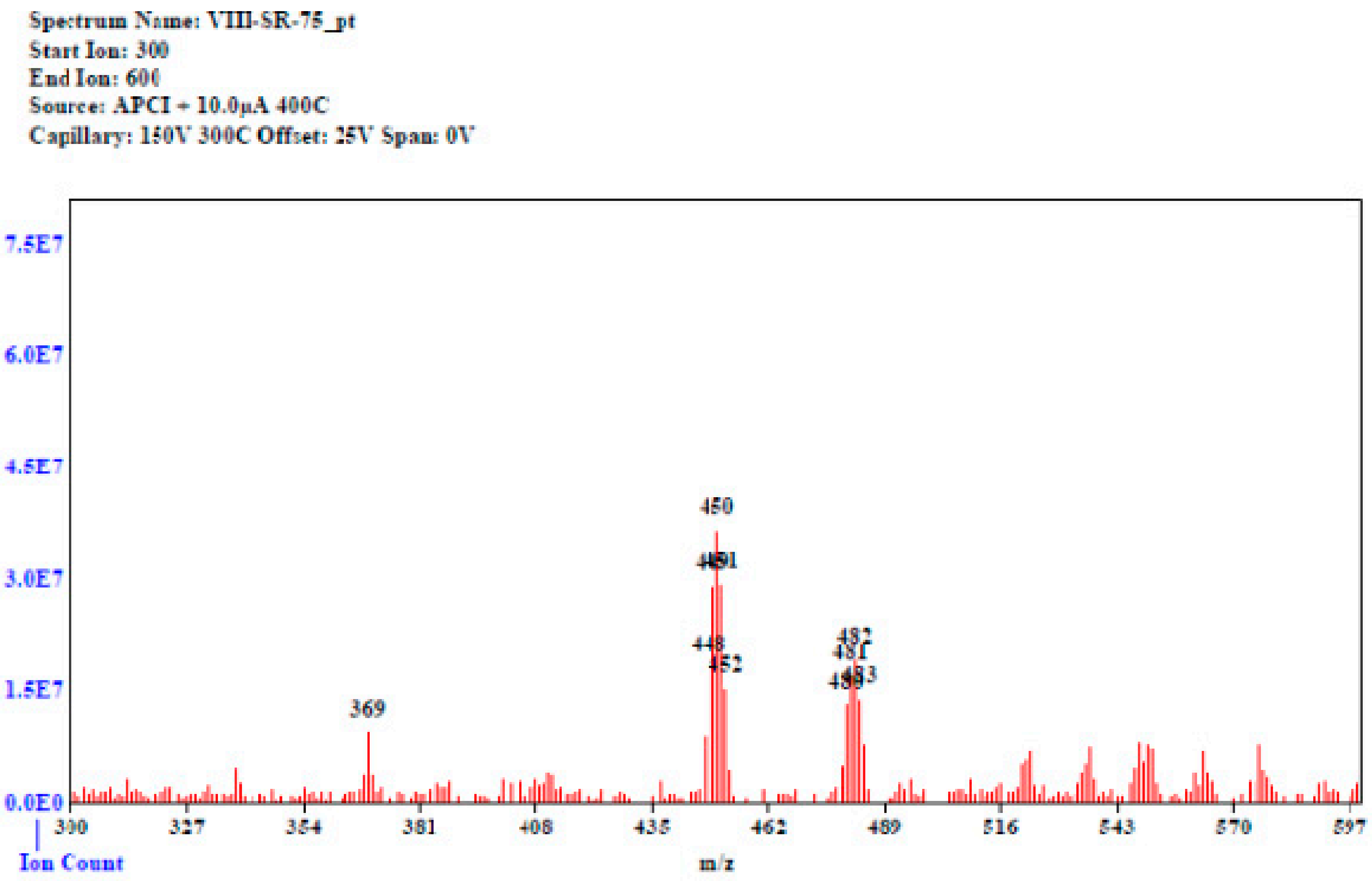

Figure S9. MS spectrum of **6**.

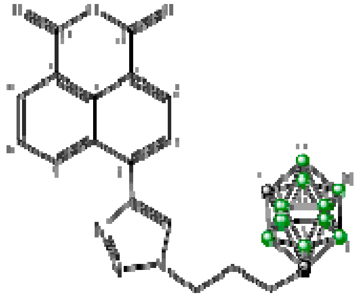

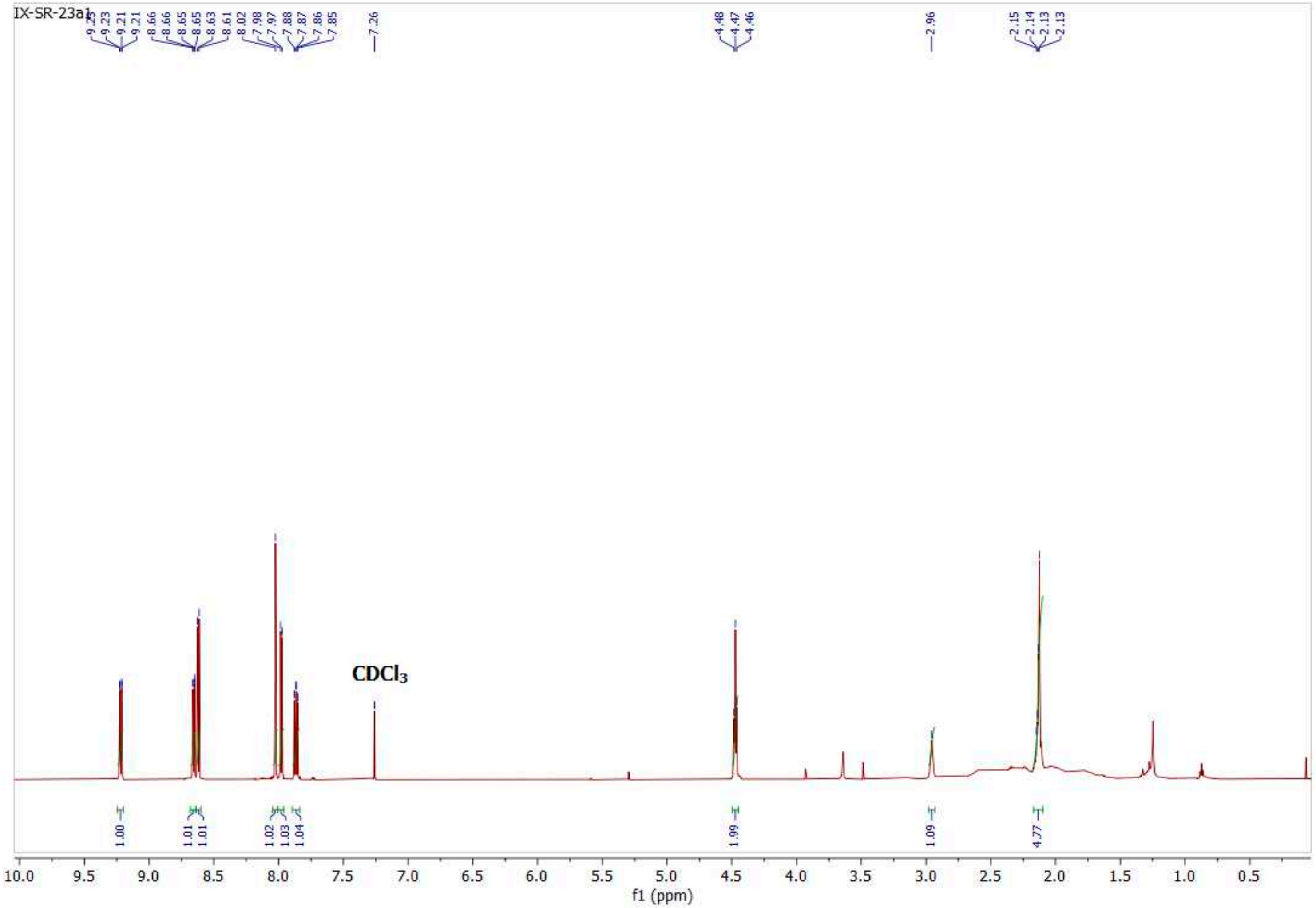

Figure S10. ^{1}H NMR spectrum of **7**.

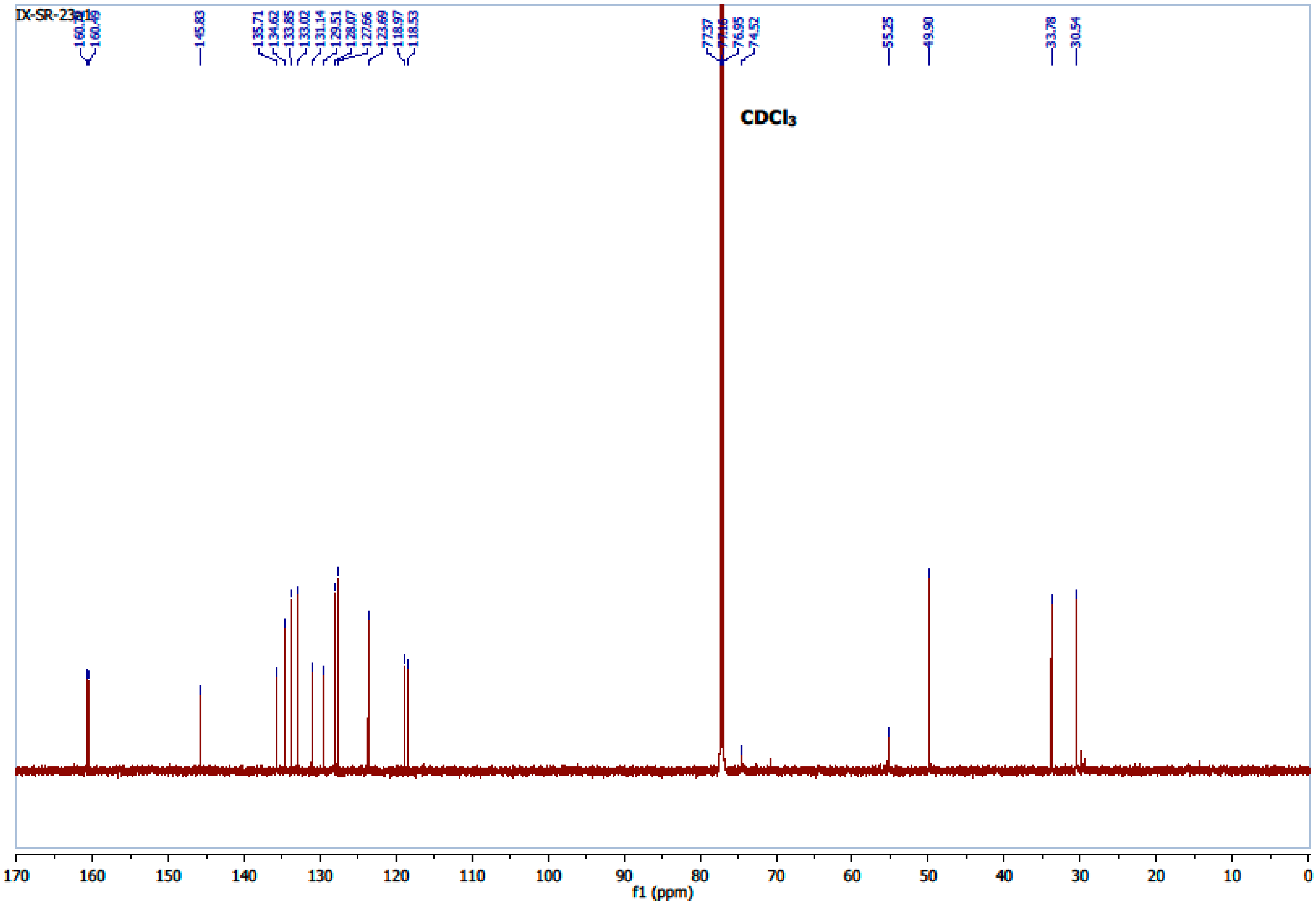

Figure S11. ^{13}C NMR spectrum of **7**.

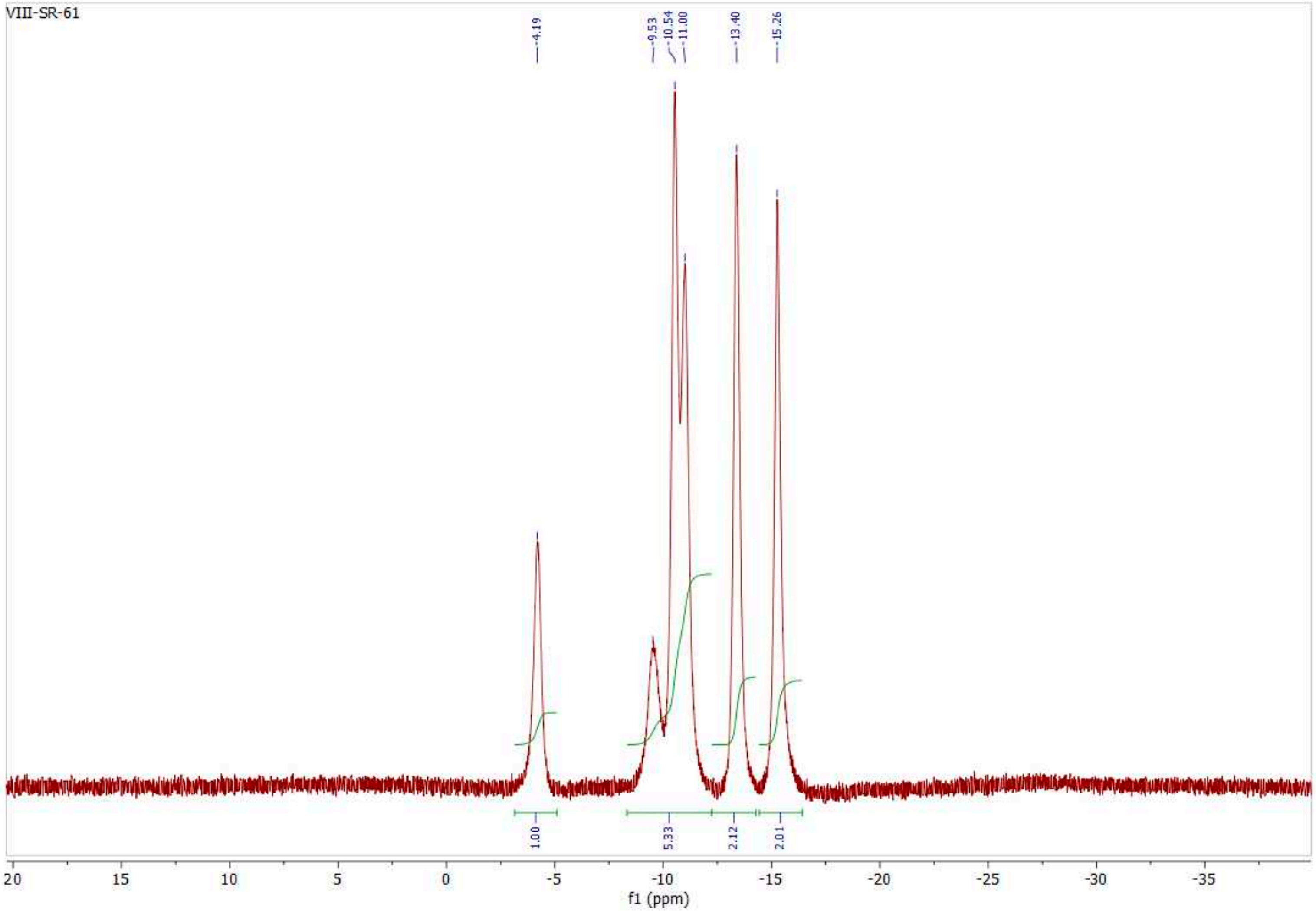

Figure S12. ^{11}B NMR spectrum of **7**.

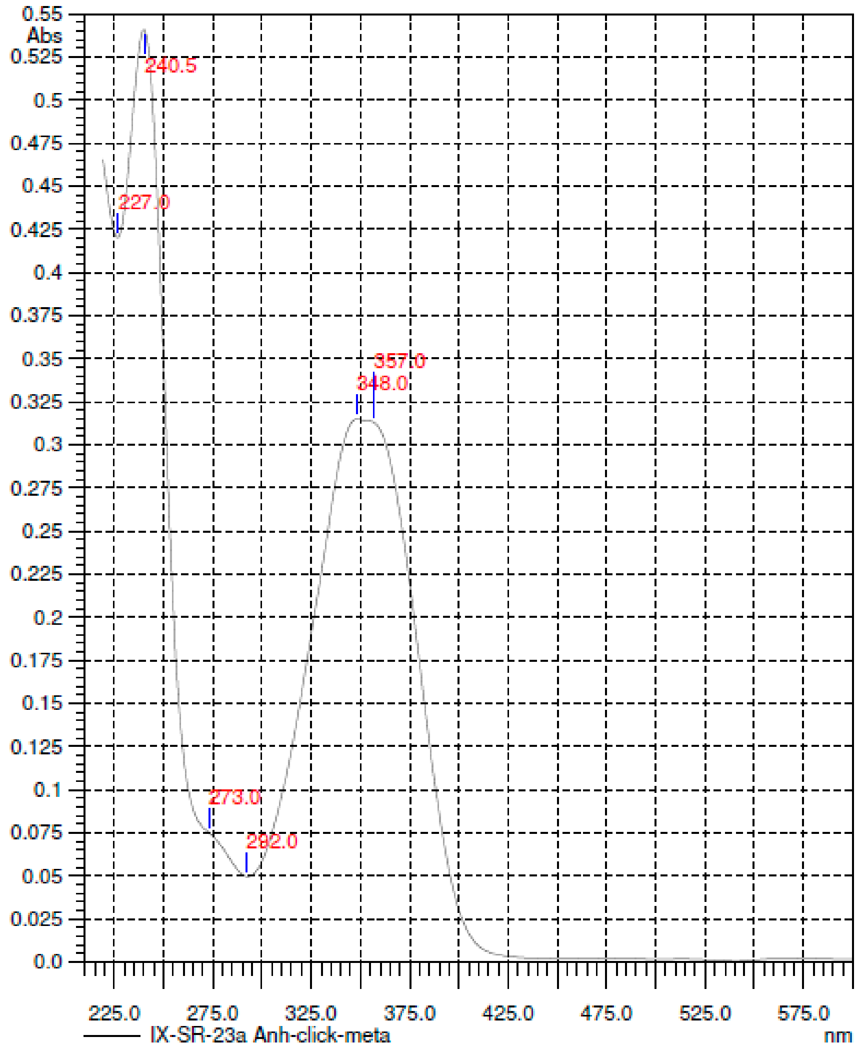

Figure S13. UV spectrum of **7**.

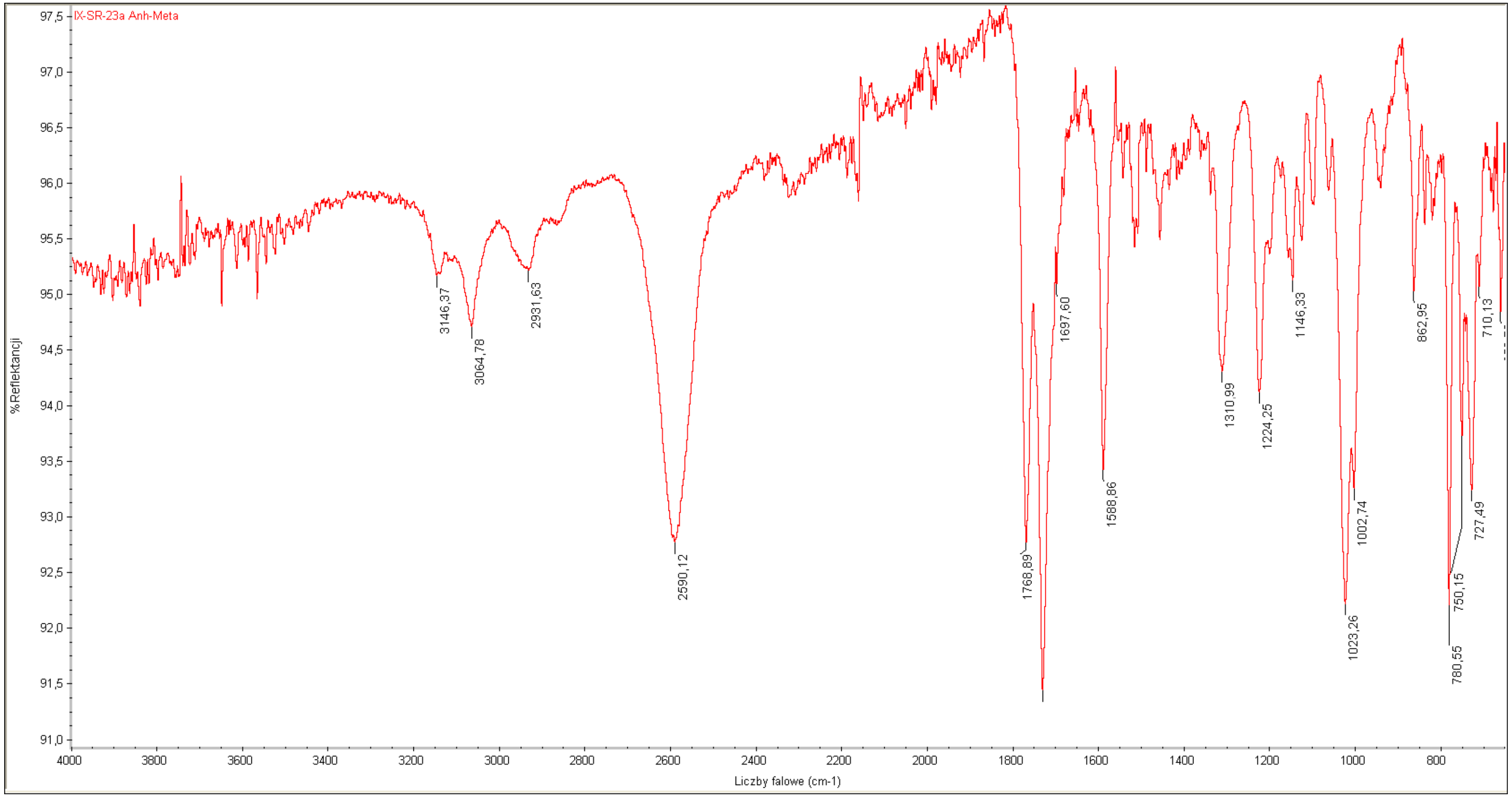

Figure S14. IR spectrum of **7**.

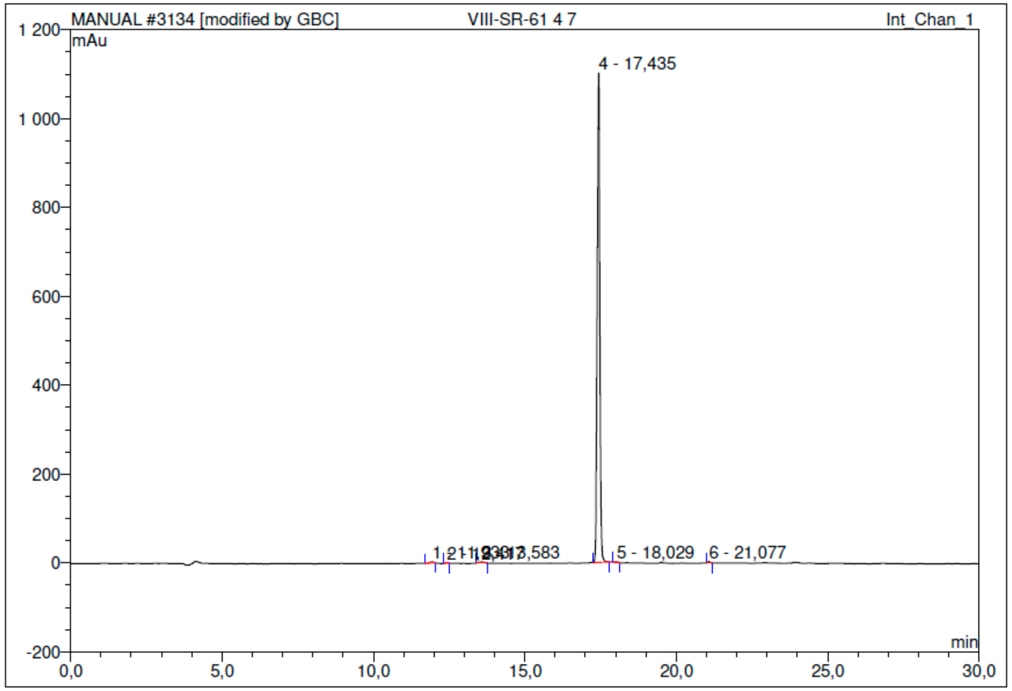

No.	Ret.Time min	Peak Name	Height mAu	Area mAu*min	Rel.Area %	Amount	Type
1	11,93	n.a.	2,497	0,292	0,27	n.a.	BMB*
2	12,42	n.a.	0,915	0,088	0,08	n.a.	BMB*
3	13,58	n.a.	2,329	0,461	0,42	n.a.	BMB*
4	17,44	n.a.	1101,051	108,762	98,80	n.a.	BMB
5	18,03	n.a.	1,377	0,189	0,17	n.a.	BMB*
6	21,08	n.a.	3,332	0,292	0,27	n.a.	BMB*
Total:			1111,501	110,084	100,00	0,000	

Figure S15. HPLC analysis of **7**.

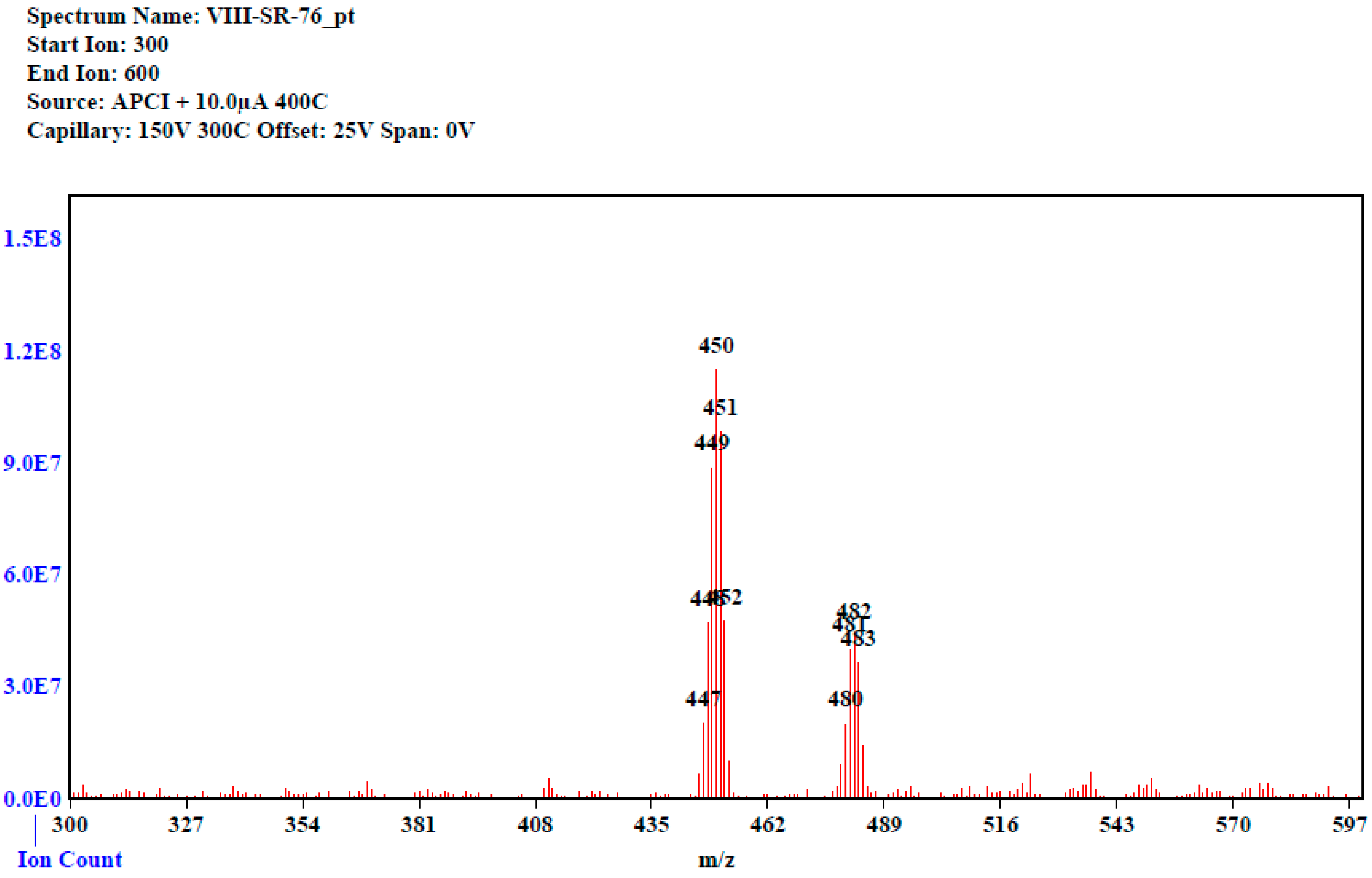

Figure S16. MS spectrum of **7**.

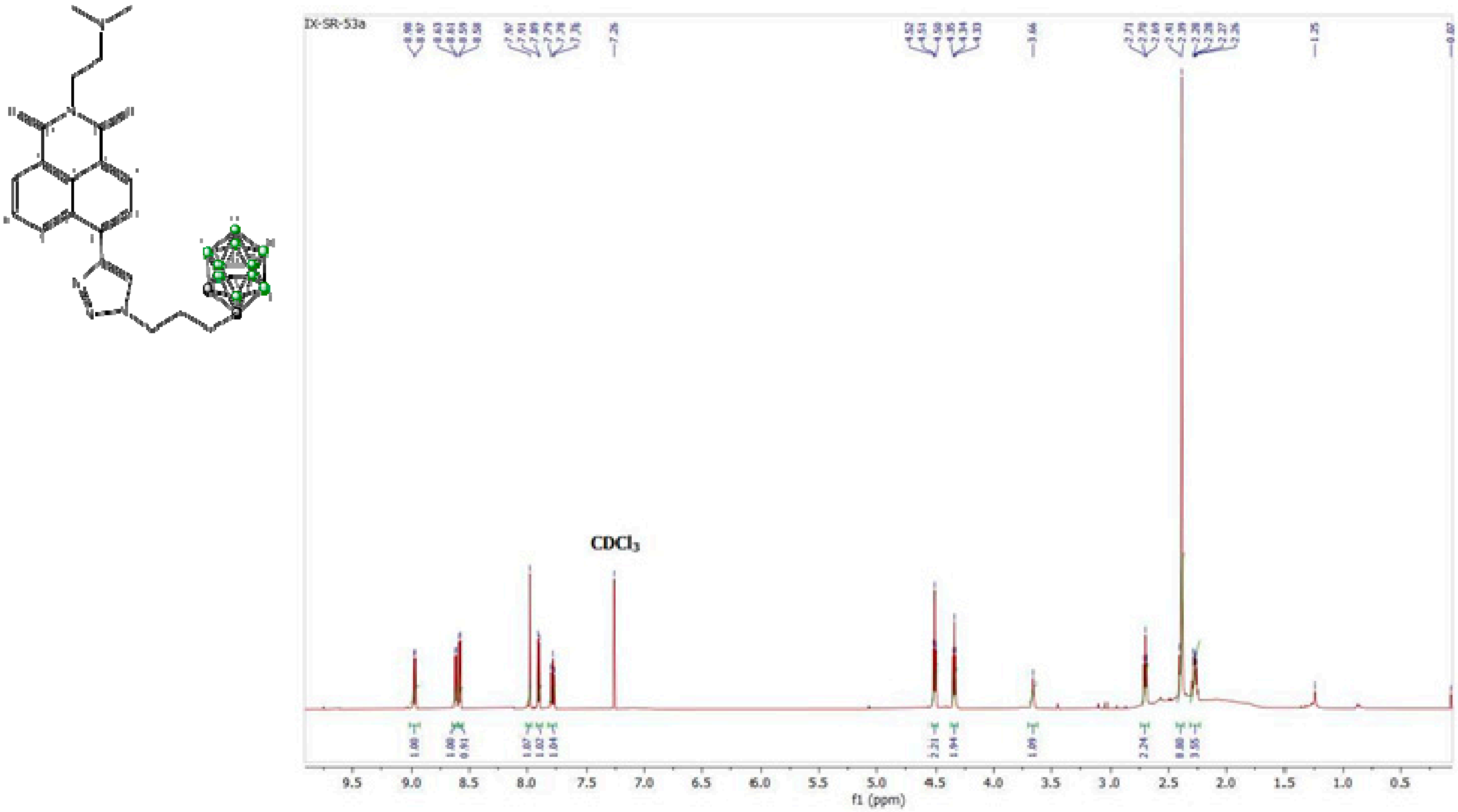

Figure S17. ^{1}H NMR spectrum of **8**.

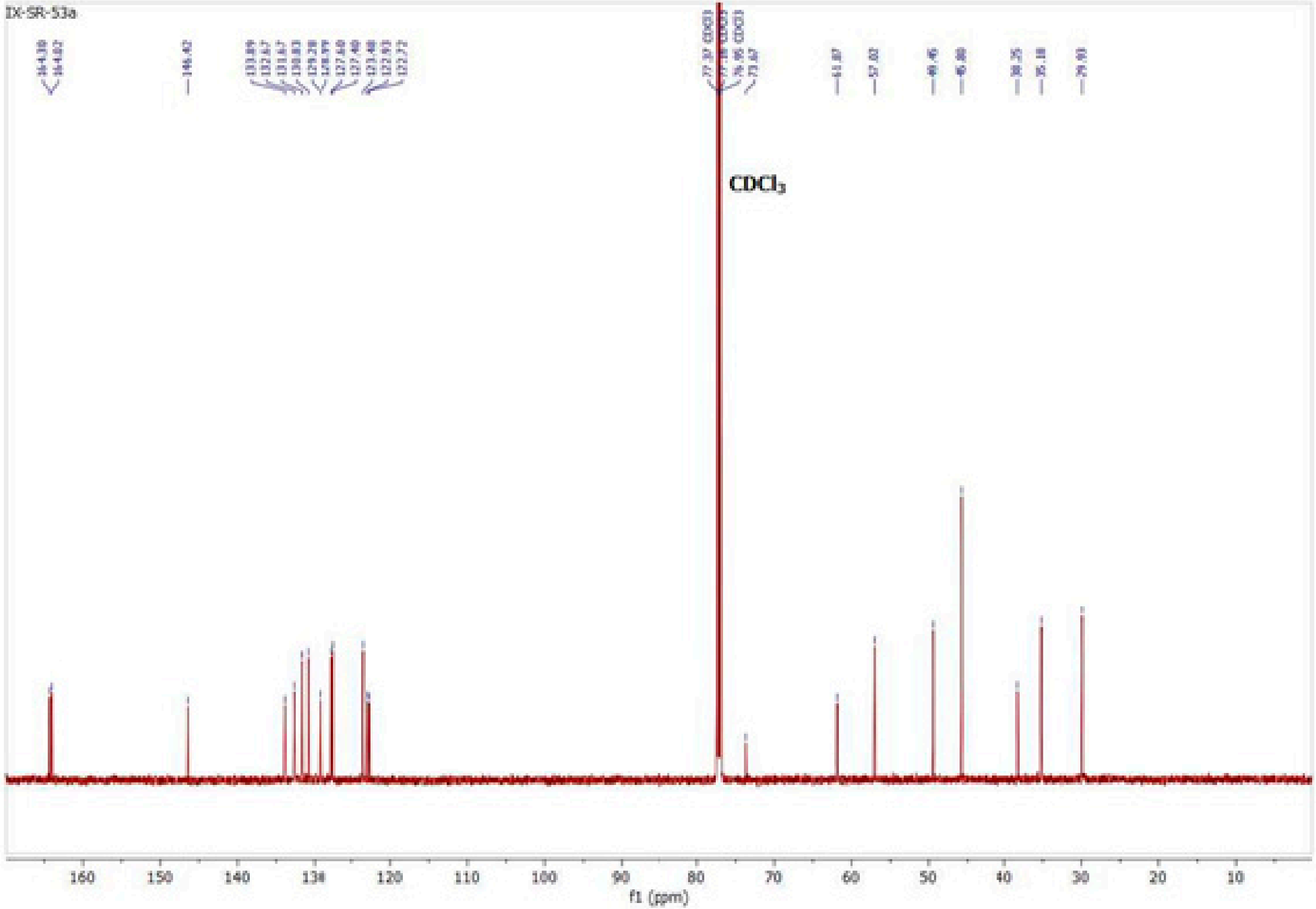

Figure S18. ^{13}C NMR spectrum of **8**.

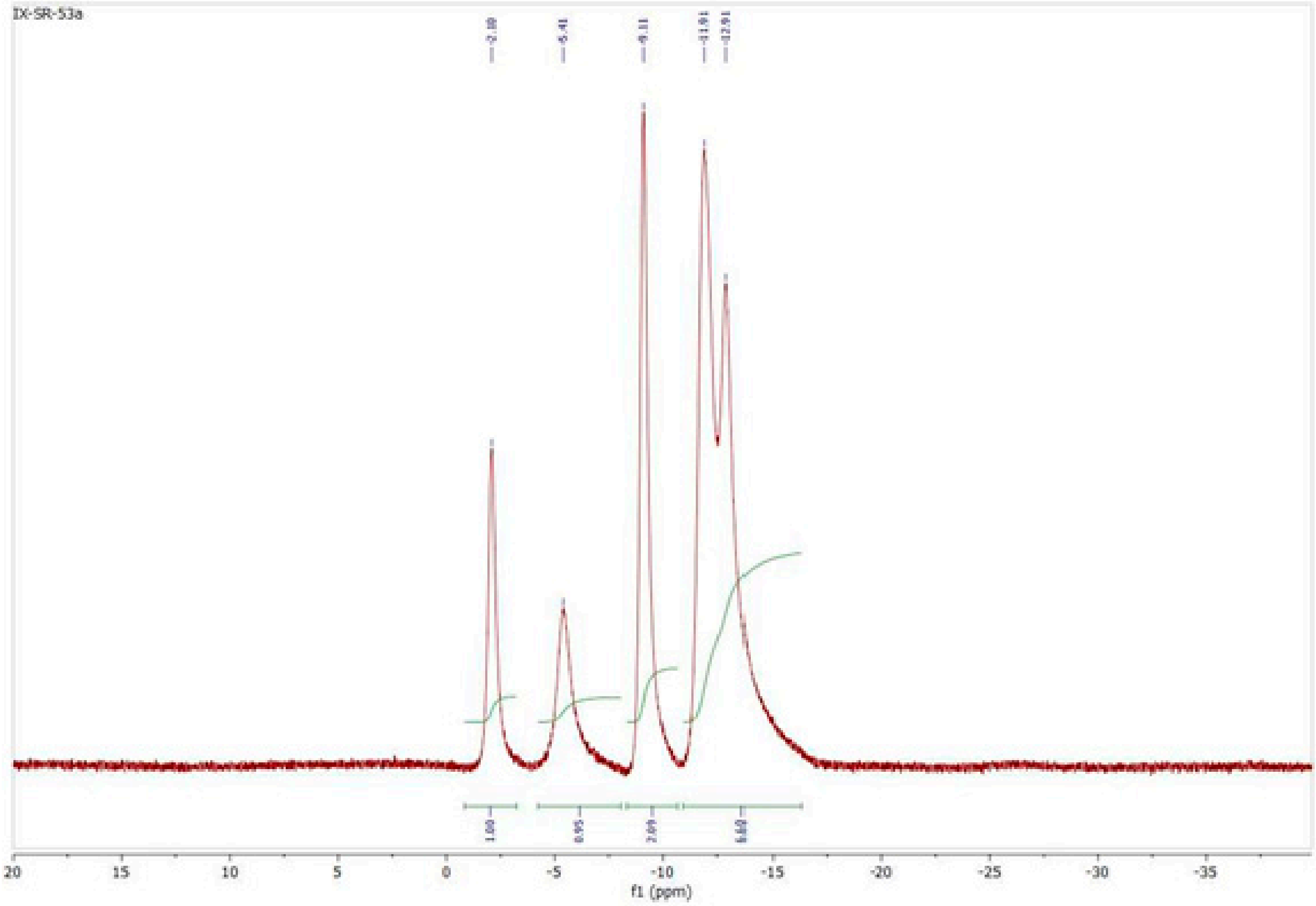

Figure S19. ^{11}B NMR spectrum of **8**.

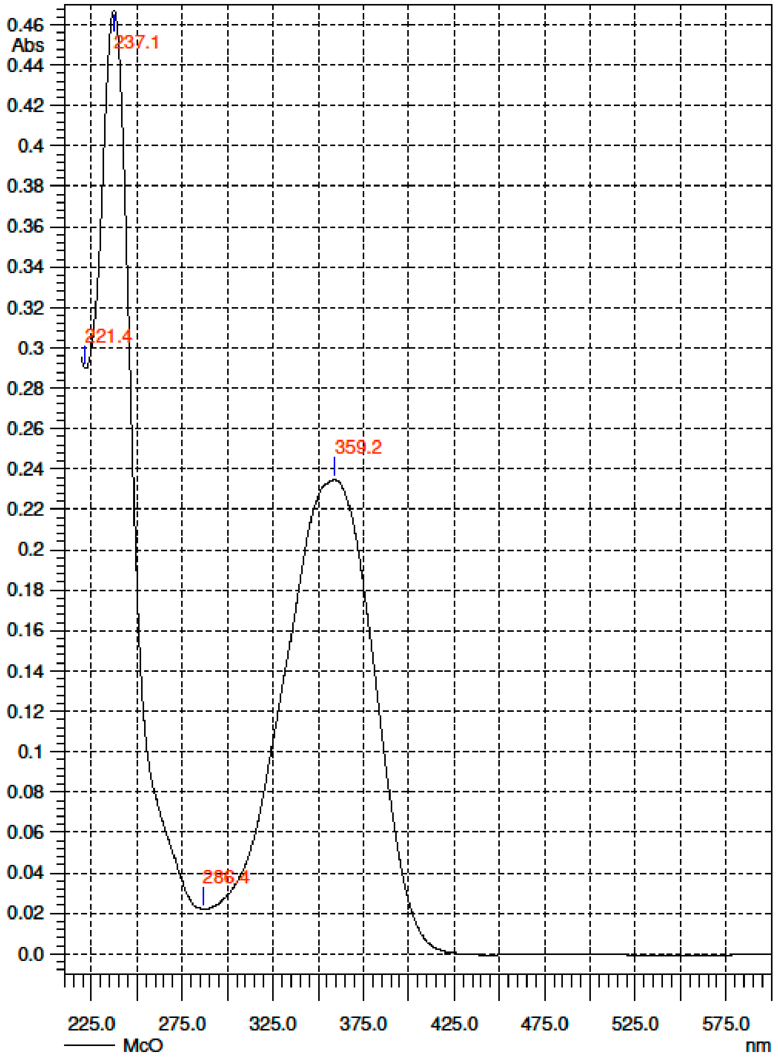

Figure S20. UV spectrum of **8**.

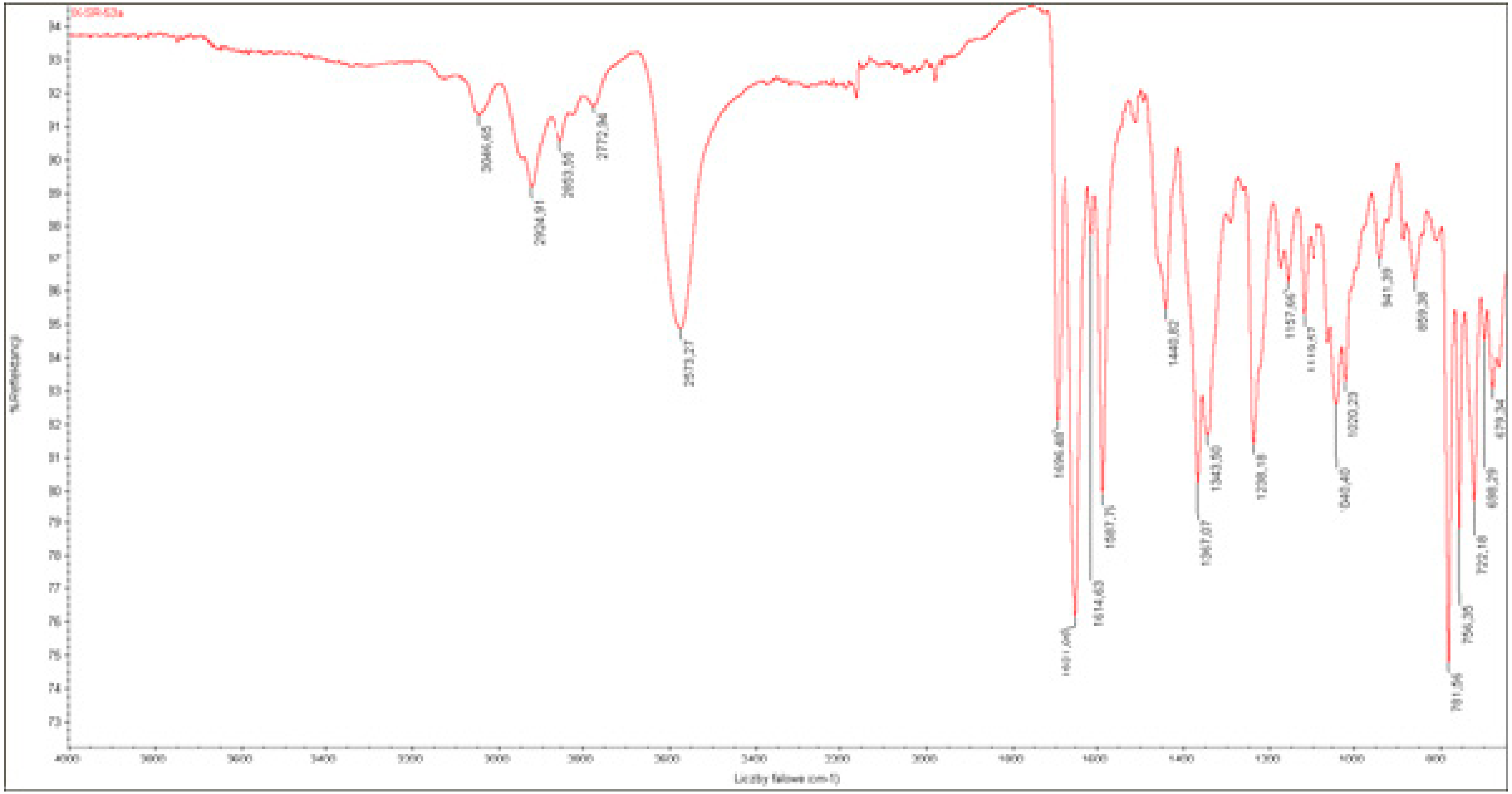

Figure S21. IR spectrum of **8**.

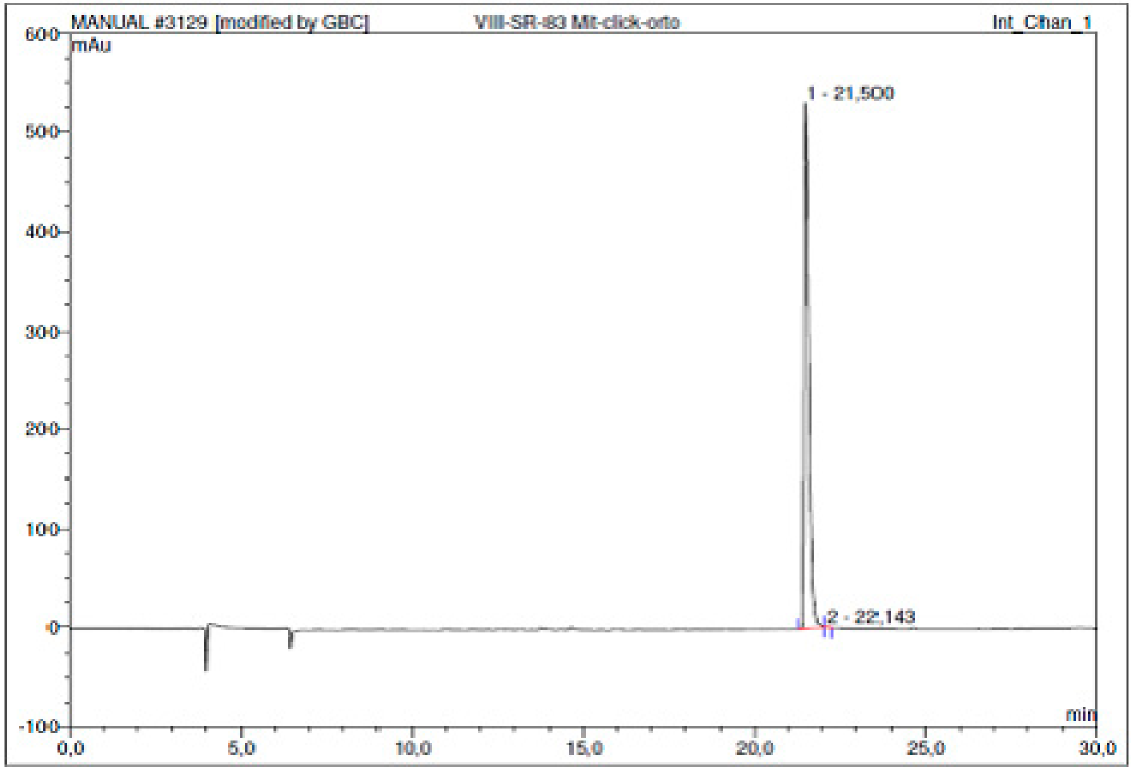

No.	Ret.Time min	Peak Name	Height mAu	Area mAu*min	Rel.Area %	Amount	Type
1	21,50	n.a.	531,082	85,171	99,96	n.a.	BMB
2	22,14	n.a.	0,370	0,038	0,04	n.a.	BMB*
Total:			531,452	85,209	100,00	0,000	

Figure S22. HPLC analysis of **8**.

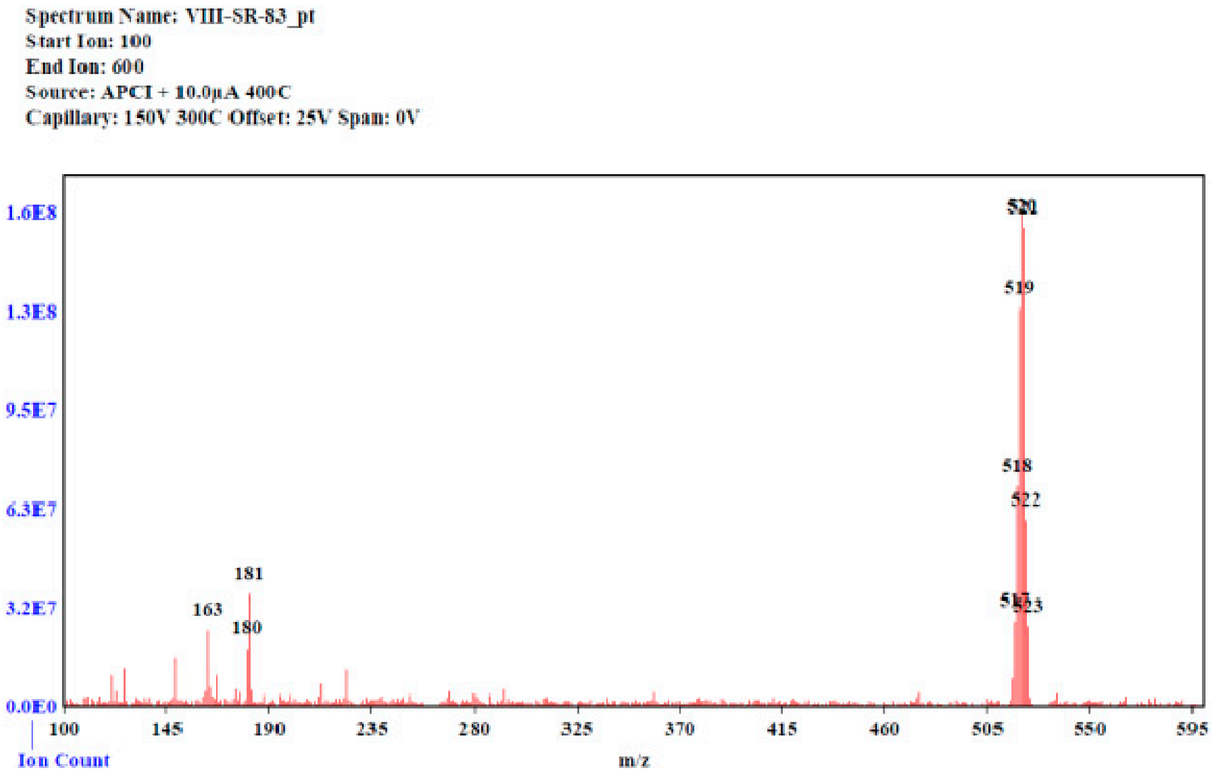

Figure S23. MS spectrum of **8**.

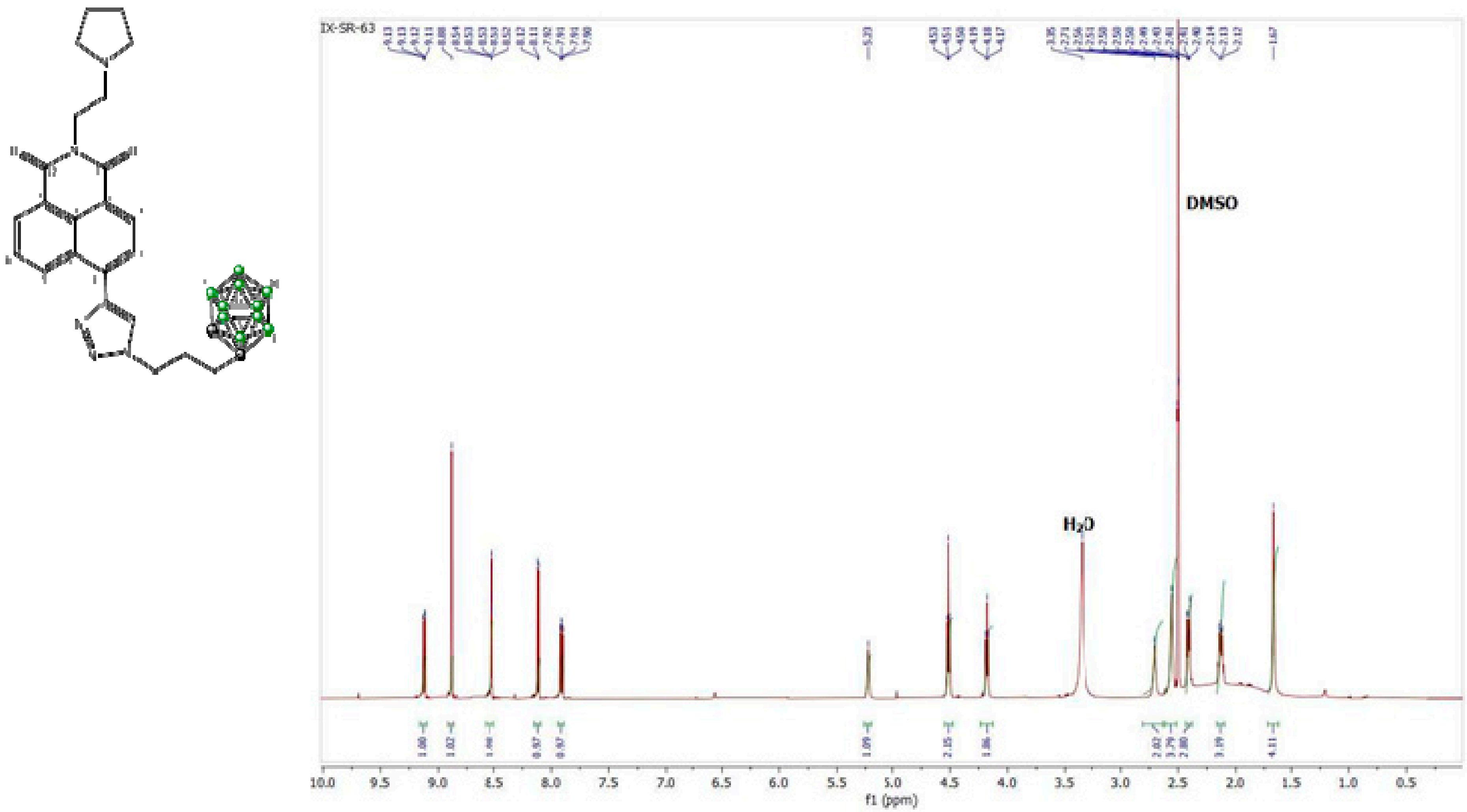

Figure S24. ^{1}H NMR spectrum of **9**.

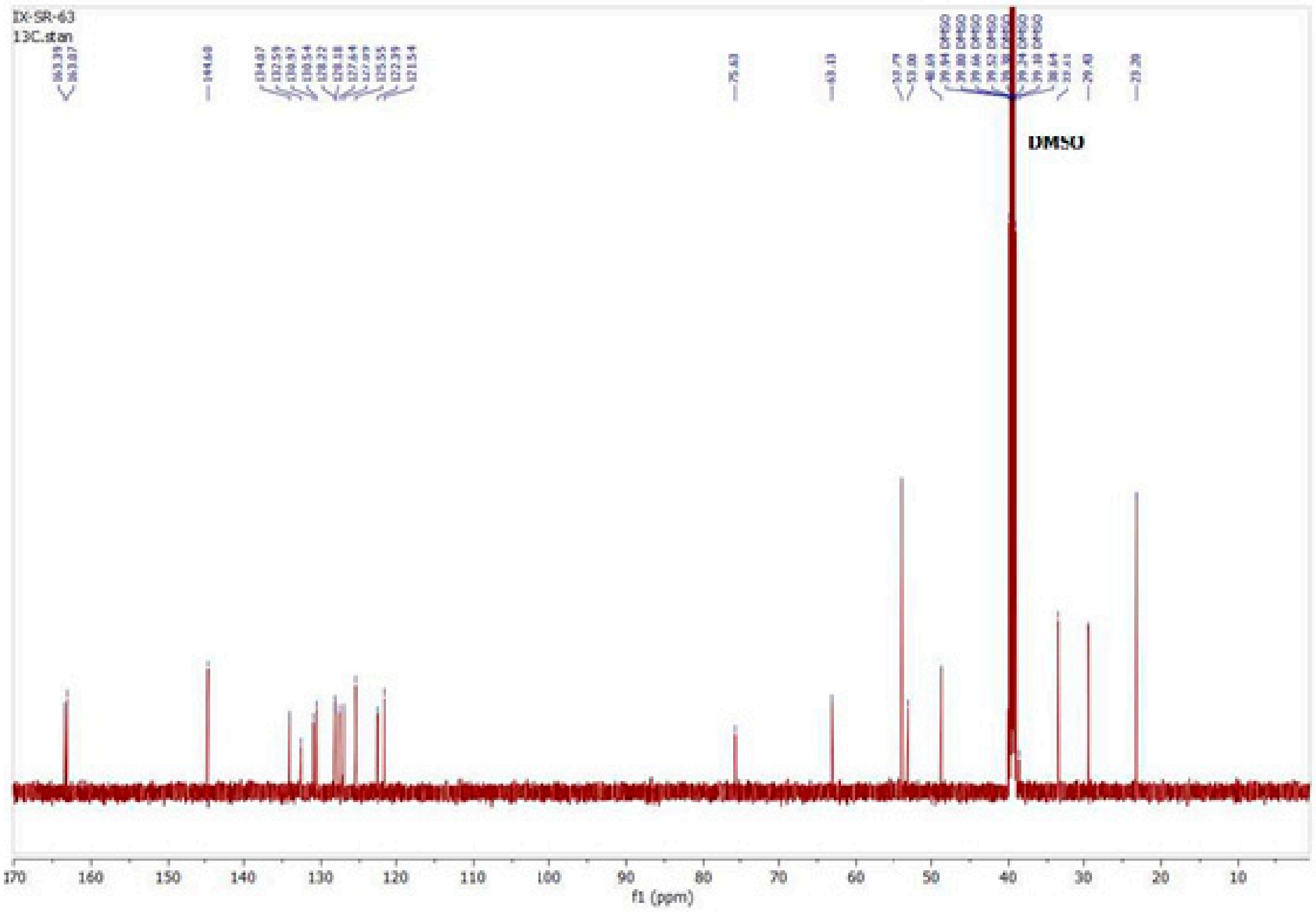

Figure S25. ^{13}C NMR spectrum of **9**.

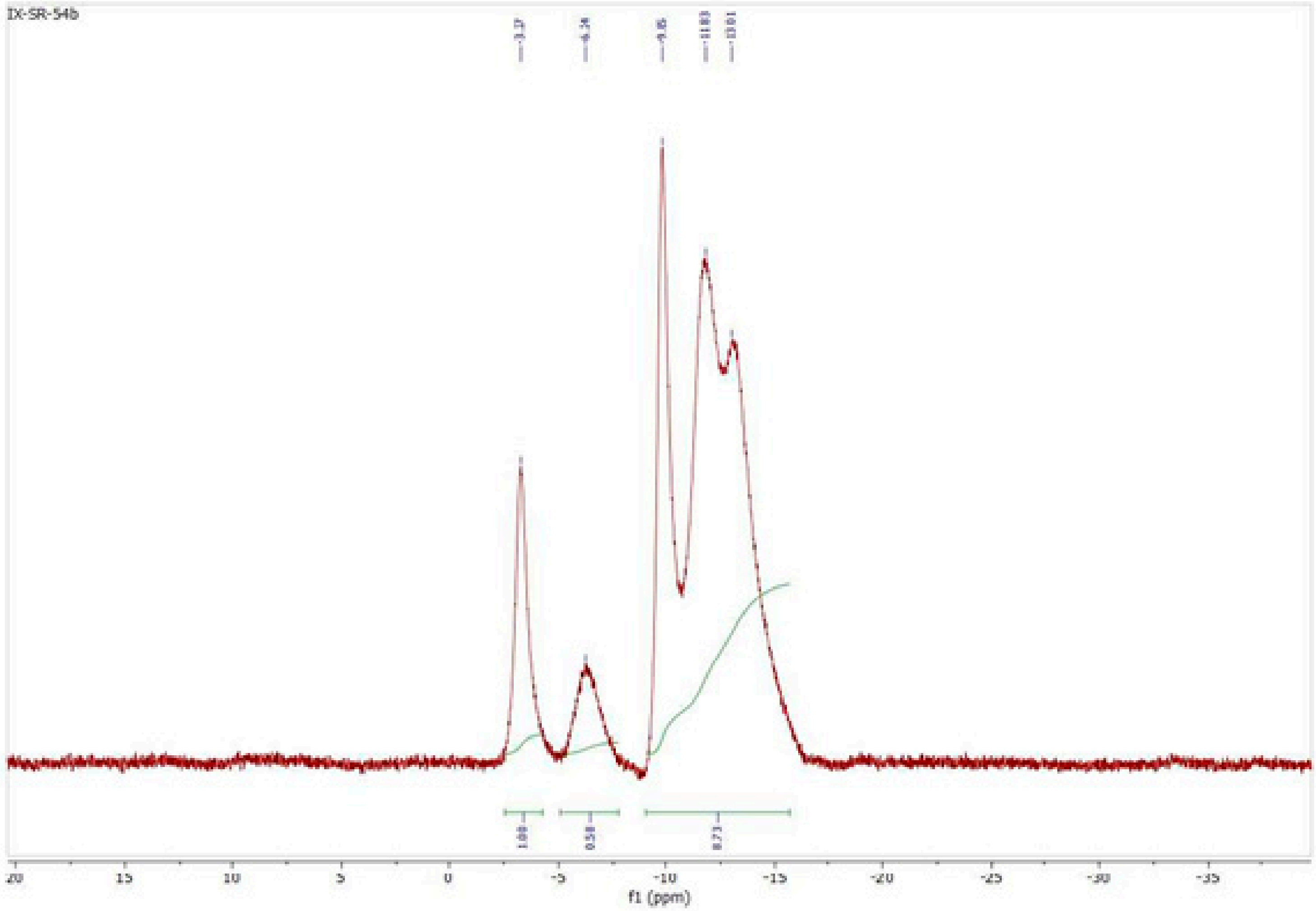

Figure S26. ^{11}B NMR spectrum of **9**.

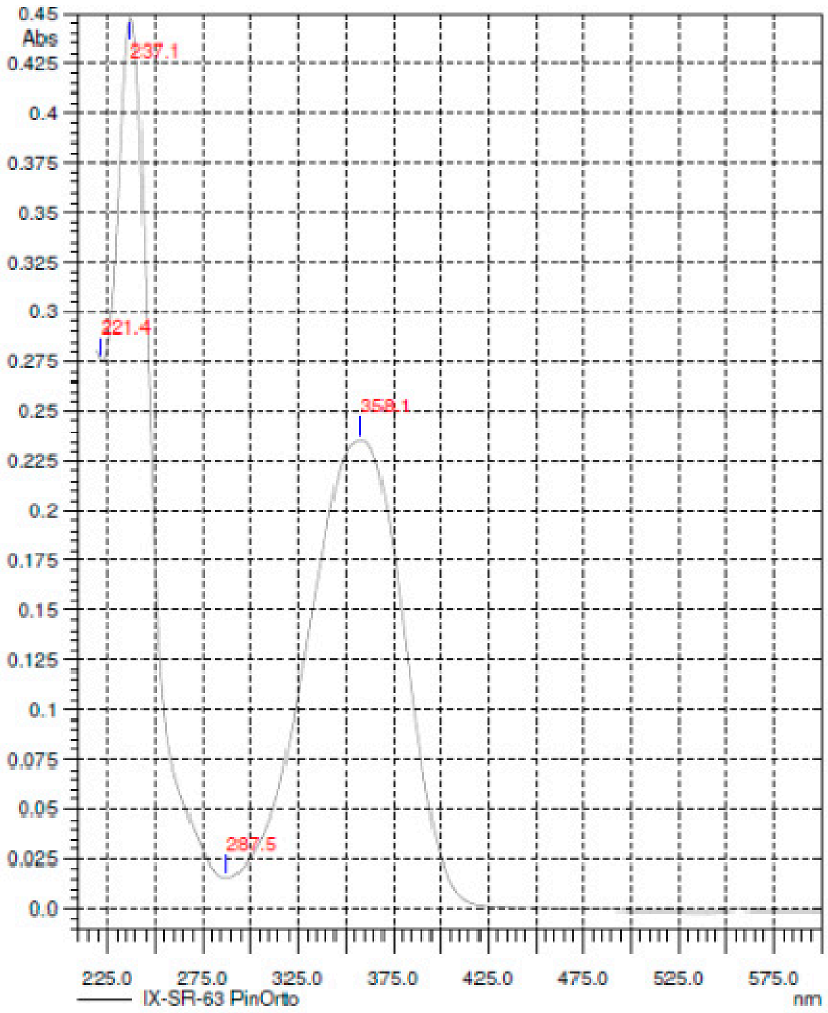

Figure S27. UV spectrum of **9**.

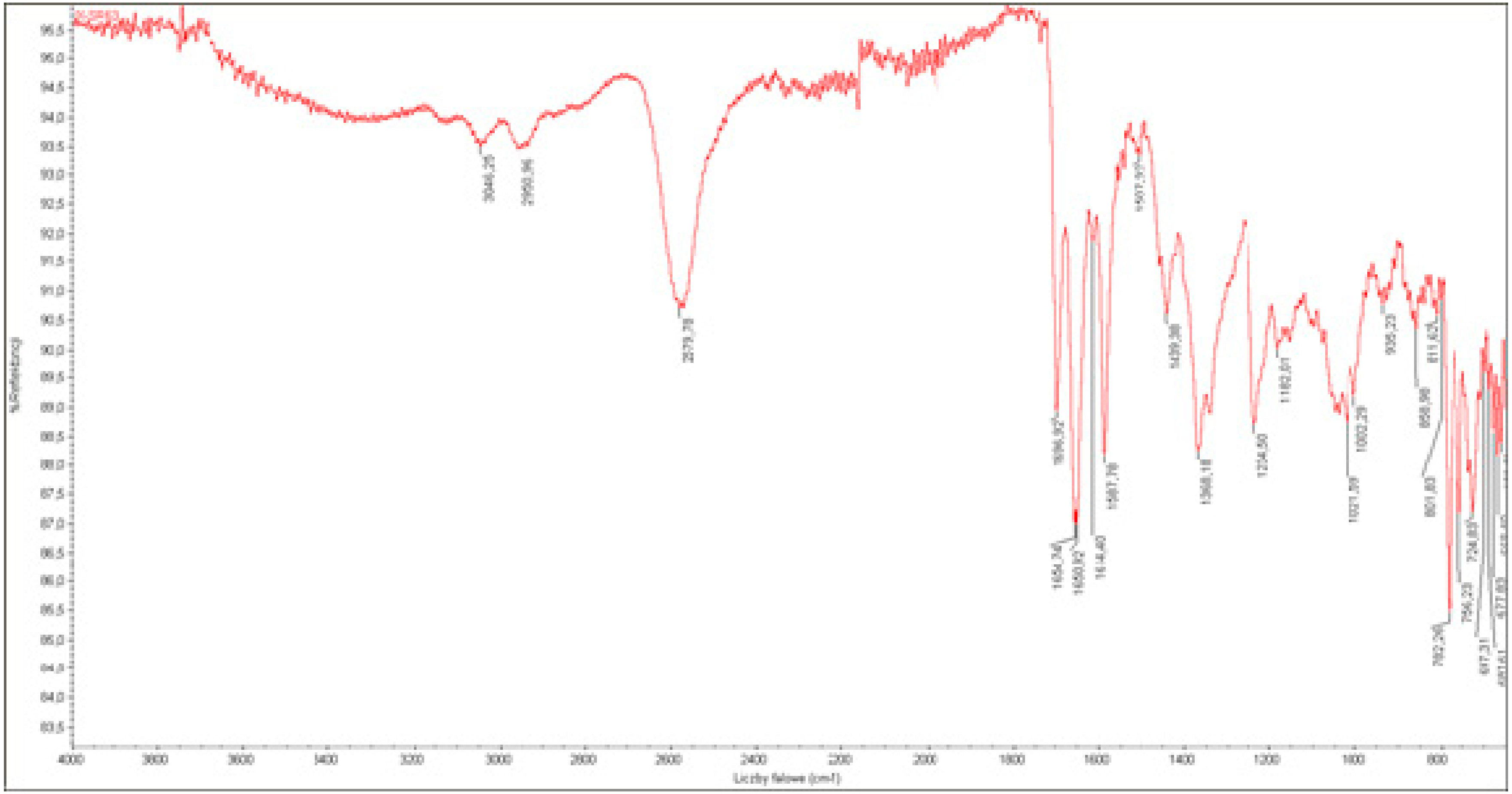

Figure S28. IR spectrum of **9**.

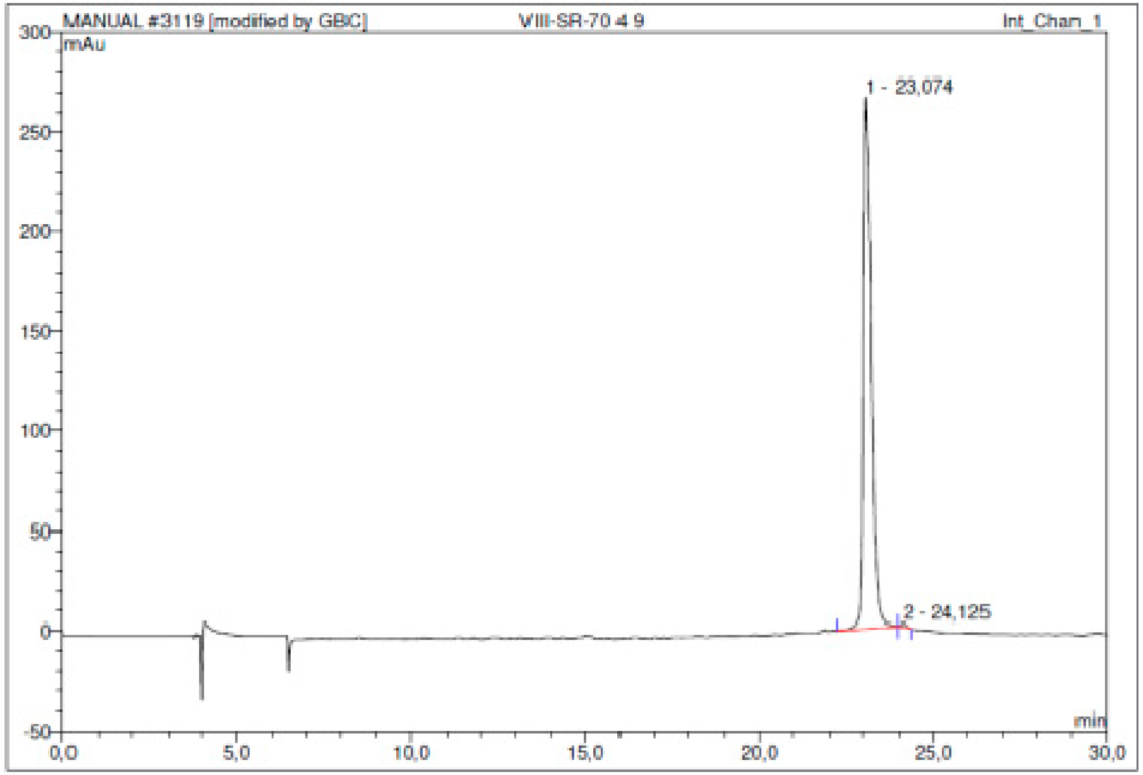

No.	Ret.Time min	Peak Name	Height mAu	Area mAu*min	Rel.Area %	Amount	Type
1	23,07	n.a.	266,597	72,719	99,56	n.a.	BMb*
2	24,12	n.a.	1,950	0,318	0,44	n.a.	bMB*
Total:			268,547	73,038	100,00	0,000	

Figure S29. HPLC analysis of **9**.

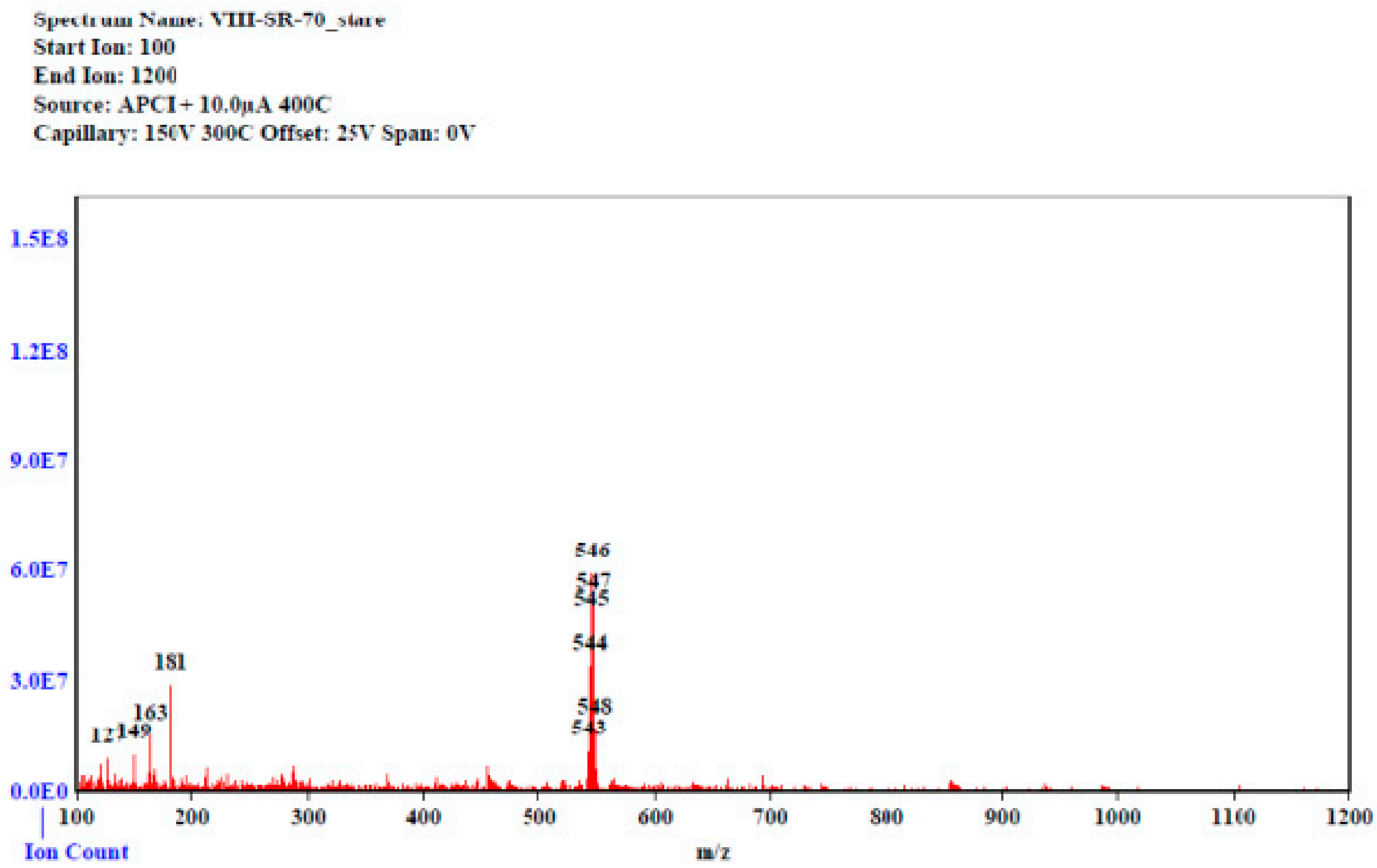

Figure S30. MS spectrum of **9**.

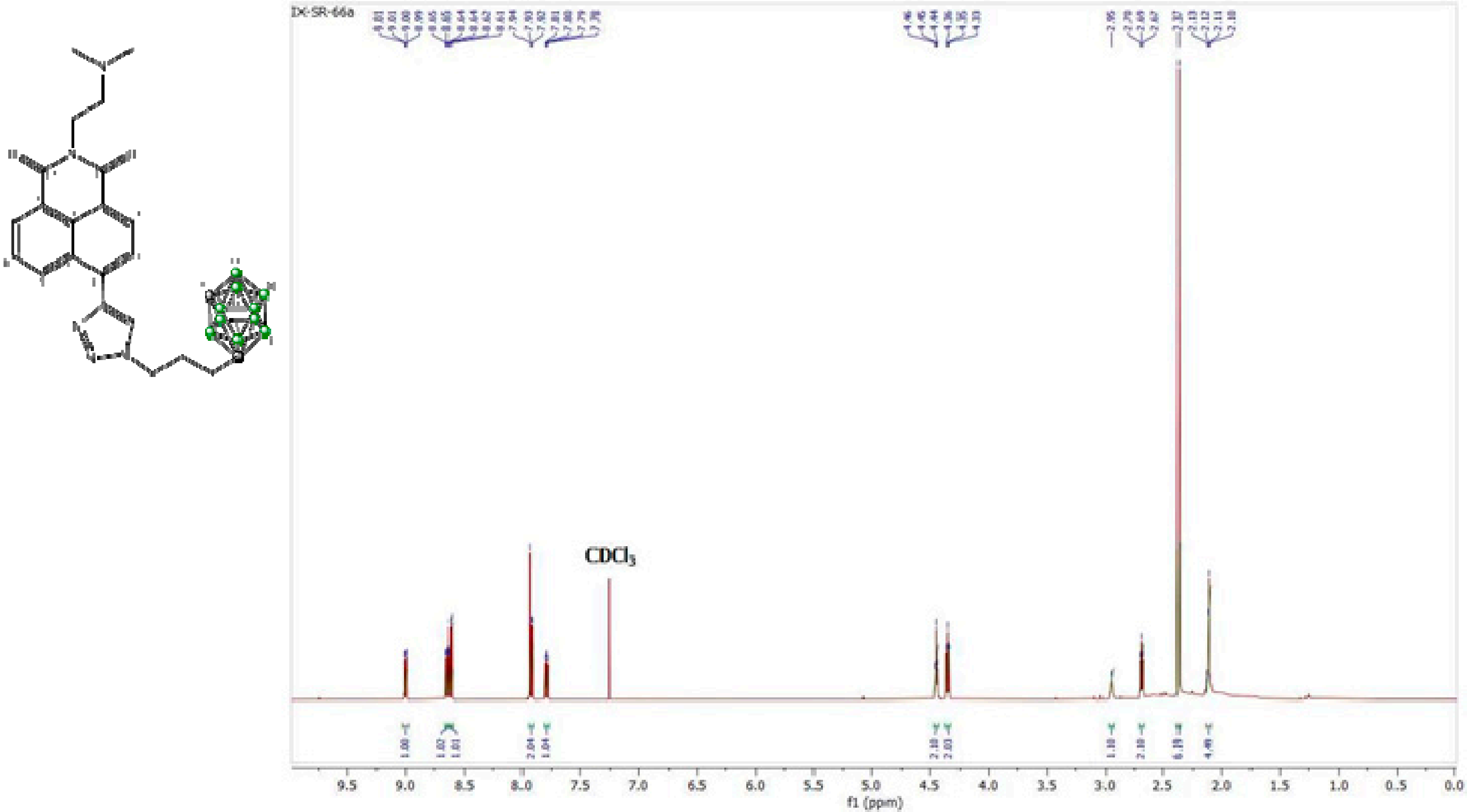

Figure S31. ^{1}H NMR spectrum of **10**.

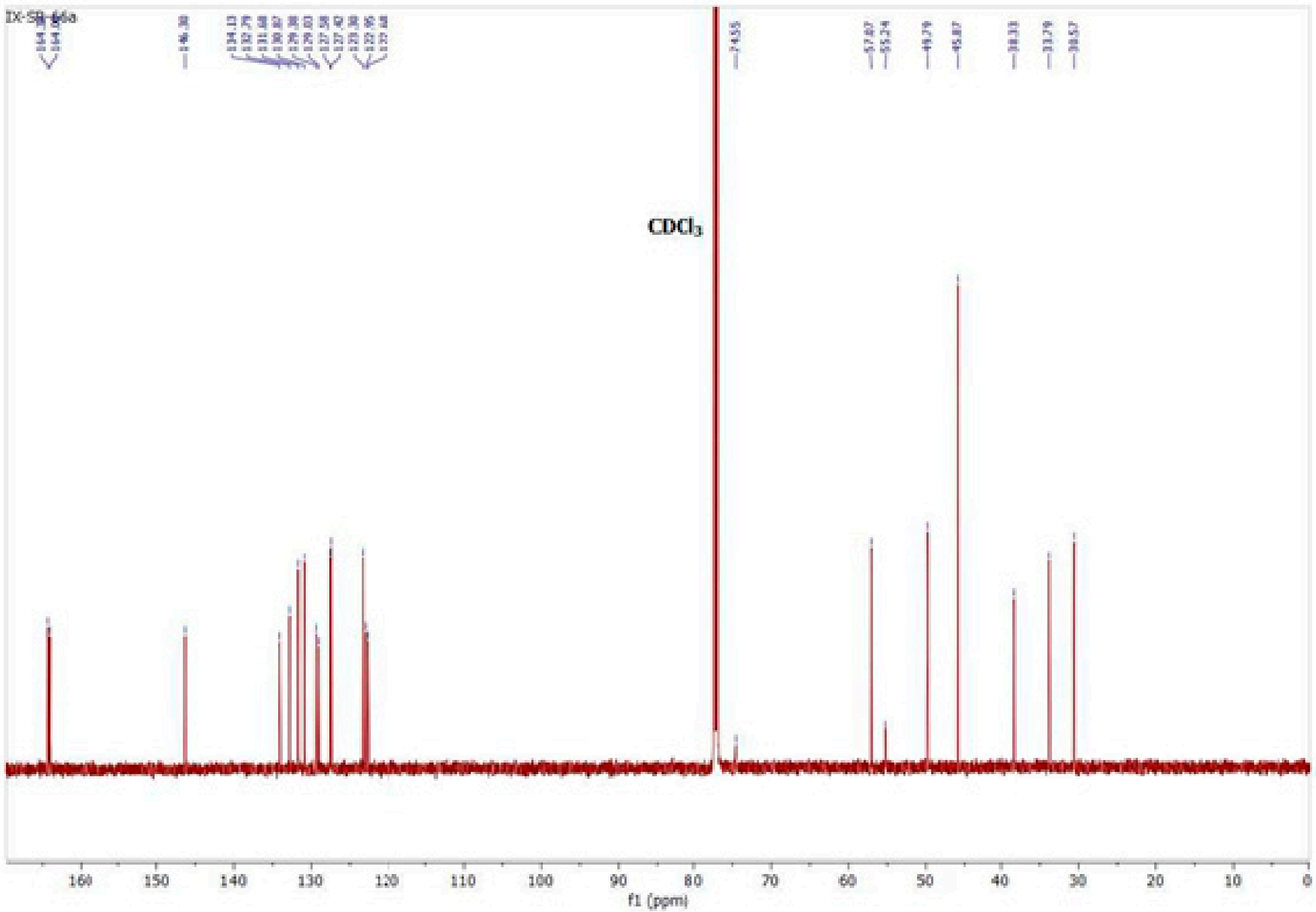

Figure S32. ^{13}C NMR spectrum of **10**.

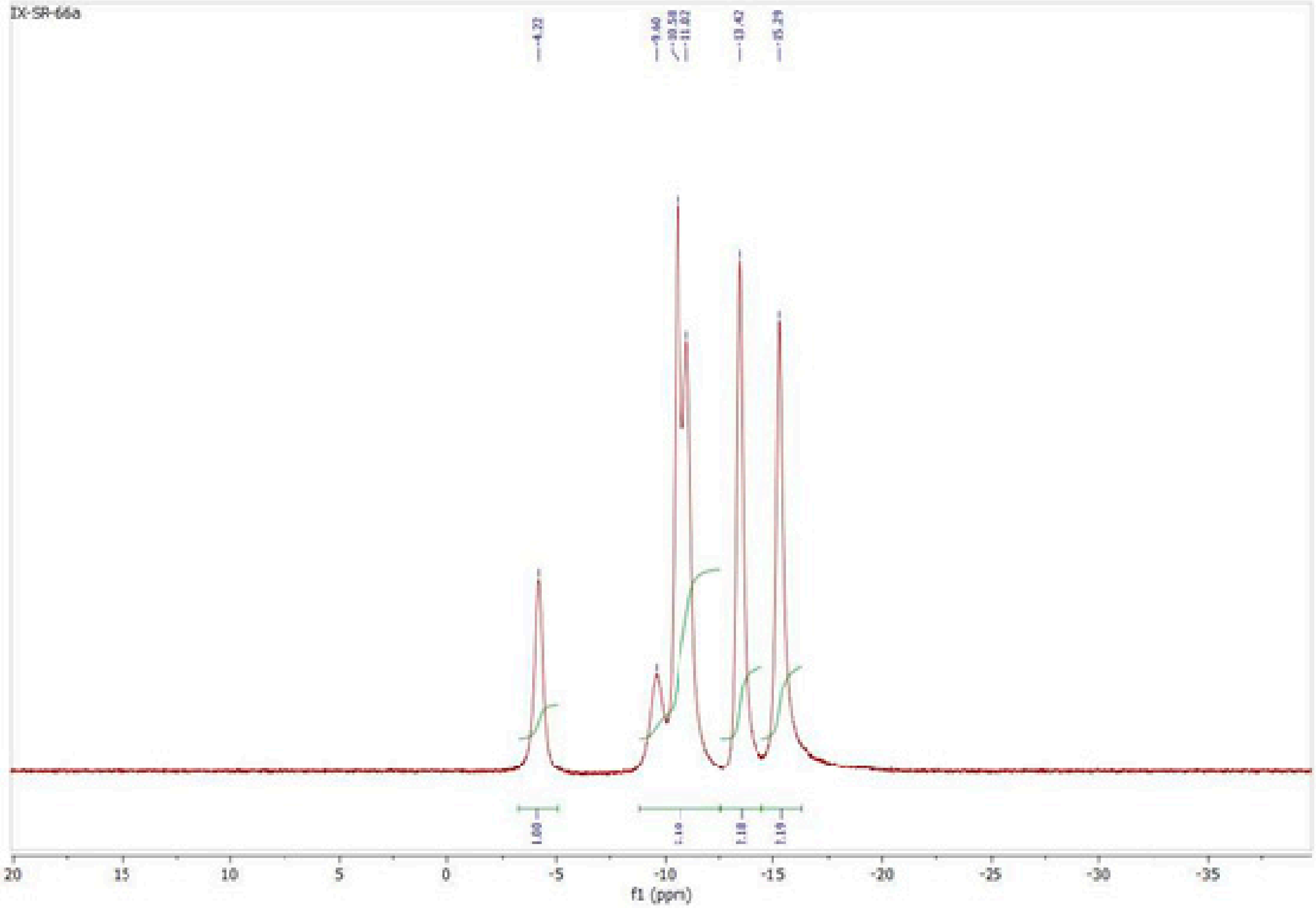

Figure S33. ^{11}B NMR spectrum of **10**.

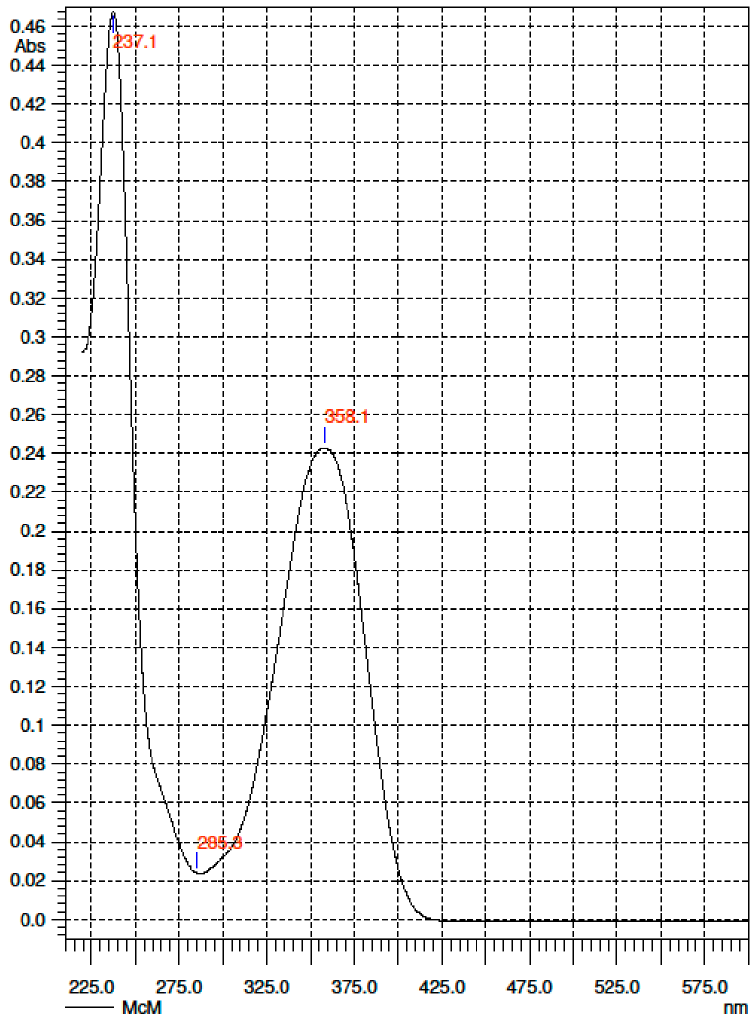

Figure S34. UV spectrum of **10**.

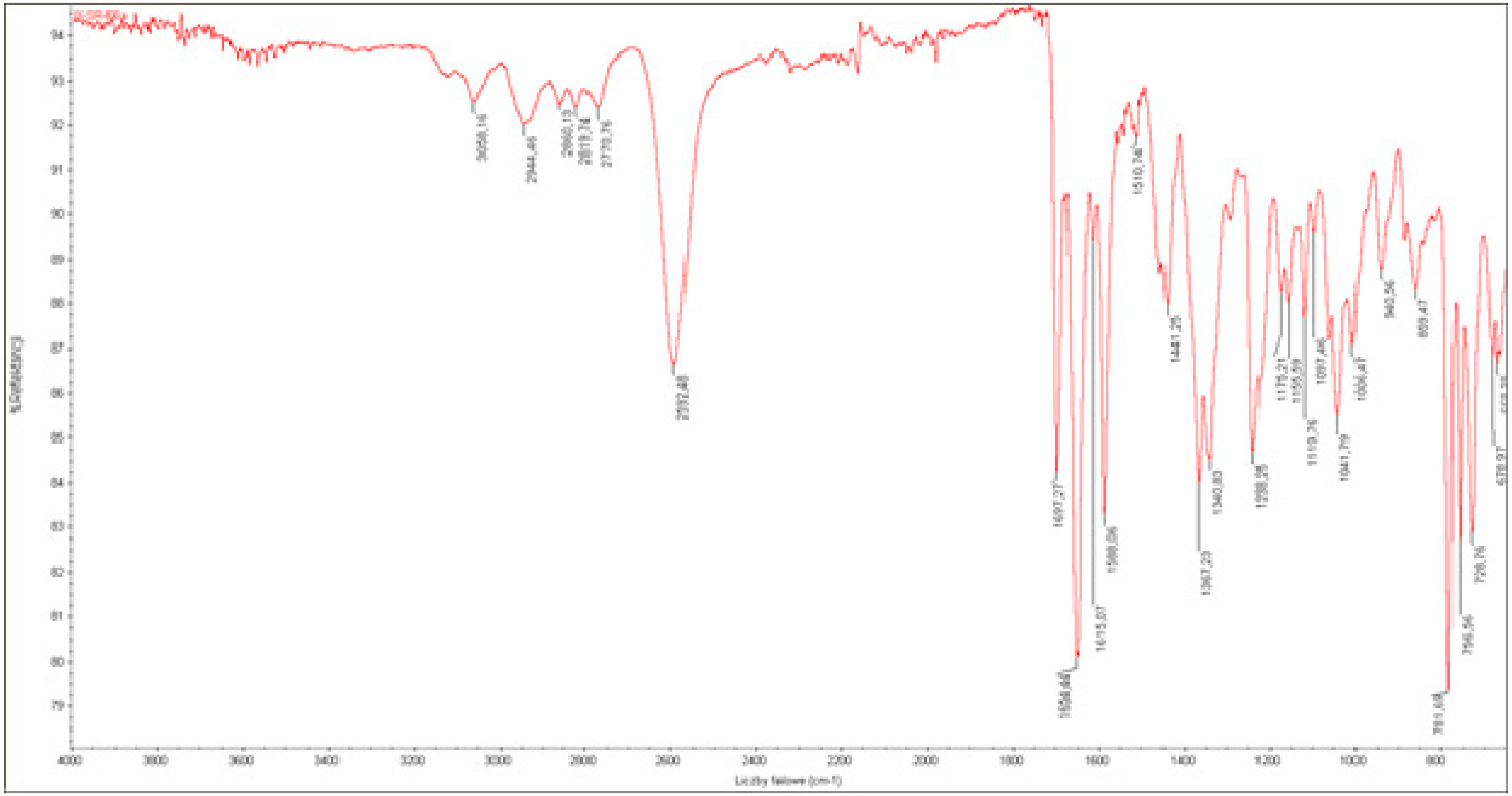

Figure S35. IR spectrum of **10**.

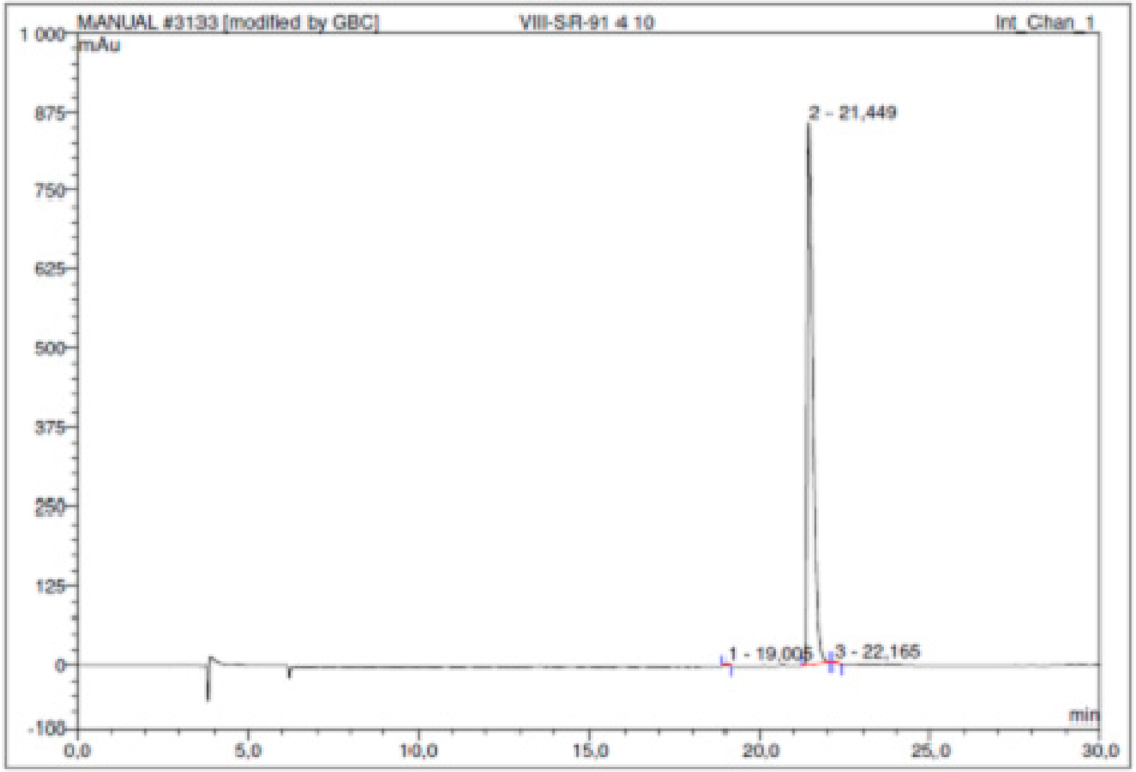

No.	Ret.Time min	Peak Name	Height mAu	Area mAu*min	Rel.Area %	Amount	Type
1	19,01	n.a.	2,694	0,301	0,18	n.a.	BMB*
2	21,45	n.a.	857,958	169,015	99,74	n.a.	BMb*
3	22,17	n.a.	1,135	0,143	0,08	n.a.	bMB*
Total:			861,788	169,459	100,00	0,000	

Figure S36. HPLC analysis of **10**.

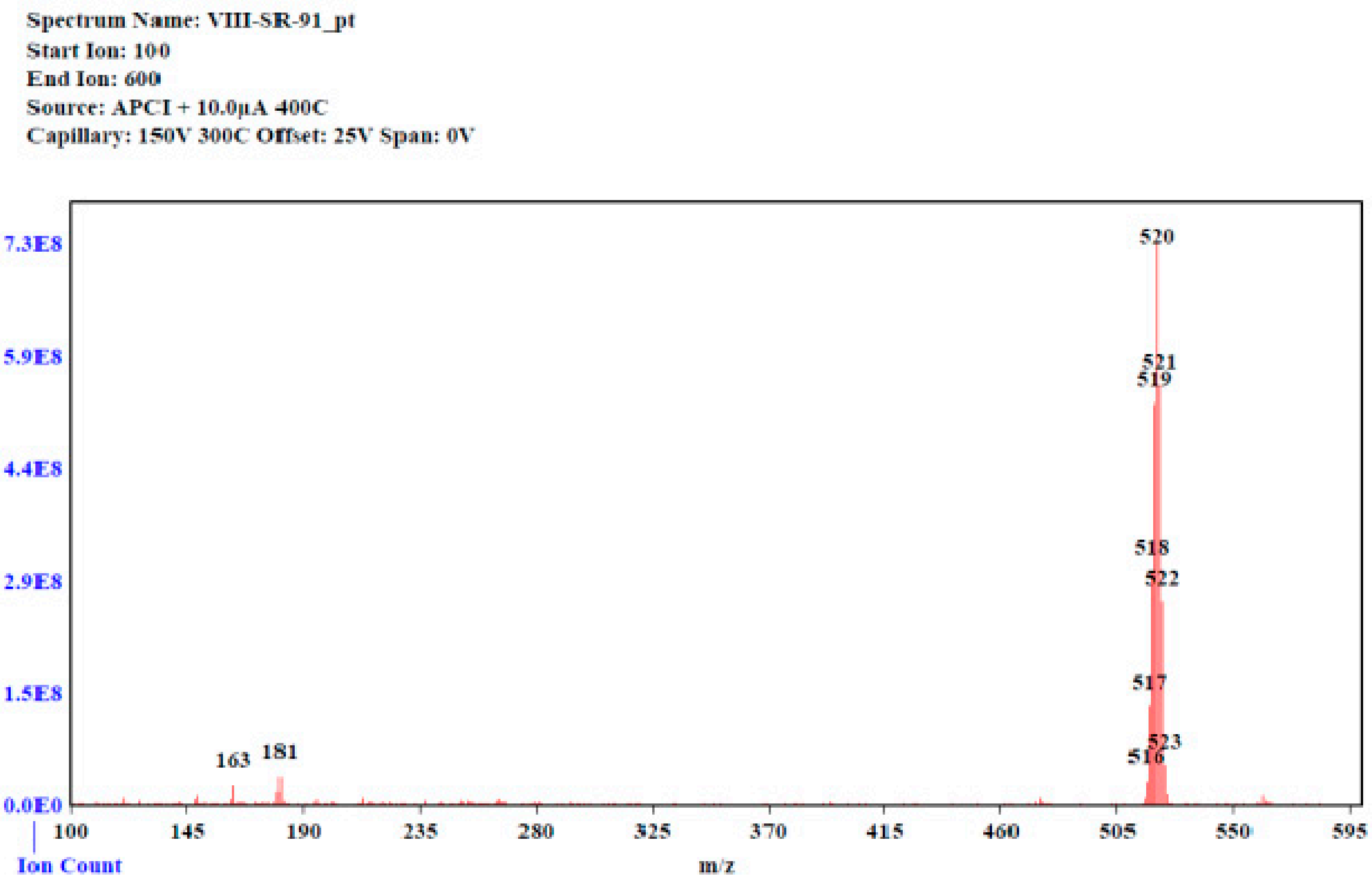

Figure S37. MS spectrum of **10**.

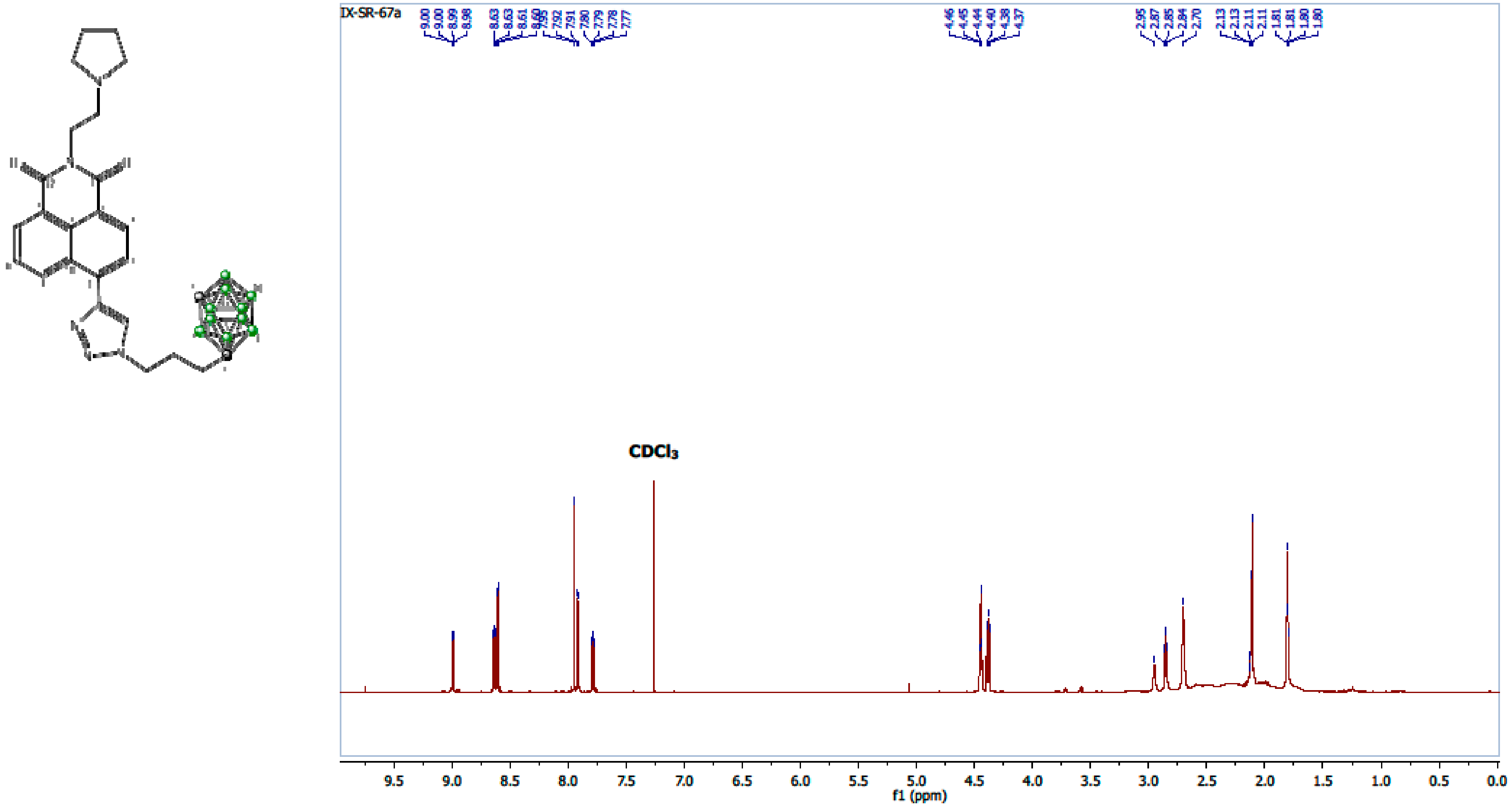

Figure S38. ^{1}H NMR spectrum of **11**.

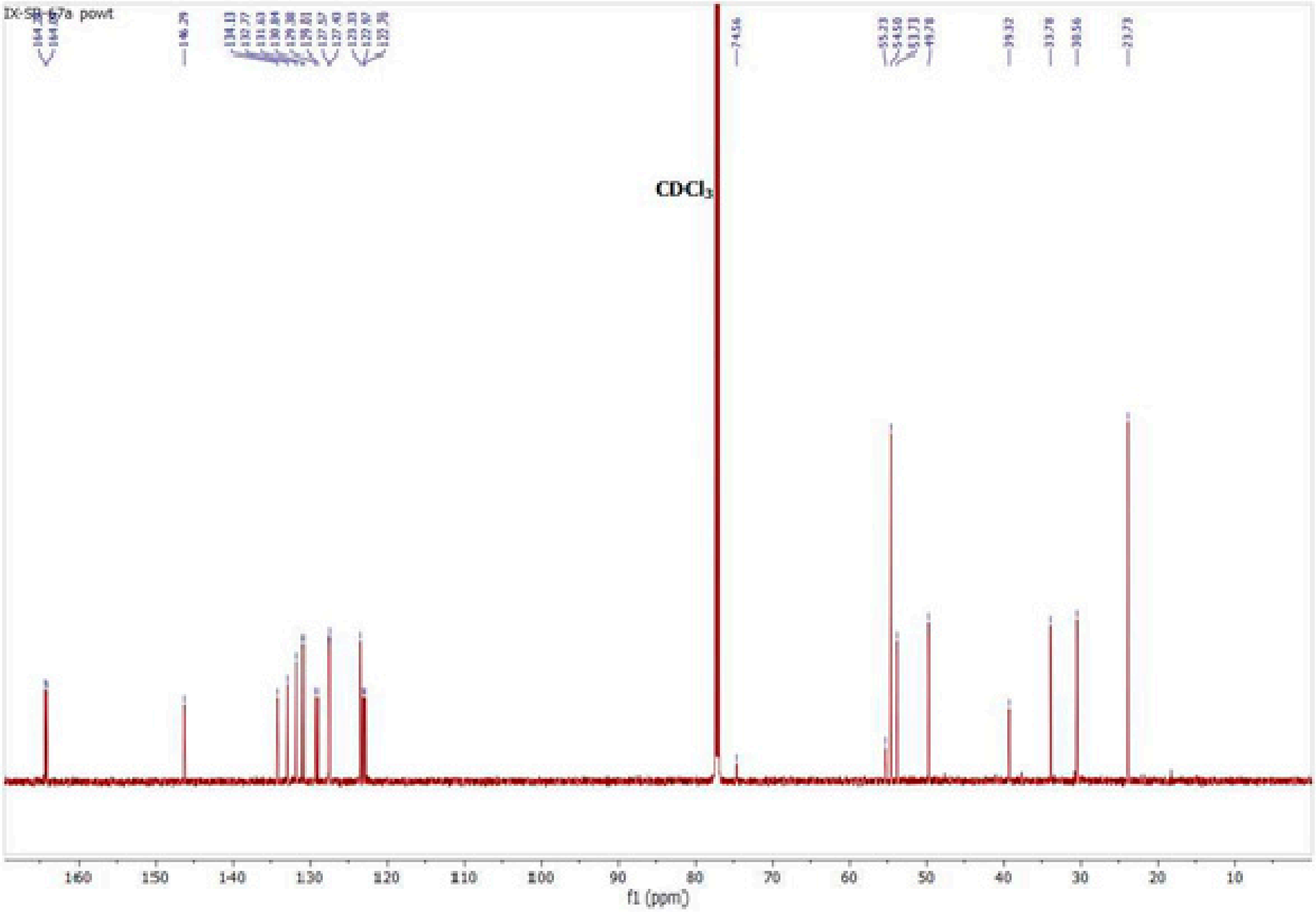

Figure S39. ^{13}C NMR spectrum of **11**.

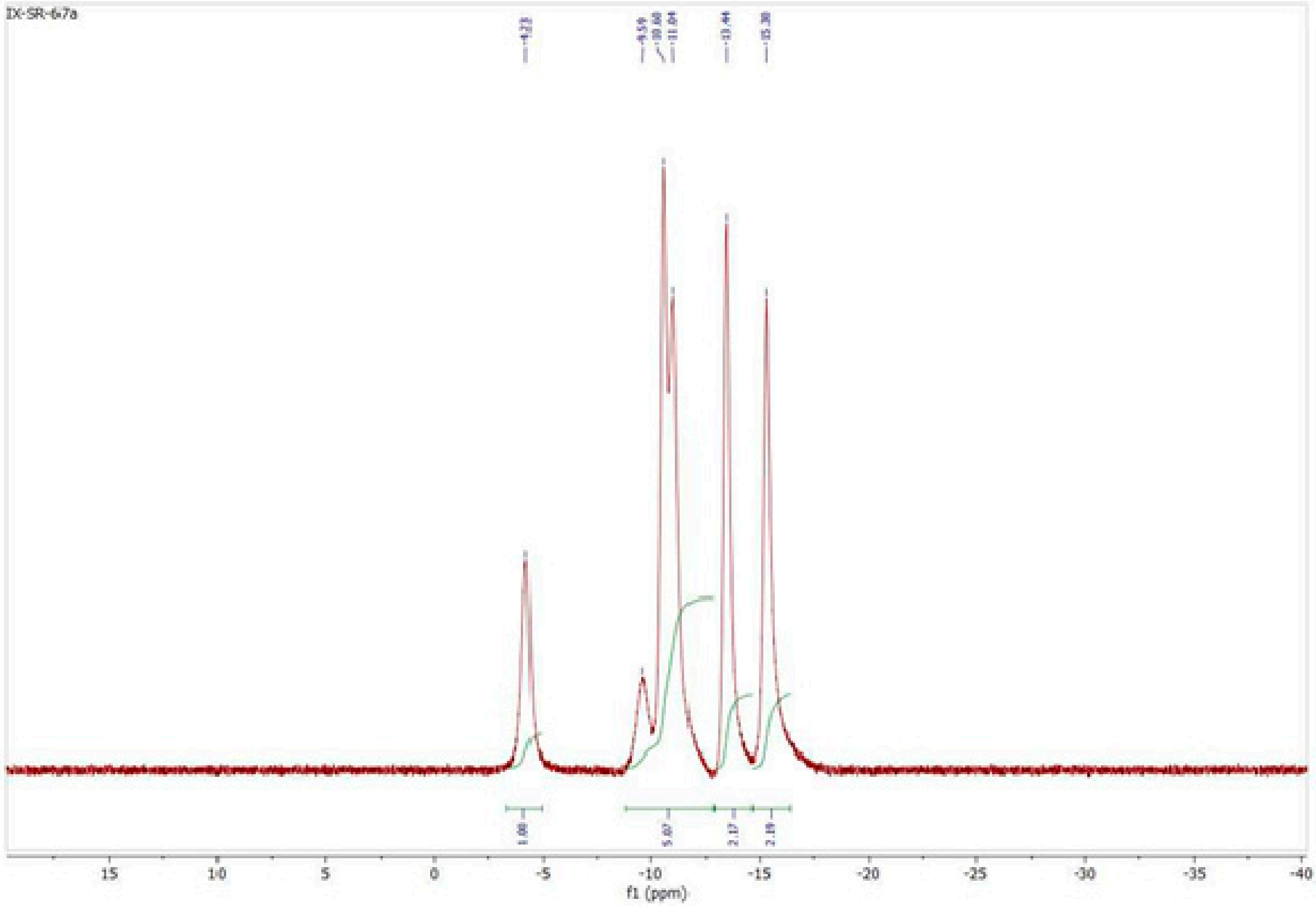

Figure S40. ^{11}B NMR spectrum of **11**.

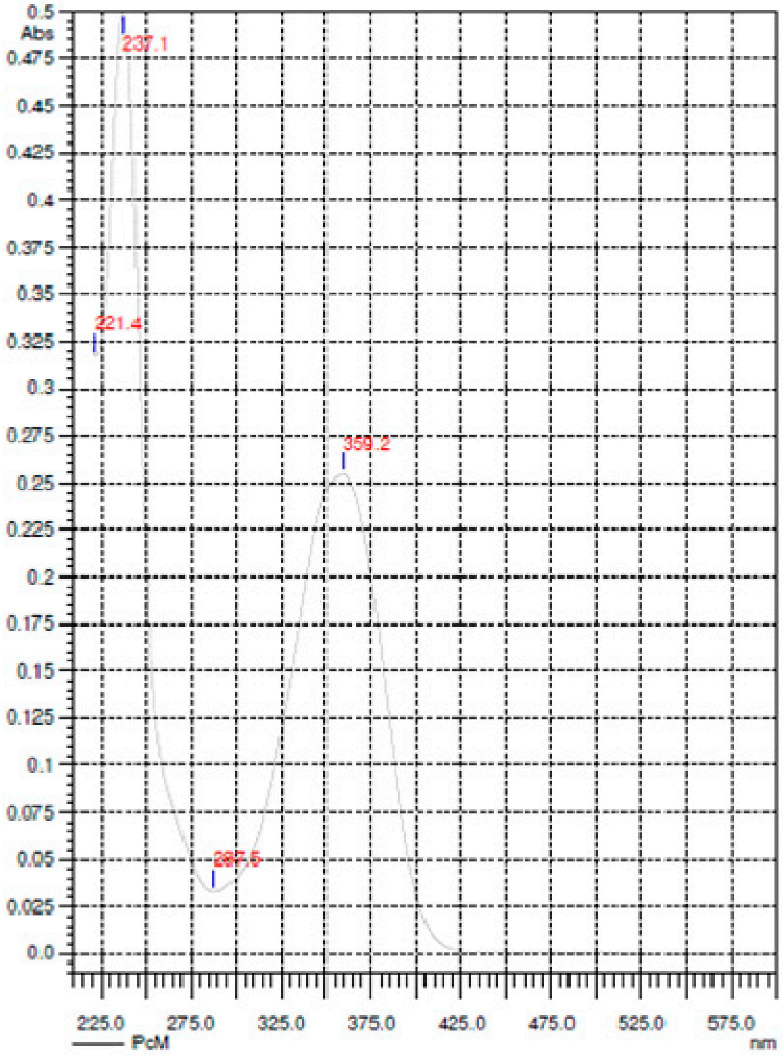

Figure S41. UV spectrum of **11**.

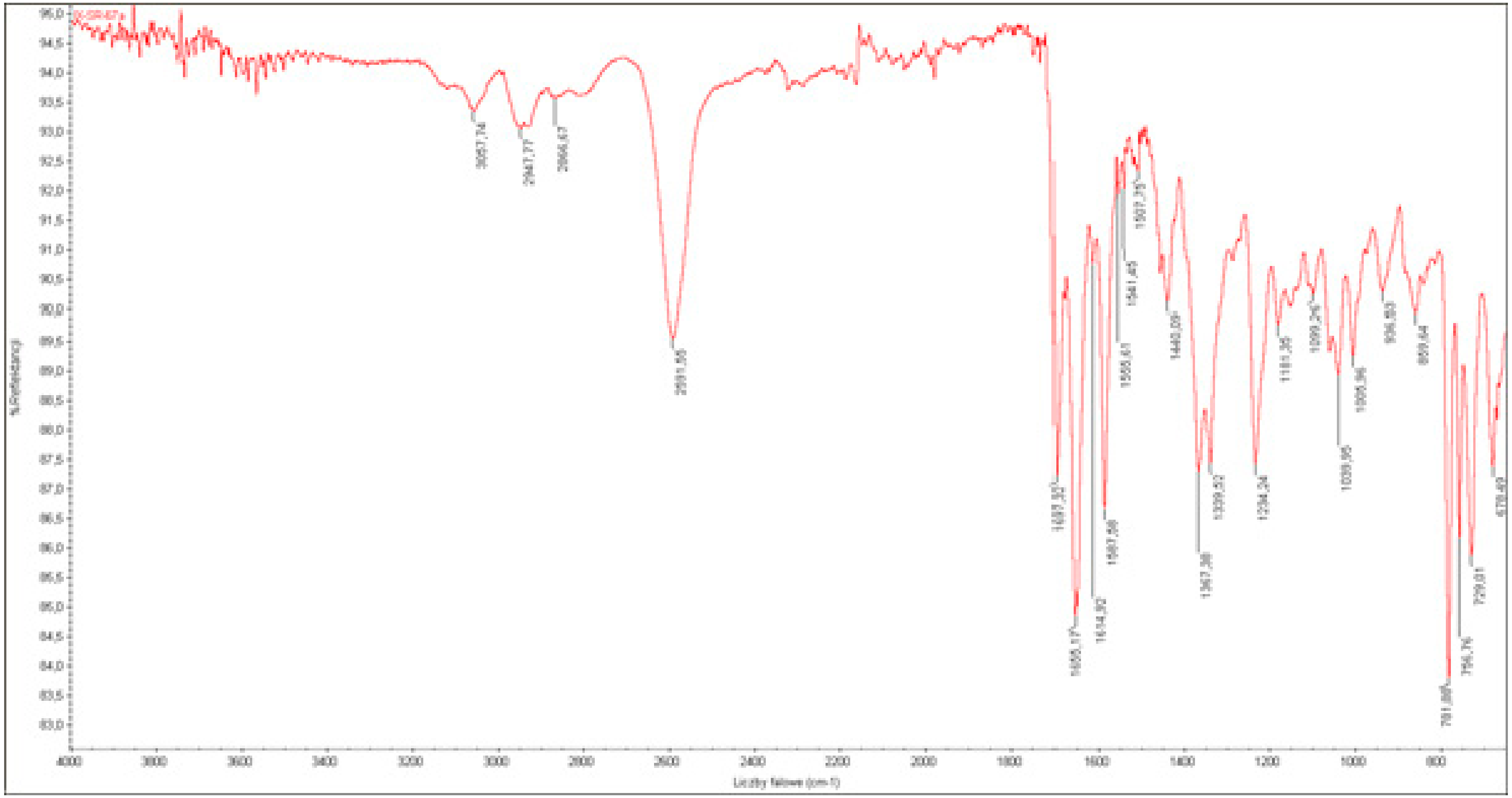

Figure S42. IR spectrum of **11**.

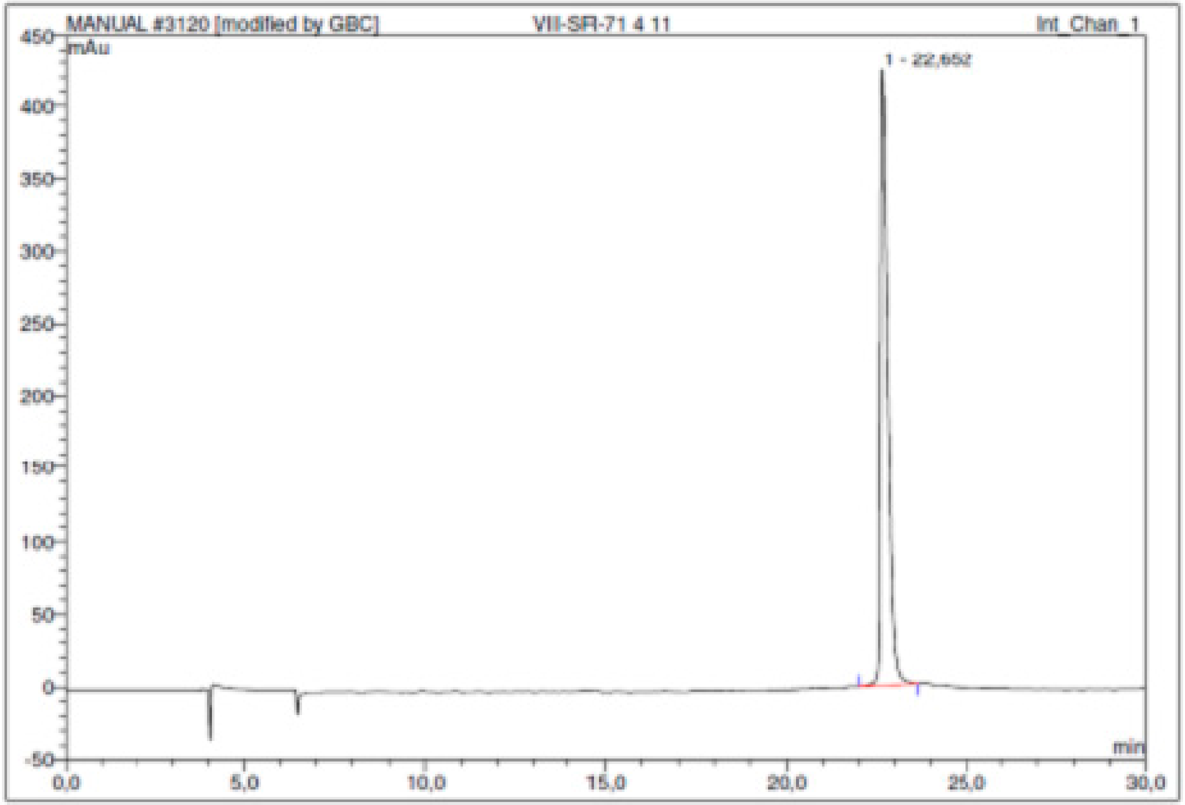

No.	Ret.Time min	Peak Name	Height mAu	Area mAu*min	Rel.Area %	Amount	Type
1	22,65	n.a.	423,990	96,564	100,00	n.a.	BMB
Total:			423,990	96,564	100,00	0,000	

Figure S43. HPLC analysis of **11**.

Spectrum Name: VIII-SR-71_PCM_pt
Start Ion: 100
End Ion: 700
Source: APCI + 10.0µA 400C
Capillary: 150V 300C Offset: 25V Span: 0V

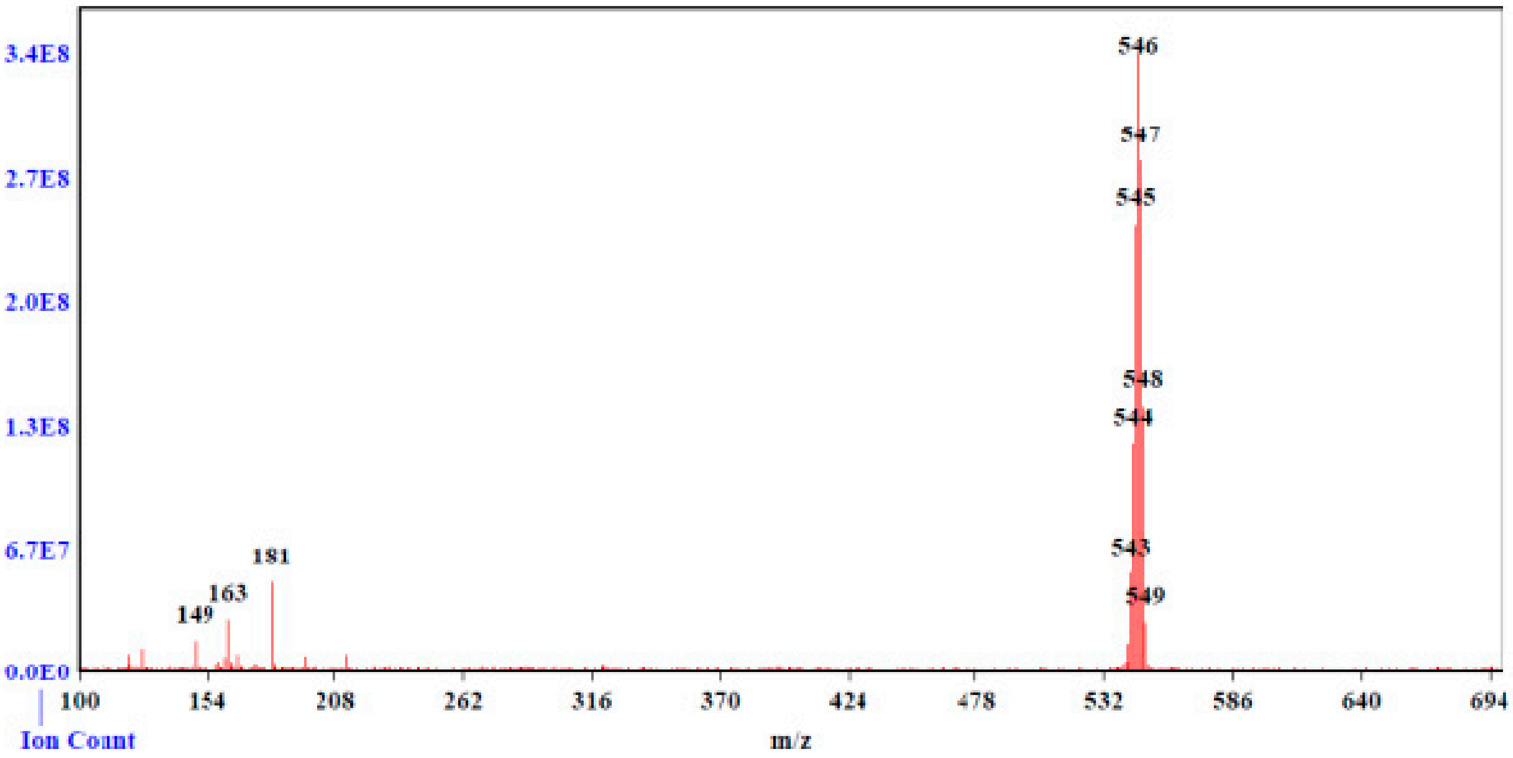

Figure S44. MS spectrum of **11**.

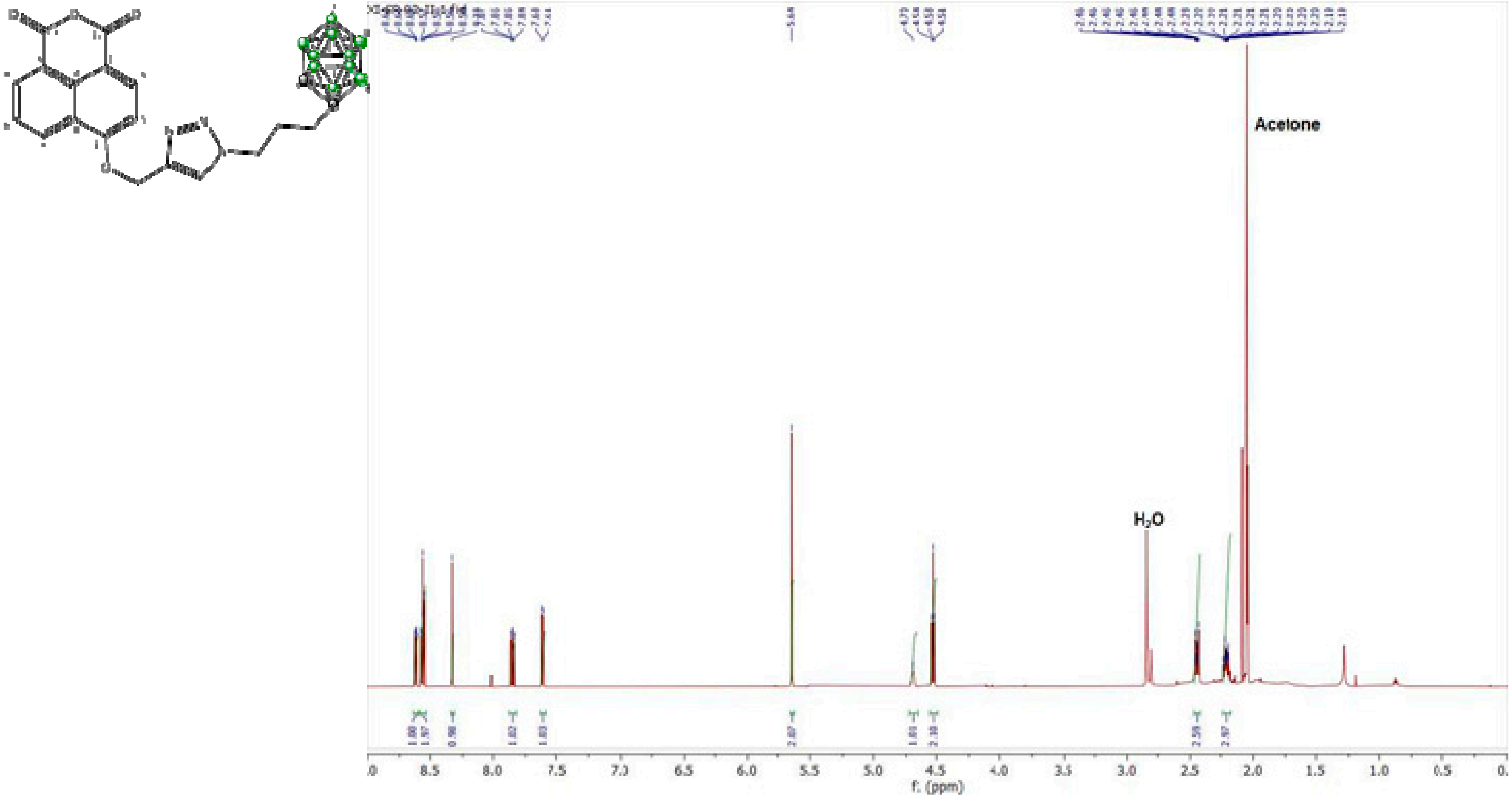

Figure S45. ^{1}H NMR spectrum of **14**.

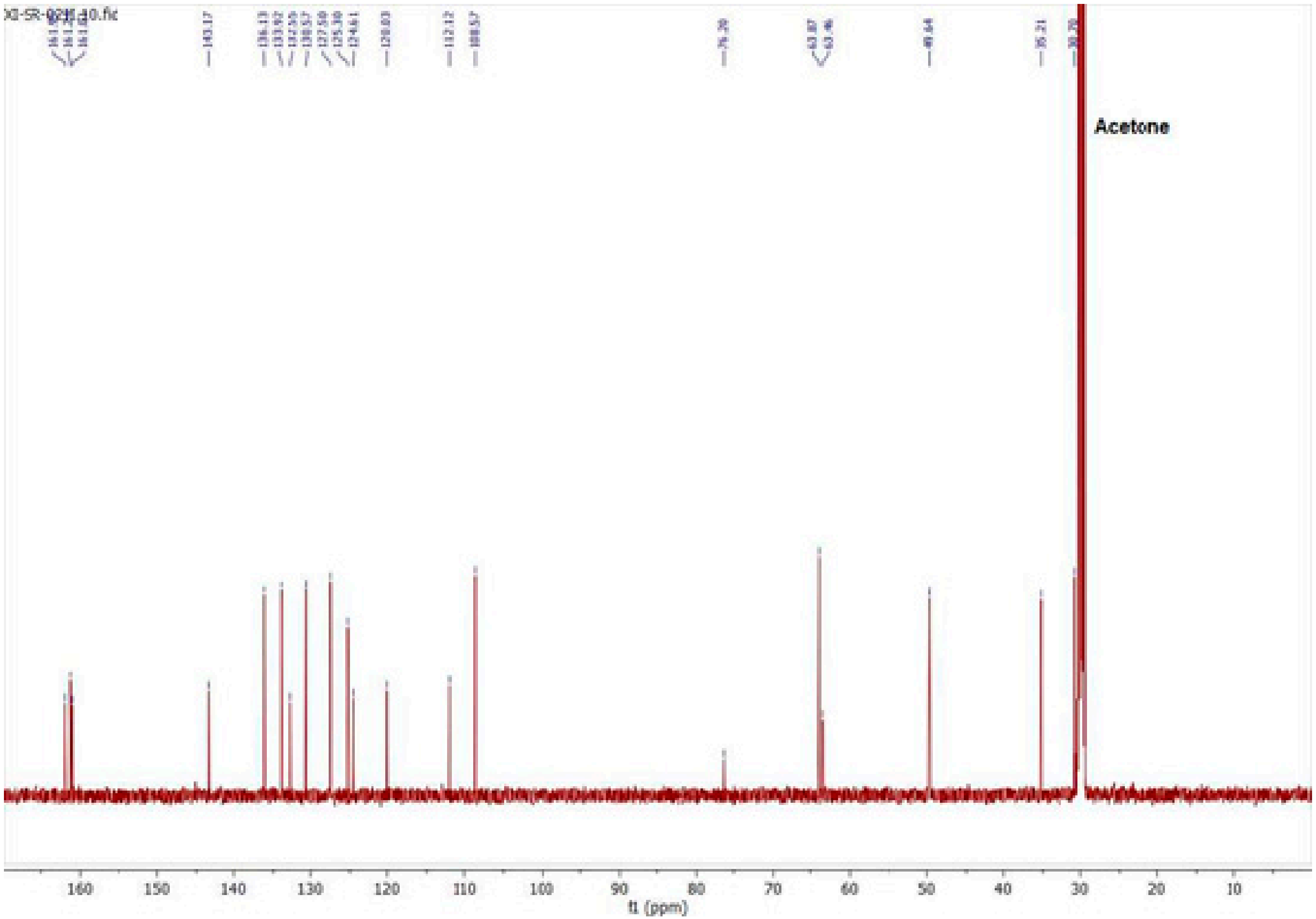

Figure S46. ^{13}C NMR spectrum of **14**.

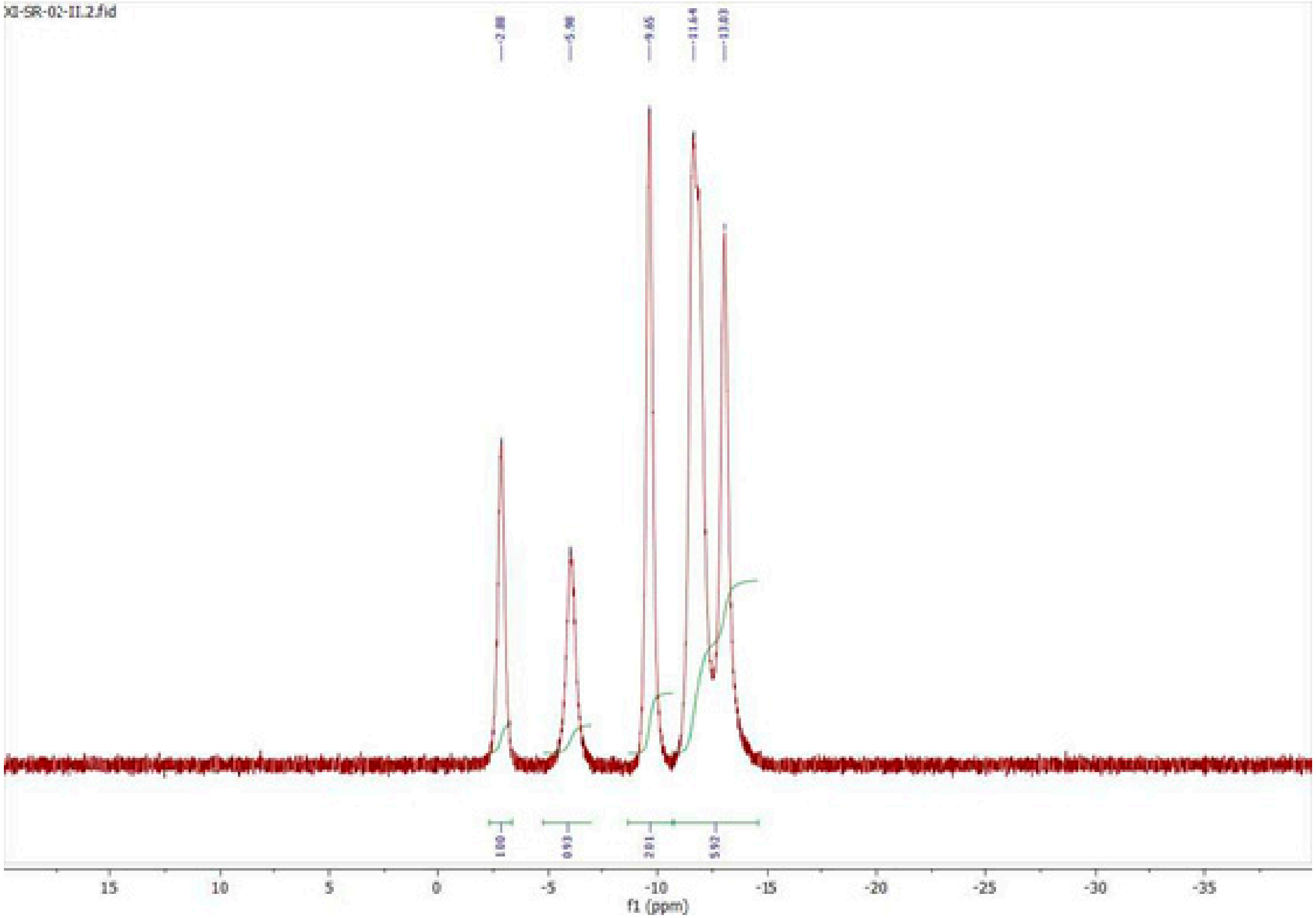

Figure S47. ^{11}B NMR spectrum of **14**.

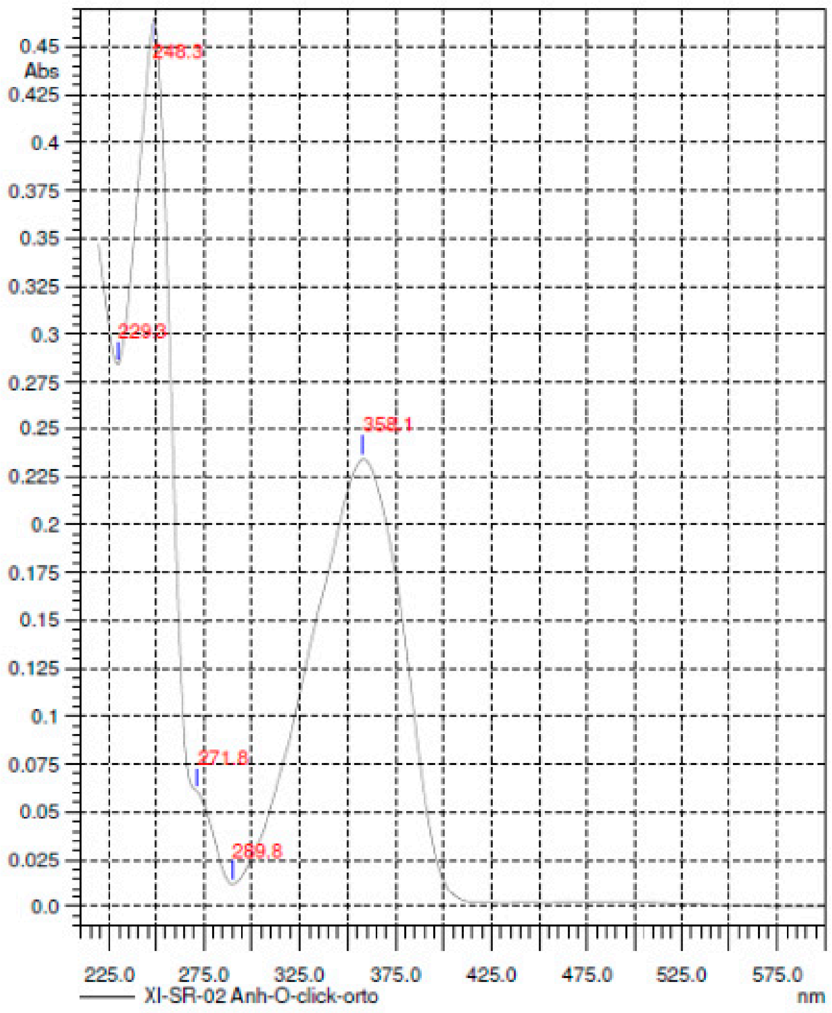

Figure S48. UV spectrum of **14**.

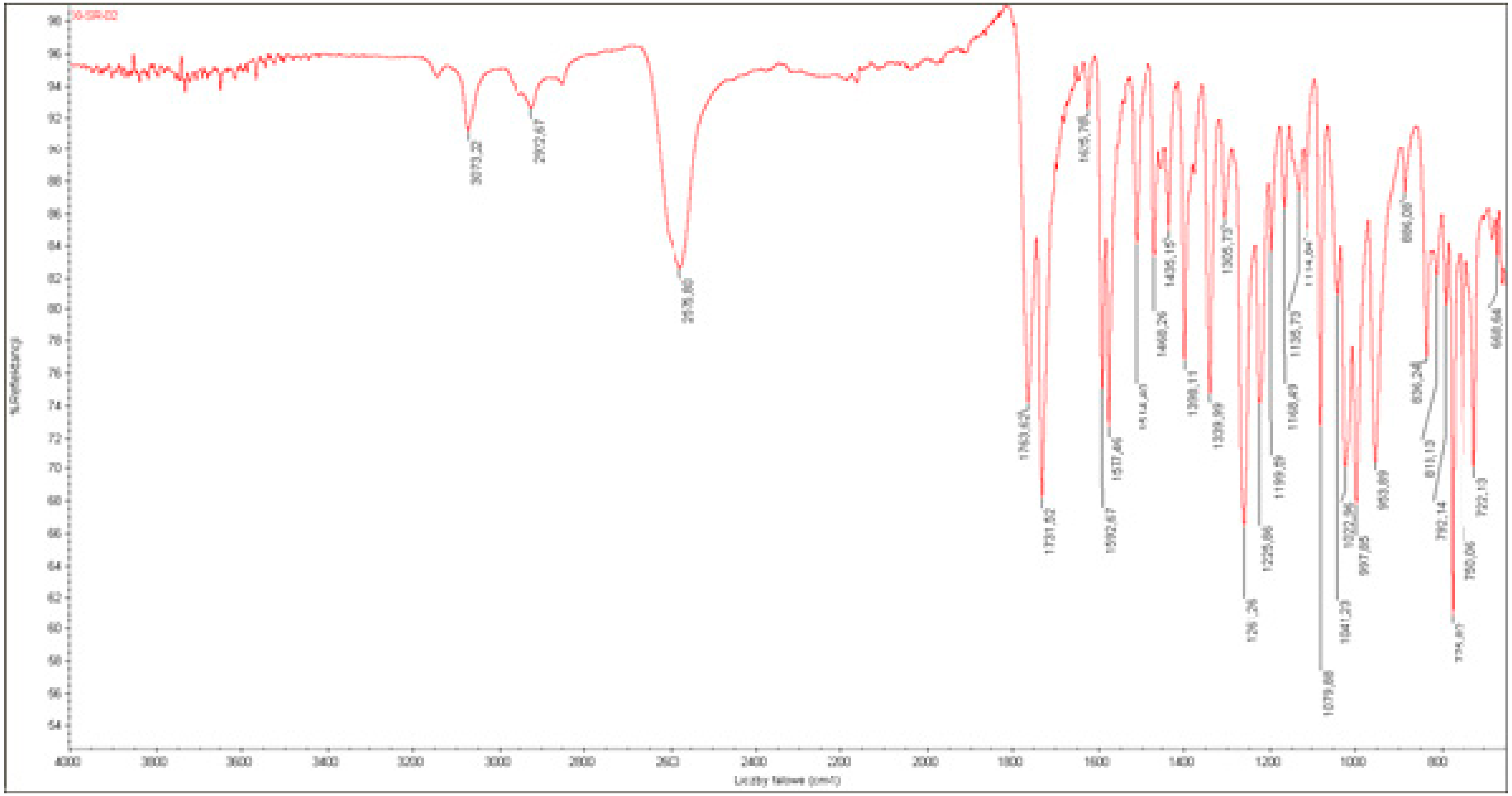

Figure S49. IR spectrum of **14**.

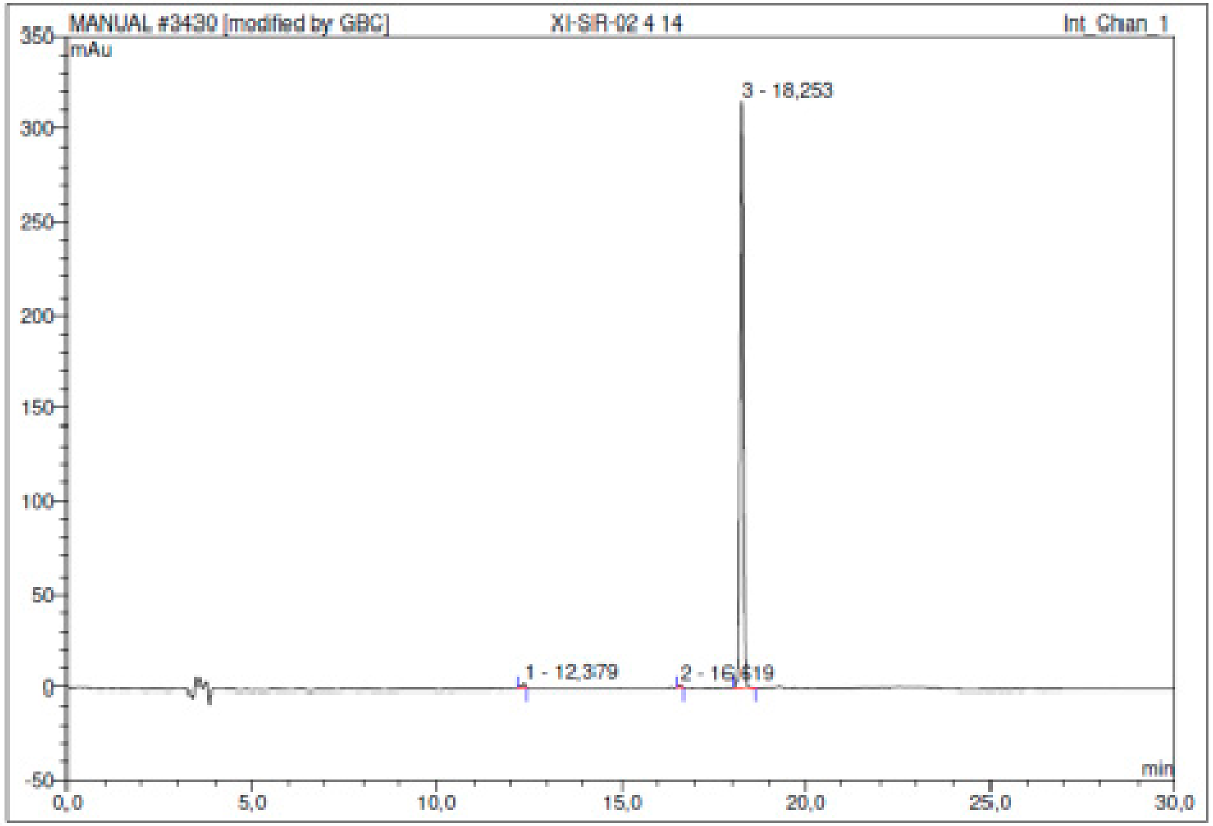

No.	Ret.Time min	Peak Name	Height mAu	Area mAu*min	Rel.Area %	Amount	Type
1	12,38	n.a.	2,257	0,268	0,82	n.a.	BMB*
2	16,62	n.a.	1,321	0,112	0,34	n.a.	BMB*
3	18,25	n.a.	315,772	32,305	98,84	n.a.	BMB
Total:			319,349	32,686	100,00	0,000	

Figure S50. HPLC analysis of **14**.

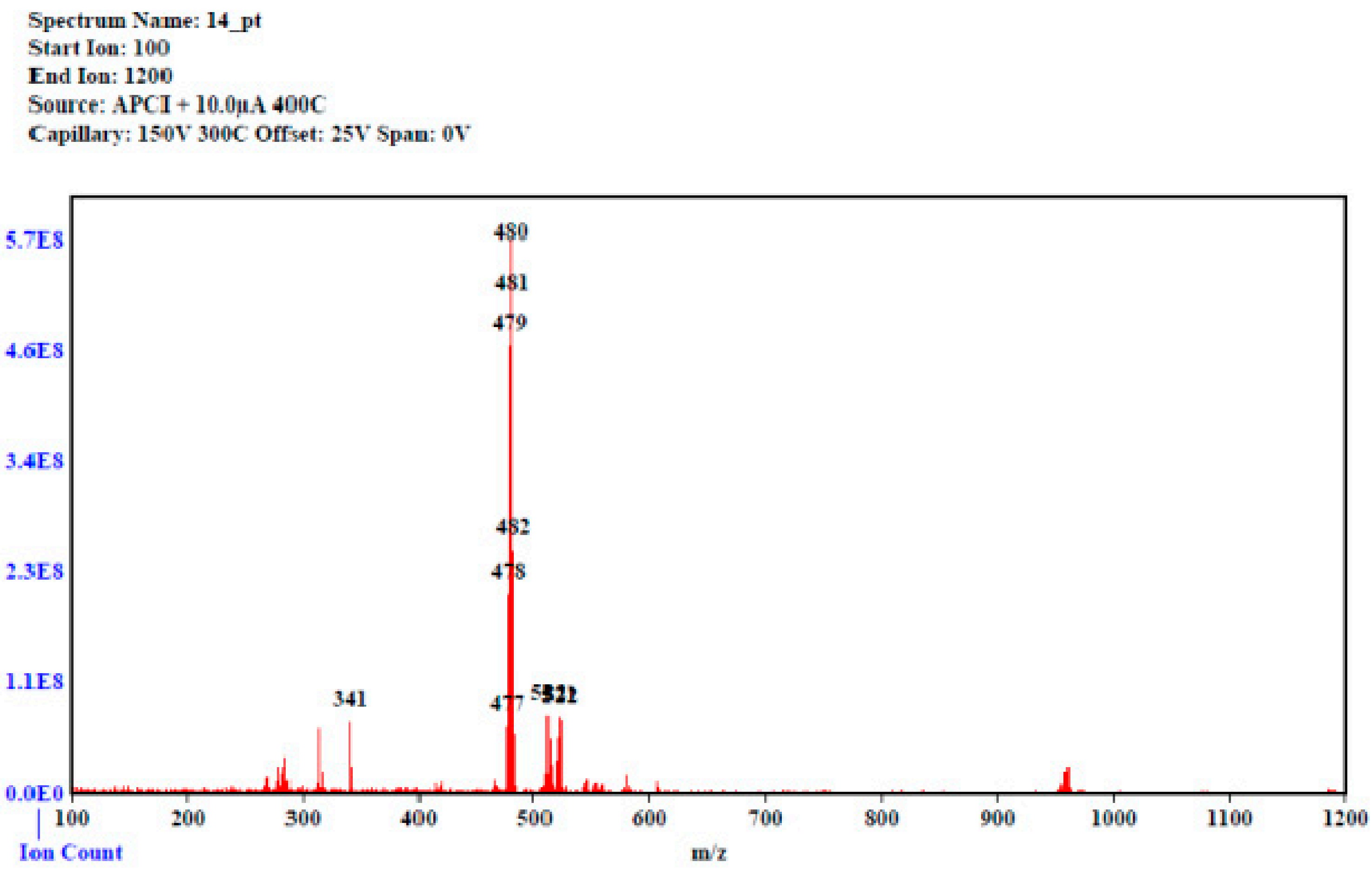

Figure S51. MS spectrum of **14**.

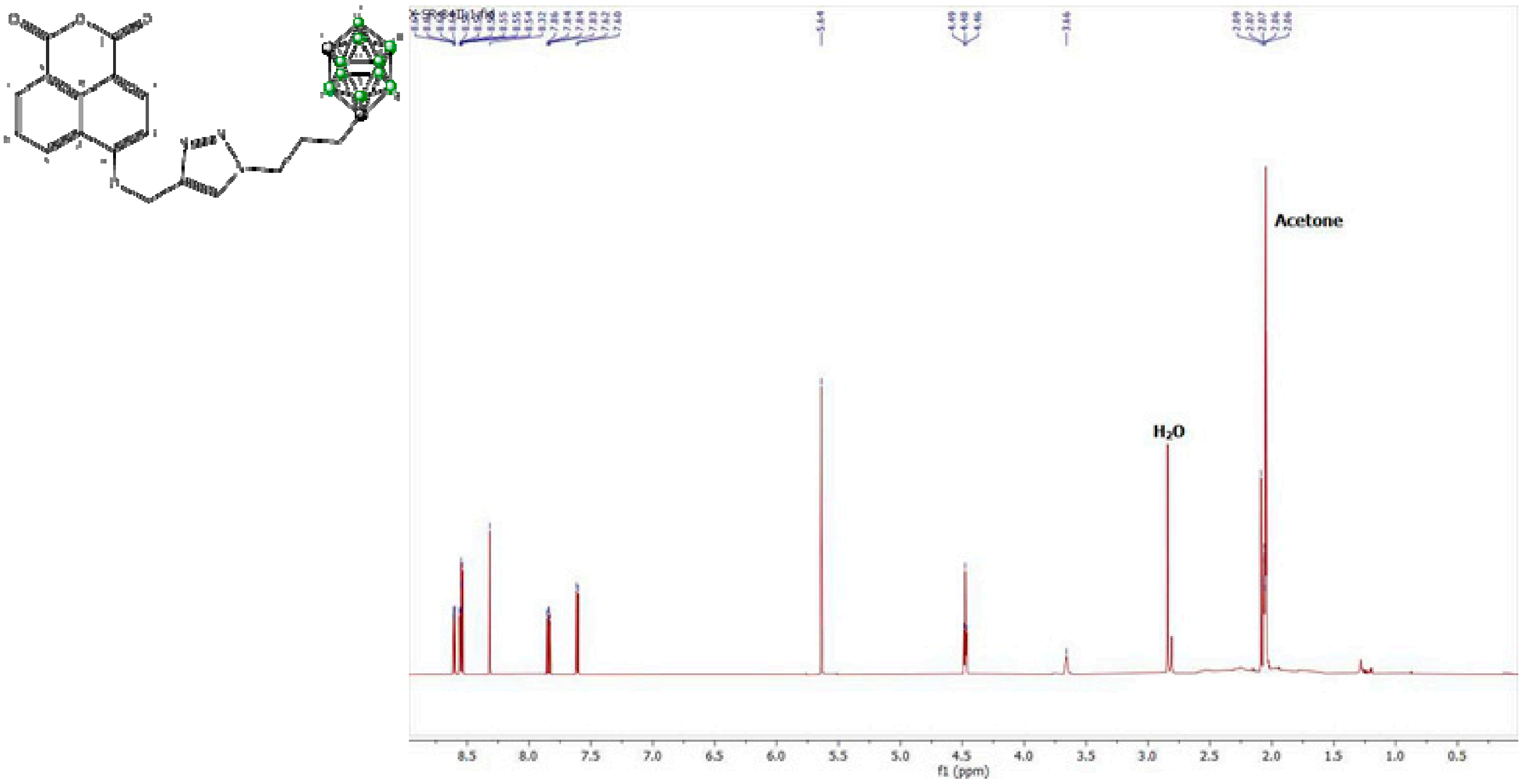

Figure S52. ^{1}H NMR spectrum of **15**.

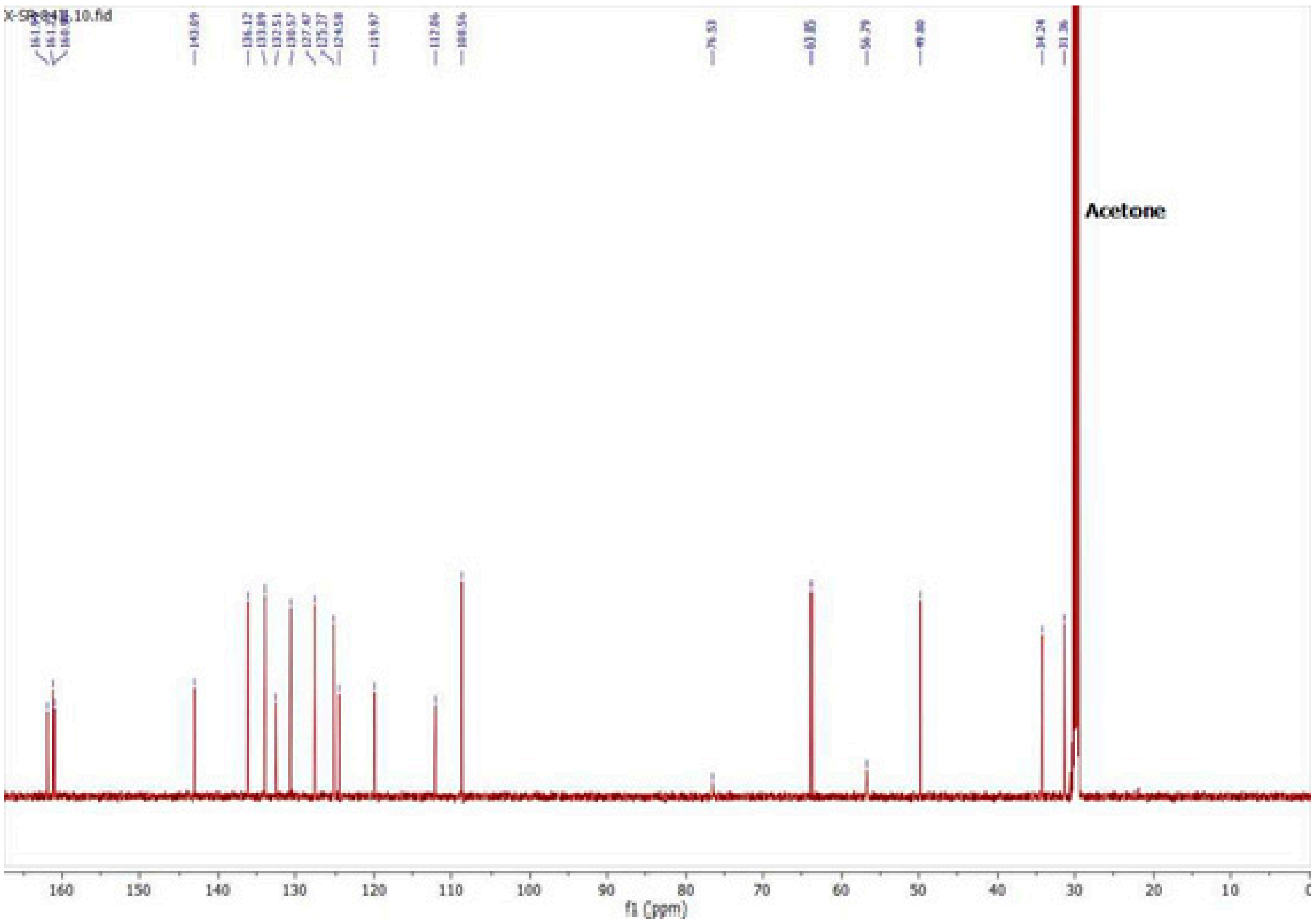

Figure S53. ^{13}C NMR spectrum of **15**.

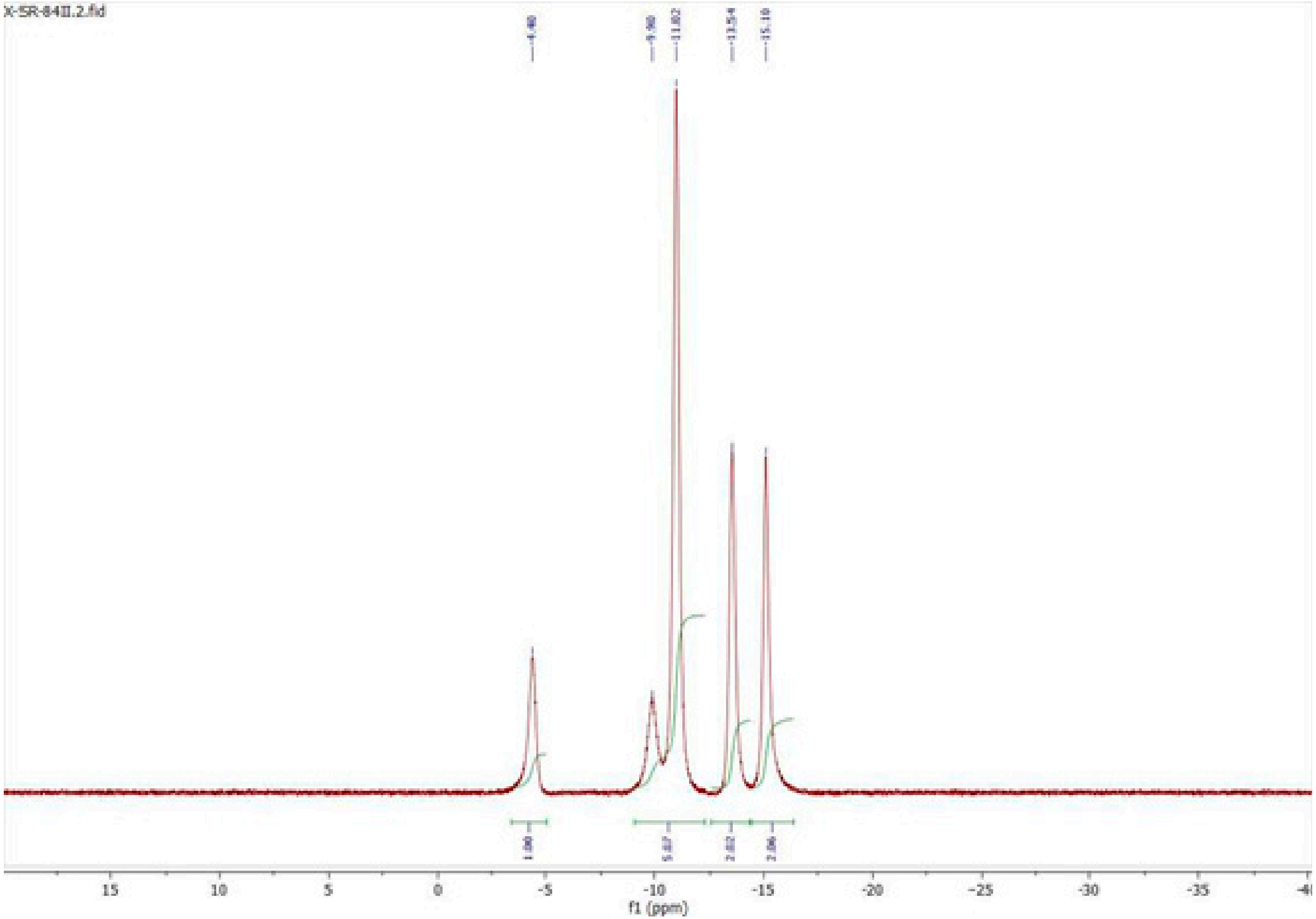

Figure S54. ^{11}B NMR spectrum of **15**.

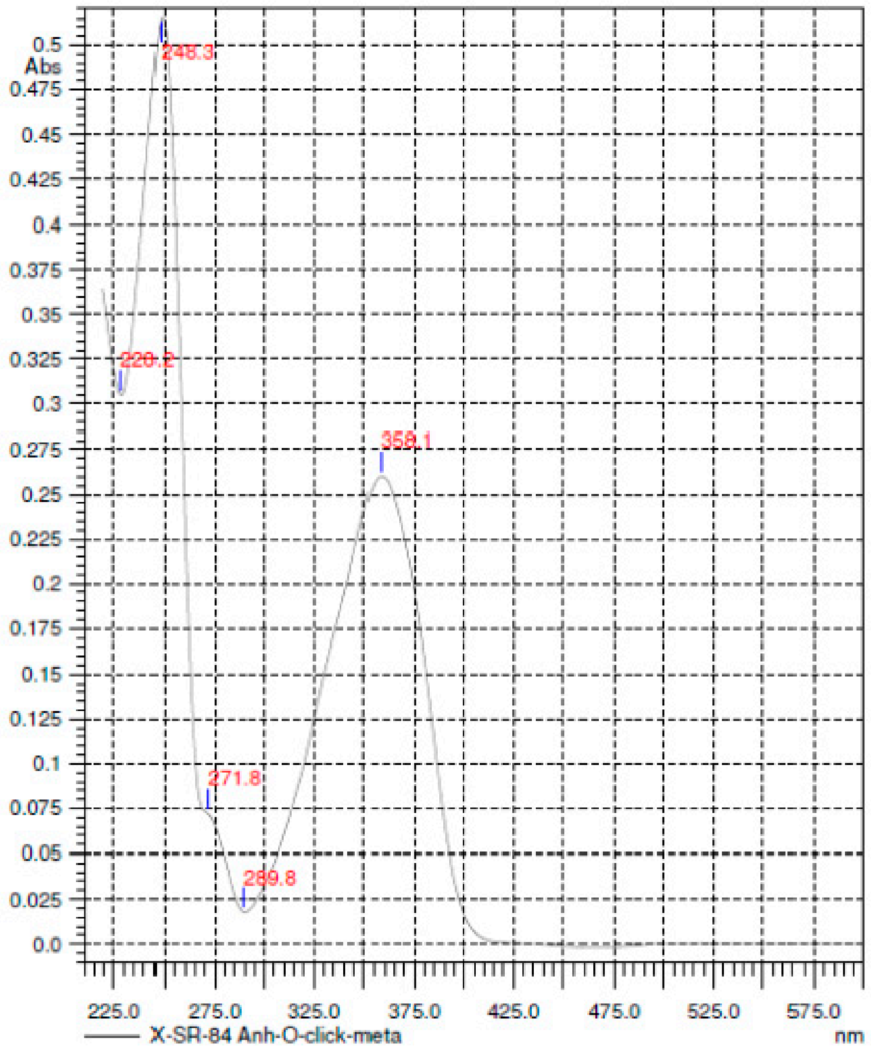

Figure S55. UV spectrum of **15**.

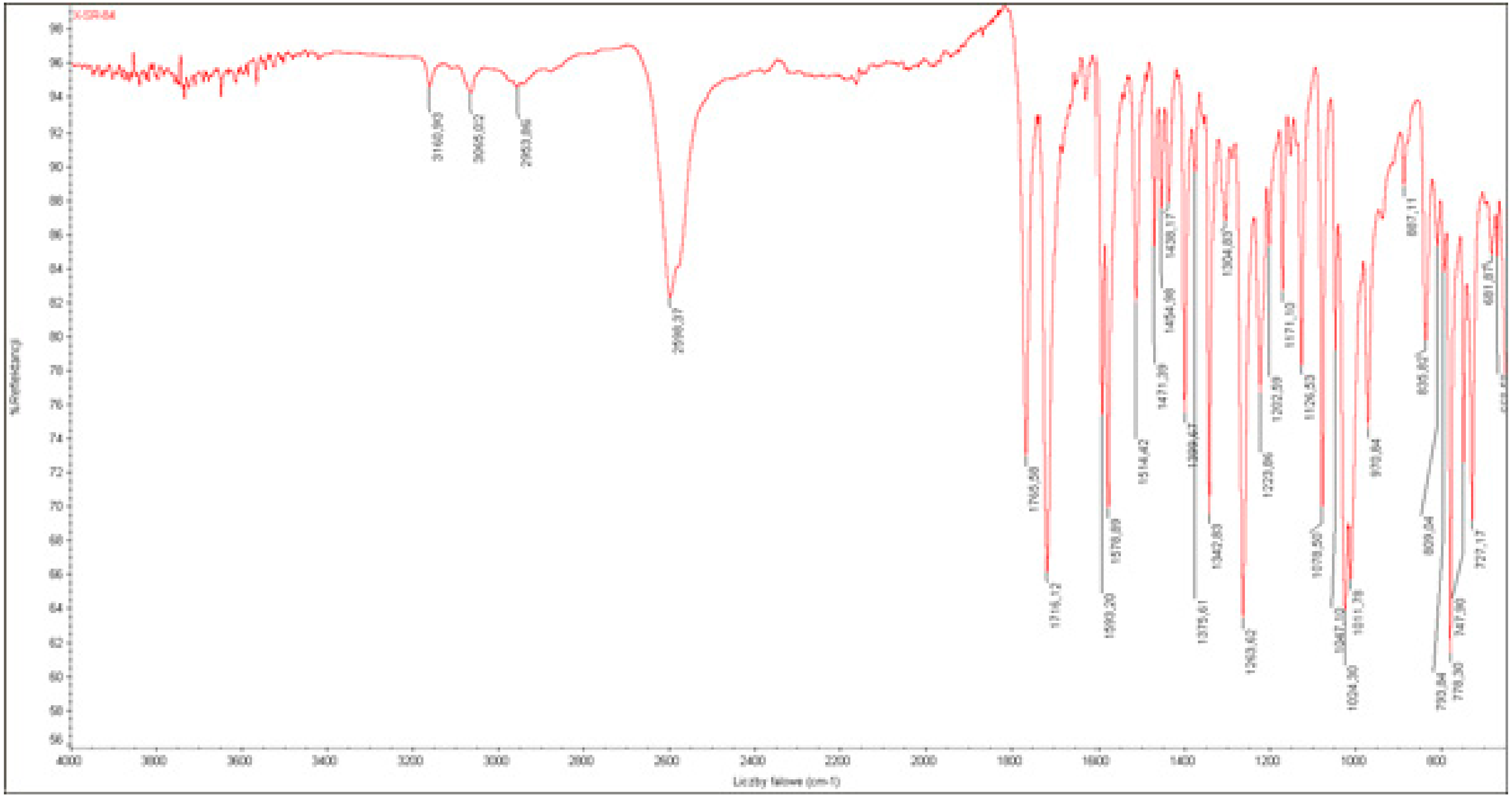

Figure S56. IR spectrum of **15**.

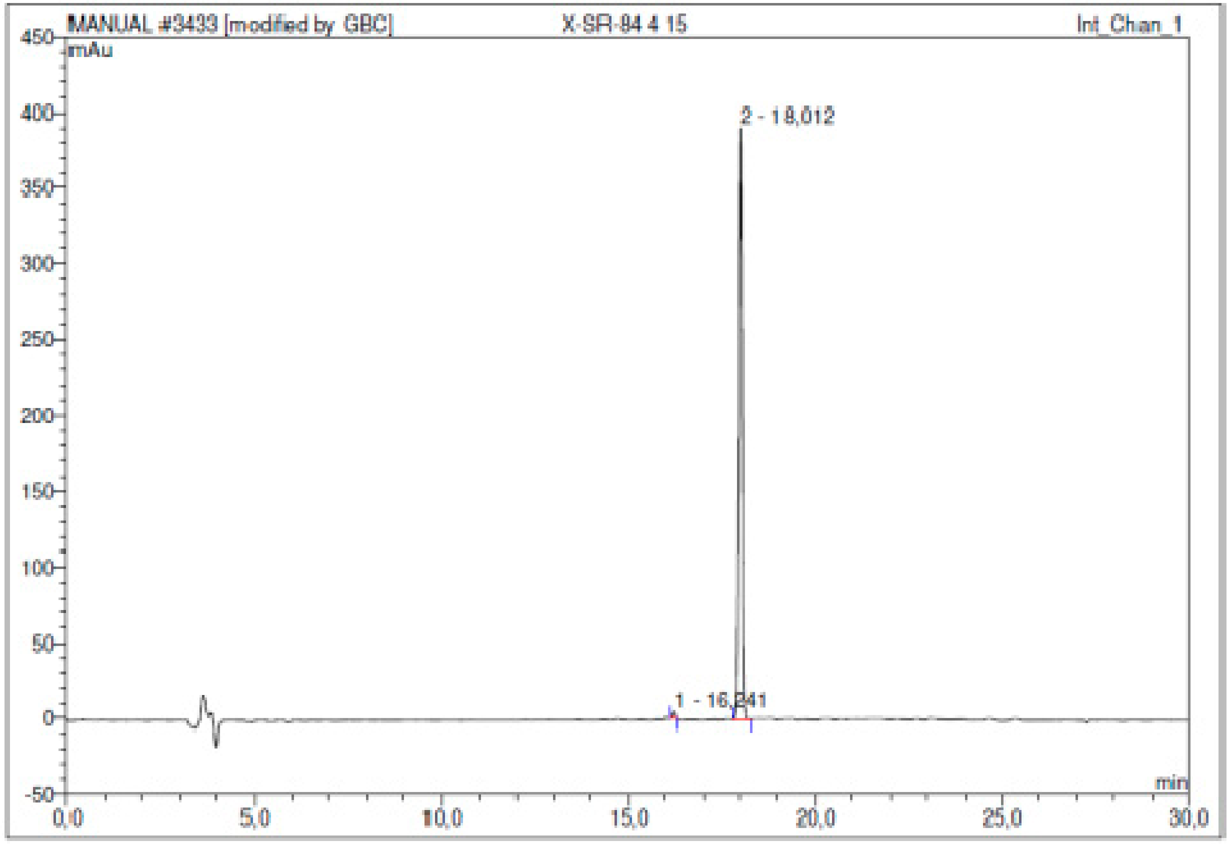

No.	Ret.Time min	Peak Name	Height mAu	Area mAu*min	Rel.Area %	Amount	Type
1	16,24	n.a.	4,021	0,343	0,81	n.a.	BMB*
2	18,01	n.a.	390,326	41,866	99,19	n.a.	BMB
Total:			394,347	42,210	100,00	0,000	

Figure S57. HPLC analysis of **15**.

Spectrum Name: X-SR-84_pt2
Start Ion: 250
End Ion: 600
Source: APCI + 10.0µA 400C
Capillary: 150V 300C Offset: 25V Span: 0V

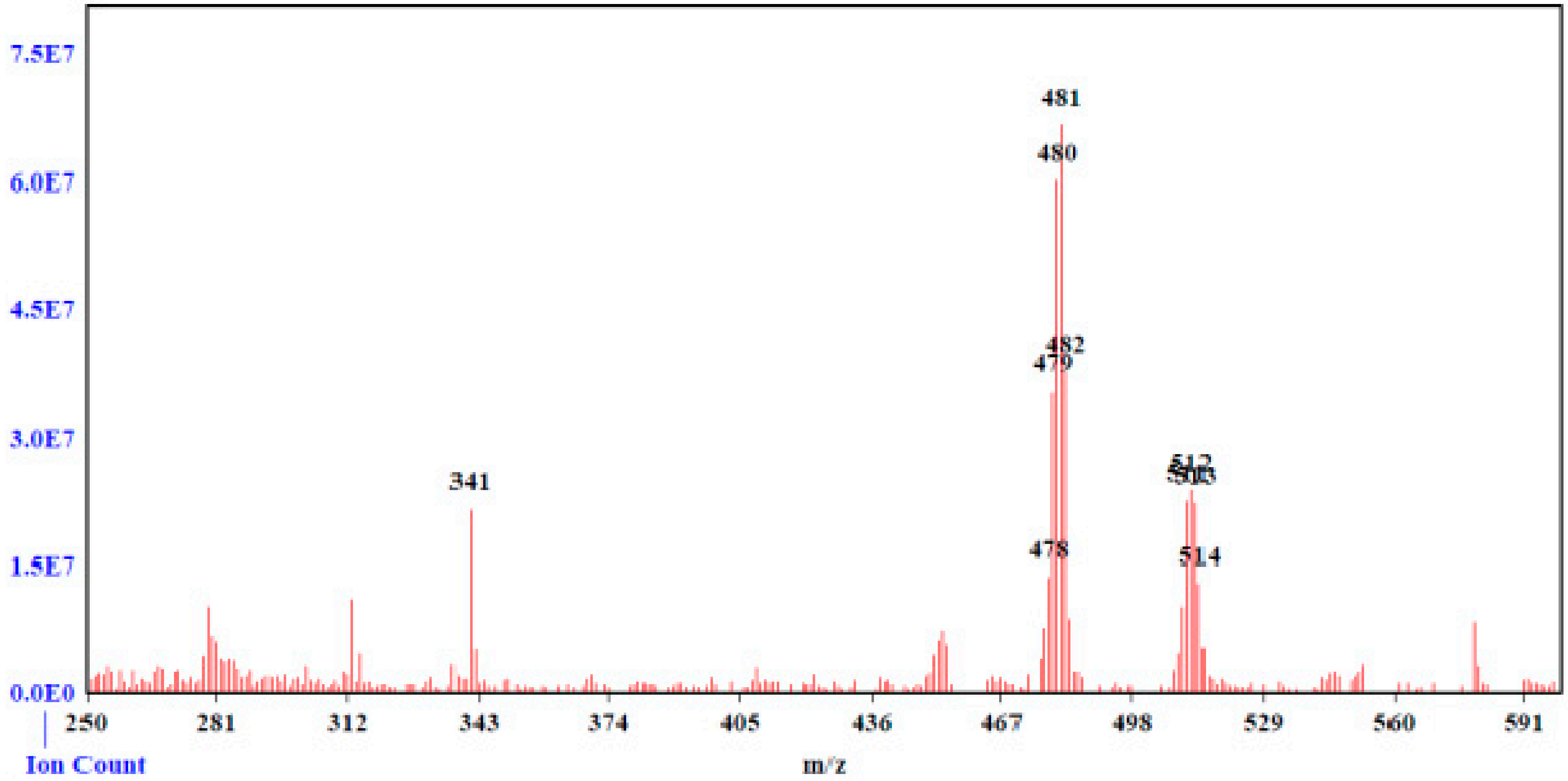

Figure S58. MS spectrum of **15**.

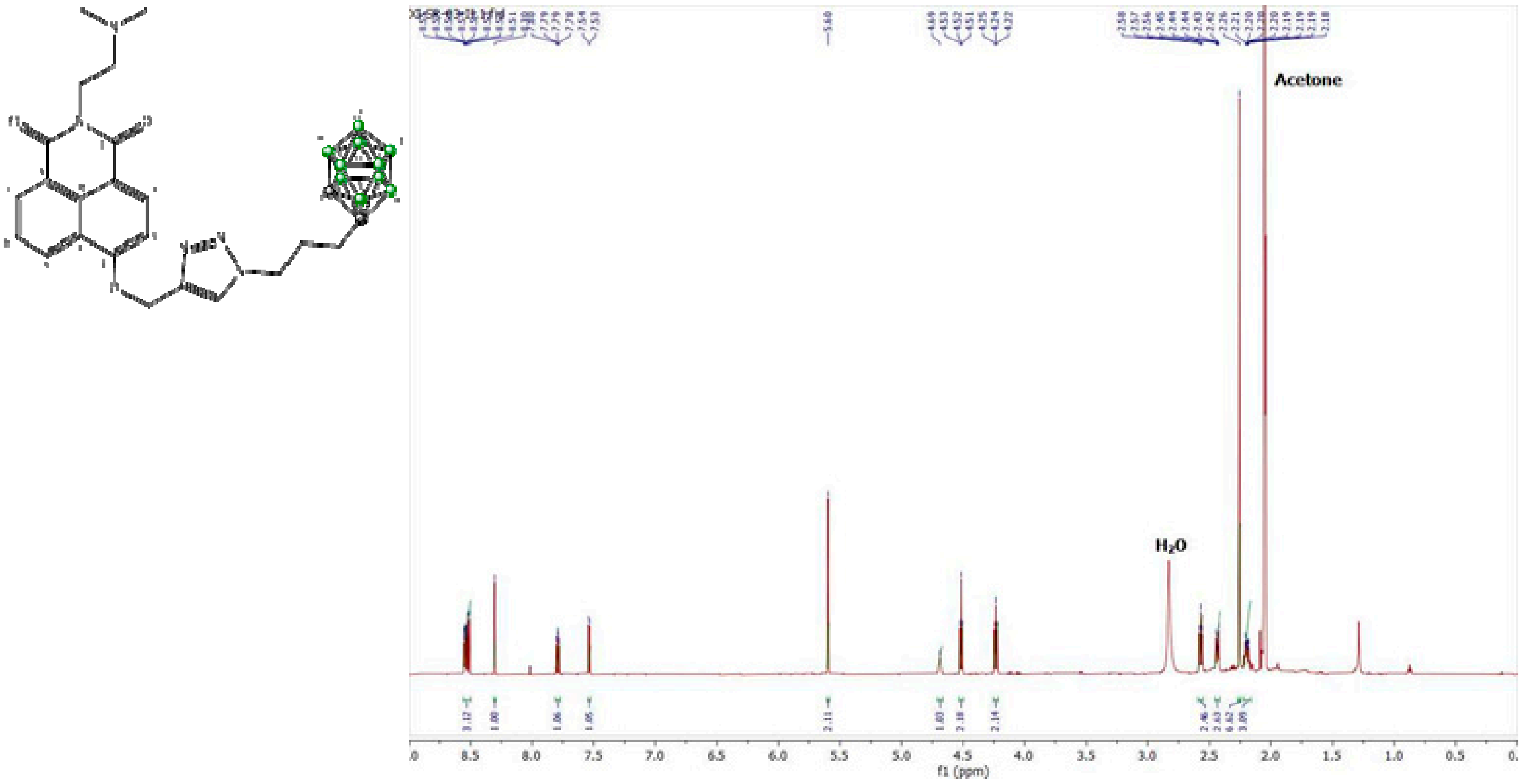

Figure S59. ^{1}H NMR spectrum of **16**.

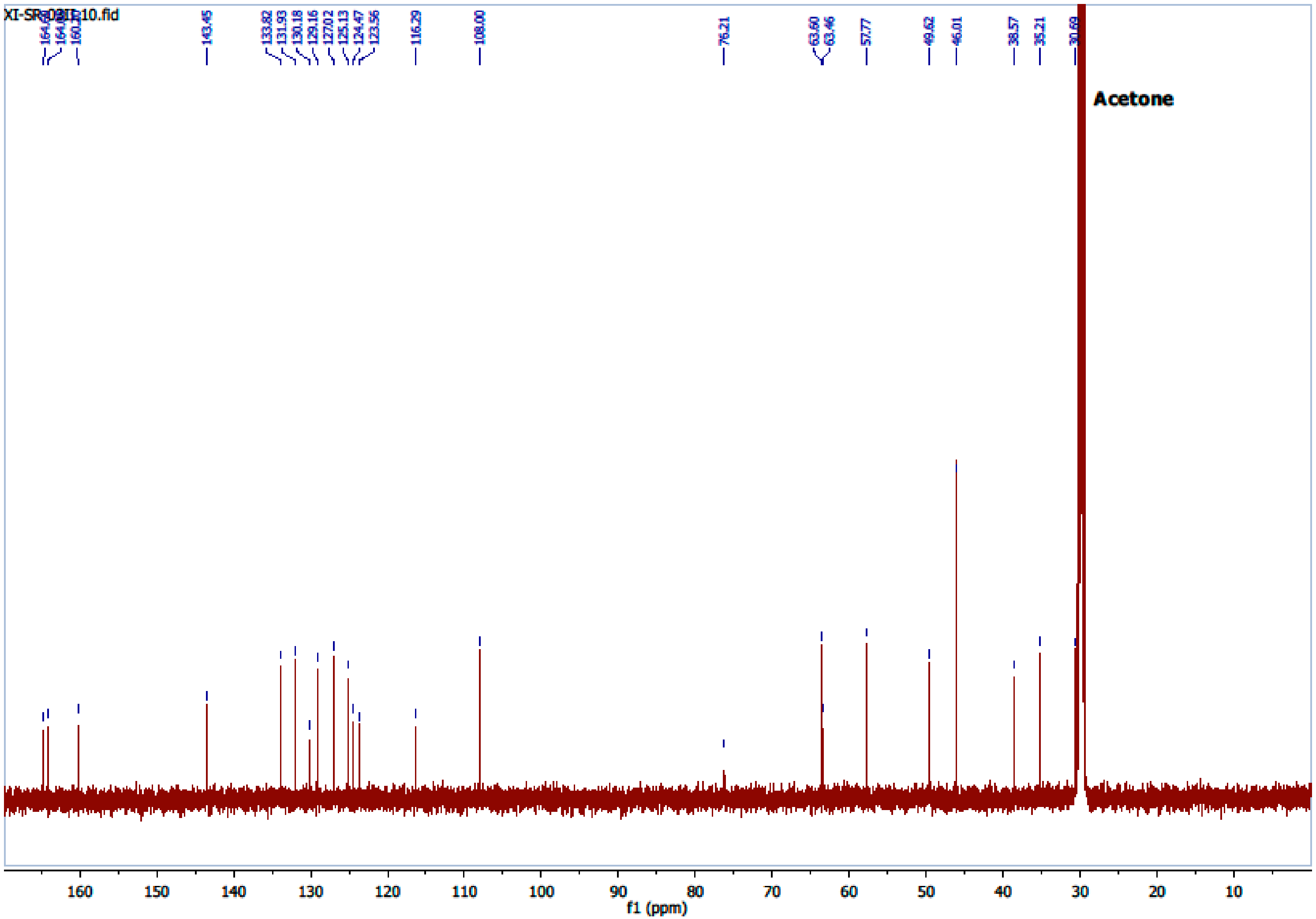

Figure S60. ^{13}C NMR spectrum of **16**.

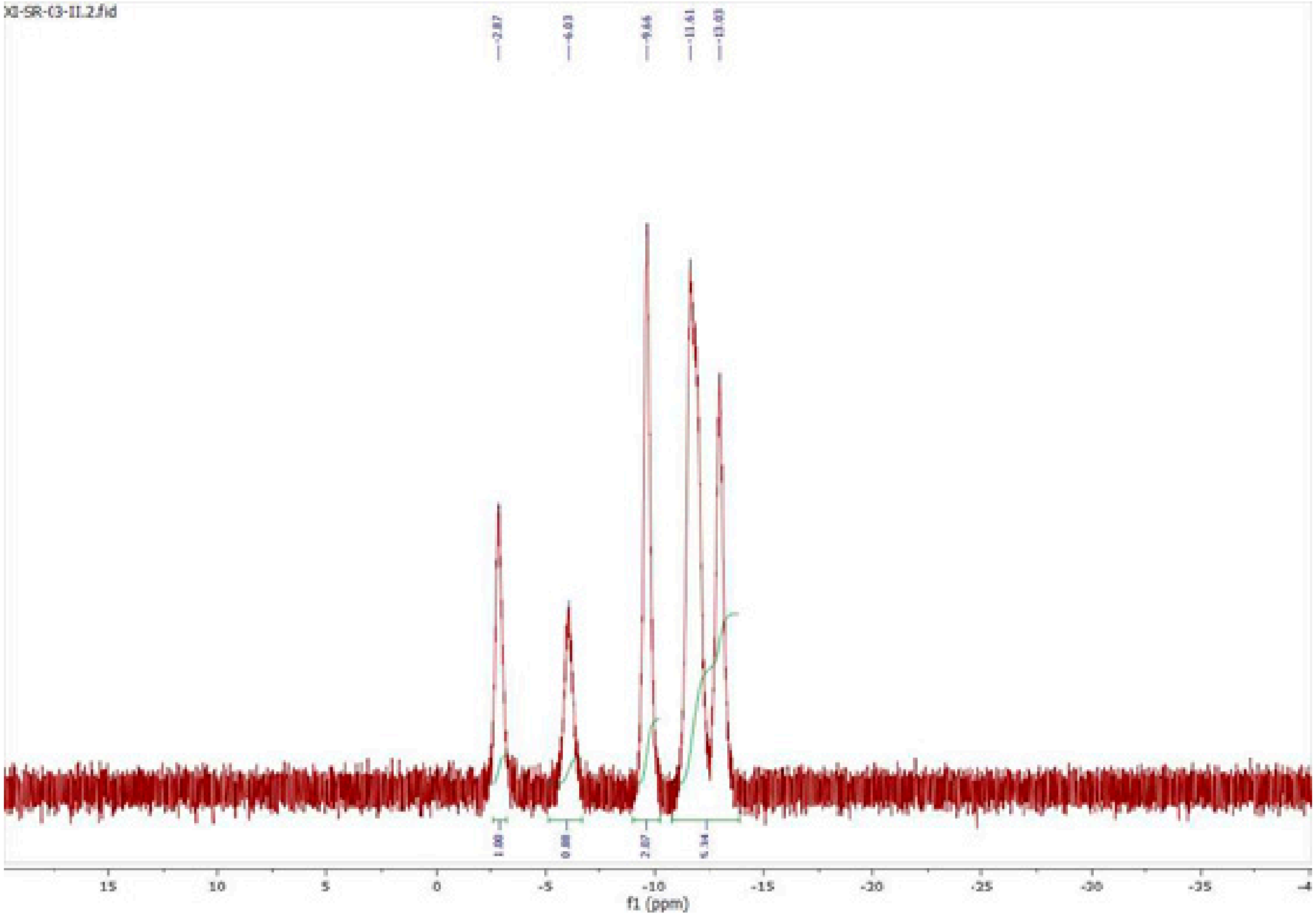

Figure S61. ^{11}B NMR spectrum of **16**.

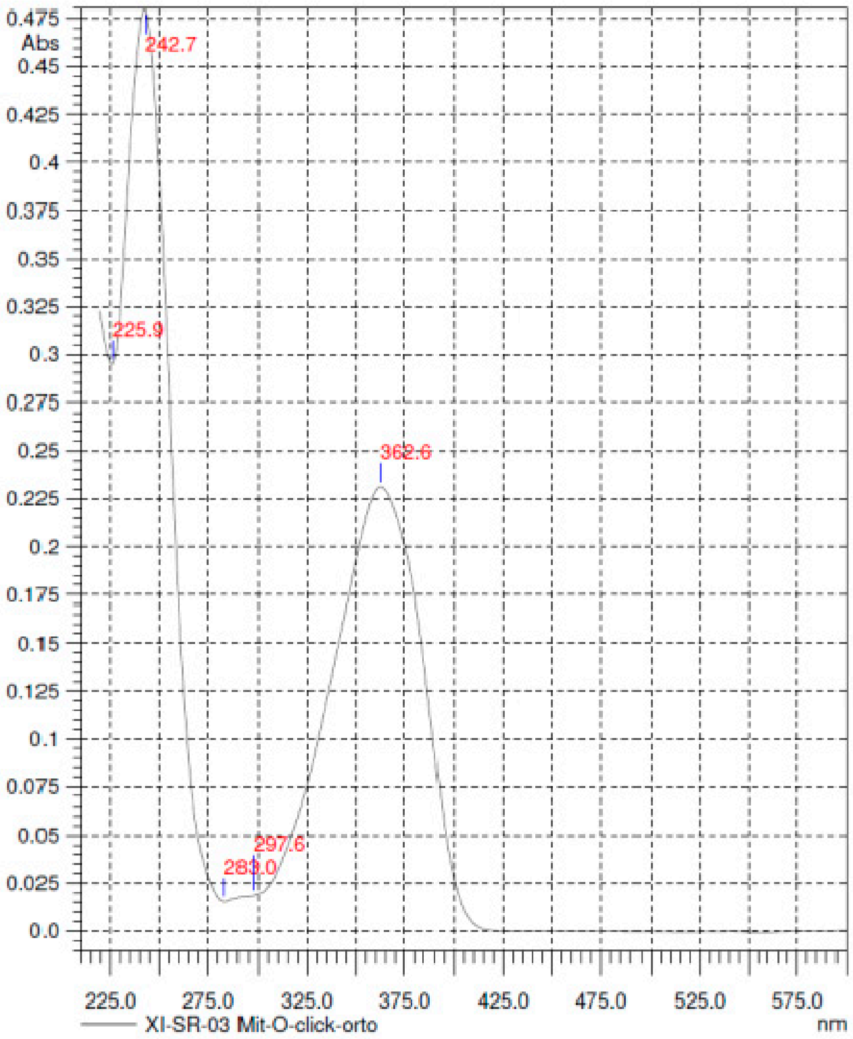

Figure S62. UV spectrum of **16**.

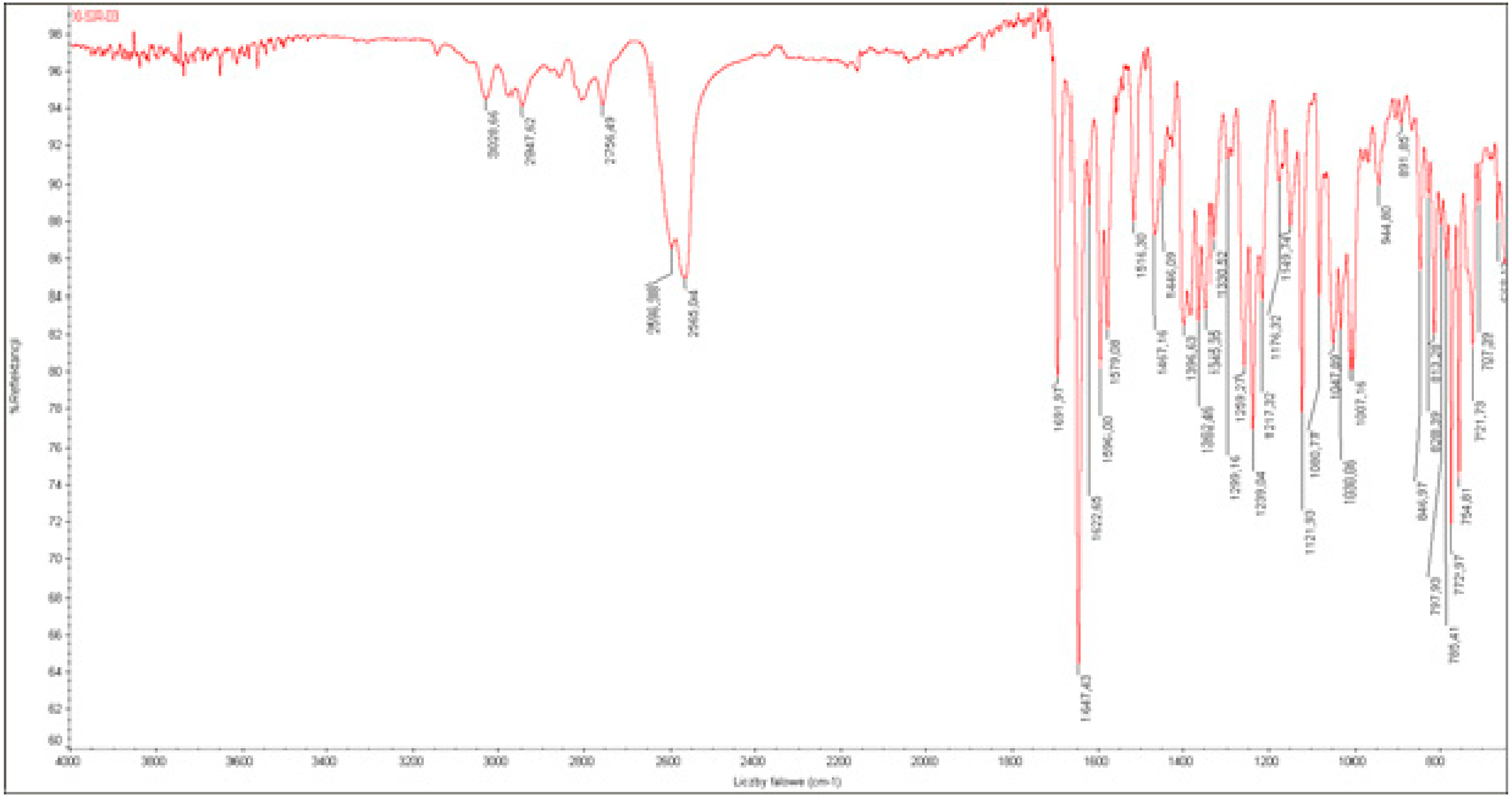

Figure S63. IR spectrum of **16**.

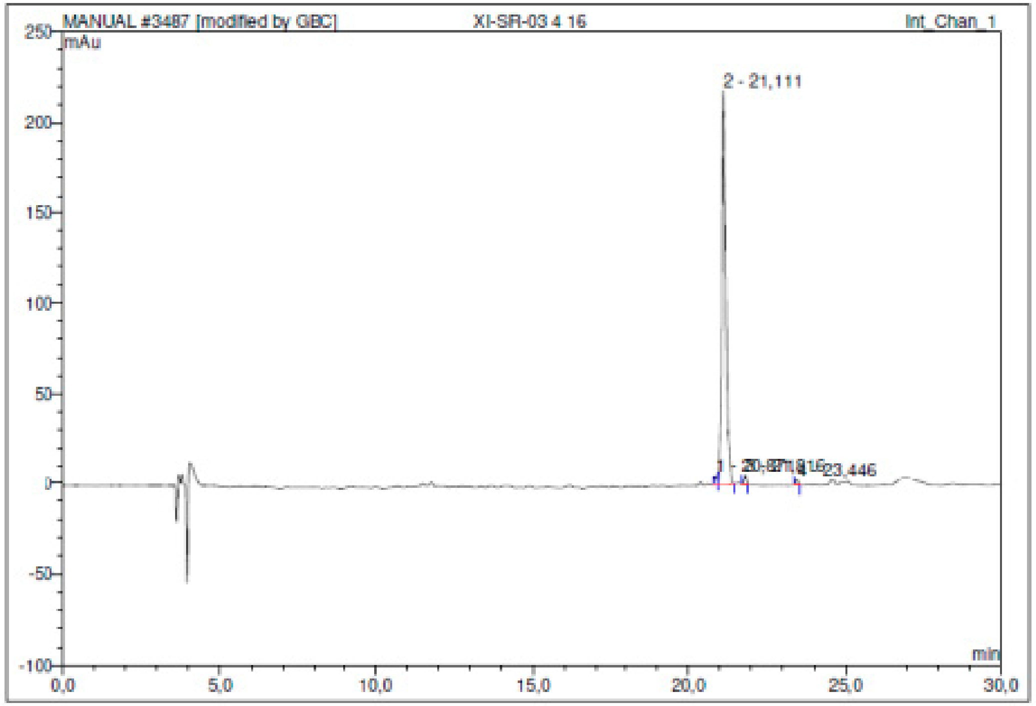

No.	Ret.Time min	Peak Name	Height mAu	Area mAu*min	Rel.Area %	Amount	Type
1	20,87	n.a.	5,819	0,477	1,39	n.a.	BM *
2	21,11	n.a.	218,327	33,108	96,19	n.a.	MB*
3	21,82	n.a.	5,976	0,539	1,57	n.a.	BMB*
4	23,45	n.a.	3,625	0,296	0,86	n.a.	BMB*
Total:			233,747	34,421	100,00	0,000	

Figure S64. HPLC chromatogram of 1**6**.

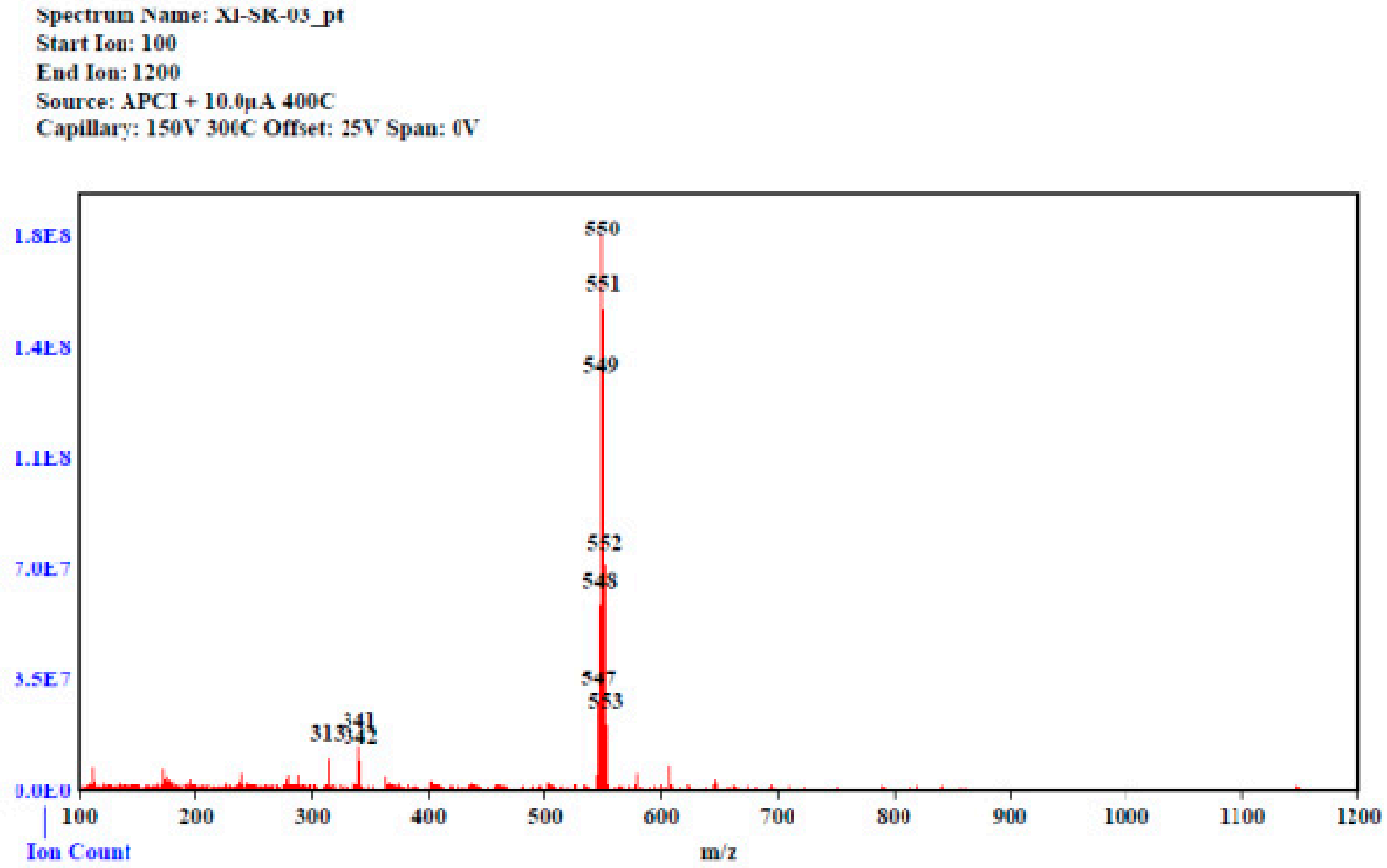

Figure S65. MS spectrum of **16**.

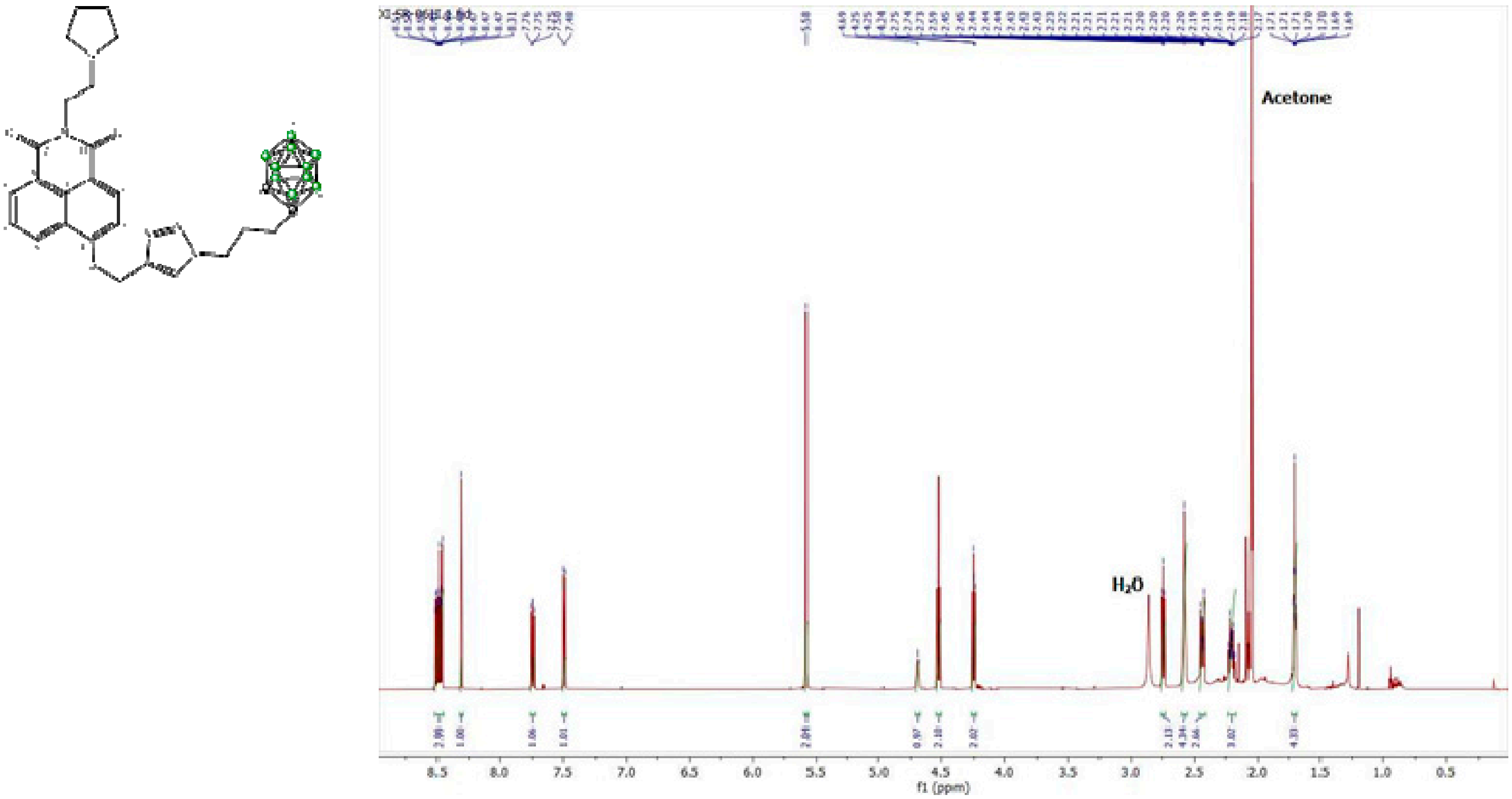

Figure S66. ^{1}H NMR spectrum of **17**.

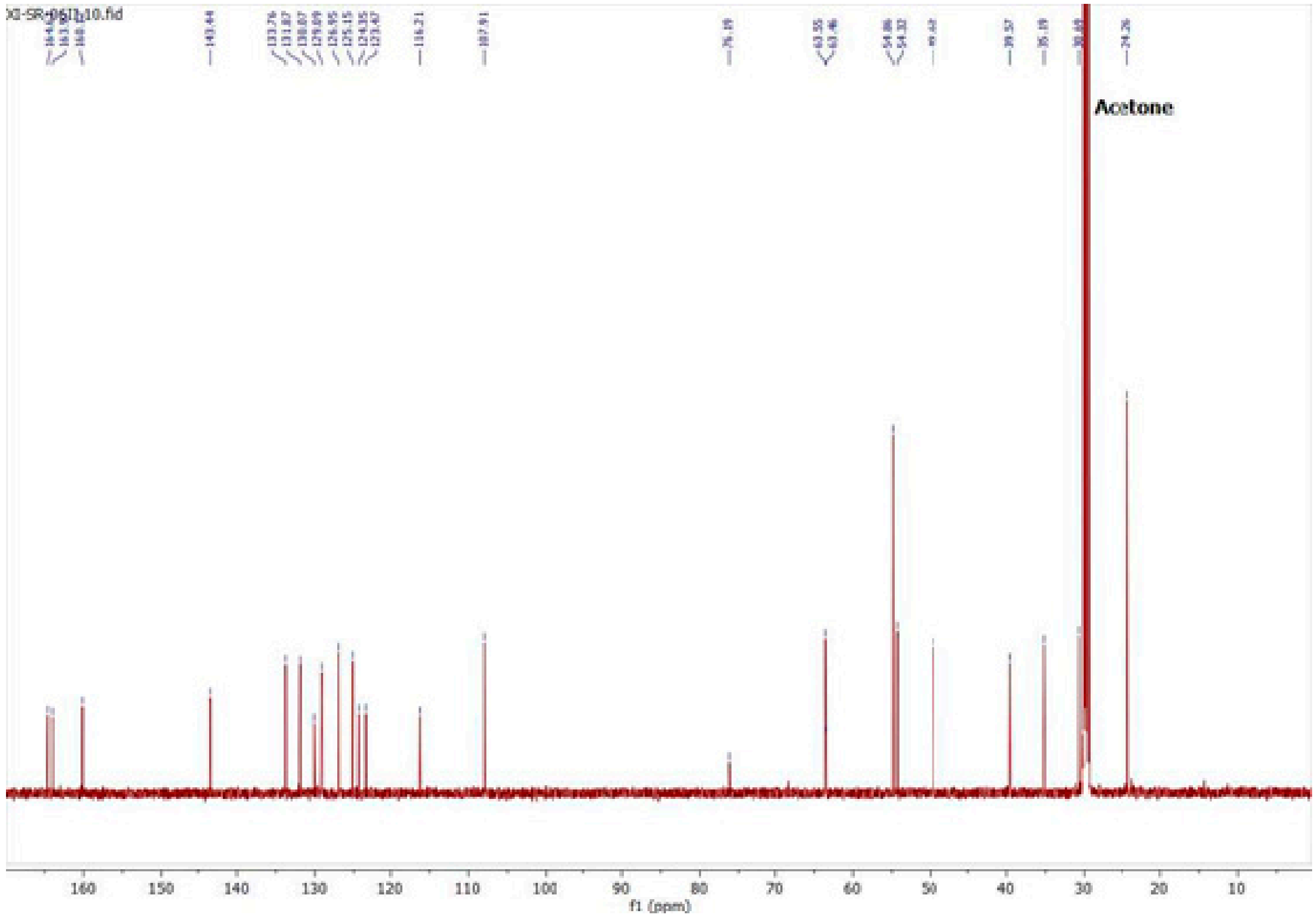

Figure S67. ^{13}C NMR spectrum of **17**.

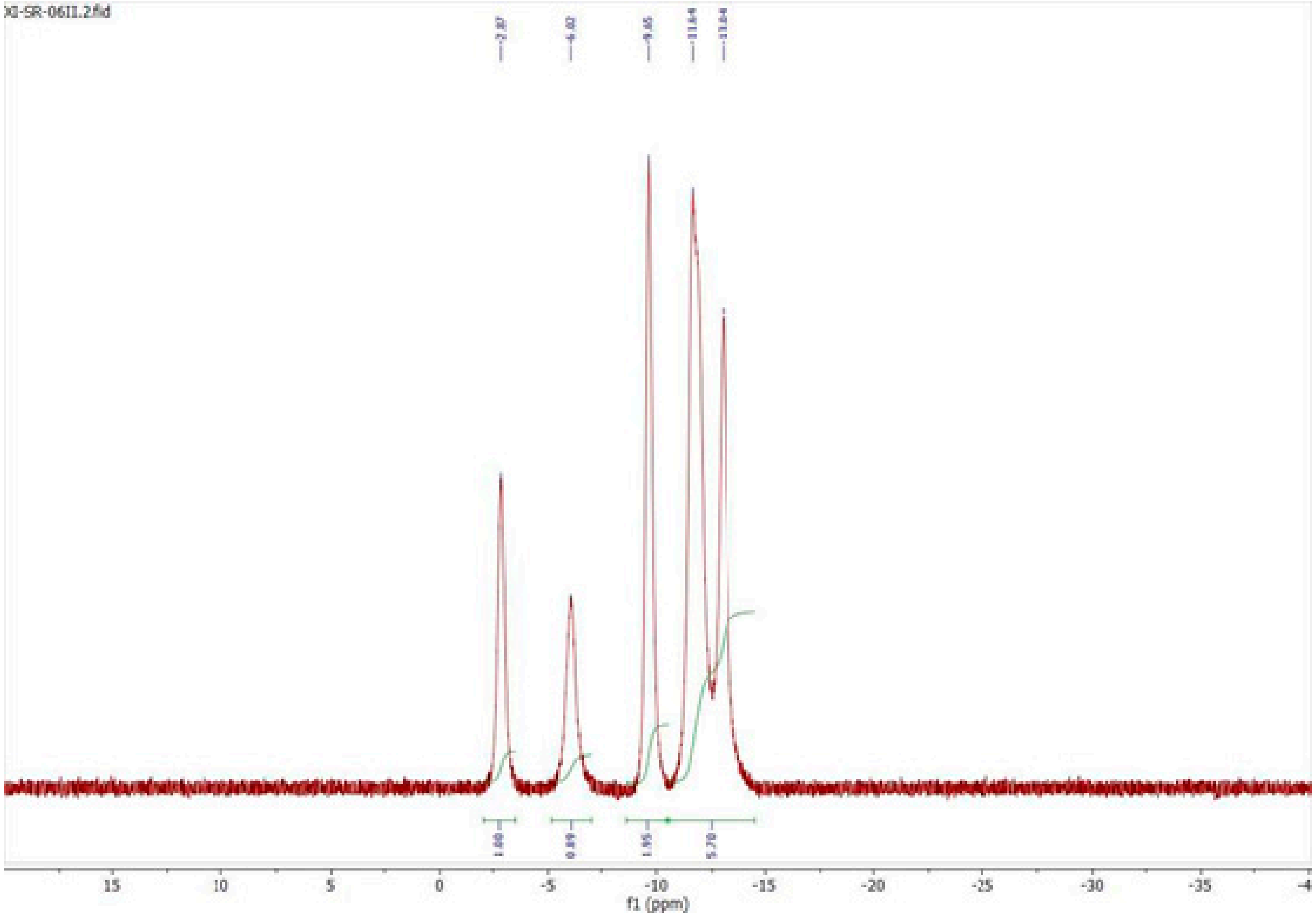

Figure S68. ^{11}B NMR spectrum of **17**.

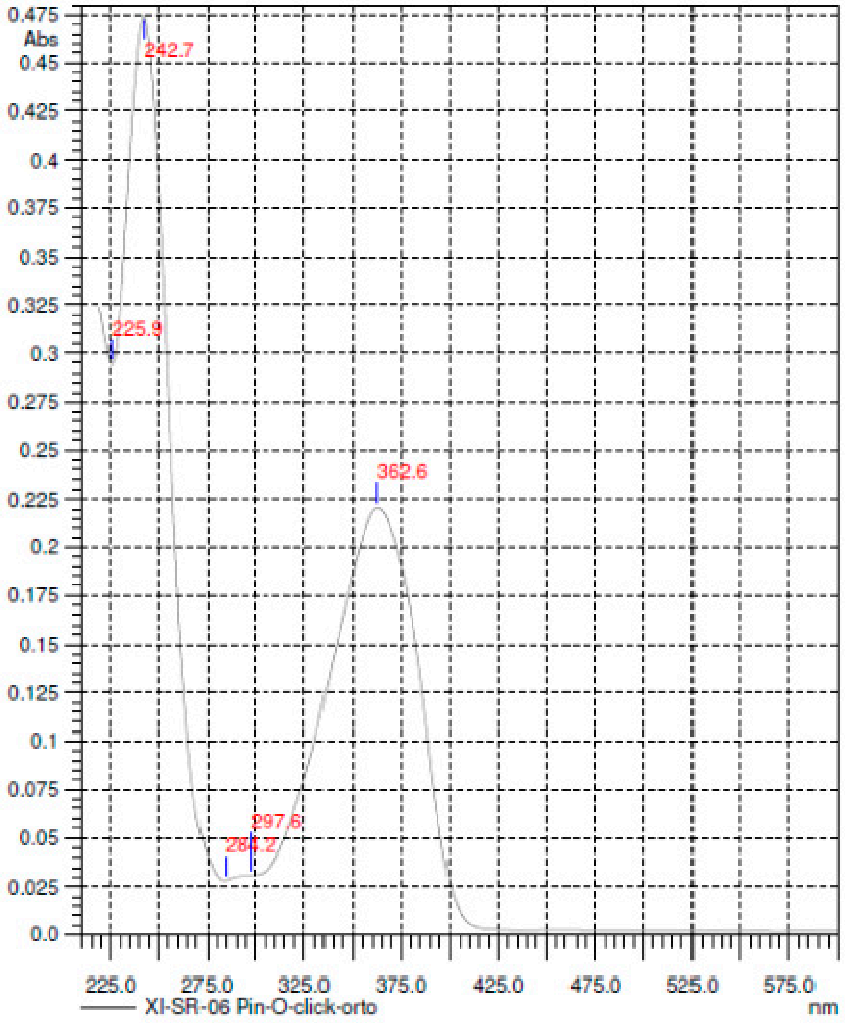

Figure S69. UV spectrum of **17**.

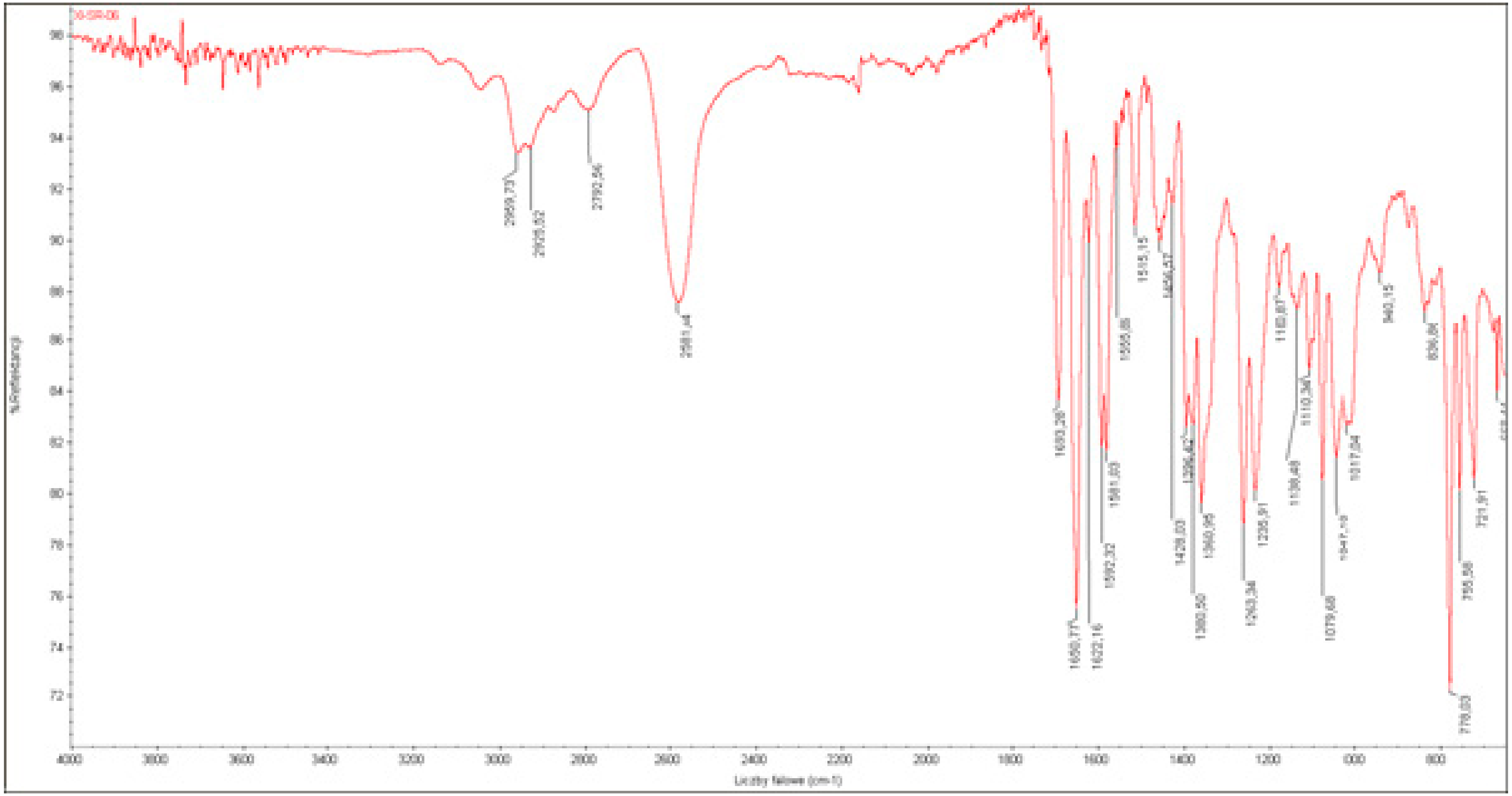

Figure S70. IR spectrum of **17**.

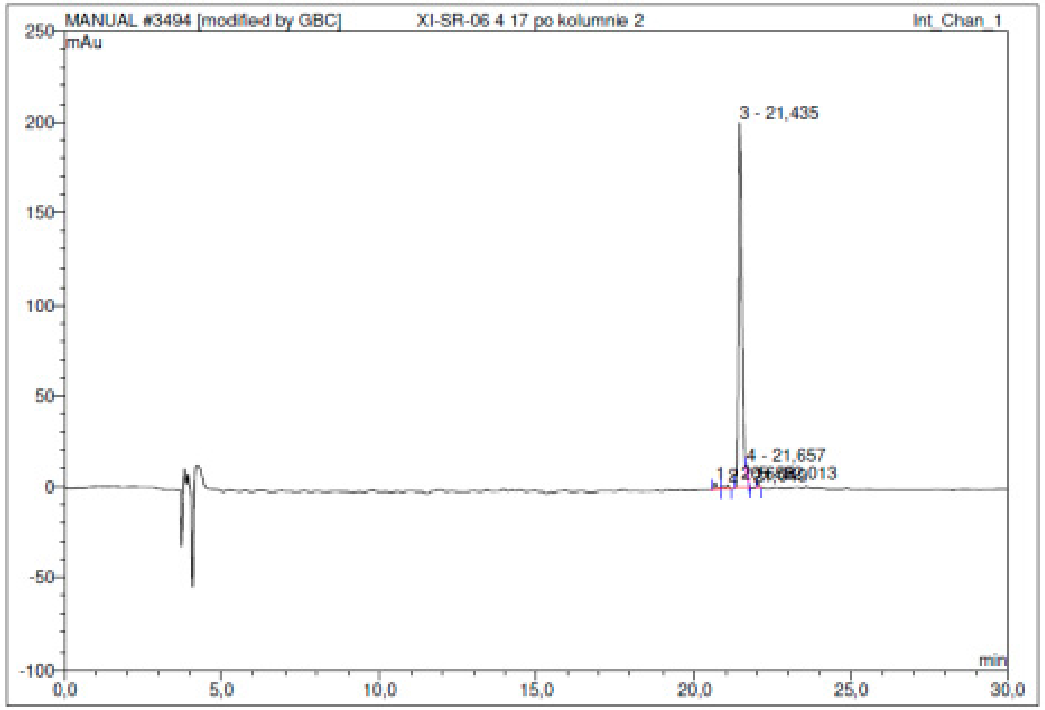

No.	Ret.Time min	Peak Name	Height mAu	Area mAu*min	Rel.Area %	Amount	Type
1	20,65	n.a.	3,600	0,323	1,15	n.a.	BMB*
2	21,05	n.a.	1,953	0,291	1,04	n.a.	BMB*
3	21,44	n.a.	199,994	26,961	96,05	n.a.	BMB*
4	21,66	n.a.	4,139	0,256	0,91	n.a.	Rd*
5	22,01	n.a.	2,615	0,238	0,85	n.a.	BMB*
Total:			212,301	28,069	100,00	0,000	

Figure S71. HPLC chromatogram of **17**.

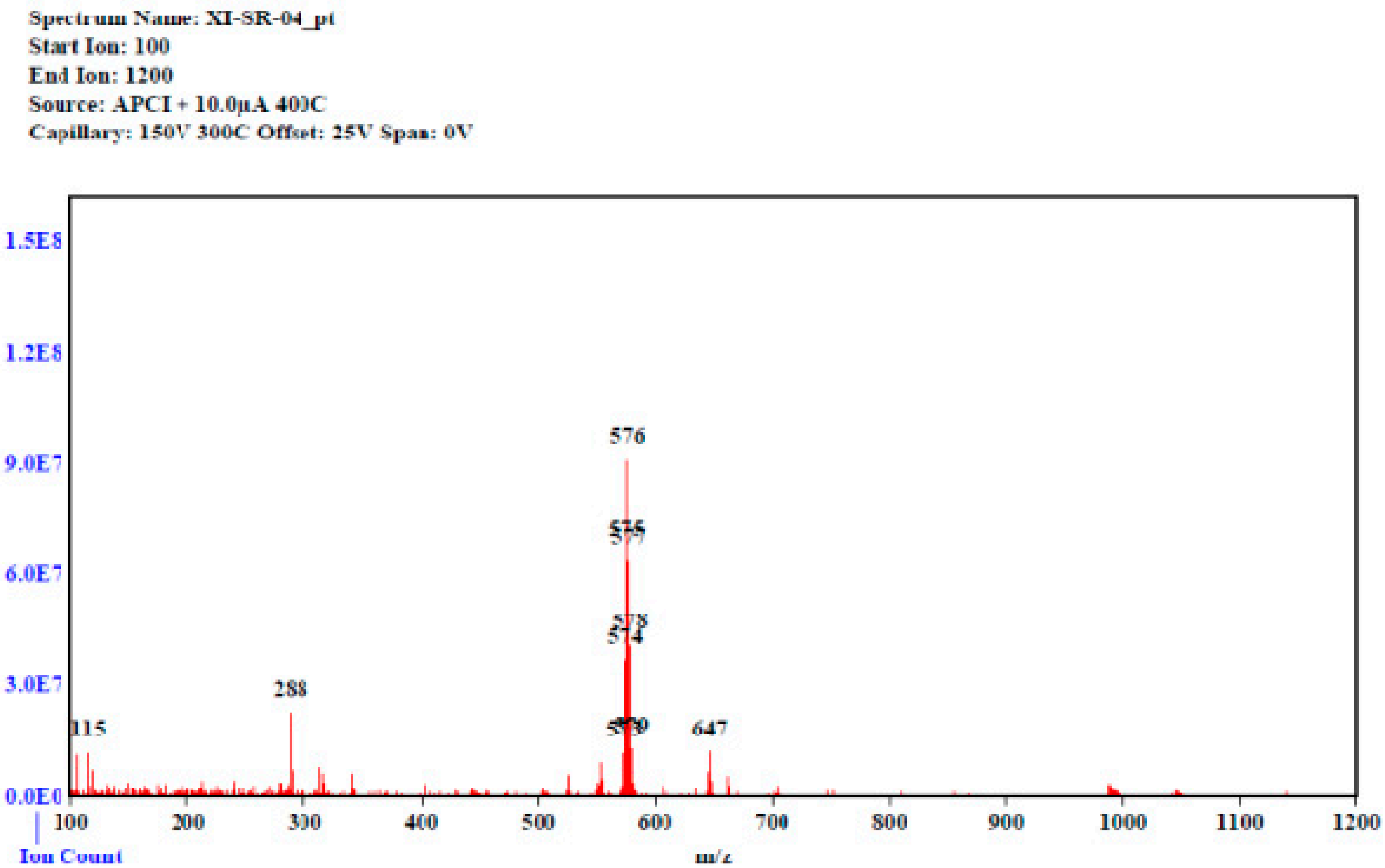

Figure S72. MS spectrum of **17**.

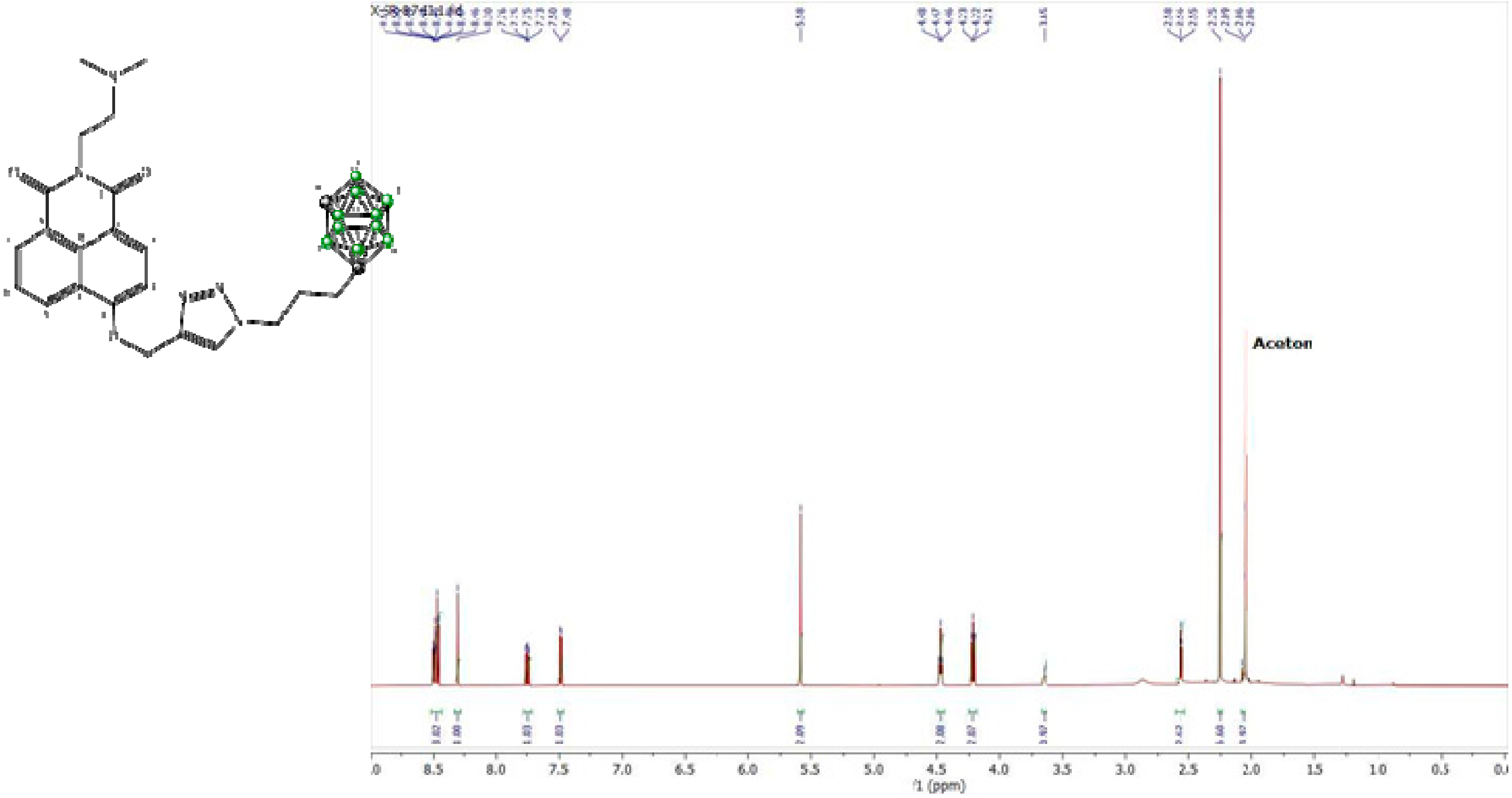

Figure S73. ^{1}H NMR spectrum of **18**.

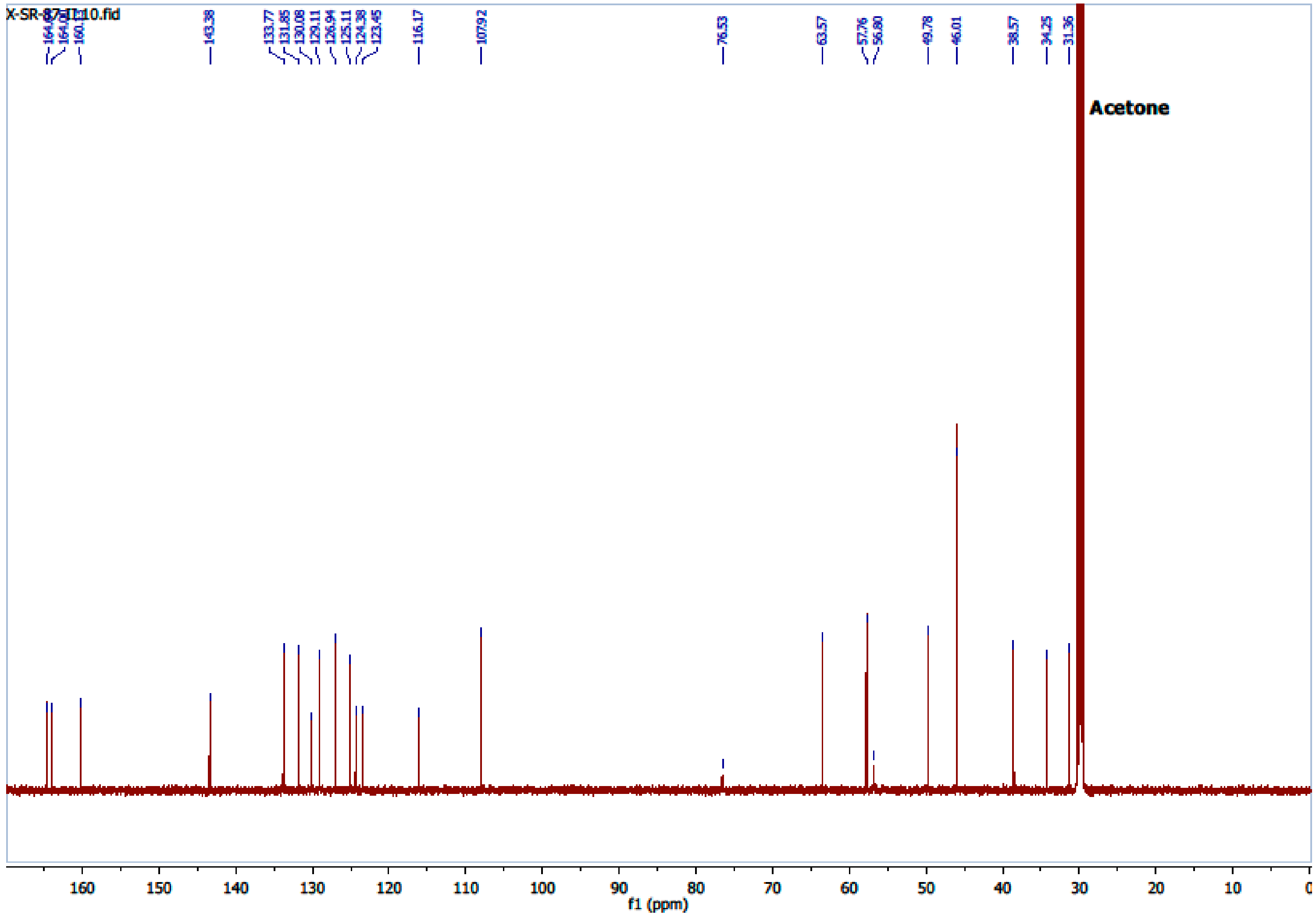

Figure S74. ^{13}C NMR spectrum of **18**.

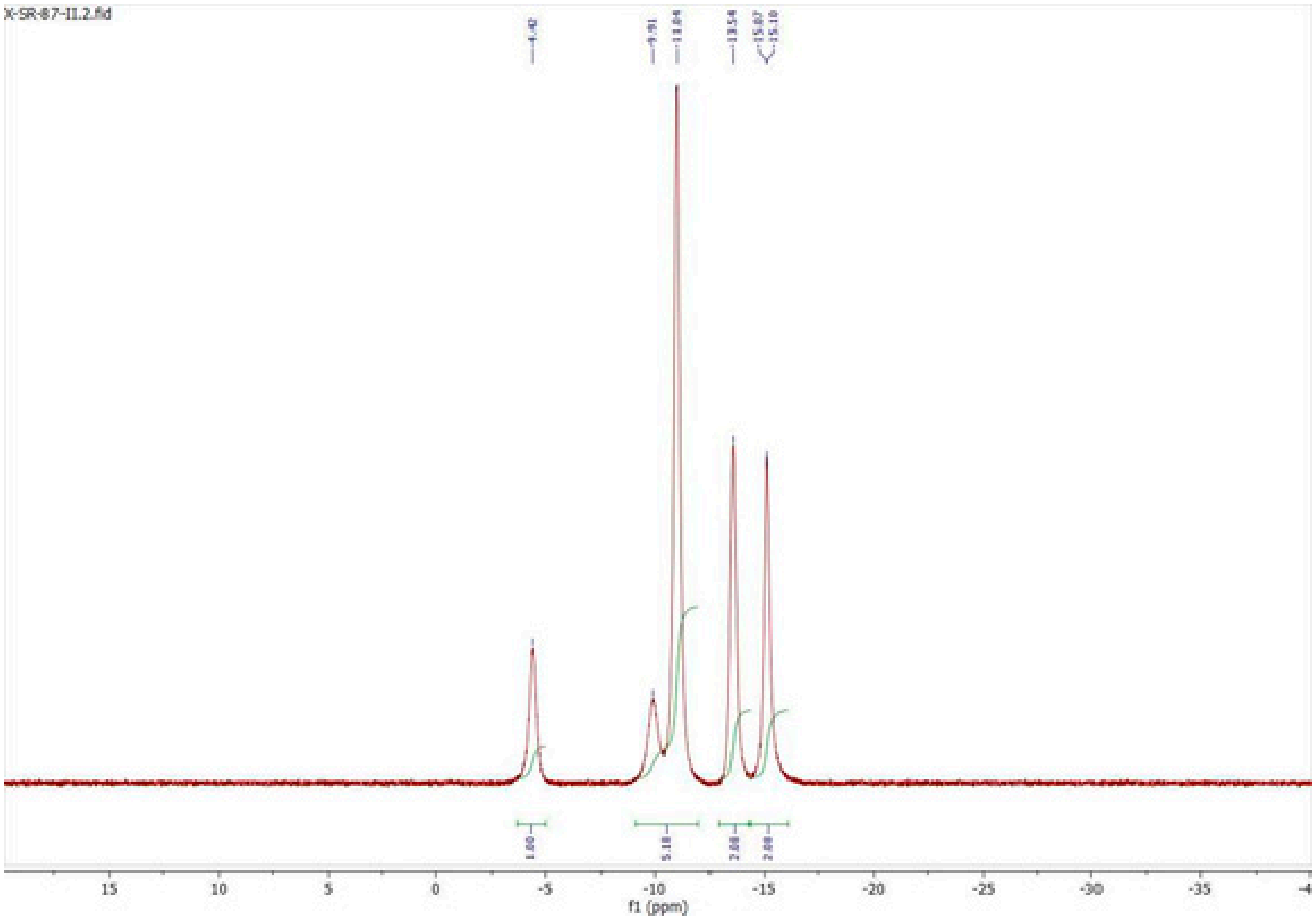

Figure S75. ^{11}B NMR spectrum of **18**.

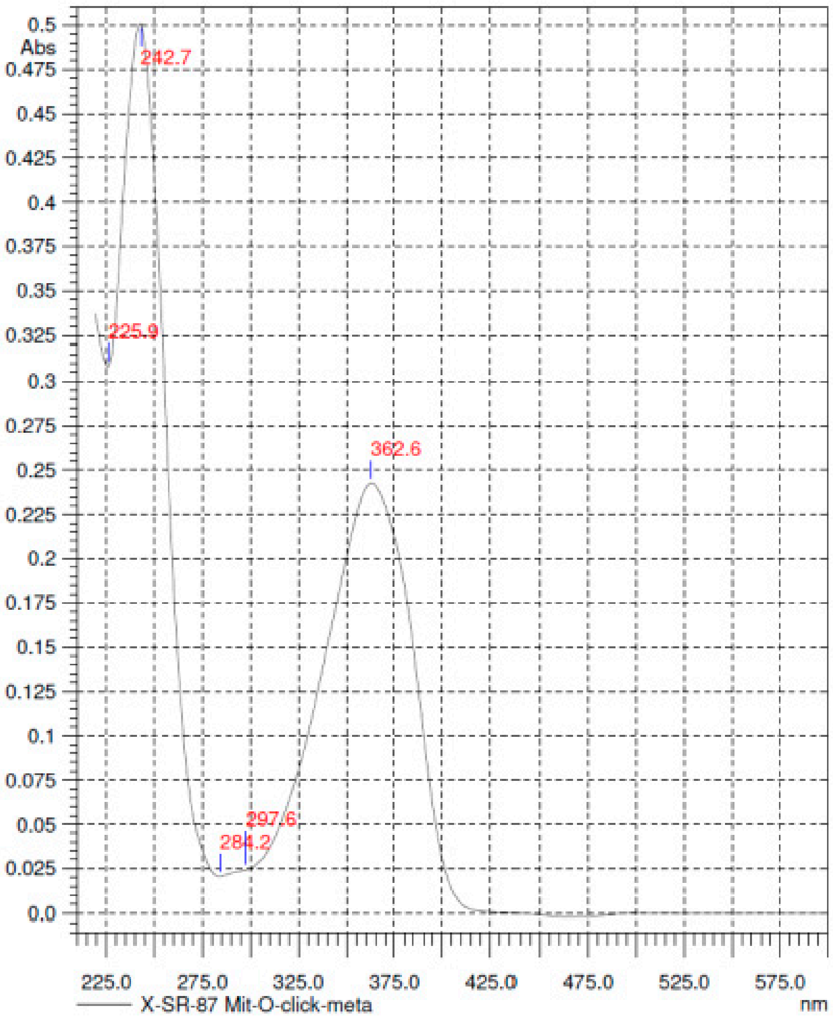

Figure S76. UV spectrum of **18**.

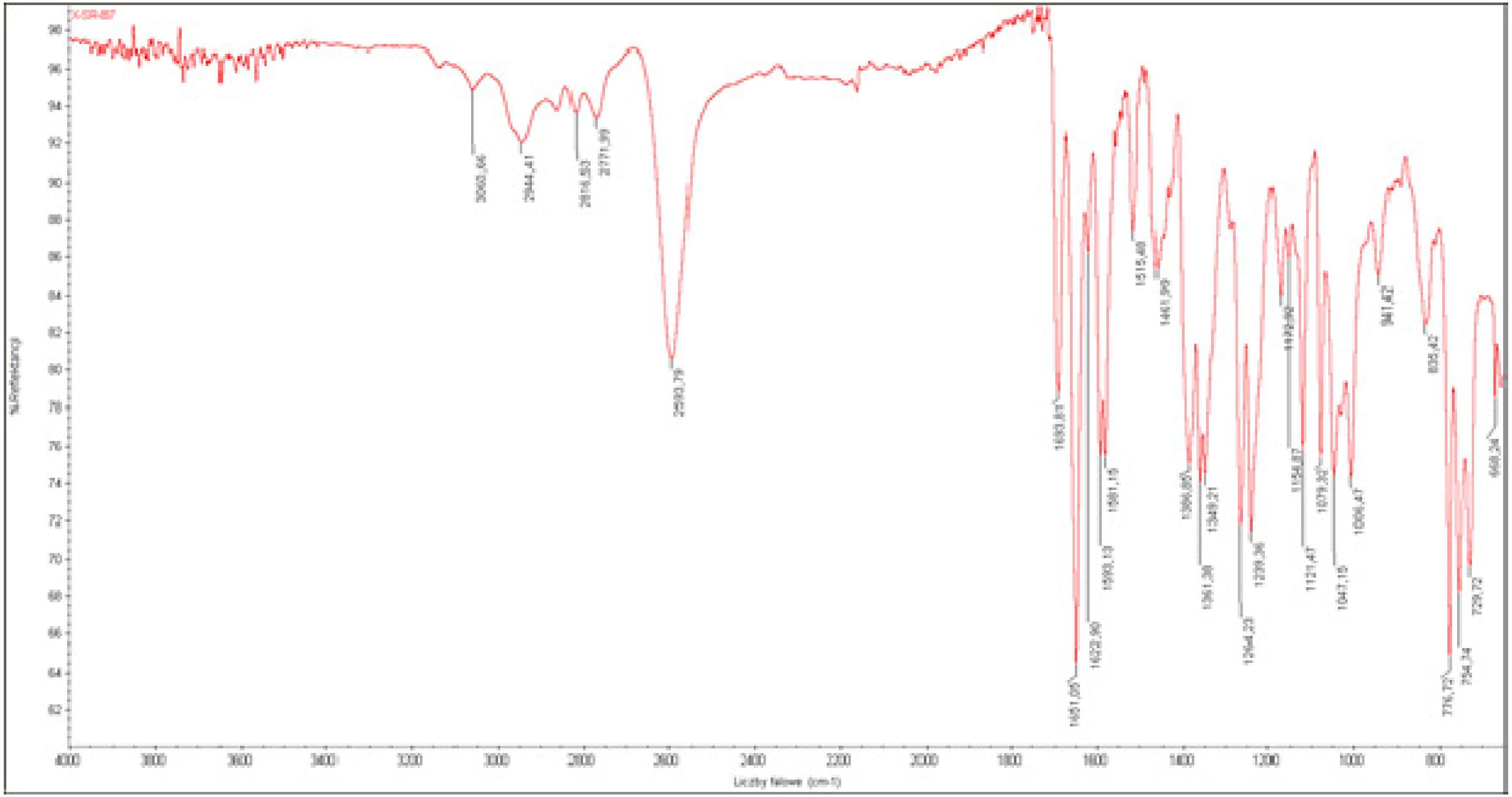

Figure S77. IR spectrum of **18**.

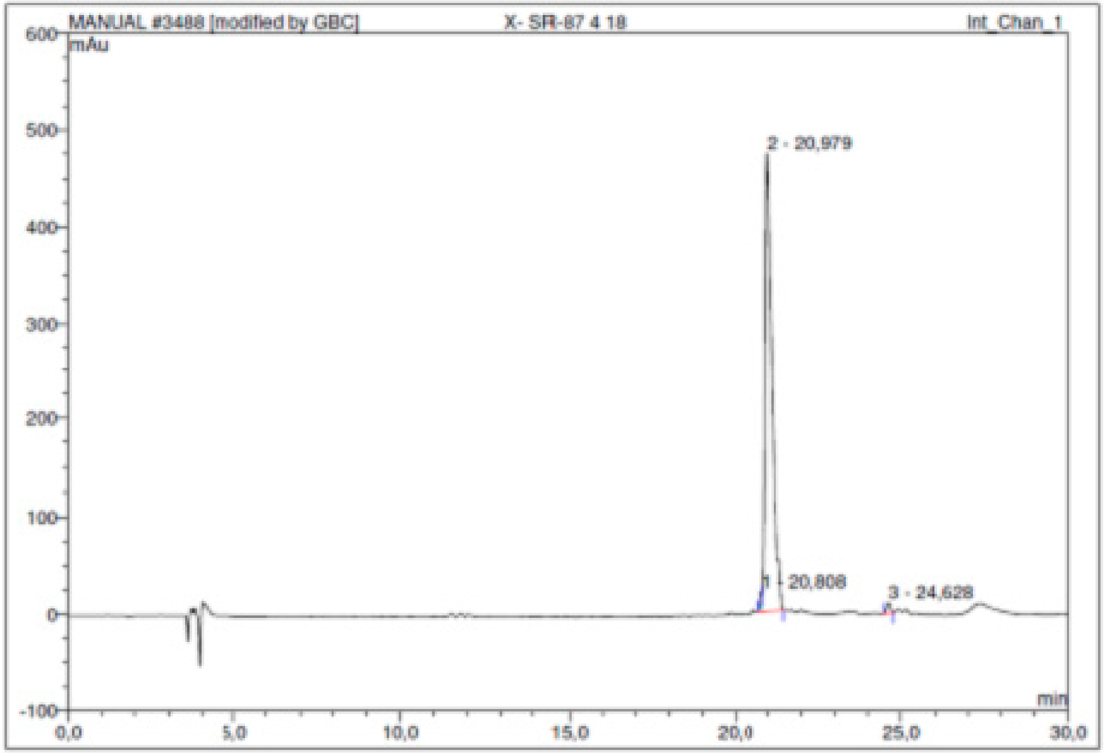

No.	Ret.Time min	Peak Name	Height mAu	Area mAu*min	Rel.Area %	Amount	Type
1	20,81	n.a.	20,492	1,456	1,30	n.a.	BM *
2	20,98	n.a.	472,719	109,545	97,46	n.a.	MB*
3	24,63	n.a.	10,590	1,393	1,24	n.a.	BMB*
Total:			503,802	112,394	100,00	0,000	

Figure S78. HPLC chromatogram of **18**.

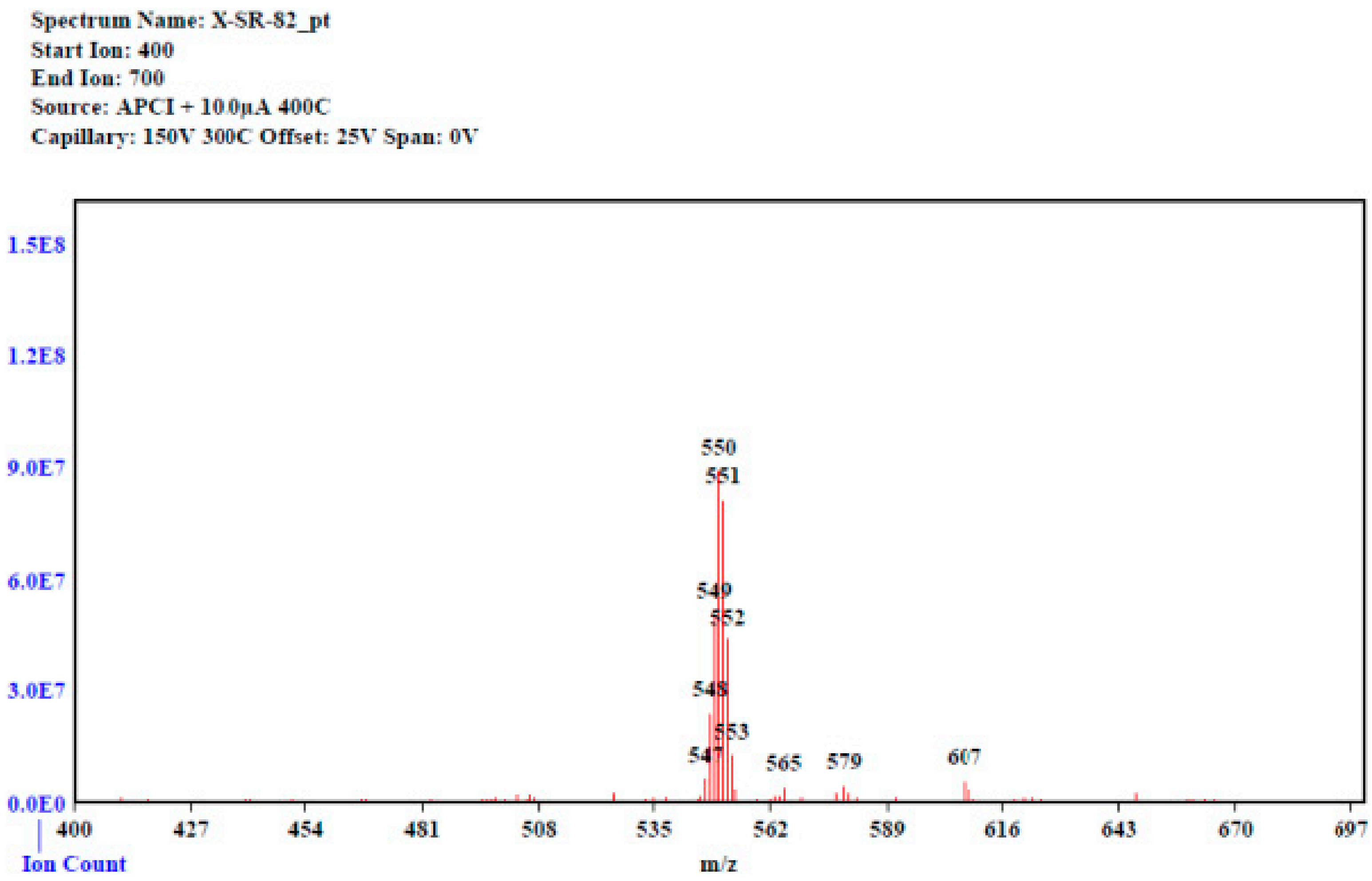

Figure S79. MS spectrum of **18**.

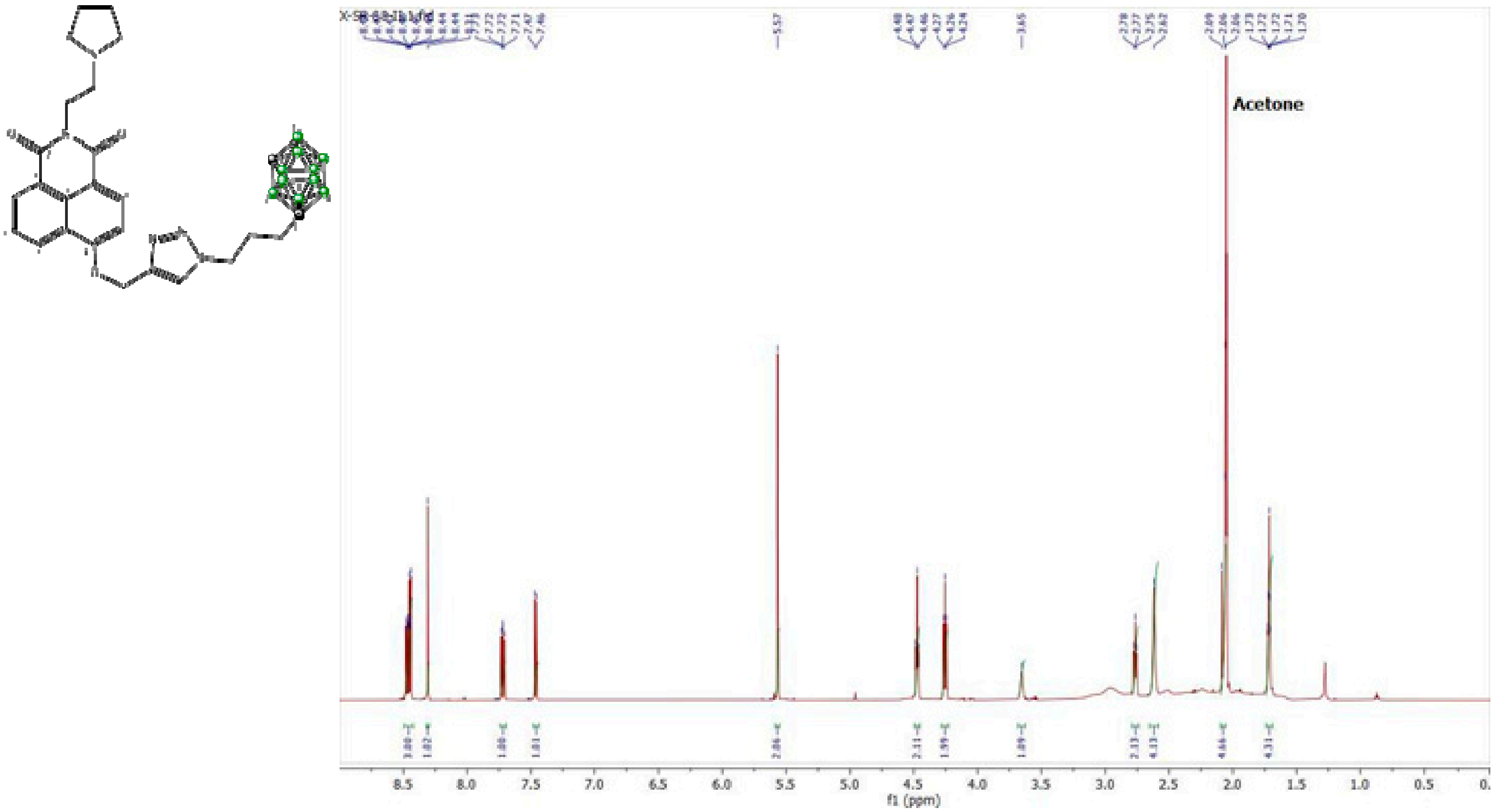

Figure S80. ^{1}H NMR spectrum of **19**.

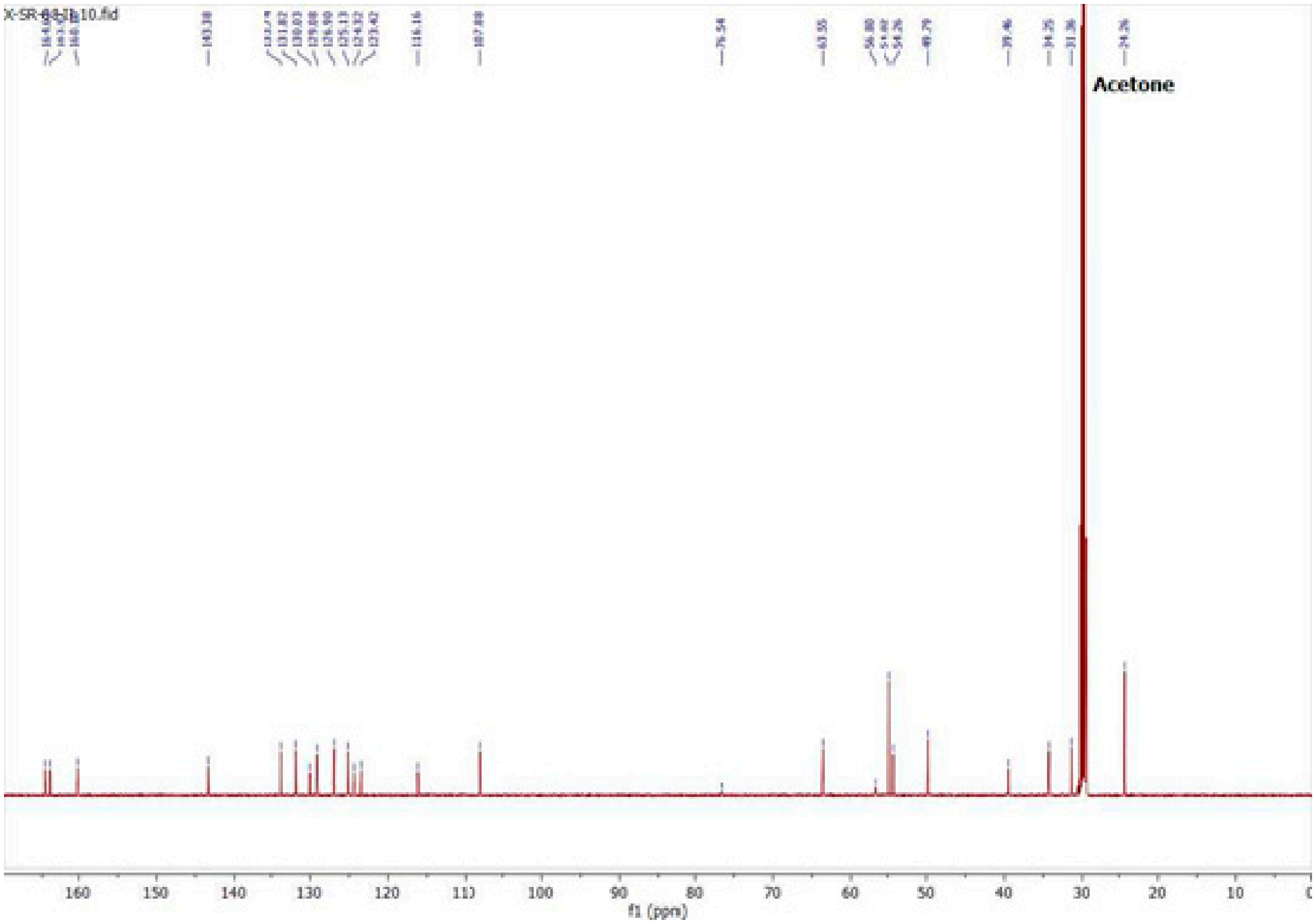

Figure S81. ^{13}C NMR spectrum of **19**.

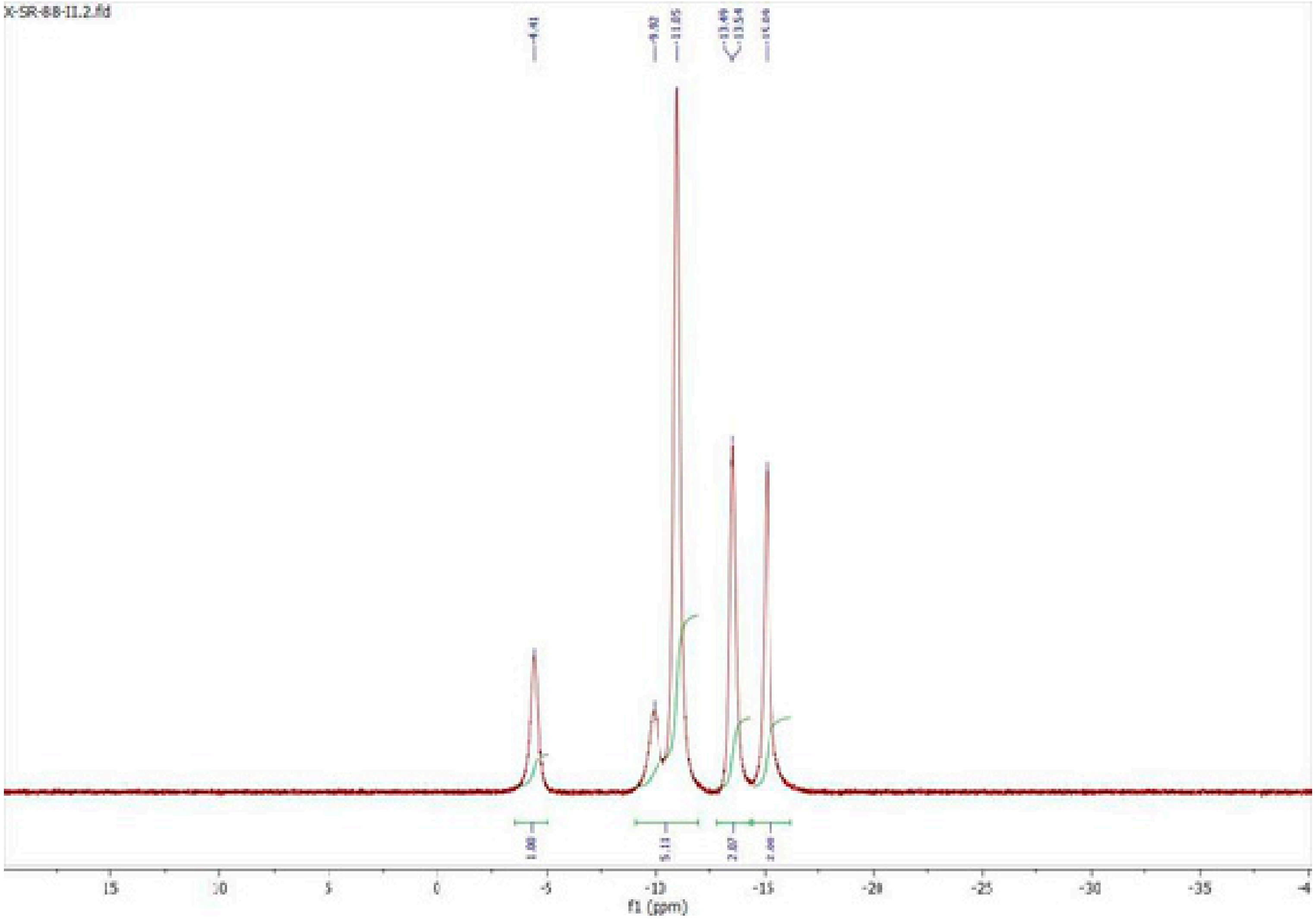

Figure S82. ^{11}B NMR spectrum of **19**.

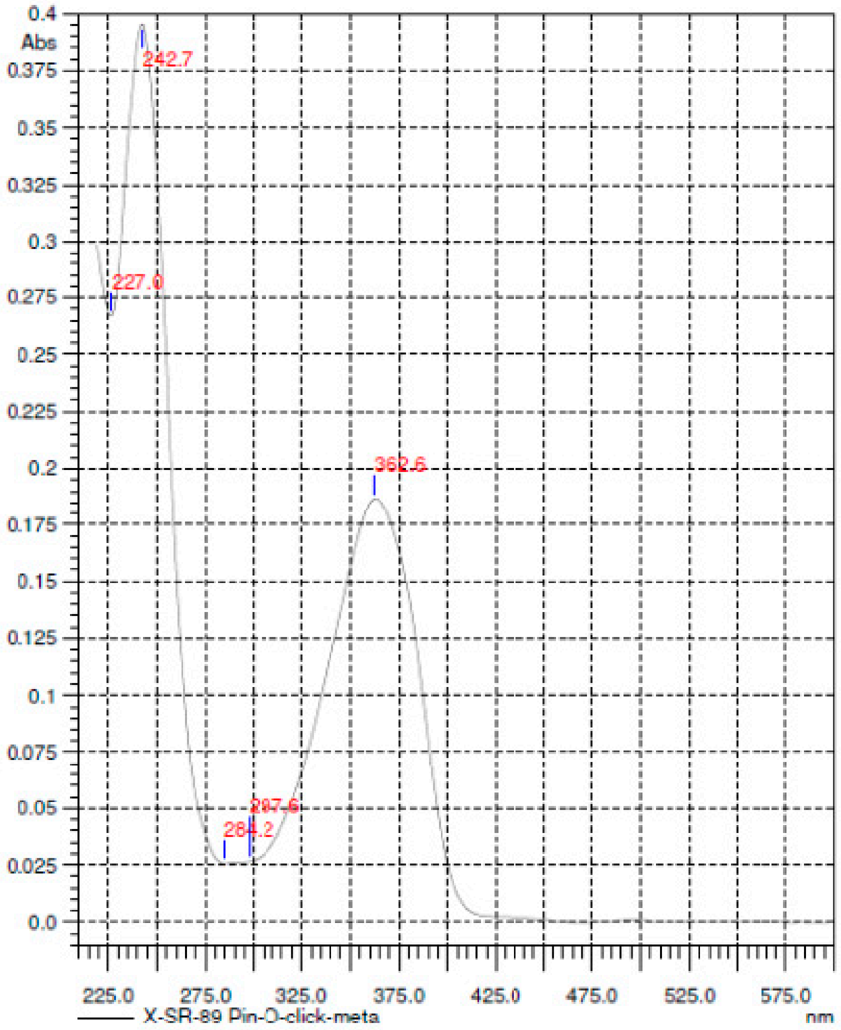

Figure S83. UV spectrum of **19**.

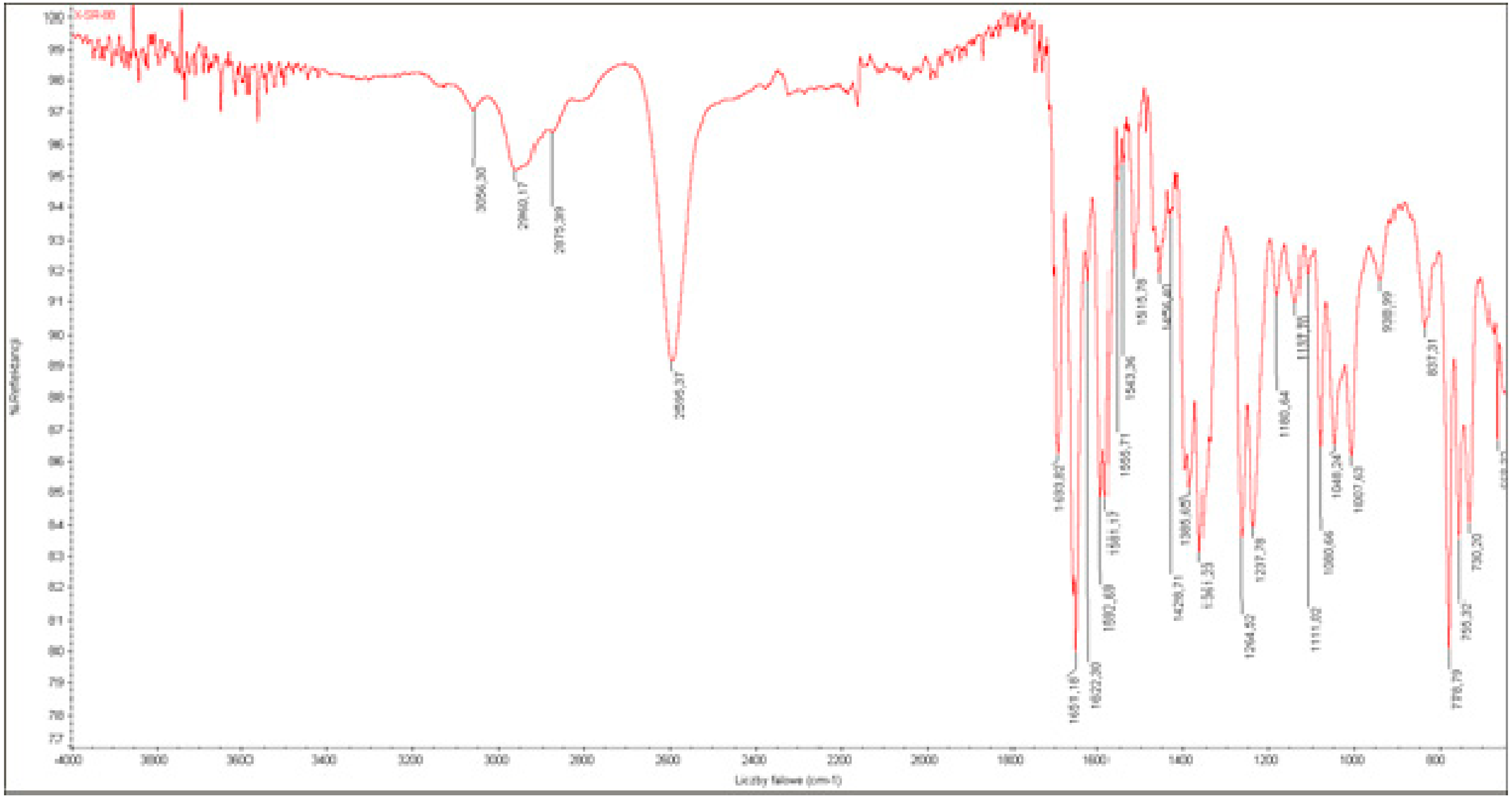

Figure S84. IR spectrum of **19**.

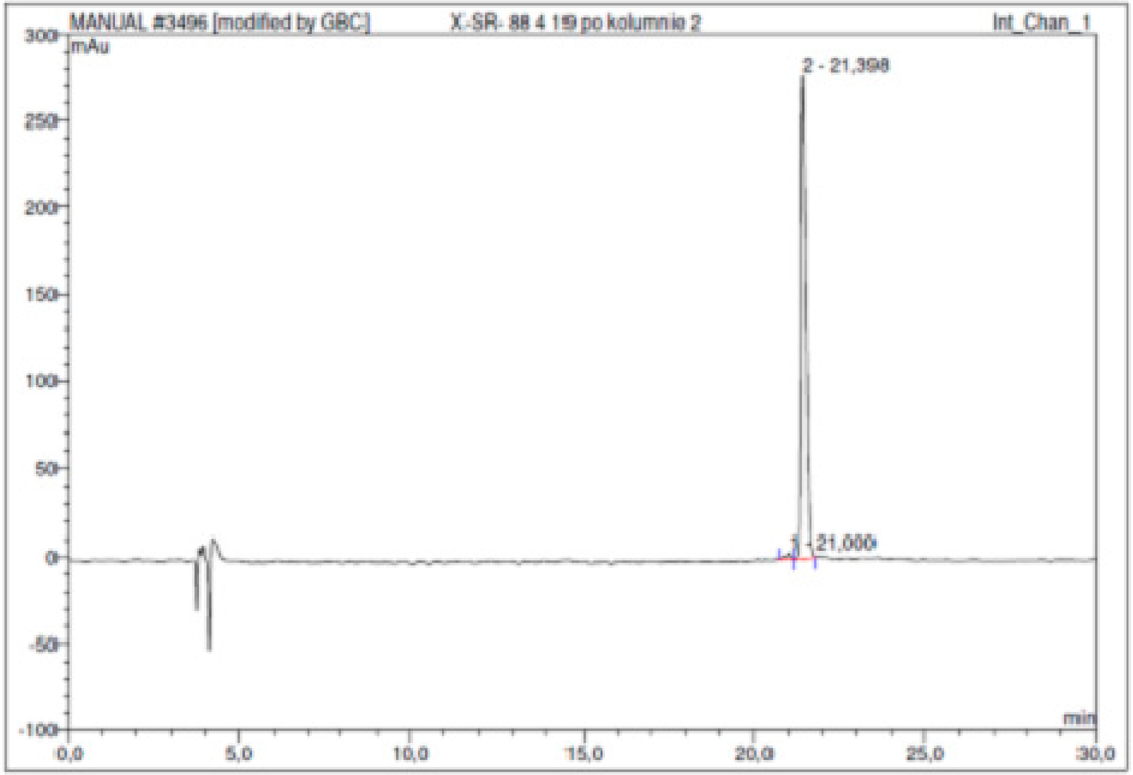

No.	Ret.Time min	Peak Name	Height mAu	Area mAu*min	Rel.Area %	Amount	Type
1	21,00	n.a.	3,392	0,508	1,08	n.a.	BMB*
2	21,40	n.a.	276,558	46,534	98,92	n.a.	BMB
Total:			279,950	47,042	100,00	0,000	

Figure S85. HPLC chromatogram of **19**.

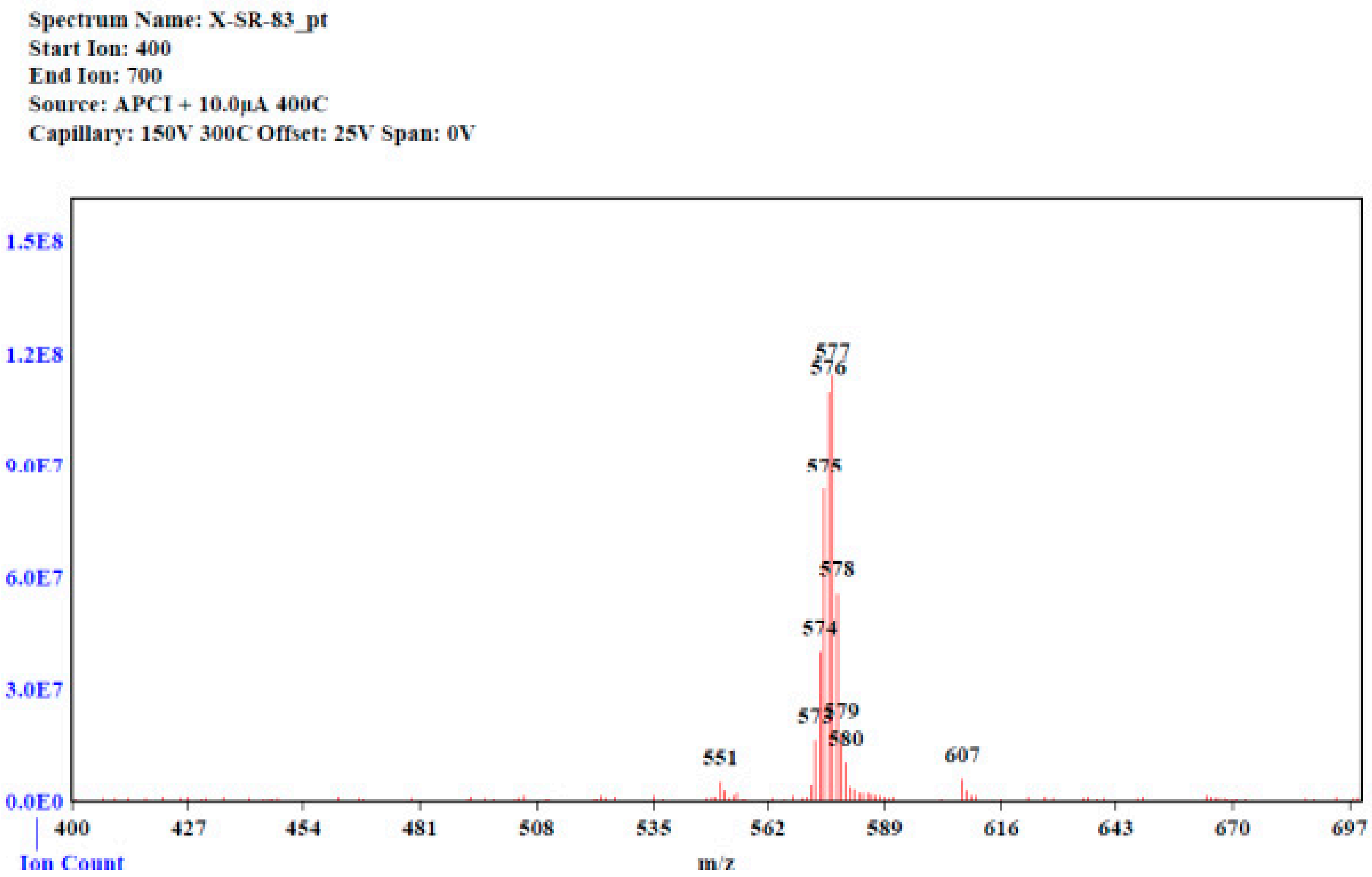

Figure S86. MS spectrum of **19**.

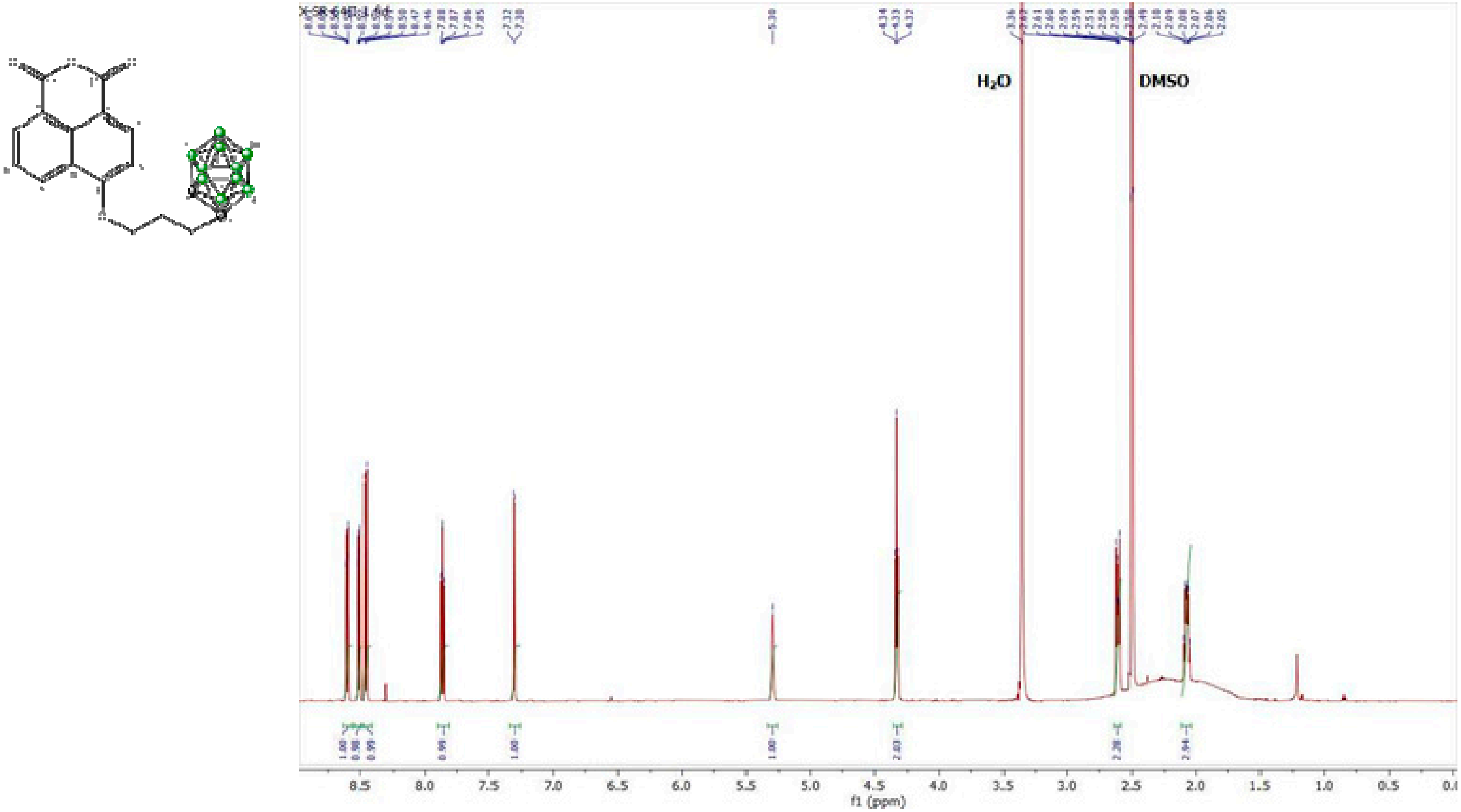

Figure S87. ^{1}H NMR spectrum of **22**.

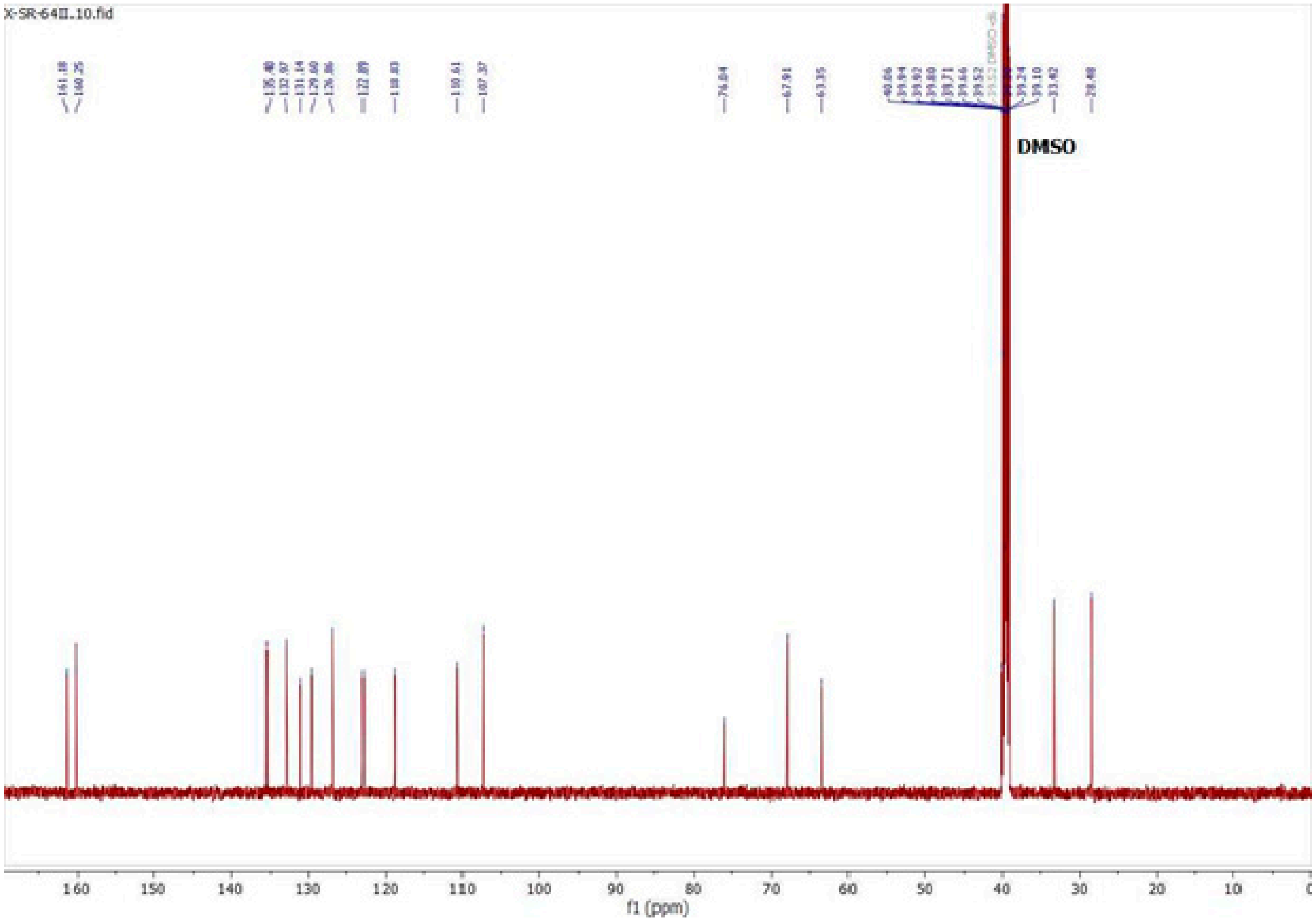

Figure S88. ^{13}C NMR spectrum of **22**.

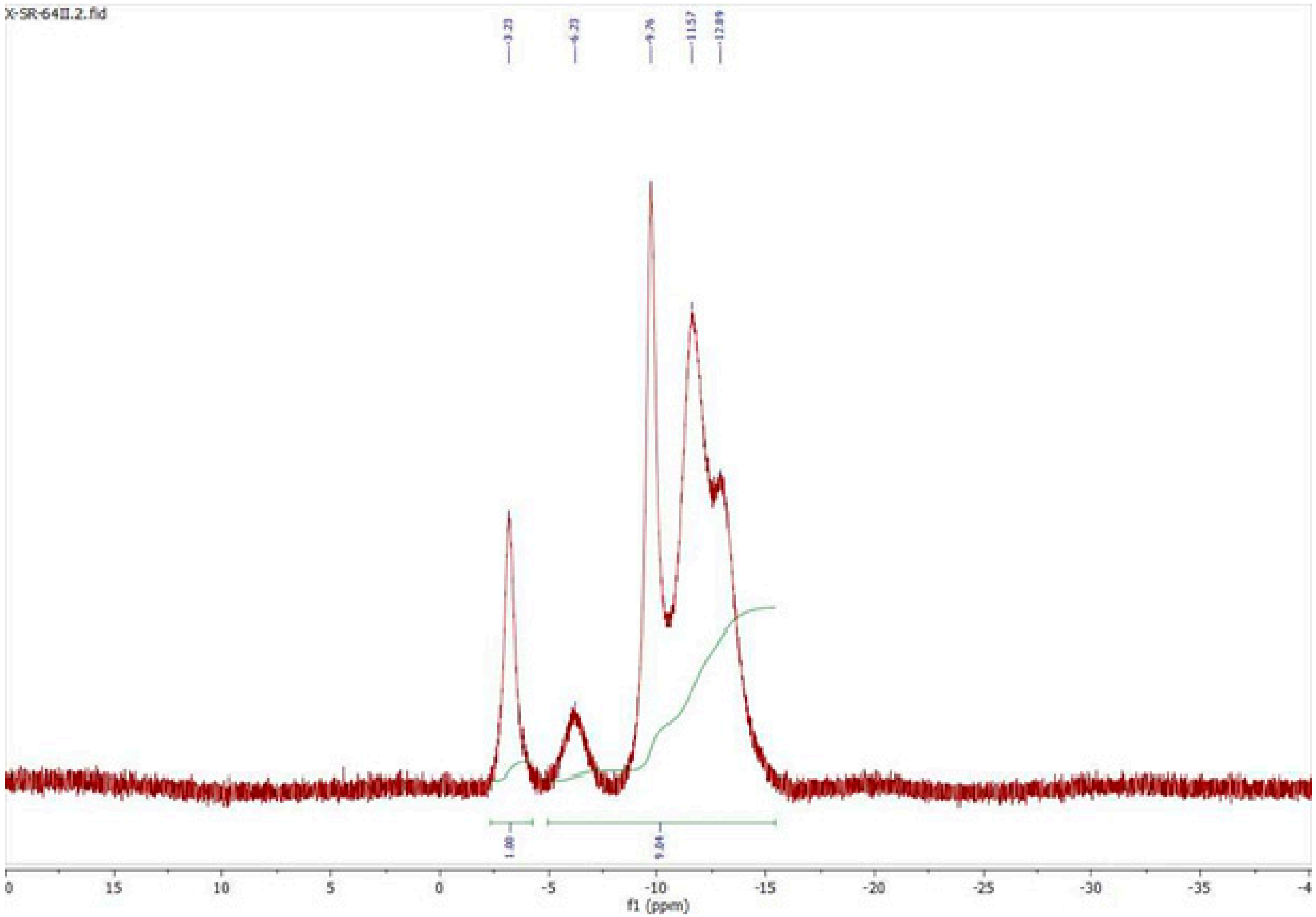

Figure S89. ^{11}B NMR spectrum of **22**.

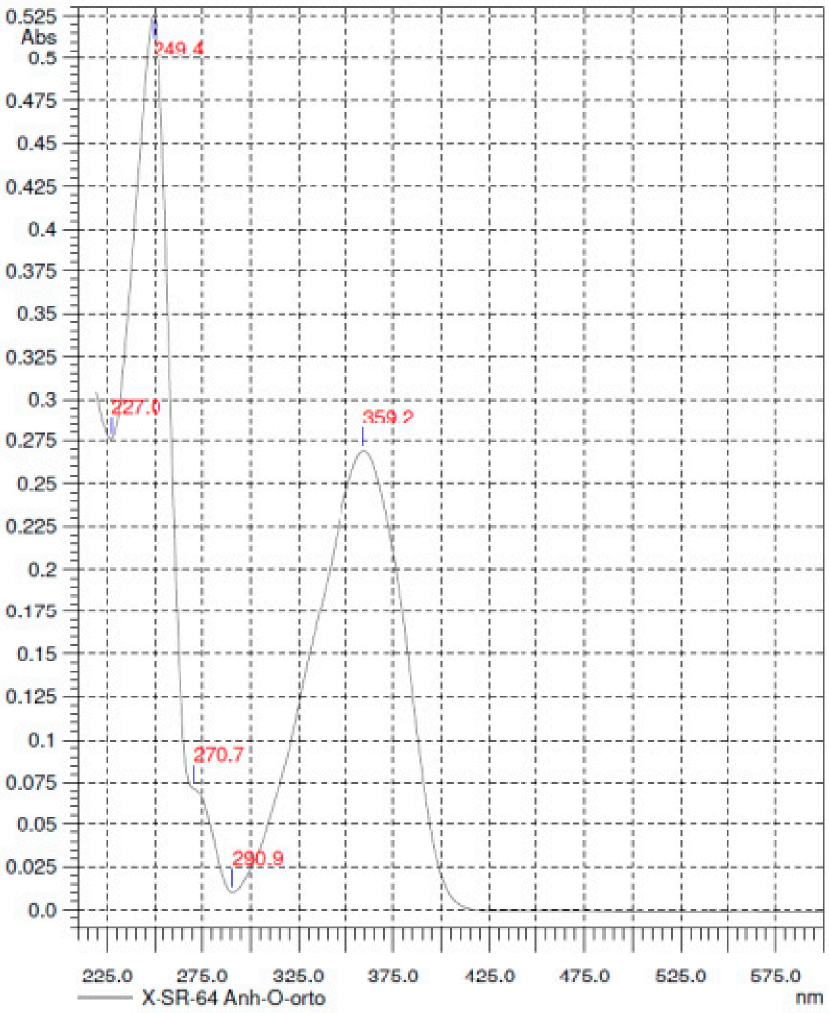

Figure S90. UV spectrum of **22.**

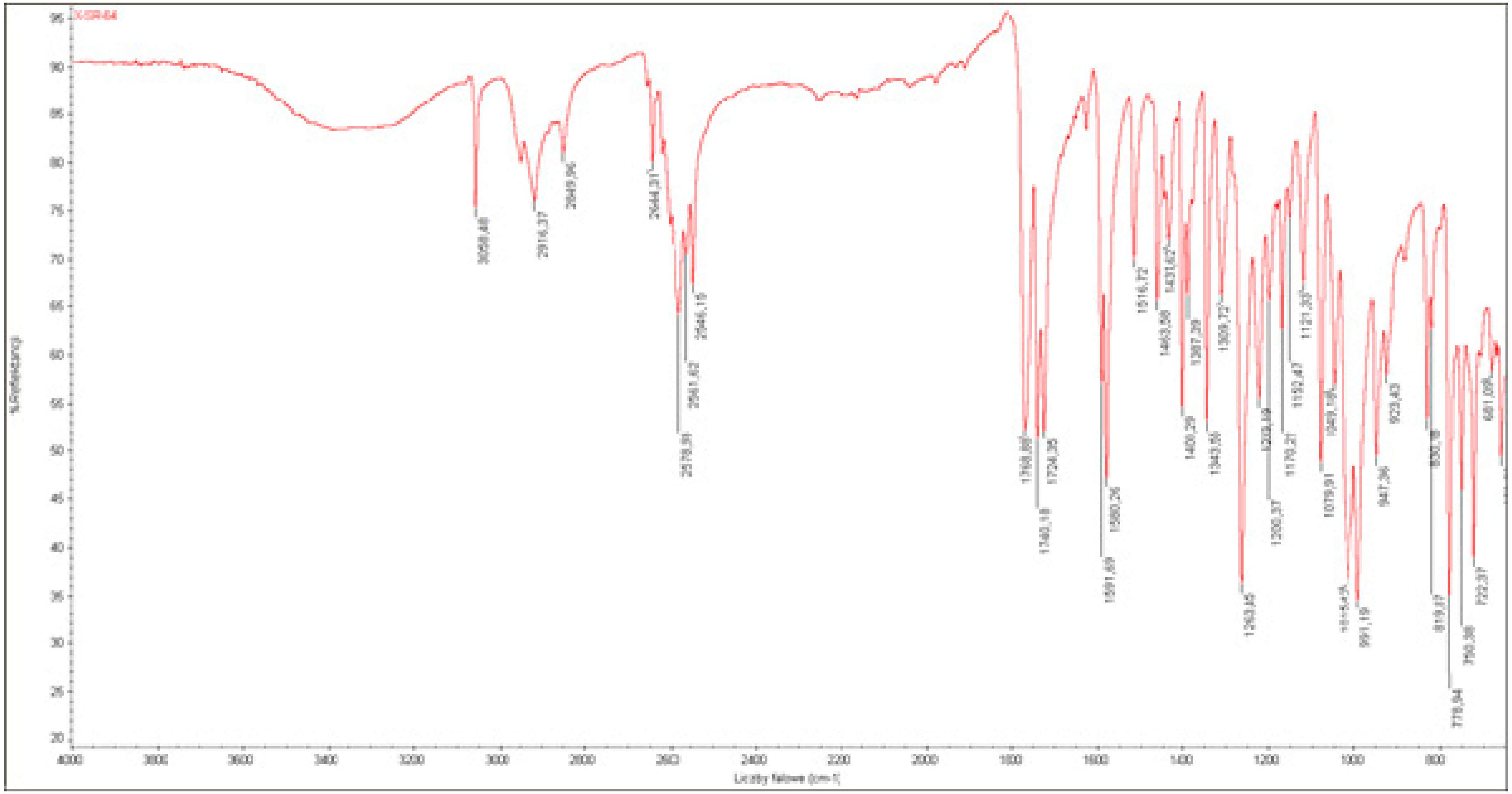

Figure S91. IR spectrum of **22**.

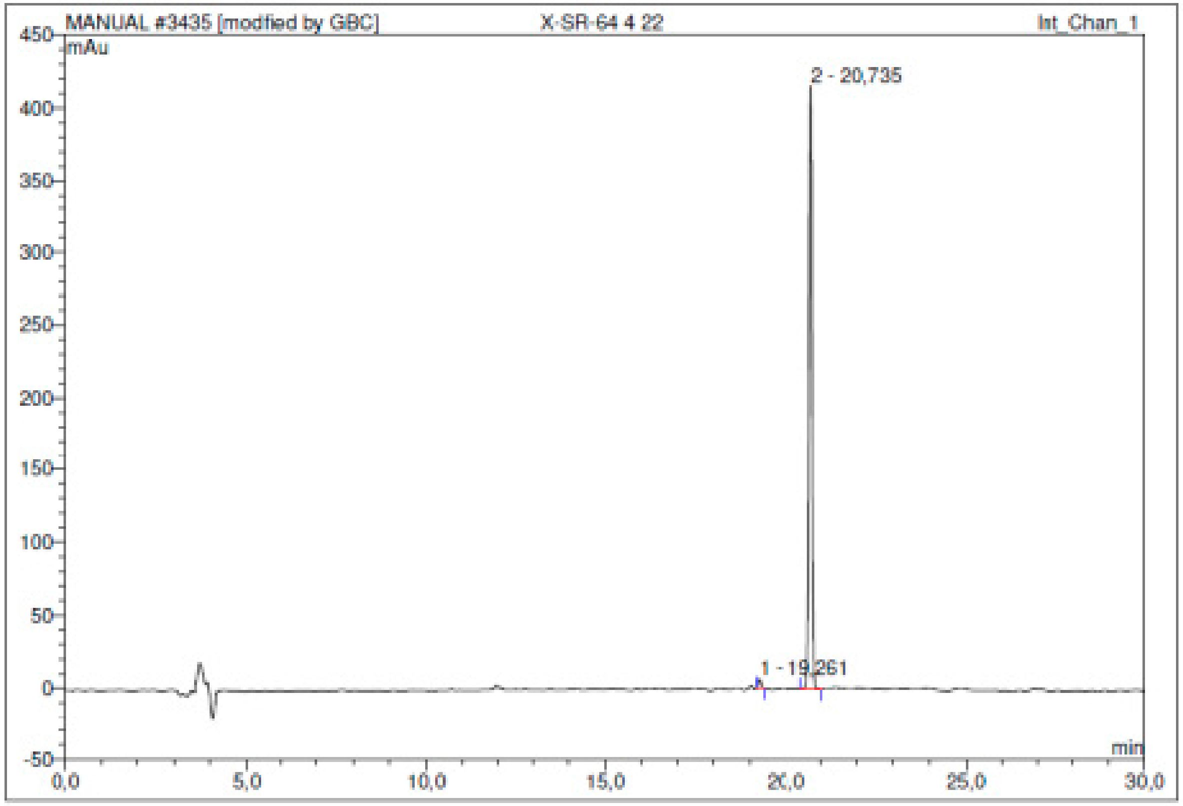

No.	Ret.Time min	Peak Name	Height mAu	Area mAu*min	Rel.Area %	Amount	Type
1	19,26	n.a.	6,173	0,554	1,35	n.a.	BMB*
2	20,74	n.a.	415,428	40,609	98,65	n.a.	BMB
Total:			421,601	41,163	100,00	0,000	

Figure S92. HPLC chromatogram of **22**.

Spectrum Name: X-SR-64_Anh-O-Orto_pt
Start Ion: 200
End Ion: 500
Source: APCI + 10.0µA 400C
Capillary: 150V 300C Offset: 25V Span: 0V

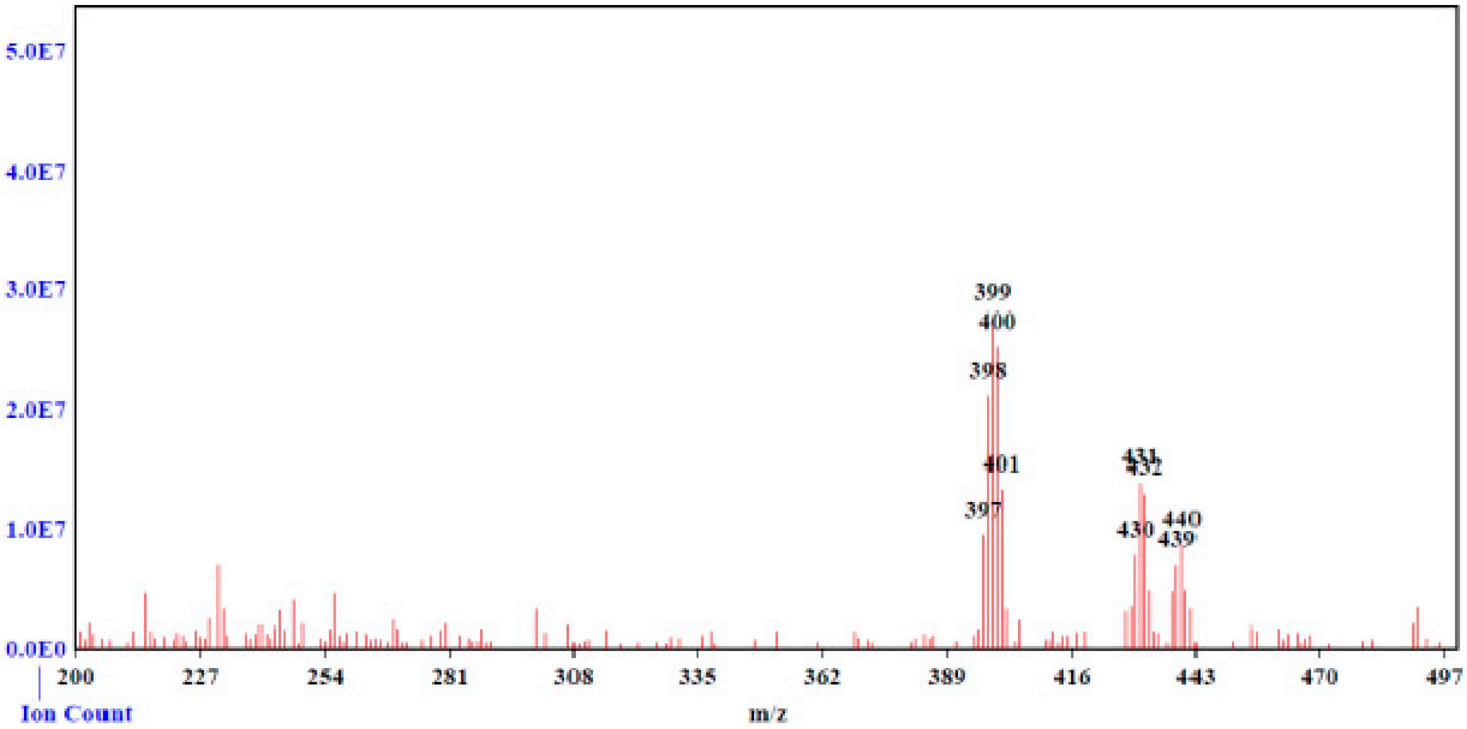

Figure S93. MS spectrum of **22**.

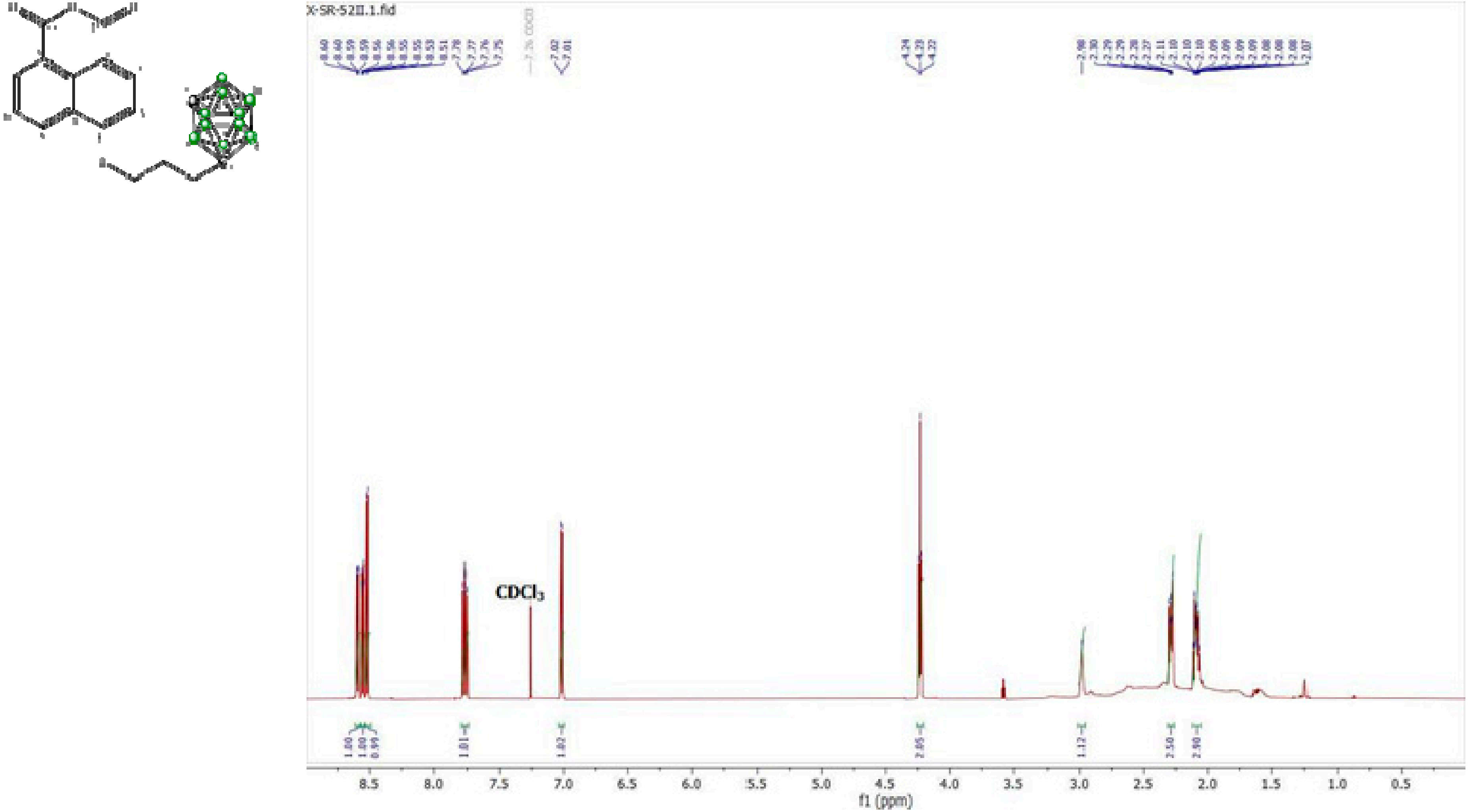

Figure S94. ^{1}H NMR spectrum of **23**.

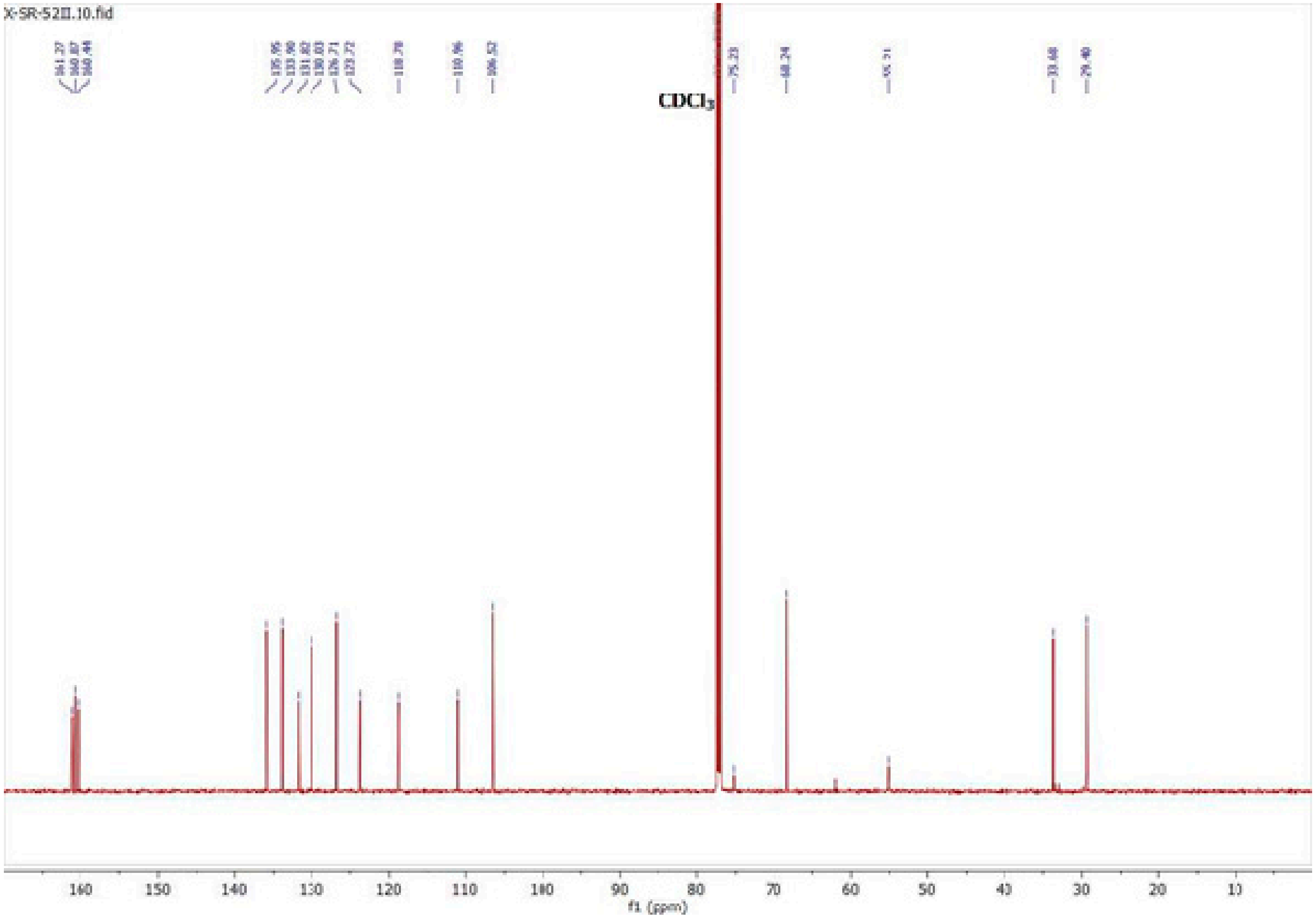

Figure S95. ^{13}C NMR spectrum of **23**.

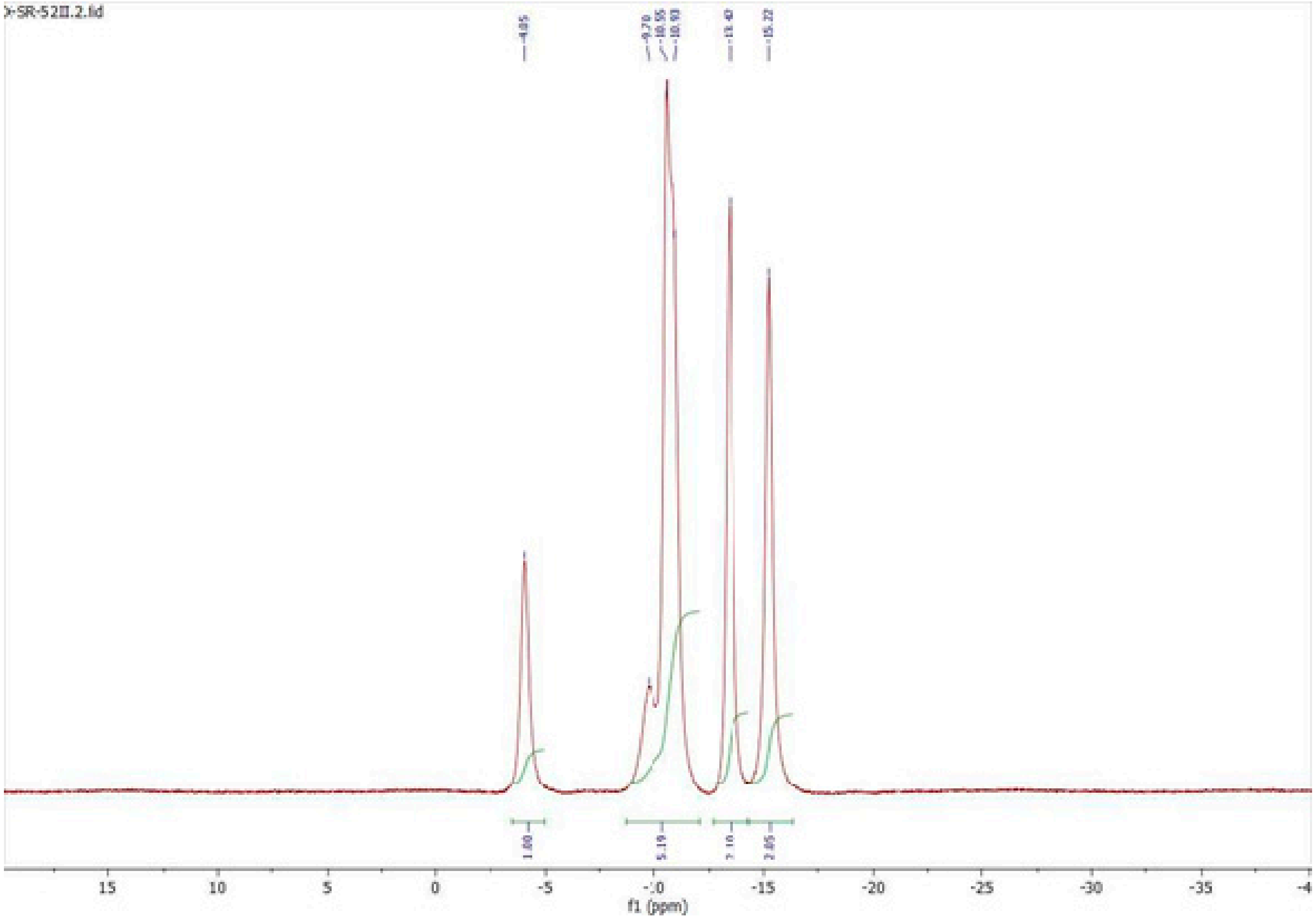

Figure S96. ^{11}B NMR spectrum of **23**.

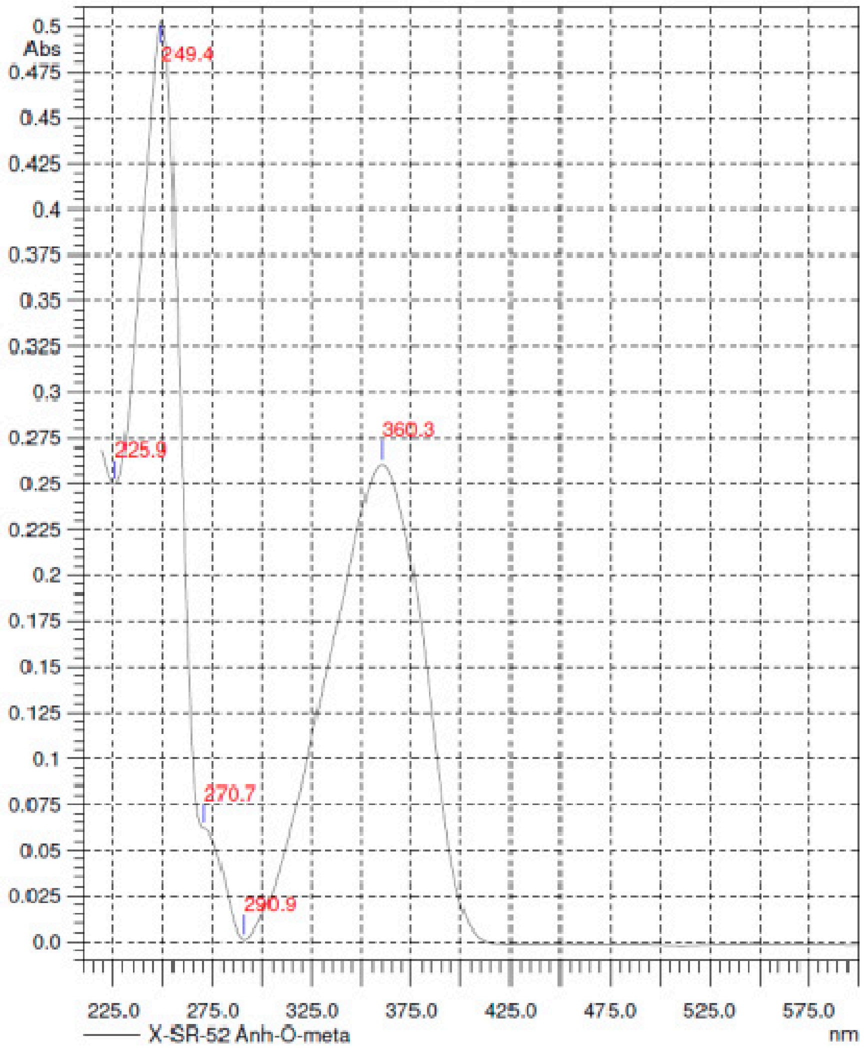

Figure S97. UV spectrum of **23.**

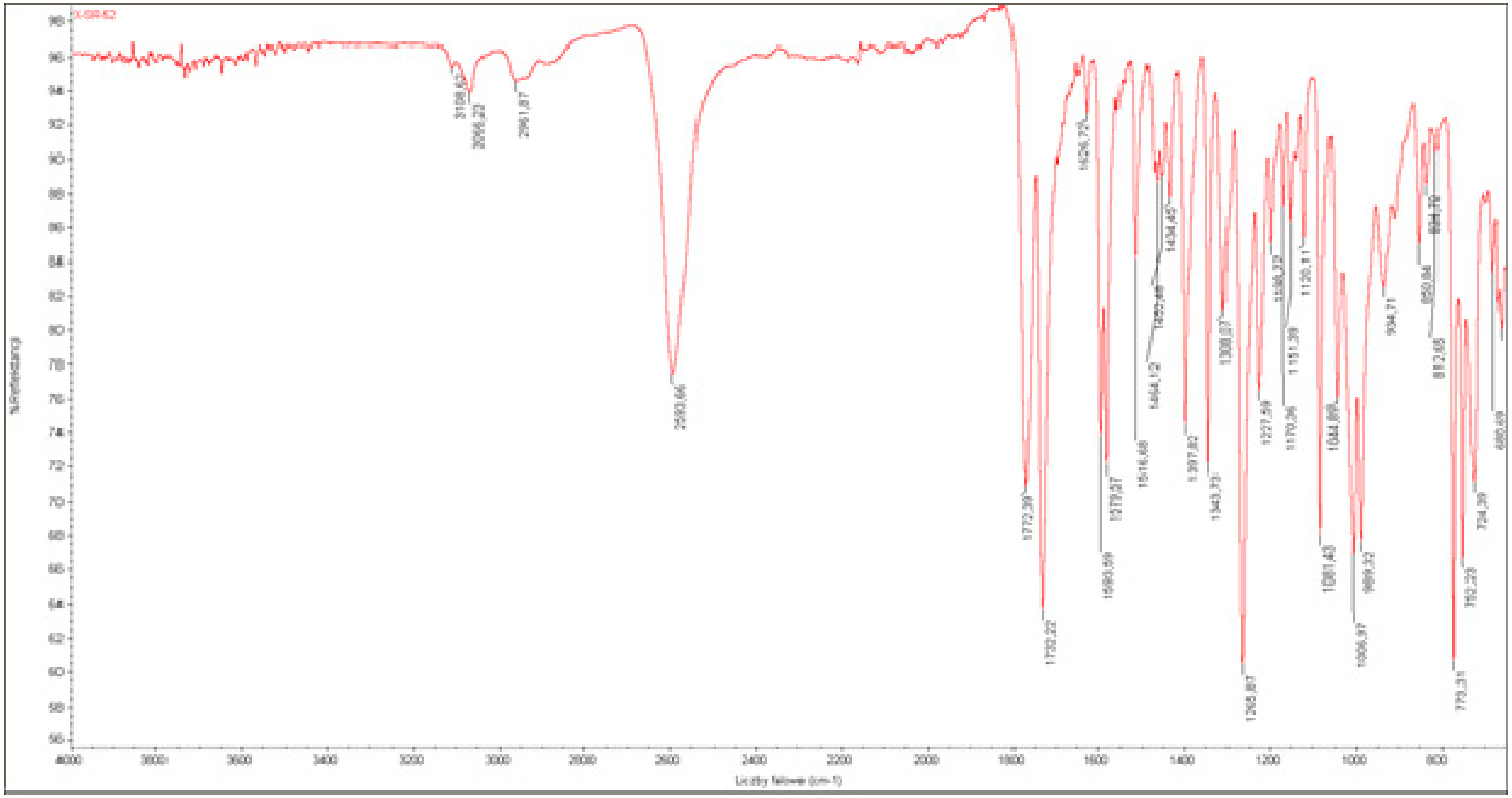

Figure S98. IR spectrum of **23**.

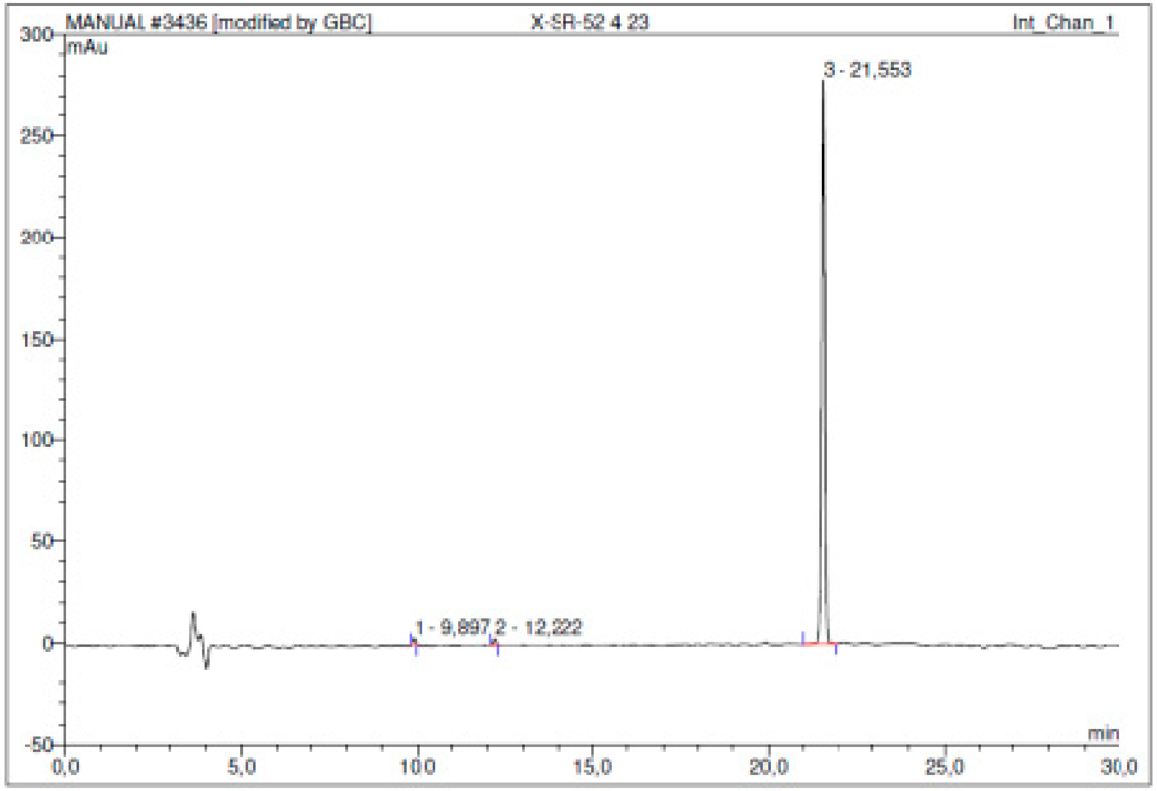

No.	Ret.Time min	Peak Name	Height mAu	Area mAu*min	Rel.Area %	Amount	Type
1	9,90	n.a.	2,787	0,255	0,88	n.a.	BMB*
2	12,22	n.a.	2,744	0,240	0,83	n.a.	BMB*
3	21,55	n.a.	277,886	28,573	98,30	n.a.	BMB
Total:			283,417	29,068	100,00	0,000	

Figure S99. HPLC chromatogram of **23**.

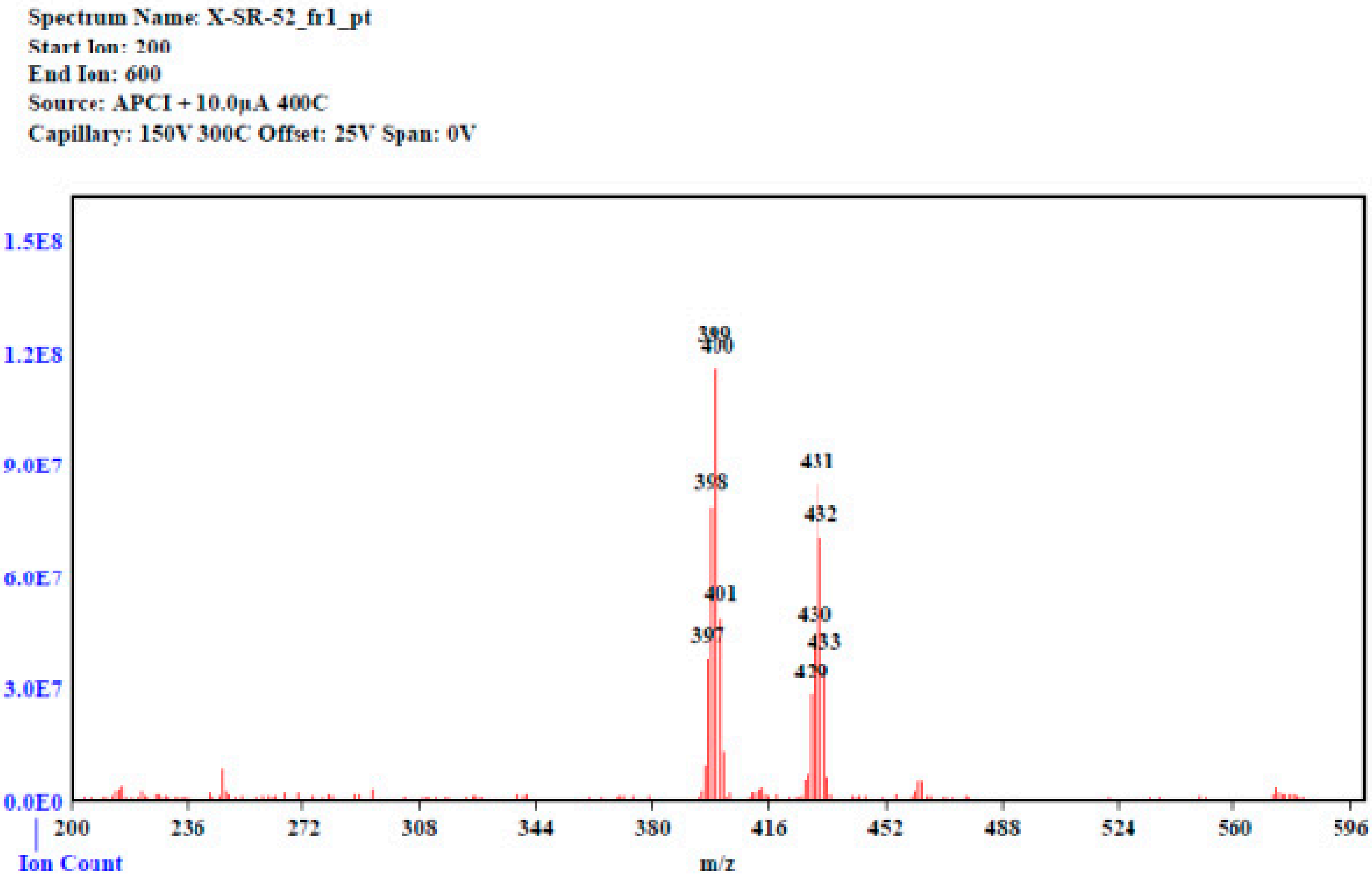

Figure S100. MS spectrum of **23**.

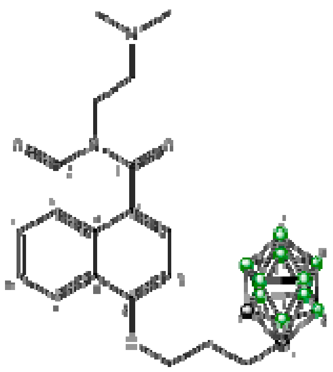

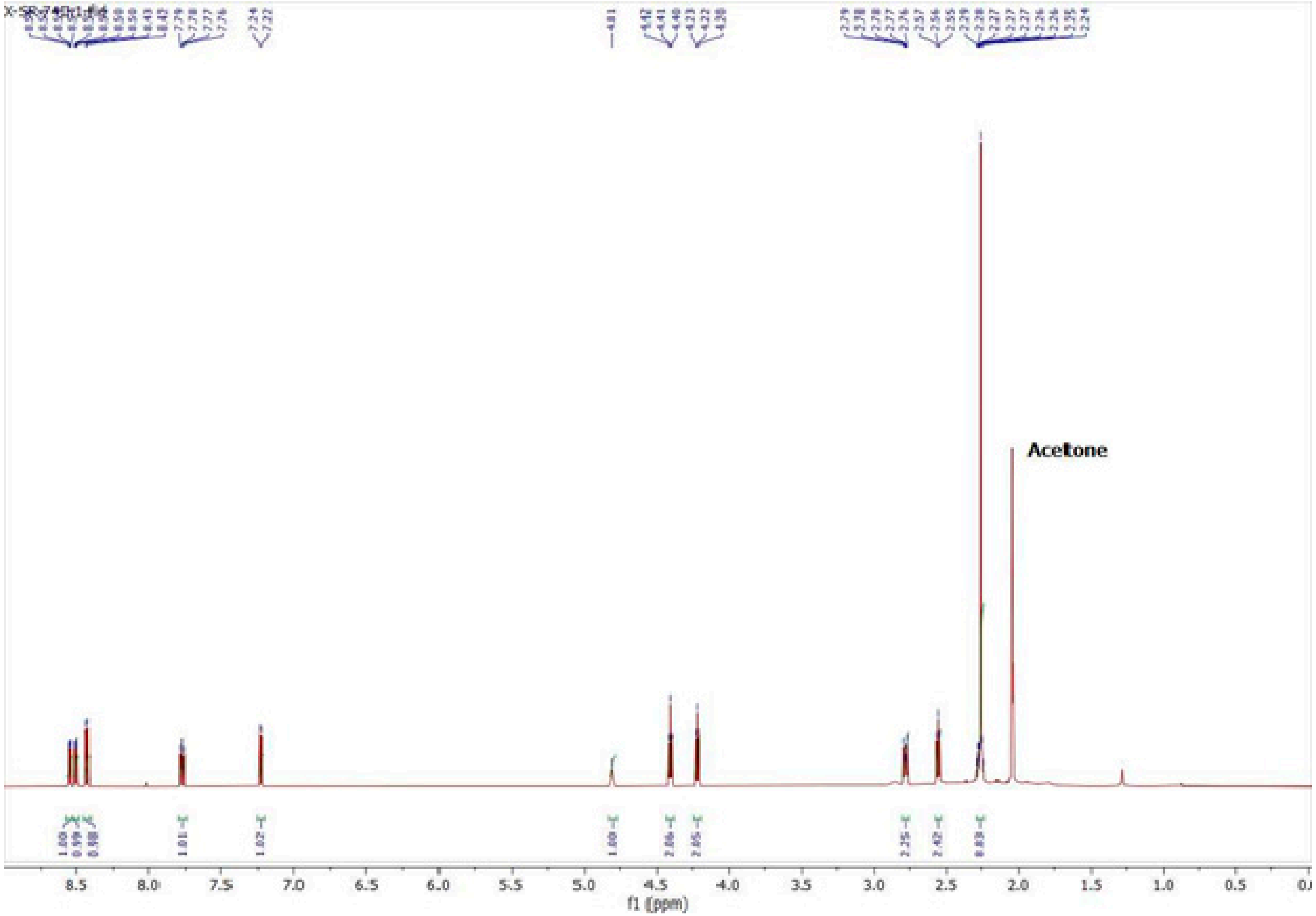

Figure S101. ^{1}H NMR spectrum of **24**.

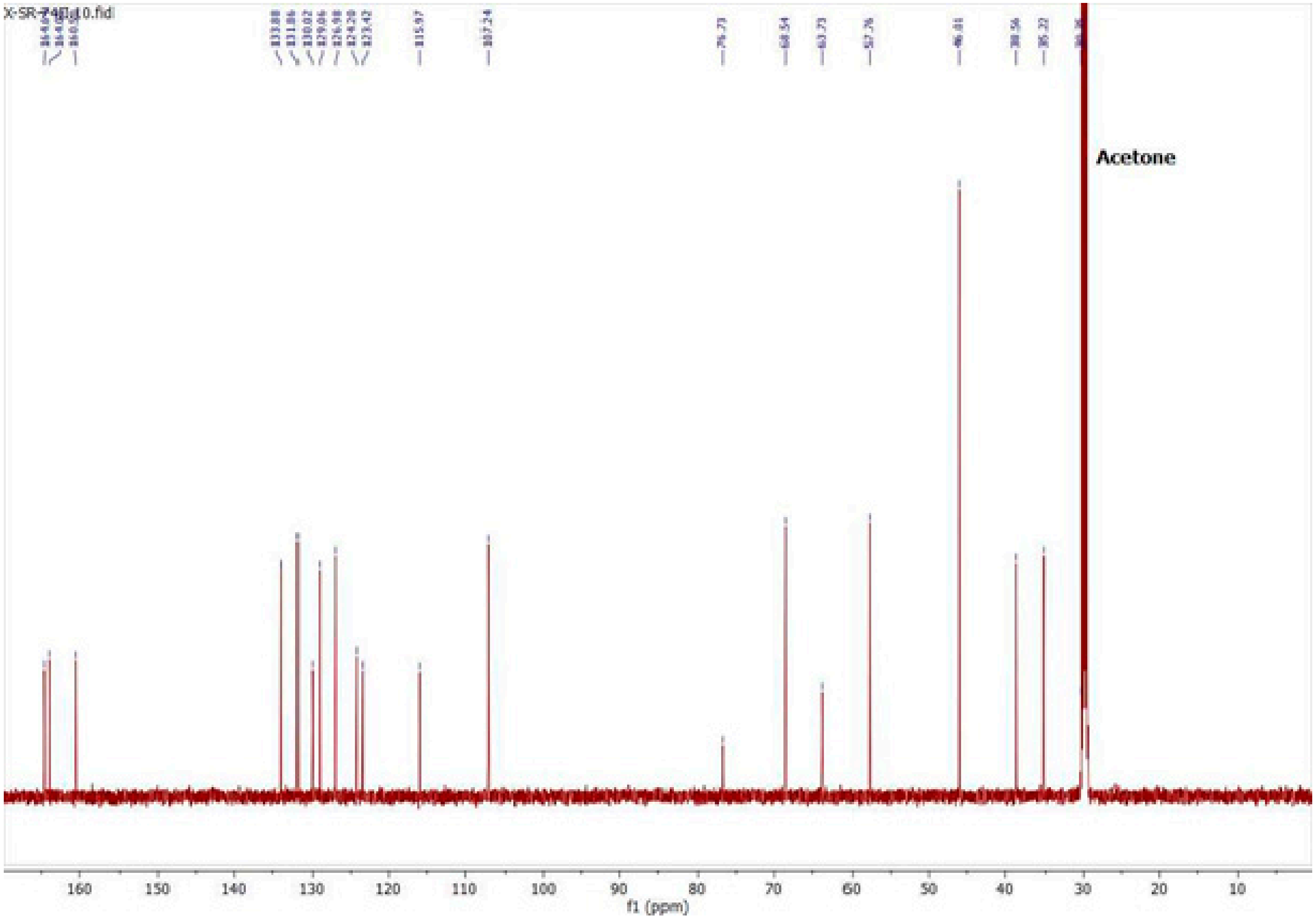

Figure S102. ^{13}C NMR spectrum of **24**.

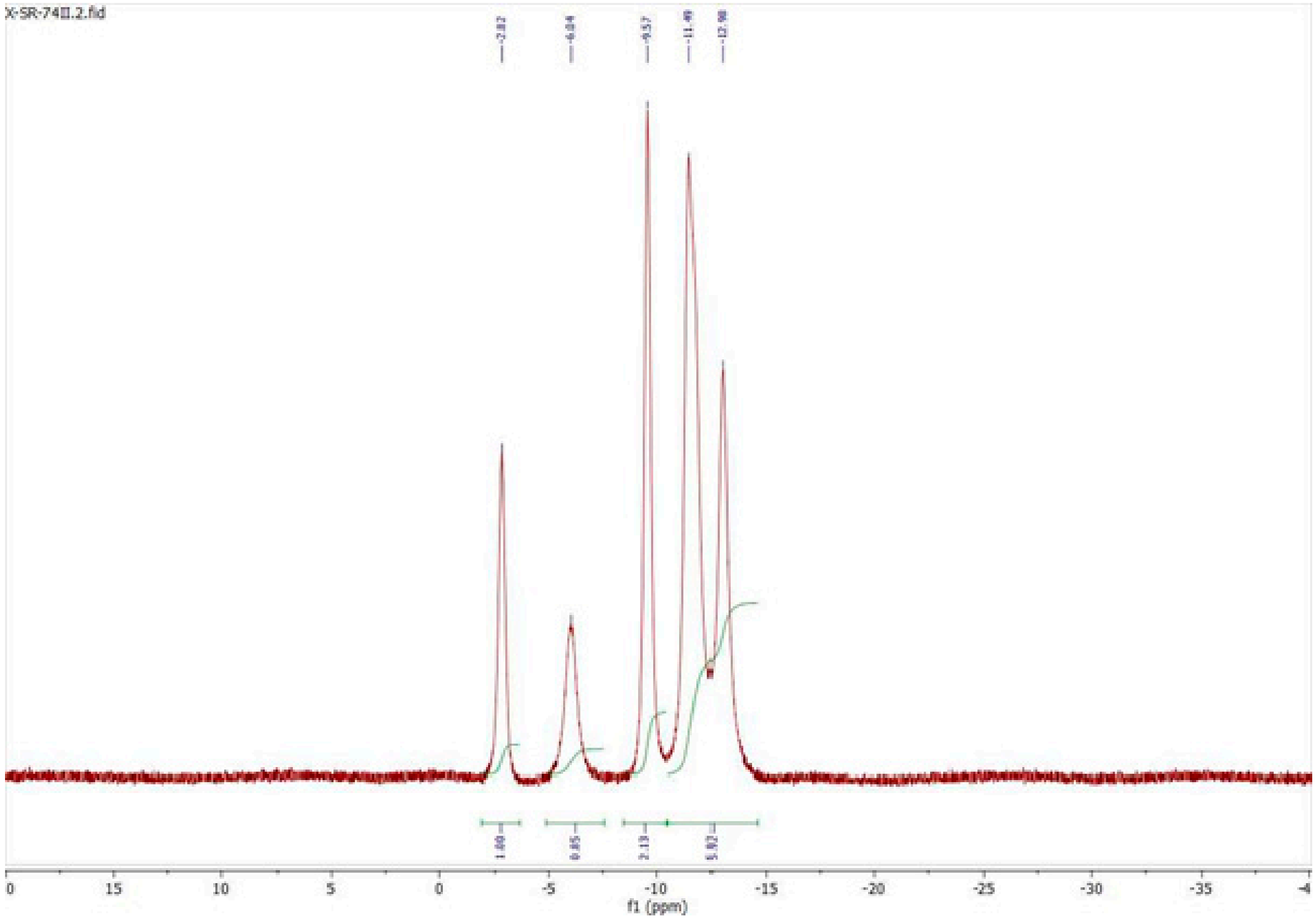

Figure S103. ^{11}B NMR spectrum of **24**.

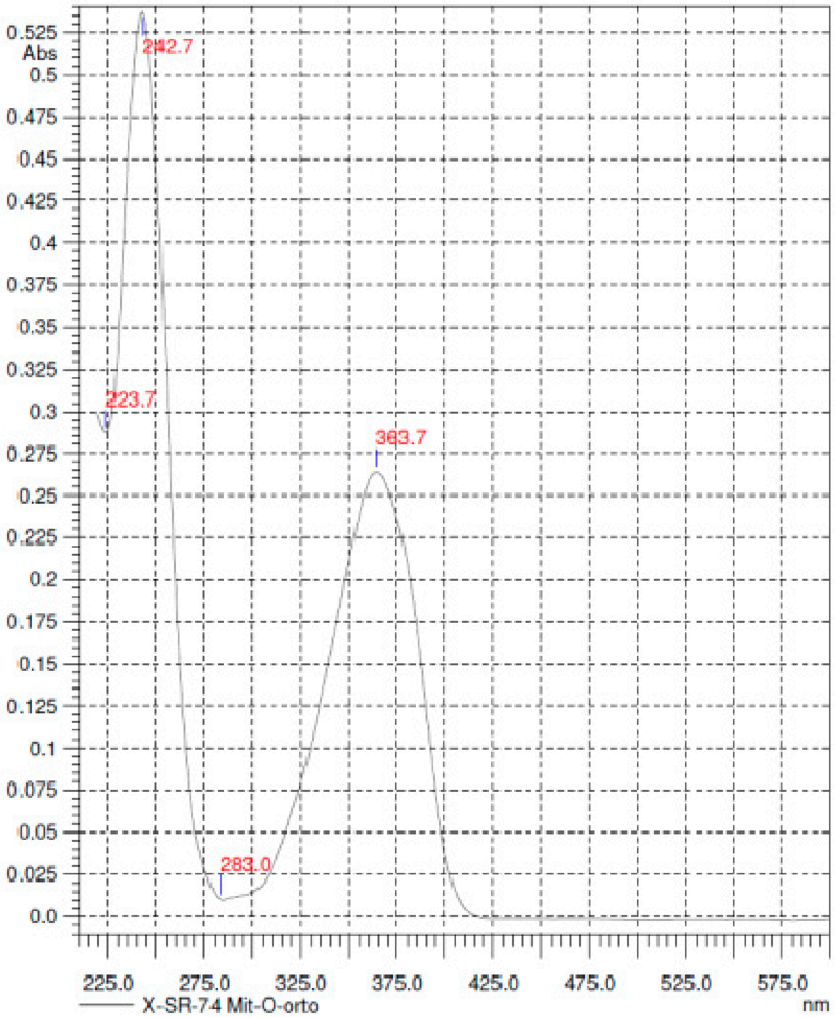

Figure S104. UV spectrum of **24**.

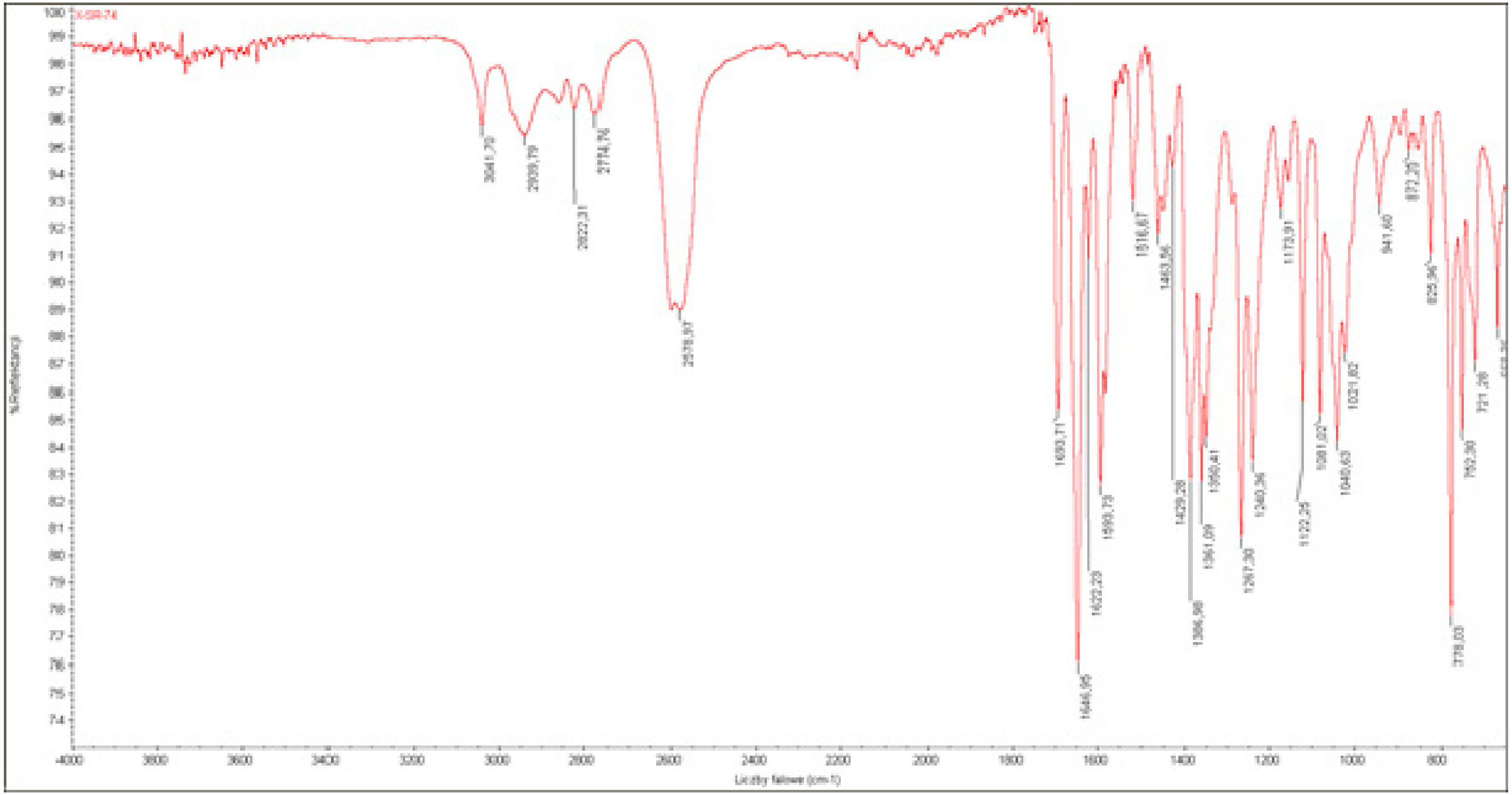

Figure S105. IR spectrum of **24**.

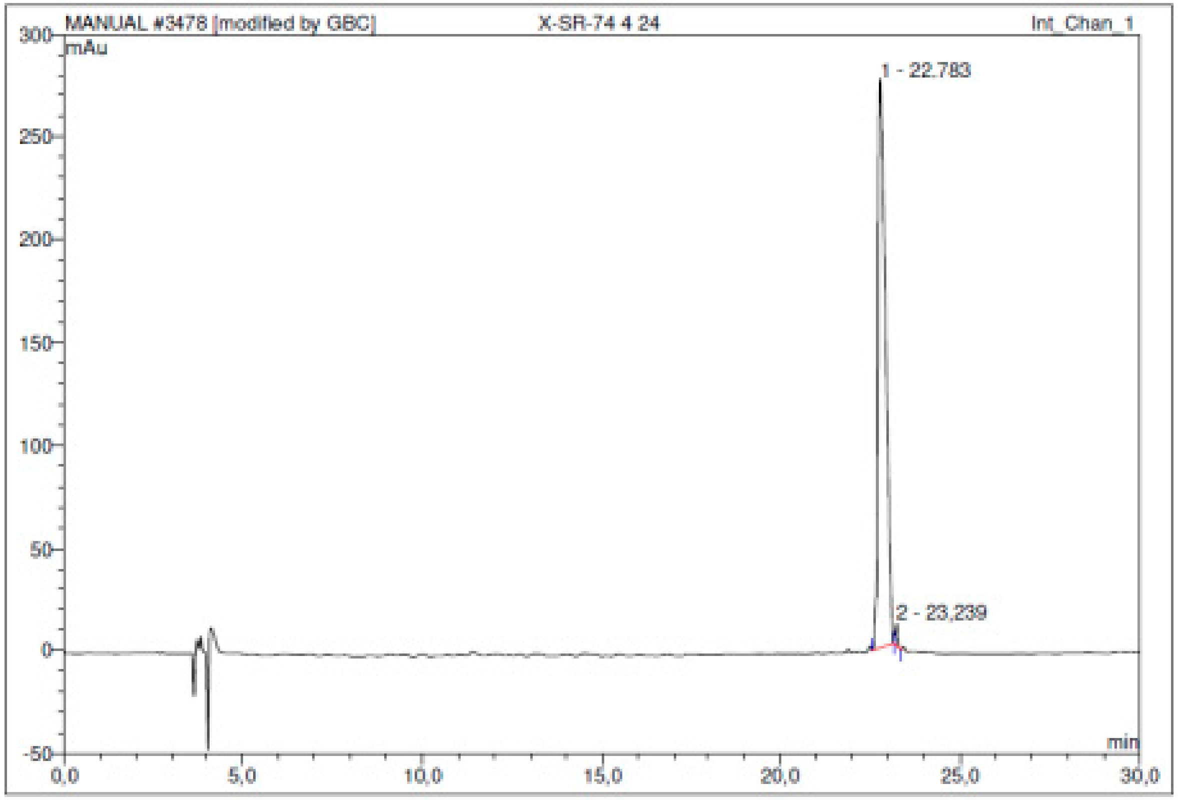

No.	Ret.Time min	Peak Name	Height mAu	Area mAu*min	Rel.Area %	Amount	Type
1	22,78	n.a.	276,901	66,956	98,87	n.a.	BMB
2	23,24	n.a.	10,489	0,762	1,13	n.a.	BMB*
Total:			287,390	67,718	100,00	0,000	

Figure S106. HPLC chromatogram of **24**.

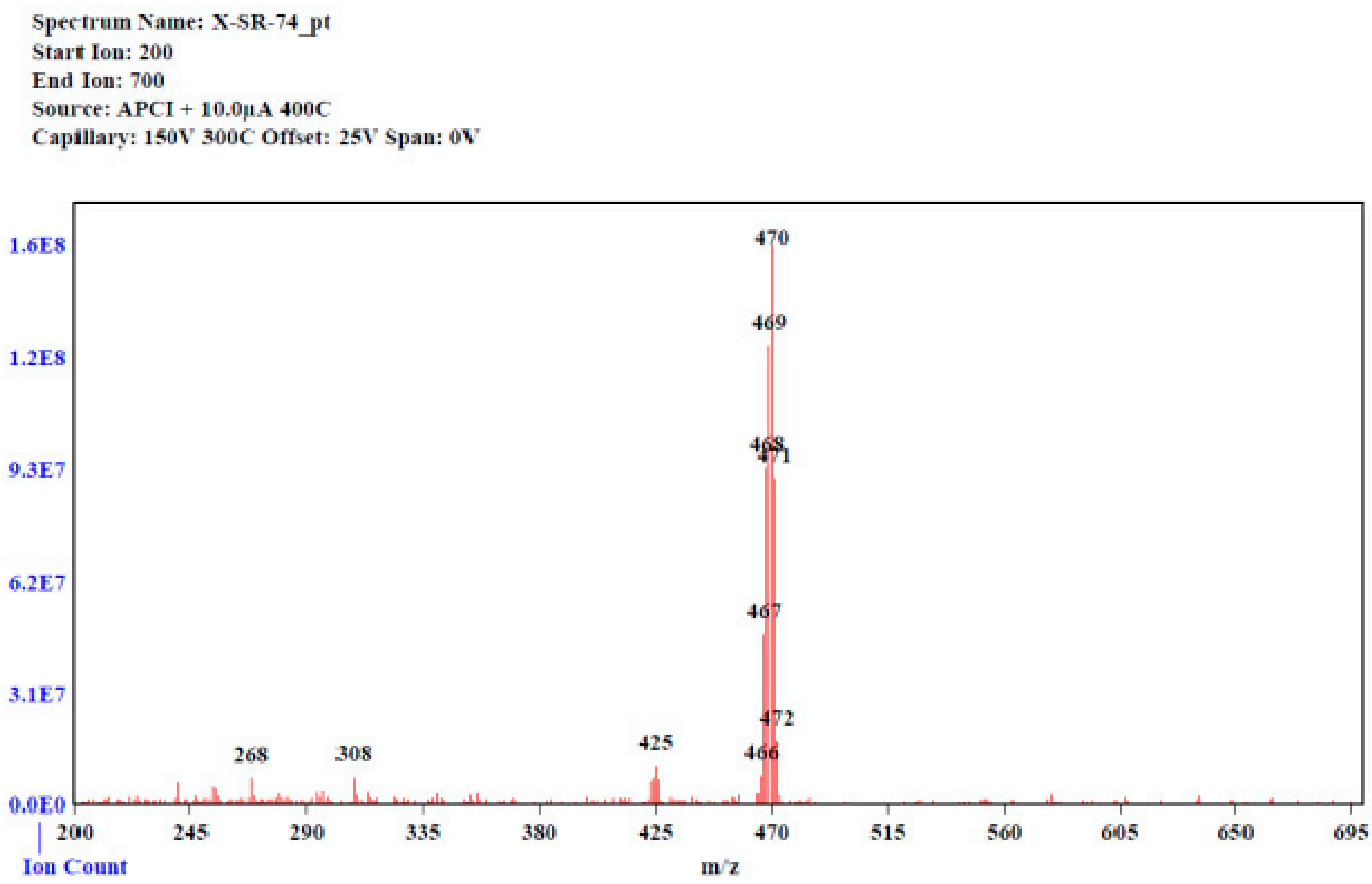

Figure S107. MS spectrum of **24**.

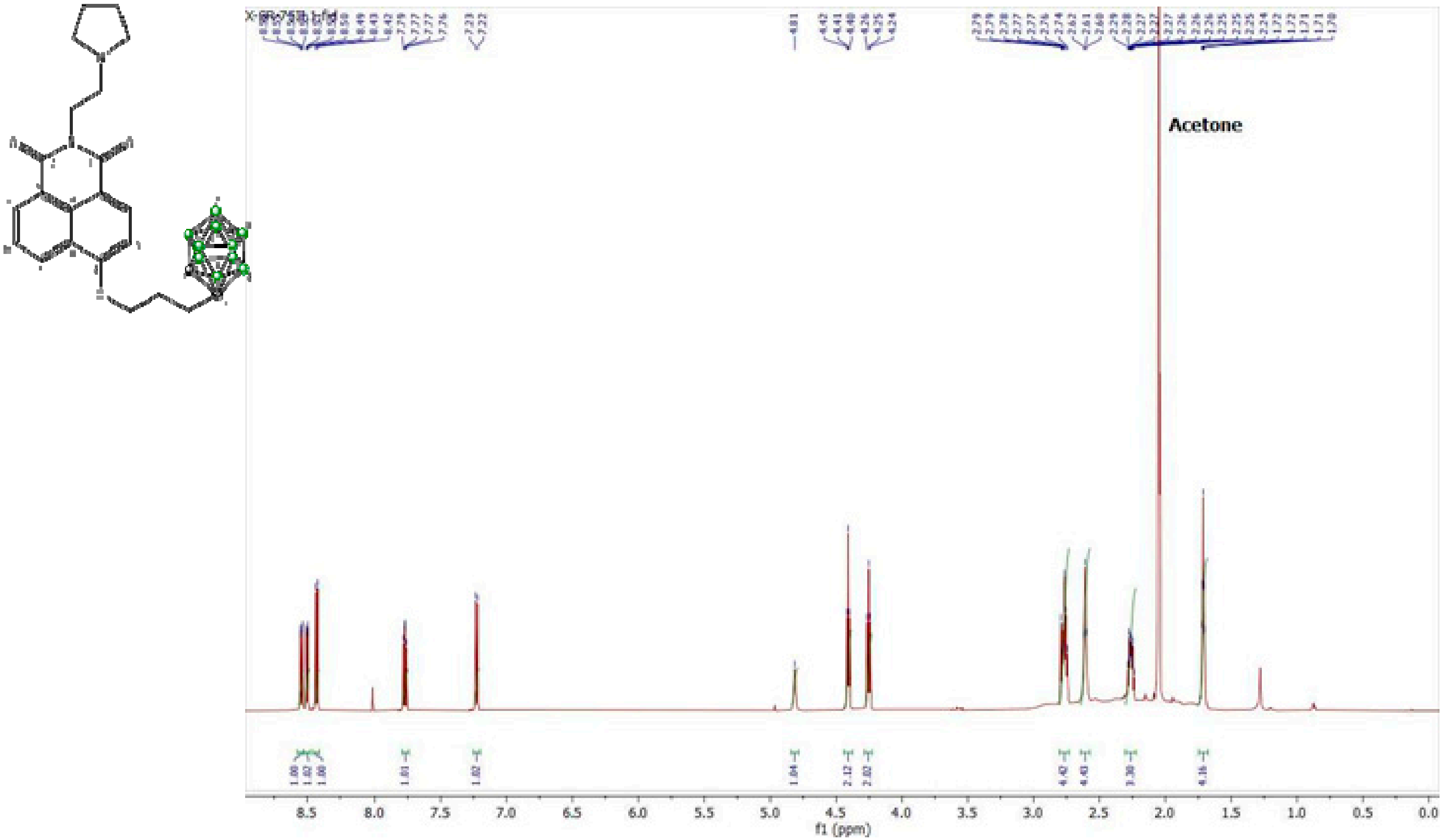

Figure S108. ^{1}H NMR spectrum of **25**.

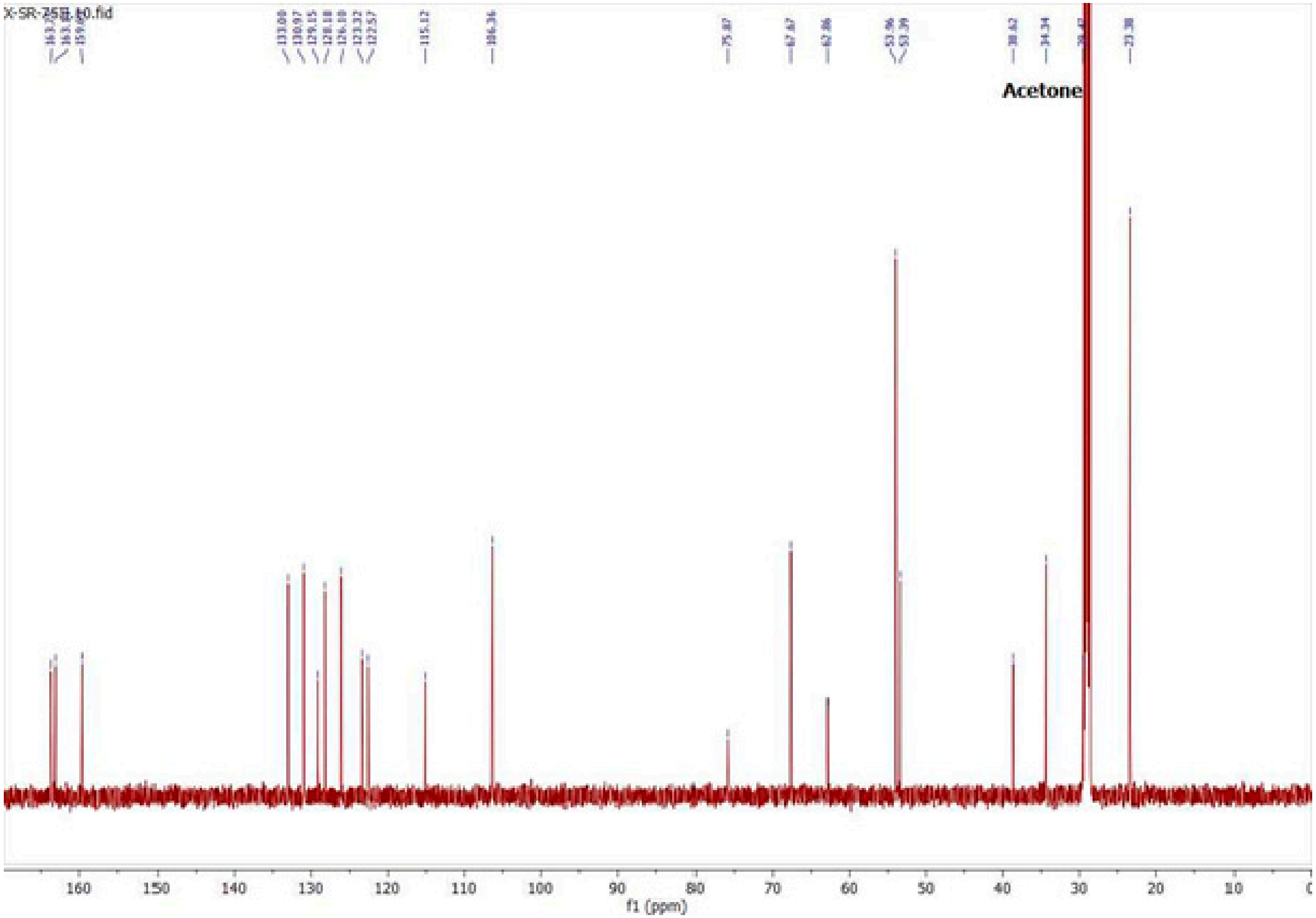

Figure S109. ^{13}C NMR spectrum of **25**.

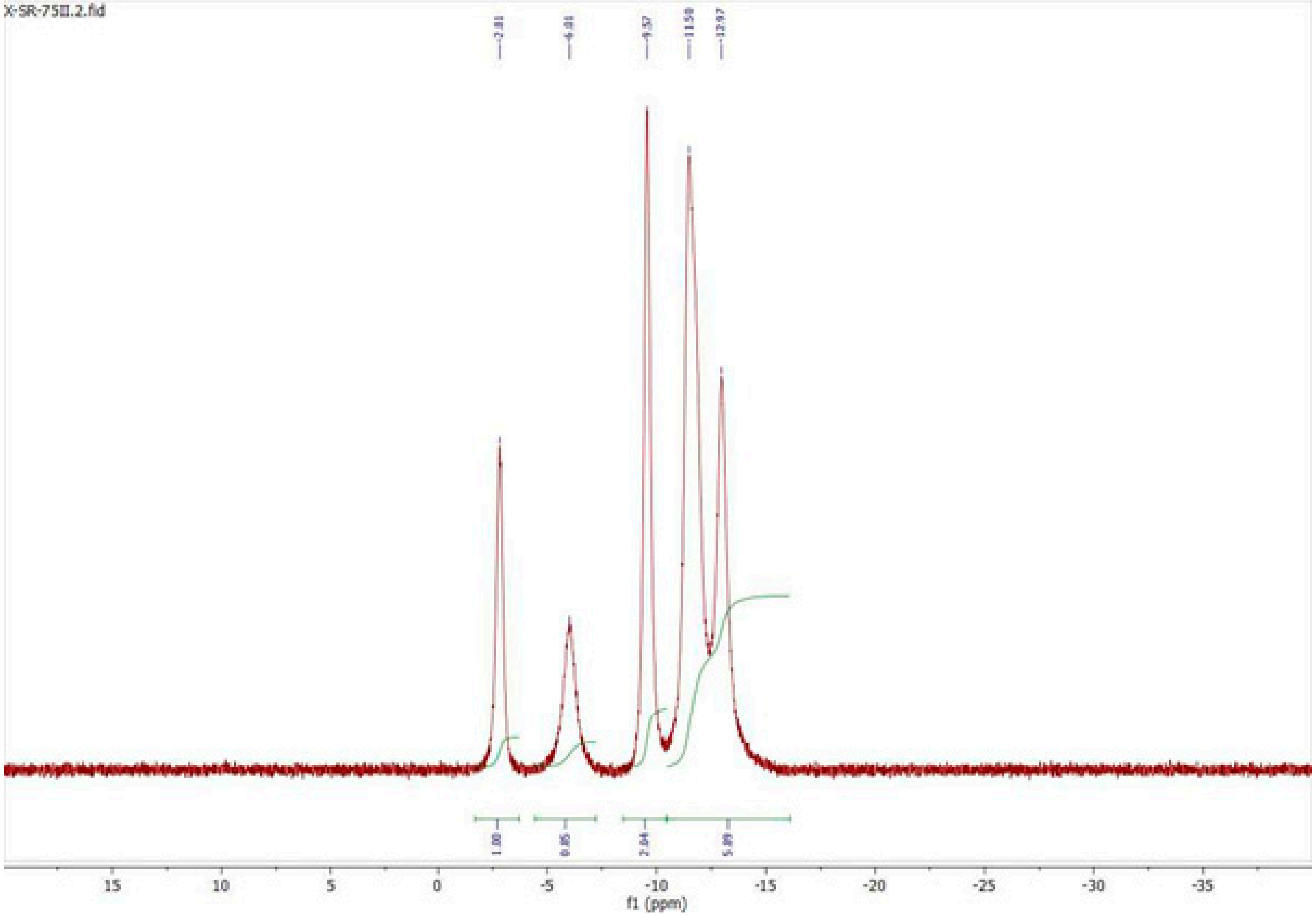

Figure S110. ^{11}B NMR spectrum of **25**.

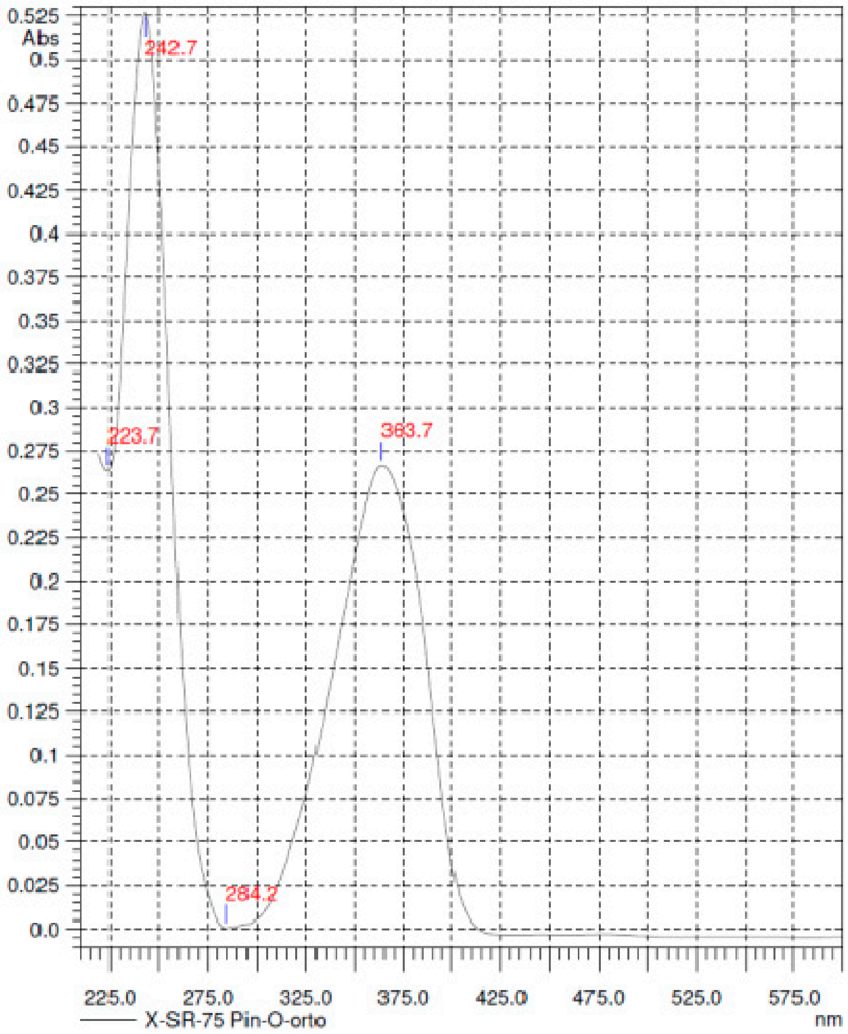

Figure S111. UV spectrum of **16.**

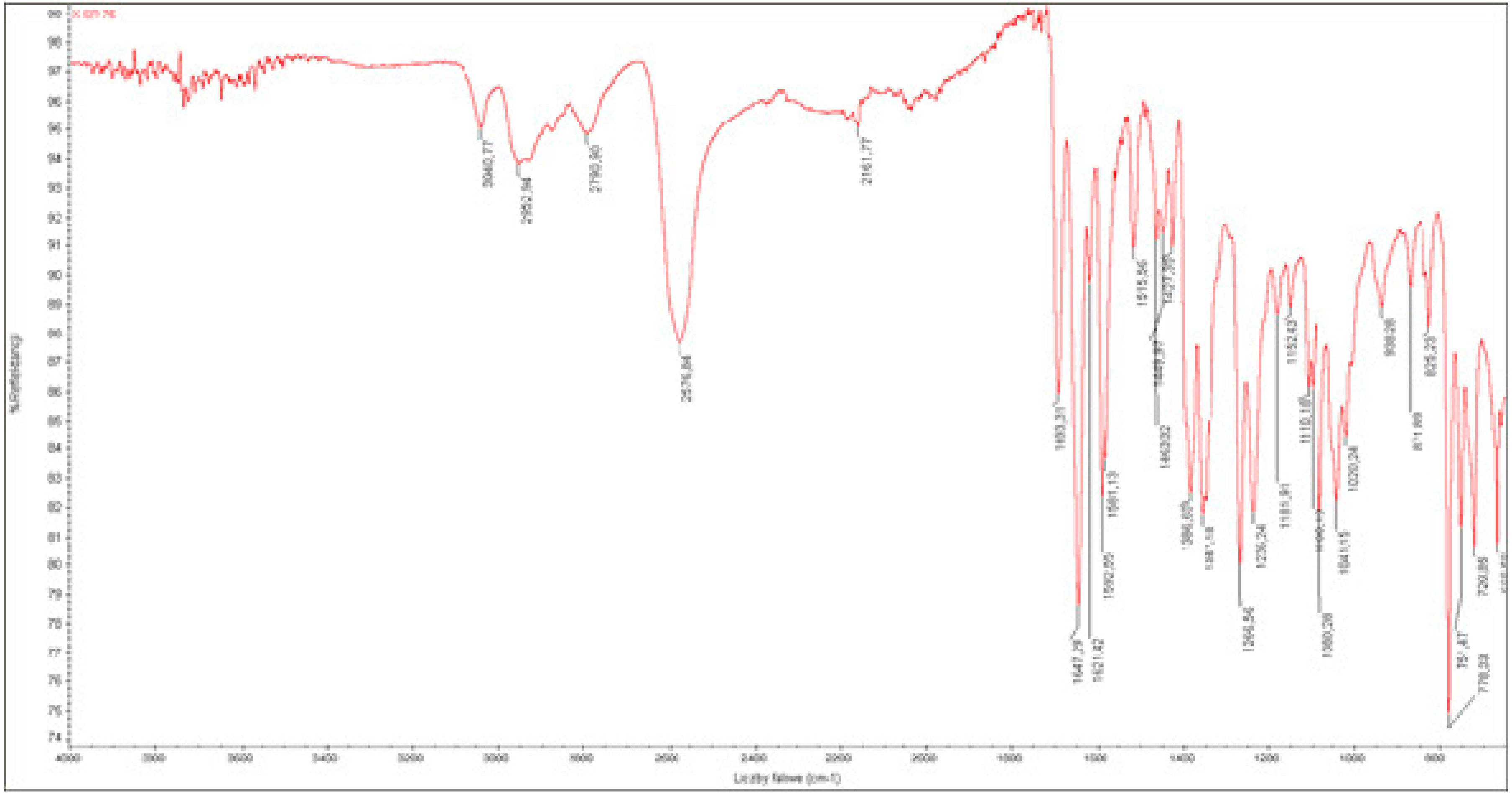

Figure S112. IR spectrum of **25**.

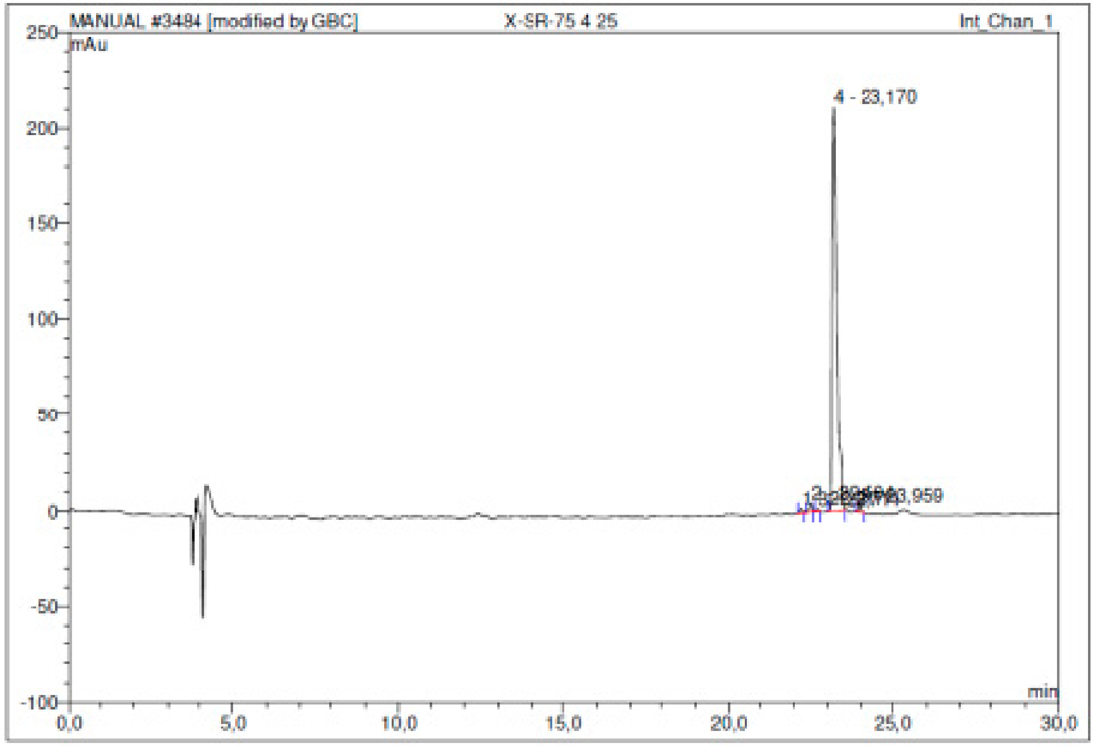

No.	Ret.Time min	Peak Name	Height mAu	Area mAu*min	Rel.Area %	Amount	Type
1	22,22	n.a.	2,207	0,193	0,55	n.a.	BMB*
2	22,50	n.a.	4,564	0,443	1,26	n.a.	BMB*
3	22,71	n.a.	1,379	0,119	0,34	n.a.	BMB*
4	23,17	n.a.	210,322	34,098	97,01	n.a.	BMB
5	23,96	n.a.	3,100	0,297	0,85	n.a.	BMB*
Total:			221,571	35,150	100,00	0,000	

Figure S113. HPLC chromatogram of **25**.

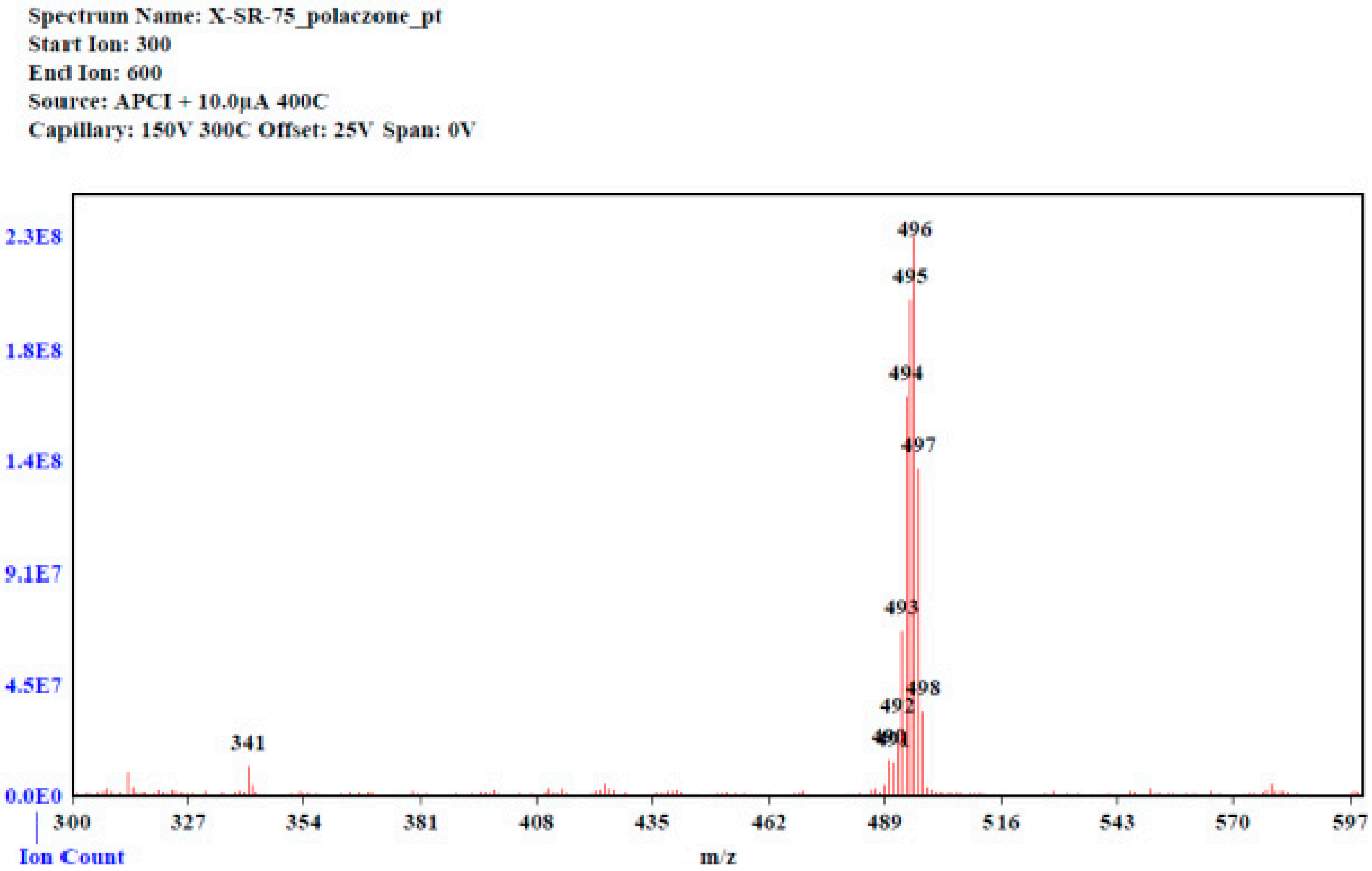

Figure S114. MS spectrum of **25**.

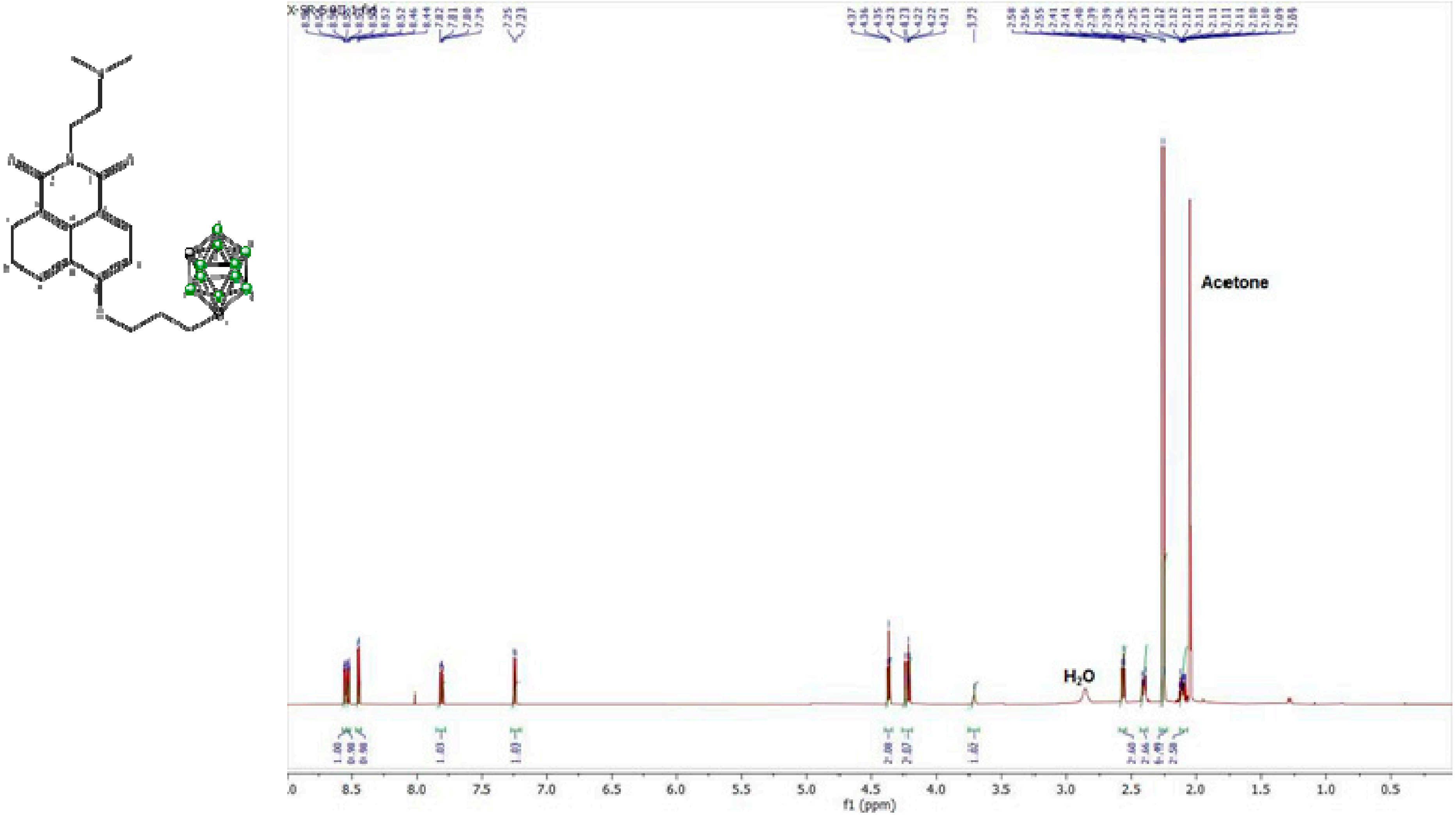

Figure S115. ^{1}H NMR spectrum of **26**.

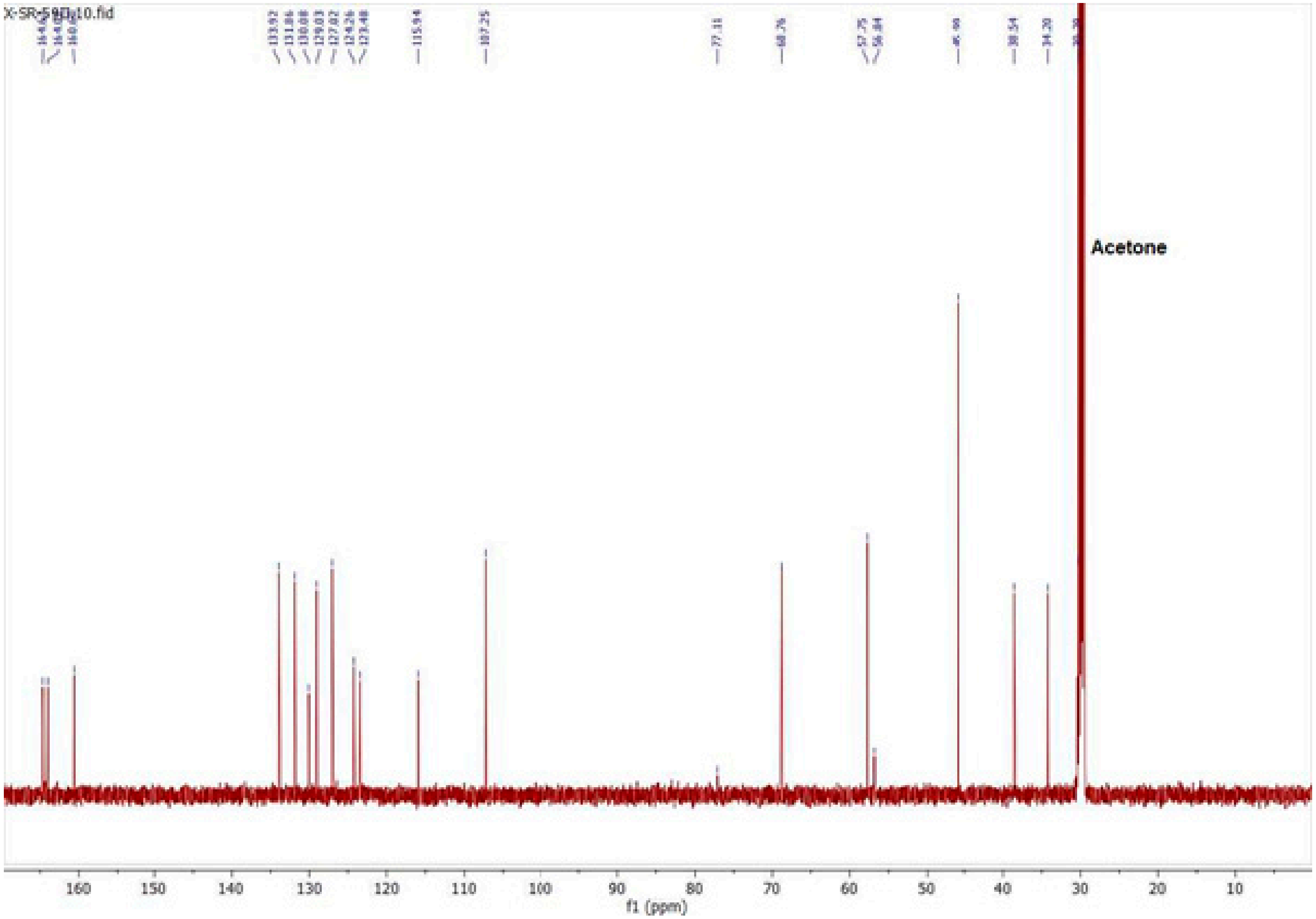

Figure S116. ^{13}C NMR spectrum of **26**.

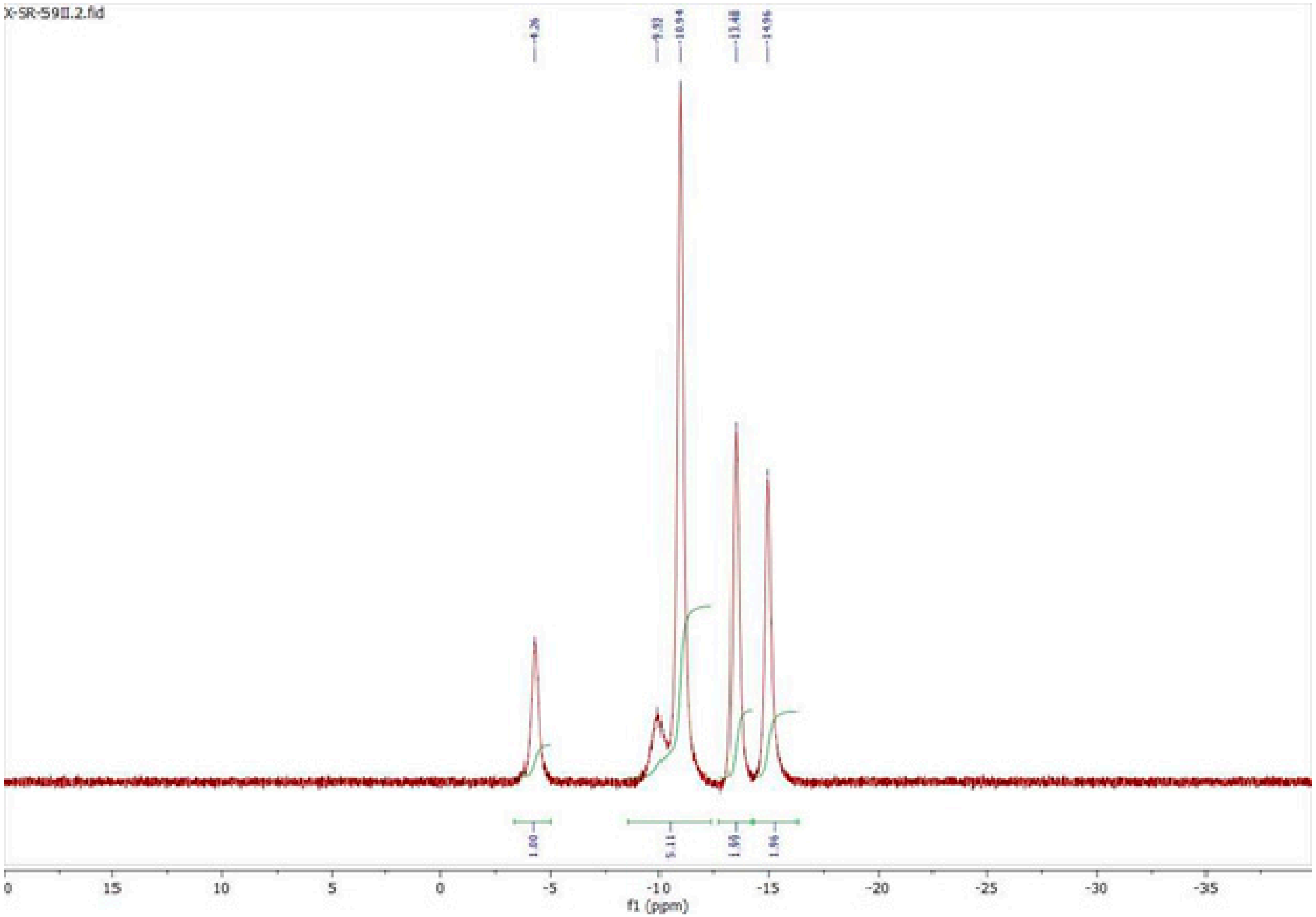

Figure S117. ^{11}B NMR spectrum of **26**.

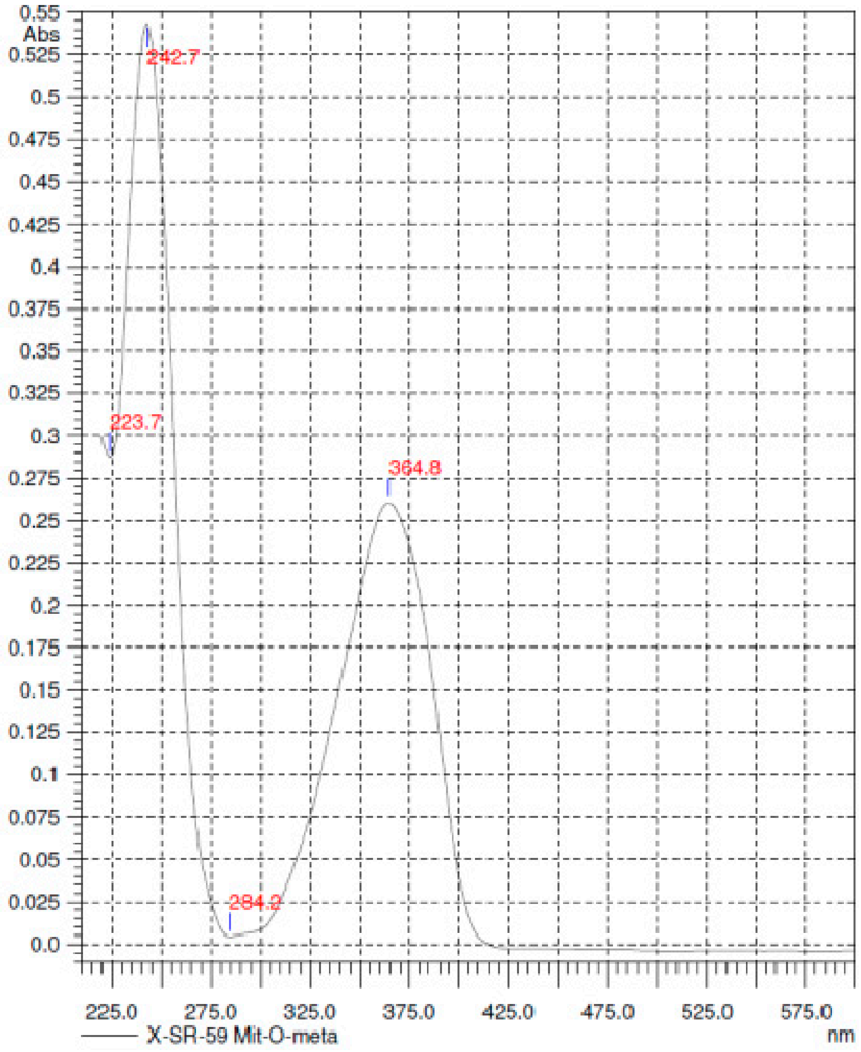

Figure S118. UV spectrum of **26**.

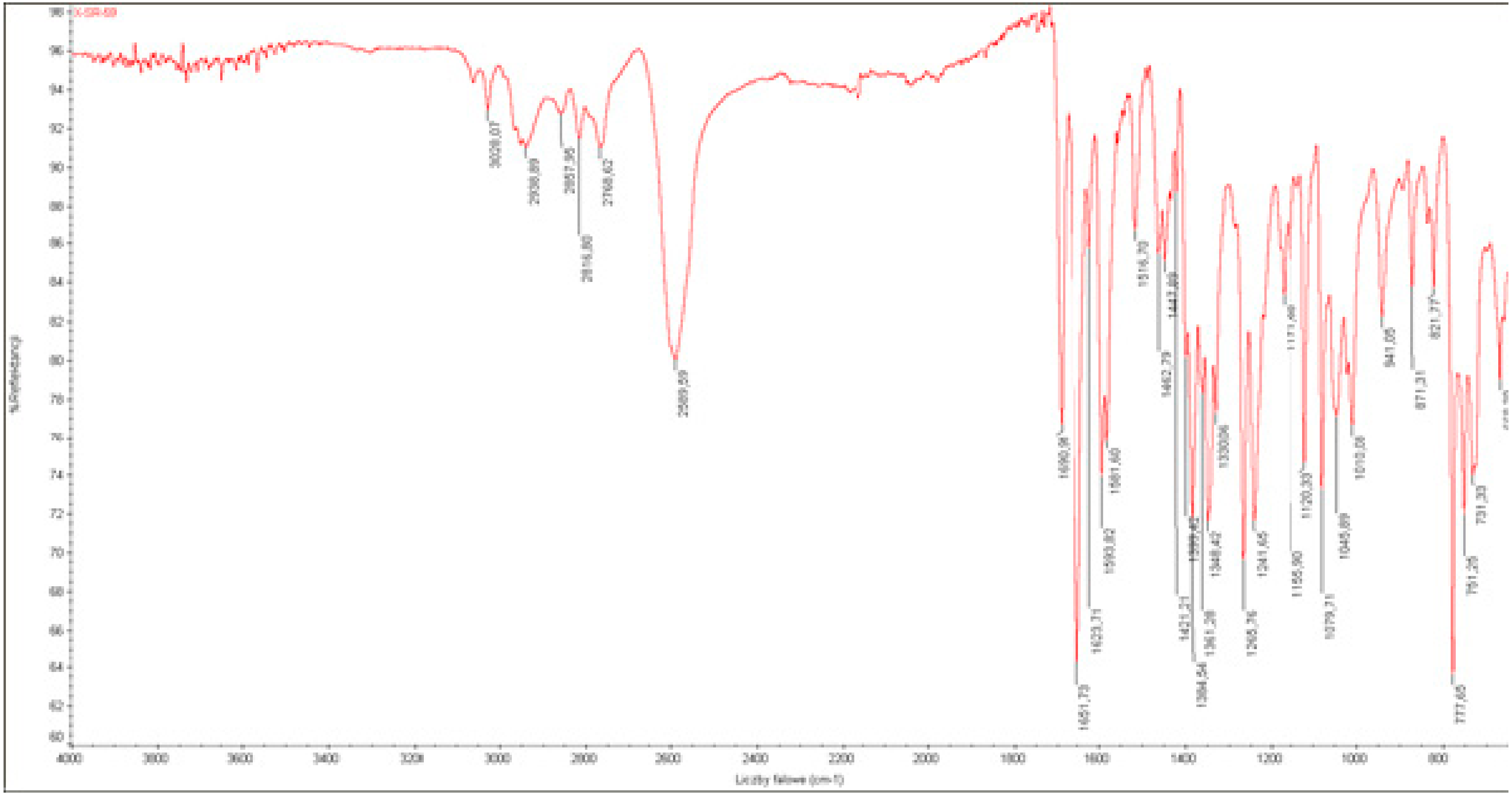

Figure S119. IR spectrum of **26**.

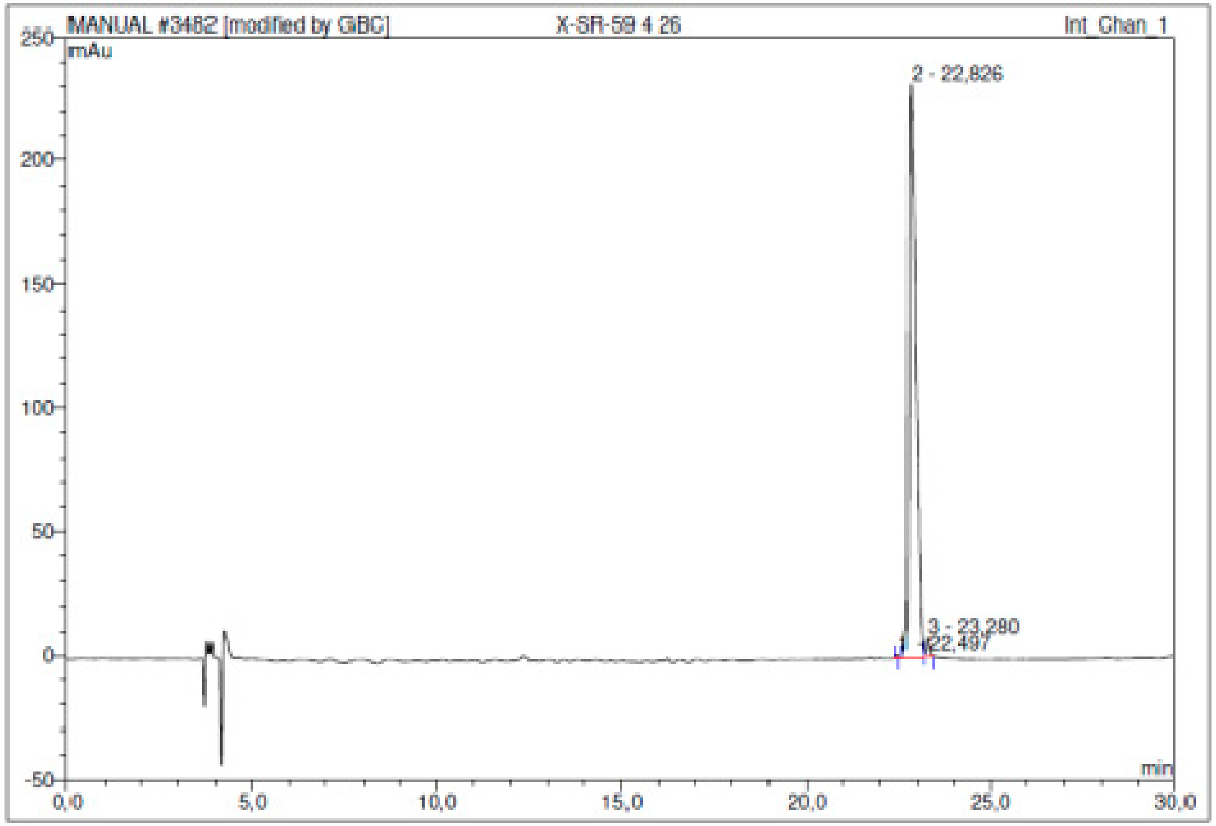

No.	Ret.Time min	Peak Name	Height mAu	Area mAu*min	Rel.Area %	Amount	Type
1	22,50	n.a.	1,183	0,081	0,17	n.a.	BMB*
2	22,83	n.a.	232,029	46,918	98,10	n.a.	BM *
3	23,28	n.a.	8,011	0,826	1,73	n.a.	MB*
Total:			241,223	47,824	100,00	0,000	

Figure S120. HPLC chromatogram of **26**.

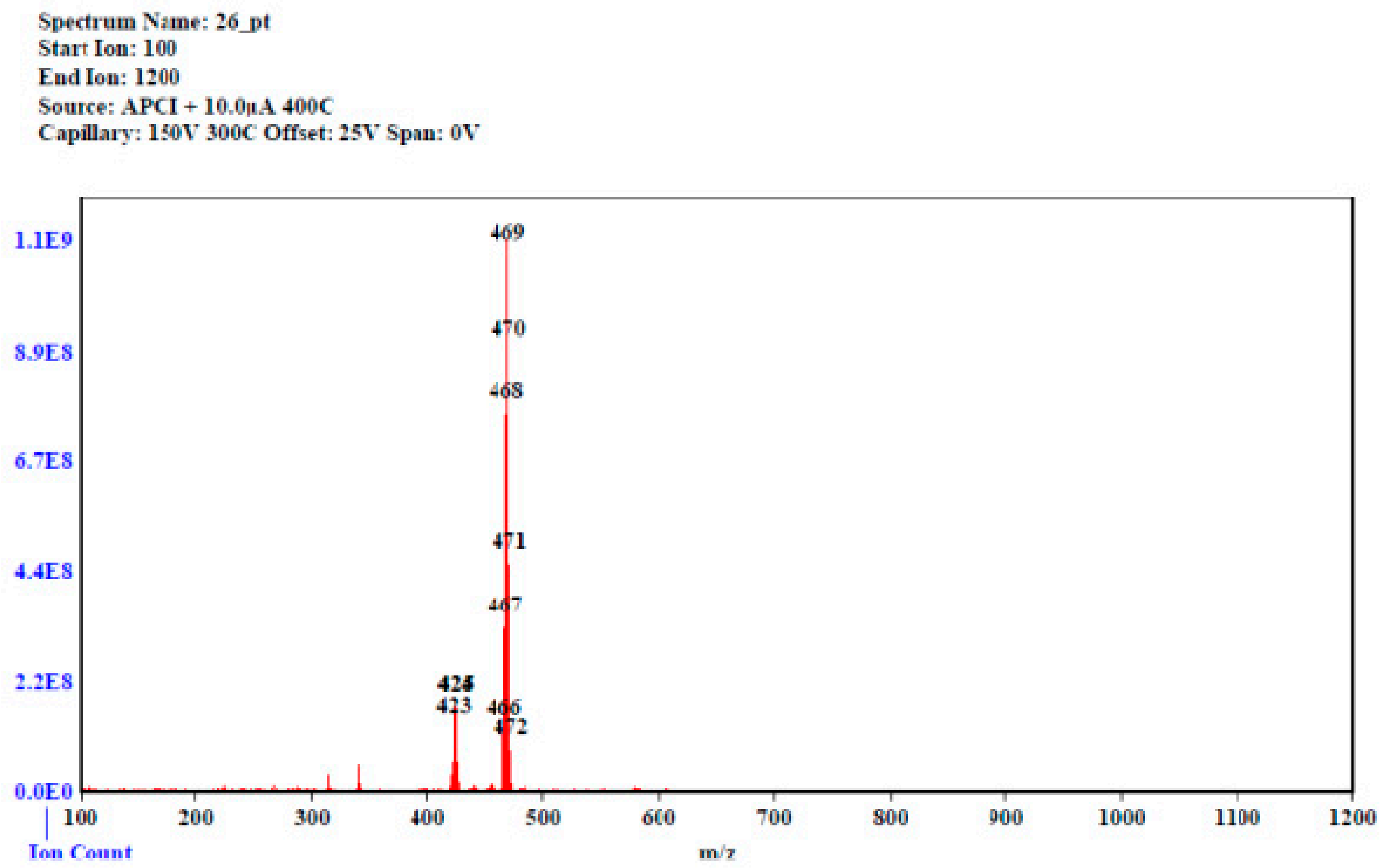

Figure S121. MS spectrum of **26**.

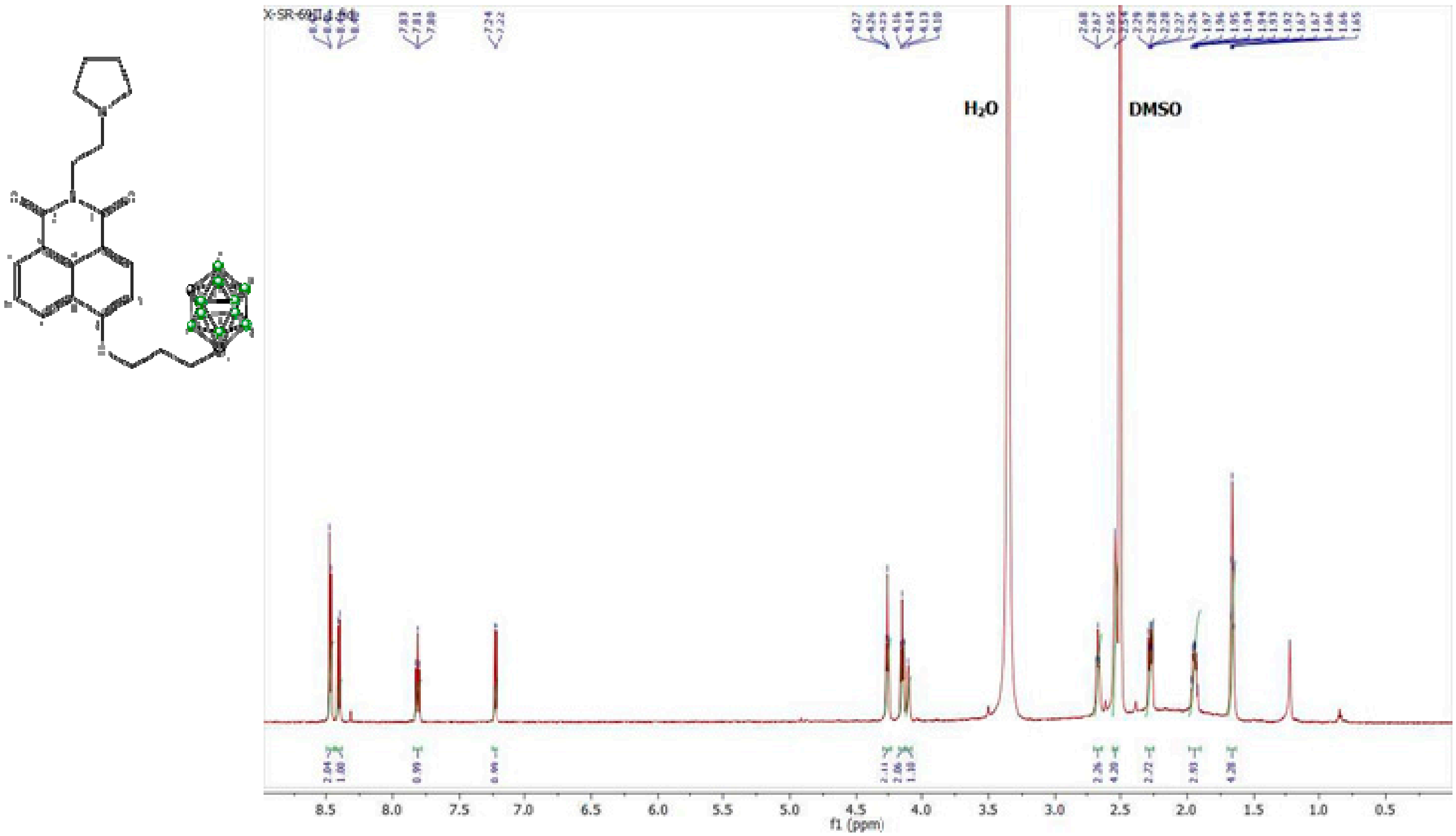

Figure S122. ^{1}H NMR spectrum of **27**.

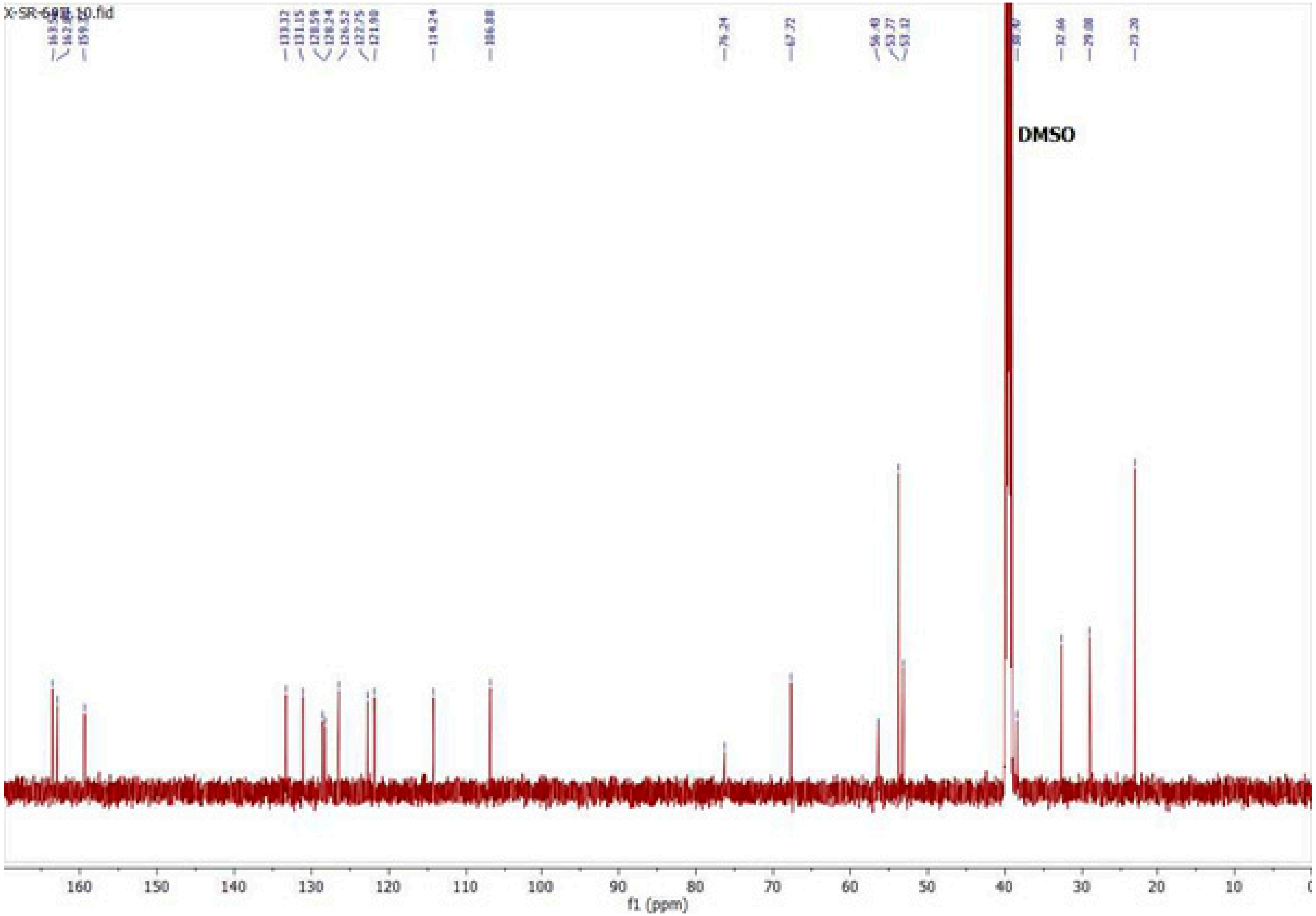

Figure S123. ^{13}C NMR spectrum of **27**.

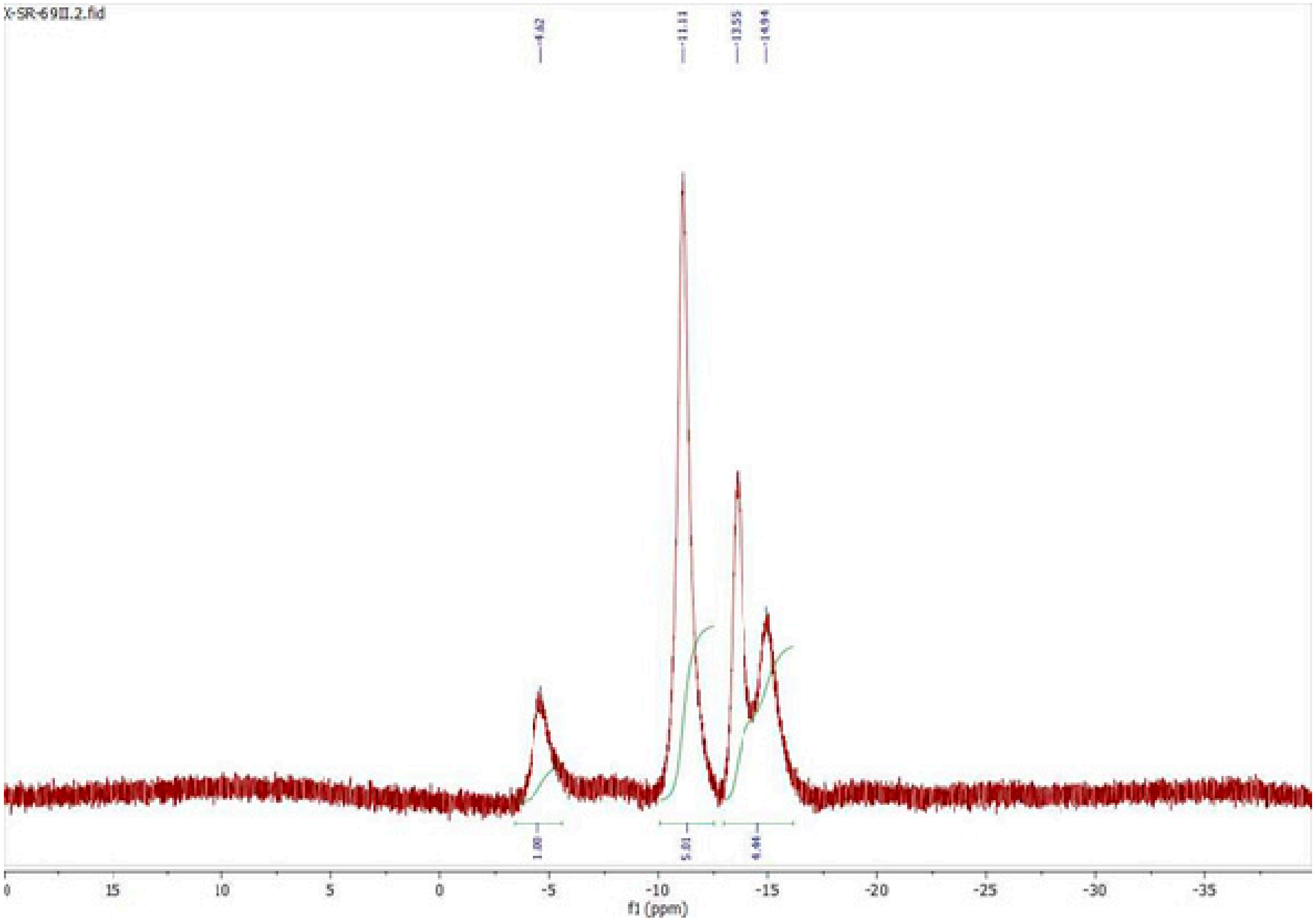

Figure S124. ^{11}B NMR spectrum of **27**.

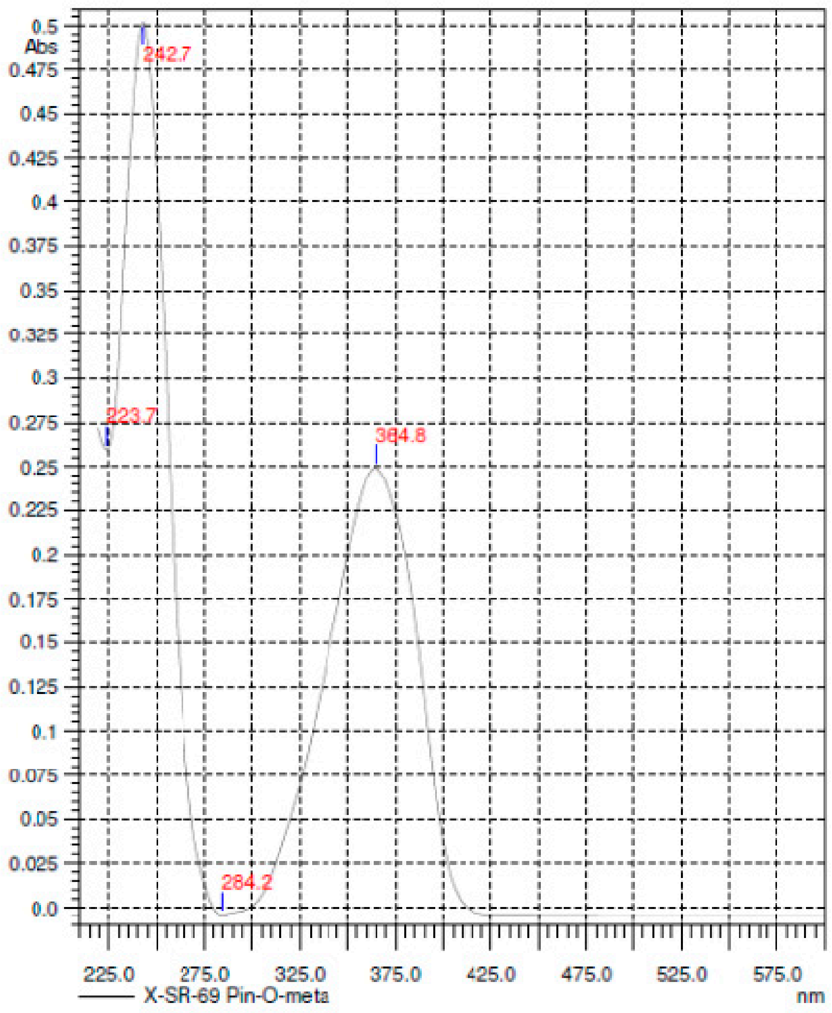

Figure S125. UV spectrum of **27.**

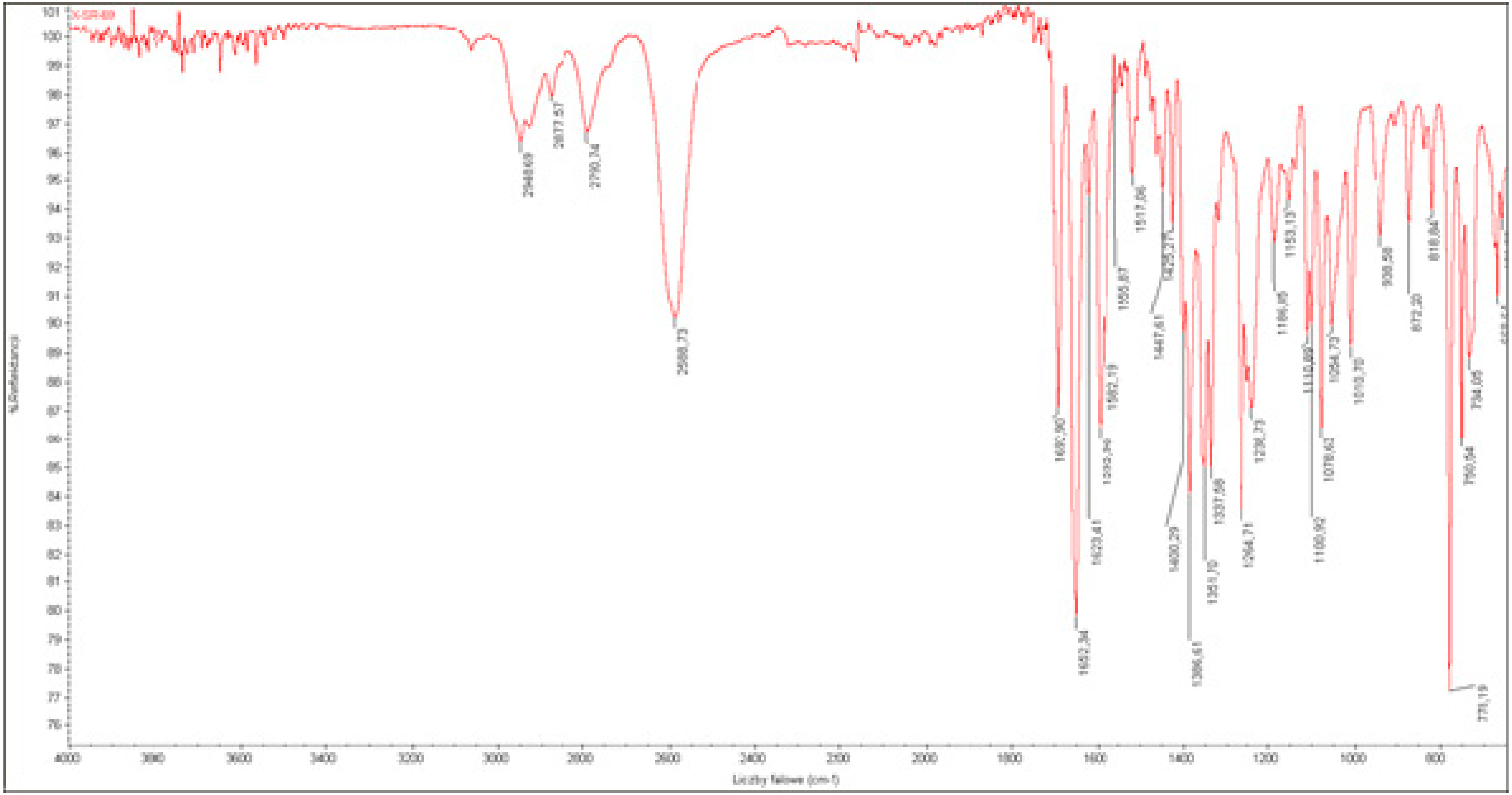

Figure S126. IR spectrum of **27**.

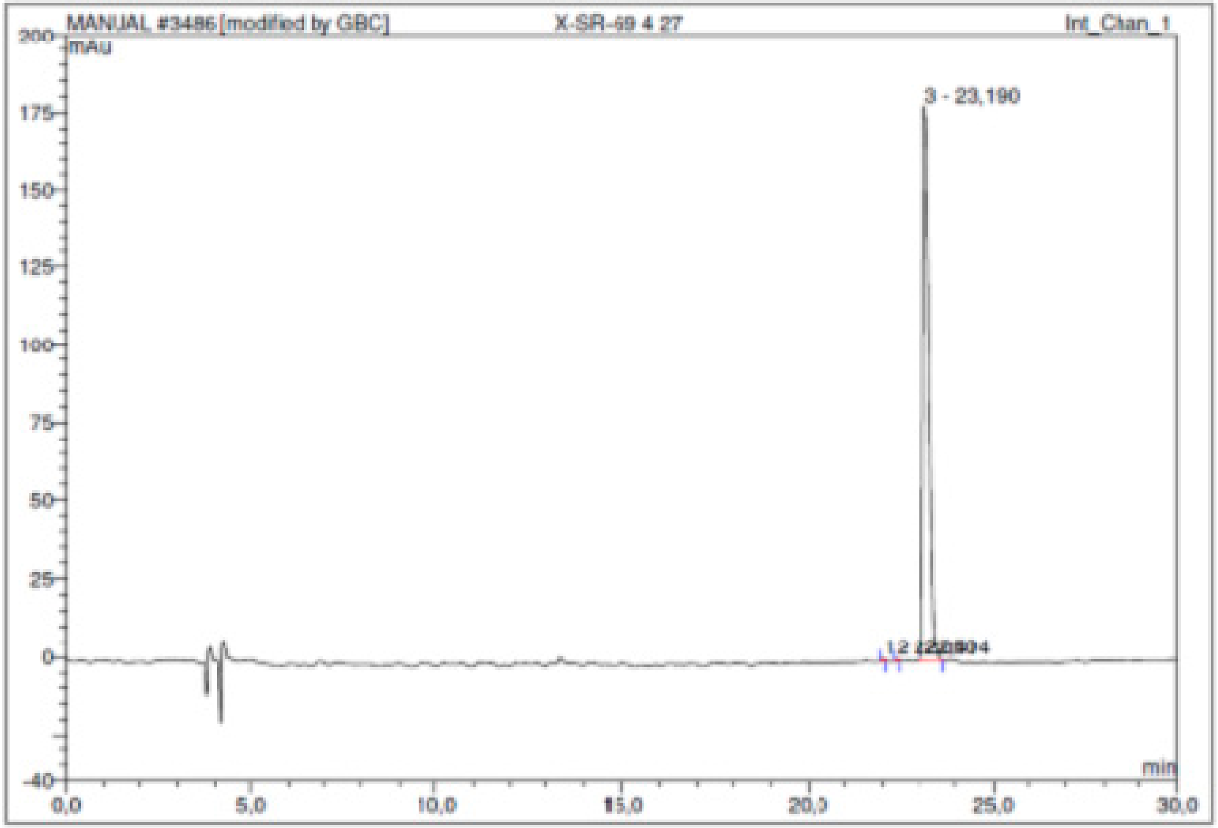

No.	Ret.Time min	Peak Name	Height mAu	Area mAu*min	Rel.Area %	Amount	Type
1	22,05	n.a.	0,709	0,055	0,16	n.a.	BMB*
2	22,41	n.a.	0,667	0,051	0,15	n.a.	BMB*
3	23,19	n.a.	178,323	33,579	99,68	n.a.	BMB
Total:			179,699	33,685	100,00	0,000	

Figure S127. HPLC chromatogram of **27**.

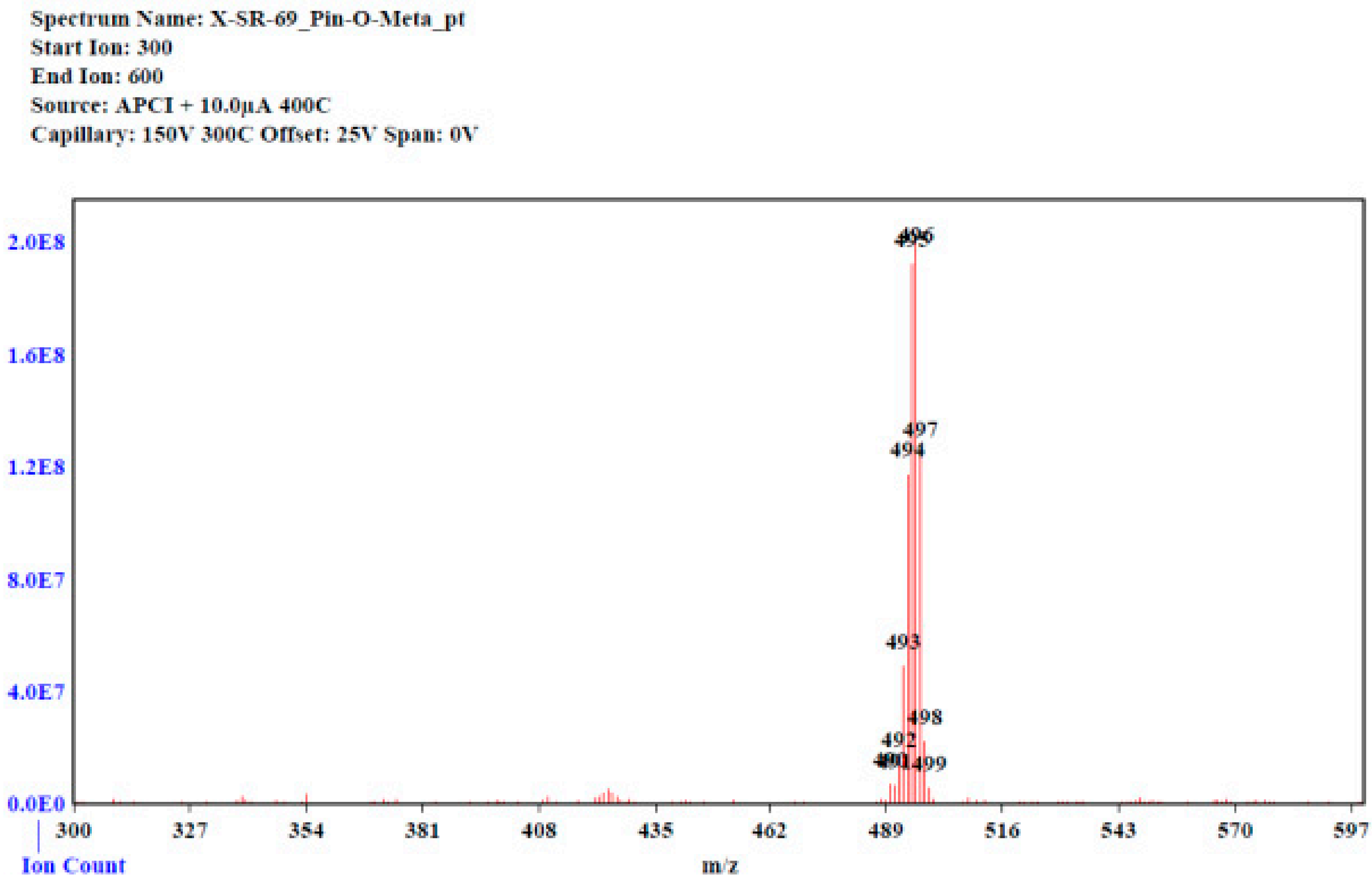

Figure S128. MS spectrum of **27**.

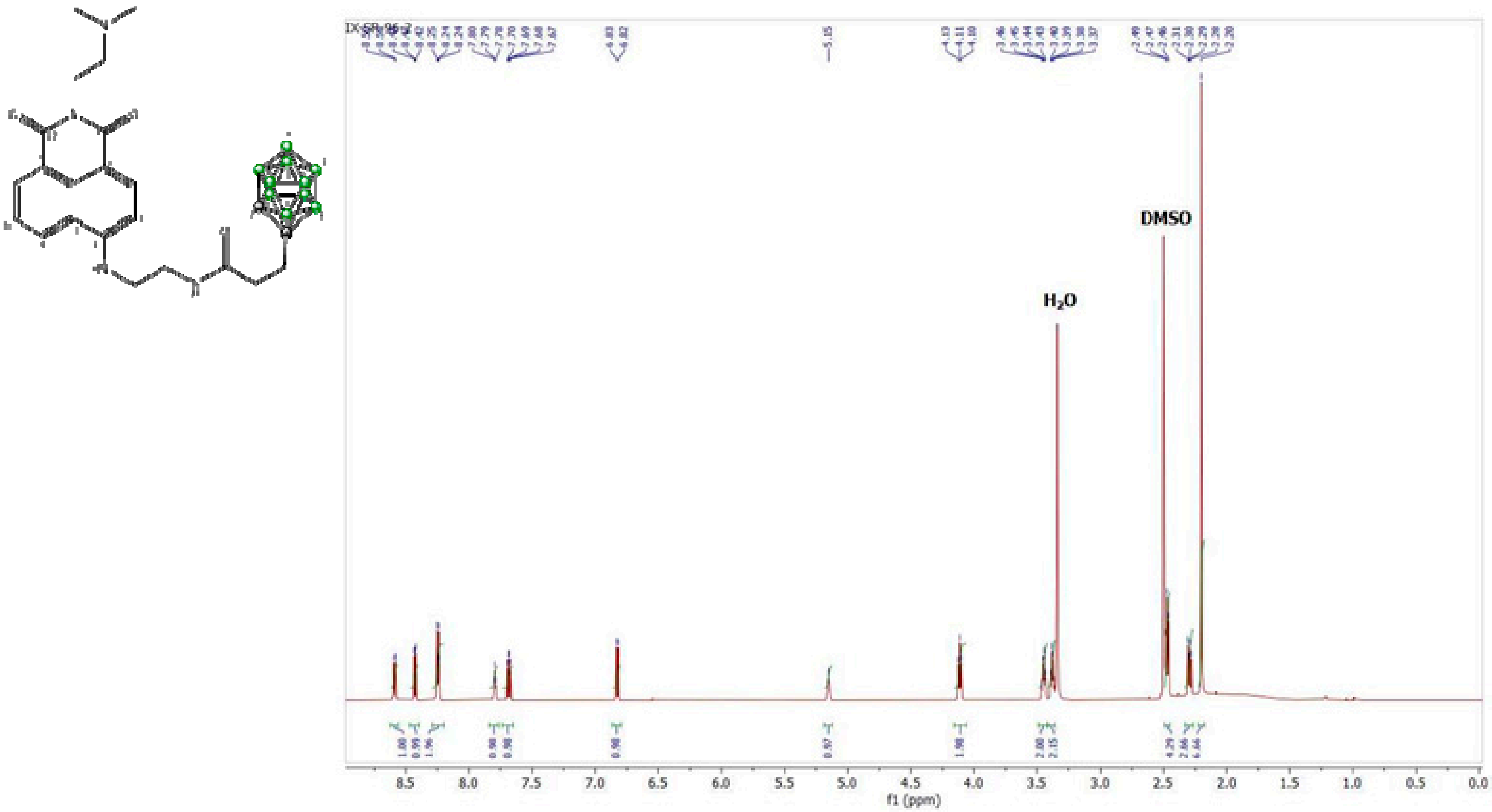

Figure S129. ^{1}H NMR spectrum of **36**.

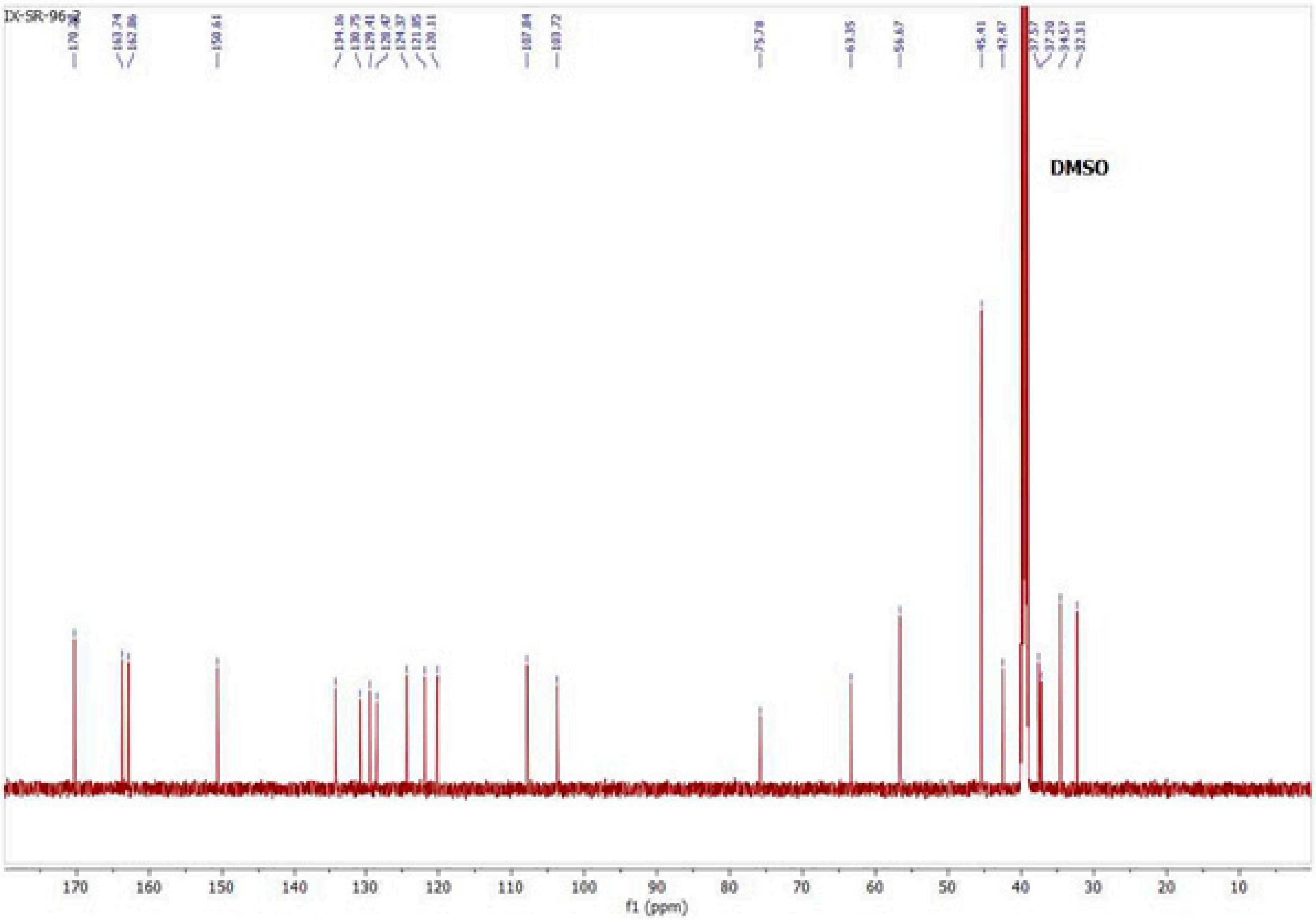

Figure S130. ^{13}C NMR spectrum of **36**.

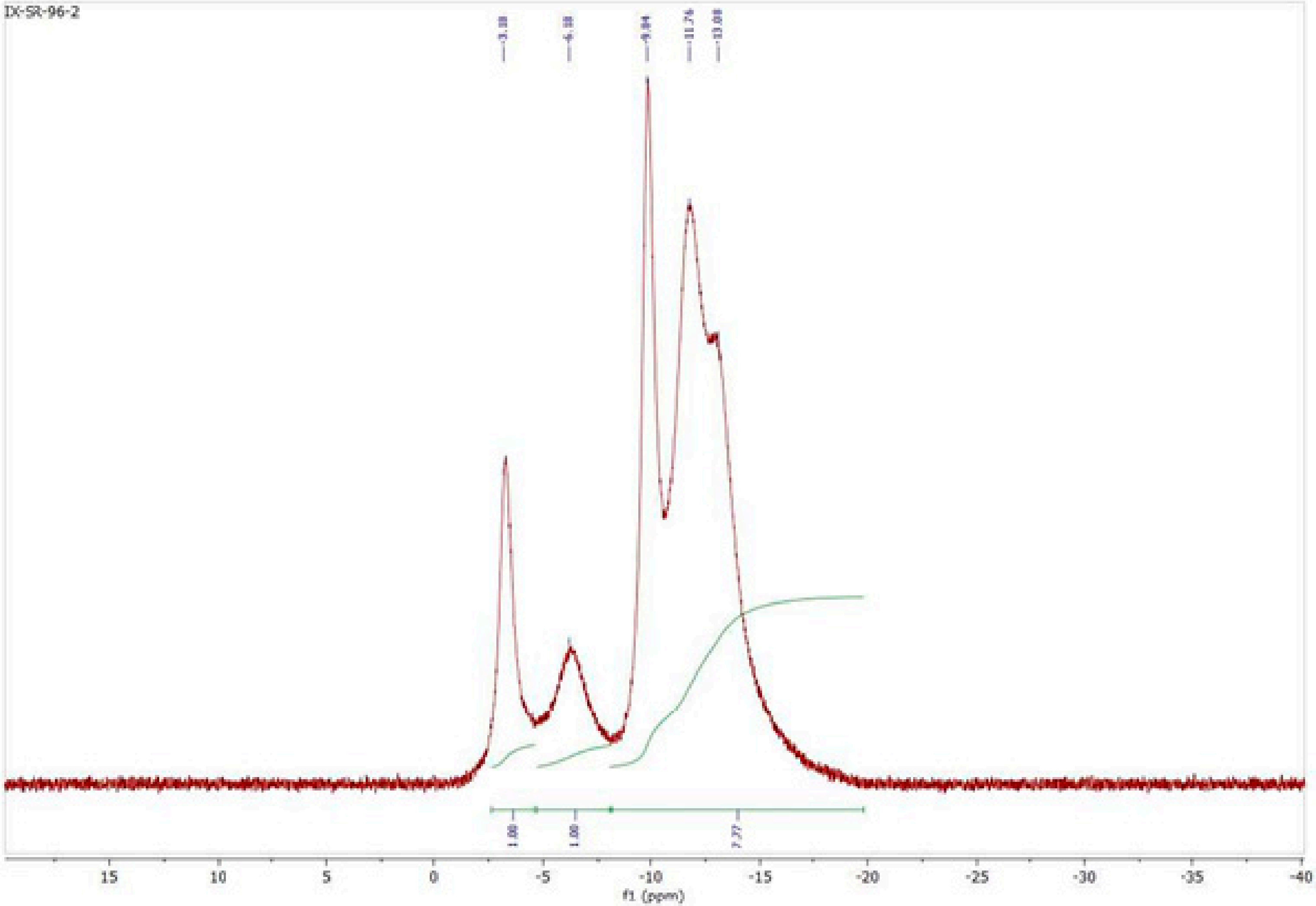

Figure S131. ^{11}B NMR spectrum of **36**.

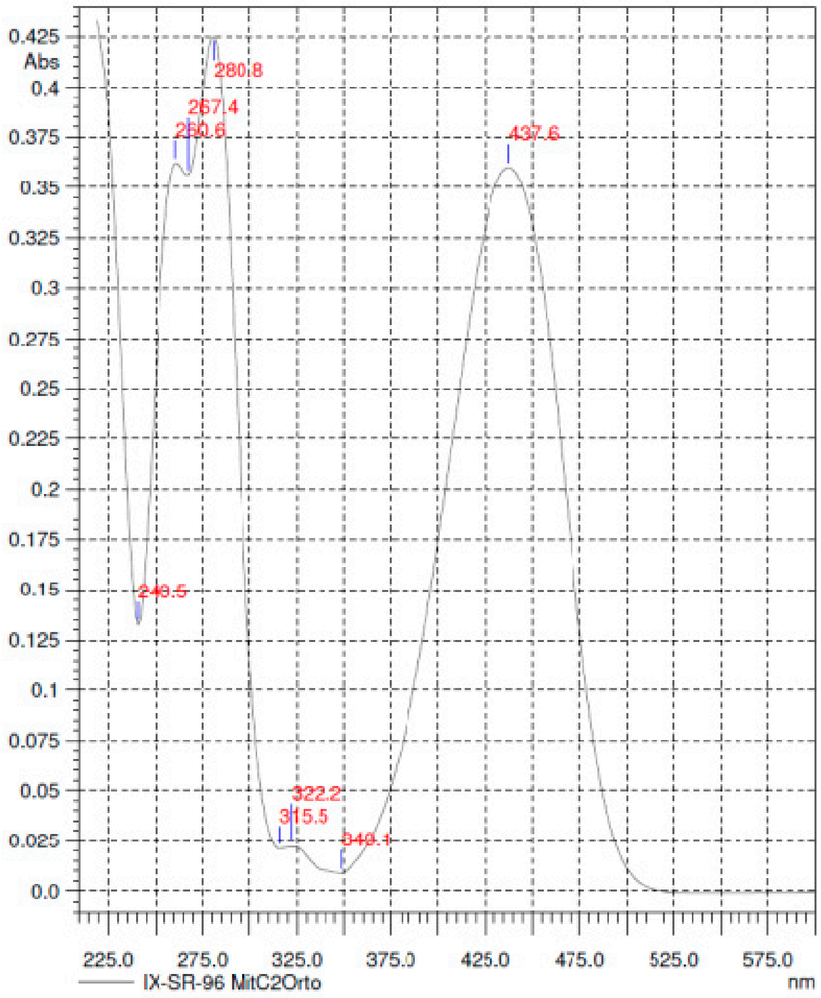

Figure S132. UV spectrum of **36.**

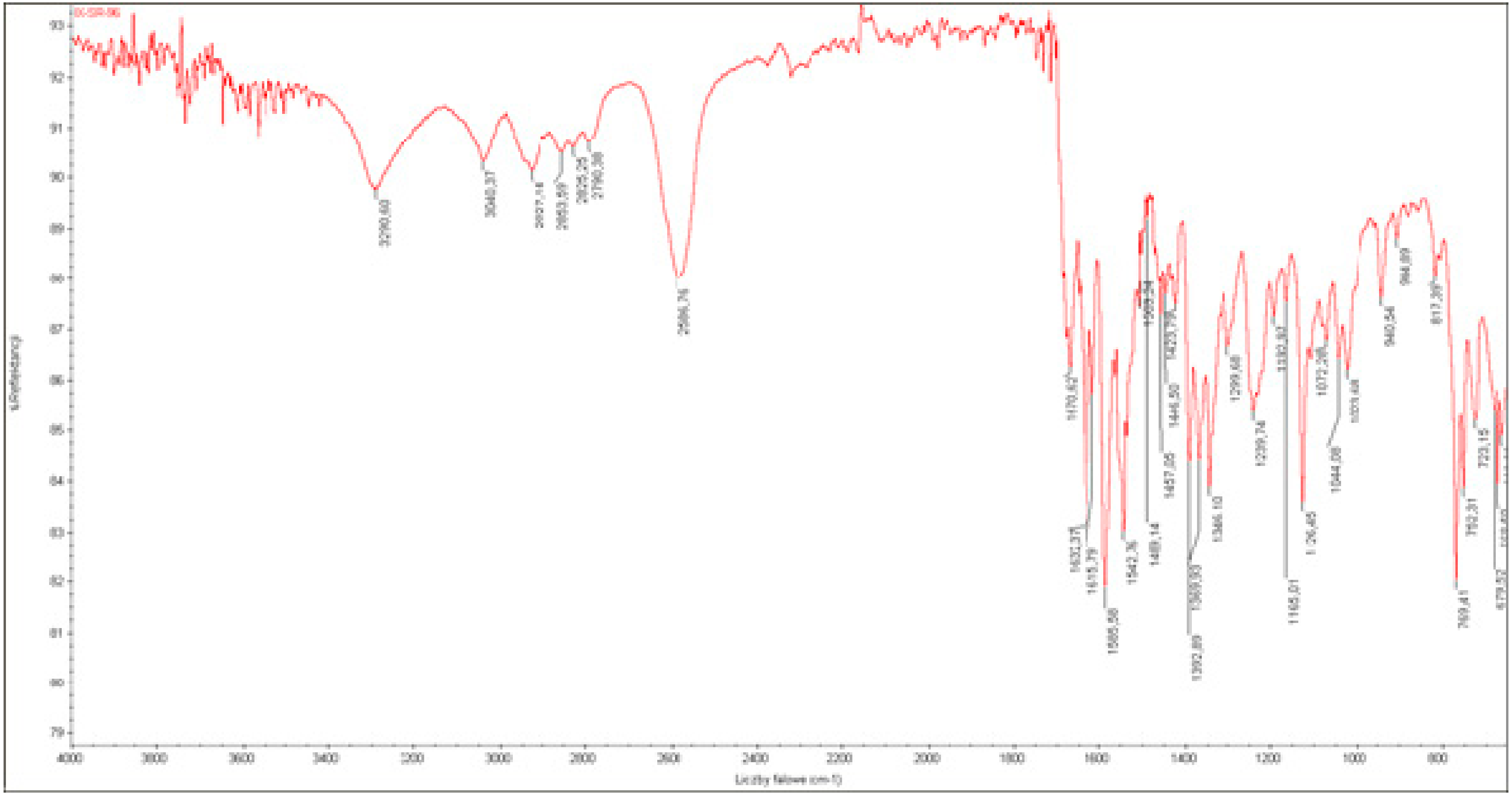

Figure S133. IR spectrum of **36**.

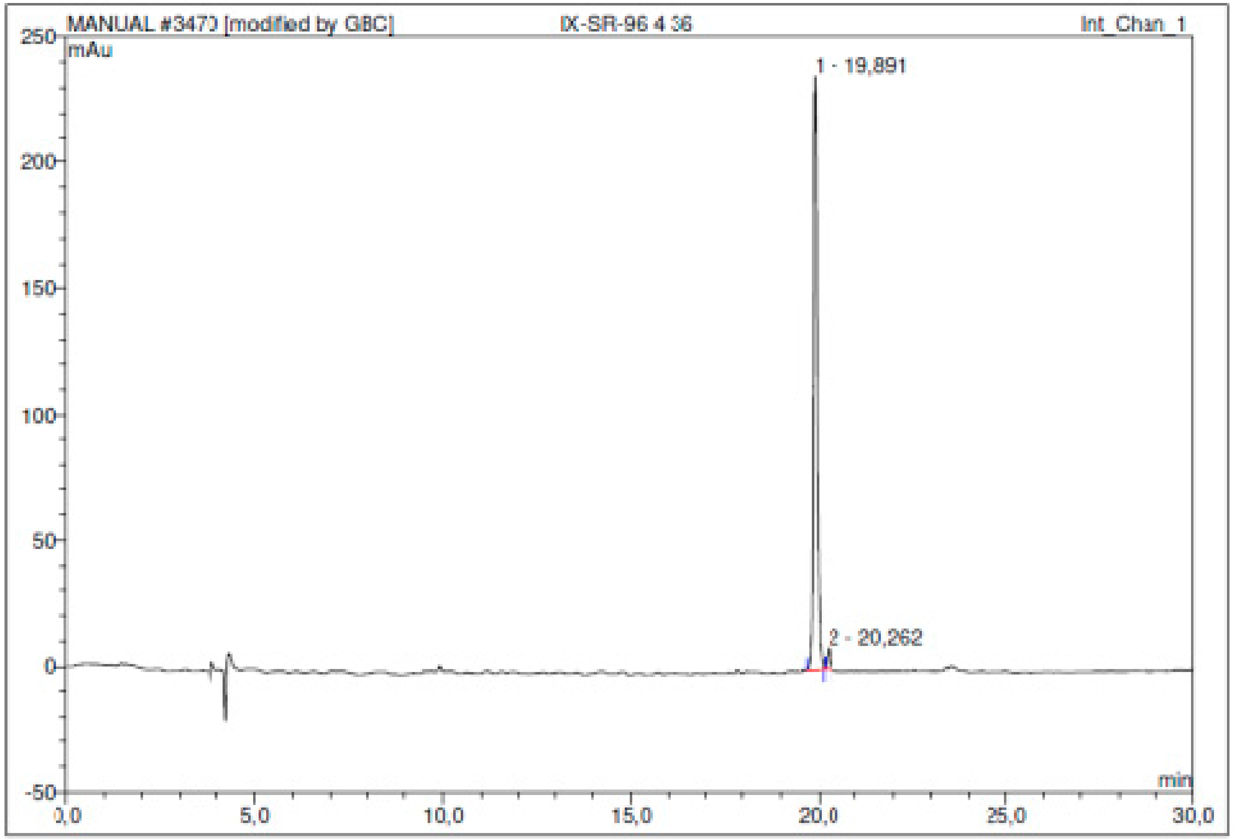

No.	Ret.Time min	Peak Name	Height mAu	Area mAu*min	Rel.Area %	Amount	Type
1	19,89	n.a.	235,846	27,796	97,45	n.a.	BMB
2	20,26	n.a.	8,106	0,728	2,55	n.a.	BMB*
Total:			243,952	28,524	100,00	0,000	

Figure S134. HPLC chromatogram of **36**.

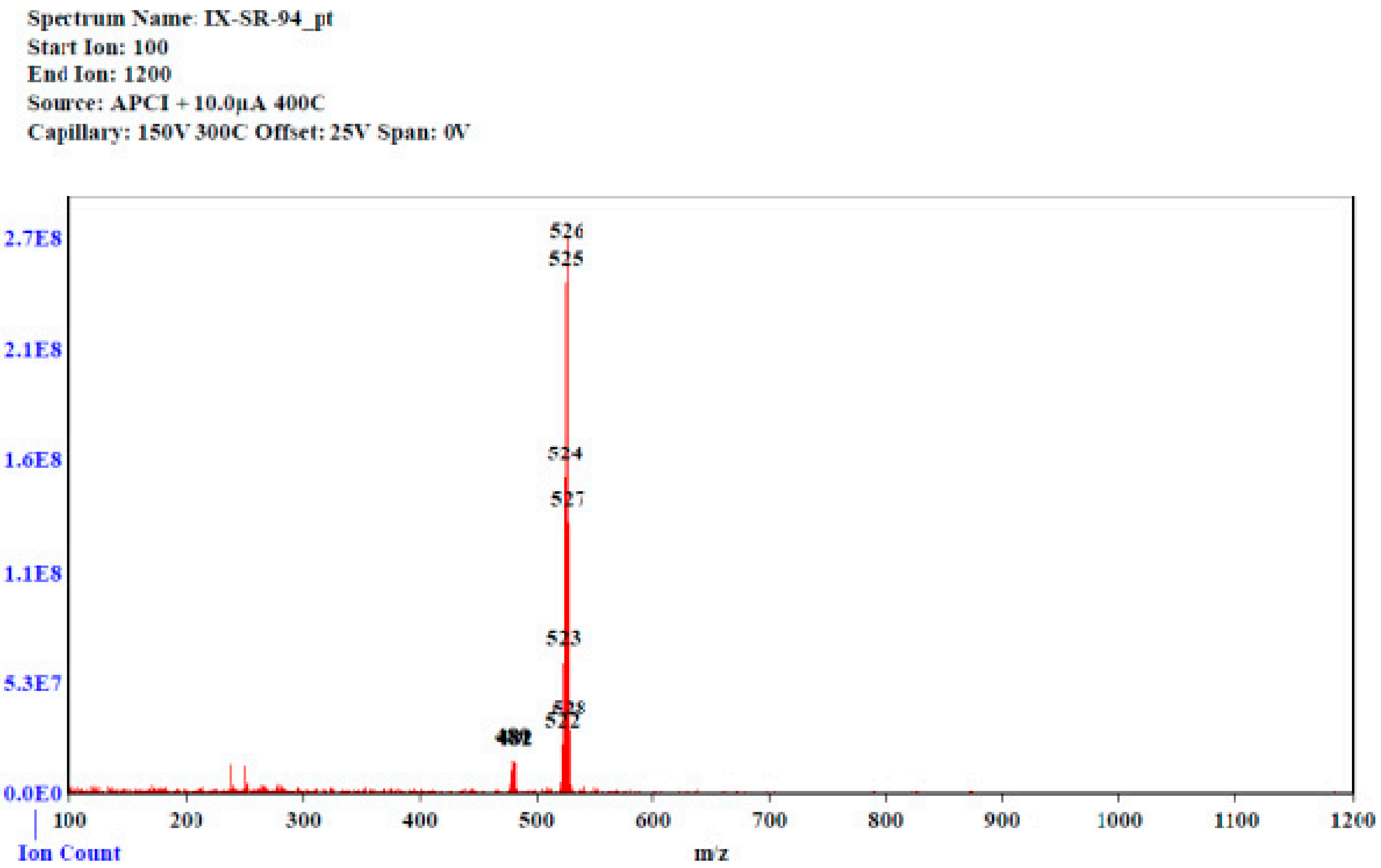

Figure S135. MS spectrum of **36**.

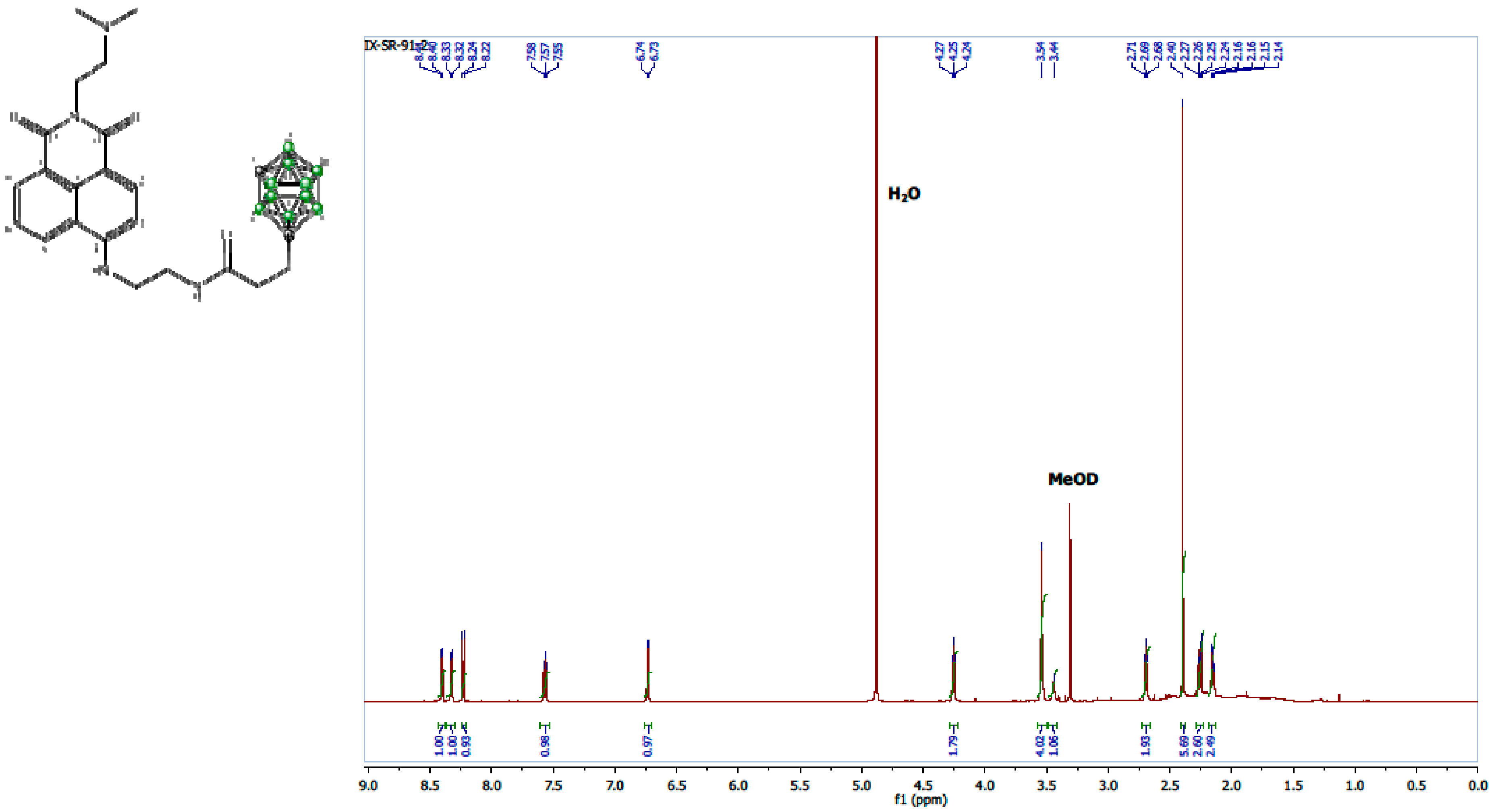

Figure S136. ^{1}H NMR spectrum of **37**.

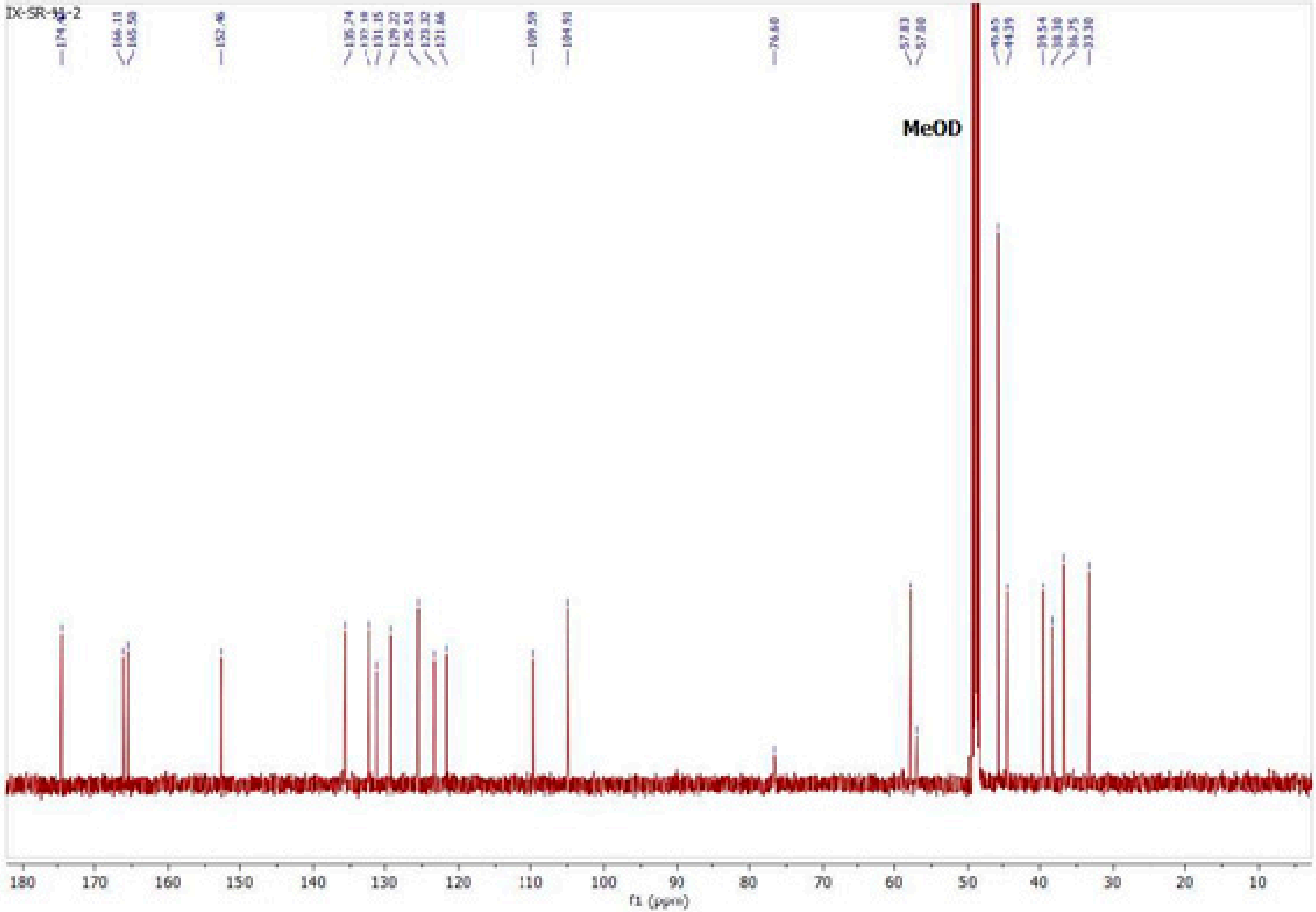

Figure S137. ^{13}C NMR spectrum of **37**.

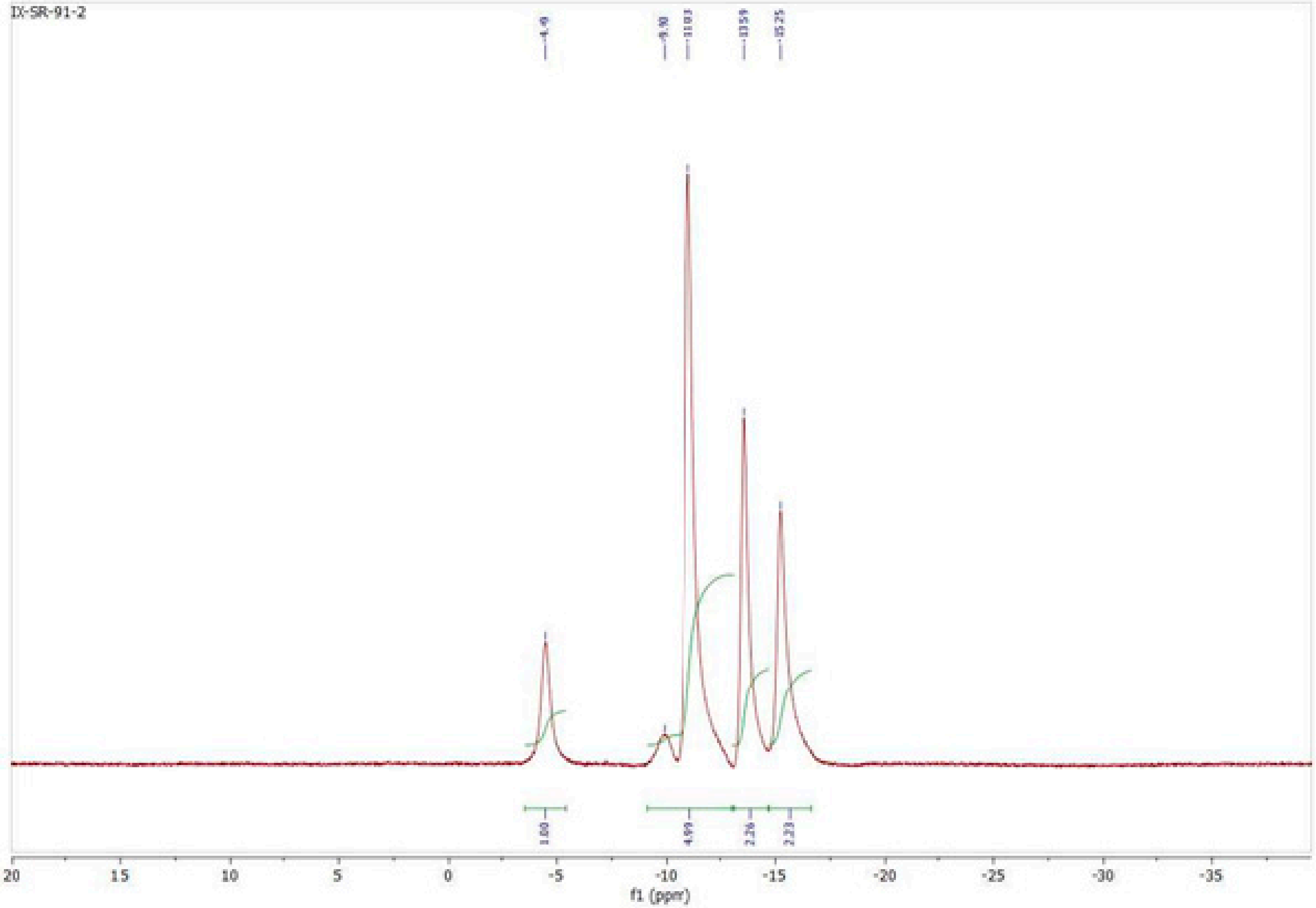

Figure S138. ^{11}B NMR spectrum of **37**.

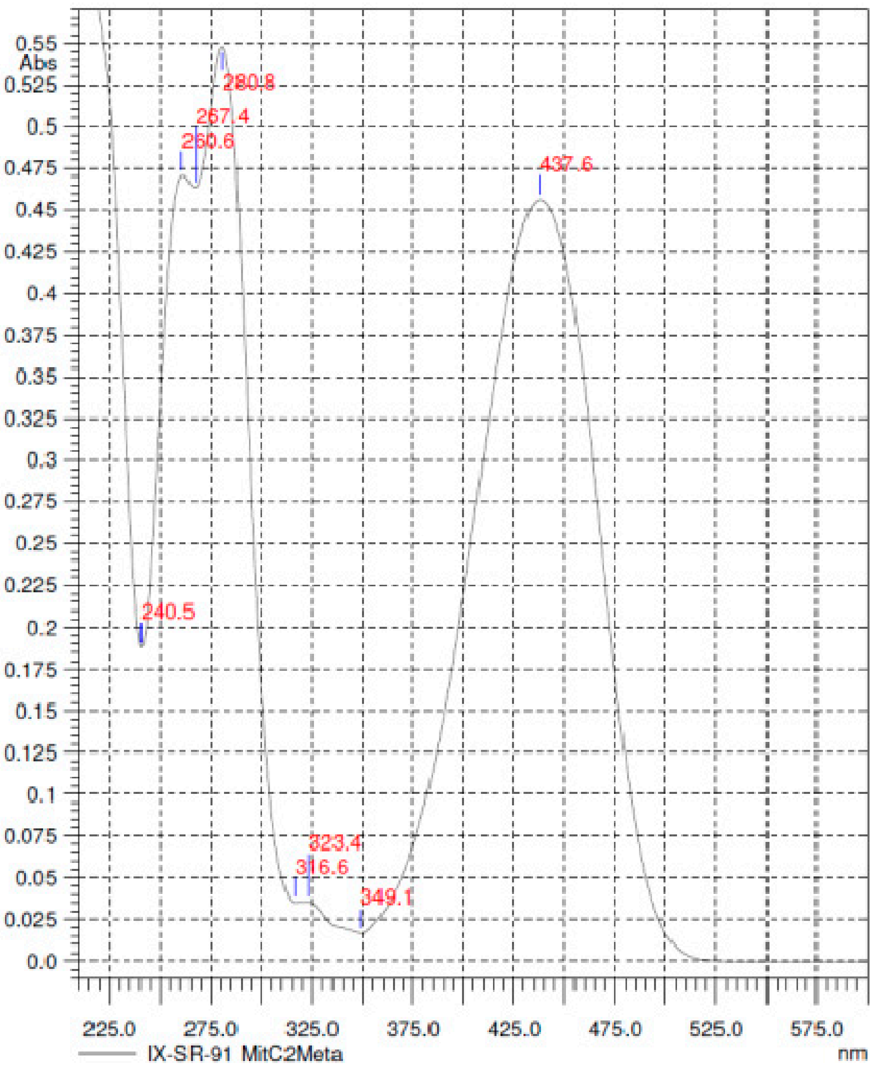

Figure S139. UV spectrum of **37.**

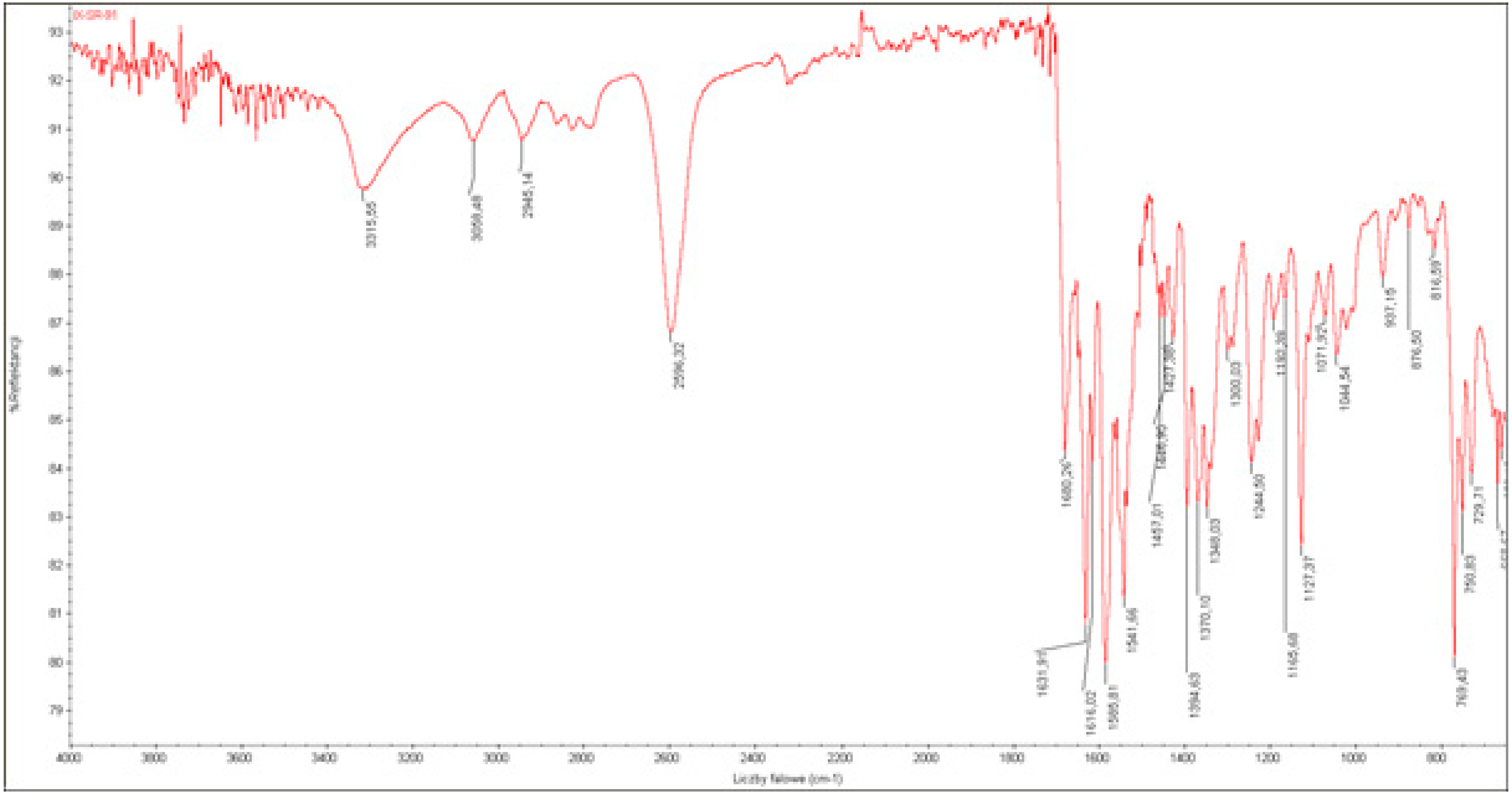

Figure S140. IR spectrum of **37**.

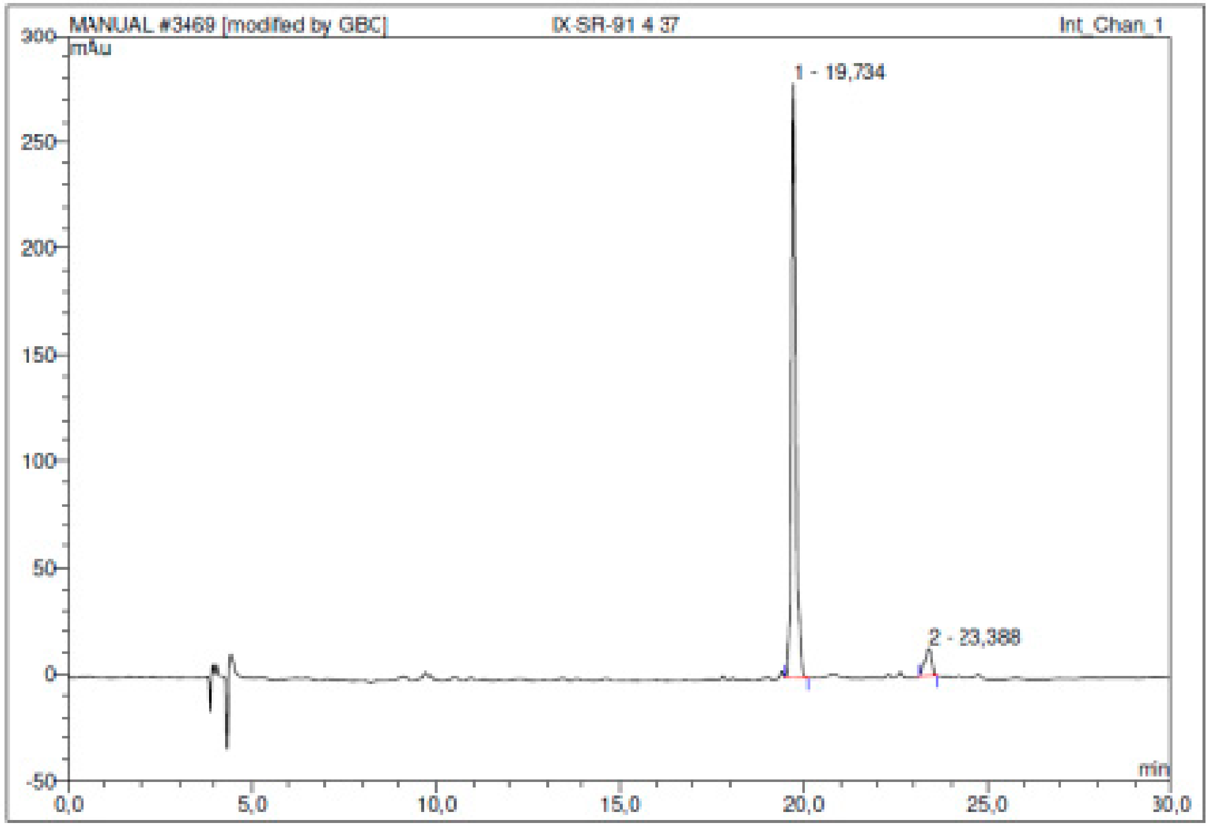

No.	Ret.Time min	Peak Name	Height mAu	Area mAu*min	Rel.Area %	Amount	Type
1	19,73	n.a.	278,969	37,437	93,18	n.a.	BMB
2	23,39	n.a.	12,580	2,740	6,82	n.a.	BMB*
Total:			291,550	40,177	100,00	0,000	

Figure S141. HPLC chromatogram of **37**.

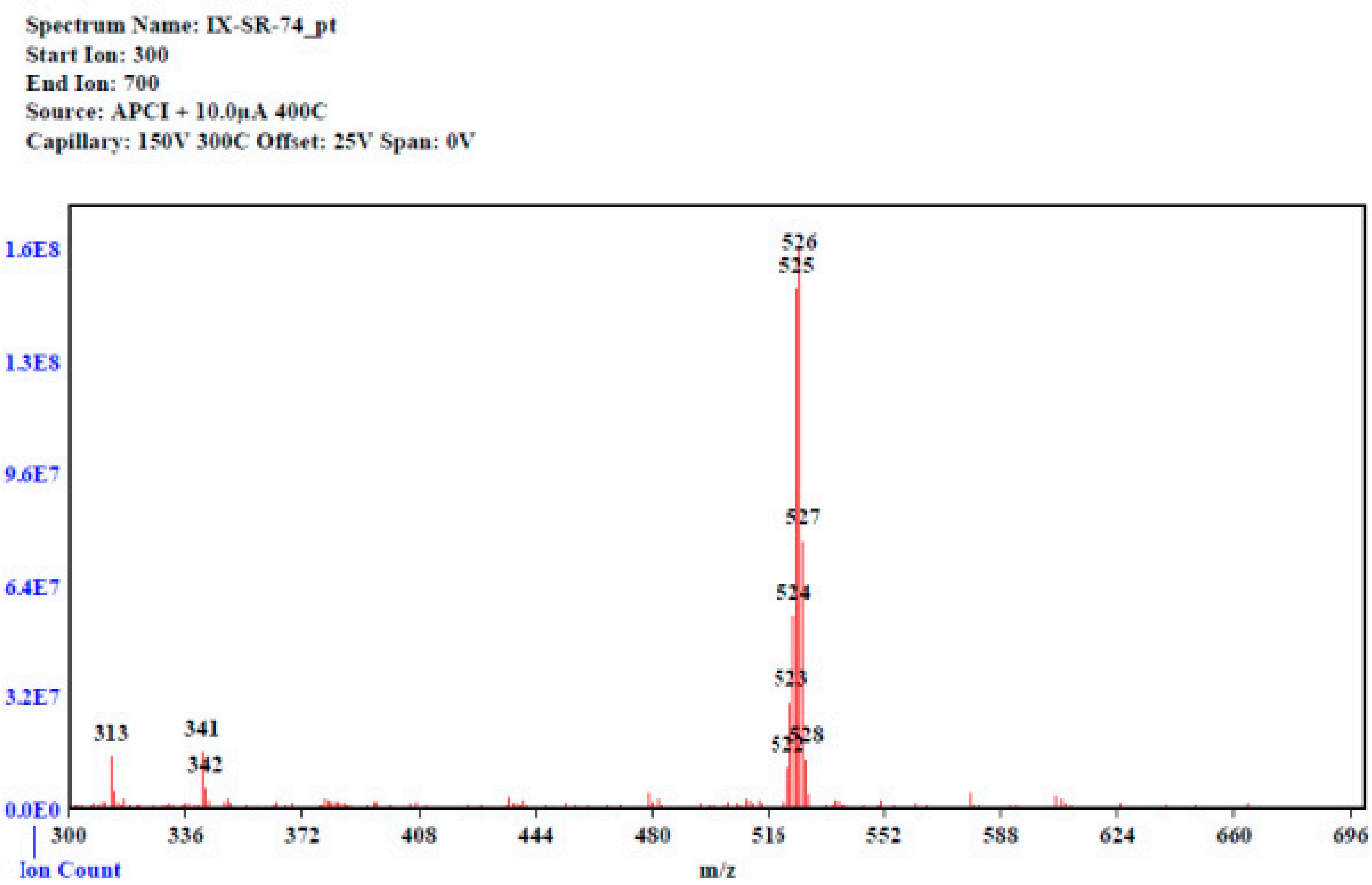

Figure S142. MS spectrum of **37**.

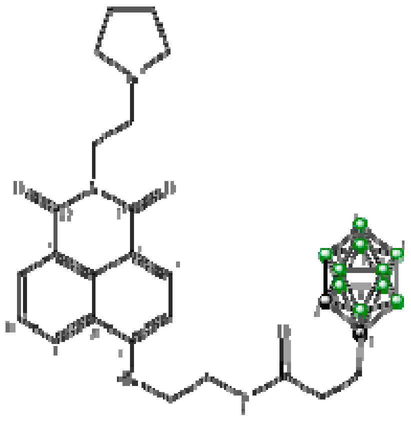

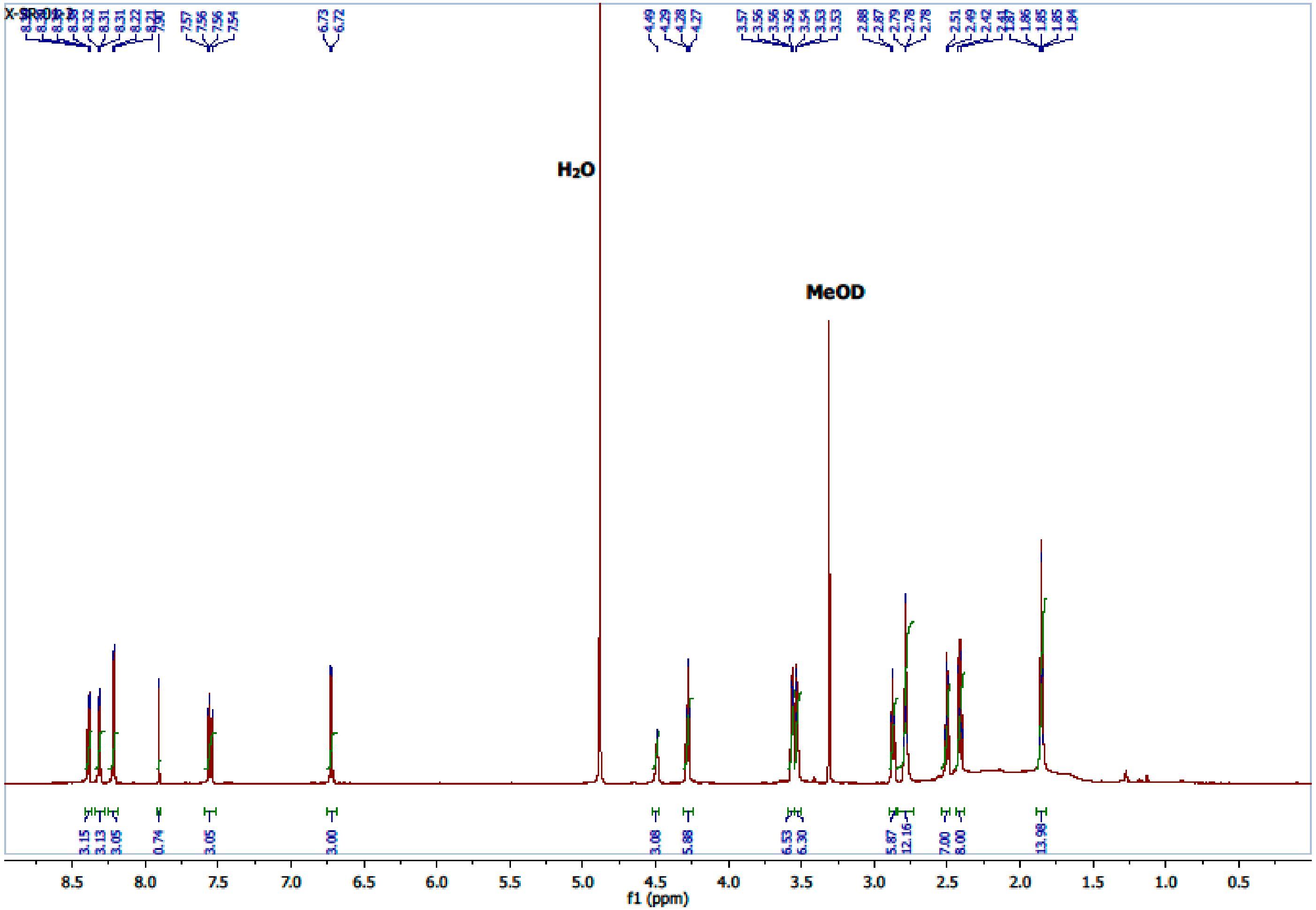

Figure S143. ^{1}H NMR spectrum of **38**.

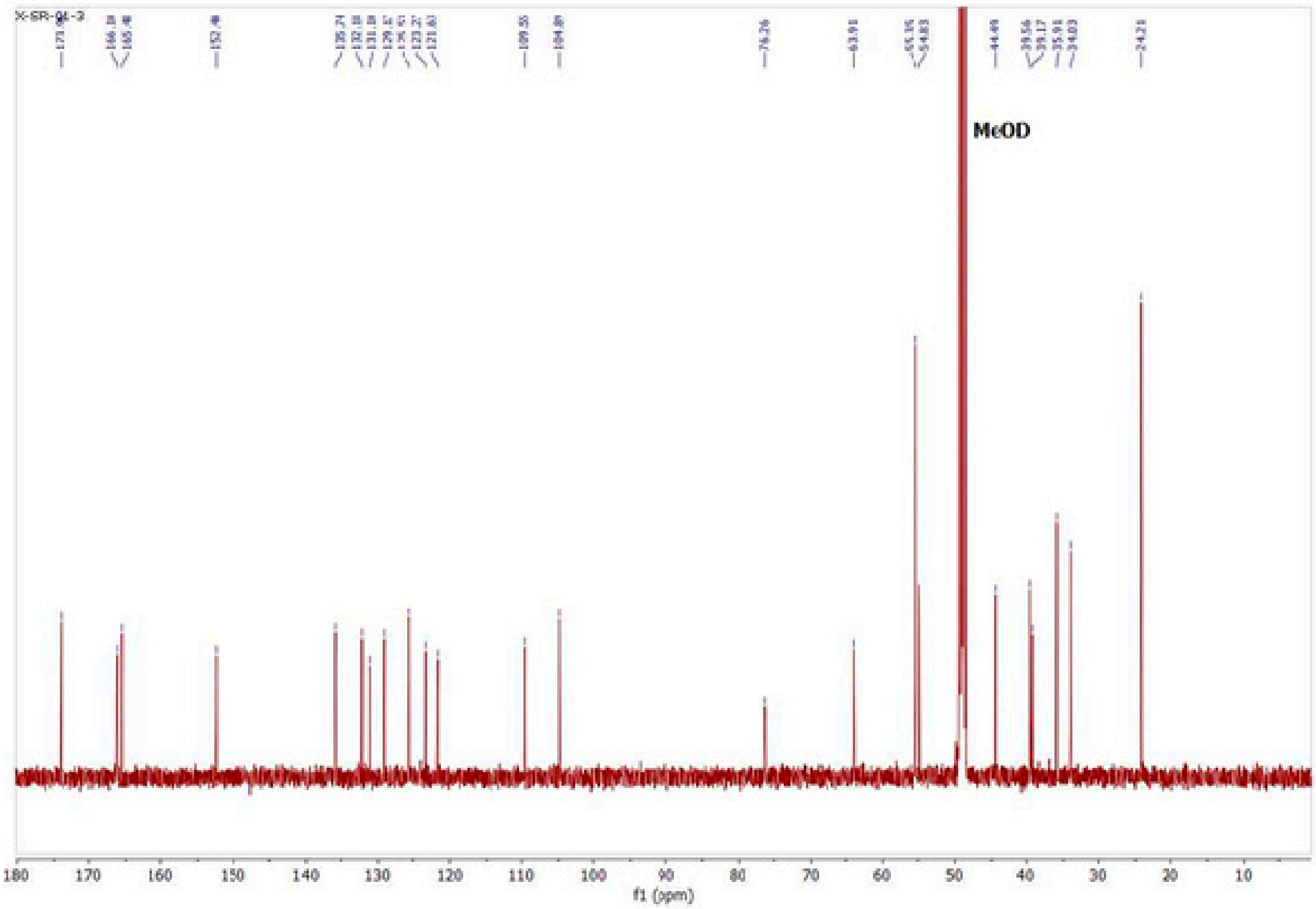

Figure S144. ^{13}C NMR spectrum of **38**.

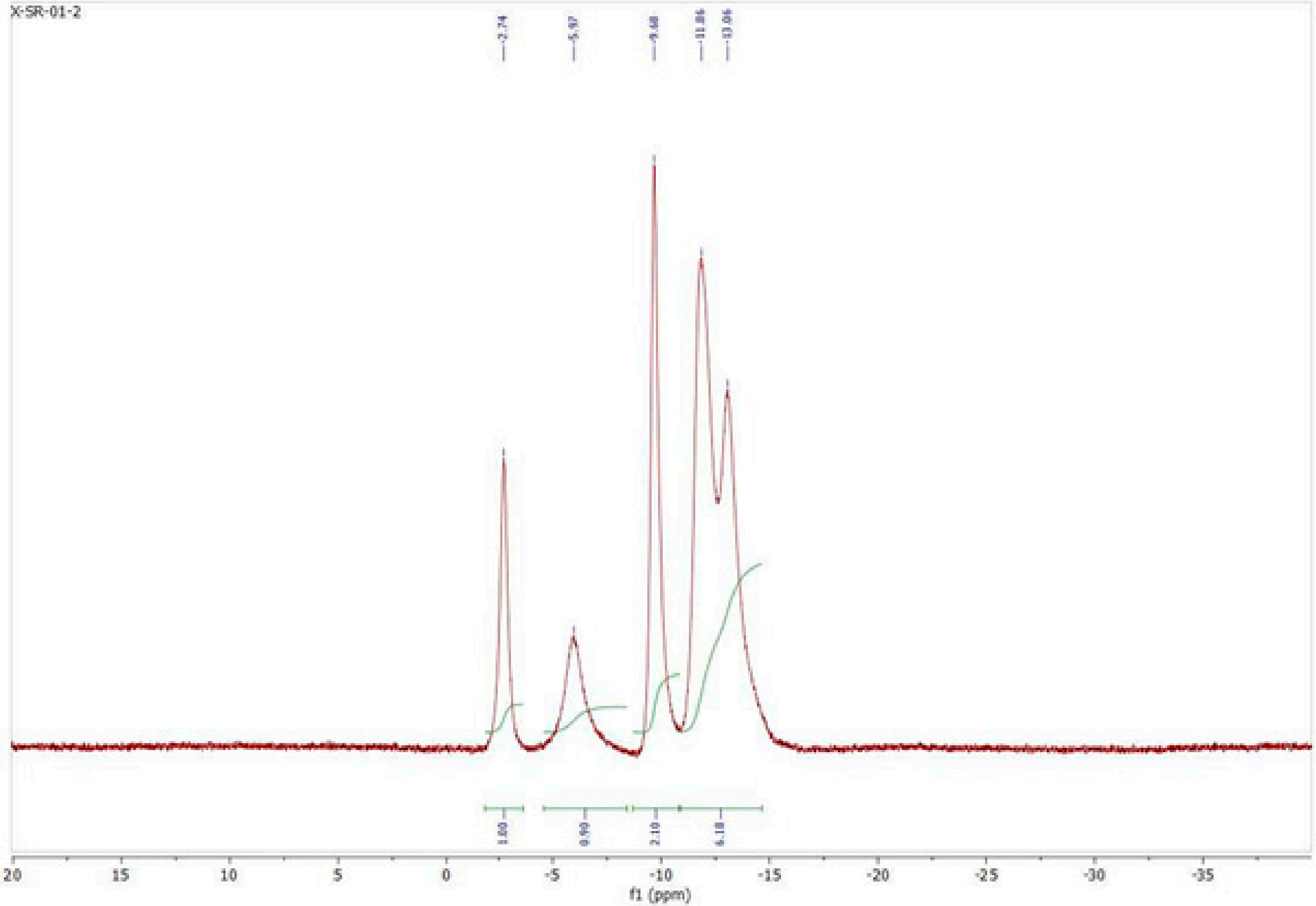

Figure S145. ^{11}B NMR spectrum of **38**.

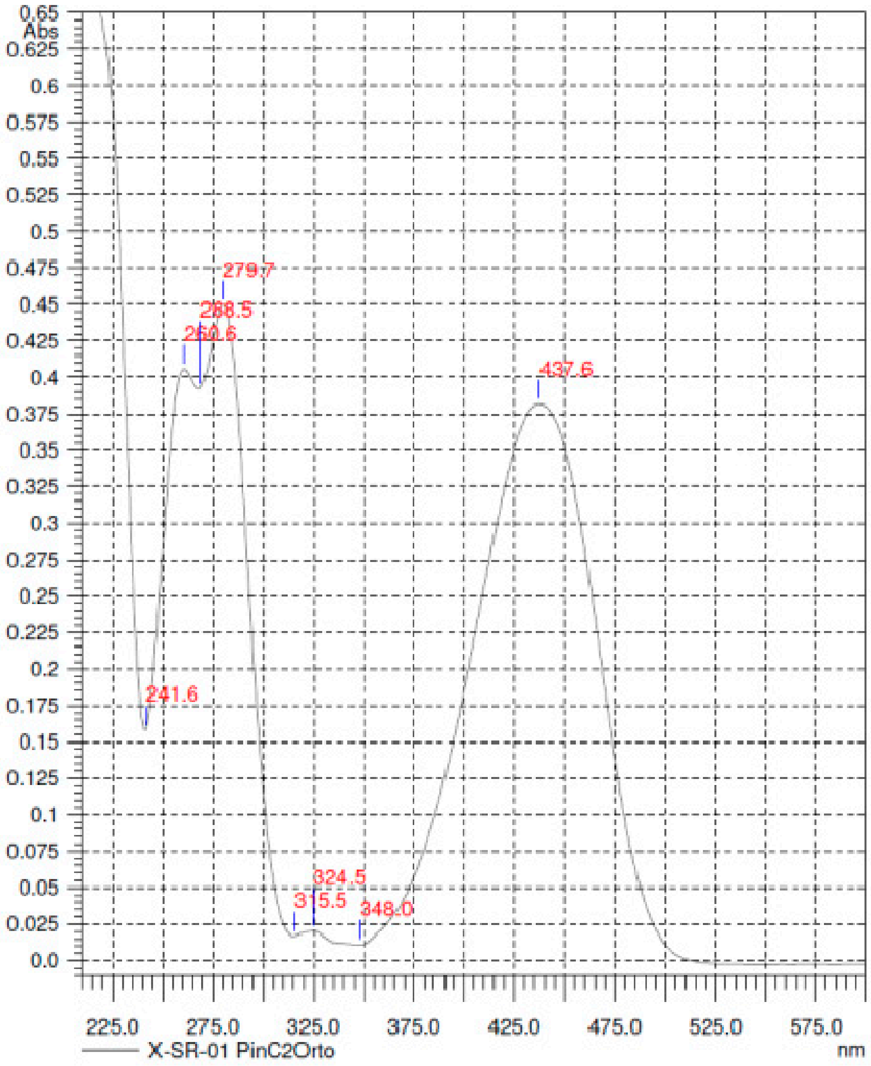

Figure S146. UV spectrum of **38**.

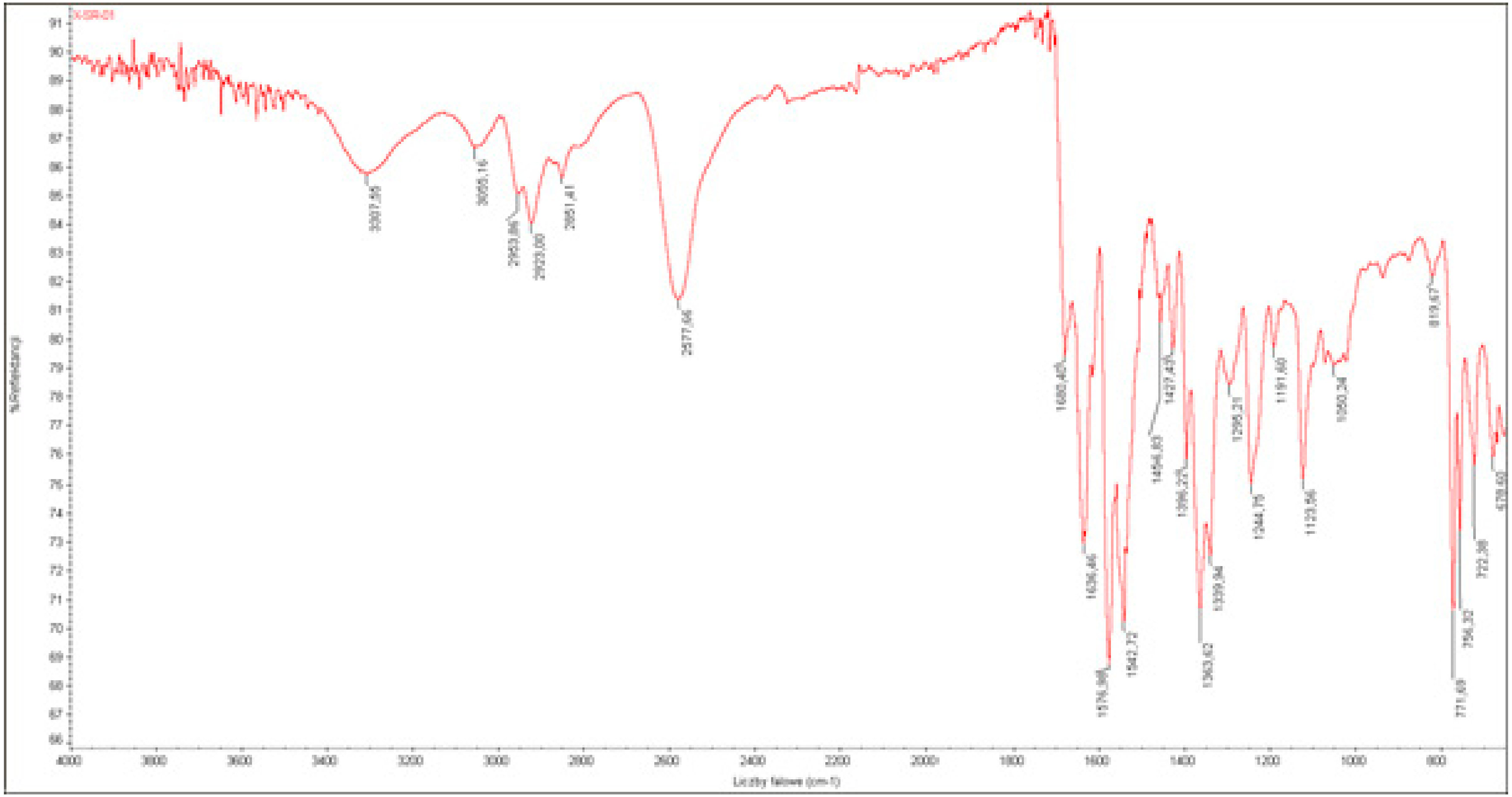

Figure S147. IR spectrum of **38**.

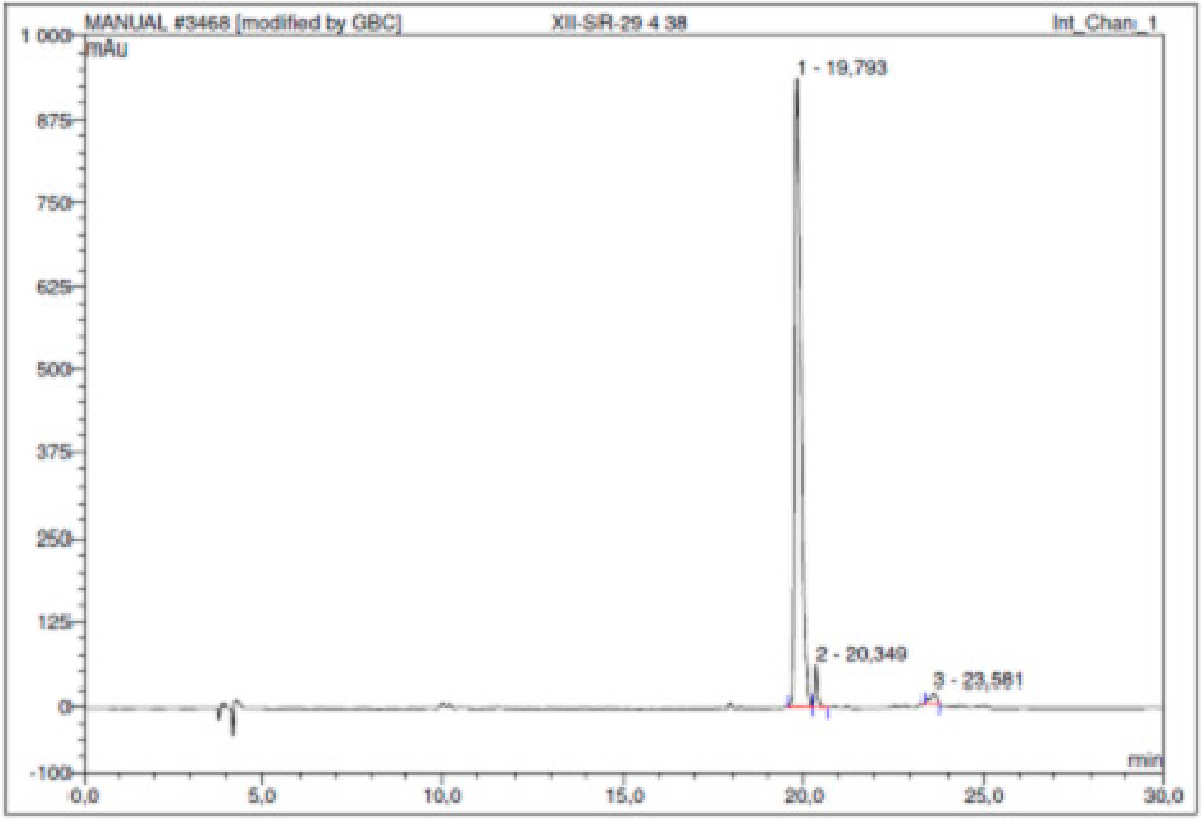

No.	Ret.Time min	Peak Name	Height mAu	Area mAu*min	Rel.Area %	Amount	Type
1	19,79	n.a.	938,417	187,695	95,10	n.a.	BMI
2	20,35	n.a.	59,887	6,157	3,12	n.a.	MB
3	23,58	n.a.	16,327	3,511	1,78	n.a.	BMB*
Total:			1014,630	197,364	100,00	0,000	

Figure S148. HPLC chromatogram of **38**.

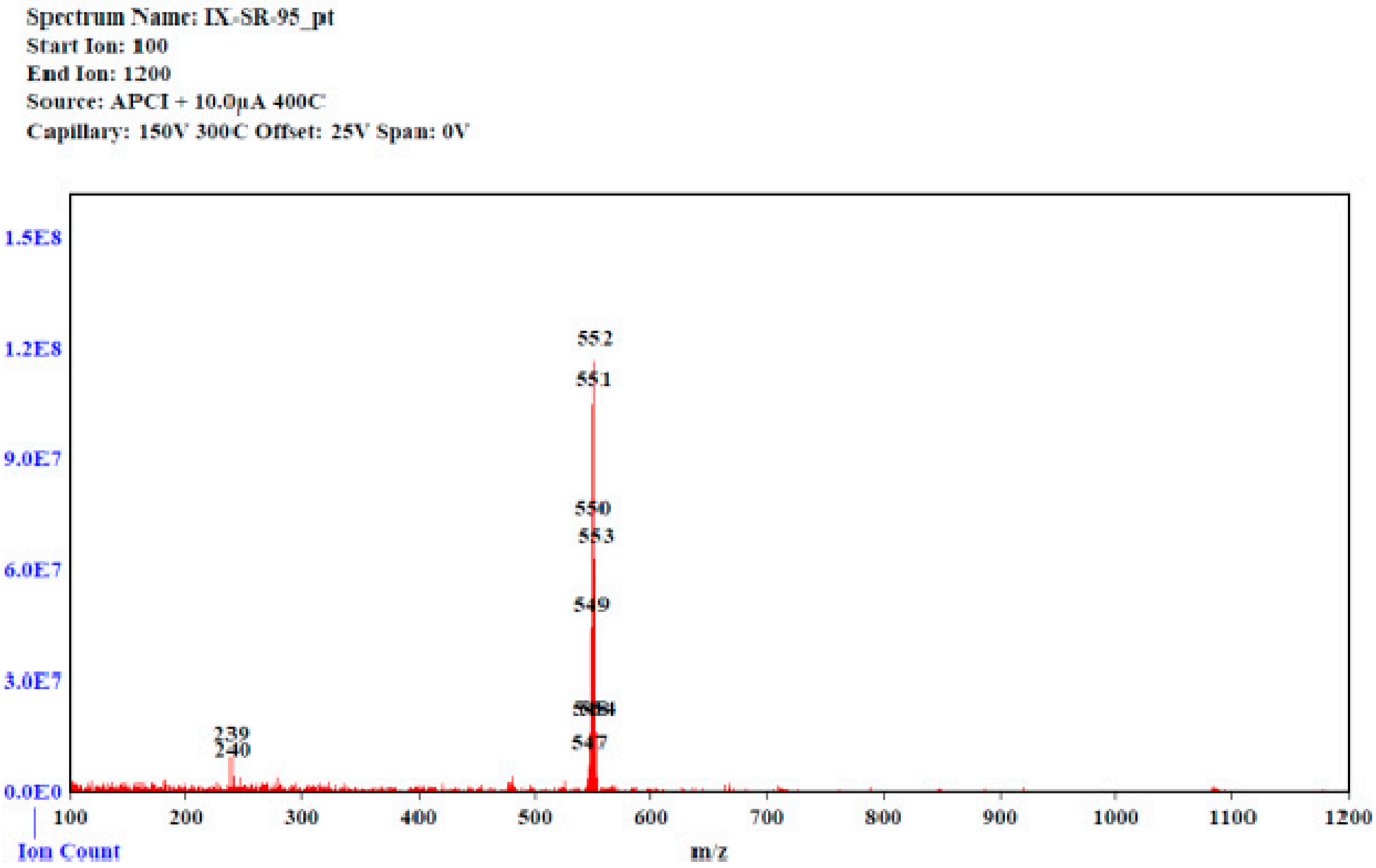

Figure S149. MS spectrum of **38**.

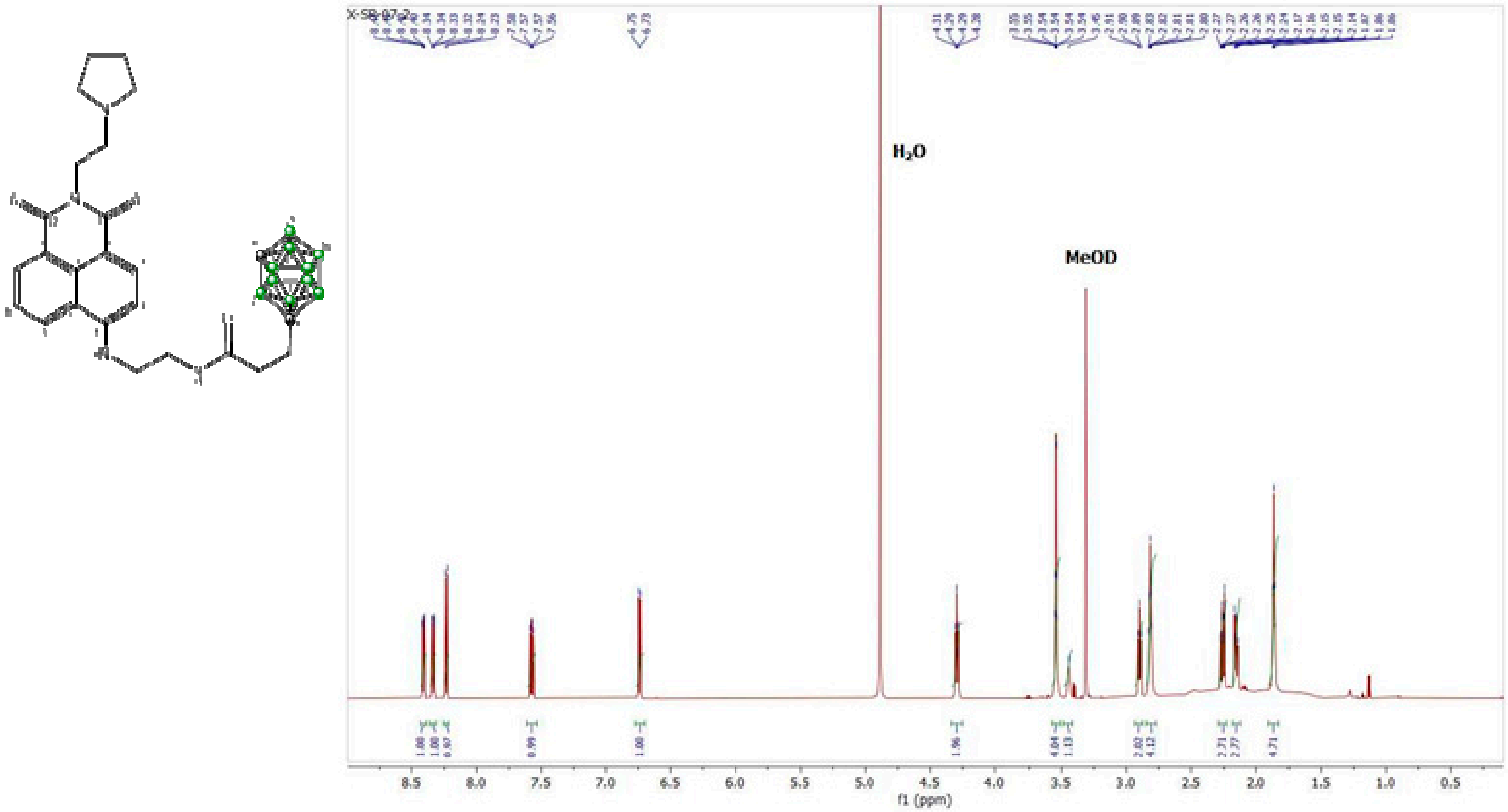

Figure S150. ^{1}H NMR spectrum of **39**.

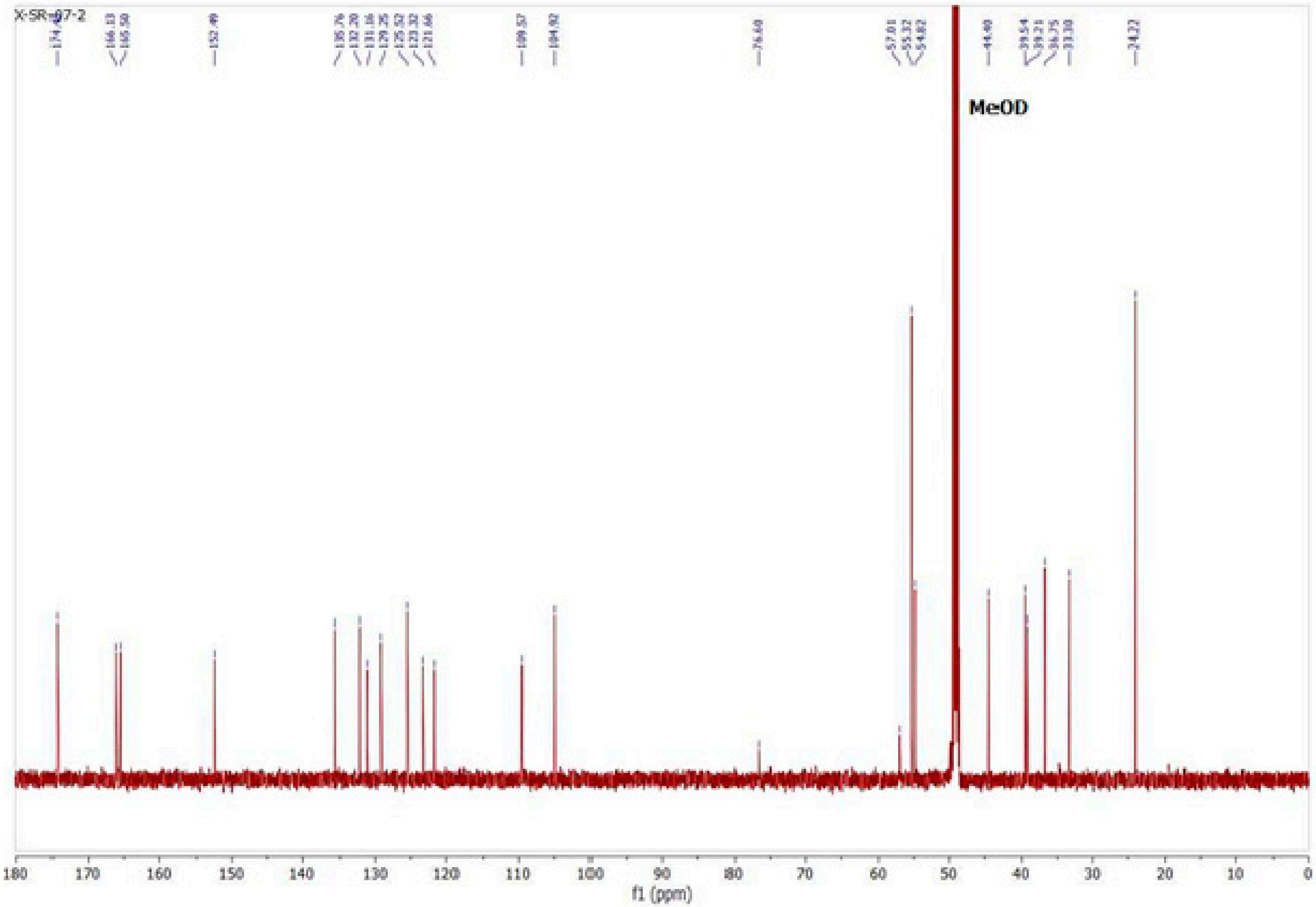

Figure S151. ^{13}C NMR spectrum of **39**.

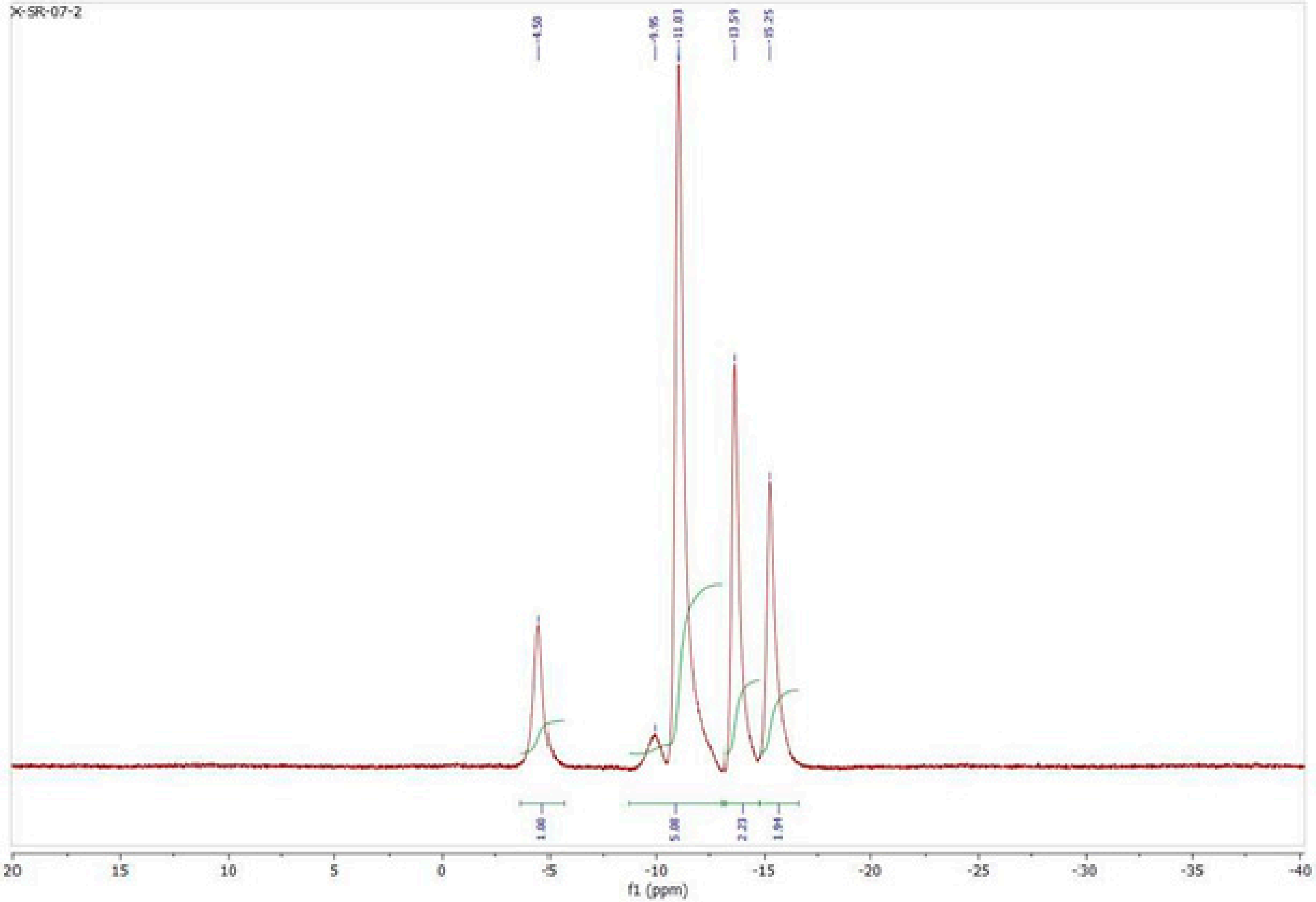

Figure S152. ^{11}B NMR spectrum of **39**.

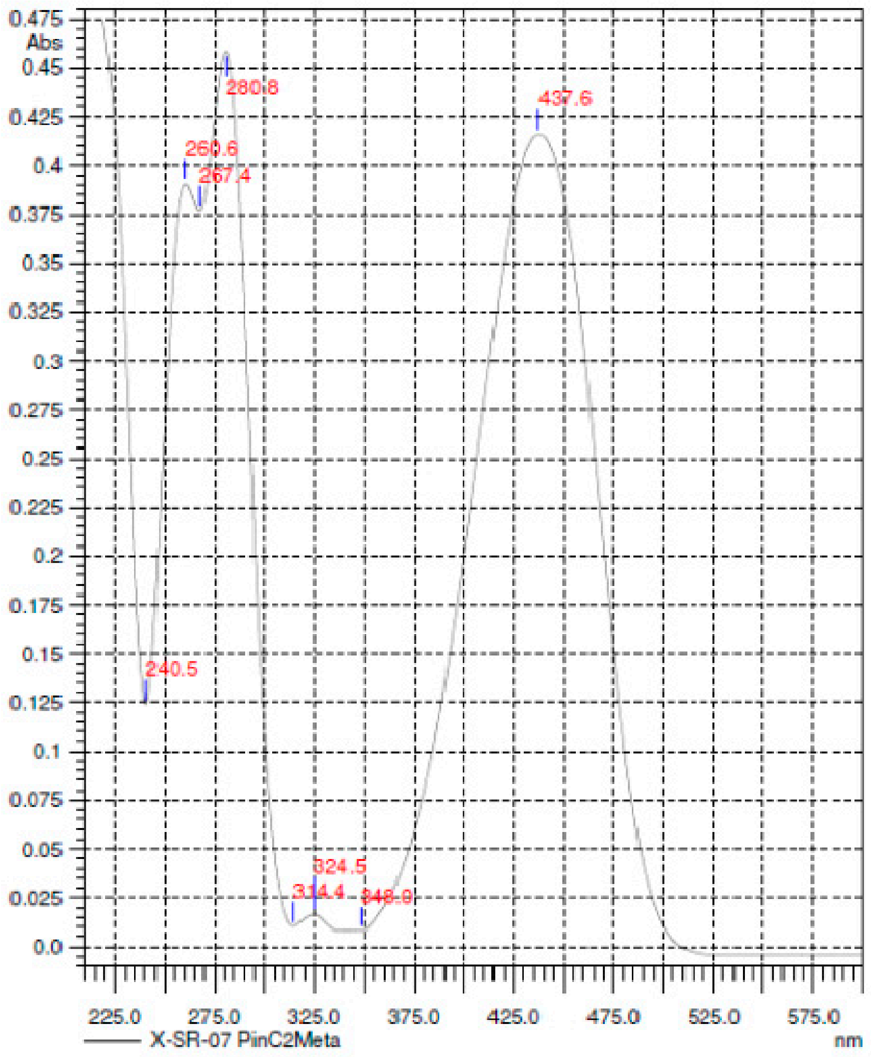

Figure S153. UV spectrum of **39**.

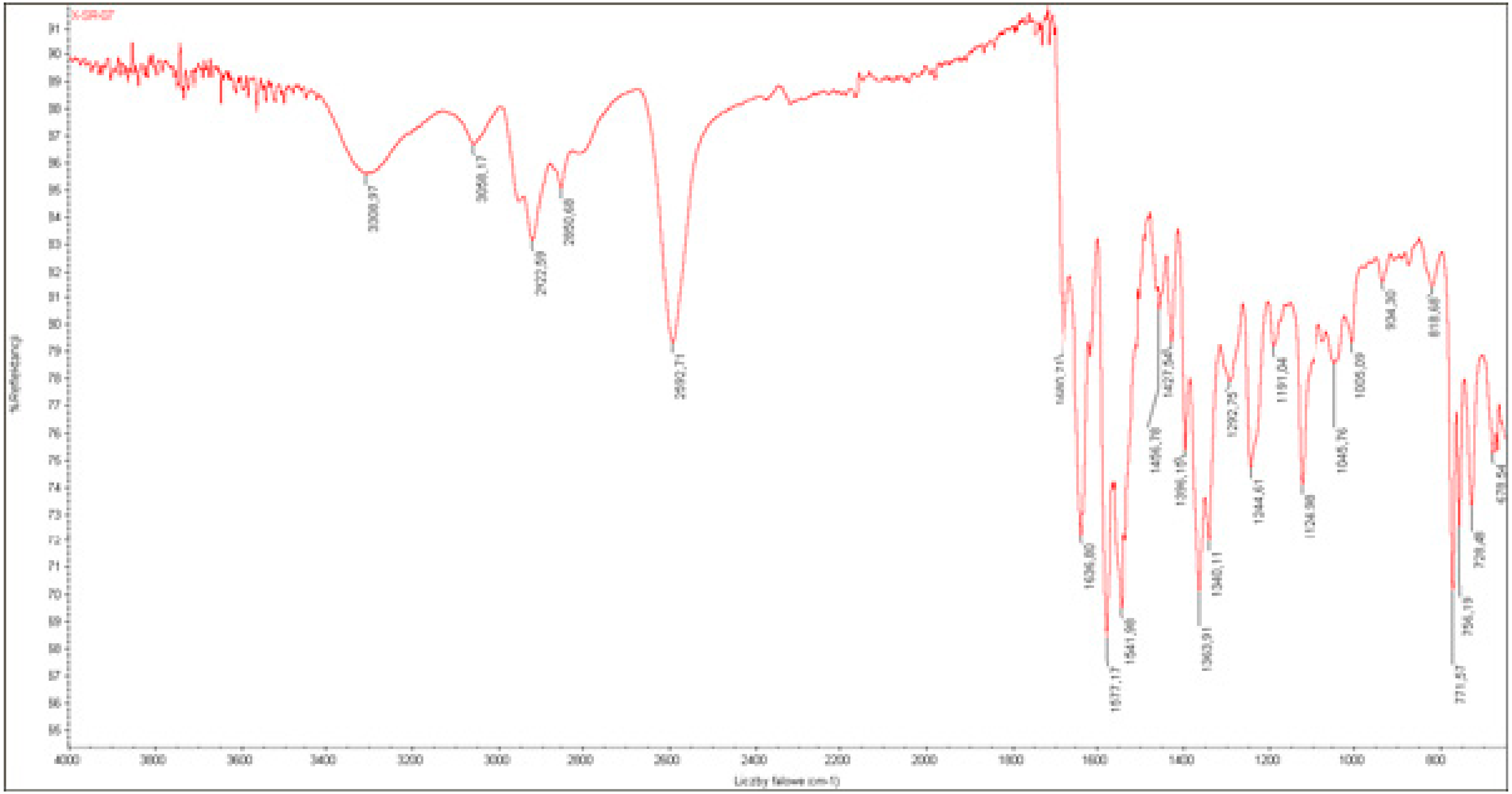

Figure S154. IR spectrum of **39**.

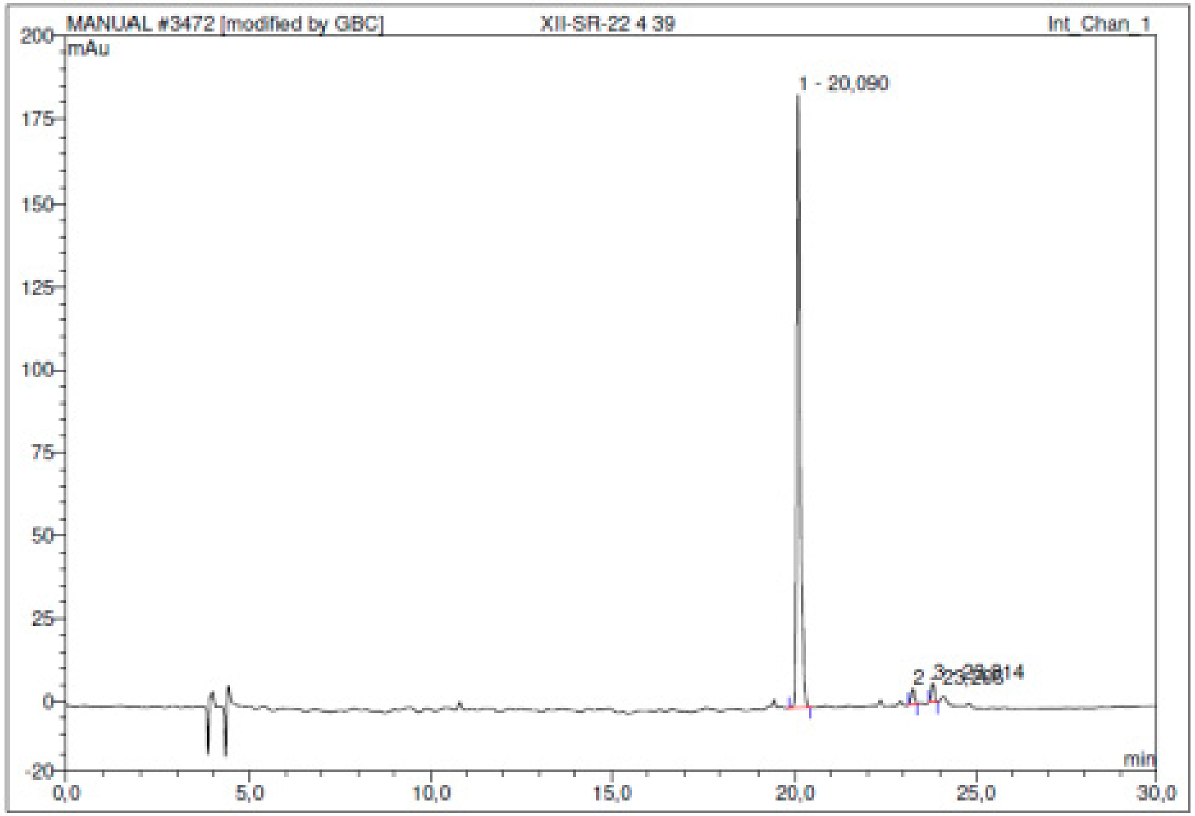

No.	Ret.Time min	Peak Name	Height mAu	Area mAu*min	Rel.Area %	Amount	Type
1	20,09	n.a.	184,065	21,996	95,28	n.a.	BMB
2	23,27	n.a.	4,624	0,509	2,21	n.a.	BMB*
3	23,81	n.a.	5,613	0,580	2,51	n.a.	BMB*
Total:			194,301	23,085	100,00	0,000	

Figure S155. HPLC chromatogram of **39**.

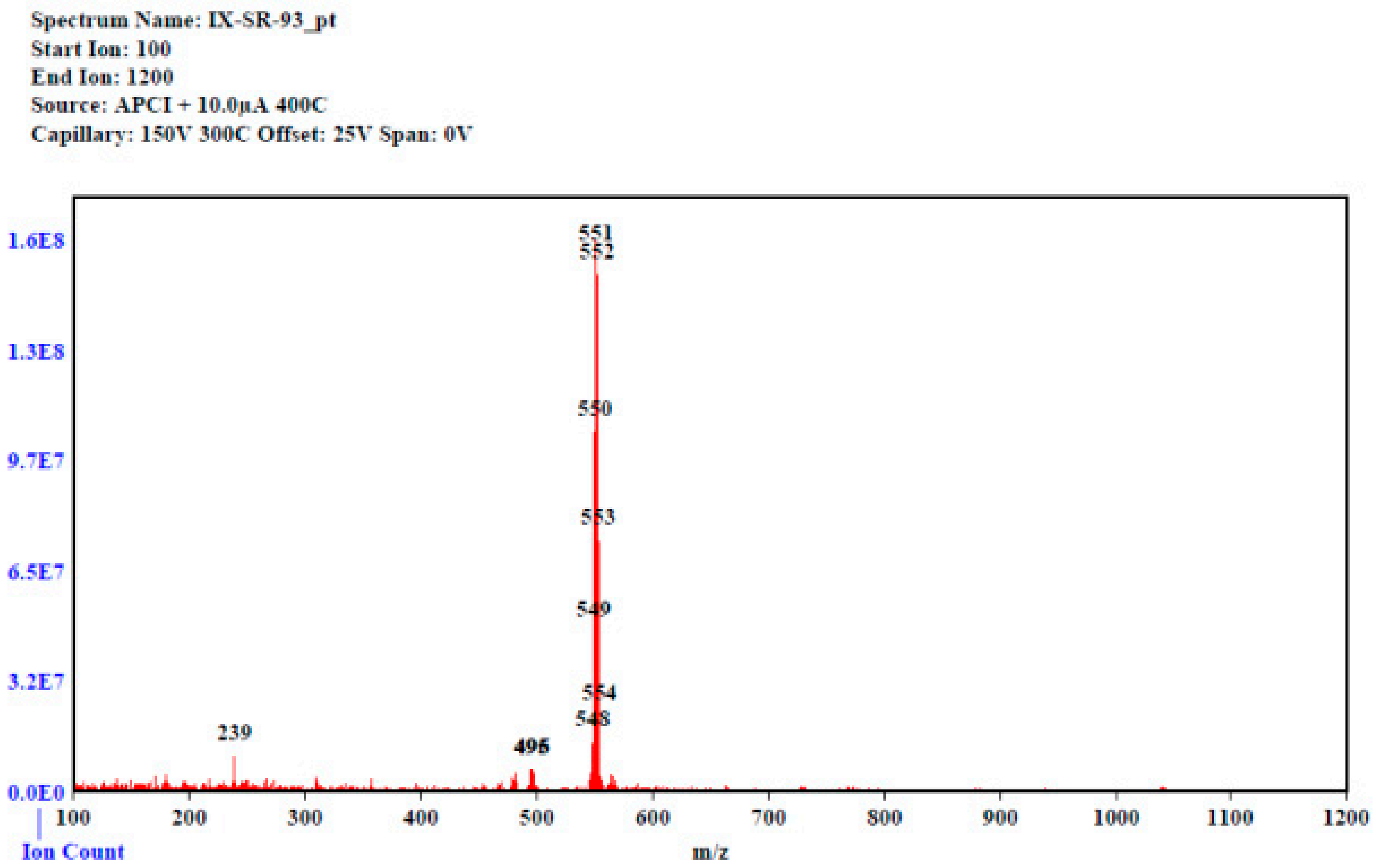

Figure S156. MS spectrum of **39**.

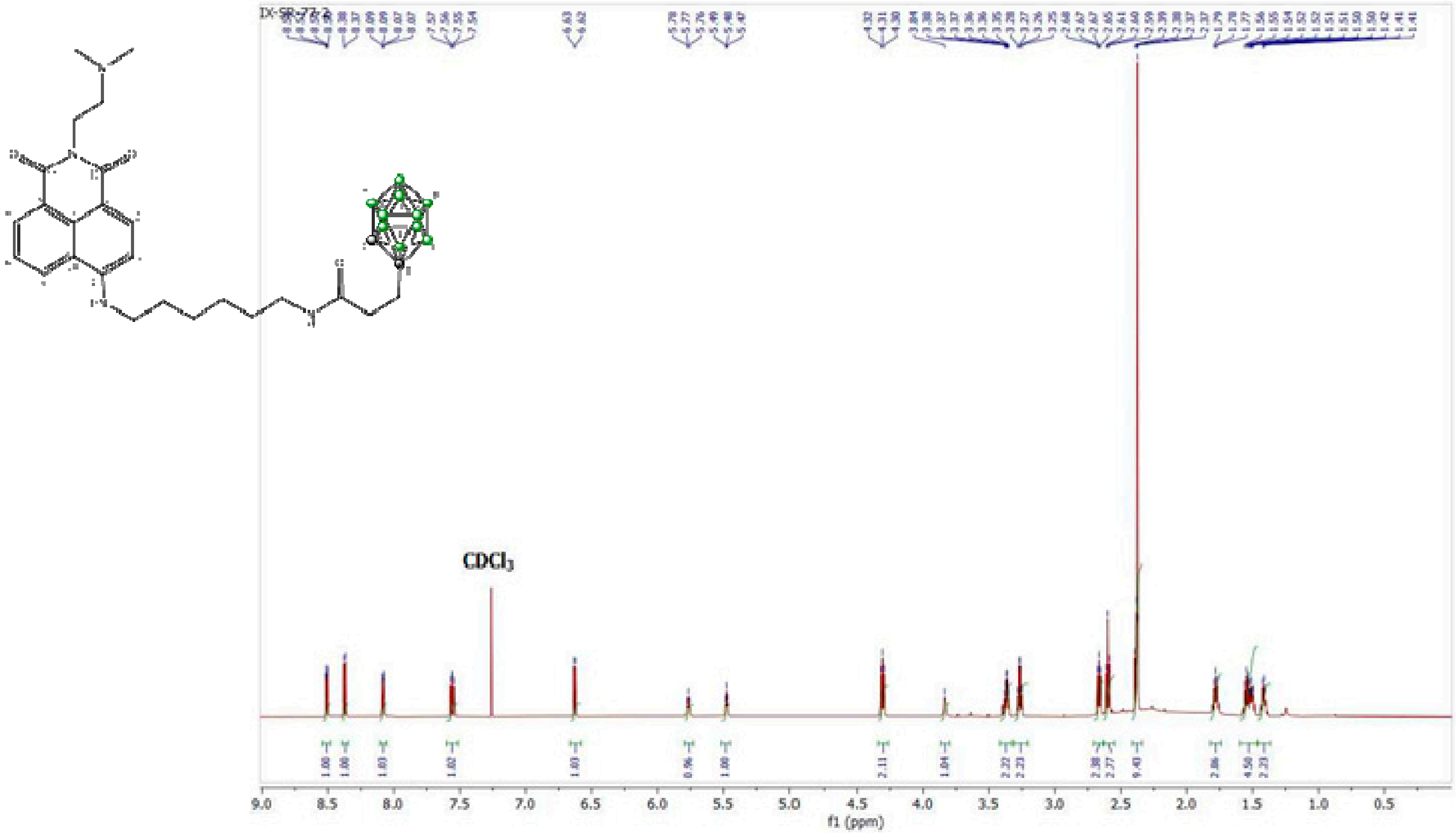

Figure S157. ^{1}H NMR spectrum of **40**.

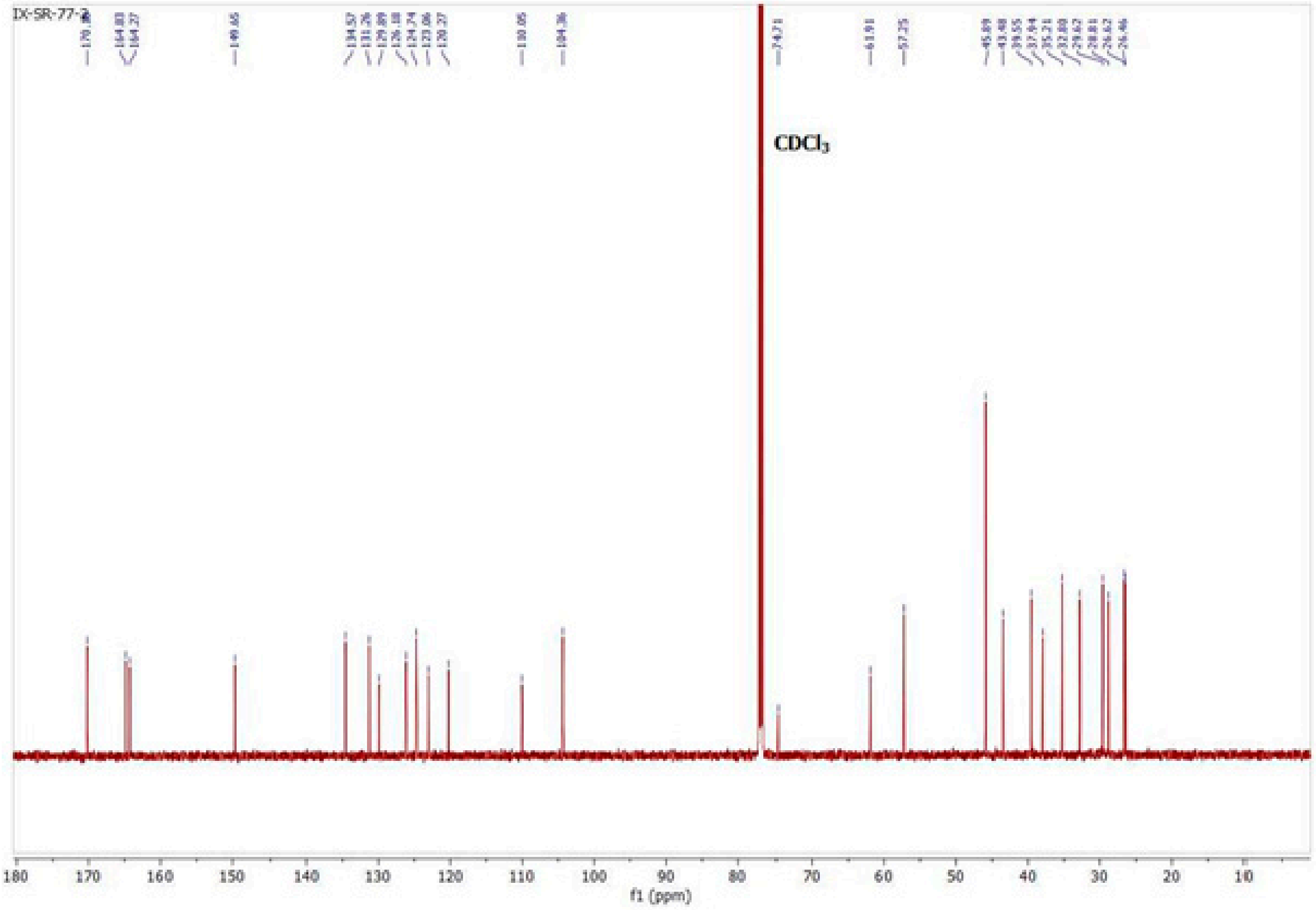

Figure S158. ^{13}C NMR spectrum of **40**.

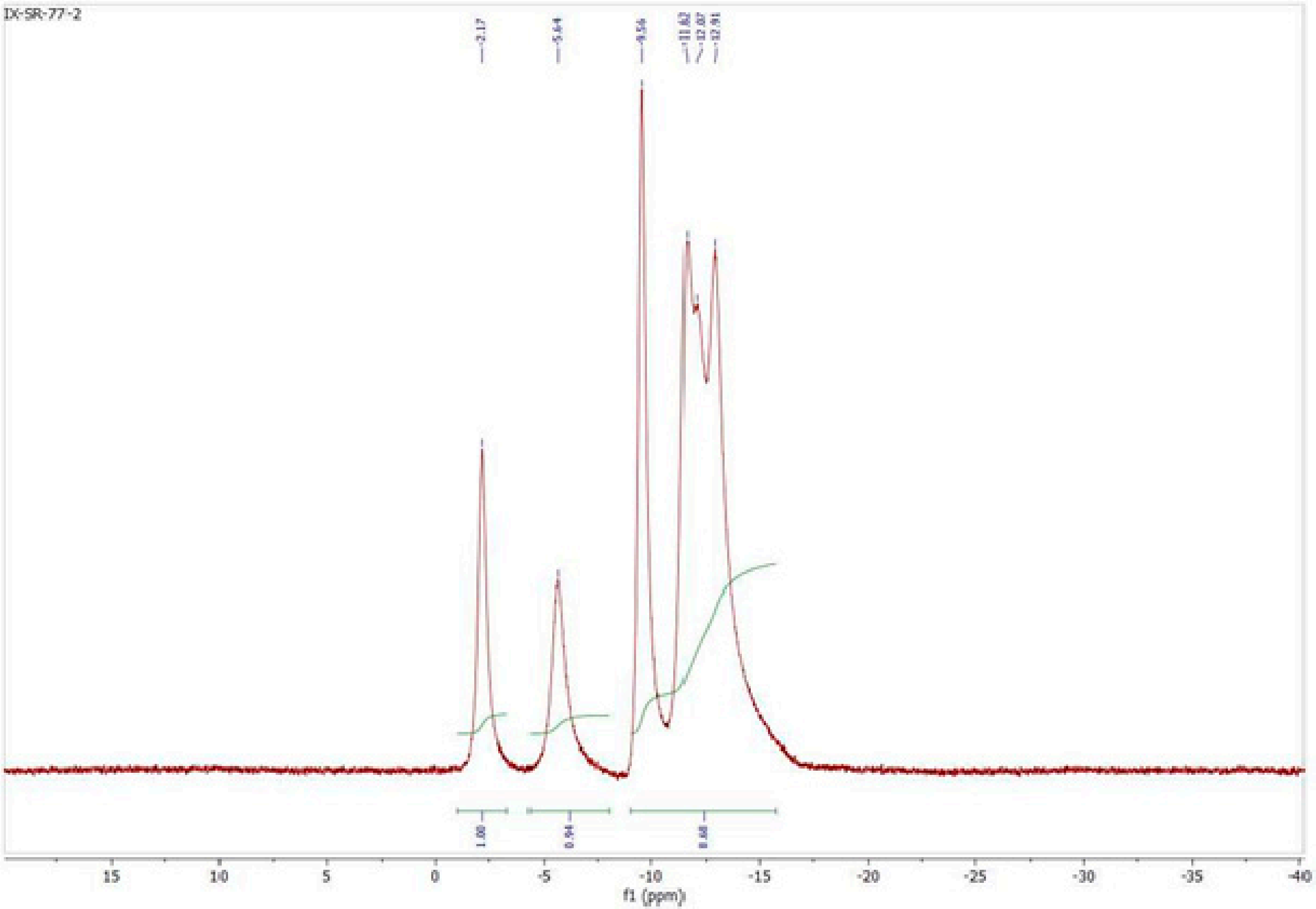

Figure S159. ^{11}B NMR spectrum of **40**.

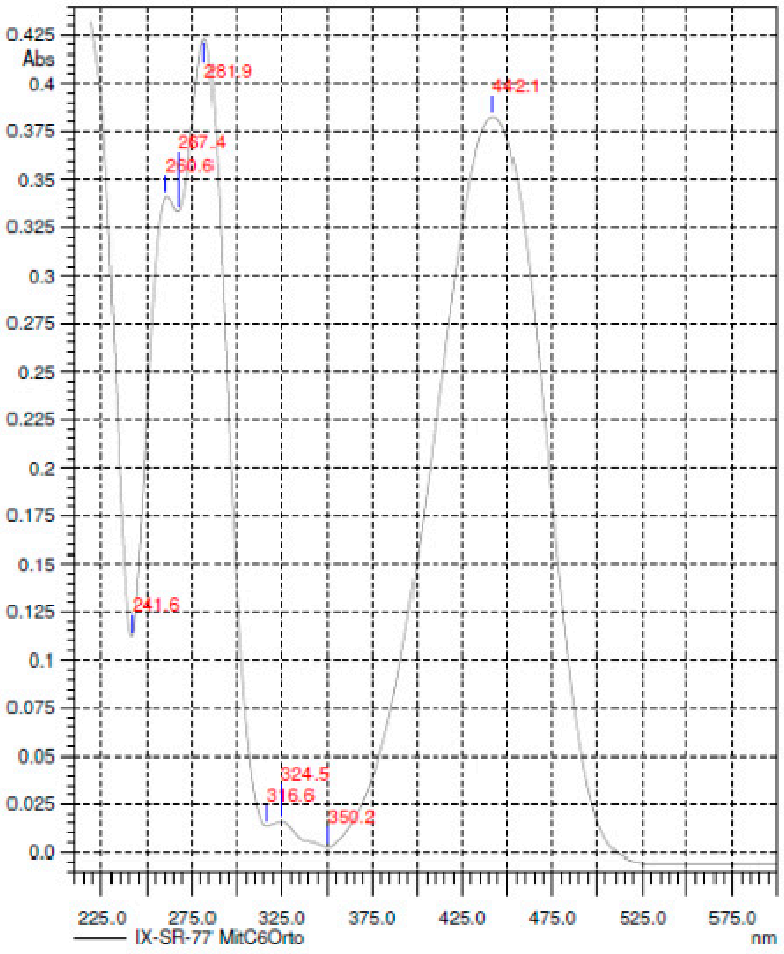

Figure S160. UV spectrum of **40**.

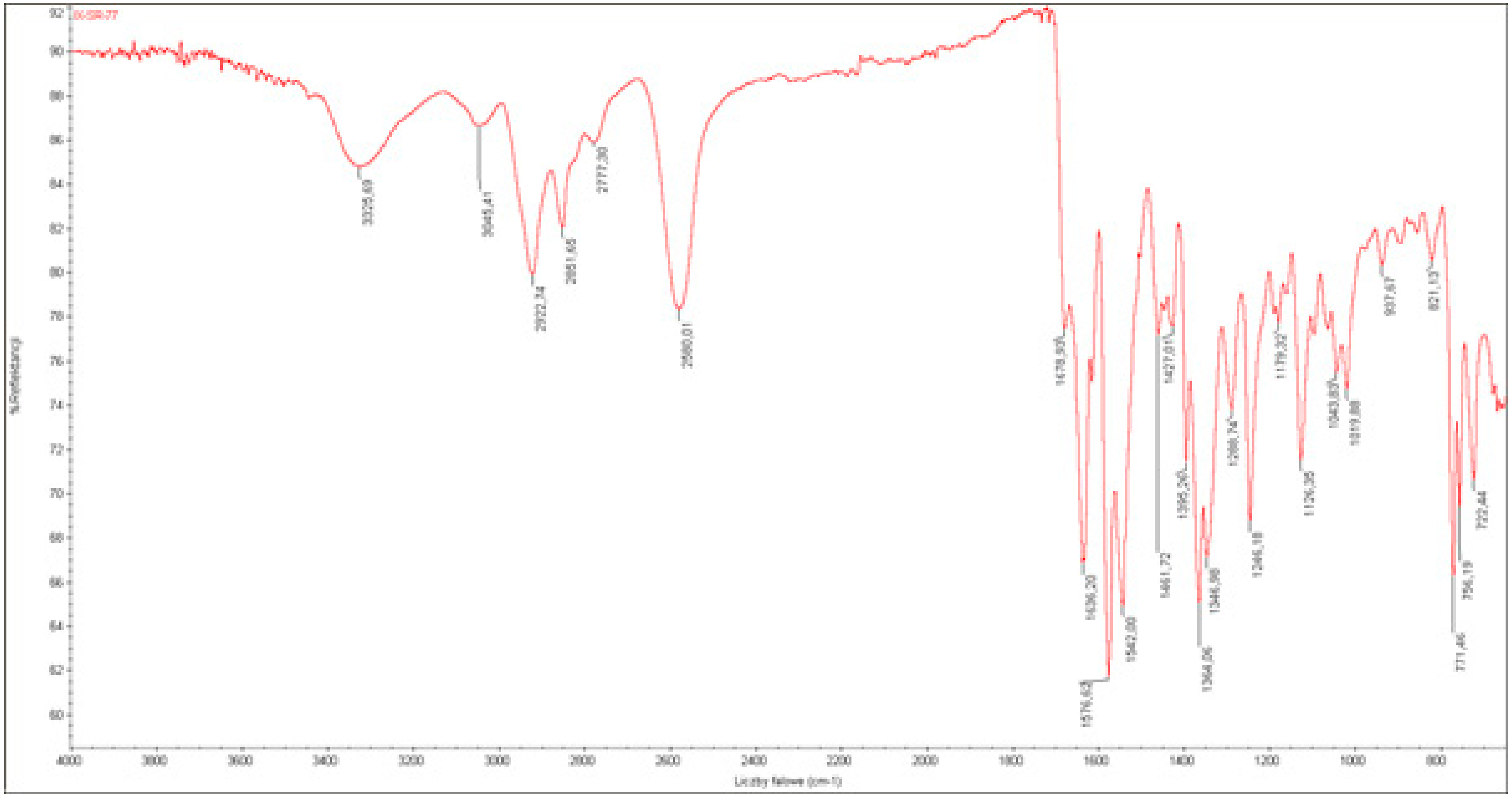

Figure S161. IR spectrum of **40**.

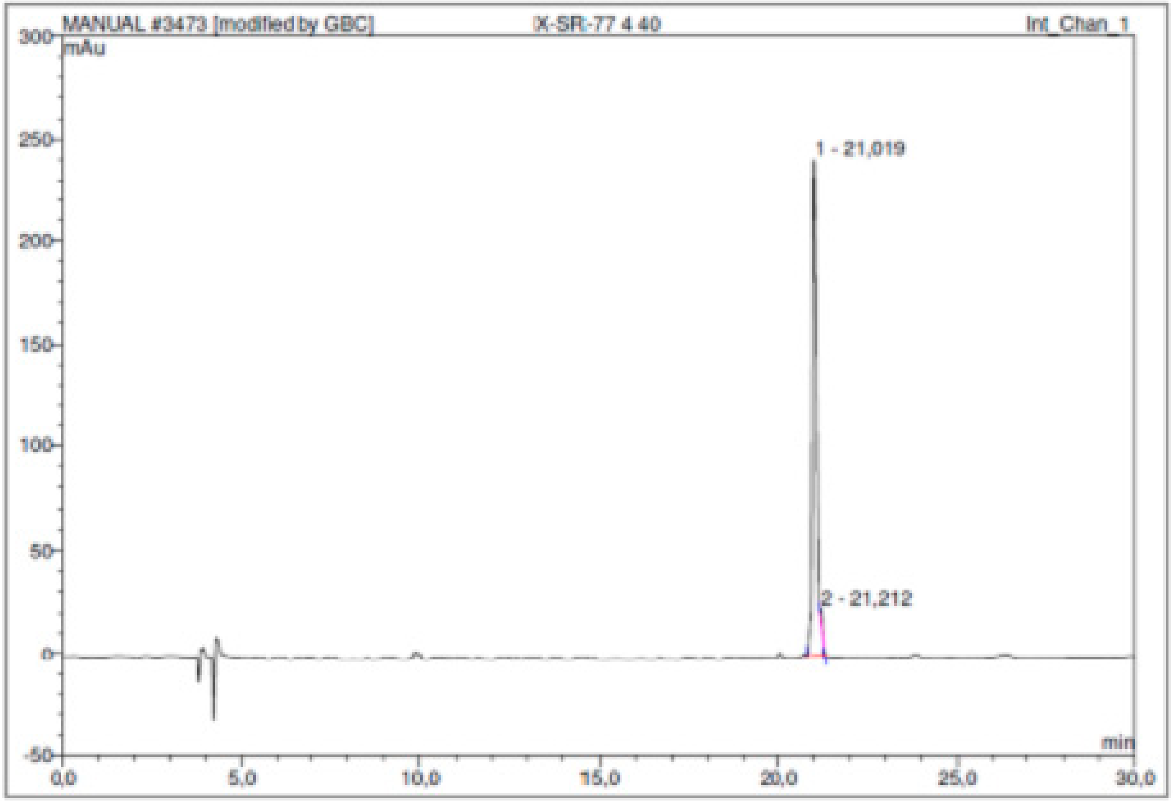

No.	Ret.Time min	Peak Name	Height mAu	Area mAu*min	Rel.Area %	Amount	Type
1	21,02	n.a.	241,531	31,920	98,46	n.a.	BMB*
2	21,21	n.a.	7,057	0,498	1,54	n.a.	Rd*
Total:			248,588	32,418	100,00	0,000	

Figure S162. HPLC chromatogram of **40**.

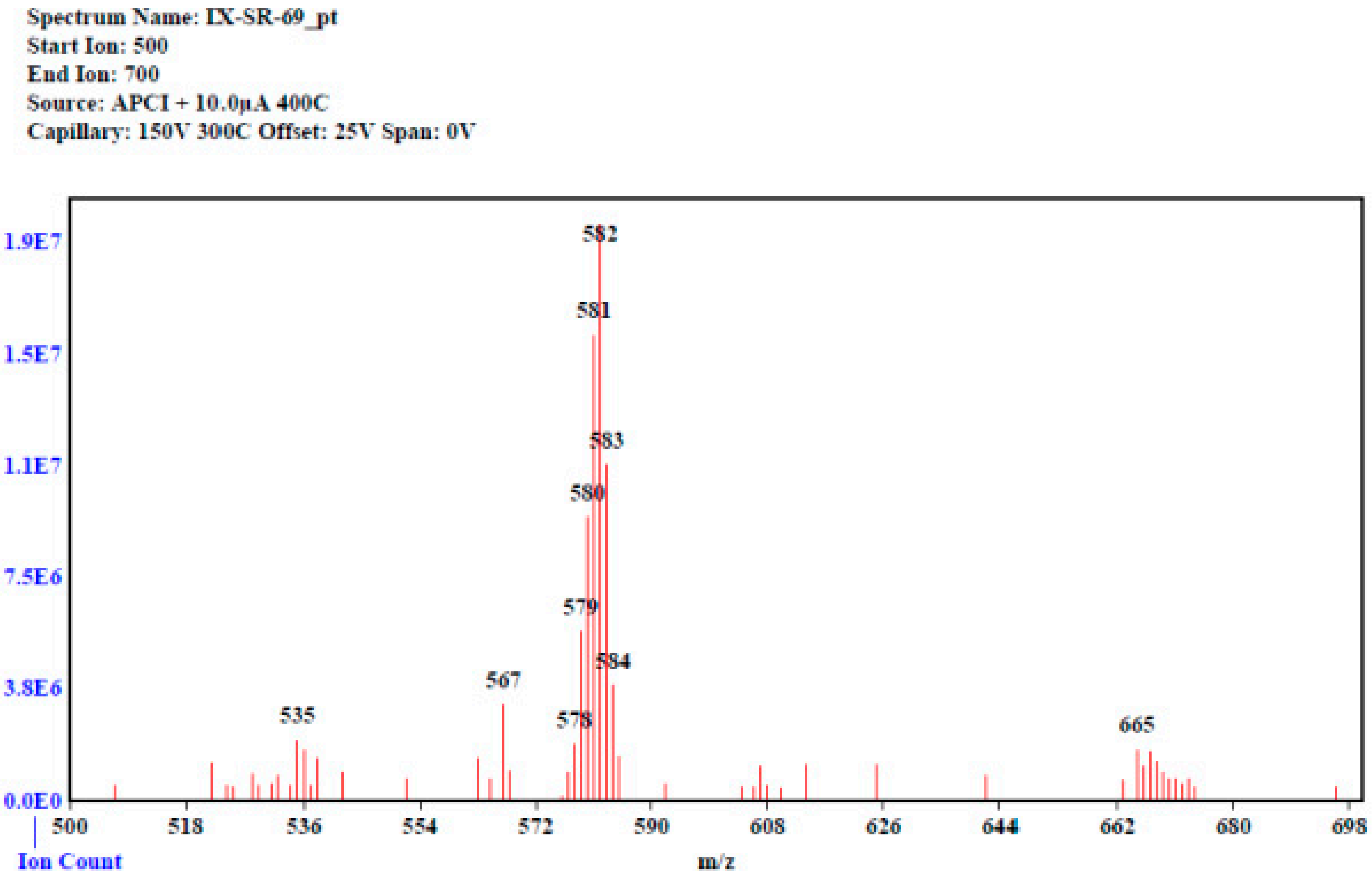

Figure S163. MS spectrum of **40**.

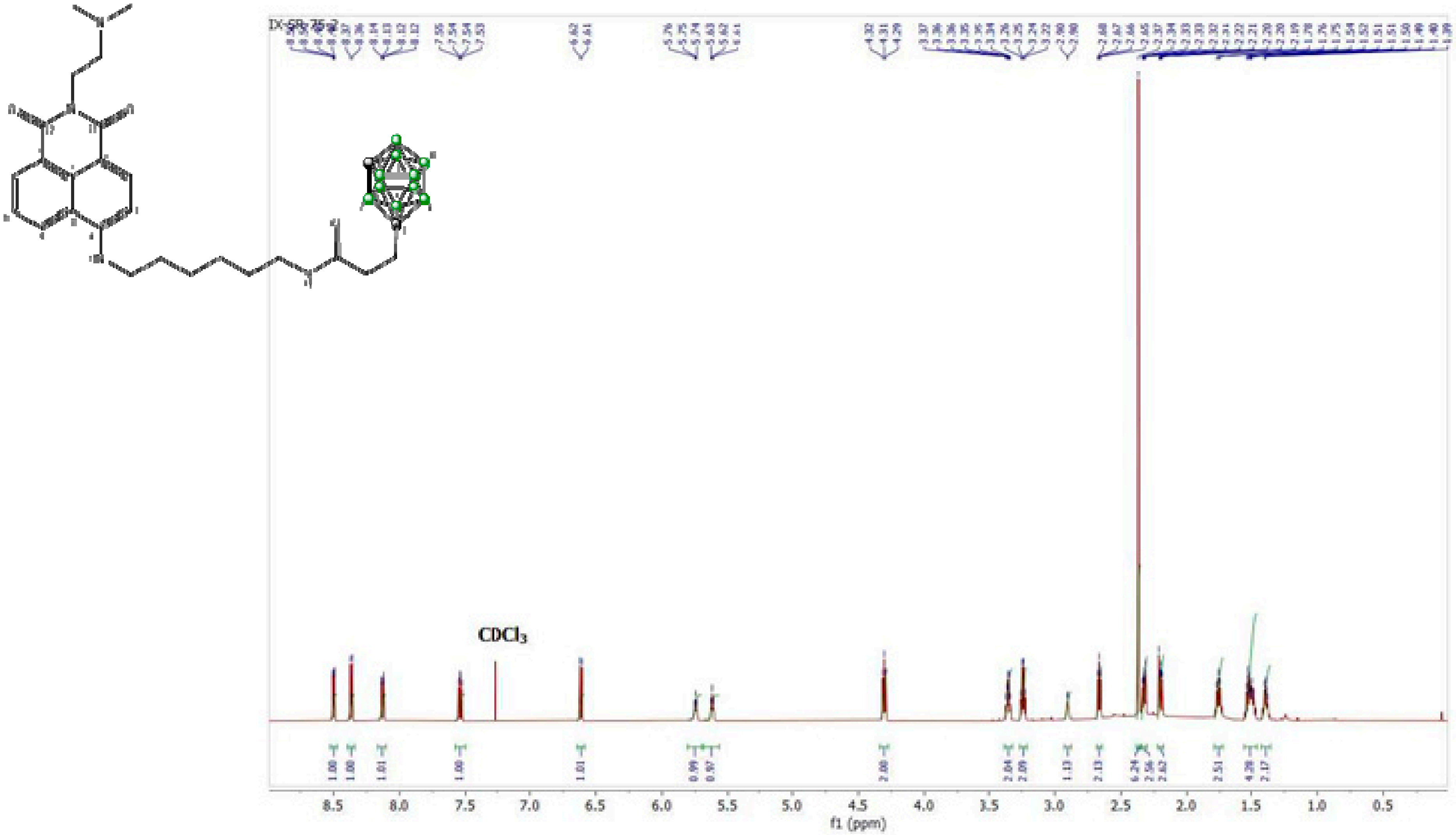

Figure S164. ^{1}H NMR spectrum of **41**.

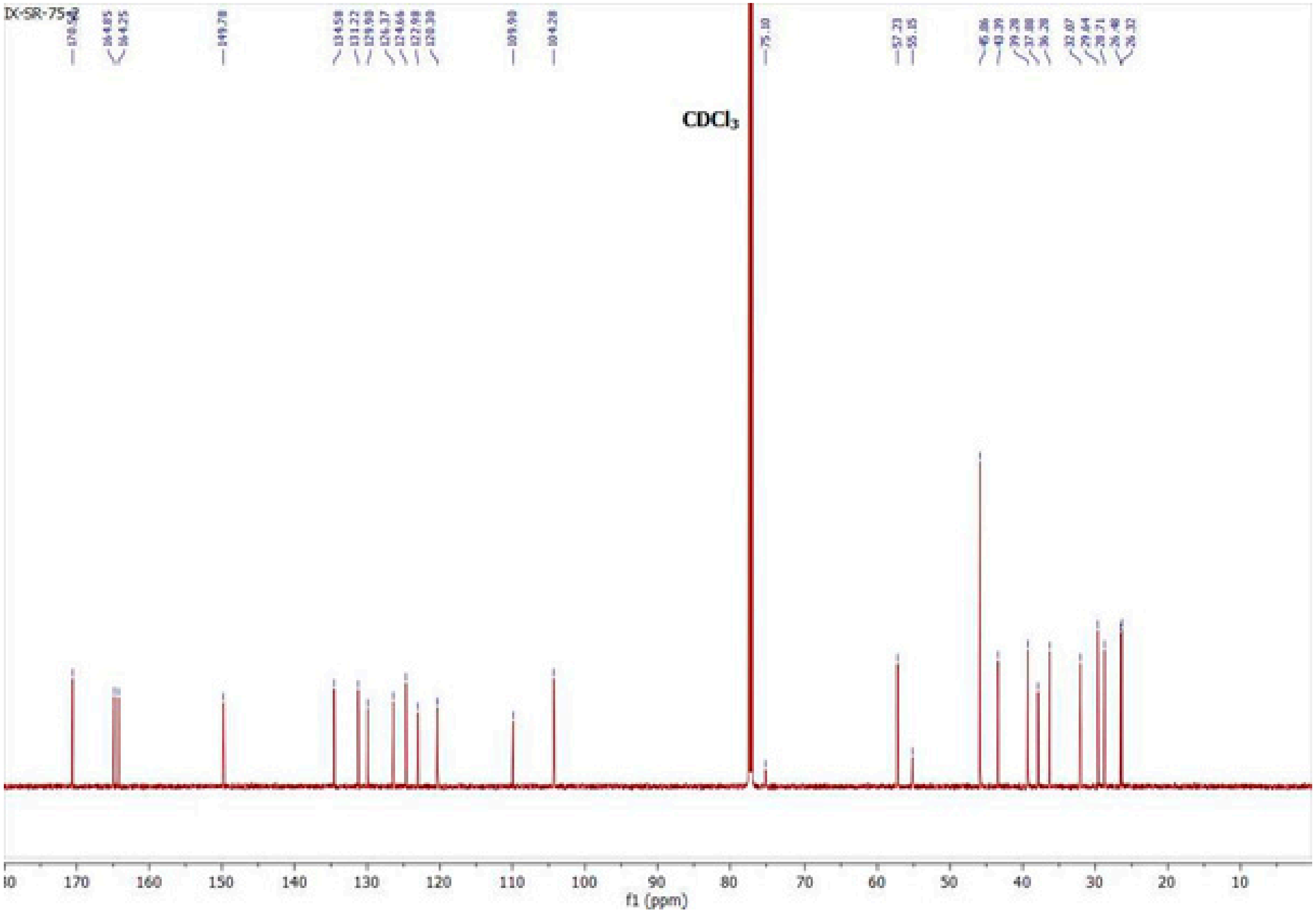

Figure S165. ^{13}C NMR spectrum of **41**.

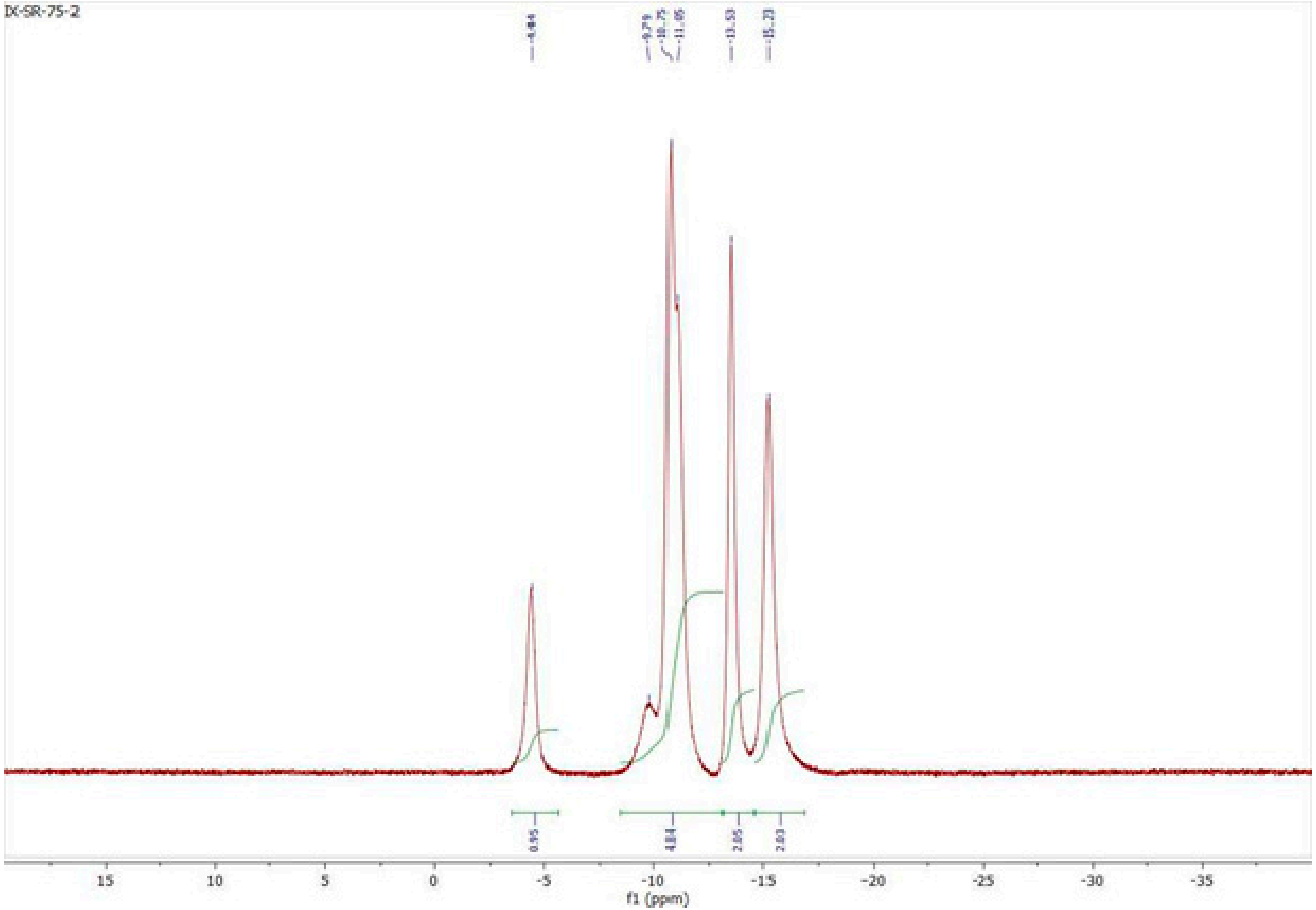

Figure S166. ^{11}B NMR spectrum of **41**.

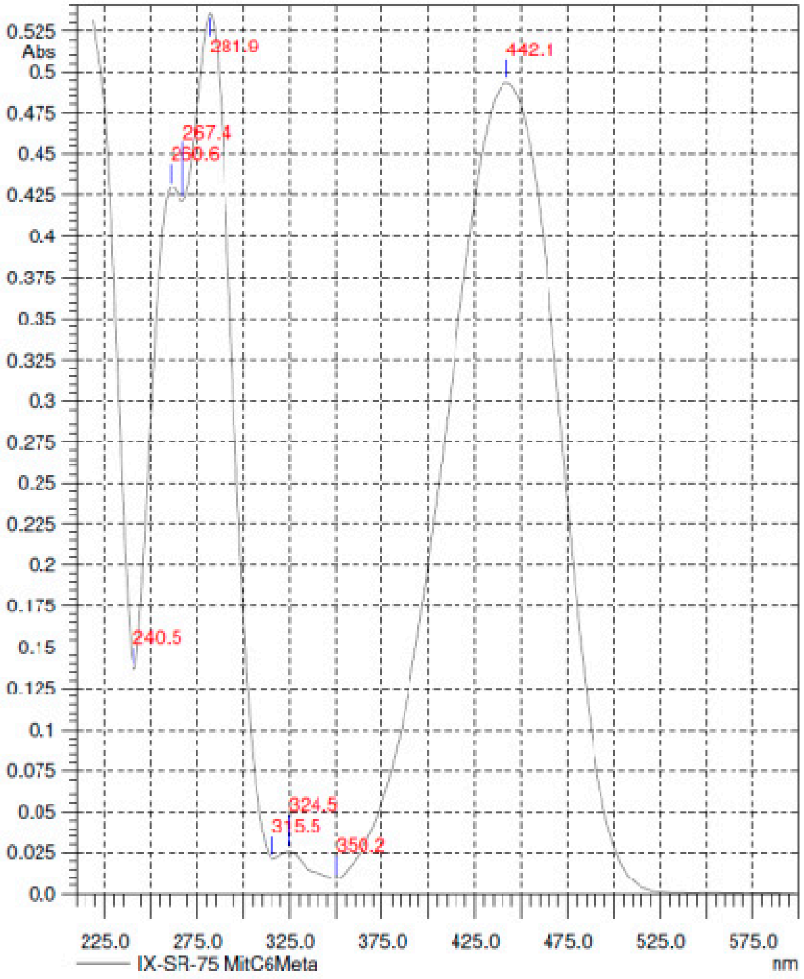

Figure S167. UV spectrum of **41.**

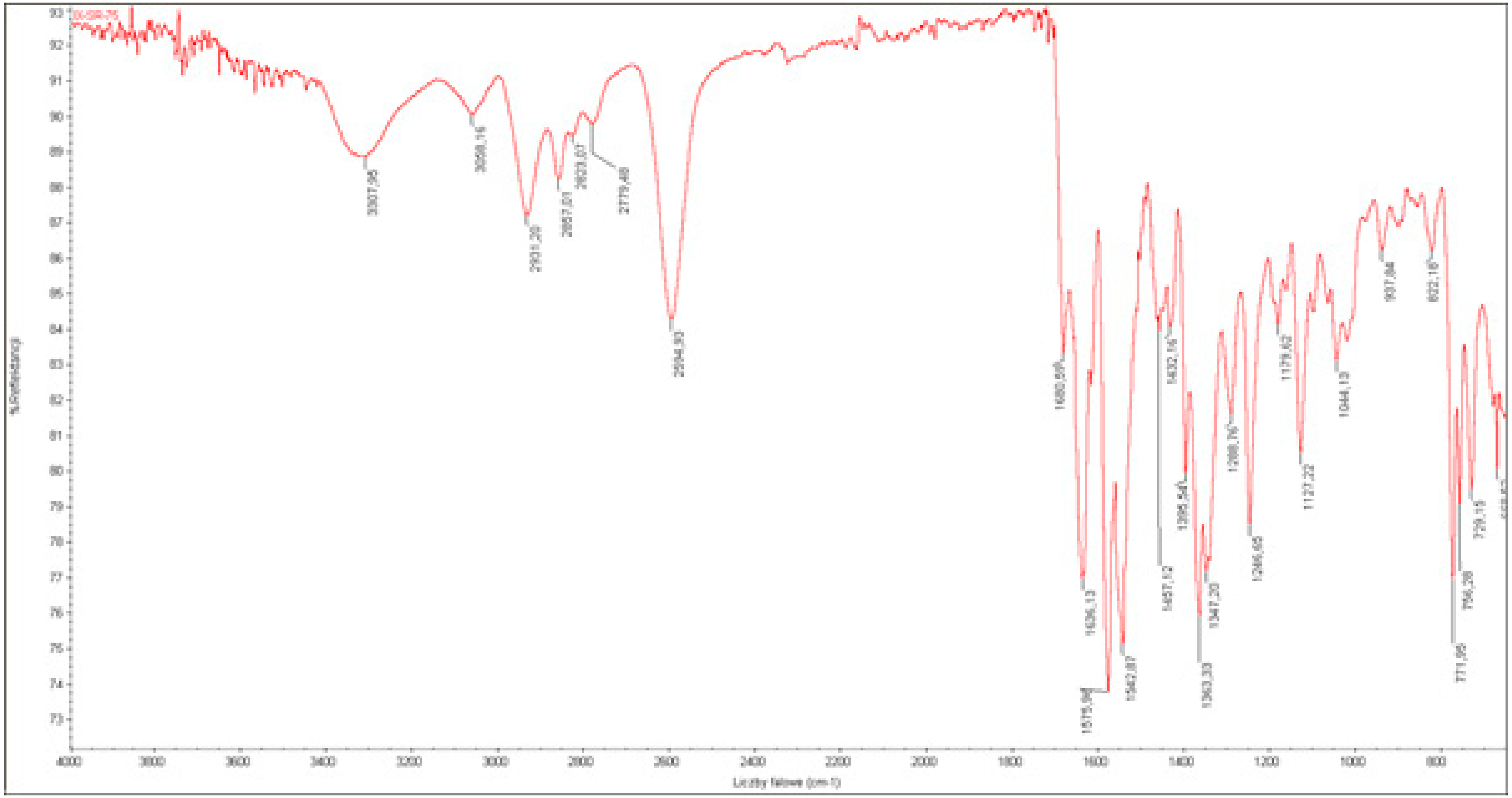

Figure S168. IR spectrum of **41**.

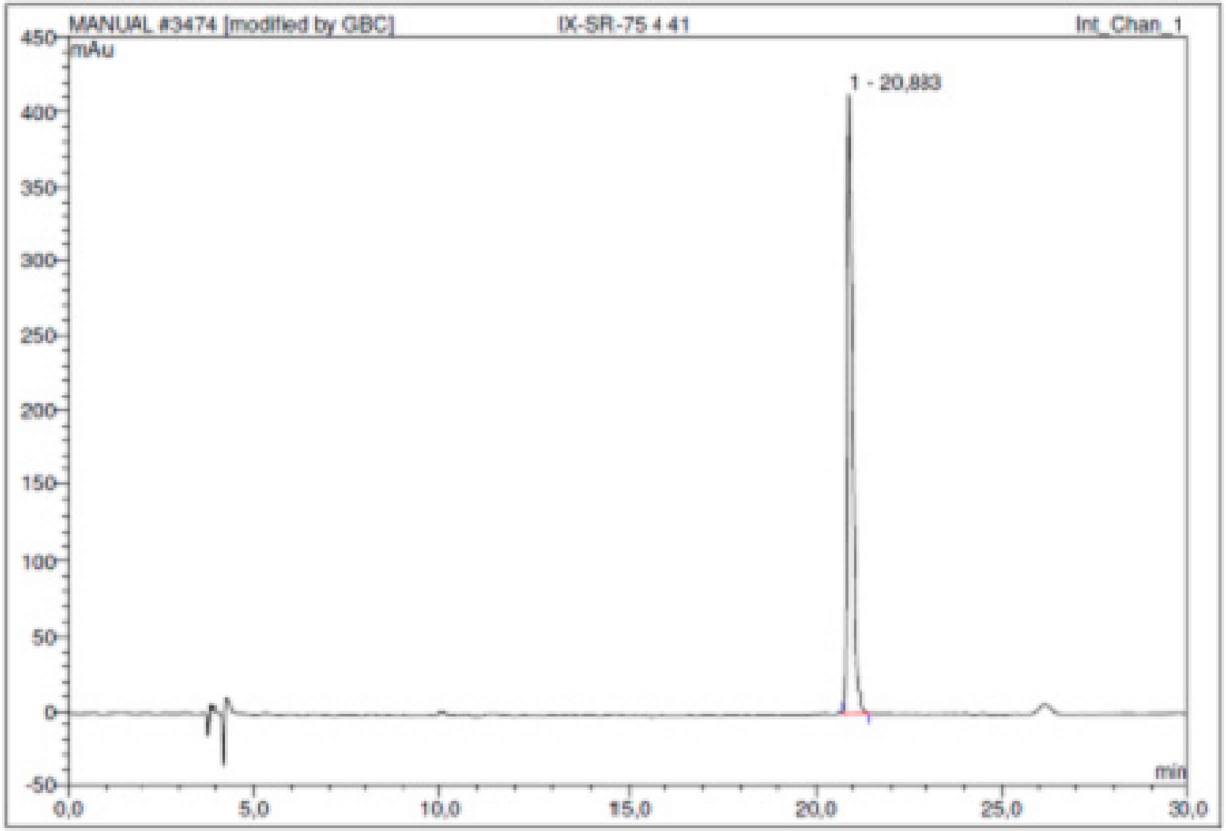

No.	Ret.Time min	Peak Name	Height mAu	Area mAu*min	Rel.Area %	Amount	Type
1	20,88	n.a.	413,168	68,858	100,00	n.a.	BMB
Total:			413,168	68,858	100,00	0,000	

Figure S169. HPLC chromatogram of **41**.

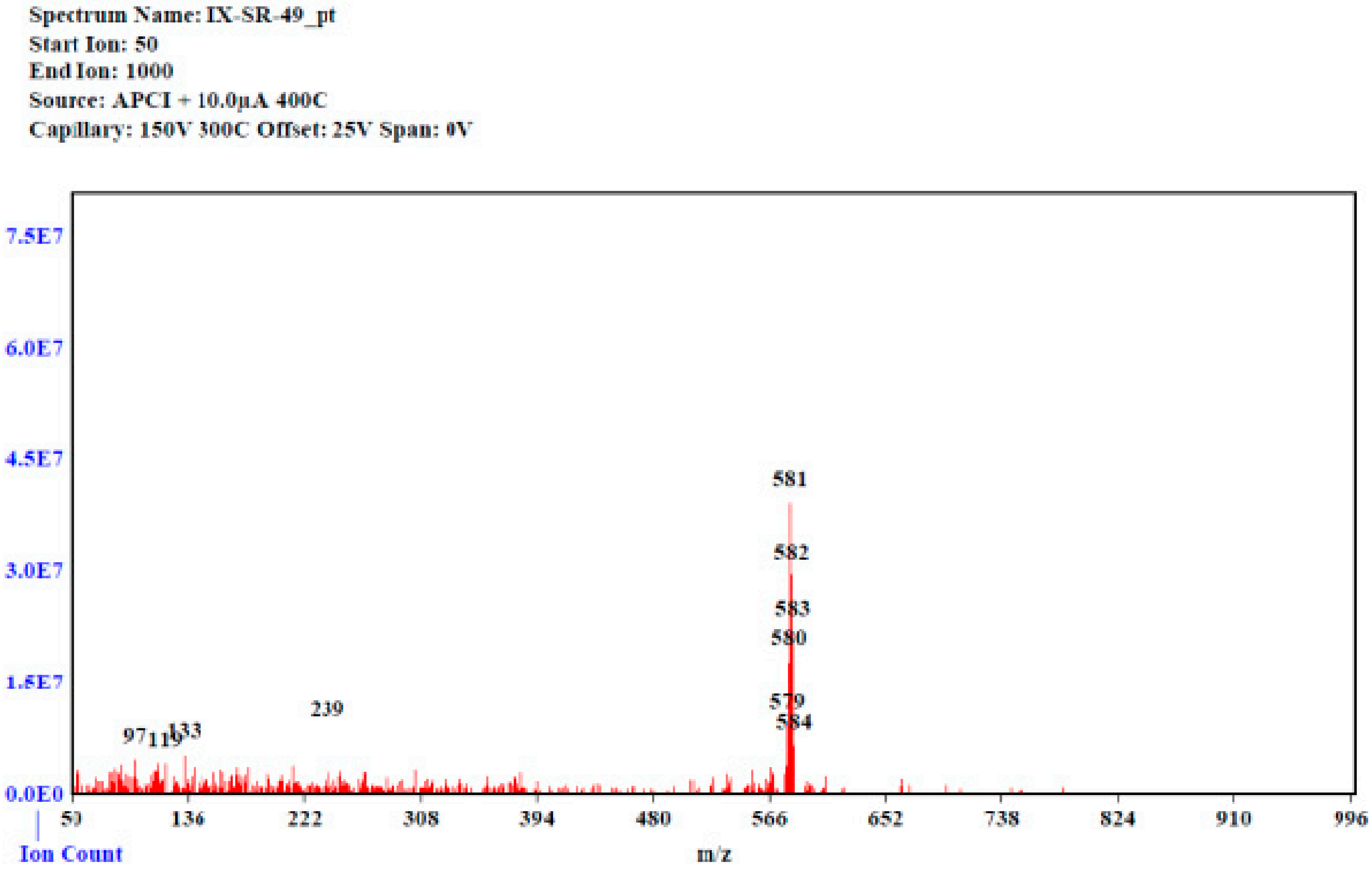

Figure S170. MS spectrum of **41**.

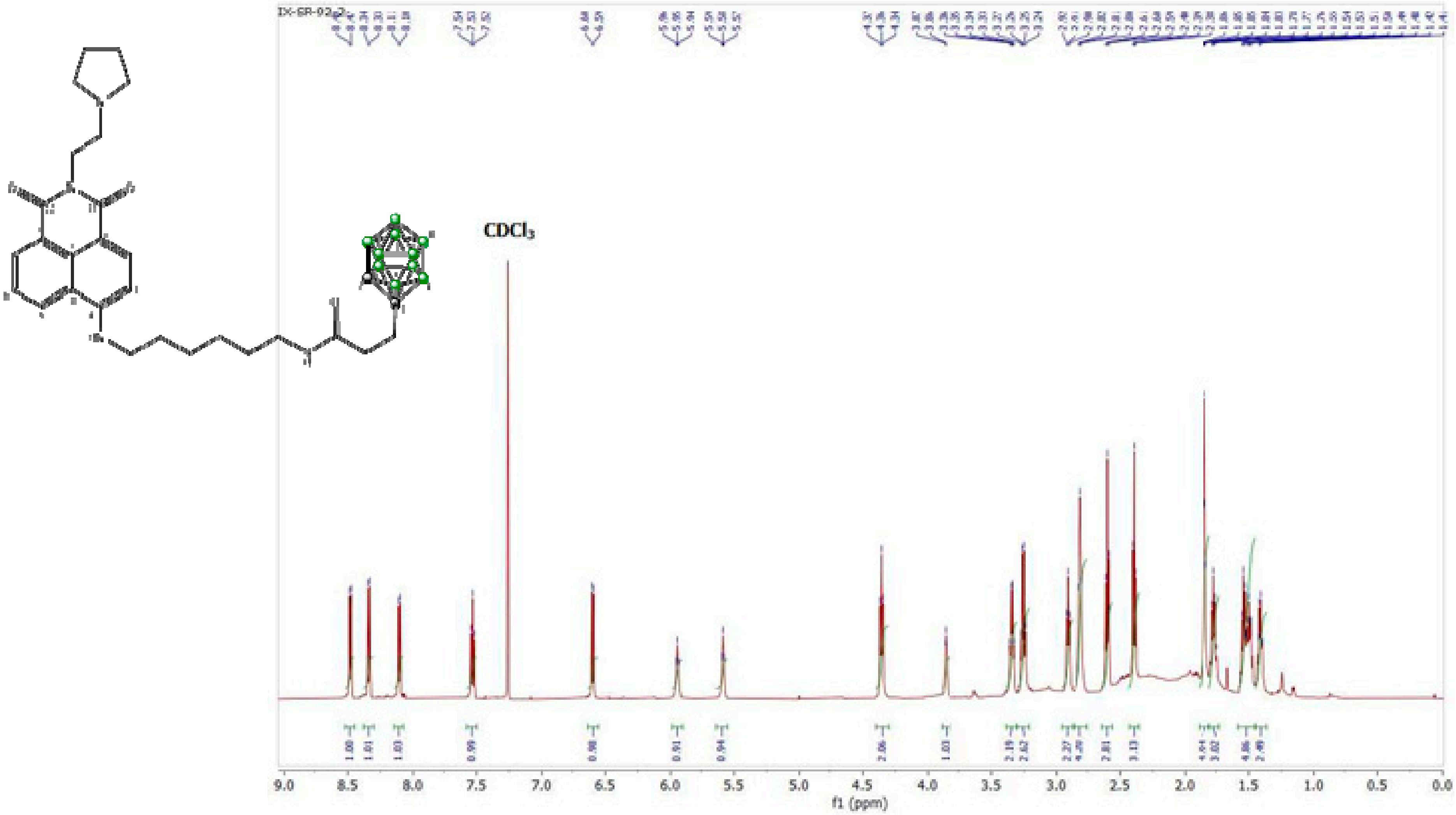

Figure S171. ^{1}H NMR spectrum of **42**.

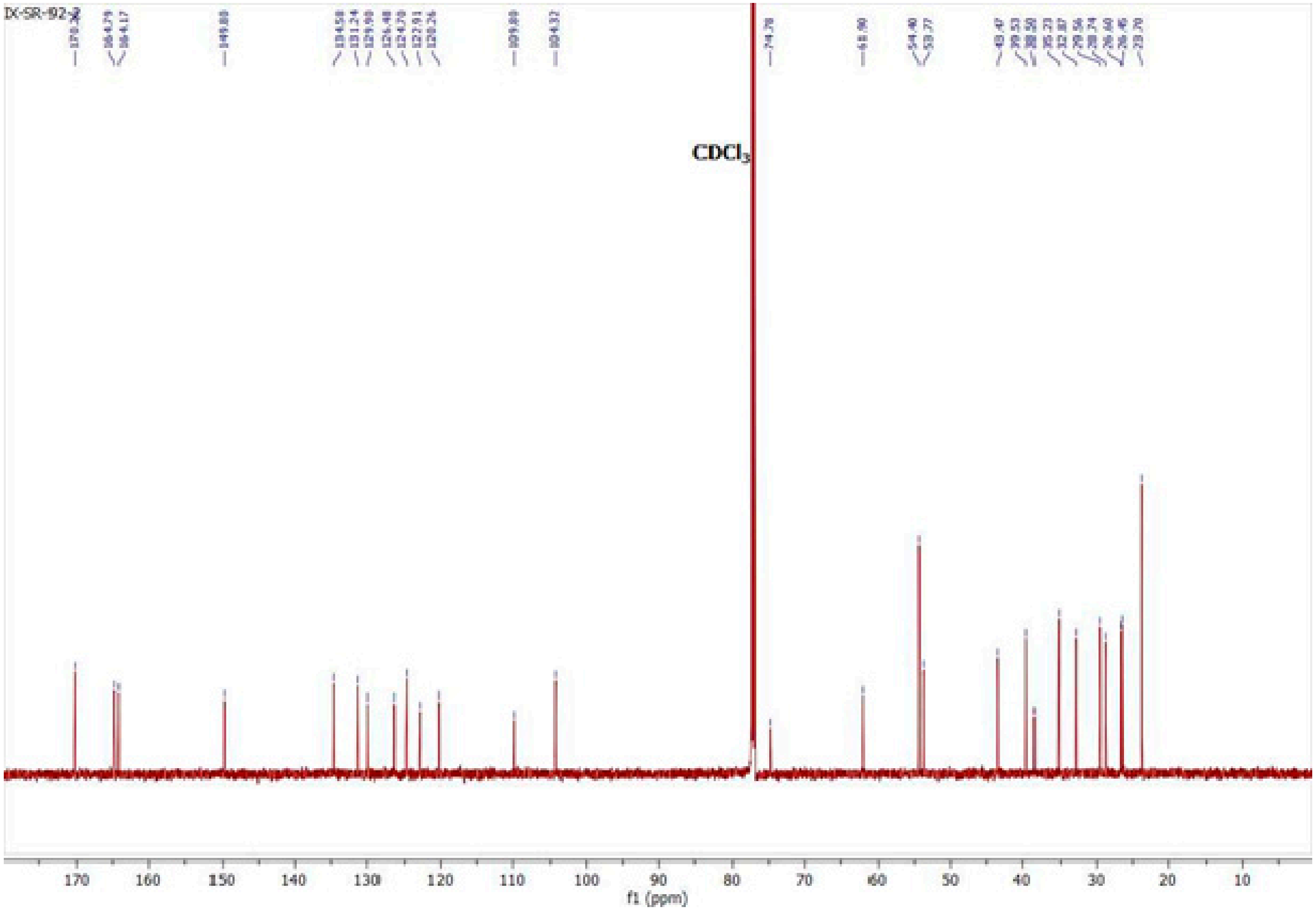

Figure S172. ^{13}C NMR spectrum of **42**.

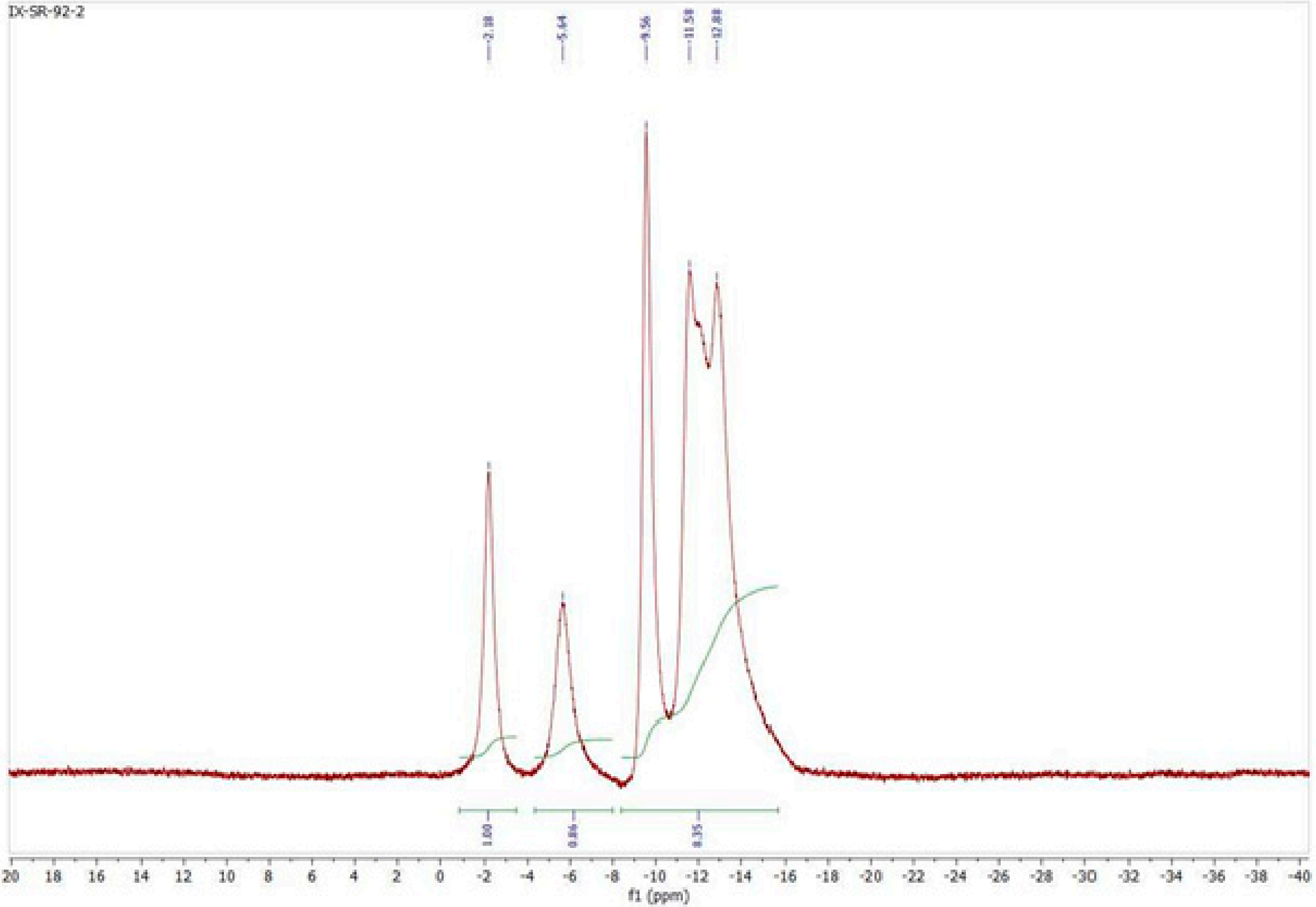

Figure S173. ^{11}B NMR spectrum of **42**.

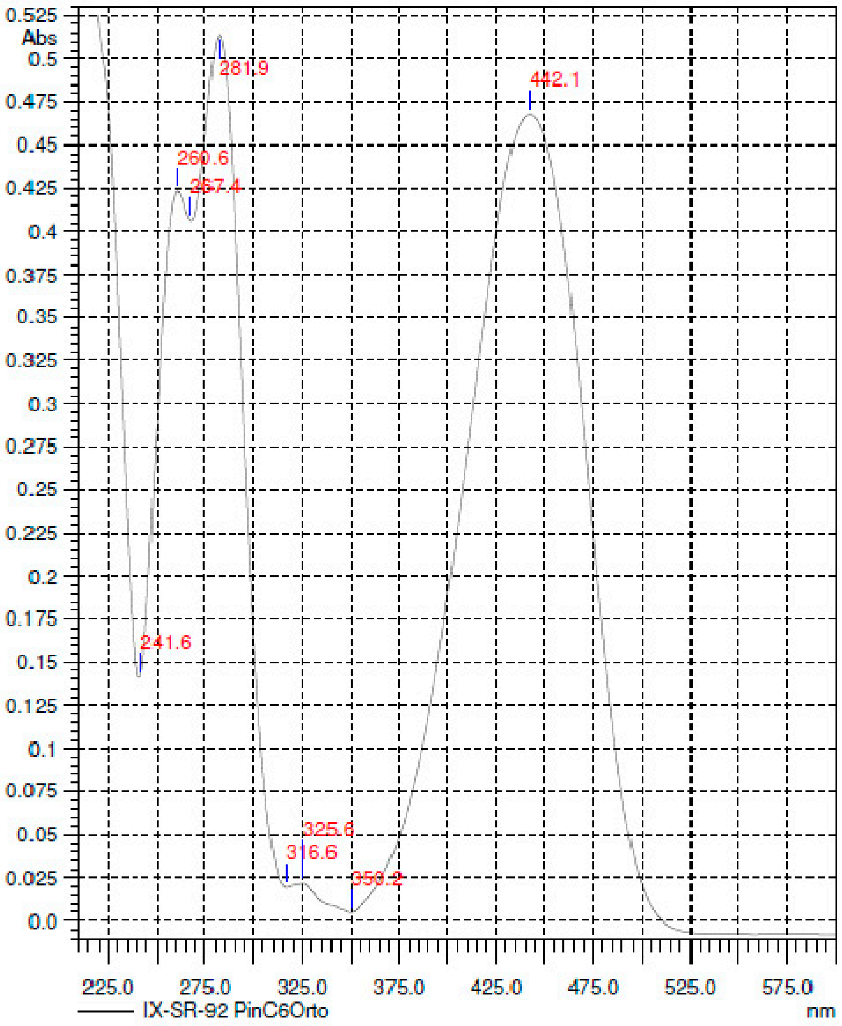

Figure S174. UV spectrum of **42.**

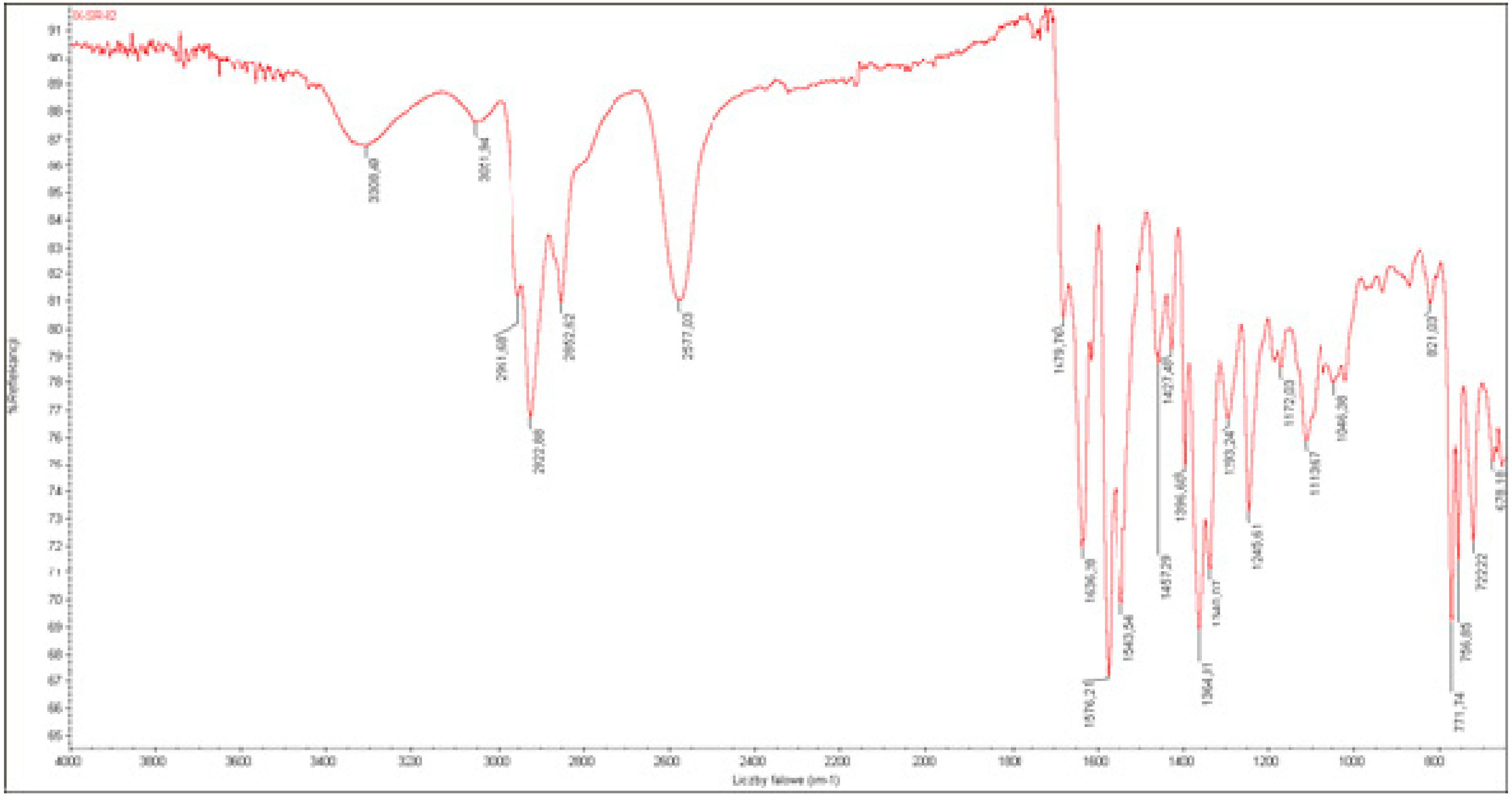

Figure S175. IR spectrum of **42**.

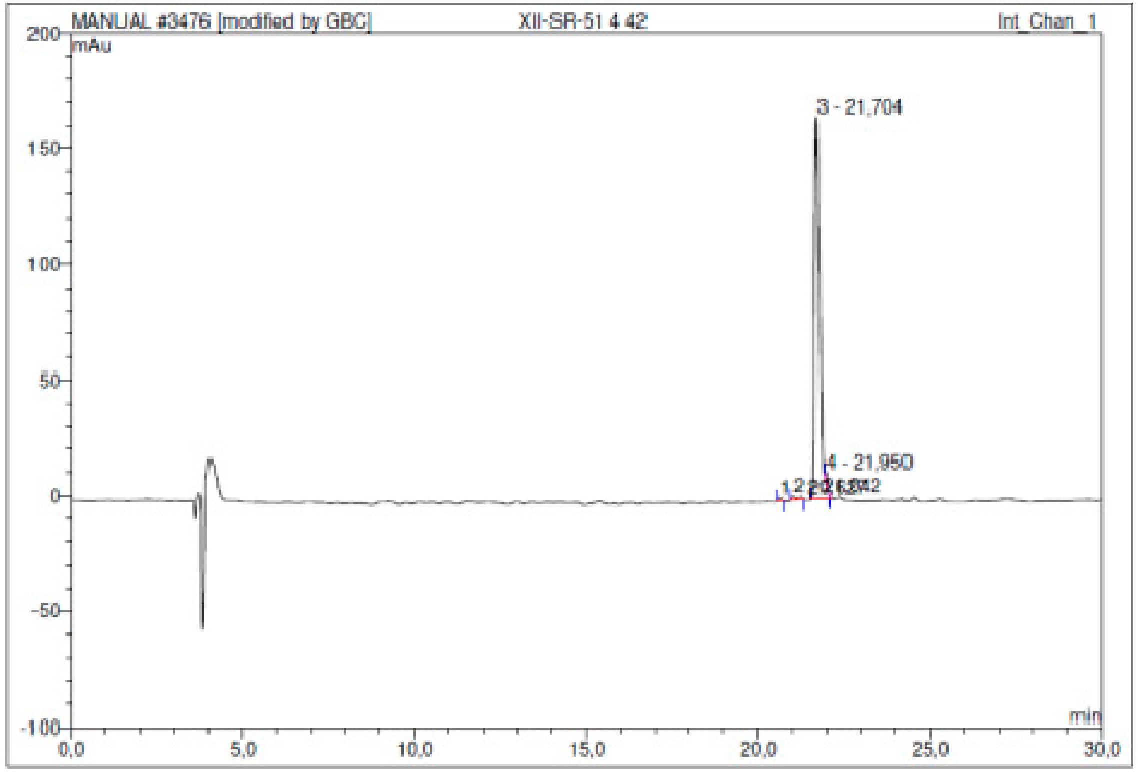

No.	Ret.Time min	Peak Name	Height mAu	Area mAu*min	Rel.Area %	Amount	Type
1	20,63	n.a.	1,508	0,152	0,58	n.a.	BMB*
2	21,04	n.a.	1,273	0,276	1,06	n.a.	BMB*
3	21,70	n.a.	164,647	25,445	97,38	n.a.	BMB*
4	21,95	n.a.	3,417	0,256	0,98	n.a.	Rd*
Total:			170,845	26,130	100,00	0,000	

Figure S176. HPLC chromatogram of **42**.

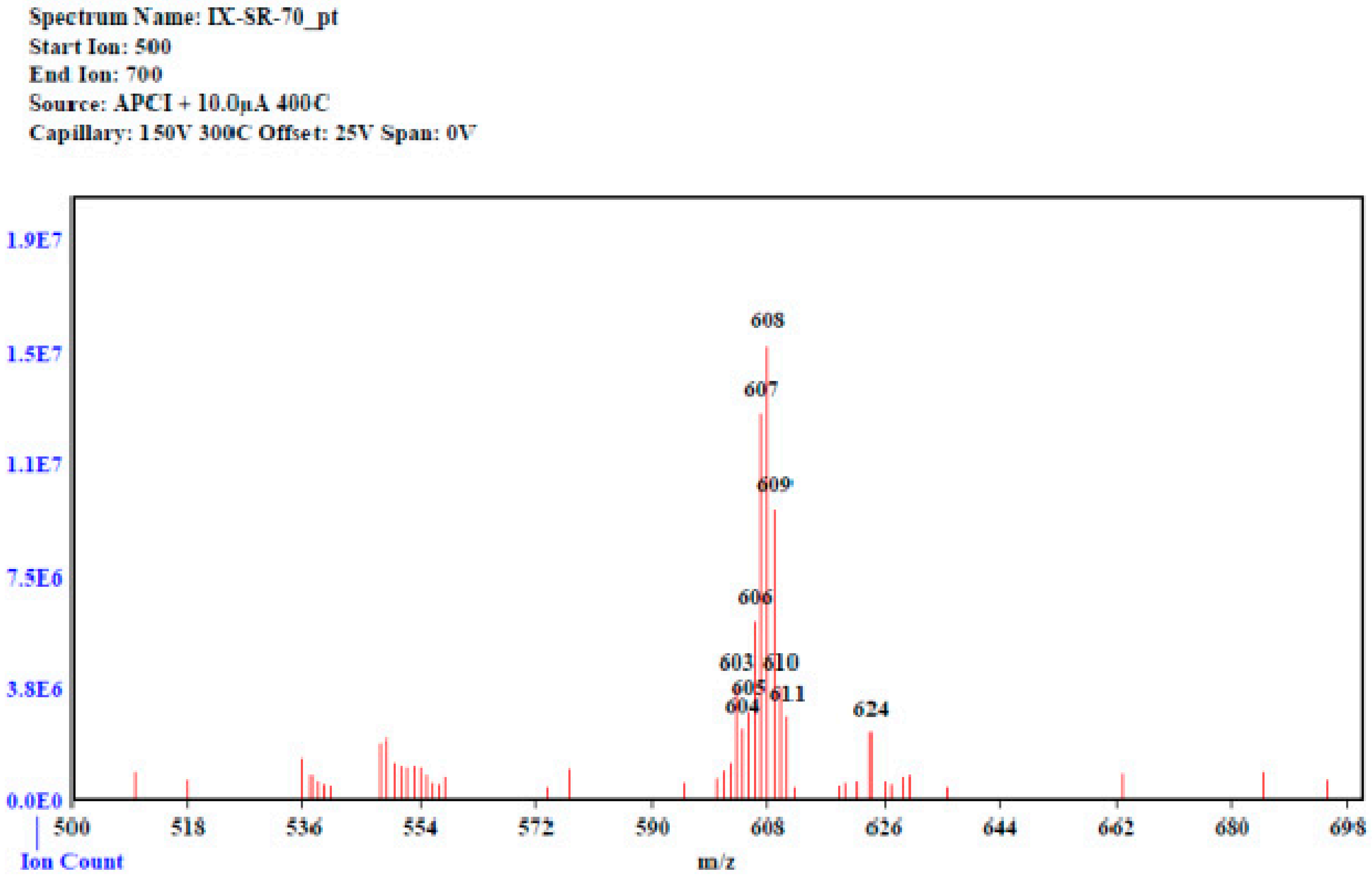

Figure S177. MS spectrum of **42**.

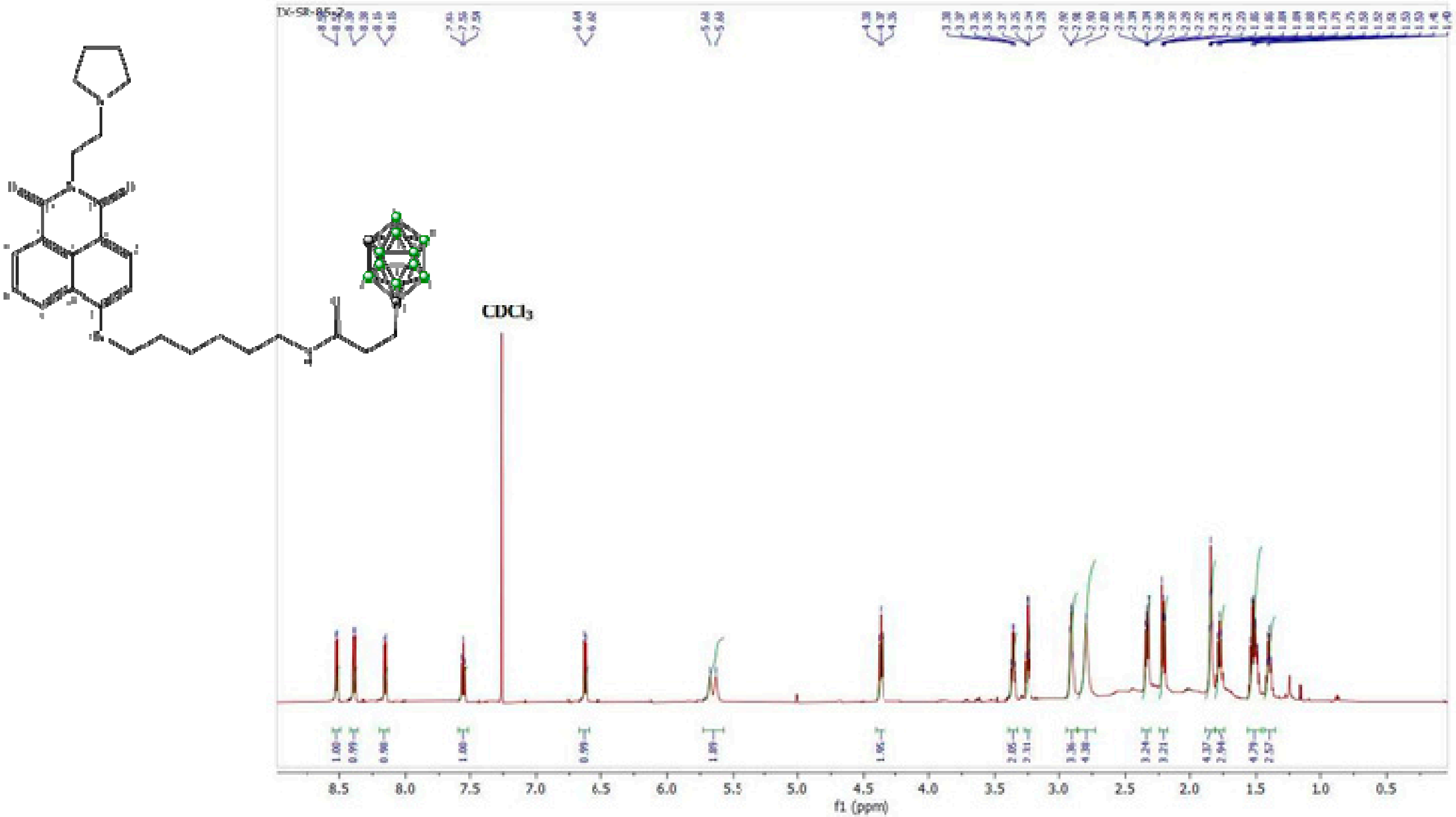

Figure S178. ^{1}H NMR spectrum of **43**.

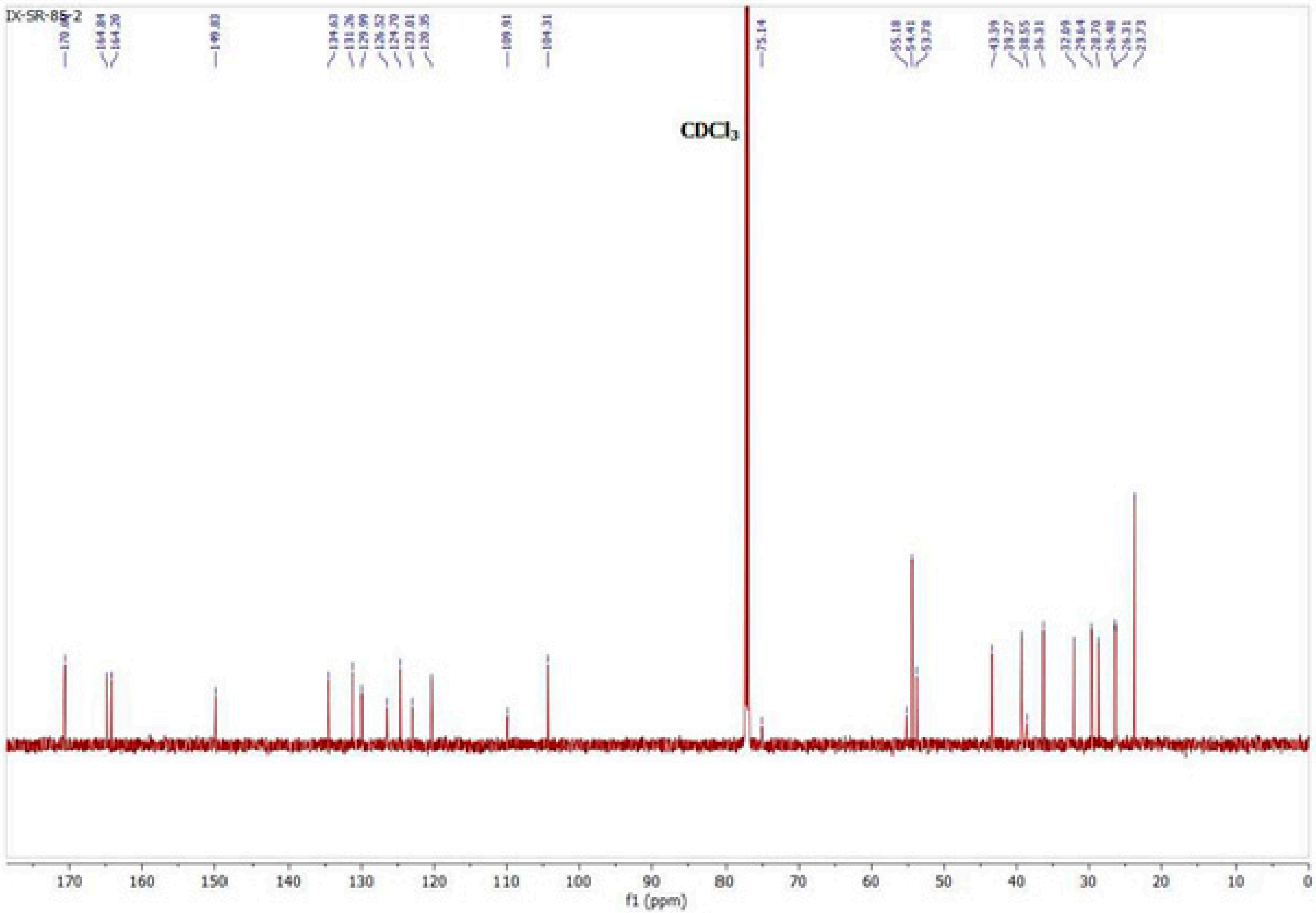

Figure S179. ^{13}C NMR spectrum of **43**.

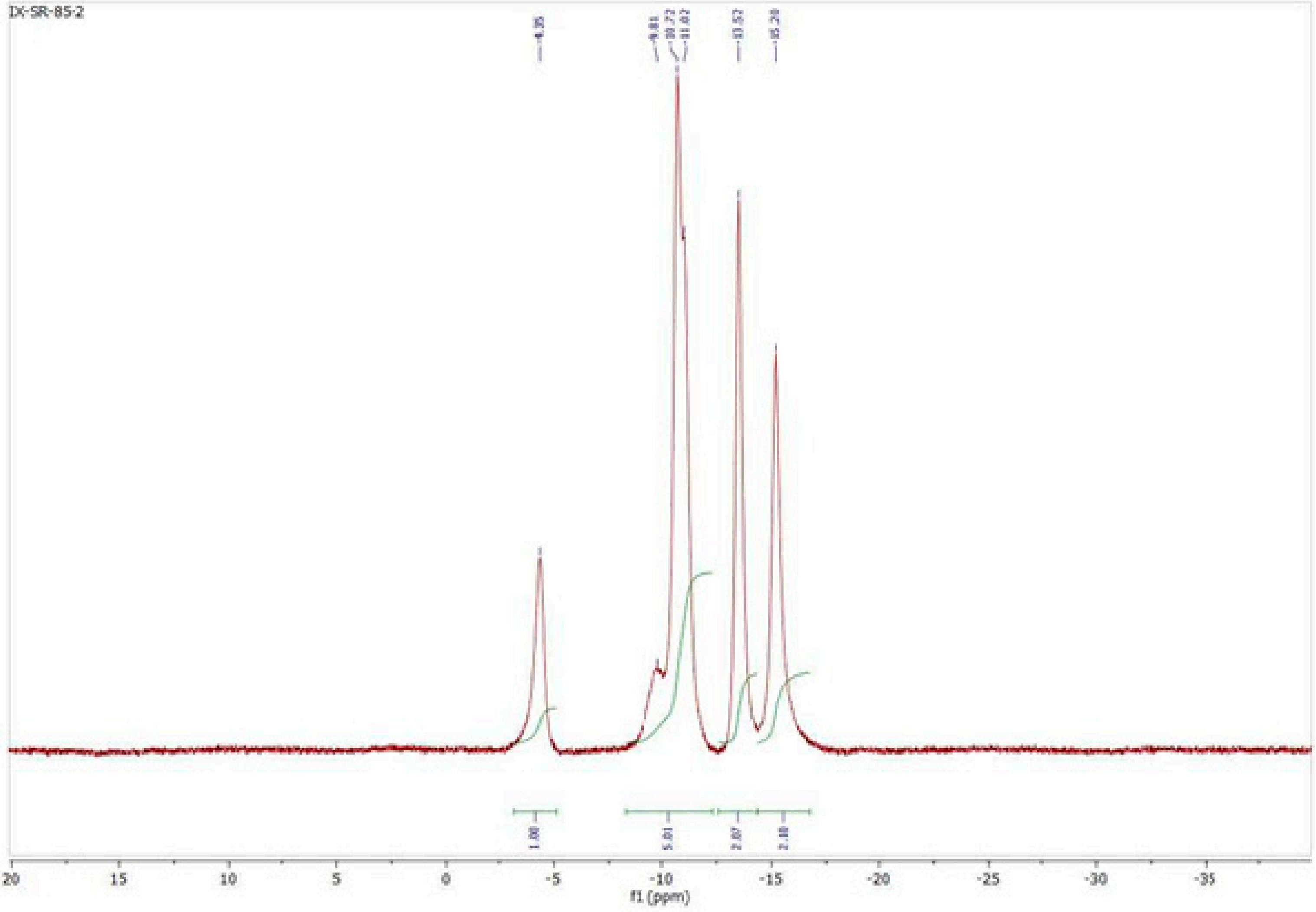

Figure S180. ^{11}B NMR spectrum of **43**.

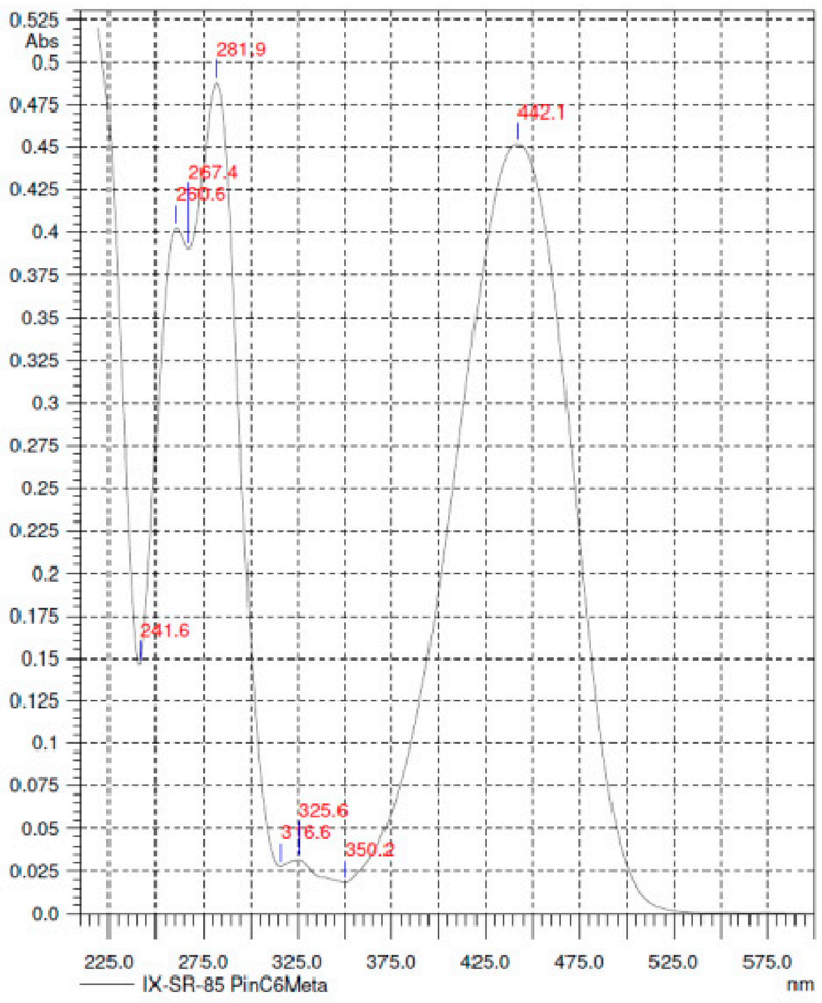

Figure S181. UV spectrum of **43**.

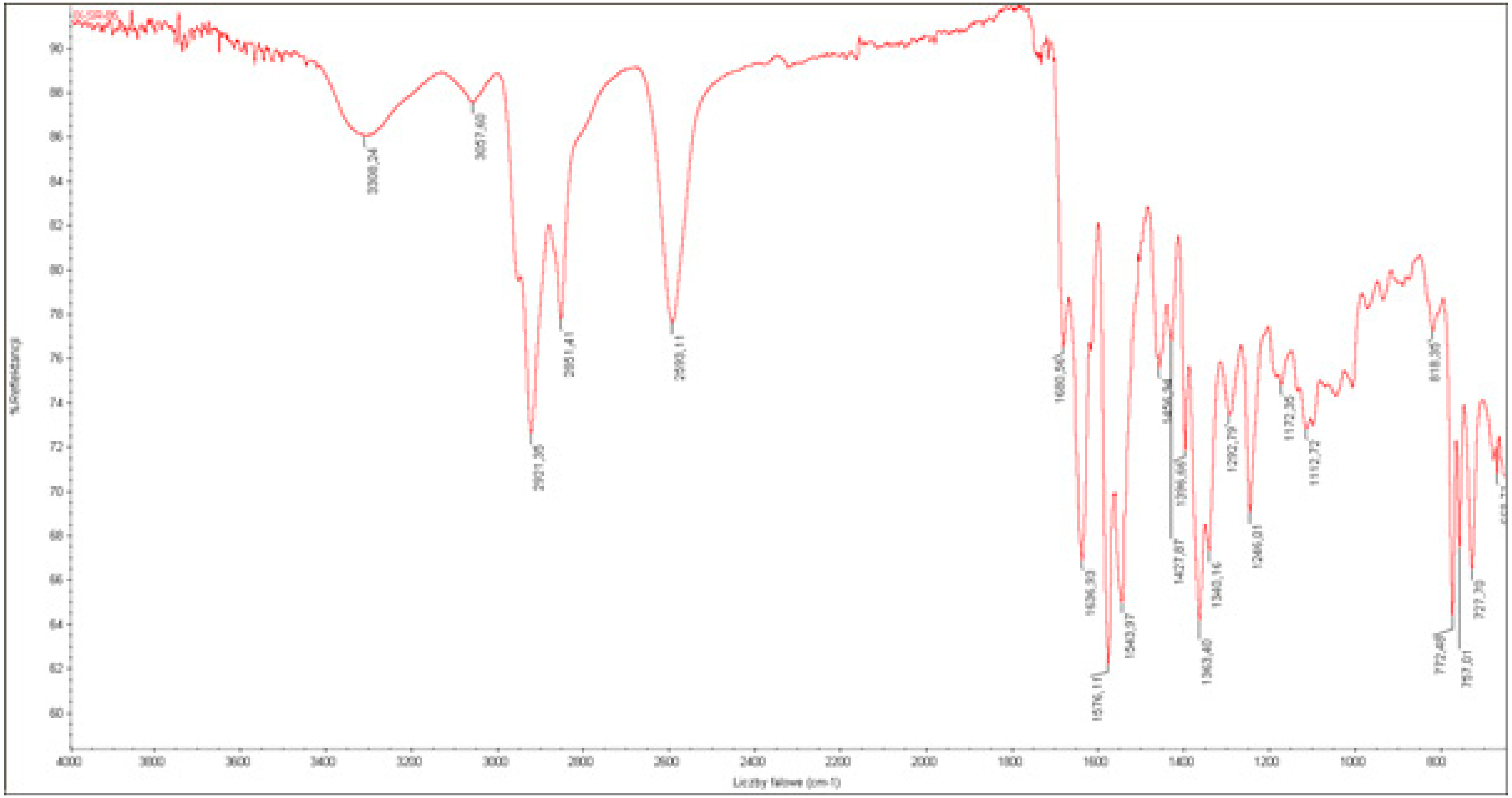

Figure S182. IR spectrum of **43**.

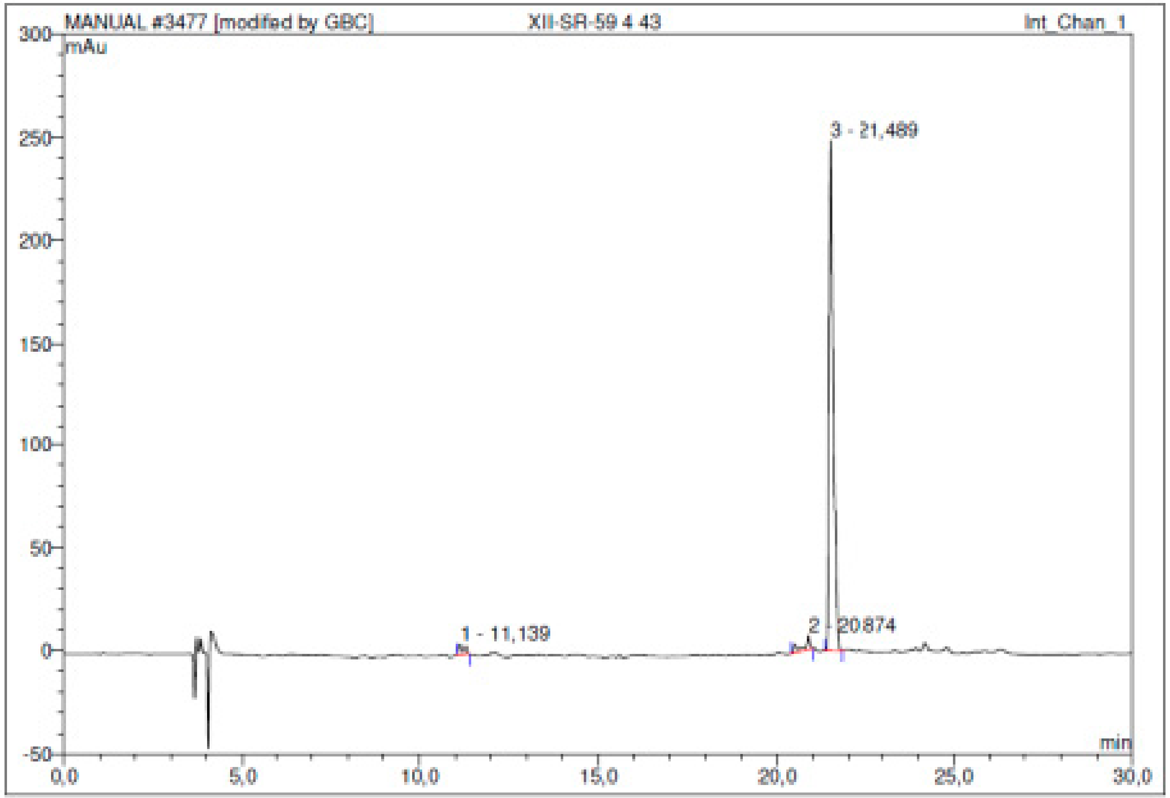

No.	Ret.Time min	Peak Name	Height mAu	Area mAu*min	Rel.Area %	Amount	Type
1	11,14	n.a.	4,957	0,831	2,25	n.a.	BMB*
2	20,87	n.a.	6,701	1,374	3,72	n.a.	BMB*
3	21,49	n.a.	248,362	34,780	94,04	n.a.	BMB
Total:			260,021	36,986	100,00	0,000	

Figure S183. HPLC chromatogram of **43**.

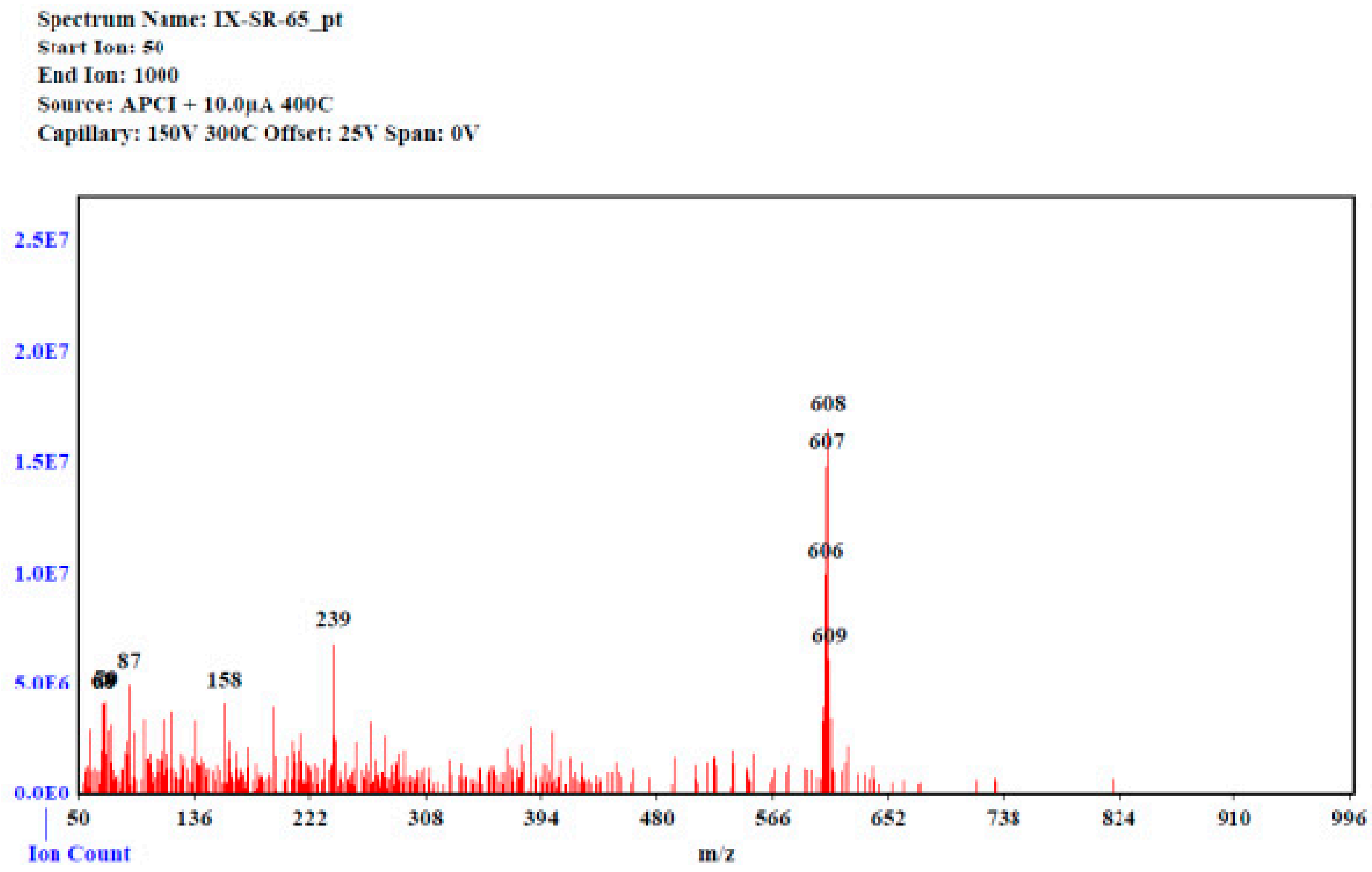

Figure S184. MS spectrum of **43**.

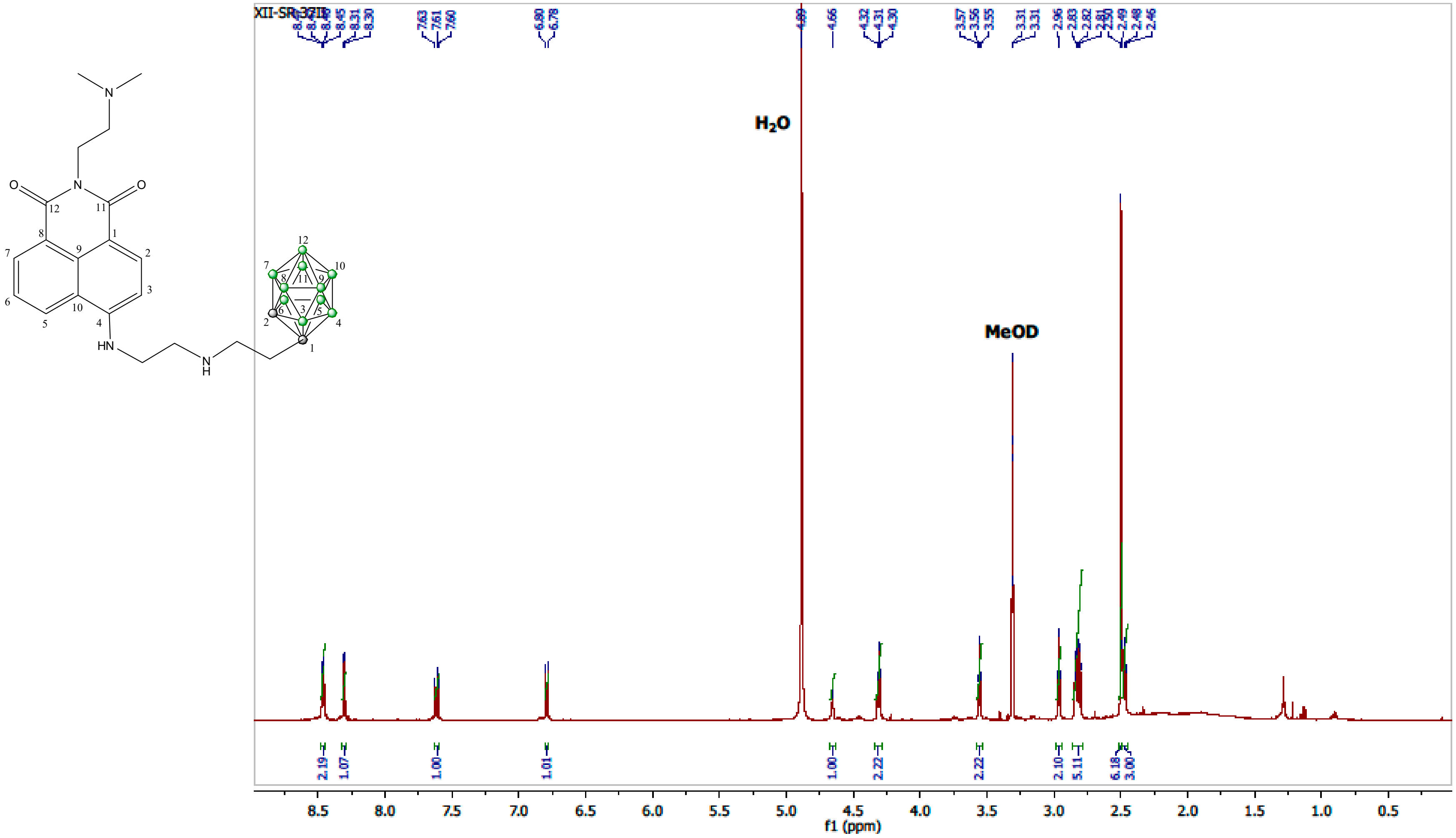

Figure S185. ^{1}H NMR spectrum of **54**.

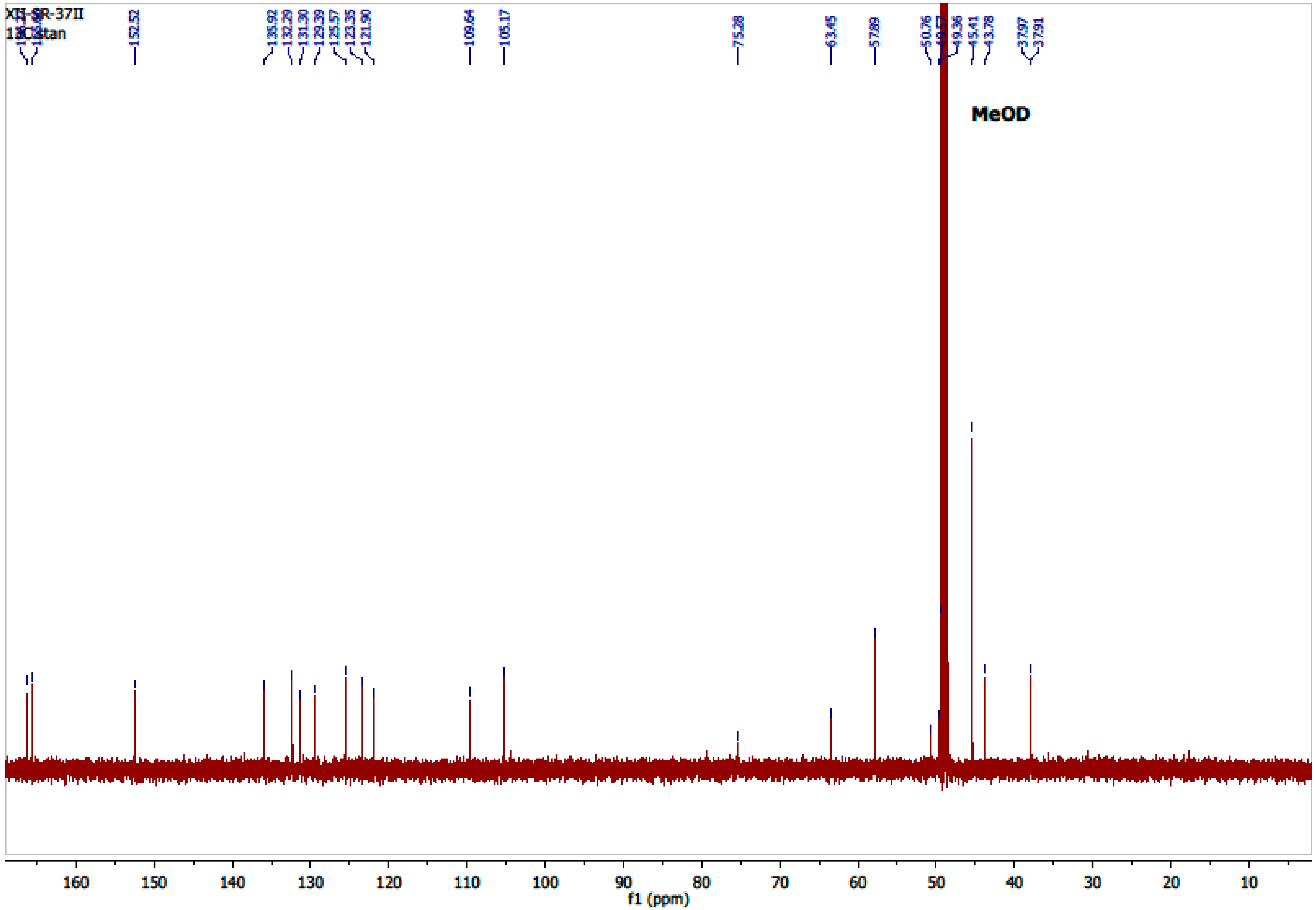

Figure S186. ^{13}C NMR spectrum of **54**.

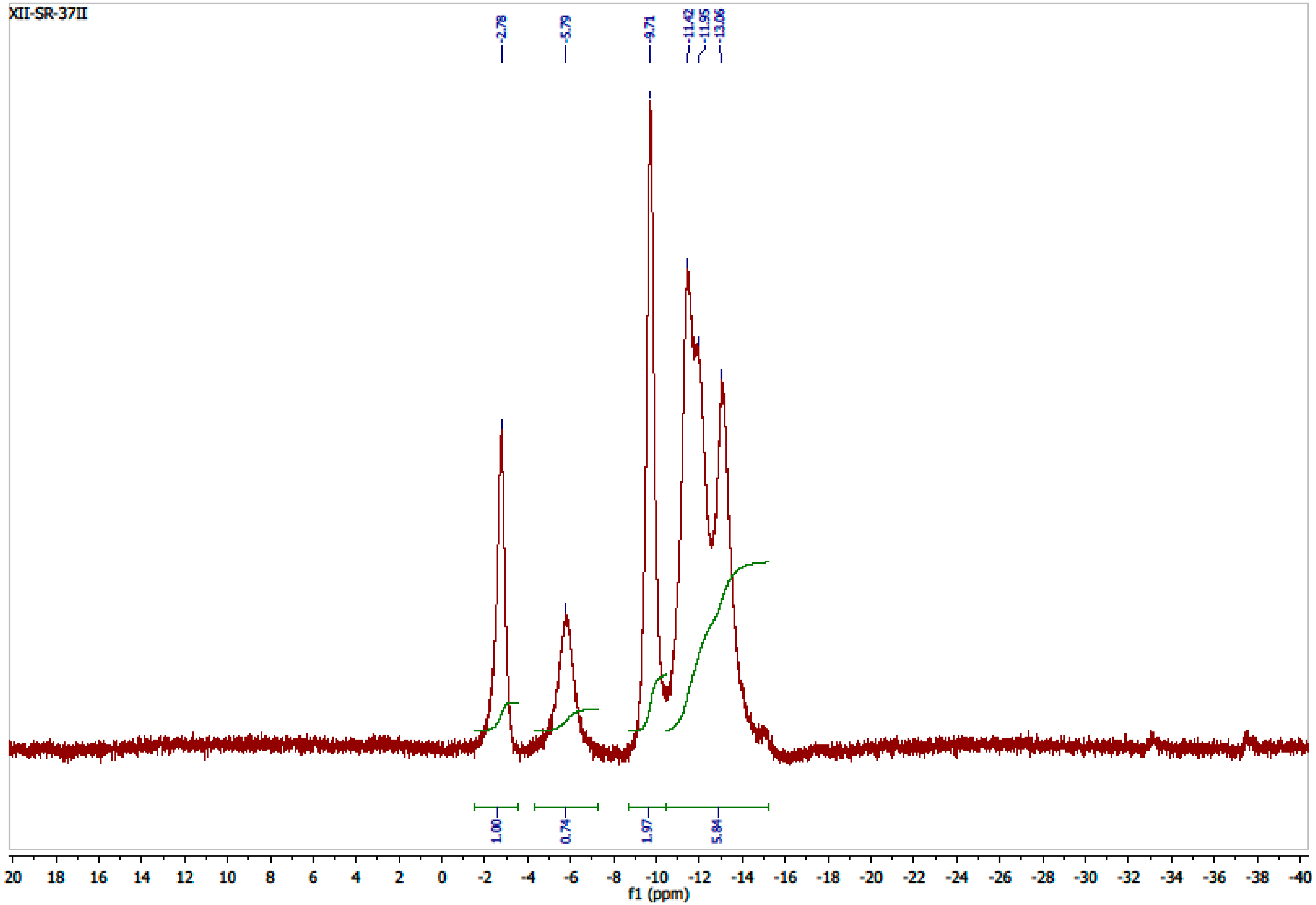

Figure S187. ^{11}B NMR spectrum of **54**.

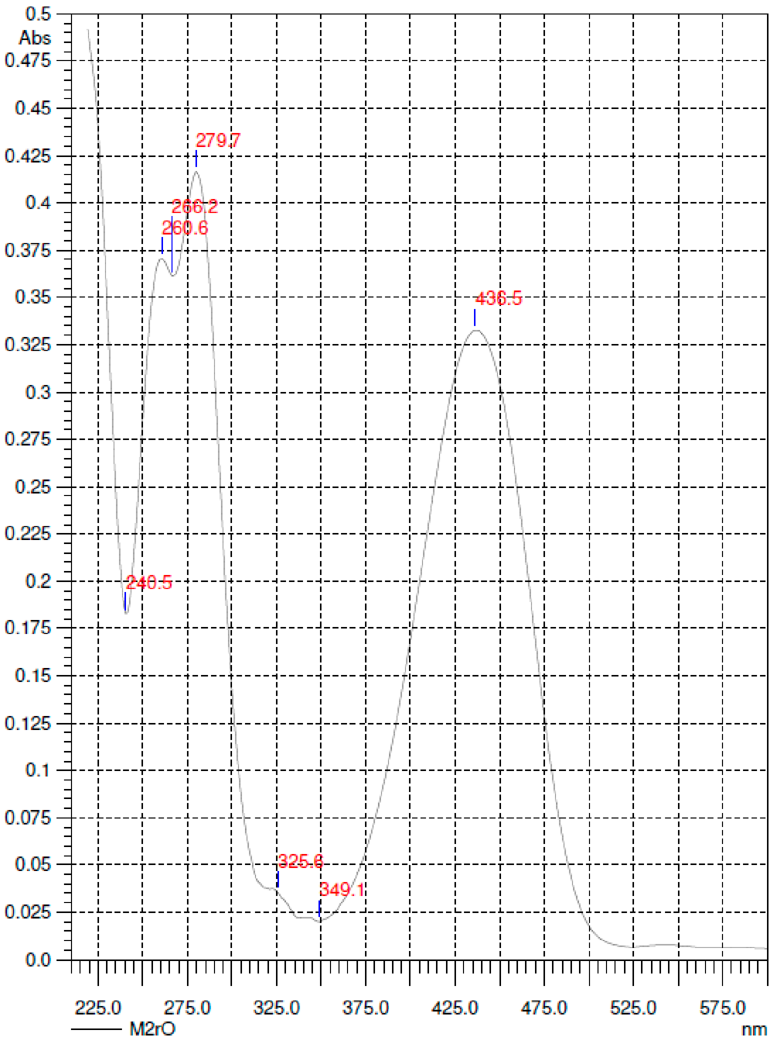

Figure S188. UV spectrum of **54.**

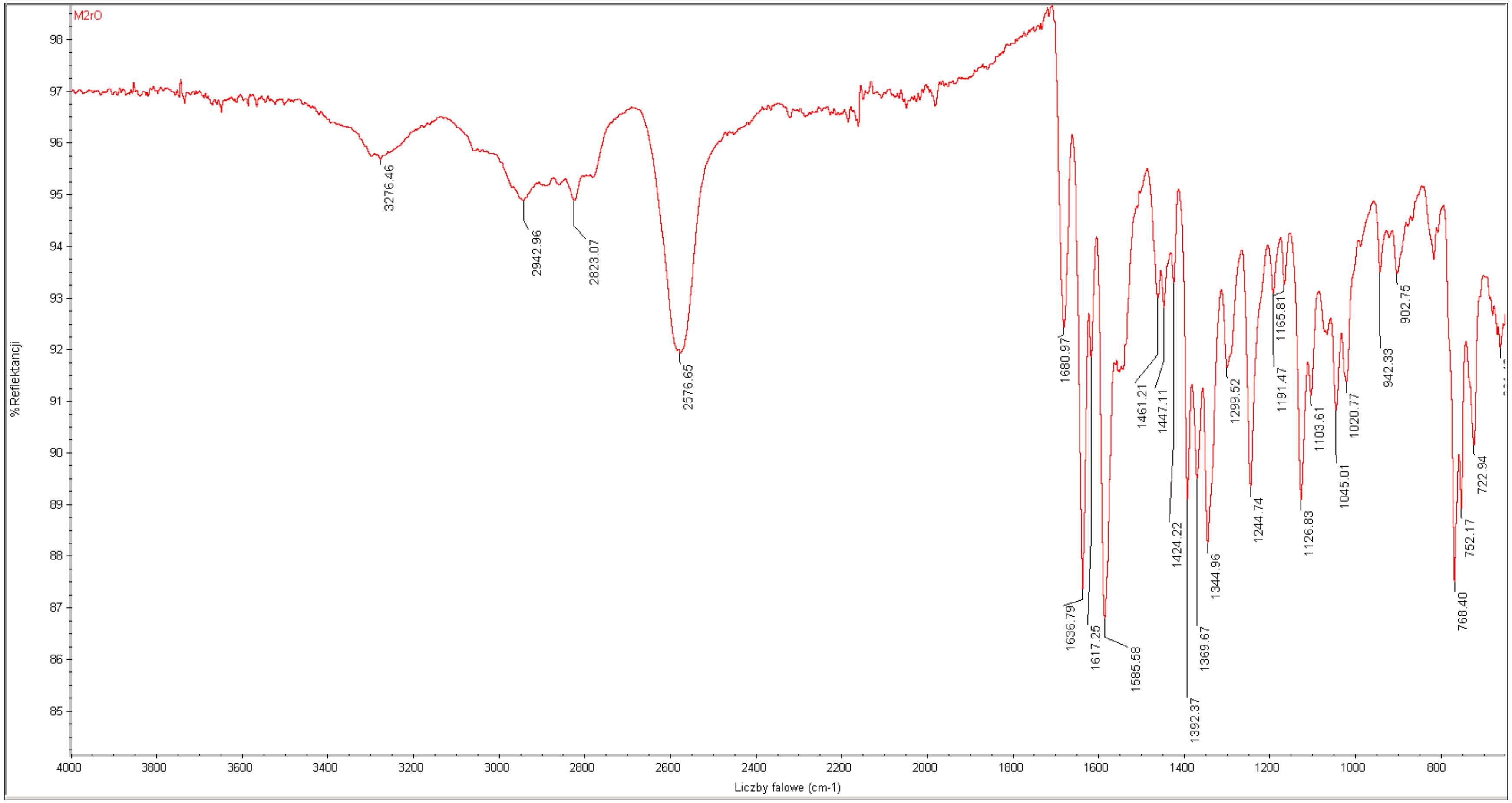

Figure S189. IR spectrum of **54**.

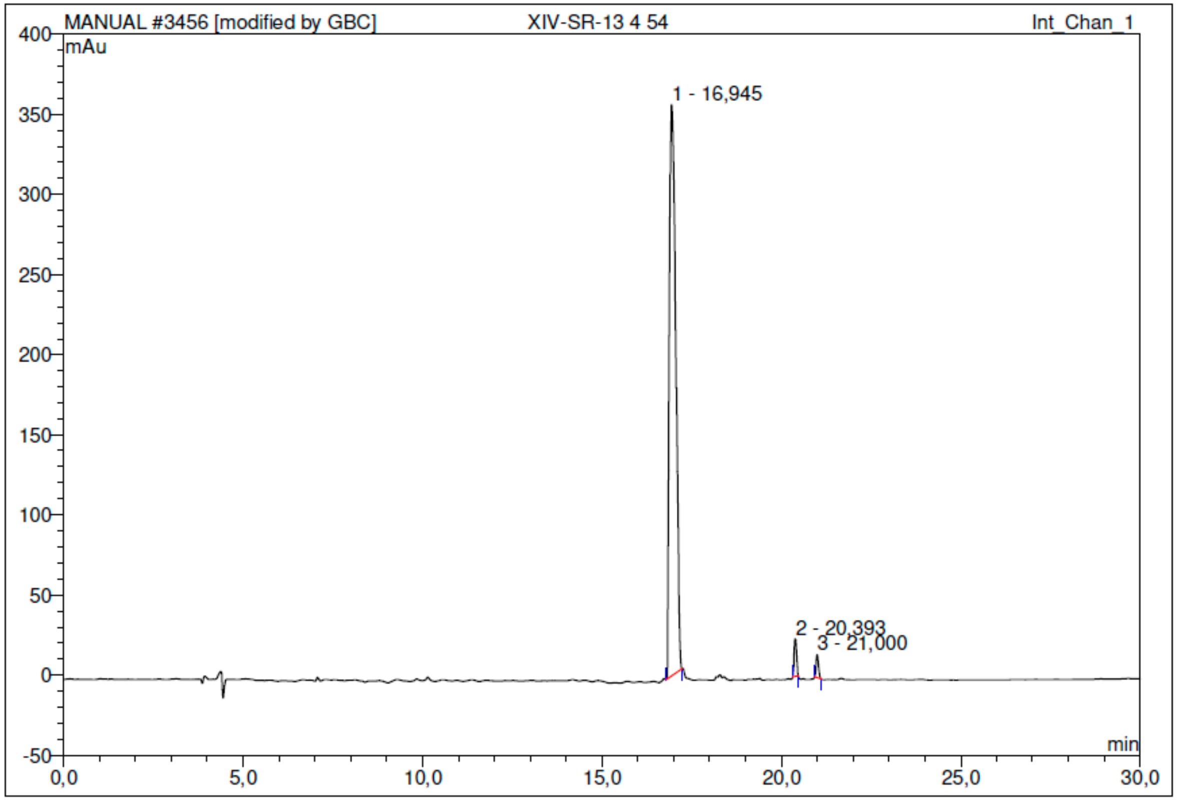

No.	Ret.Time min	Peak Name	Height mAu	Area mAu*min	Rel.Area %	Amount	Type
1	16,94	n.a.	356,425	73,272	96,18	n.a.	BMB
2	20,39	n.a.	23,359	1,749	2,30	n.a.	BMB*
3	21,00	n.a.	14,232	1,158	1,52	n.a.	BMB*
Total:			394,016	76,180	100,00	0,000	

Figure S190. HPLC chromatogram of 1**6**.

Spectrum Name: XII-SR-37_2kol_pt1
Start Ion: 350
End Ion: 700
Source: APCI + 10.0µA 400C
Capillary: 150V 300C Offset: 25V Span: 0V

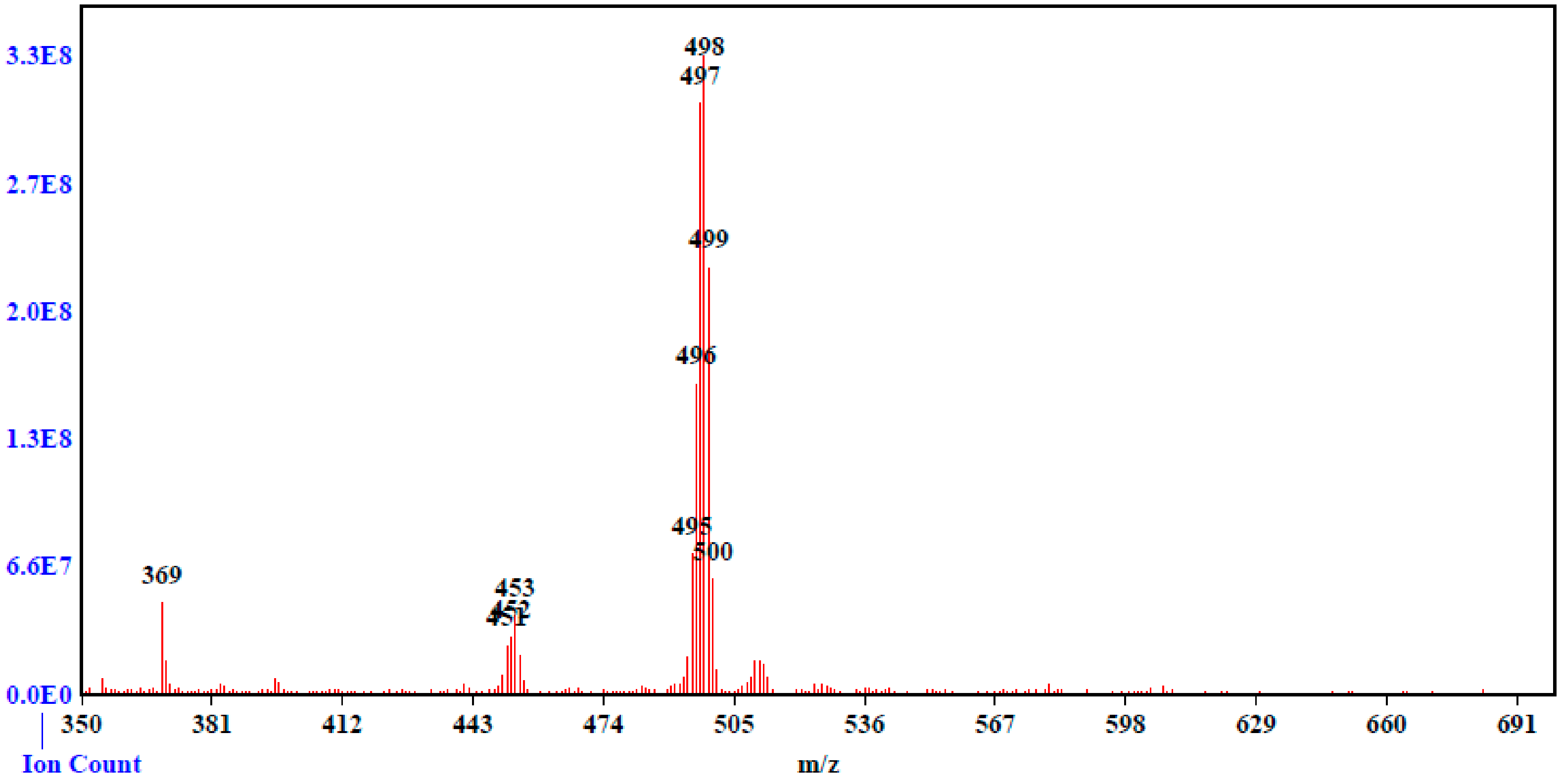

Figure S191. MS spectrum of **54**.

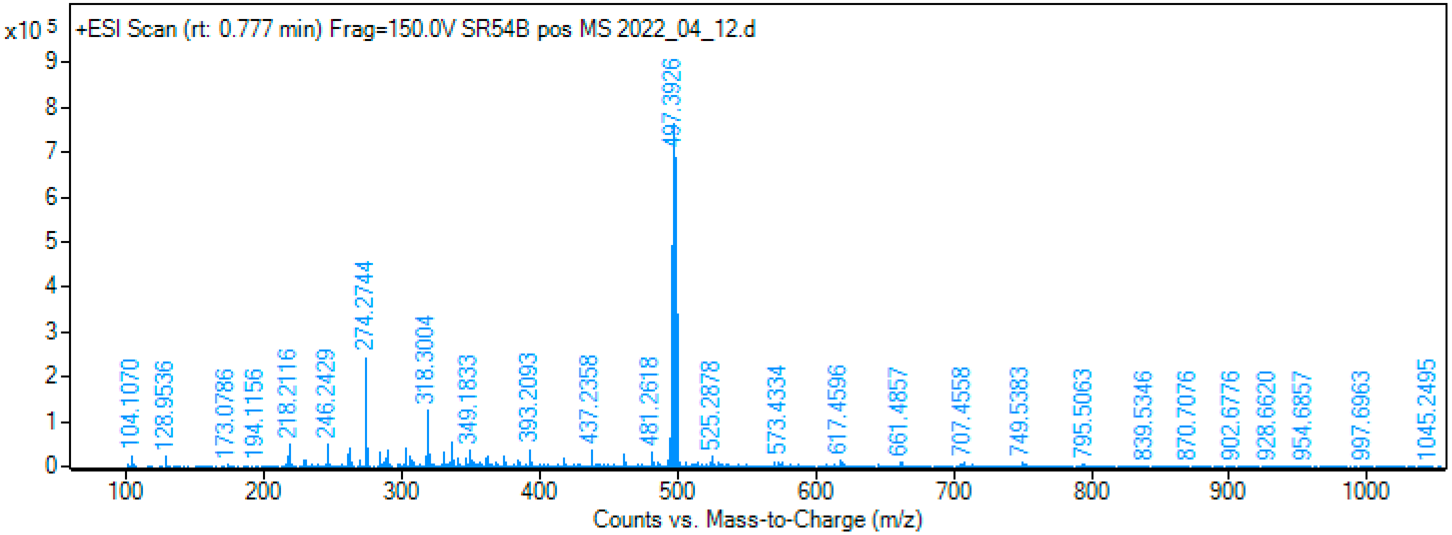

Figure S192. HRMS spectrum of **54**.

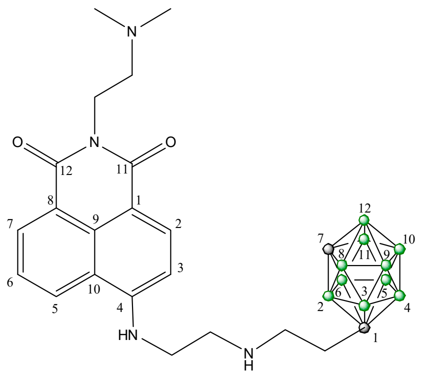

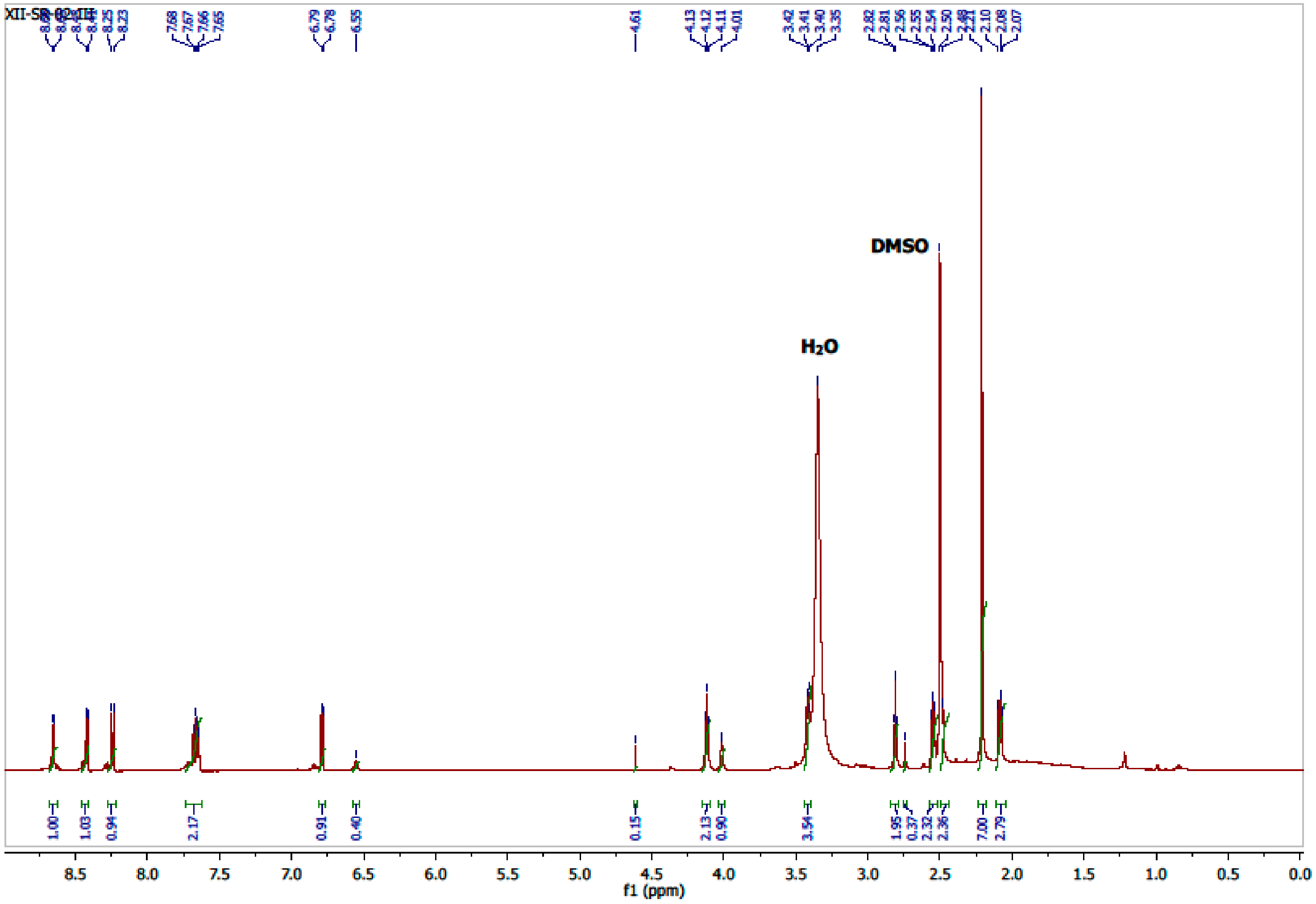

Figure S193. ^{1}H NMR spectrum of **55**.

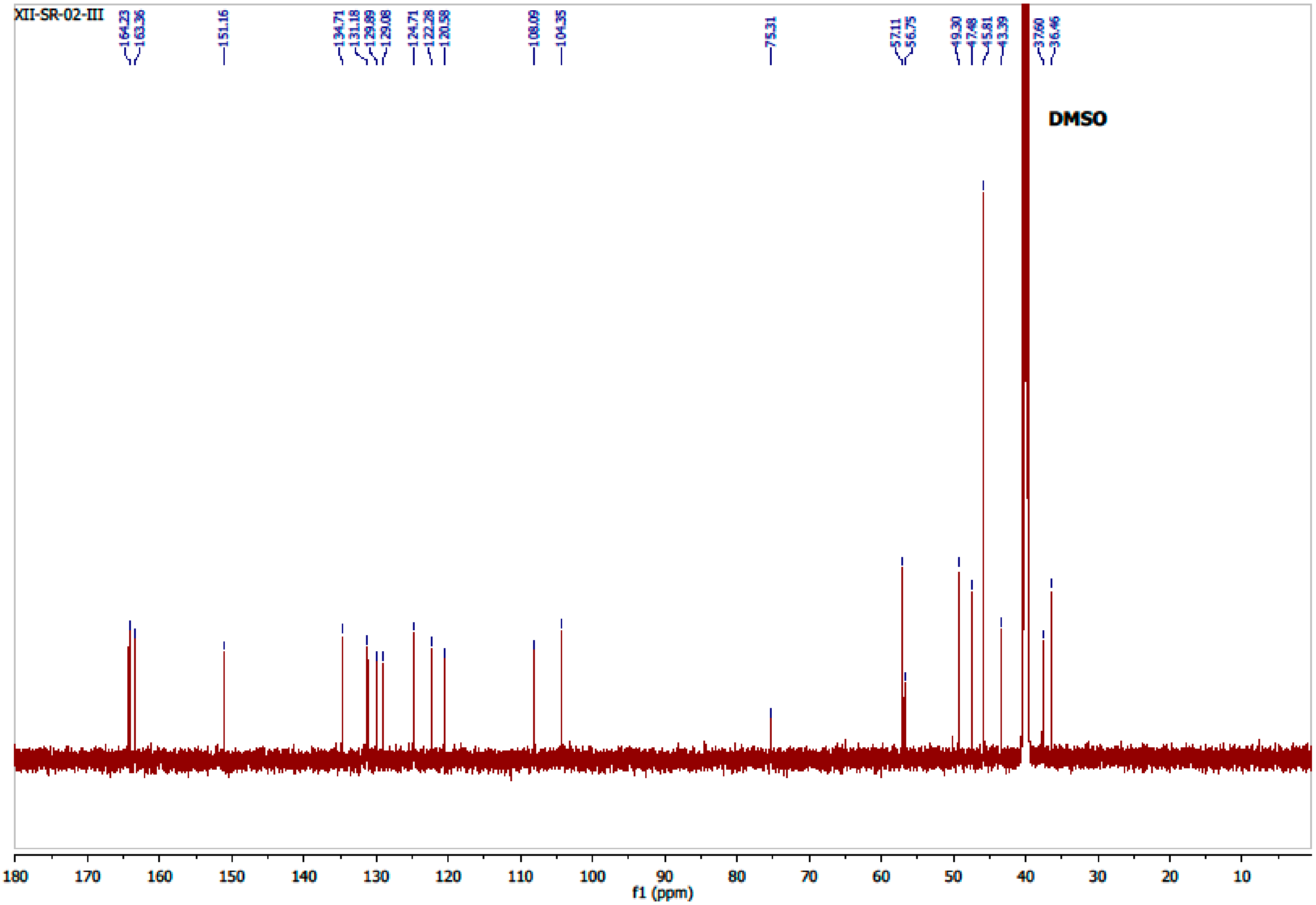

Figure S194. ^{13}C NMR spectrum of **55**.

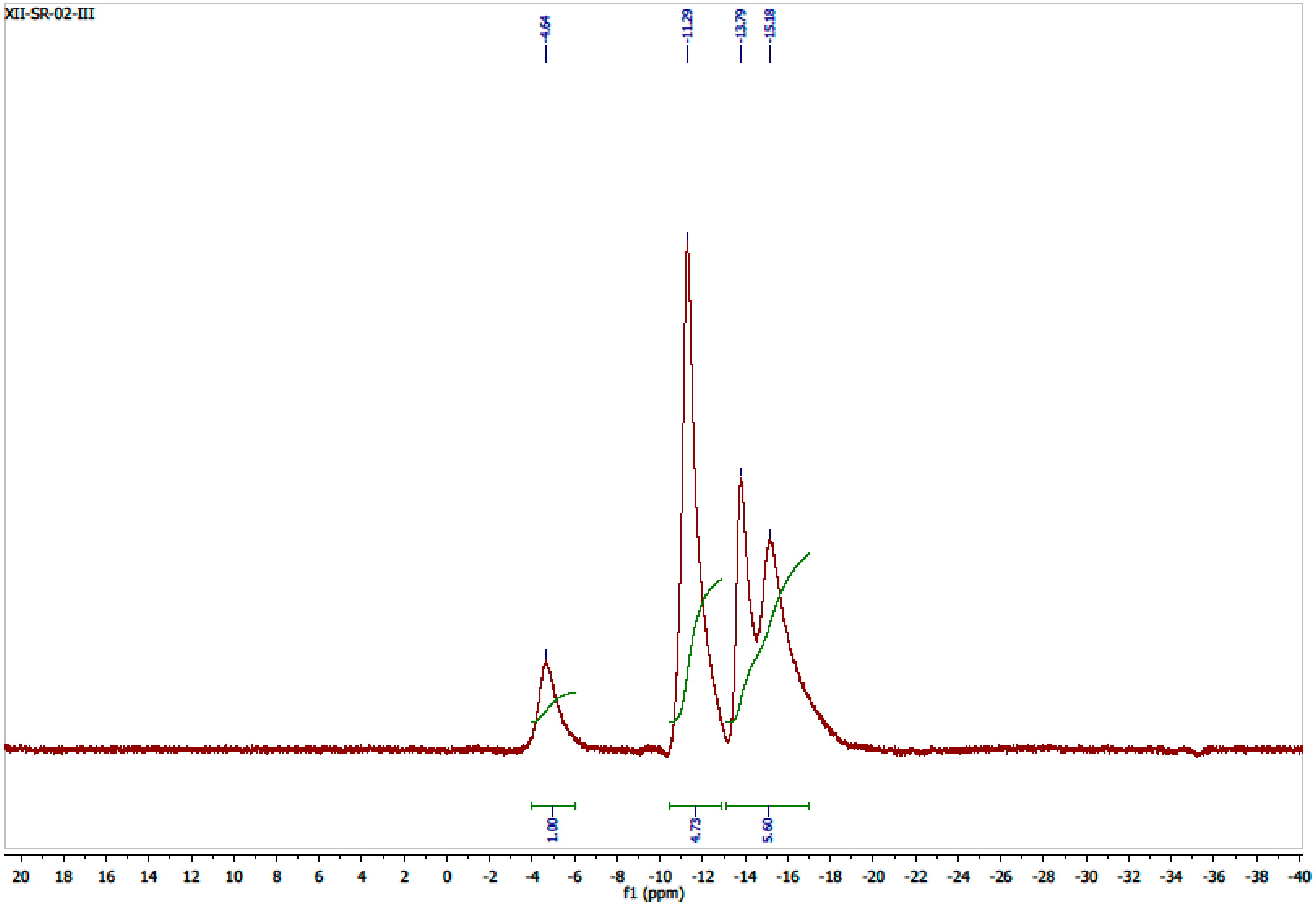

Figure S195. ^{11}B NMR spectrum of **55**.

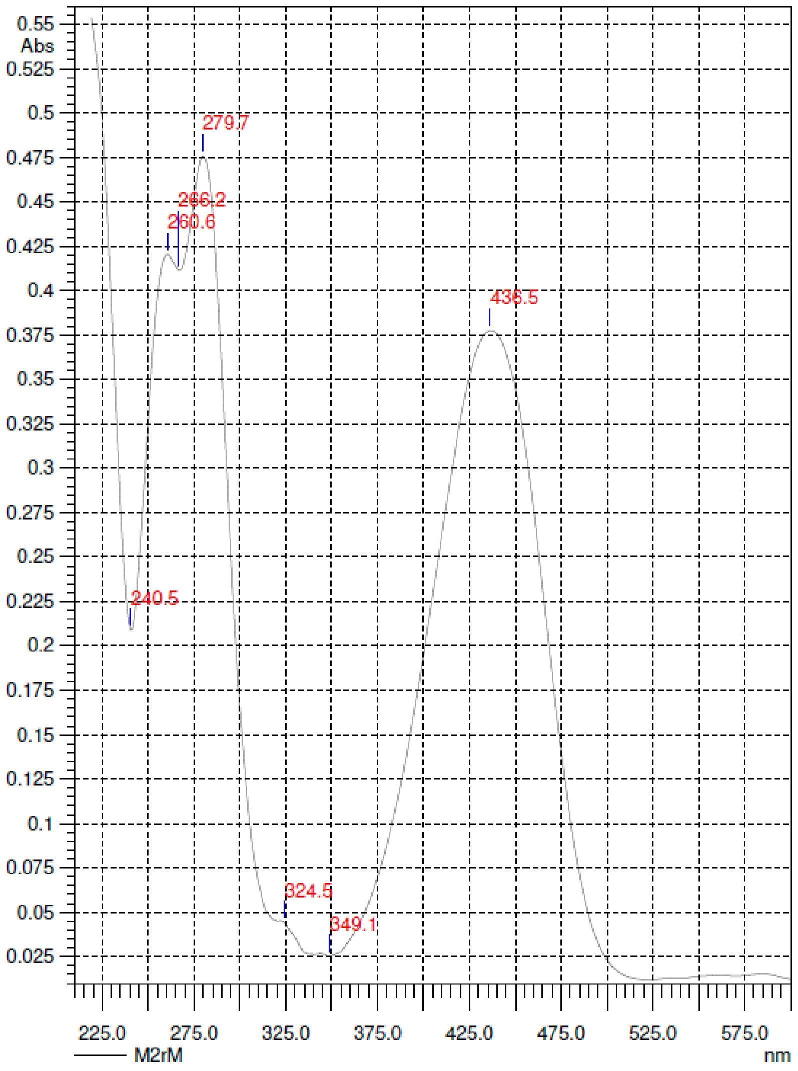

Figure S196. UV spectrum of **55**.

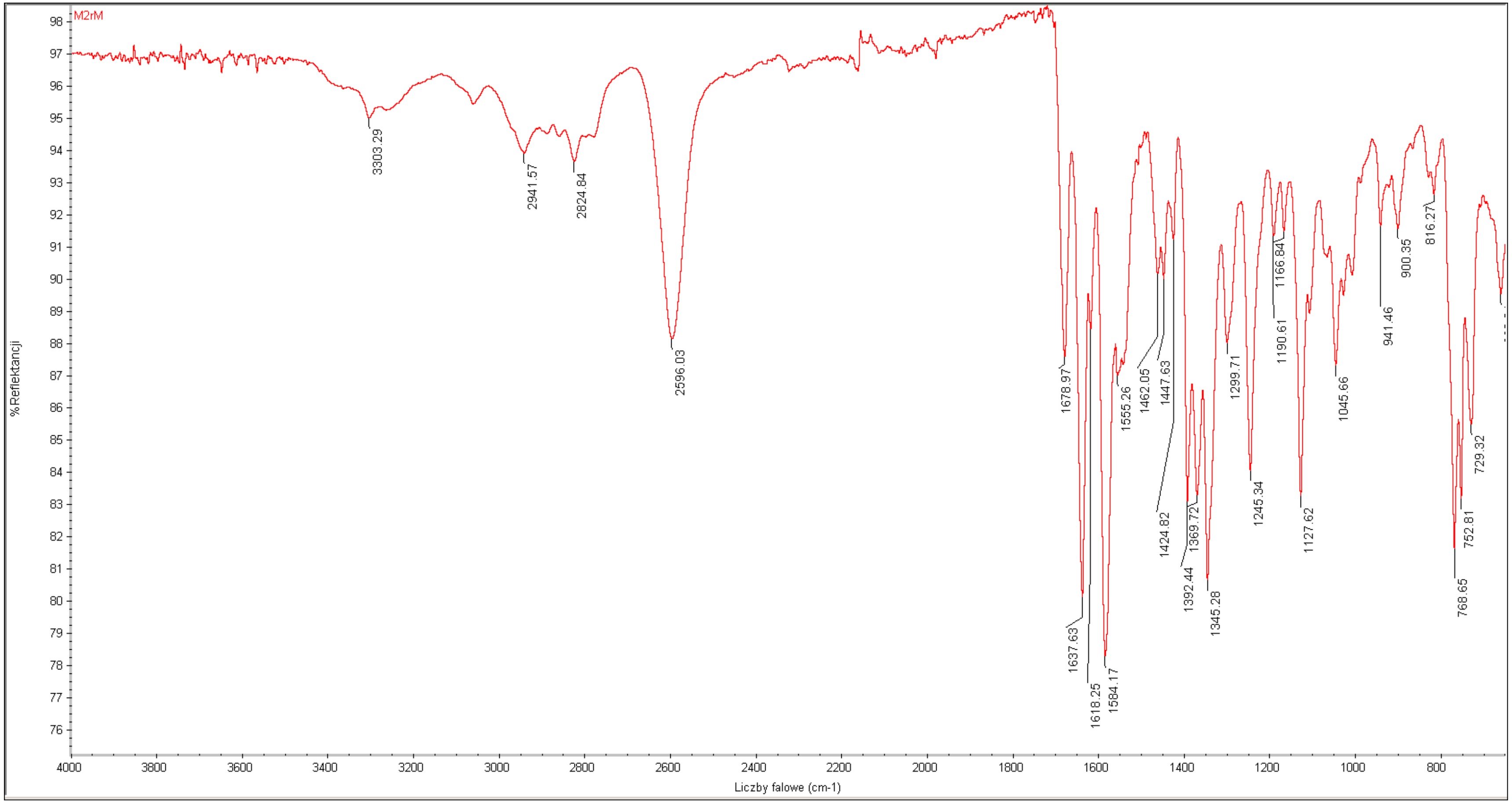

Figure S197. IR spectrum of **55**.

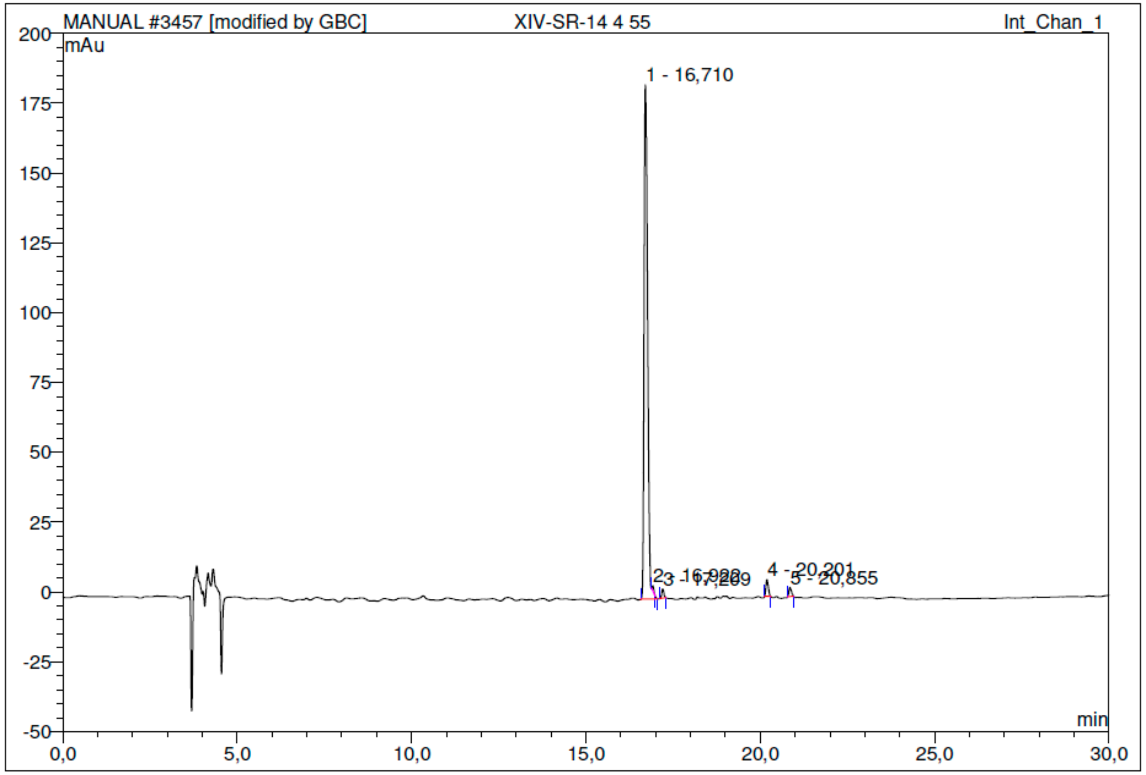

No.	Ret.Time min	Peak Name	Height mAu	Area mAu*min	Rel.Area %	Amount	Type
1	16,71	n.a.	184,076	20,768	95,08	n.a.	BMB*
2	16,92	n.a.	1,822	0,094	0,43	n.a.	Rd*
3	17,21	n.a.	3,239	0,239	1,10	n.a.	BMB*
4	20,20	n.a.	5,973	0,460	2,10	n.a.	BMB*
5	20,85	n.a.	3,186	0,282	1,29	n.a.	BMB*
**Total:**			198,296	21,843	100,00	0,000	

Figure S198. HPLC chromatogram of **55**.

Spectrum Name: XII-SR-02_MC2M_pt
Start Ion: 300
End Ion: 700
Source: APCI + 10.0µA 400C
Capillary: 150V 300C Offset: 25V Span: 0V

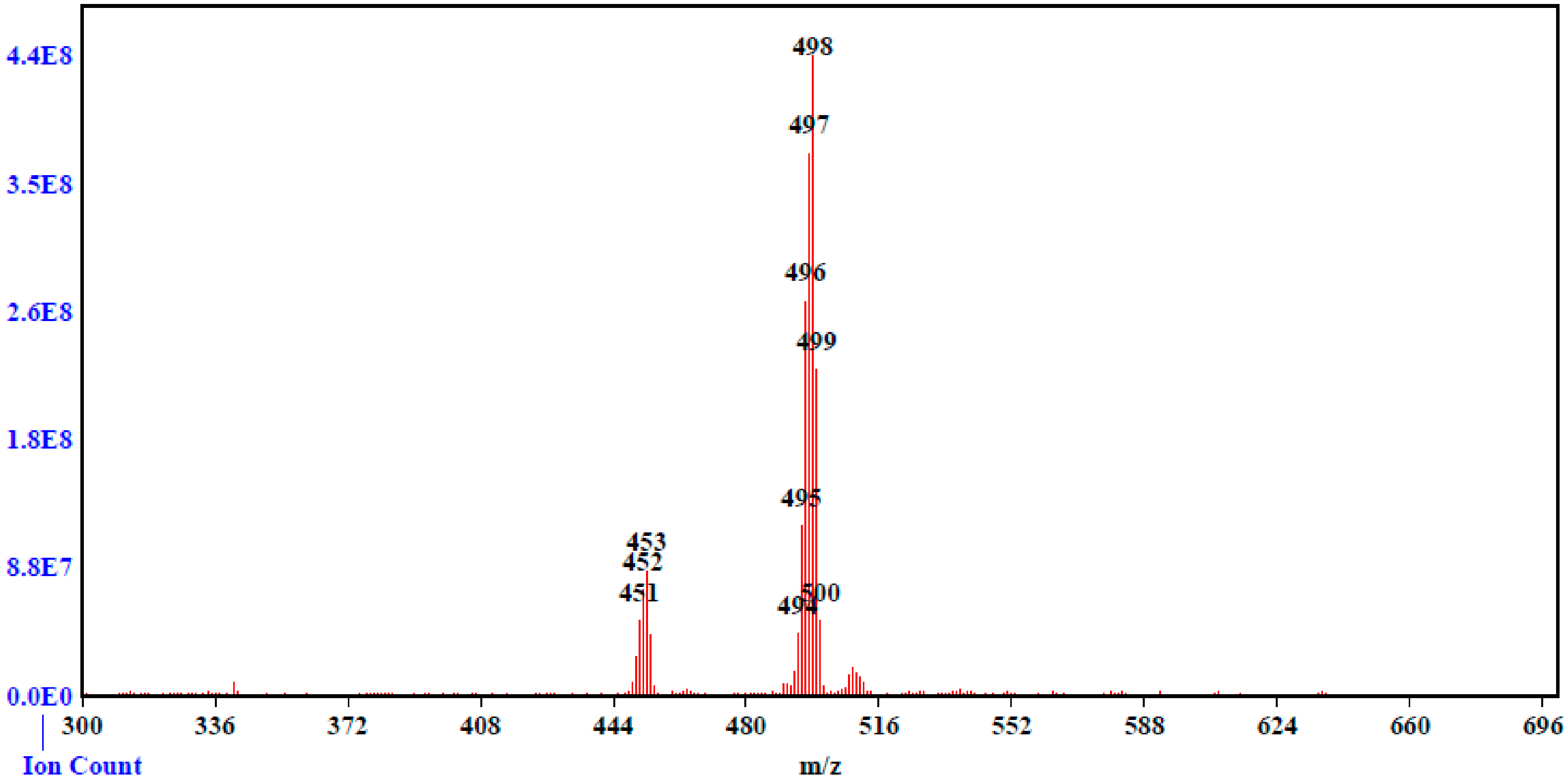

Figure S199. MS spectrum of **55**.

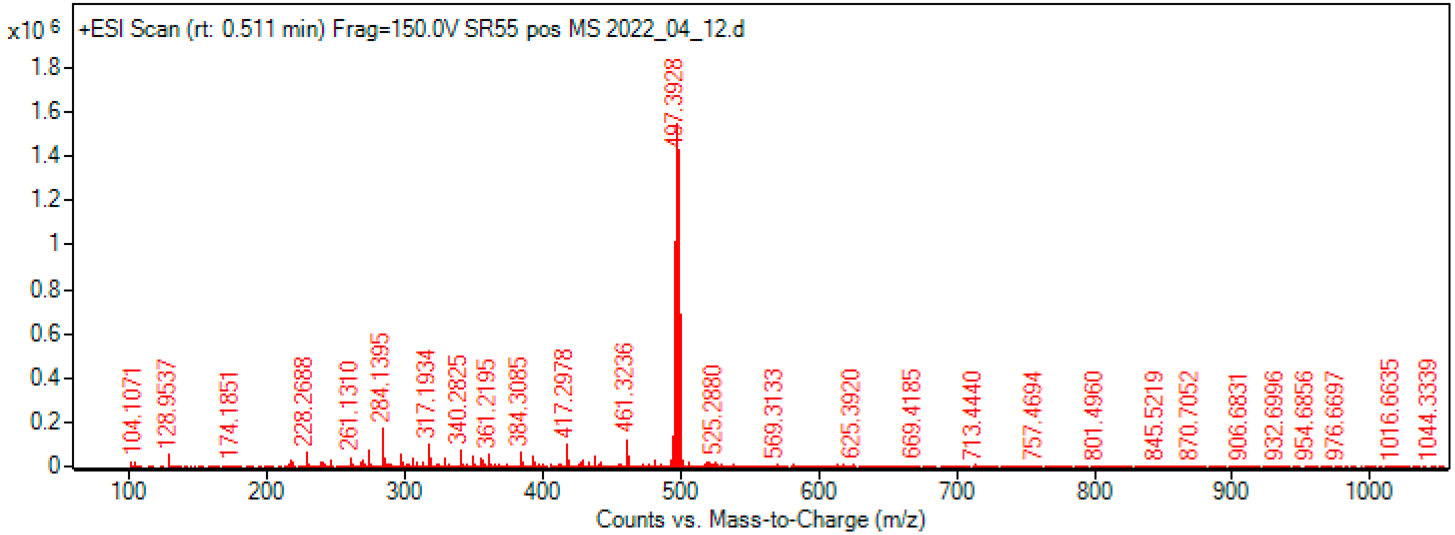

Figure S200. HRMS spectrum of **55**.

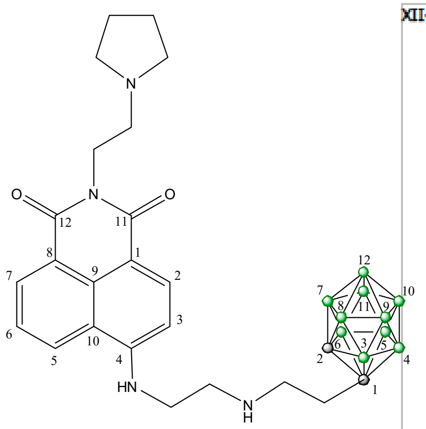

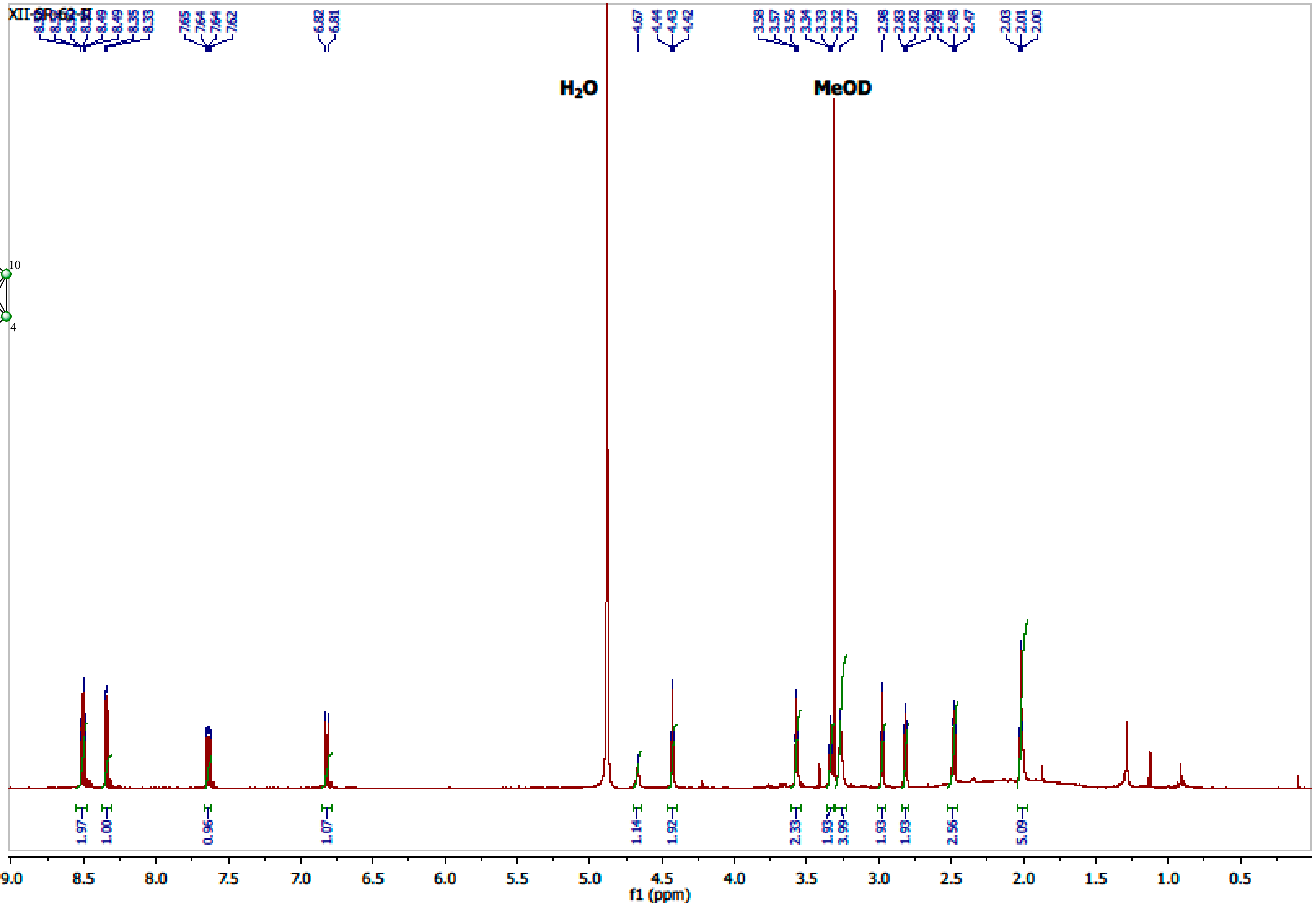

Figure S201. ^{1}H NMR spectrum of **56**.

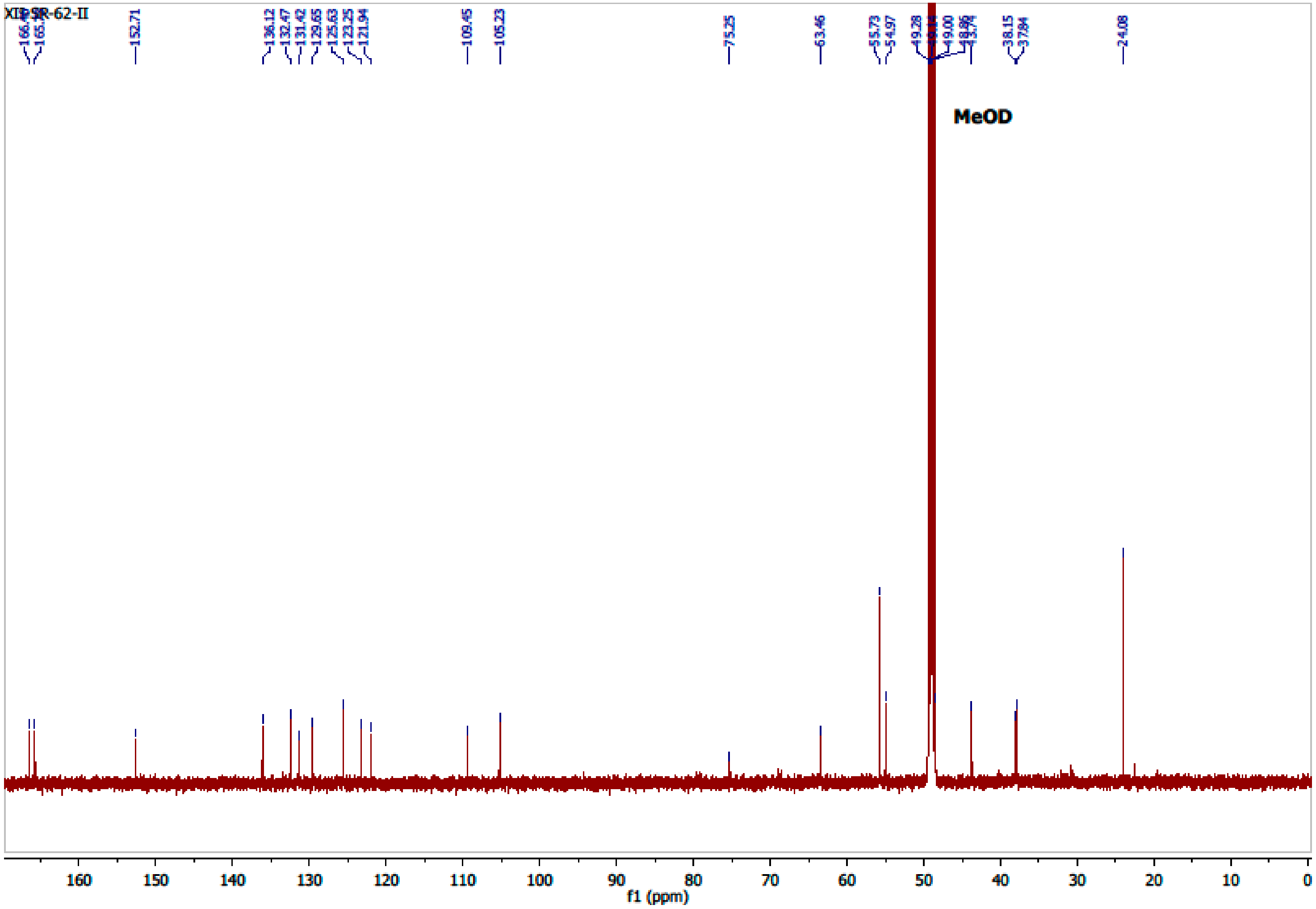

Figure S202. ^{13}C NMR spectrum of **56**.

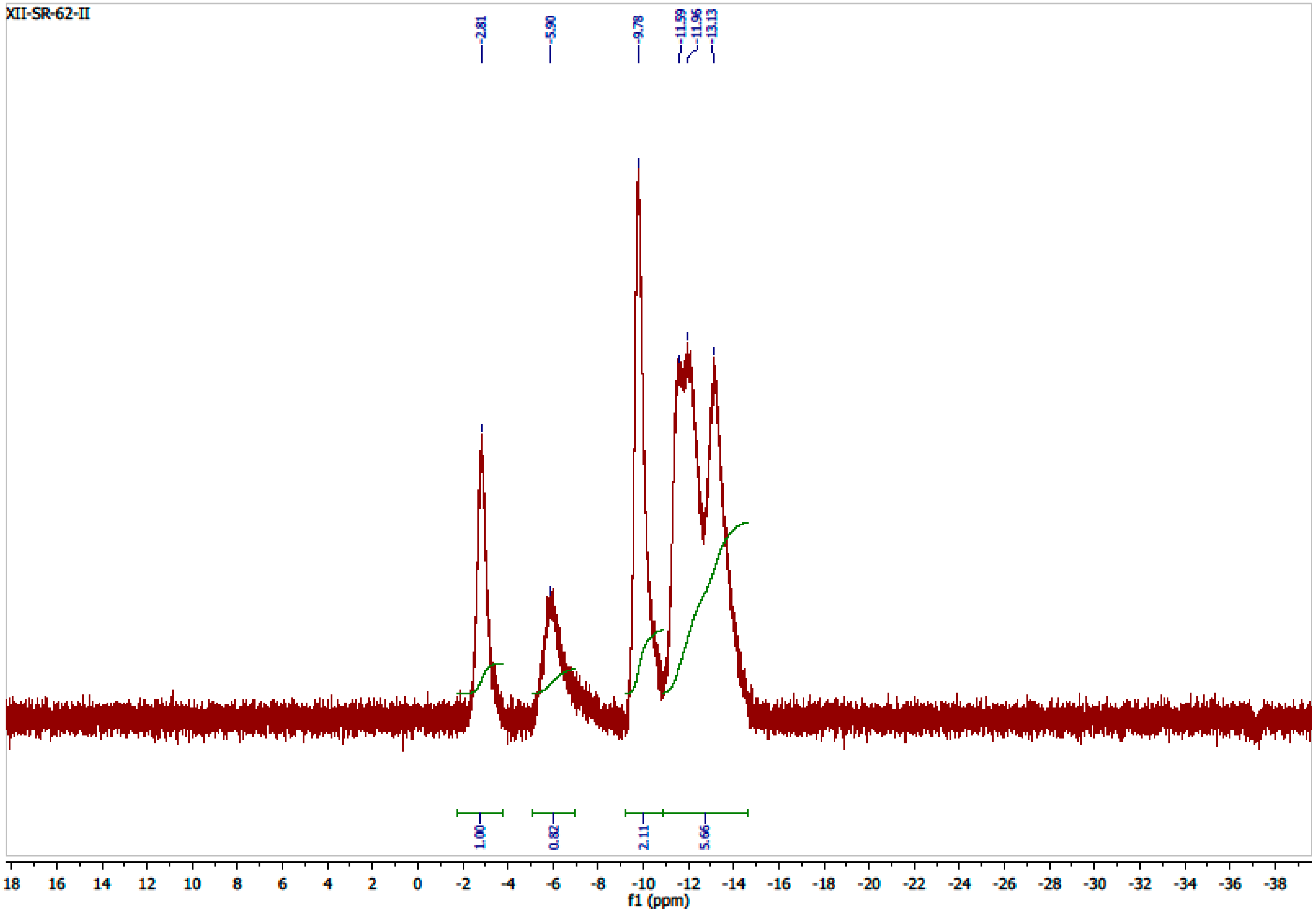

Figure S203. ^{11}B NMR spectrum of **56**.

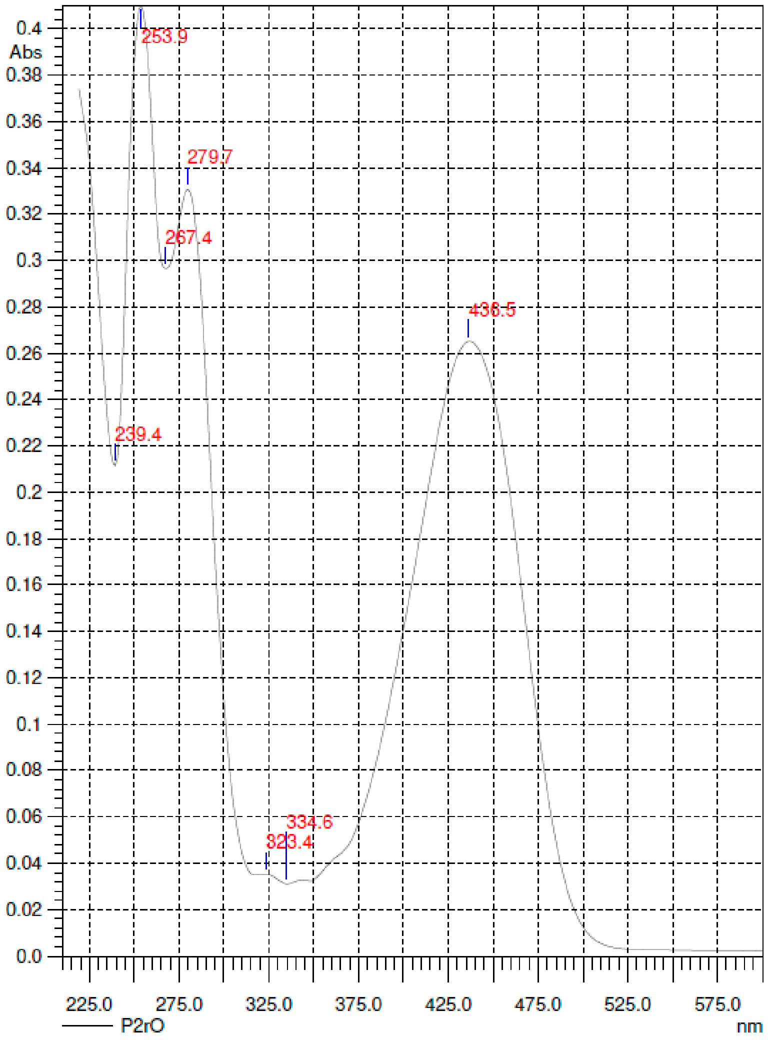

Figure S204. UV spectrum of **56**.

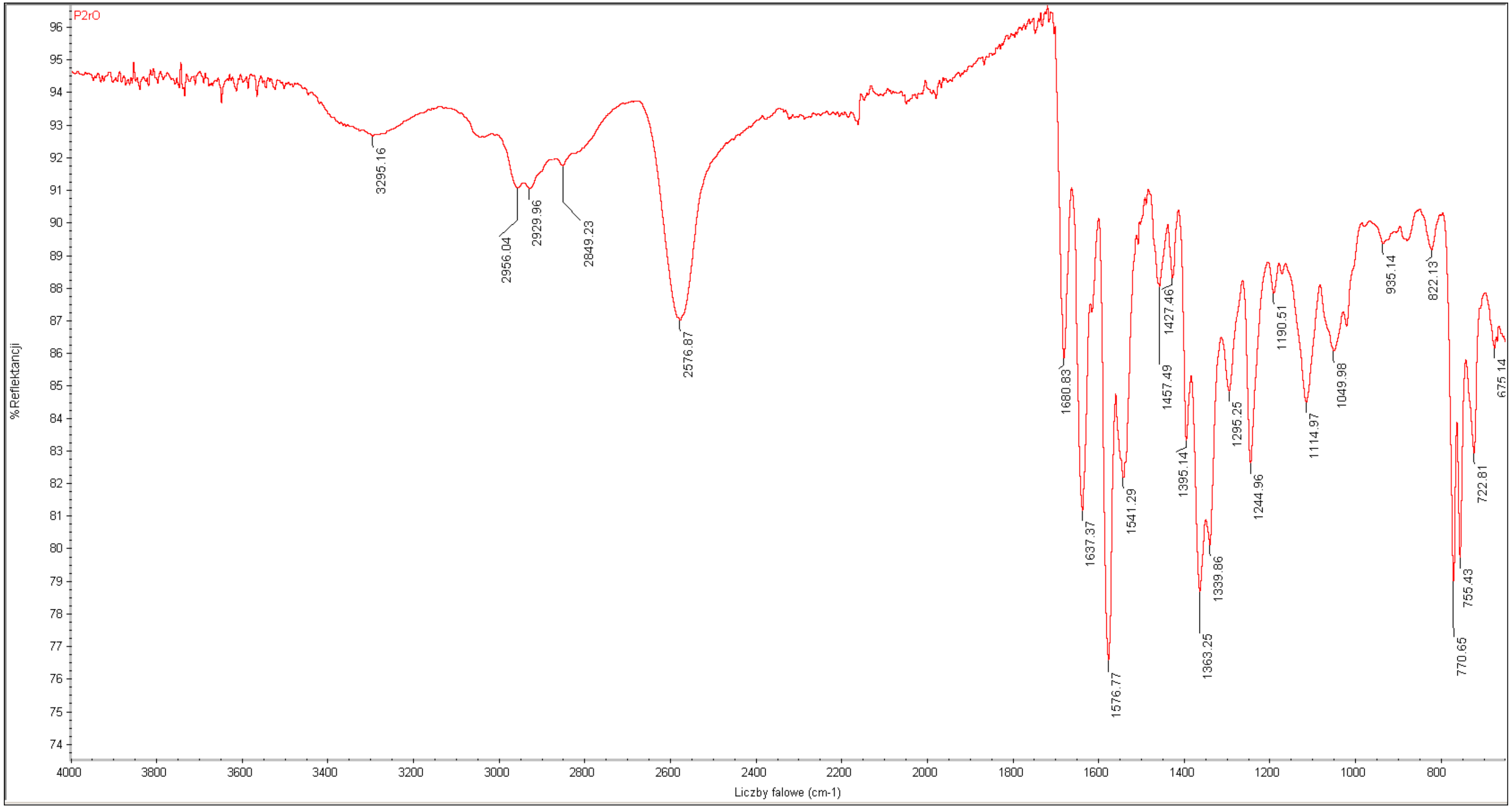

Figure S205. IR spectrum of **56**.

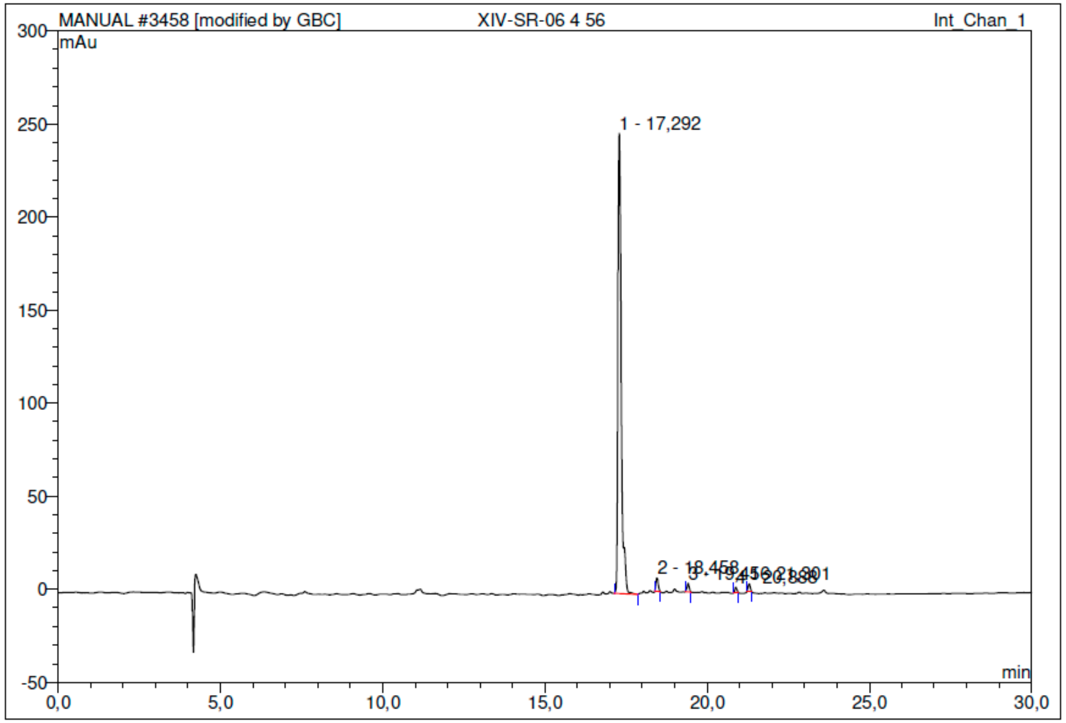

No.	Ret.Time min	Peak Name	Height mAu	Area mAu*min	Rel.Area %	Amount	Type
1	17,29	n.a.	247,303	29,369	95,29	n.a.	BMB
2	18,46	n.a.	7,209	0,565	1,83	n.a.	BMB*
3	19,42	n.a.	4,584	0,372	1,21	n.a.	BMB*
4	20,89	n.a.	2,863	0,198	0,64	n.a.	BMB*
5	21,30	n.a.	4,214	0,317	1,03	n.a.	BMB*
Total:			266,173	30,821	100,00	0,000	

Figure S206. HPLC chromatogram of **56**.

Spectrum Name: XII-SR-62_P2redO_pt
Start Ion: 200
End Ion: 700
Source: APCI + 10.0μA 400C
Capillary: 150V 300C Offset: 25V Span: 0V

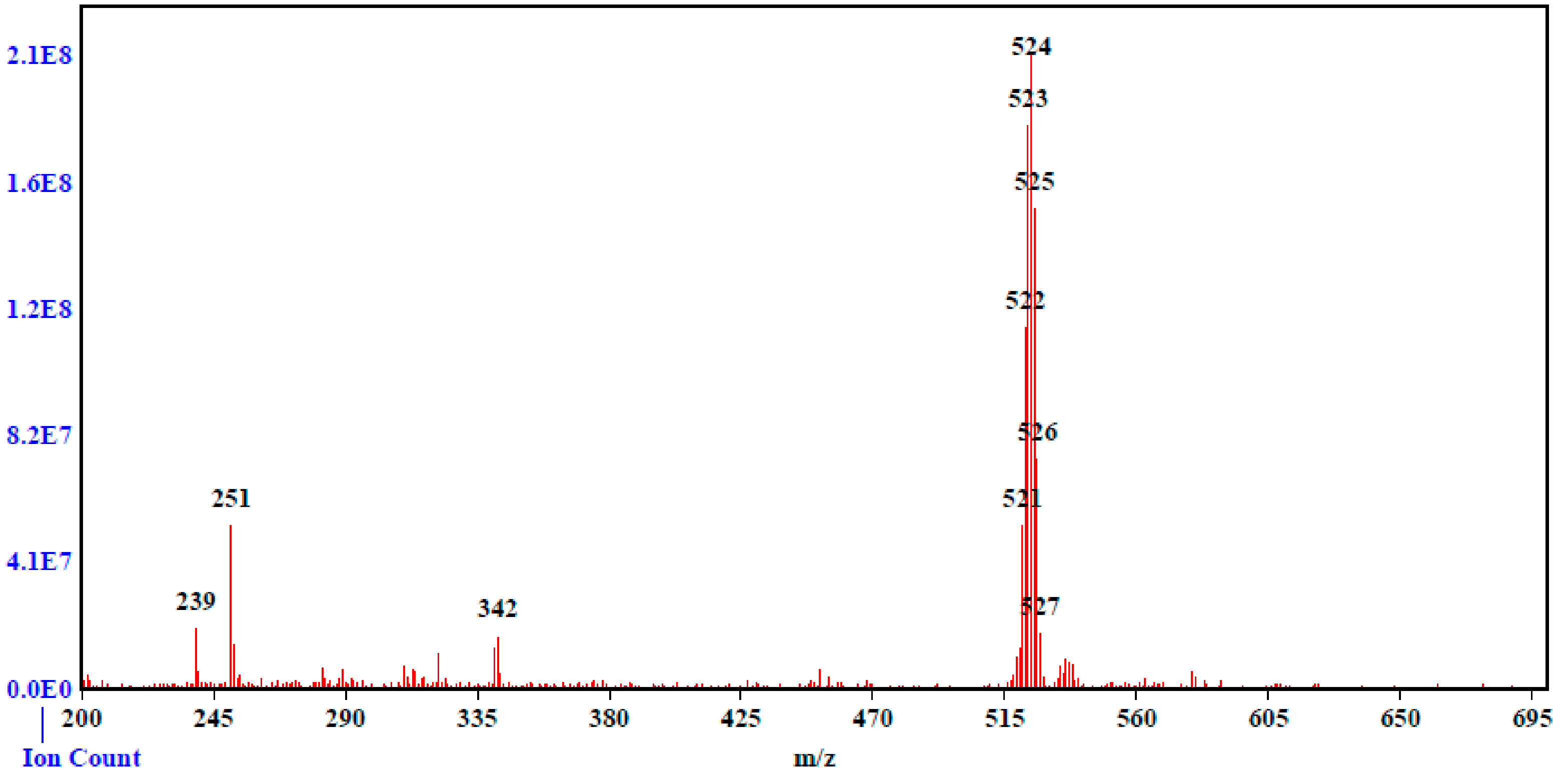

Figure S207. MS spectrum of **56**.

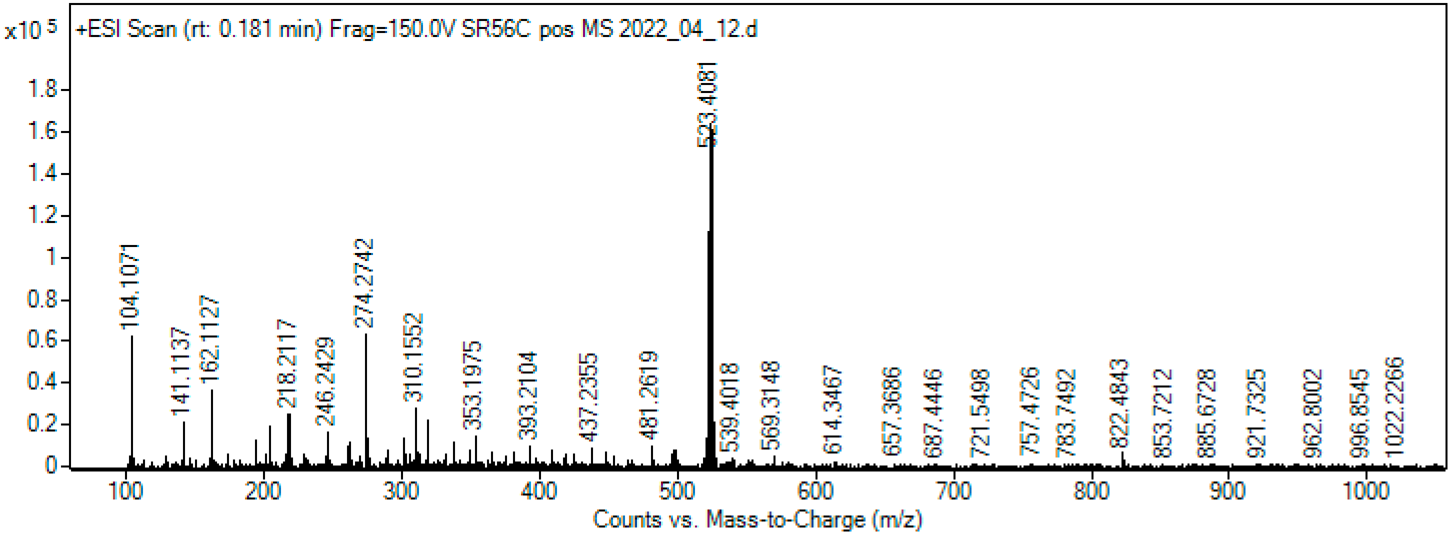

Figure S208. HRMS spectrum of **56**.

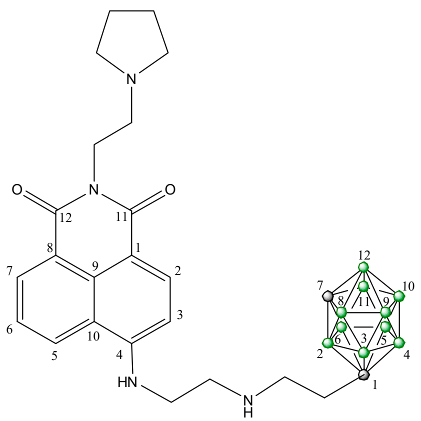

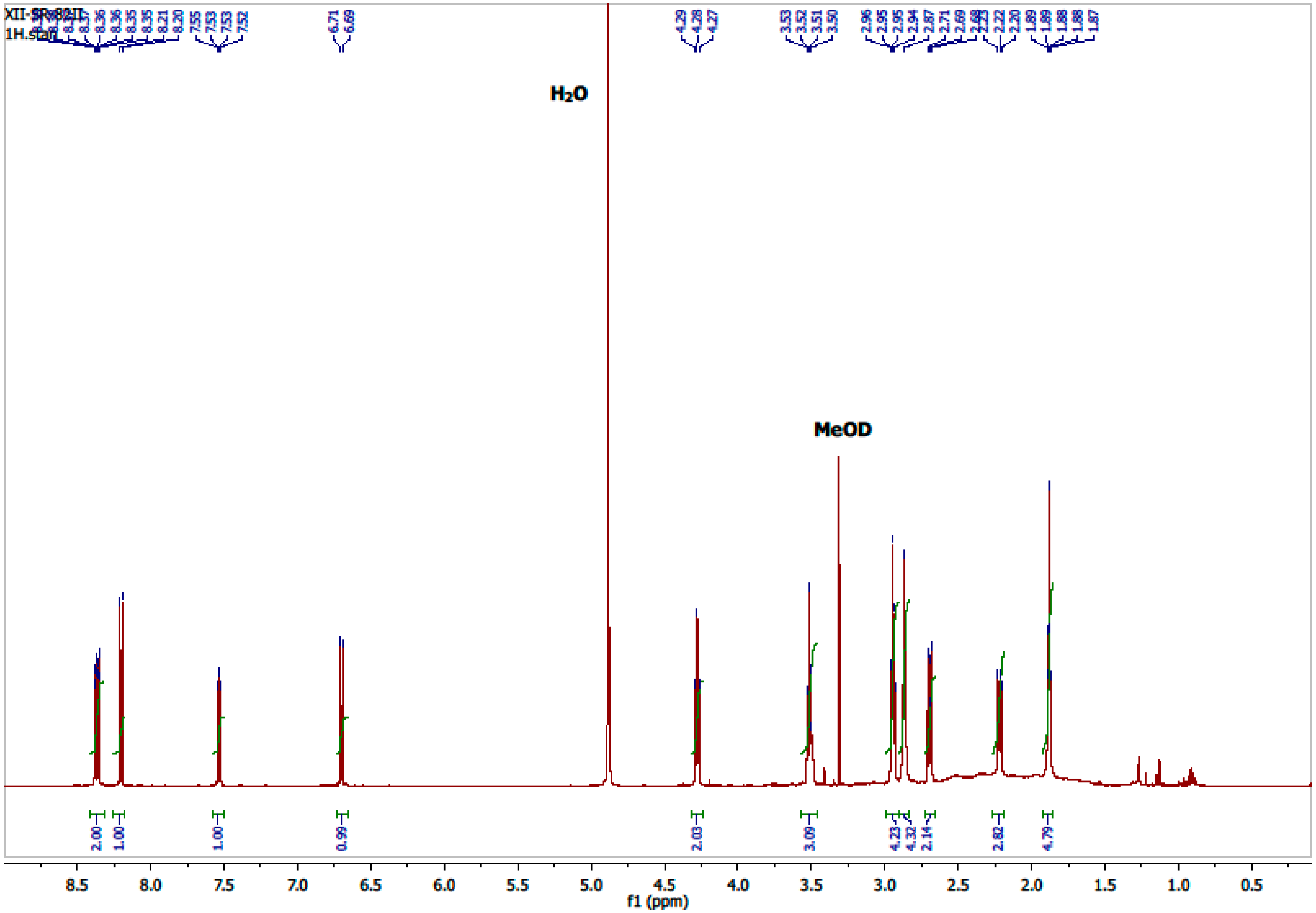

Figure S209. ^{1}H NMR spectrum of **57**.

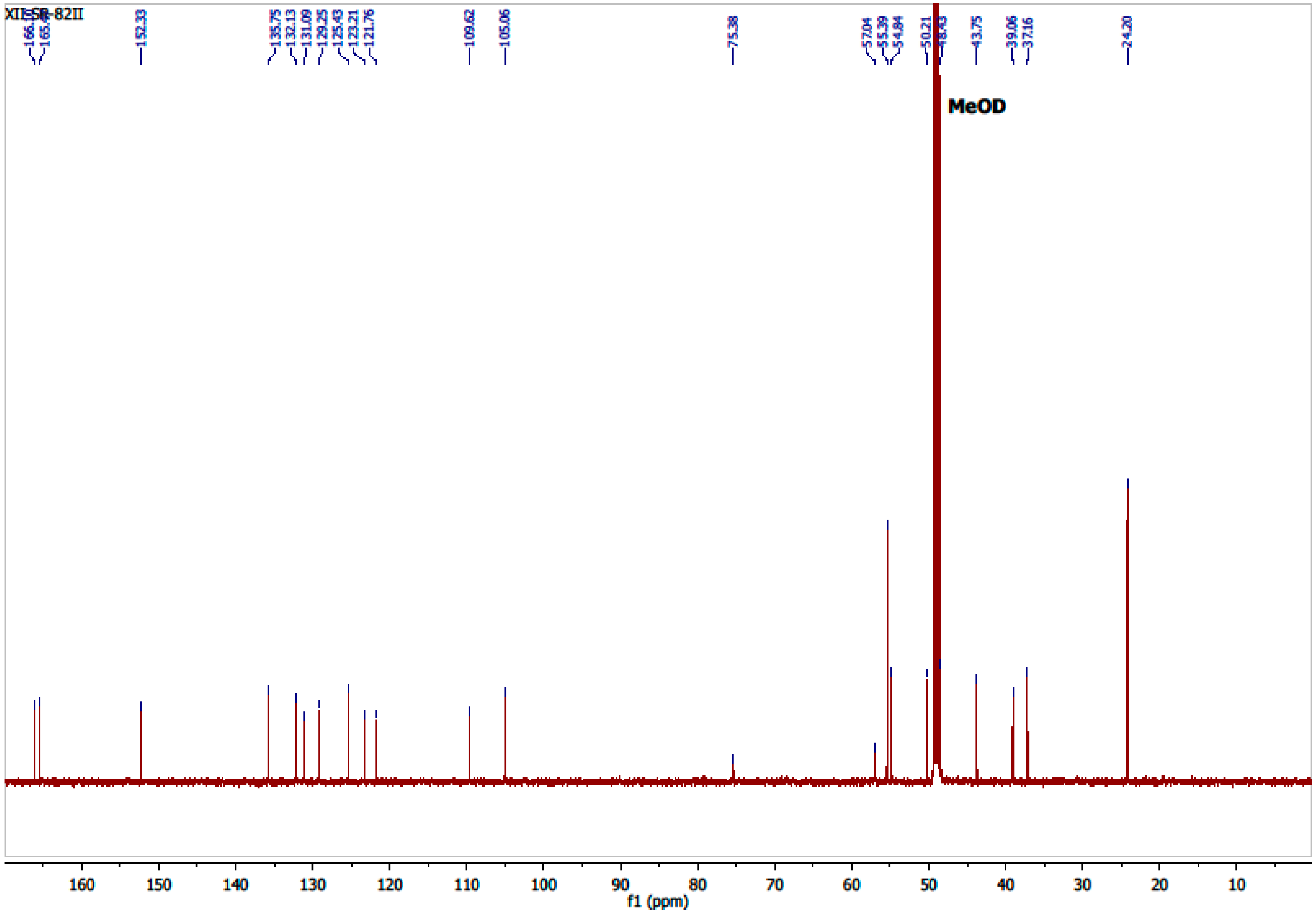

Figure S210. ^{13}C NMR spectrum of **57**.

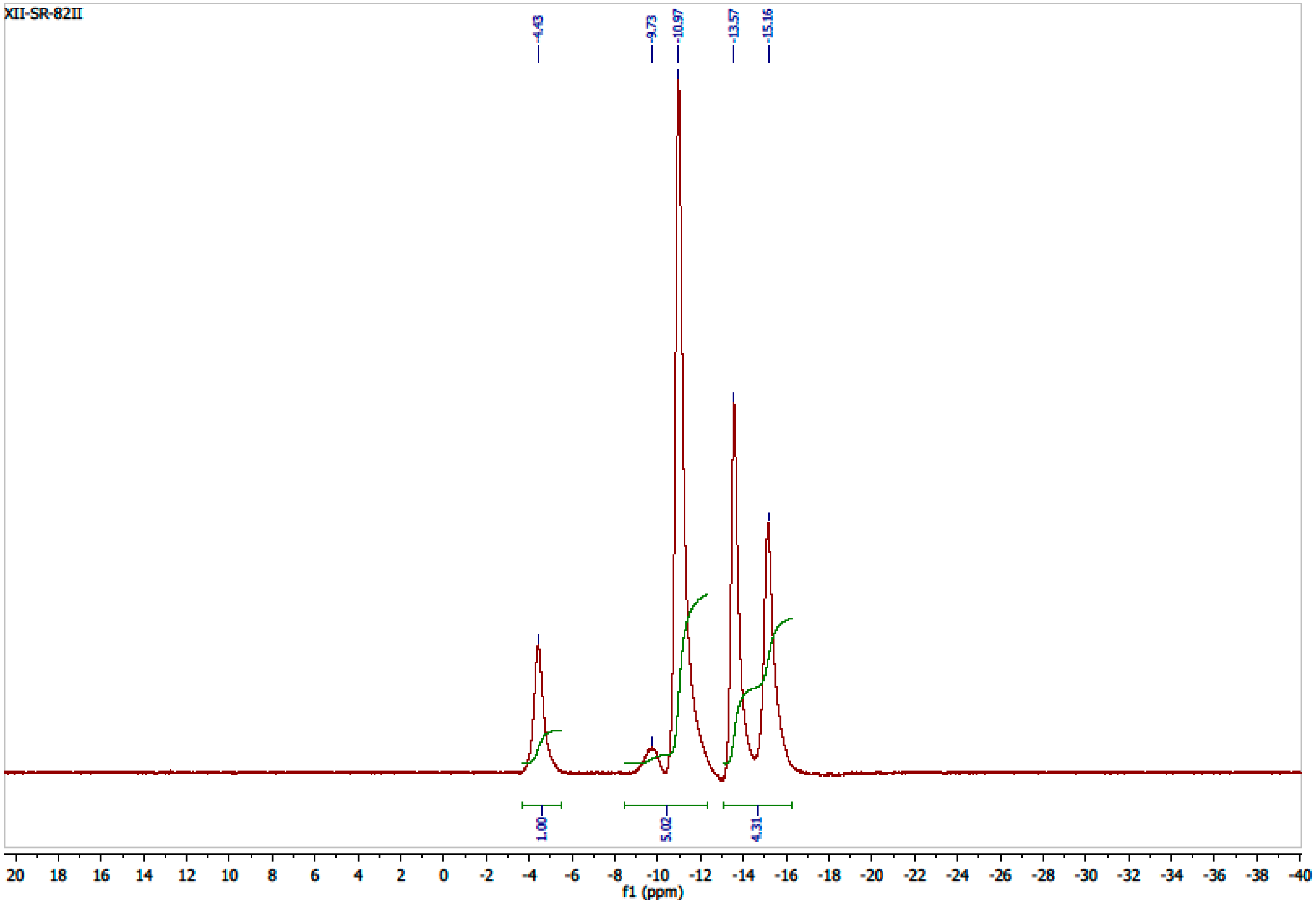

Figure S211. ^{11}B NMR spectrum of **57**.

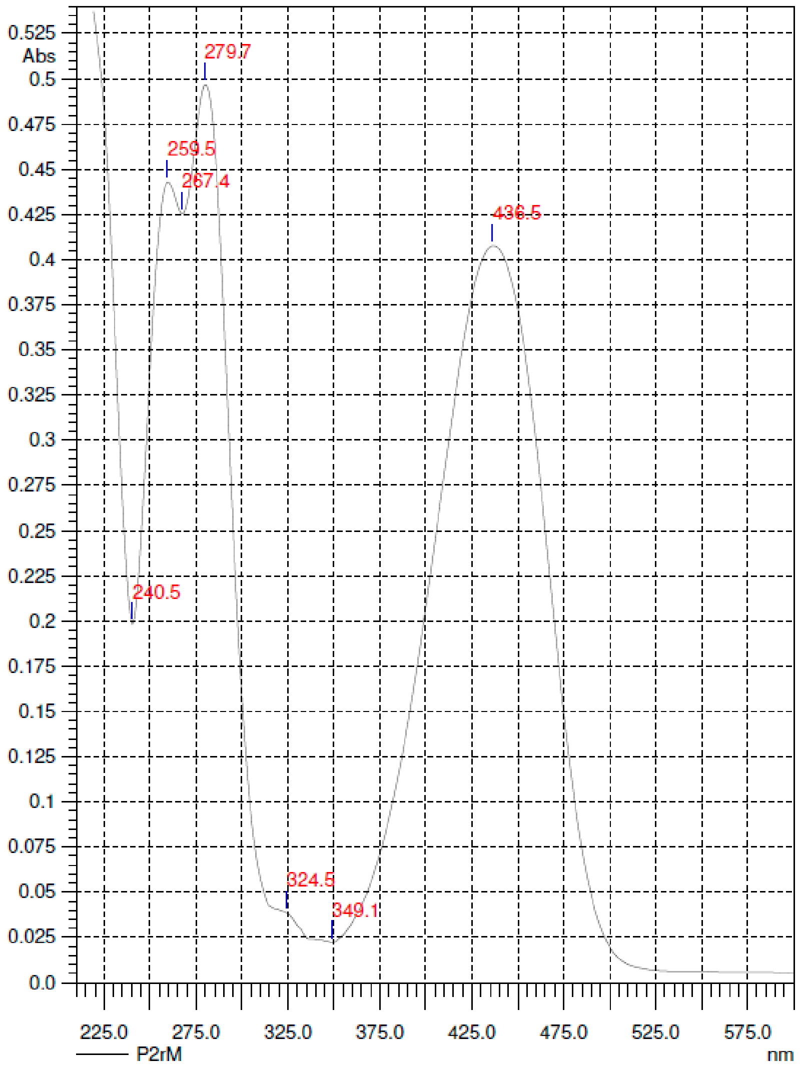

Figure S212. UV spectrum of **57**.

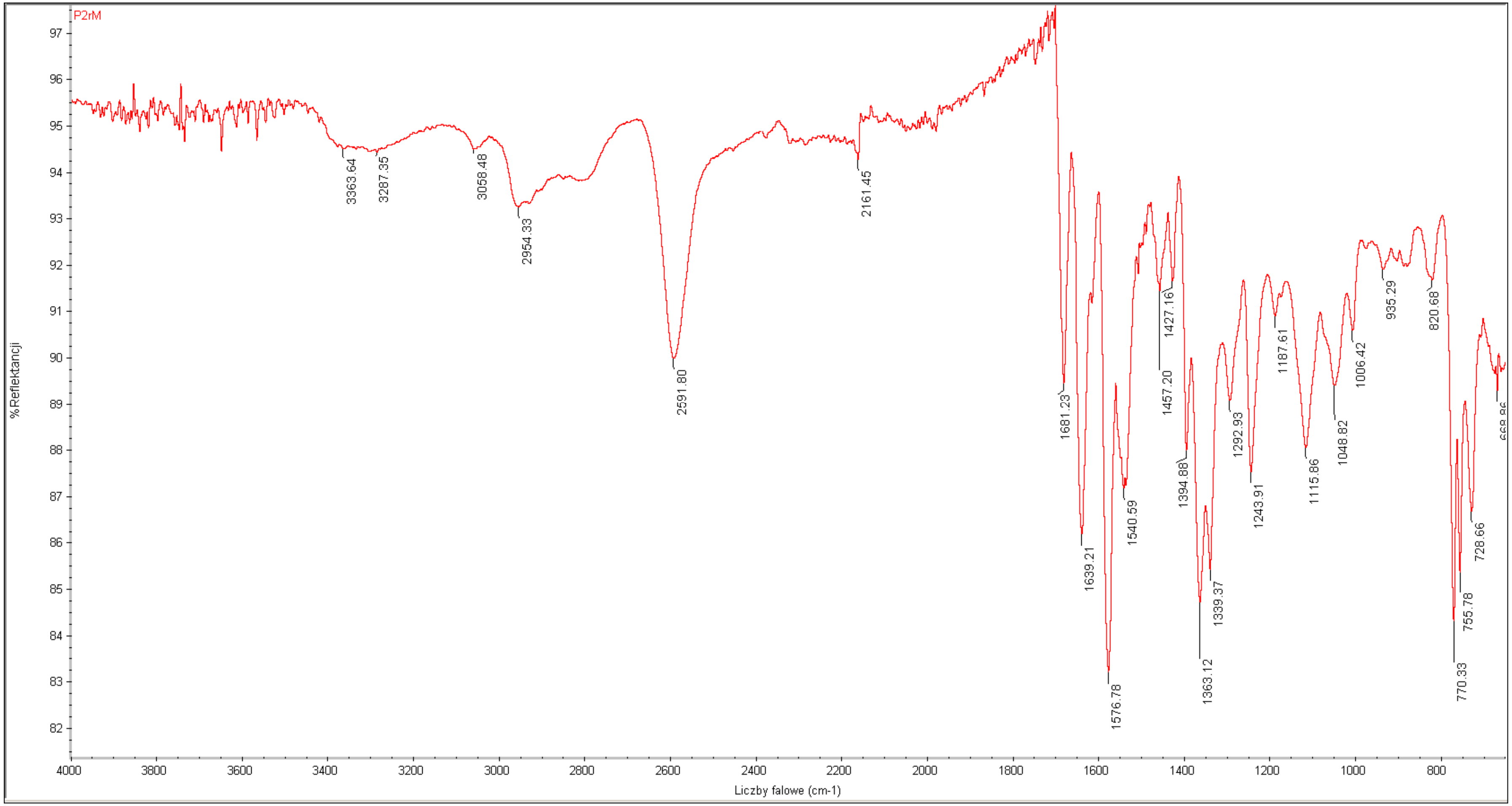

Figure S213. IR spectrum of **57**.

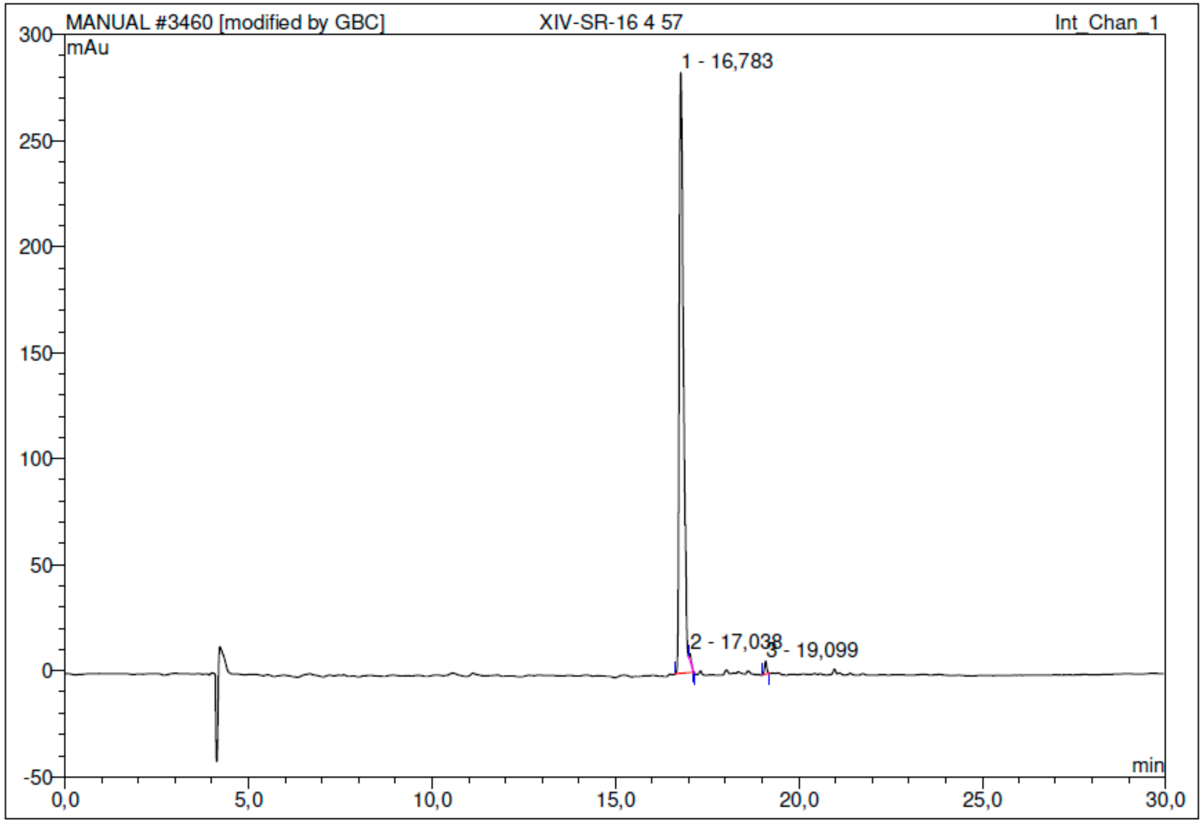

No.	Ret.Time min	Peak Name	Height mAu	Area mAu*min	Rel.Area %	Amount	Type
1	16,78	n.a.	283,289	38,902	98,30	n.a.	BMB*
2	17,04	n.a.	3,725	0,228	0,58	n.a.	Rd*
3	19,10	n.a.	6,136	0,445	1,13	n.a.	BMB*
**Total:**			293,151	39,576	100,00	0,000	

Figure S214. HPLC chromatogram of **57**.

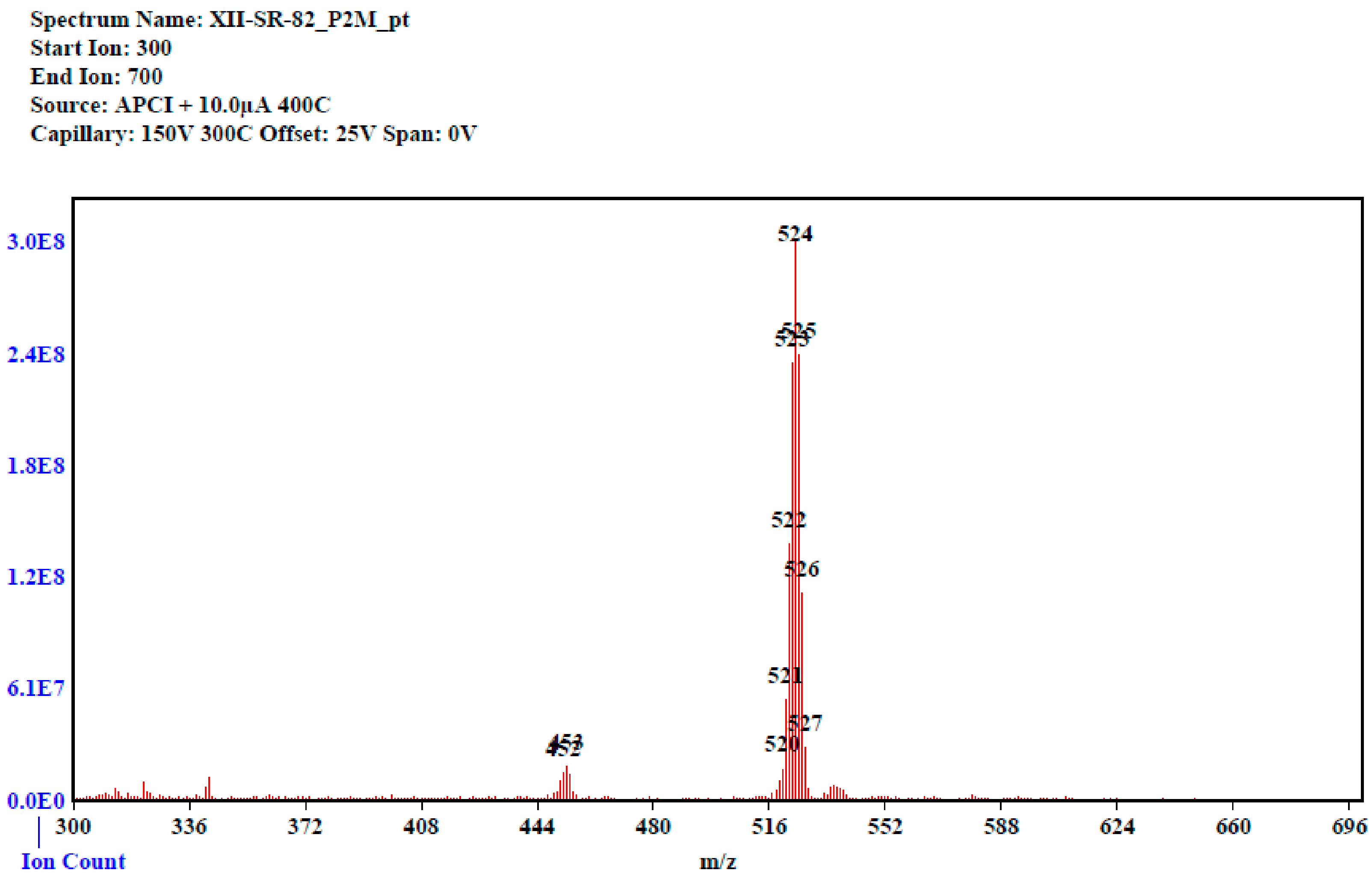

Figure S215. MS spectrum of **57**.

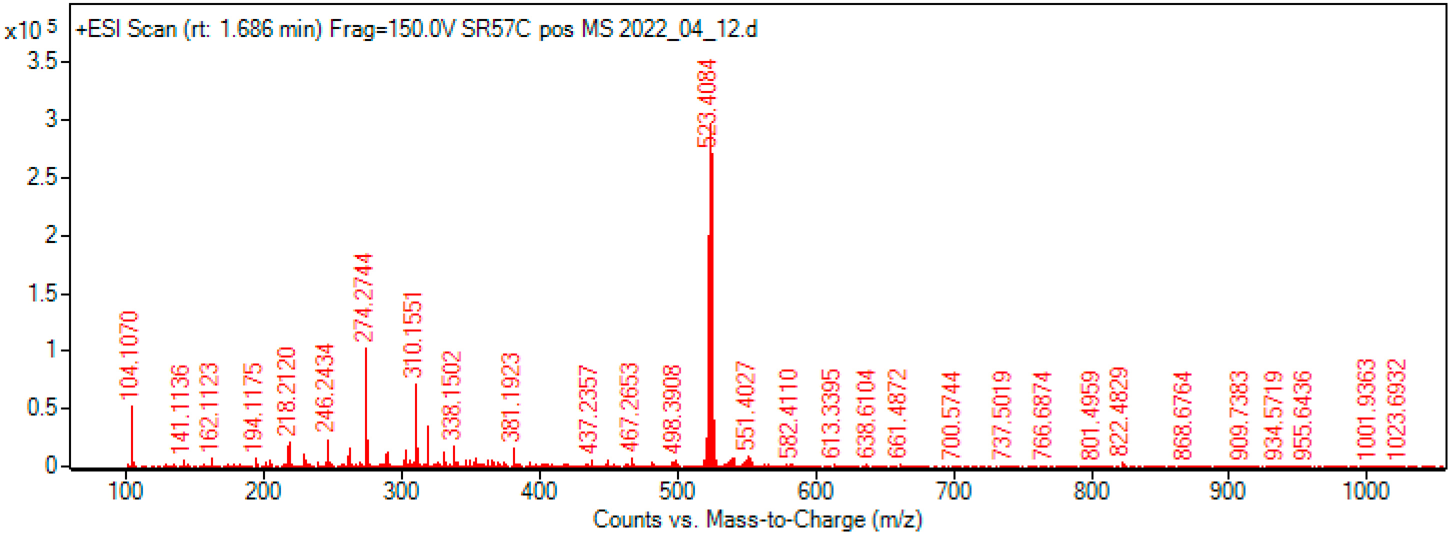

Figure S216. HRMS spectrum of **57**.

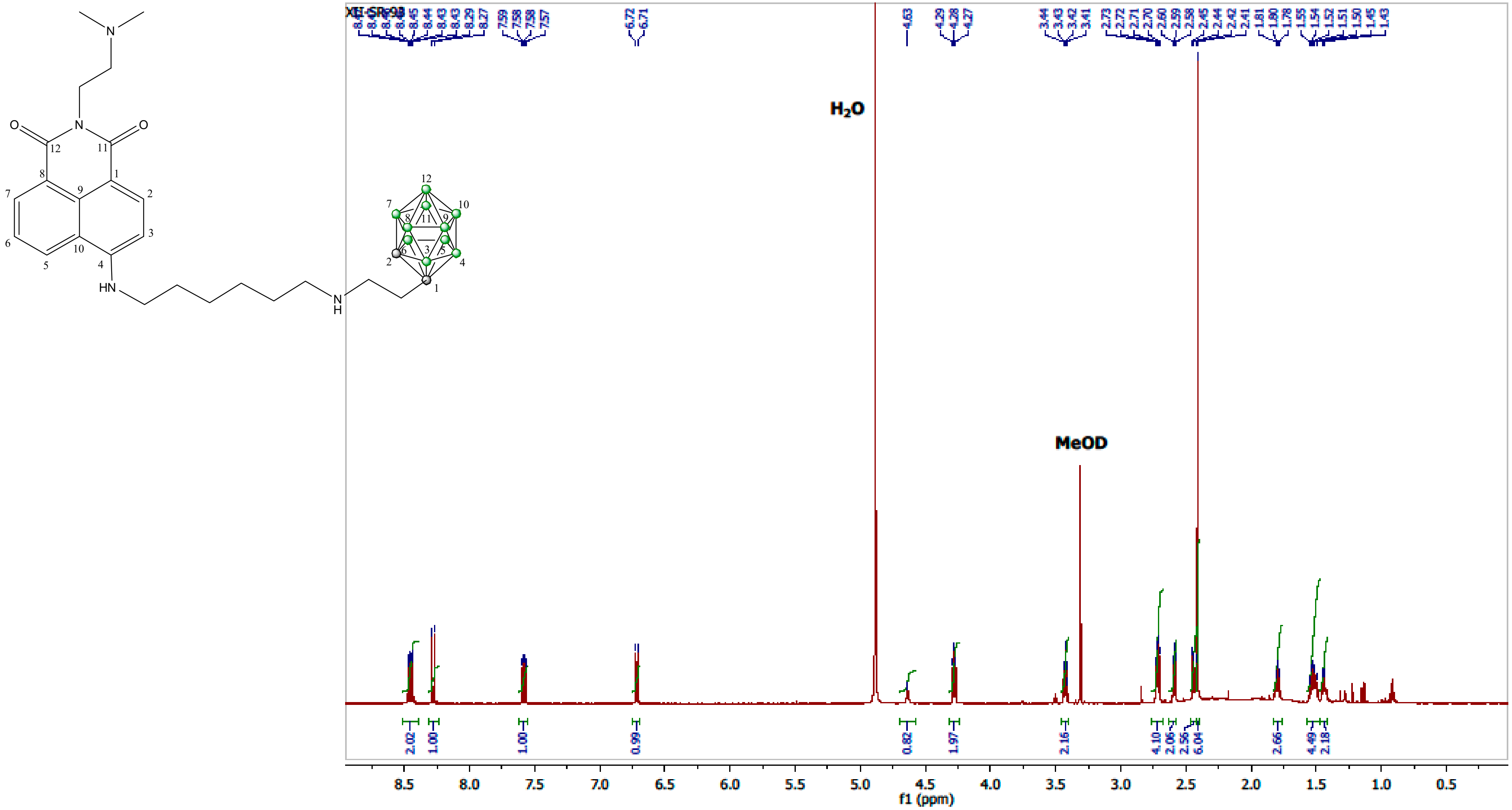

Figure S217. ^{1}H NMR spectrum of **58**.

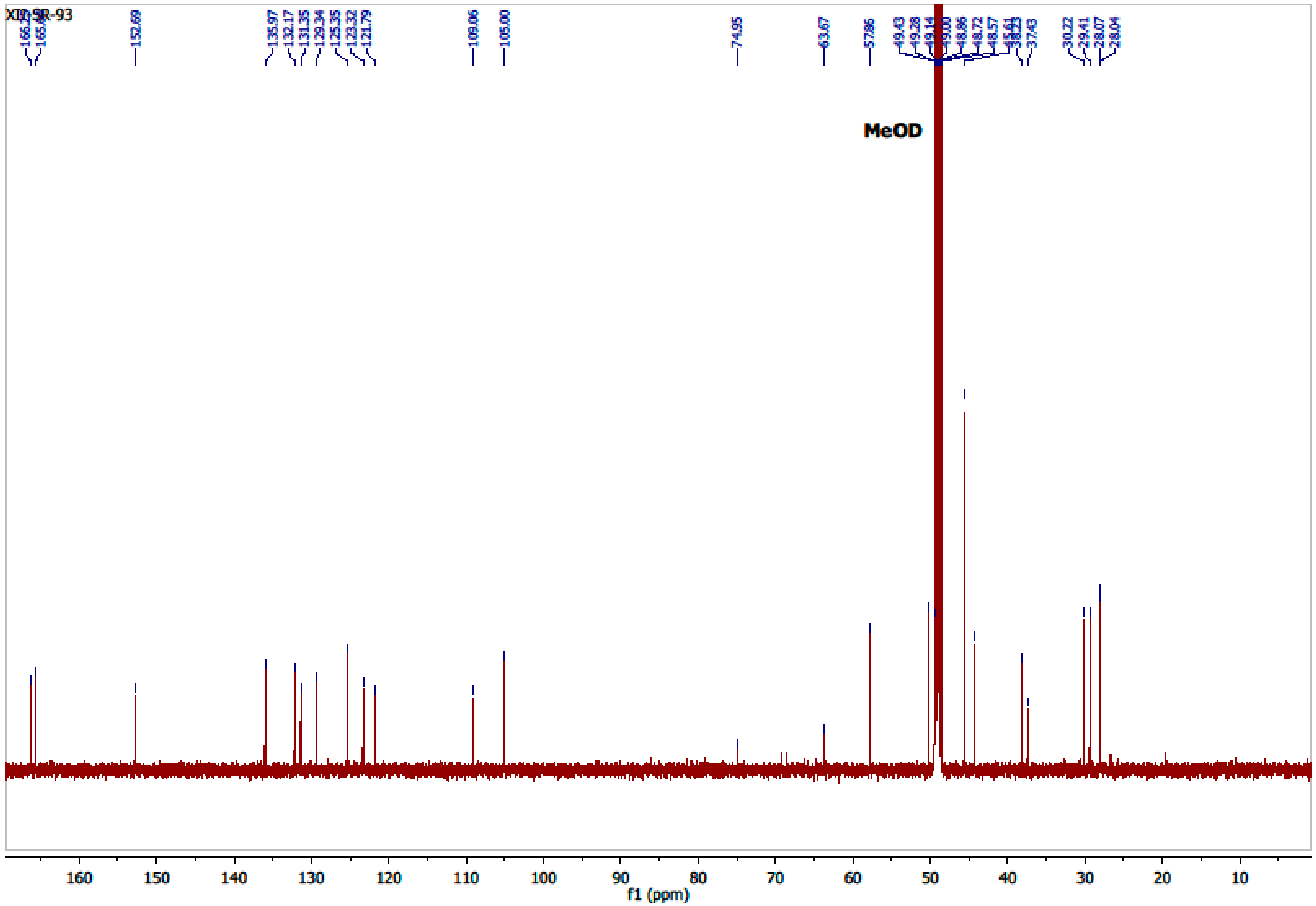

Figure S218. ^{13}C NMR spectrum of **58**.

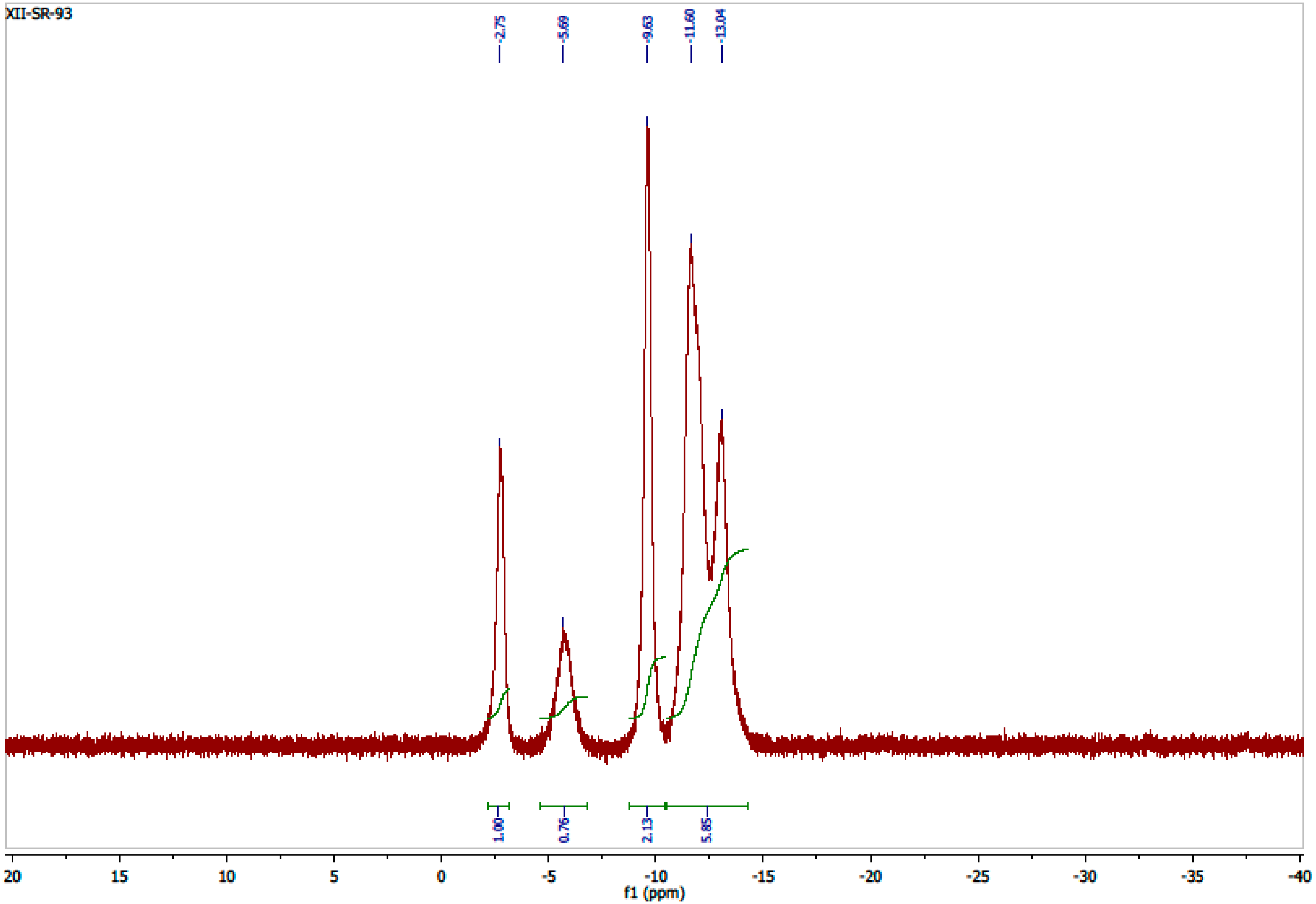

Figure S219. ^{11}B NMR spectrum of **58**.

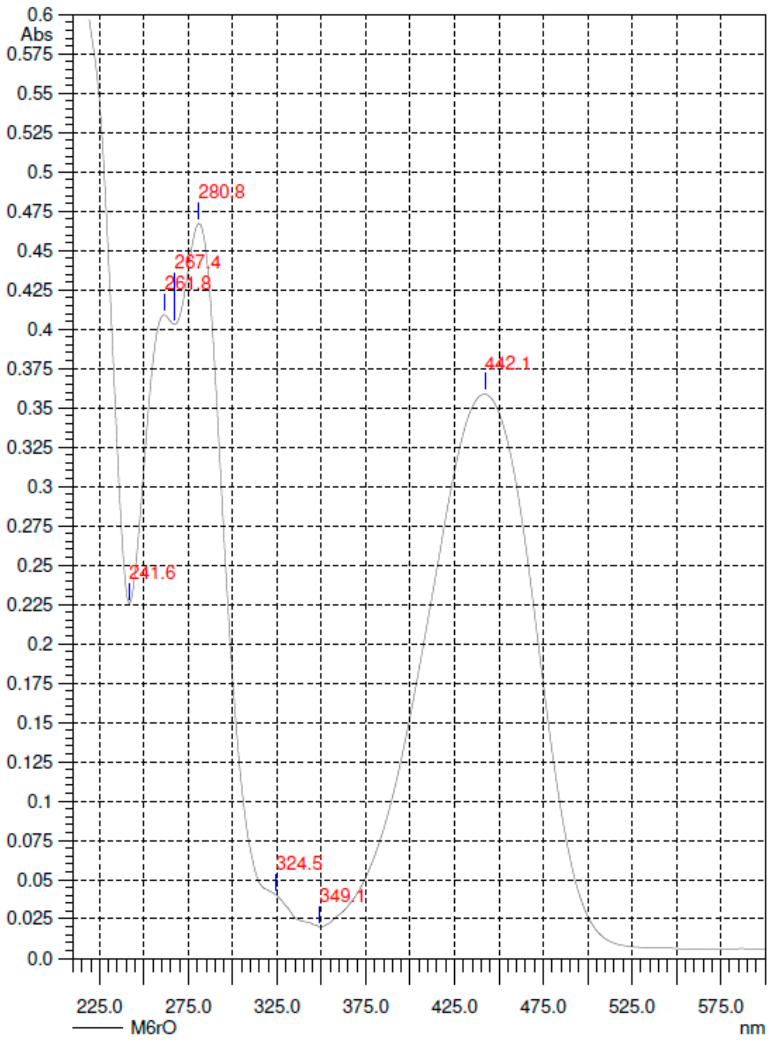

Figure S220. UV spectrum of **58**.

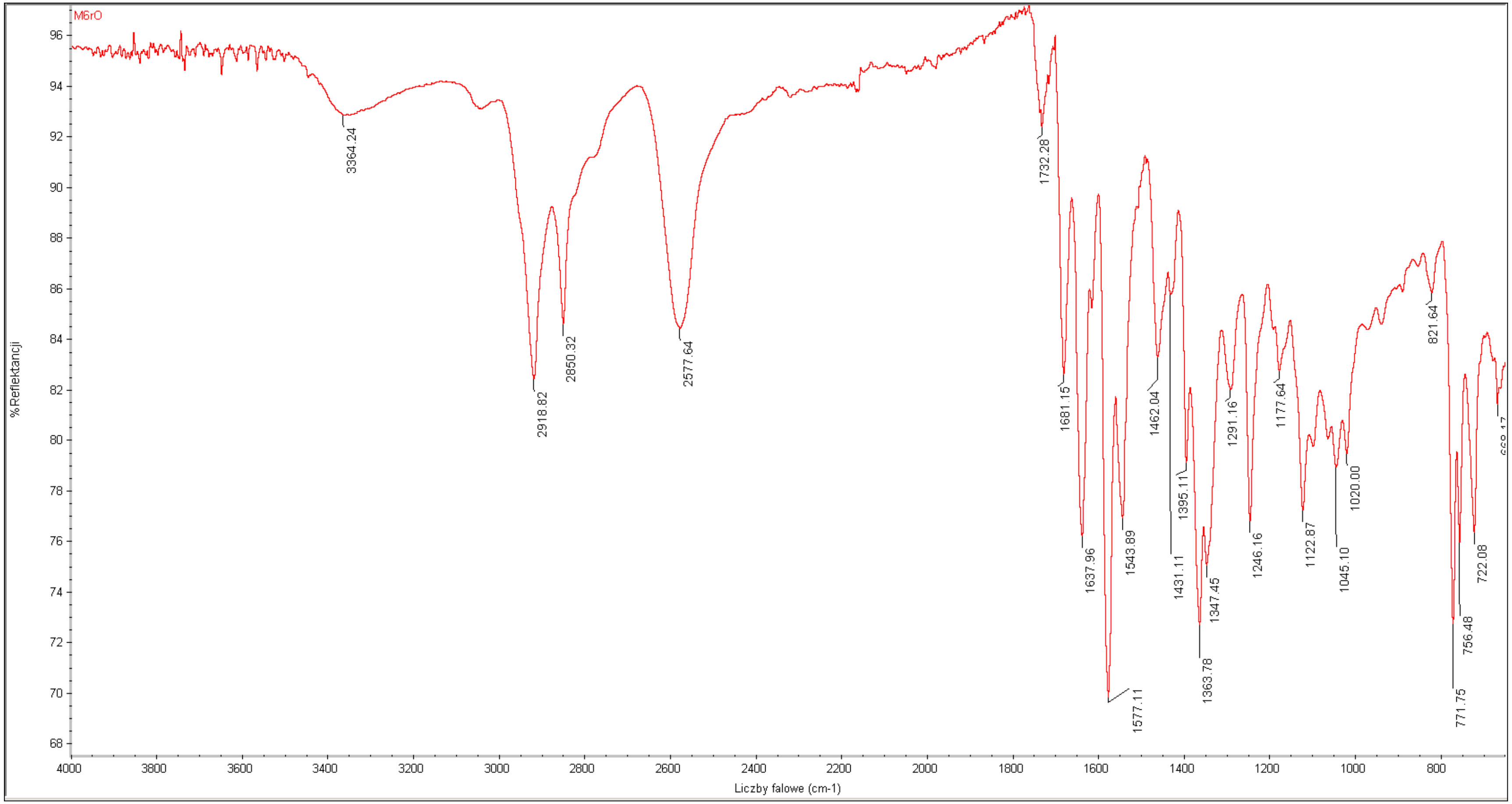

Figure S221. IR spectrum of **58**.

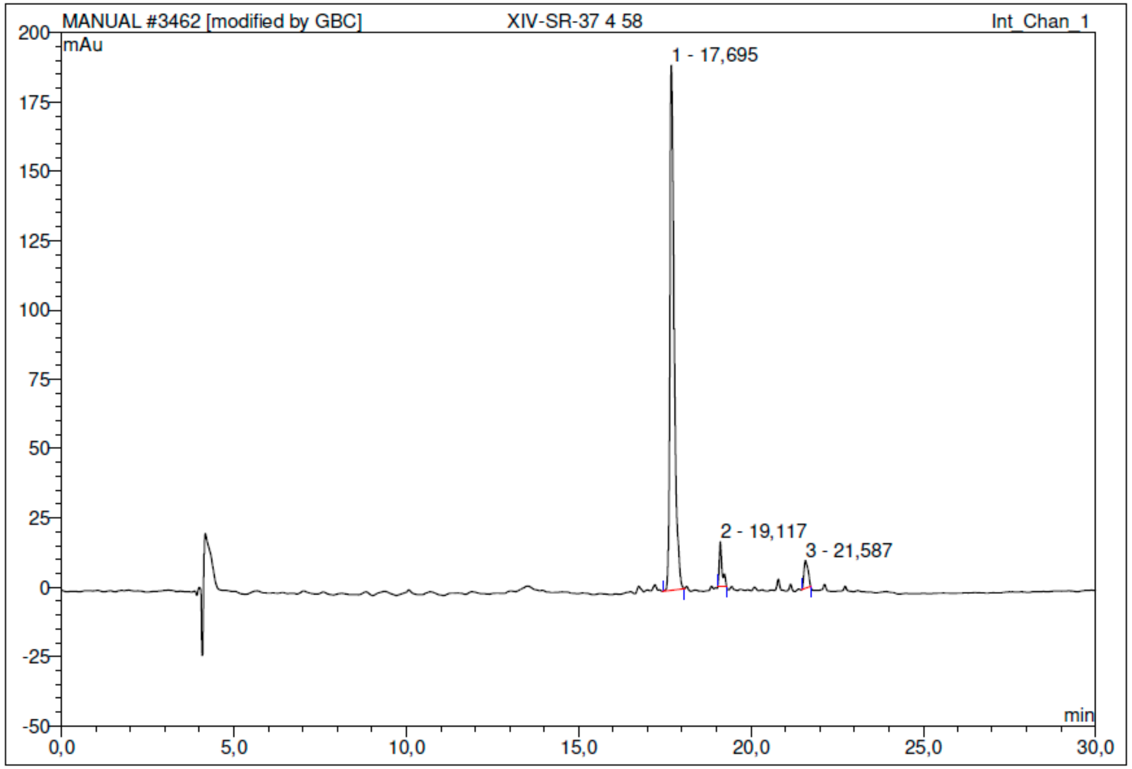

No.	Ret.Time min	Peak Name	Height mAu	Area mAu*min	Rel.Area %	Amount	Type
1	17,70	n.a.	189,309	27,709	90,33	n.a.	BMB
2	19,12	n.a.	15,889	1,610	5,25	n.a.	BMB*
3	21,59	n.a.	10,015	1,356	4,42	n.a.	BMB*
Total:			215,213	30,675	100,00	0,000	

Figure S222. HPLC chromatogram of **58**.

Spectrum Name: XII-SR-93_pt
Start Ion: 300
End Ion: 700
Source: APCI + 10.0μA 400C
Capillary: 150V 300C Offset: 25V Span: 0V

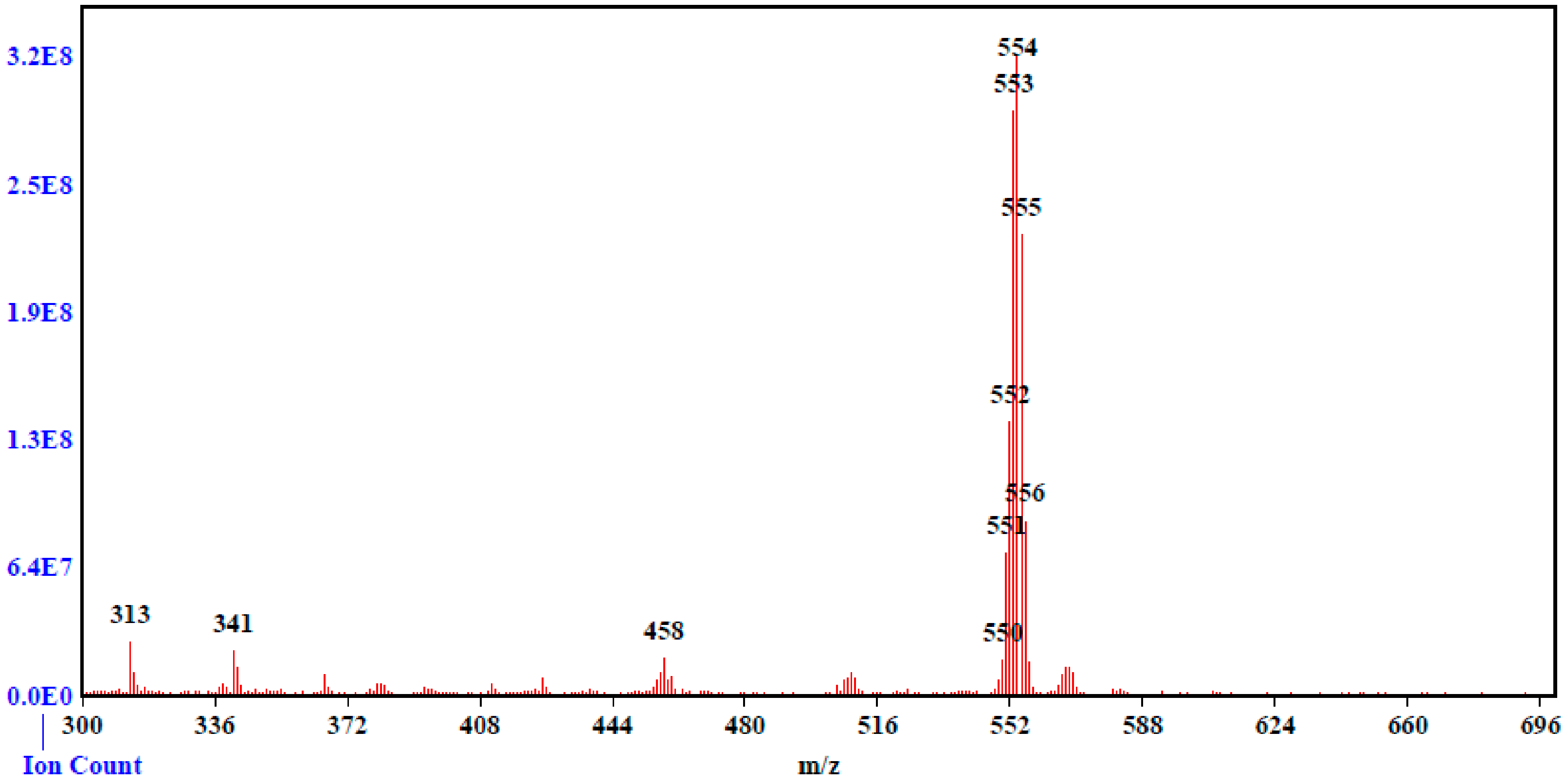

Figure S223. MS spectrum of **58**.

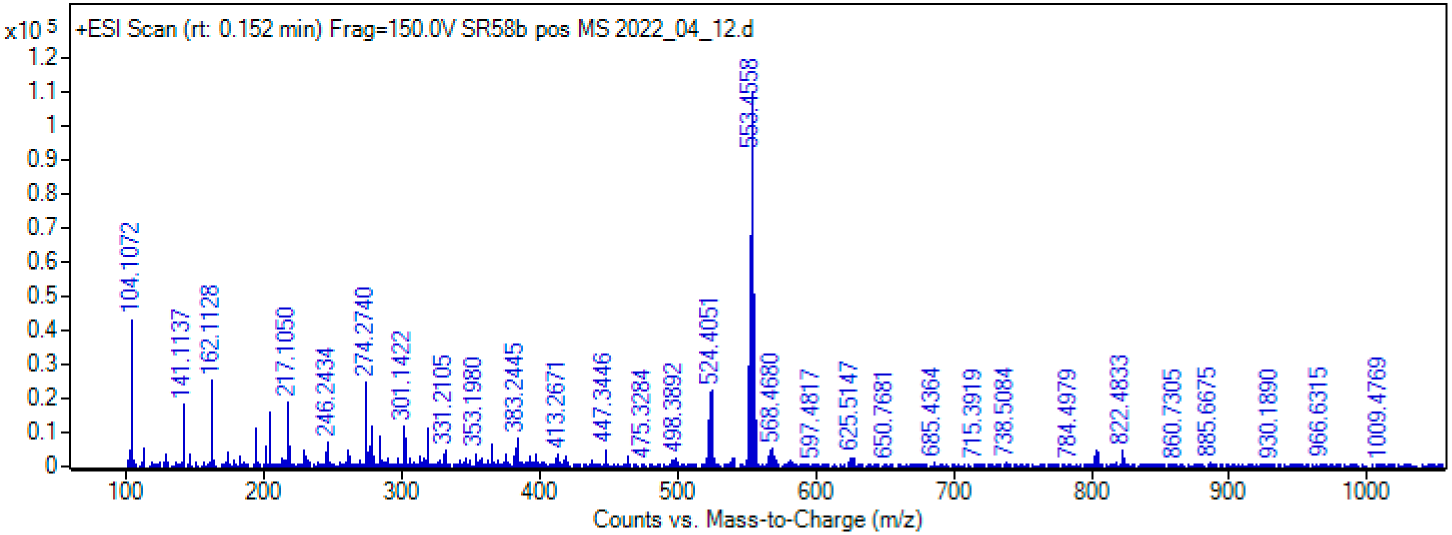

Figure S224. HRMS spectrum of **58**.

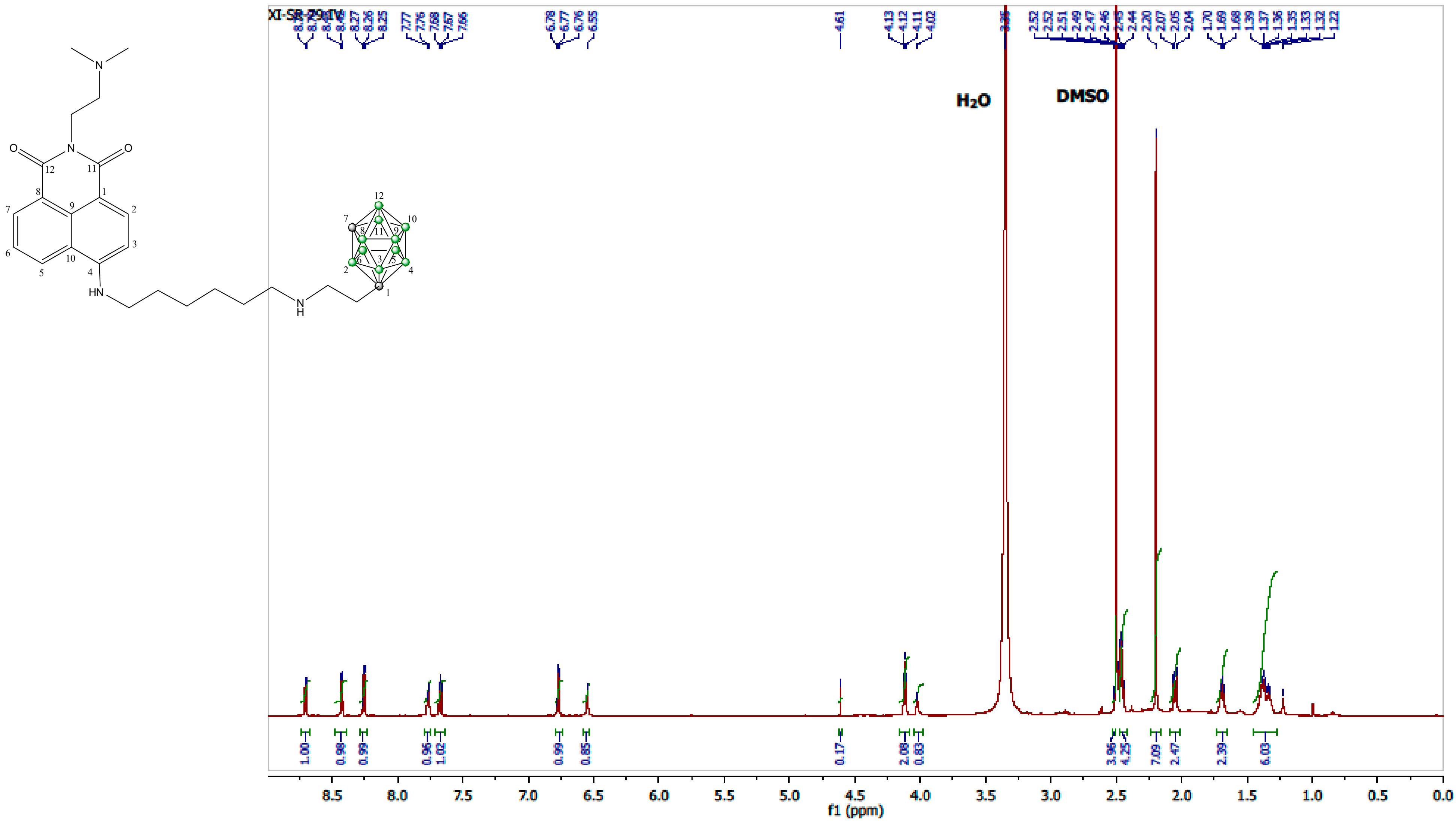

Figure S225. ^{1}H NMR spectrum of **59**.

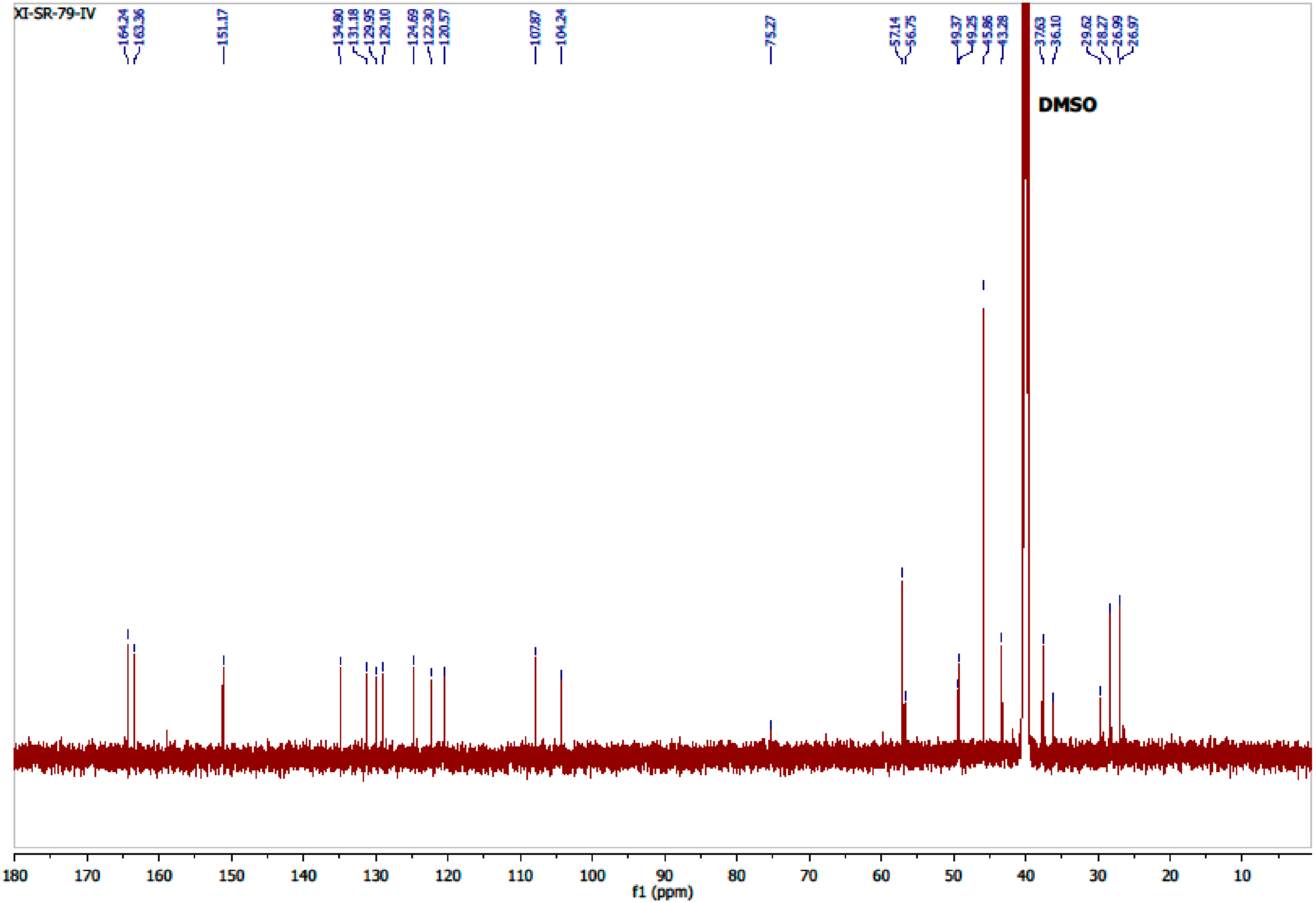

Figure S226. ^{13}C NMR spectrum of **59**.

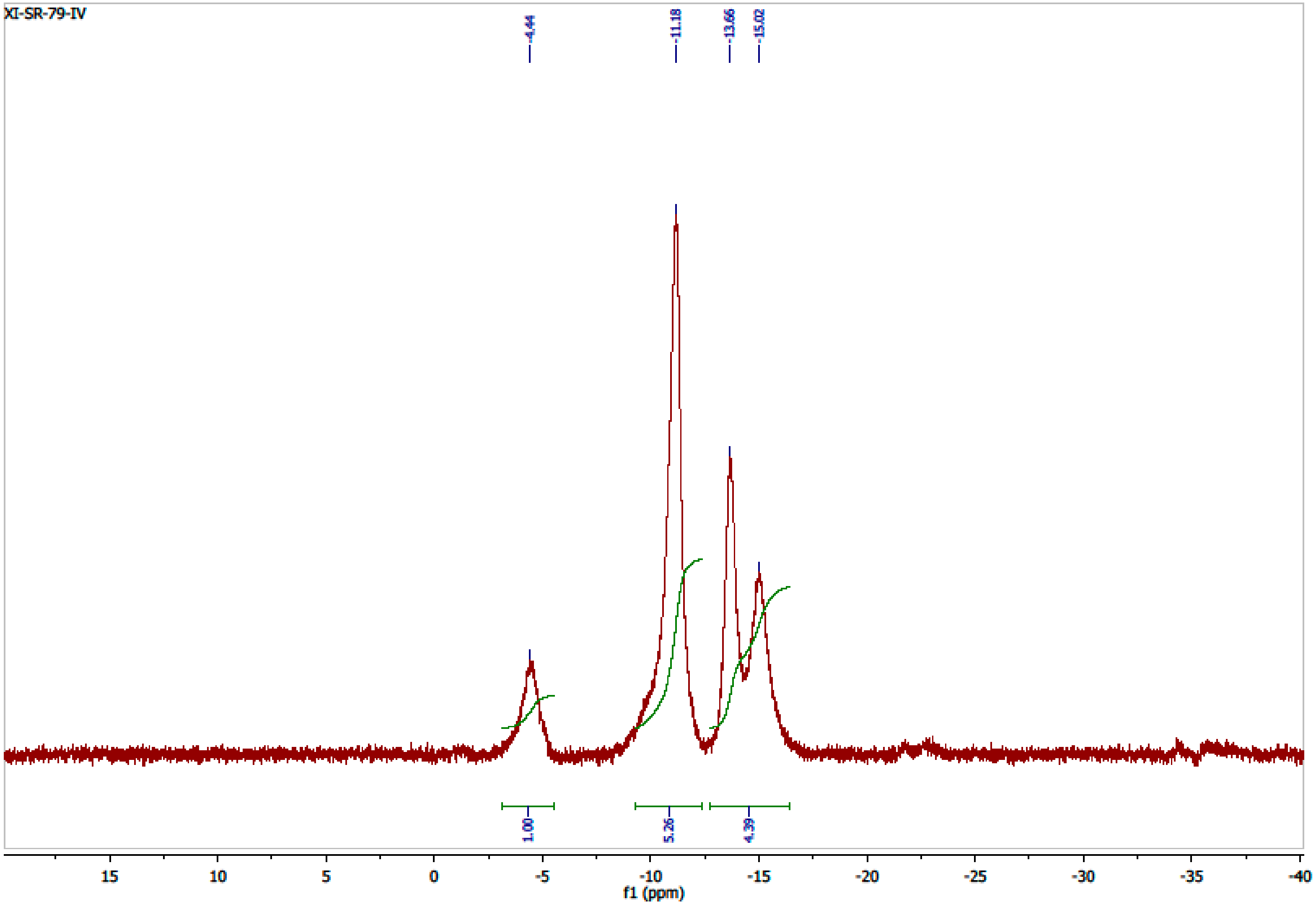

Figure S227. ^{11}B NMR spectrum of **59**.

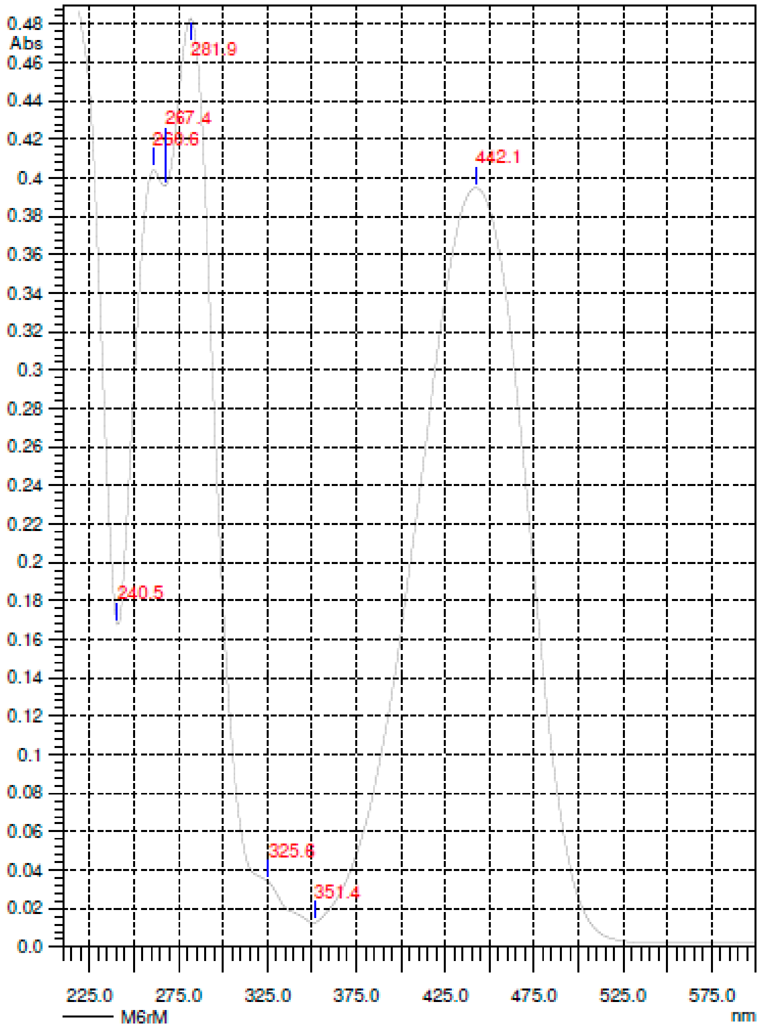

Figure S228. UV spectrum of **59**.

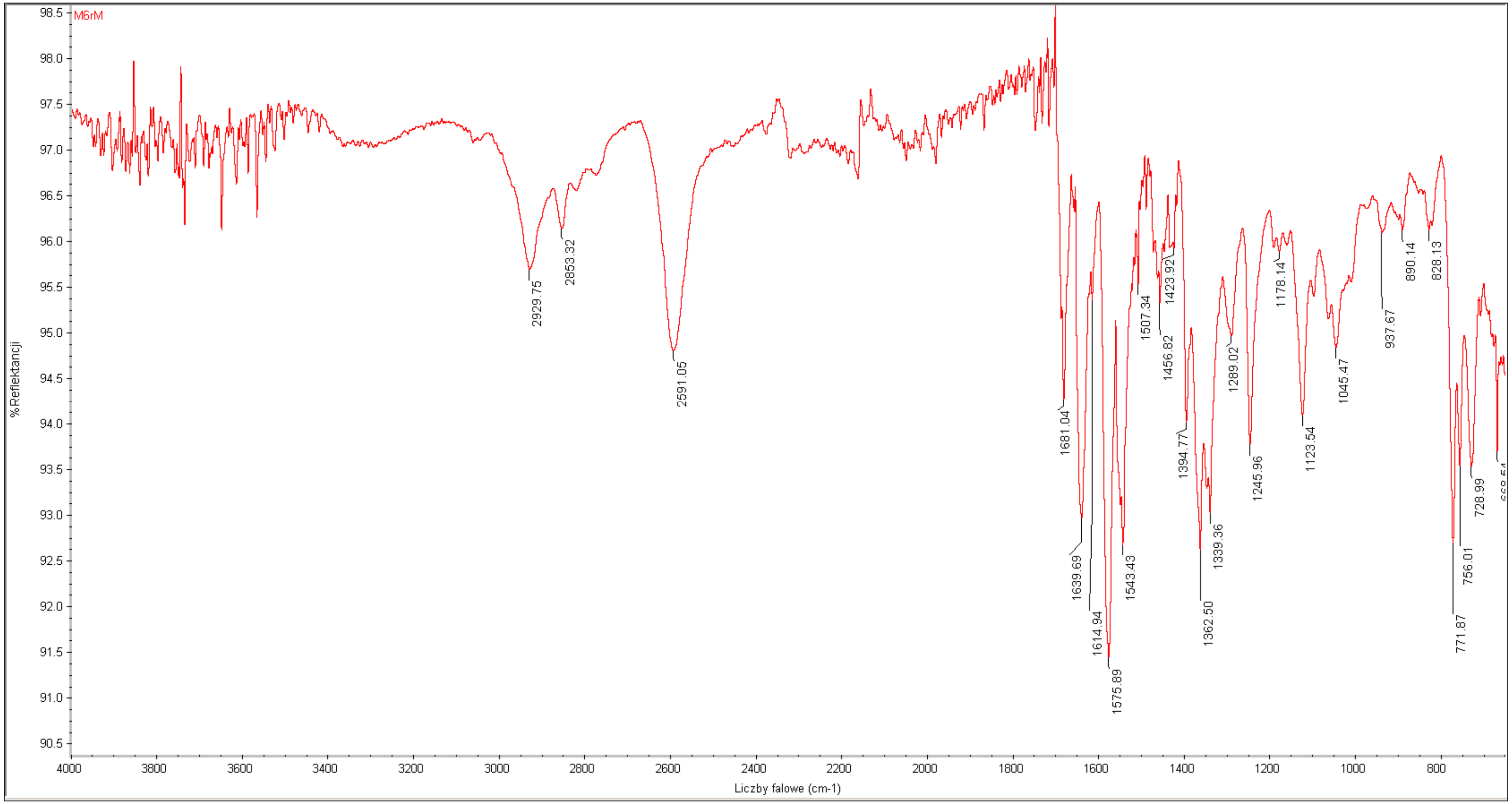

Figure S229. IR spectrum of **59**.

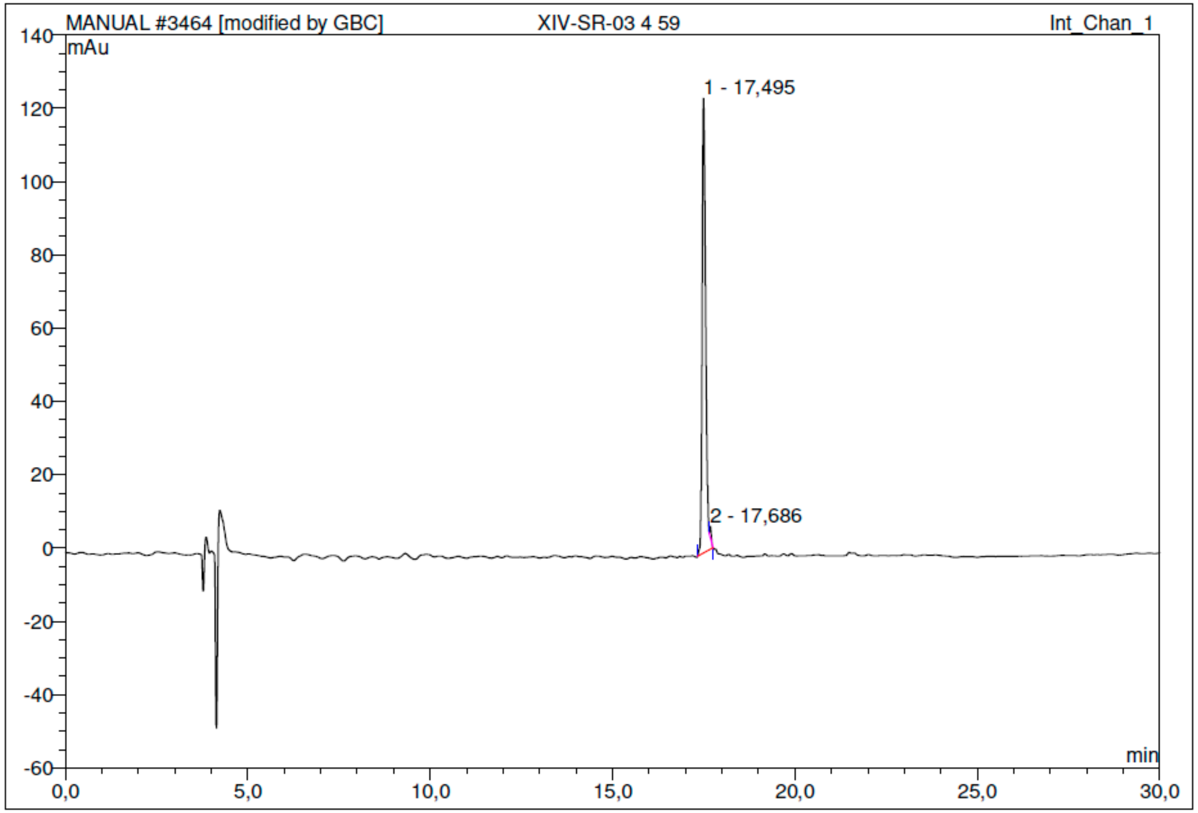

No.	Ret.Time min	Peak Name	Height mAu	Area mAu*min	Rel.Area %	Amount	Type
1	17,50	n.a.	124,038	13,973	98,89	n.a.	BMB*
2	17,69	n.a.	3,177	0,157	1,11	n.a.	Rd*
Total:			127,215	14,130	100,00	0,000	

Figure S230. HPLC chromatogram of **59**.

Spectrum Name: XI-SR-79_9-10_pt2
Start Ion: 200
End Ion: 800
Source: APCI + 10.0µA 400C
Capillary: 150V 300C Offset: 25V Span: 0V

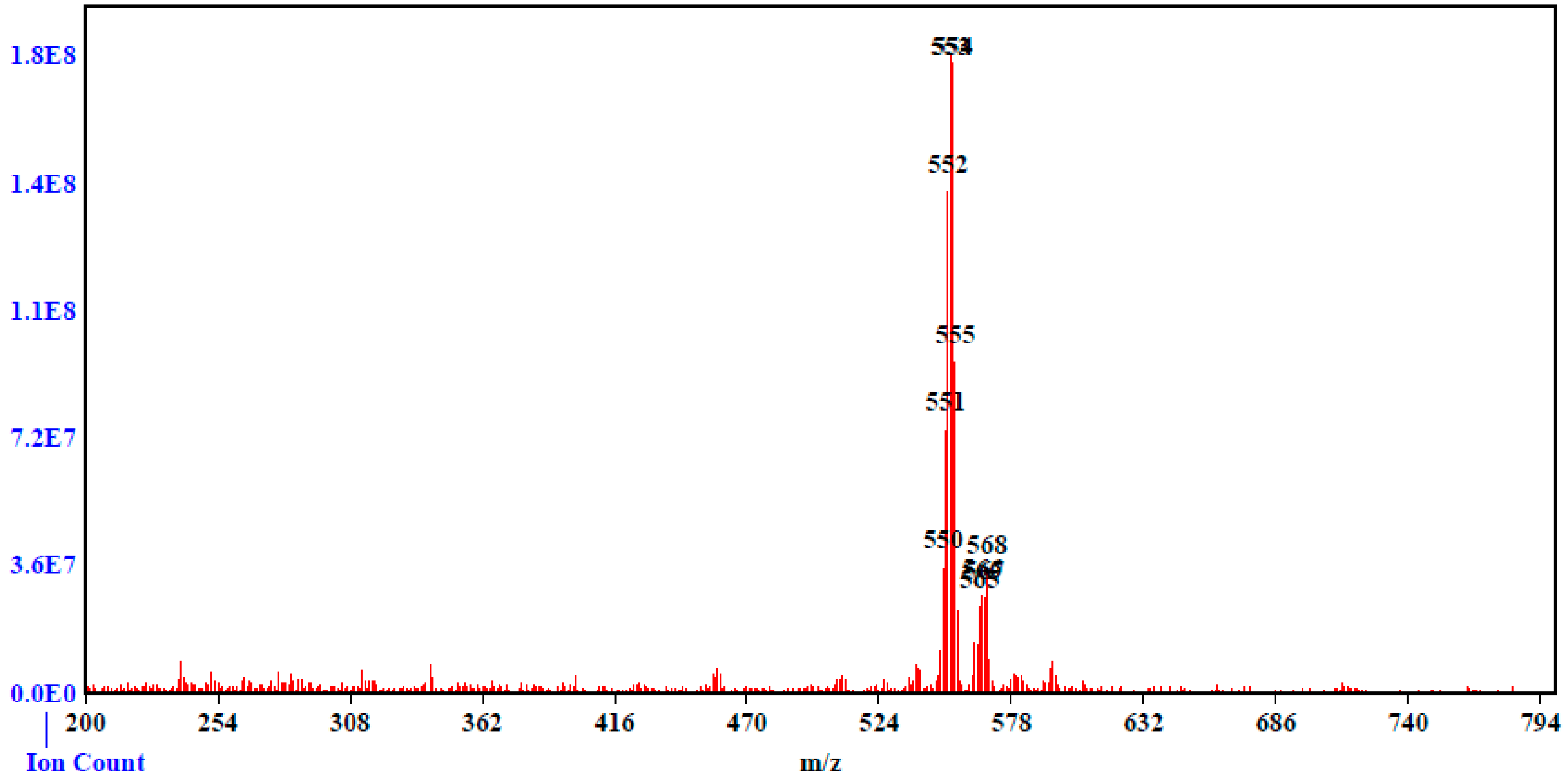

Figure S231. MS spectrum of **59**.

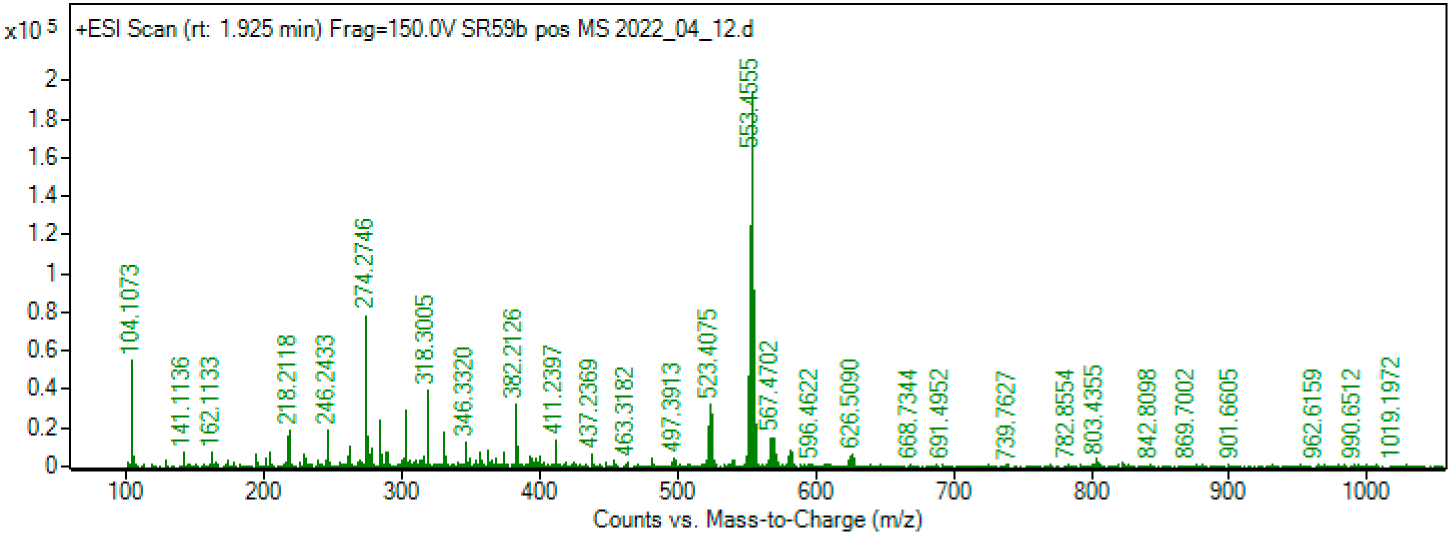

Figure S232. HRMS spectrum of **59**.

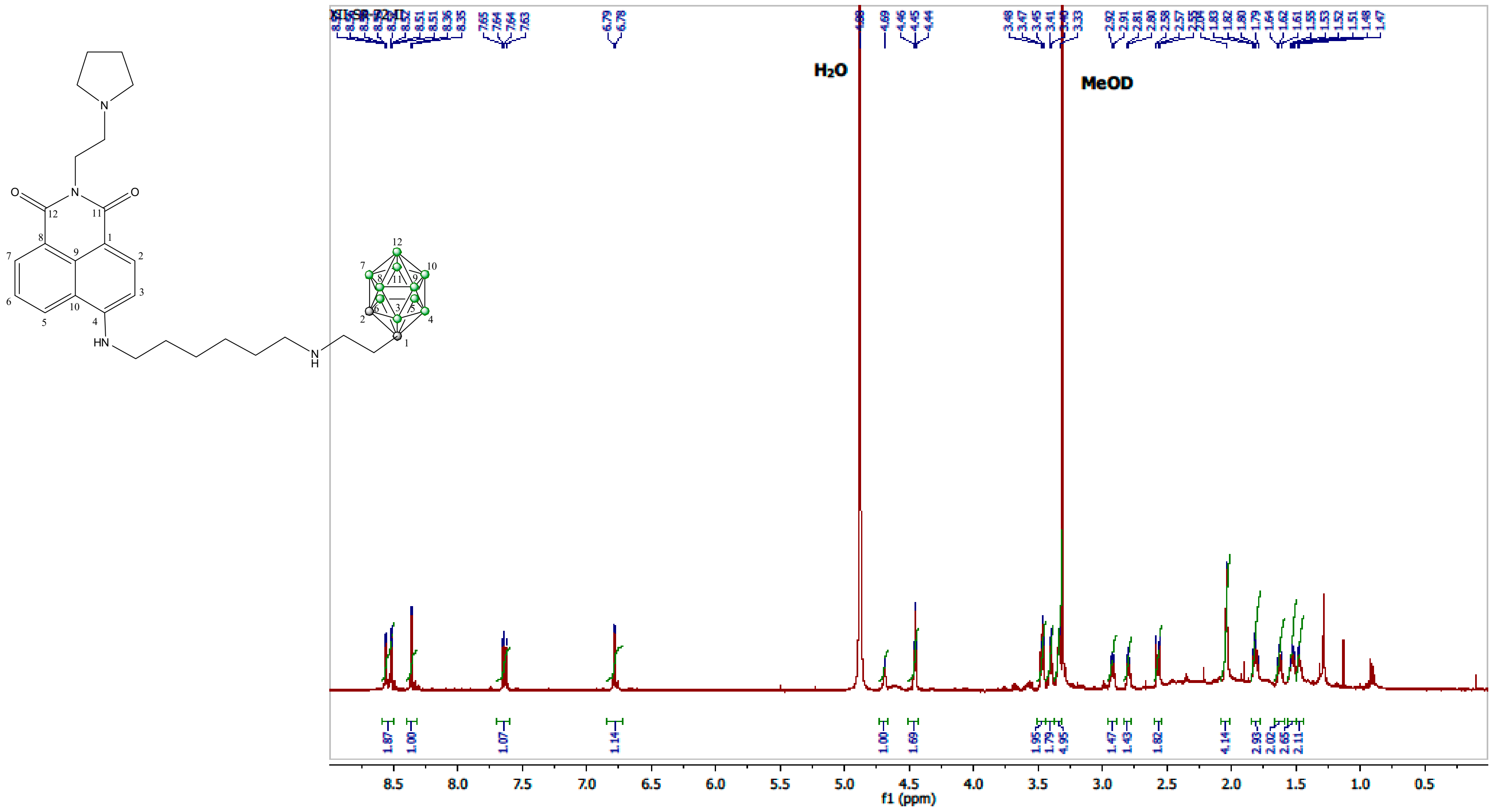

Figure S233. ^{1}H NMR spectrum of **60**.

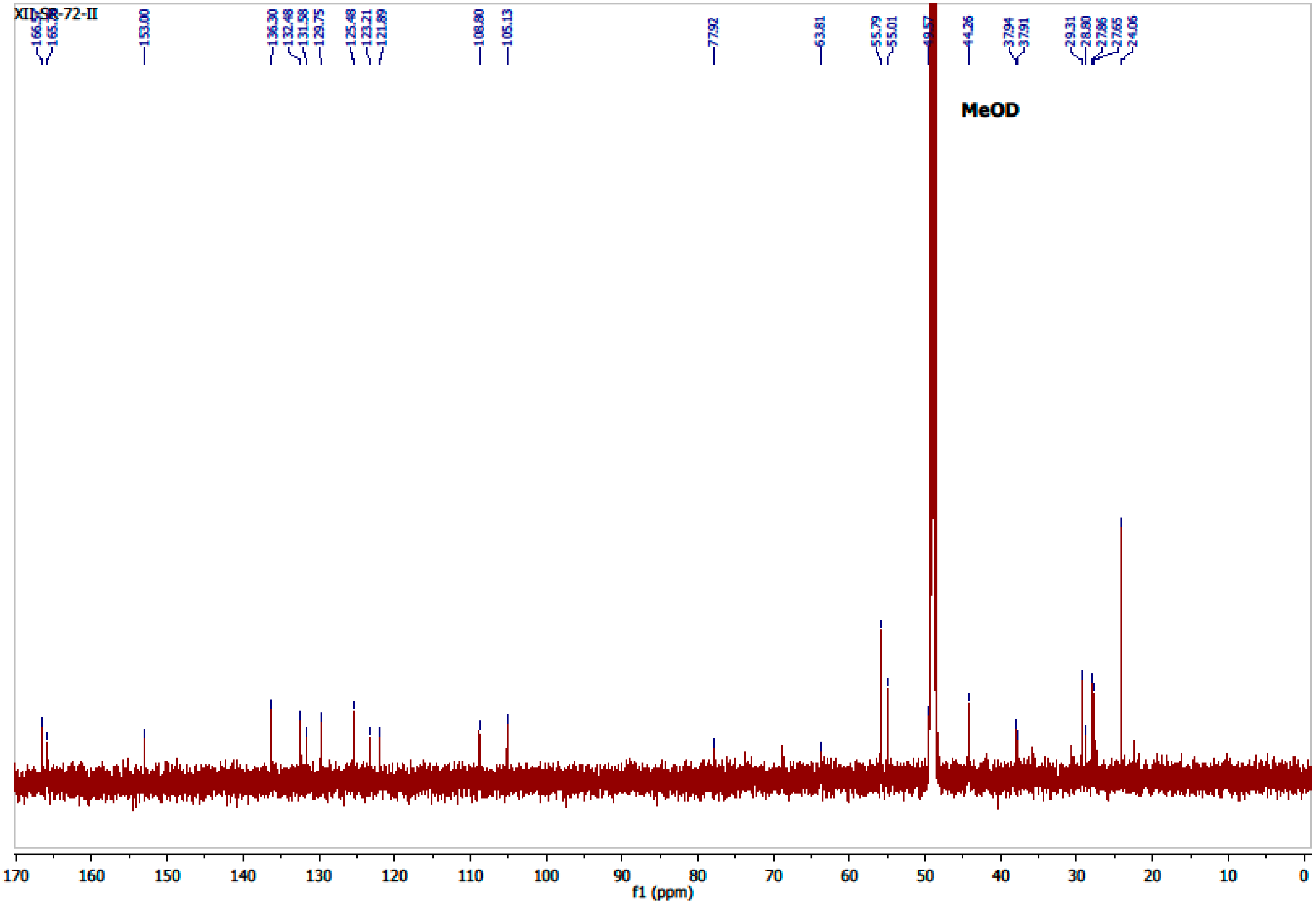

Figure S234. ^{13}C NMR spectrum of **60**.

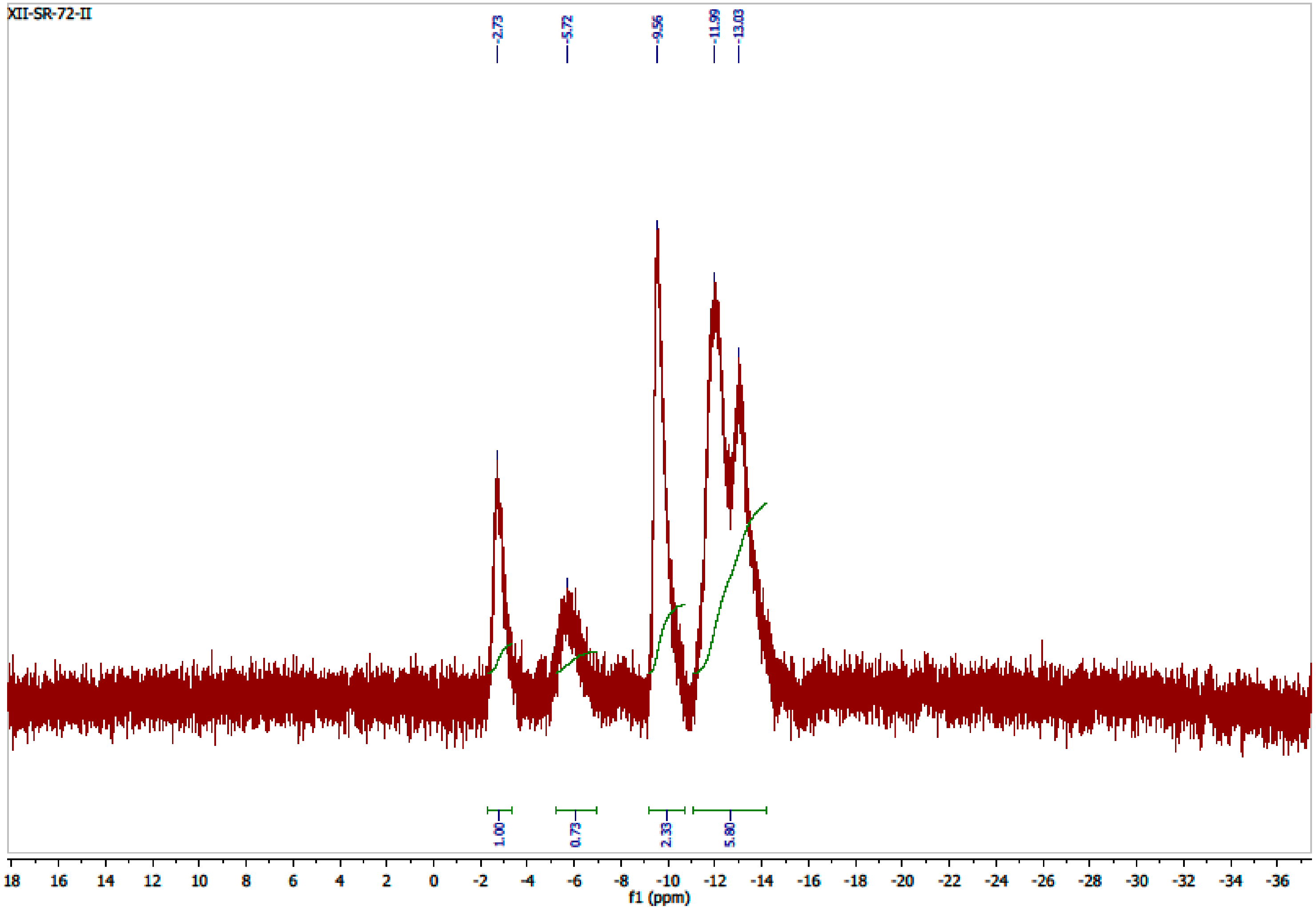

Figure S235. ^{11}B NMR spectrum of **60**.

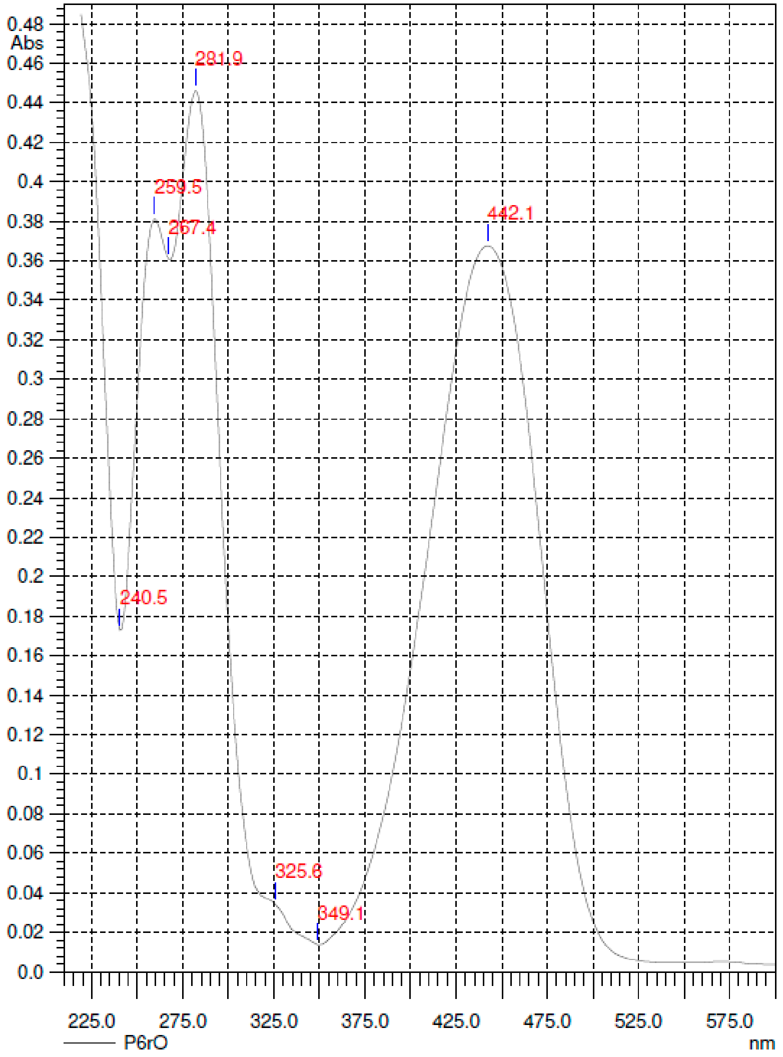

Figure S236. UV spectrum of **60**.

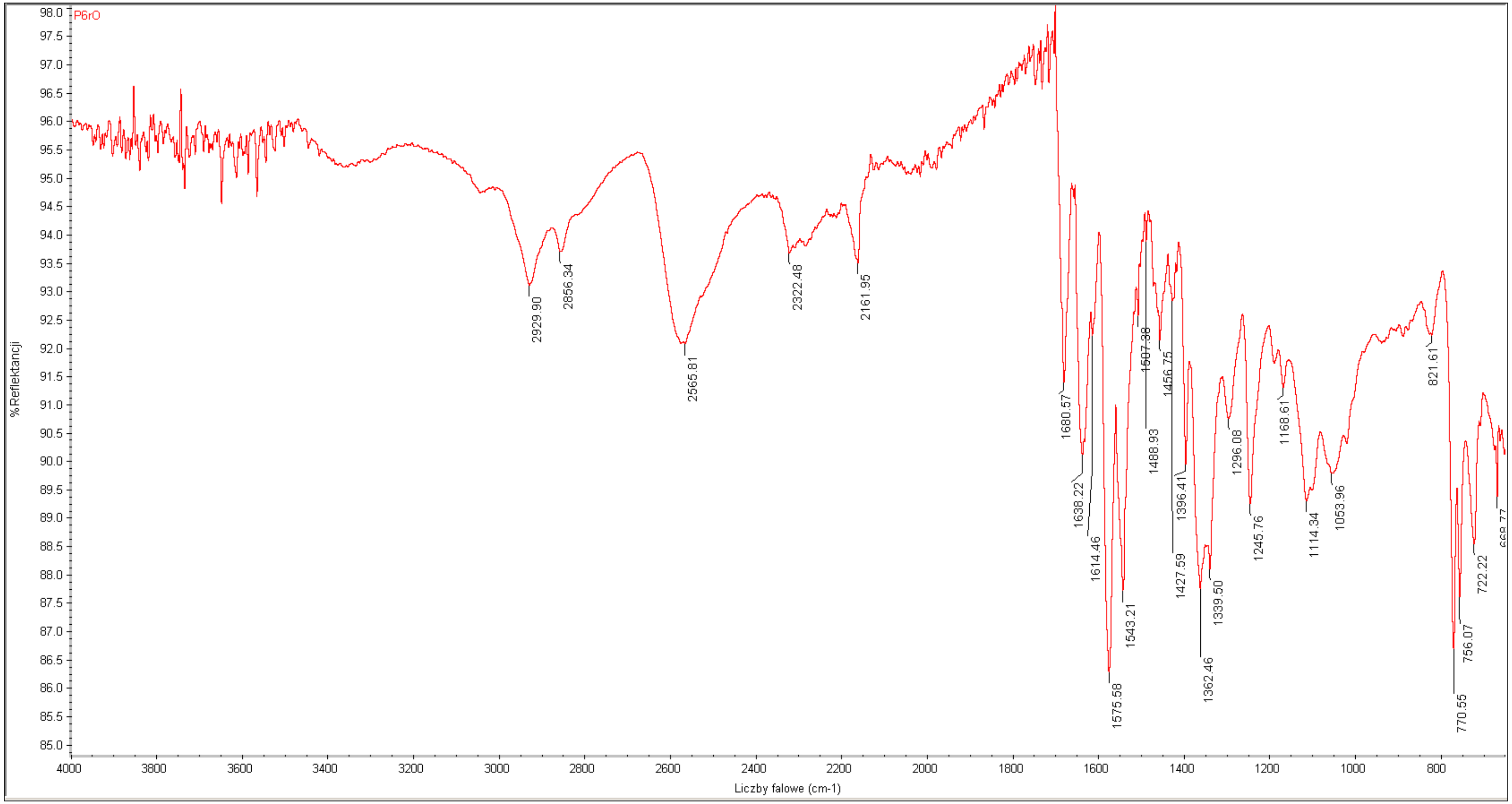

Figure S237. IR spectrum of **60**.

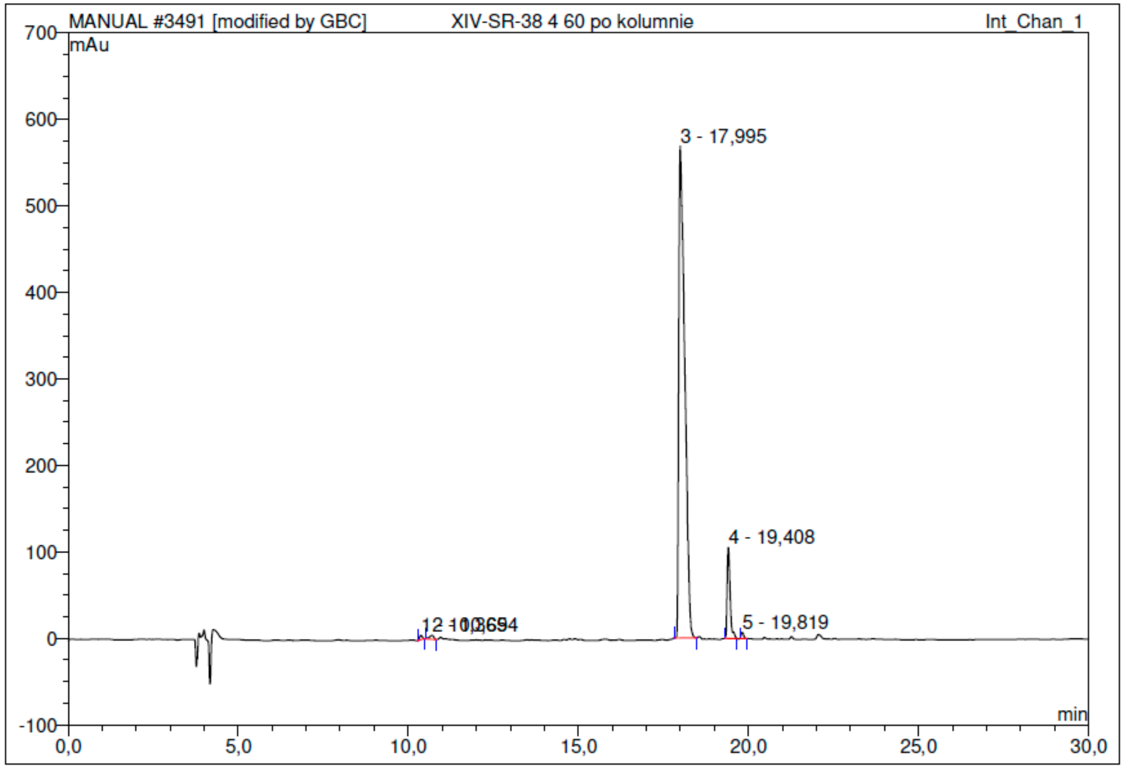

No.	Ret.Time min	Peak Name	Height mAu	Area mAu*min	Rel.Area %	Amount	Type
1	10,37	n.a.	5,024	0,494	0,38	n.a.	BMB*
2	10,69	n.a.	4,593	0,683	0,53	n.a.	BMB*
3	17,99	n.a.	568,405	116,093	90,40	n.a.	BMB
4	19,41	n.a.	105,257	10,596	8,25	n.a.	BMB*
5	19,82	n.a.	6,960	0,562	0,44	n.a.	BMB*
Total:			690,239	128,428	100,00	0,000	

Figure S238. HPLC chromatogram of **60**.

Spectrum Name: XIV-SR-P6O_pt
Start Ion: 100
End Ion: 700
Source: APCI + 10.0µA 400C
Capillary: 150V 300C Offset: 25V Span: 0V

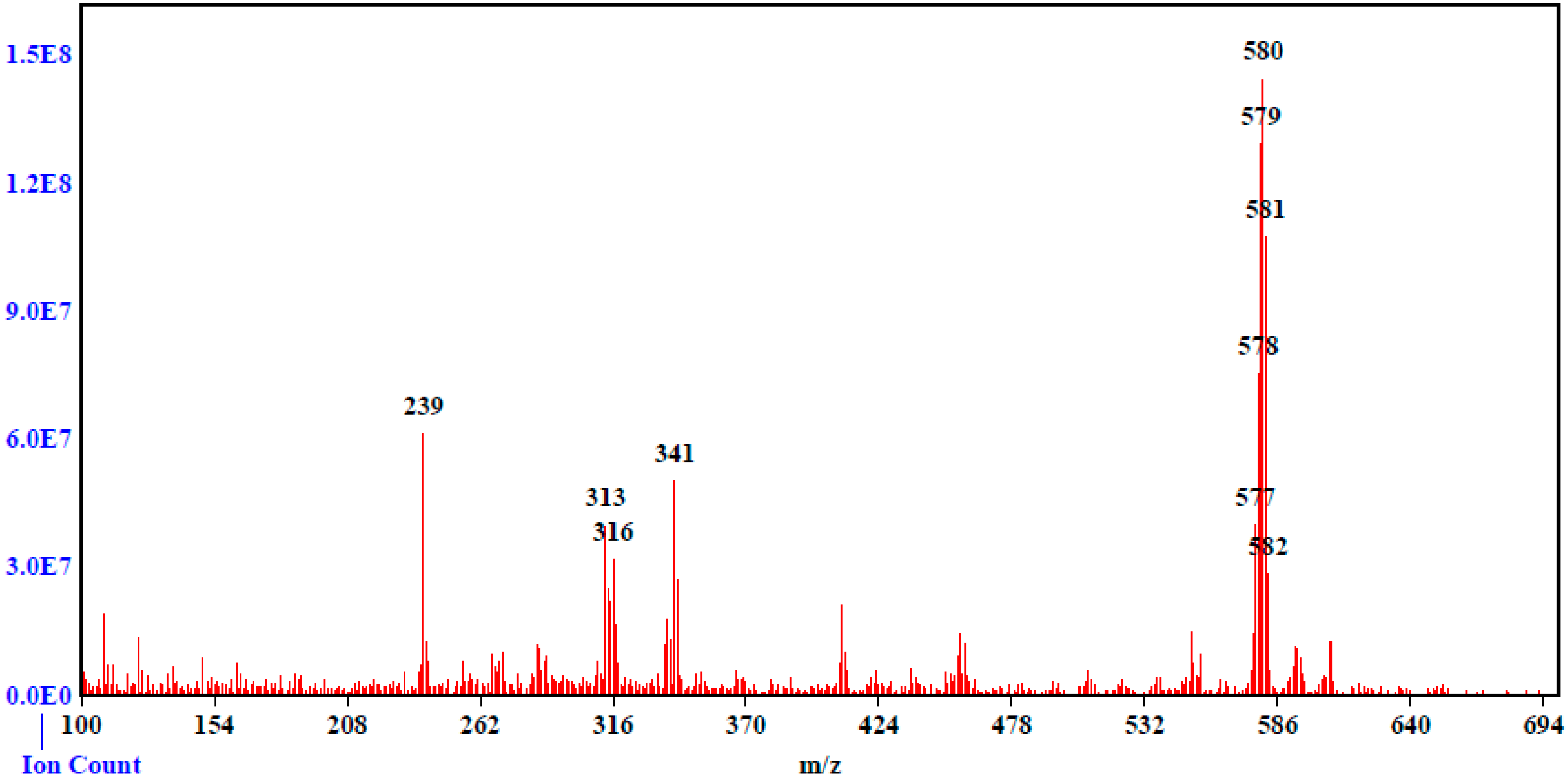

Figure S239. MS spectrum of **60**.

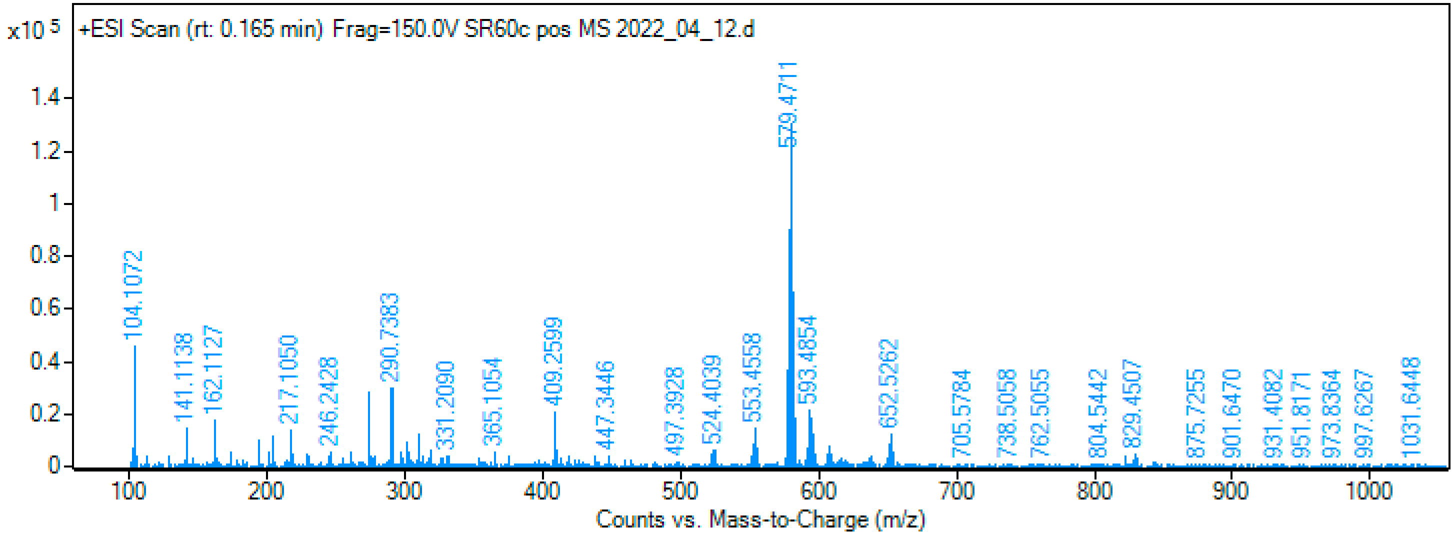

Figure S240. HRMS spectrum of **60**.

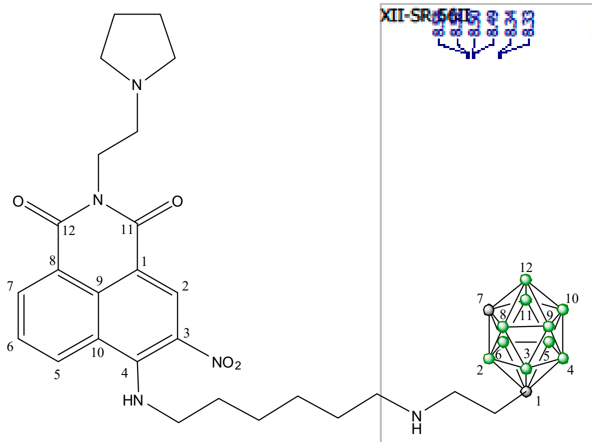

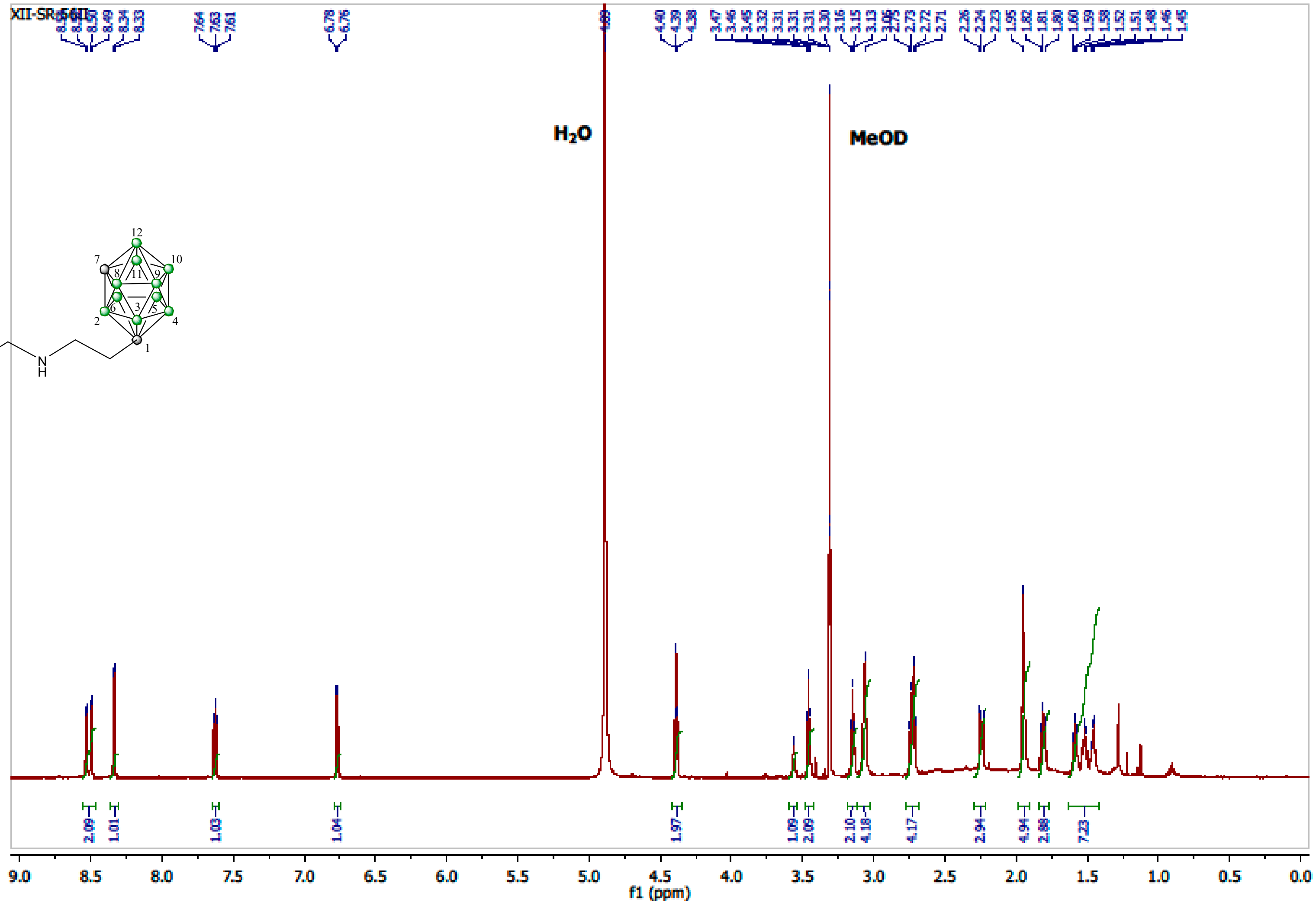

Figure S241. ^{1}H NMR spectrum of **61**.

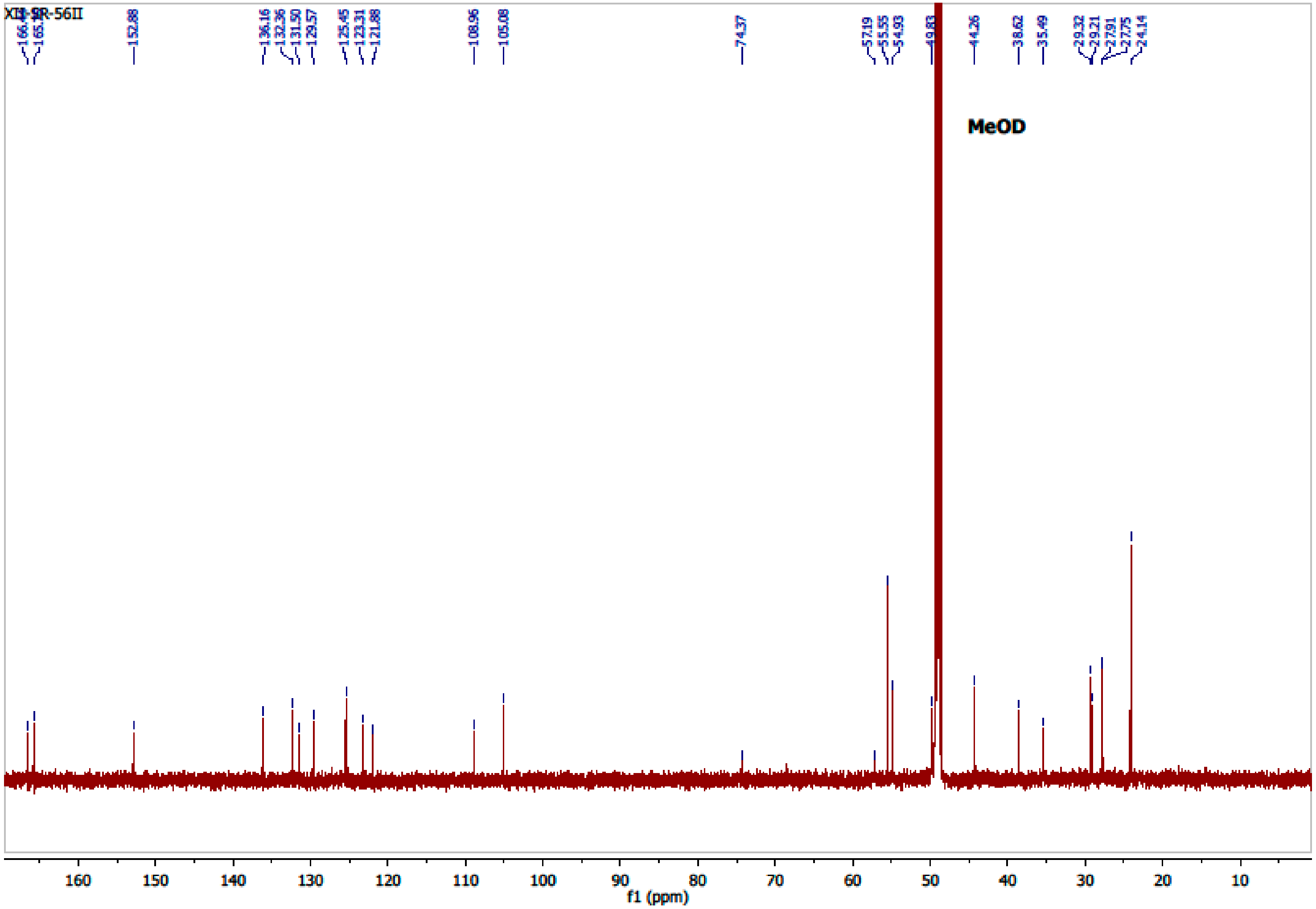

Figure S242. ^{13}C NMR spectrum of **61**.

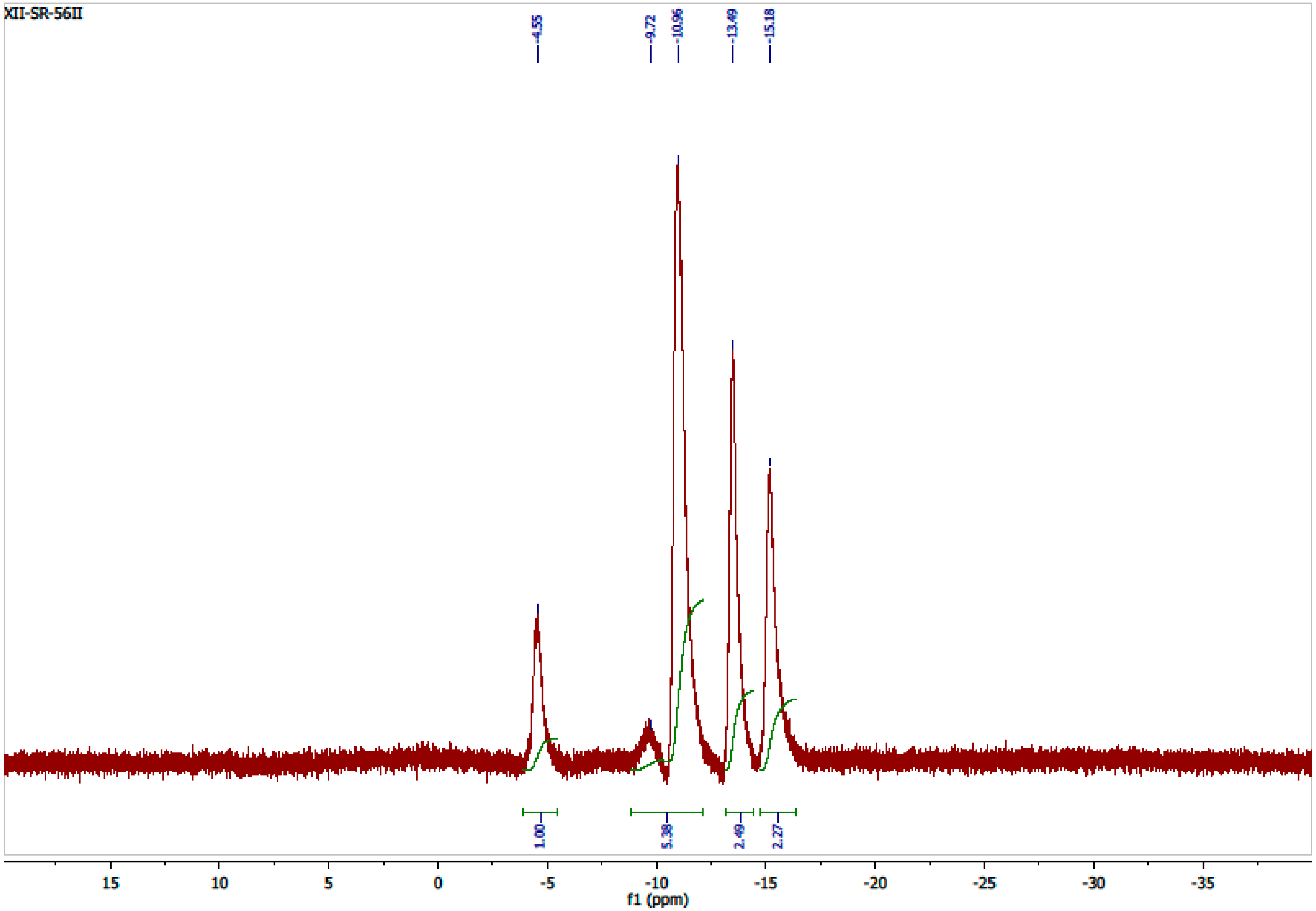

Figure S243. ^{11}B NMR spectrum of **61**.

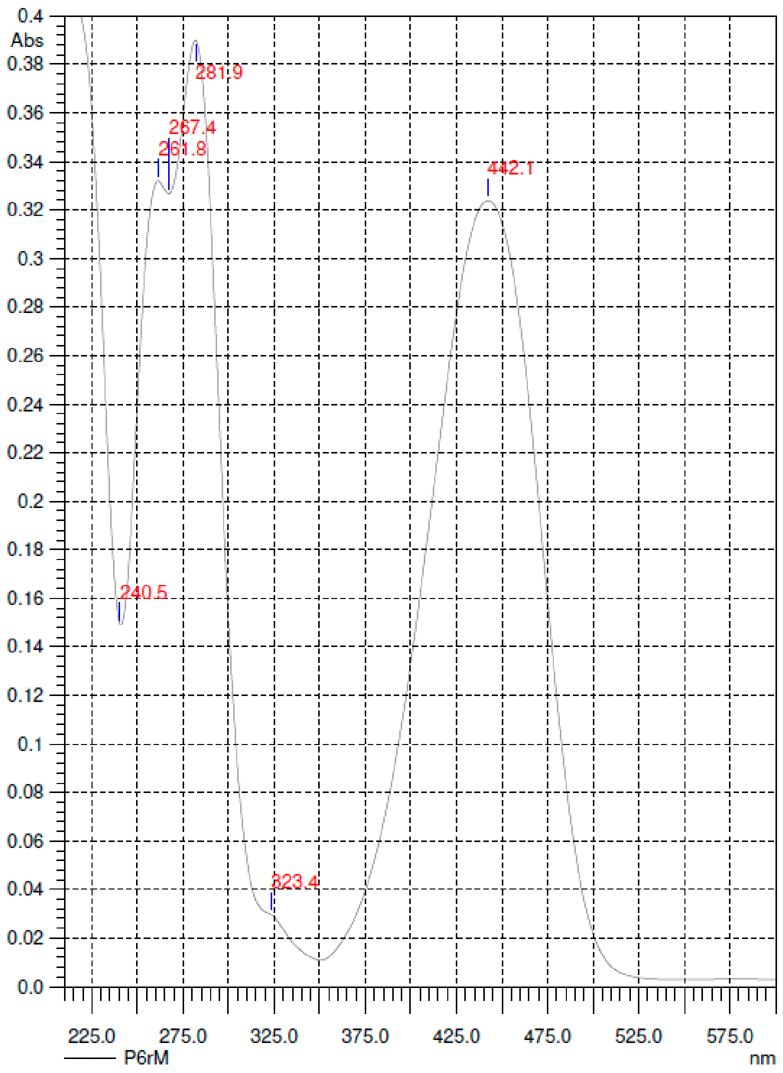

Figure S244. UV spectrum of **61.**

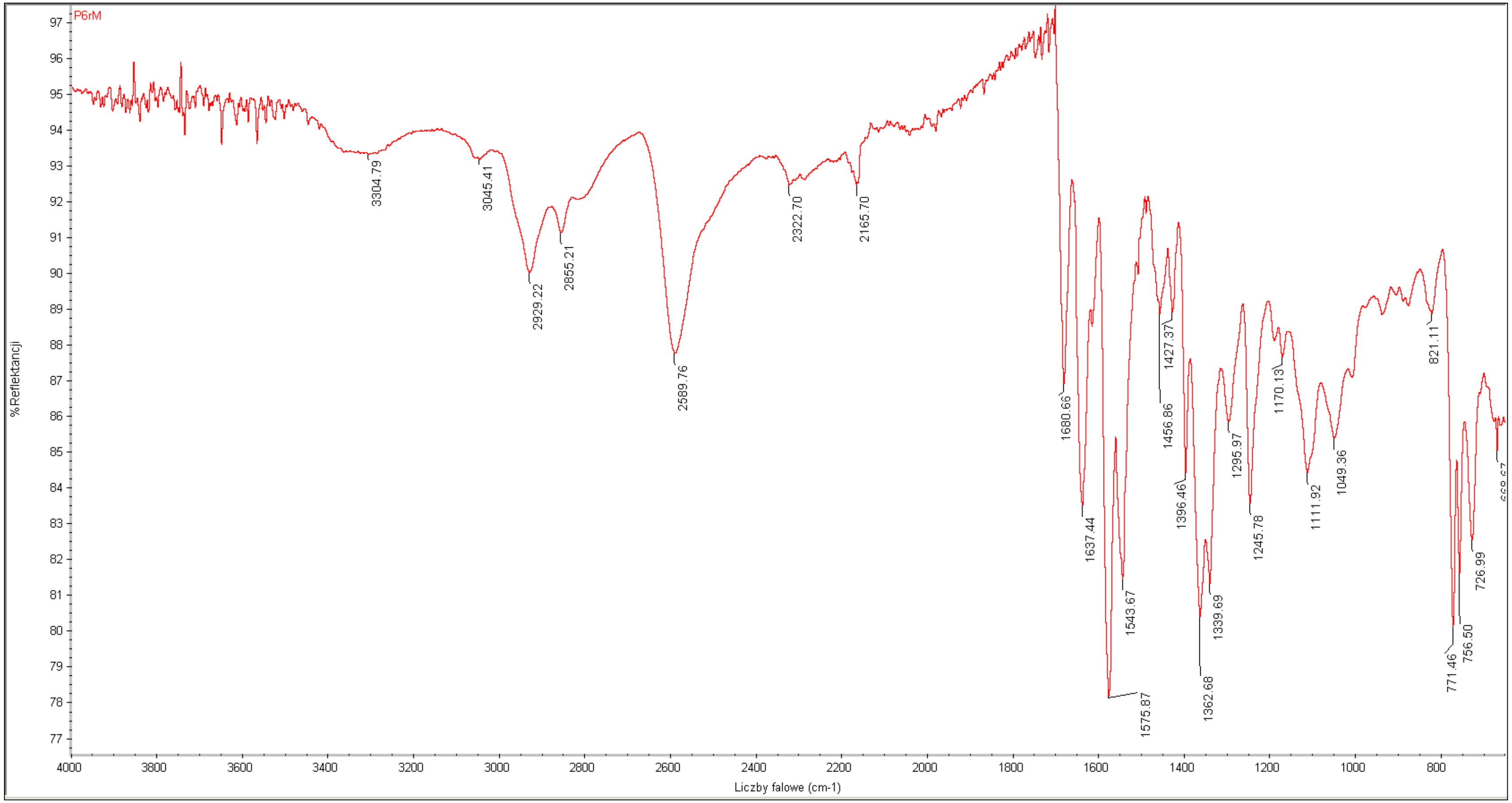

Figure S245. IR spectrum of **61**.

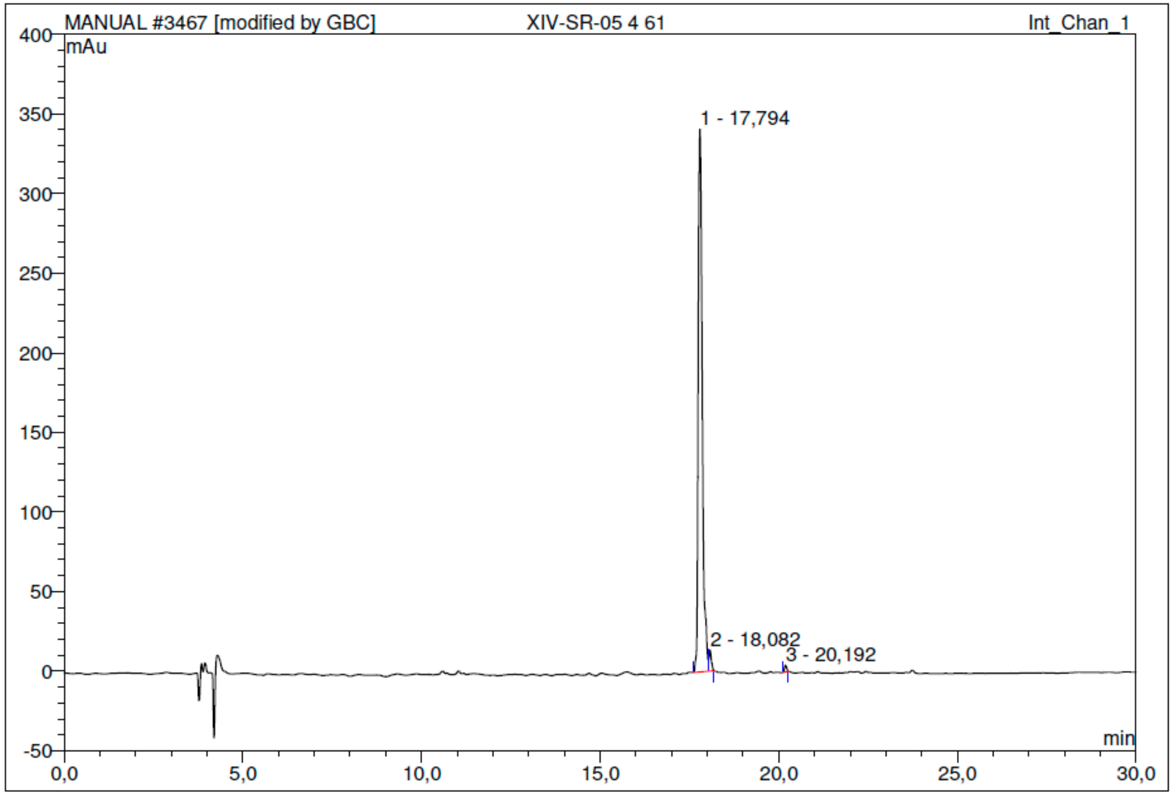

No.	Ret.Time min	Peak Name	Height mAu	Area mAu*min	Rel.Area %	Amount	Type
1	17,79	n.a.	340,970	43,408	96,88	n.a.	BM *
2	18,08	n.a.	13,151	1,104	2,46	n.a.	MB*
3	20,19	n.a.	4,250	0,296	0,66	n.a.	BMB*
Total:			358,371	44,808	100,00	0,000	

Figure S246. HPLC chromatogram of **61**.

Spectrum Name: XIV-SR-P6M_pt
Start Ion: 100
End Ion: 700
Source: APCI + 10.0µA 400C
Capillary: 150V 300C Offset: 25V Span: 0V

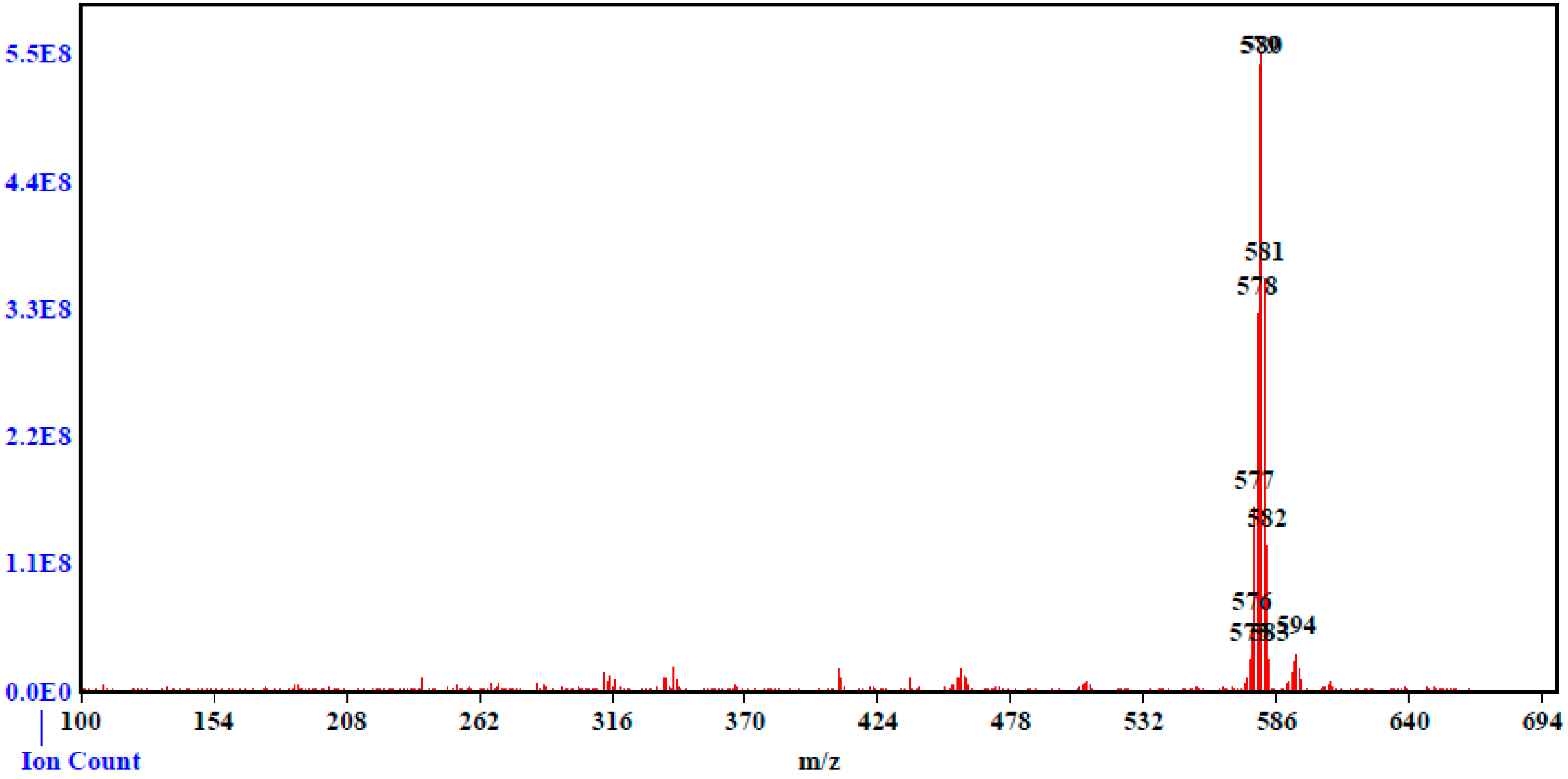

Figure S247. MS spectrum of **61**.

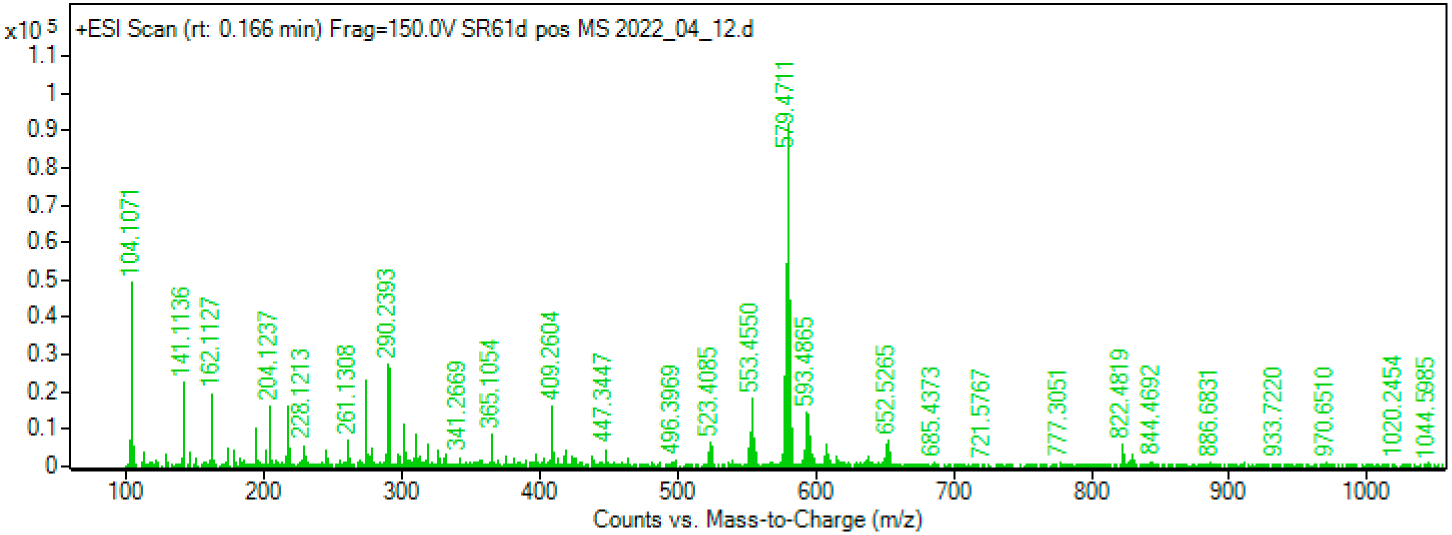

Figure S248. MS spectrum of **61**.

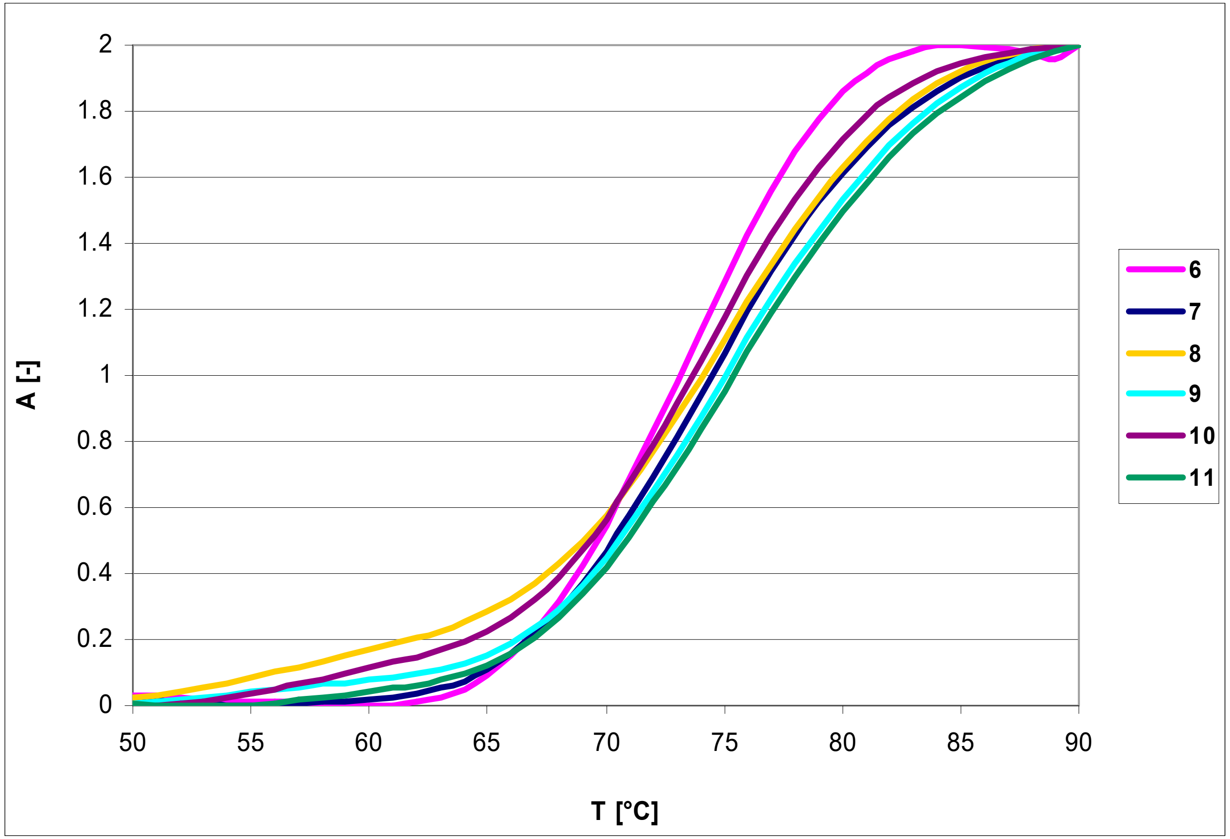

Figure S249. Melting curves of ct-DNA upon addition of **6-11** (c (ct-DNA) = 2 × 10^{-5} mol dm^{-3}) at molar ratio r = 0.3 (r = [compound]/[ct-DNA]), sodium cacodylate buffer (pH 7.0, 20 mM).

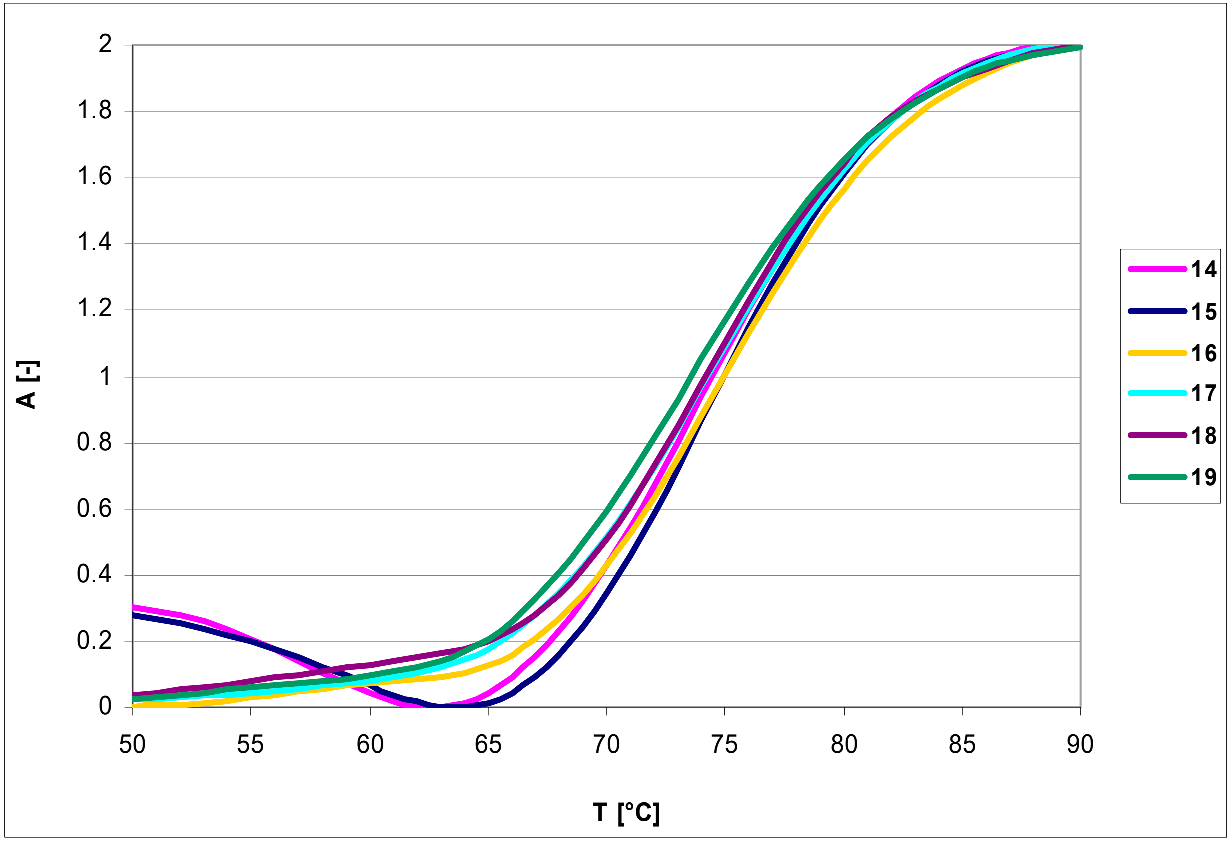

Figure S250. Melting curves of ct-DNA upon addition of **14-19** (c (ct-DNA) = 2 × 10^{-5} mol dm^{-3}) at molar ratio r = 0.3 (r = [compound]/[ct-DNA]), sodium cacodylate buffer (pH 7.0, 20 mM).

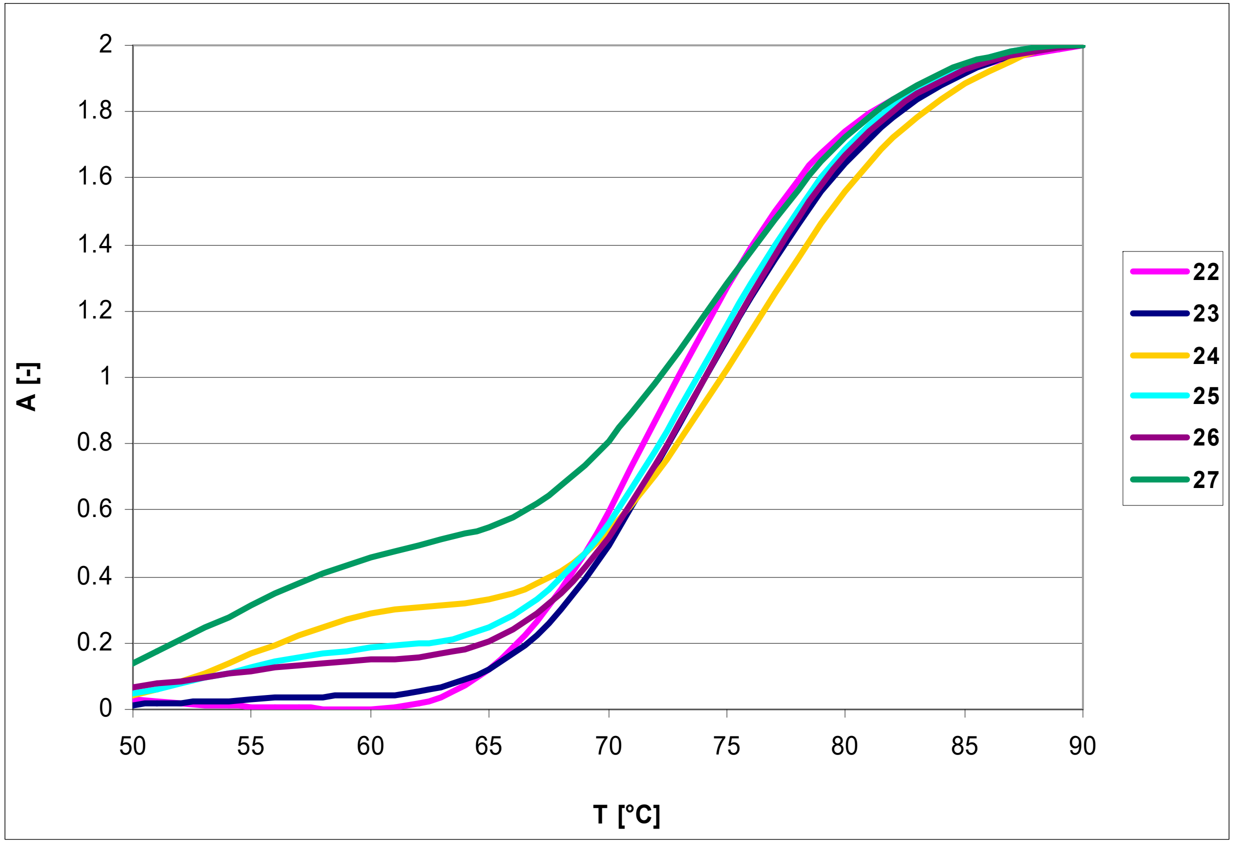

Figure S251. Melting curves of ct-DNA upon addition of **22-27** (c (ct-DNA) = 2×10^{-5} mol dm^{-3}) at molar ratio r = 0.3 (r = [compound]/[ct-DNA]), sodium cacodylate buffer (pH 7.0, 20 mM).

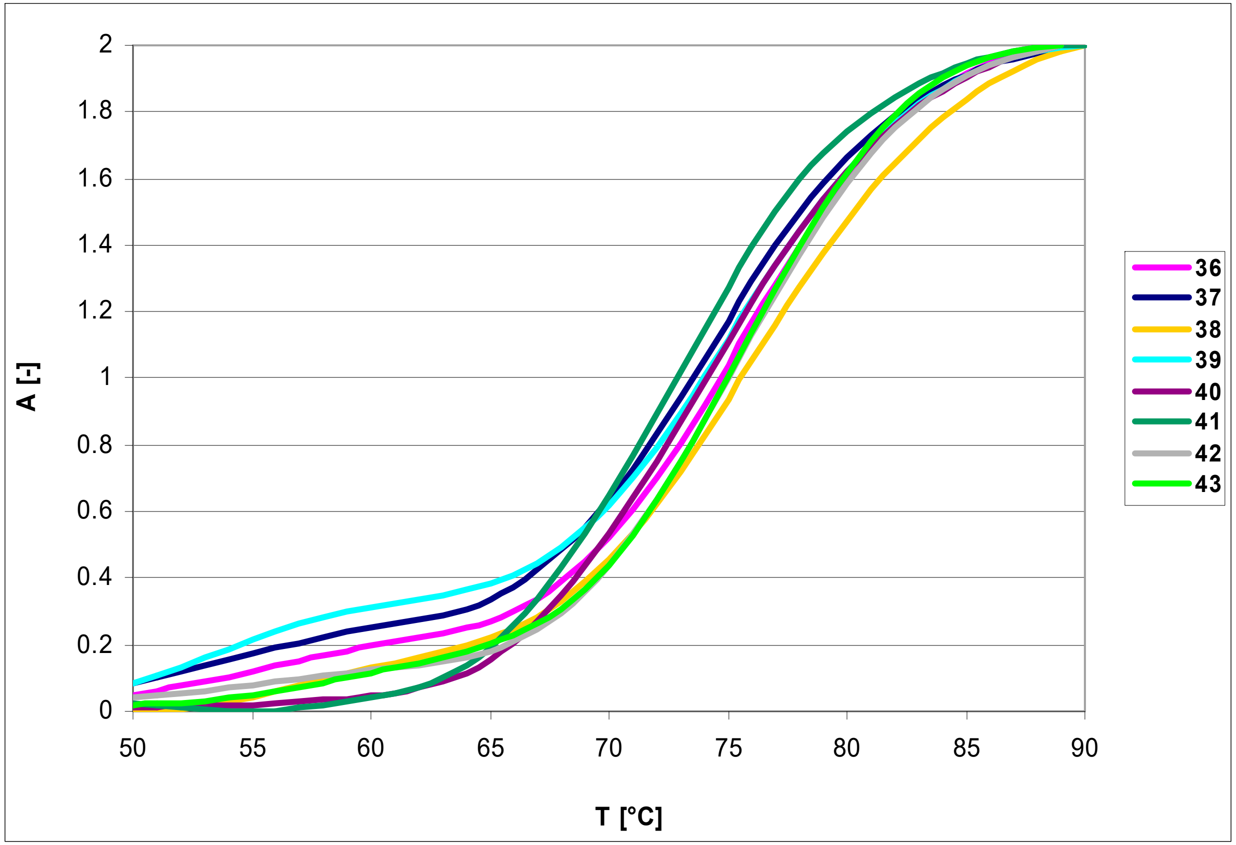

Figure S252. Melting curves of ct-DNA upon addition of **36-43** (c (ct-DNA) = 2 × 10^{-5} mol dm^{-3}) at molar ratio r = 0.3 (r = [compound]/[ct-DNA]), sodium cacodylate buffer (pH 7.0, 20 mM).

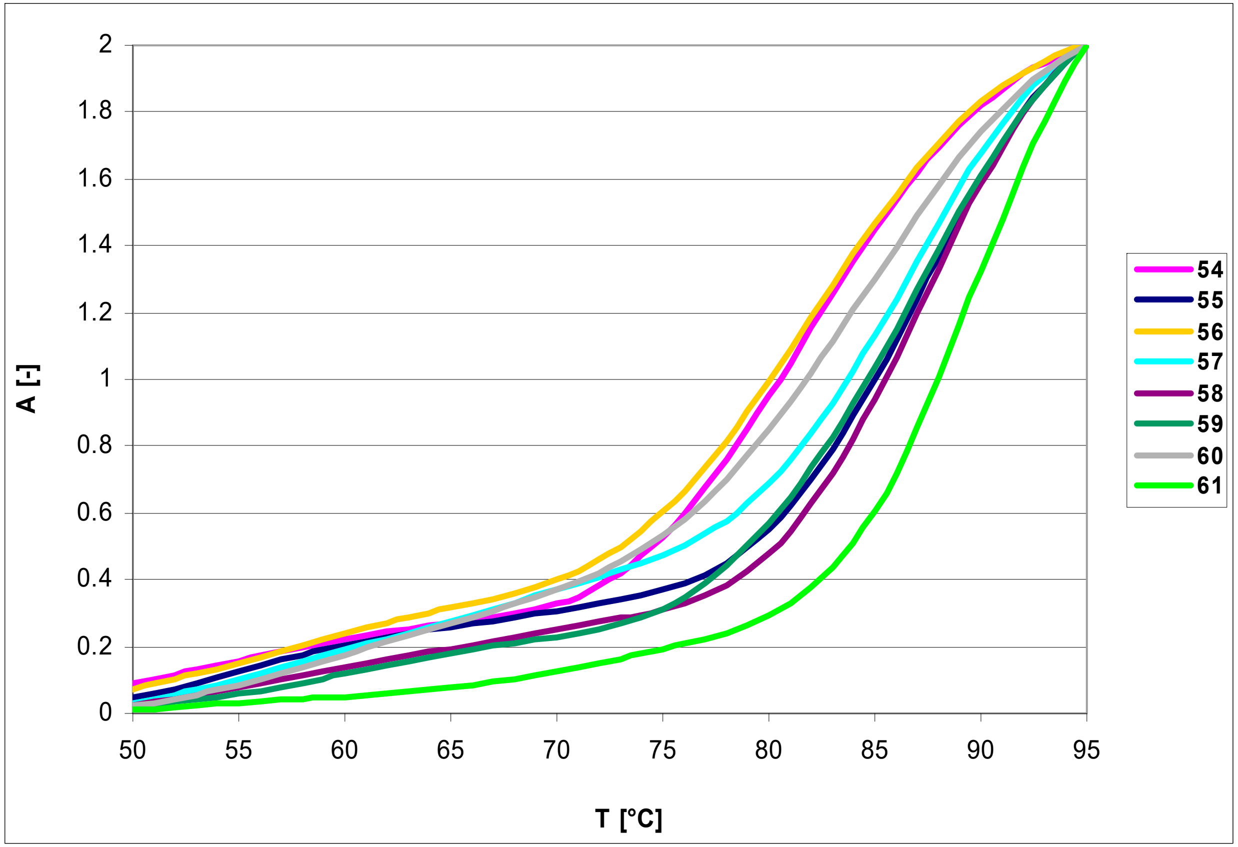

Figure S253. Melting curves of ct-DNA upon addition of **54-61** (c (ct-DNA) = 2 × 10^{-5} mol dm^{-3}) at molar ratio r = 0.3 (r = [compound]/[ct-DNA]), sodium cacodylate buffer (pH 7.0, 20 mM).

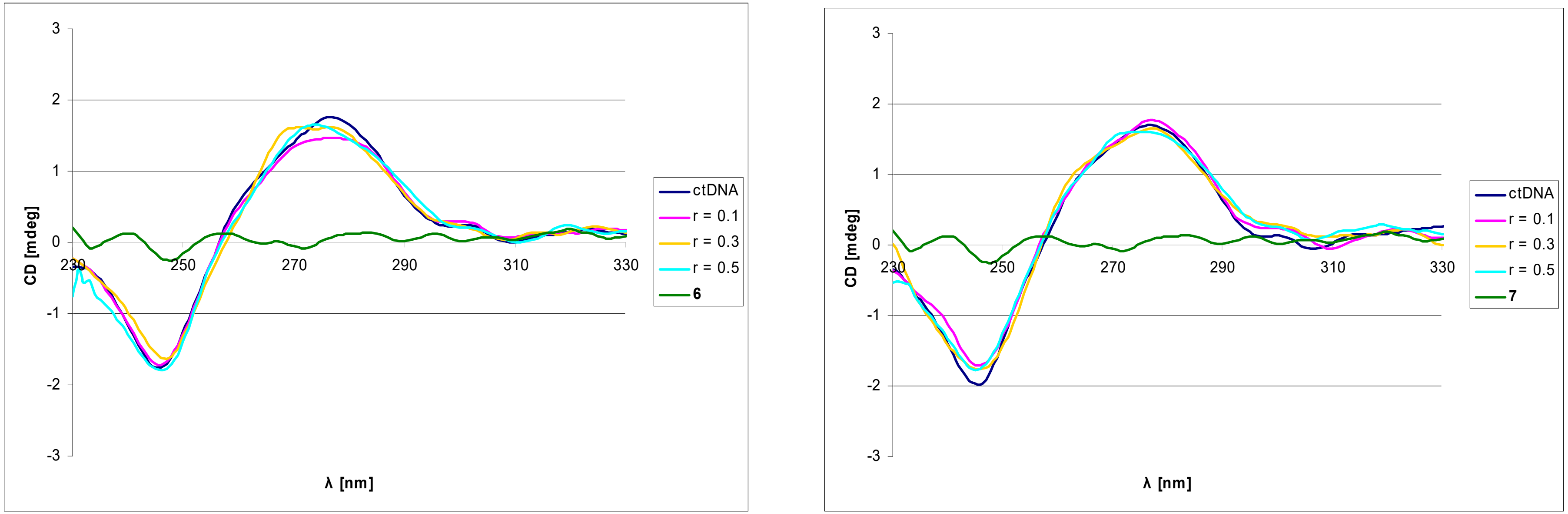

Figure S254. Changes in the CD spectrum of ct-DNA upon addition of **6** (c (ct-DNA) = 4 × 10^{-5} mol dm^{-3}) (left) and **7** (c (ct-DNA) = 4 × 10^{-5} mol dm^{-3}) (right) at different molar ratios r = [compound]/[ct-DNA], sodium cacodylate buffer (pH 7.0, 20 mM).

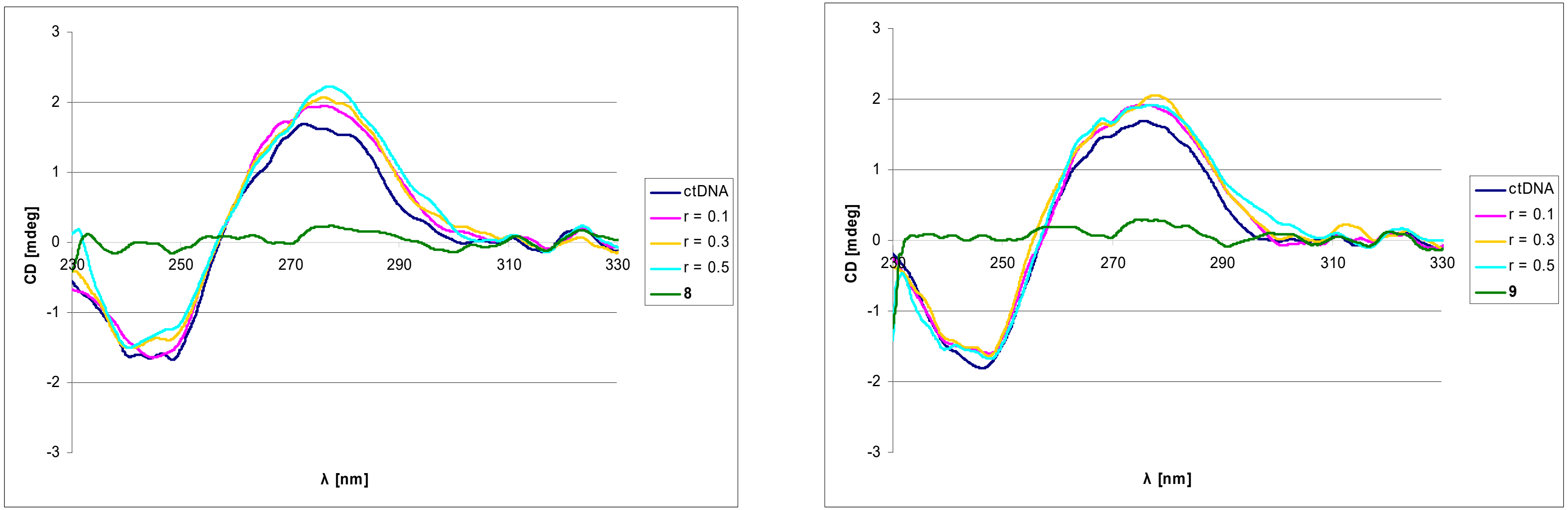

Figure S255. Changes in the CD spectrum of ct-DNA upon addition of **8** (c (ct-DNA) = 4 × 10^{-5} mol dm^{-3}) (left) and **9** (c (ct-DNA) = 4 × 10^{-5} mol dm^{-3}) (right) at different molar ratios r = [compound]/[ct-DNA], sodium cacodylate buffer (pH 7.0, 20 mM).

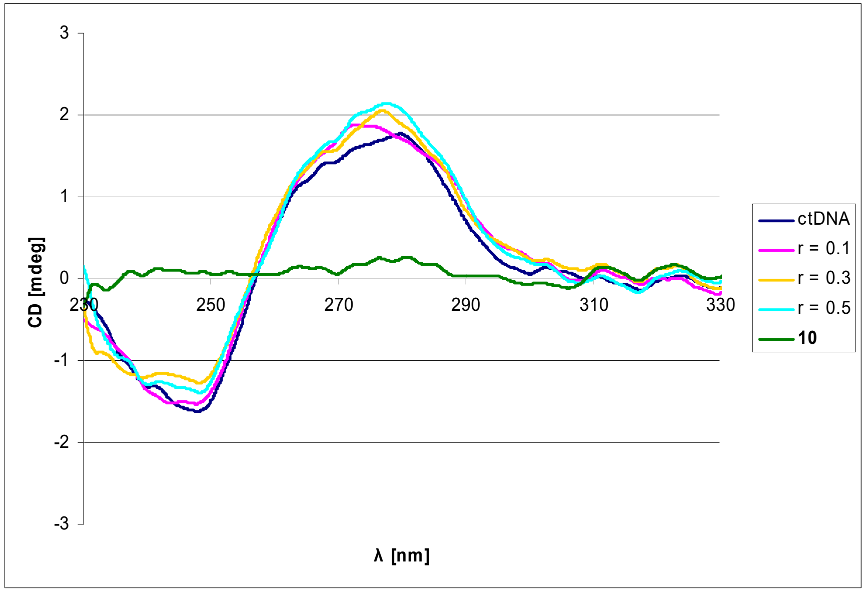

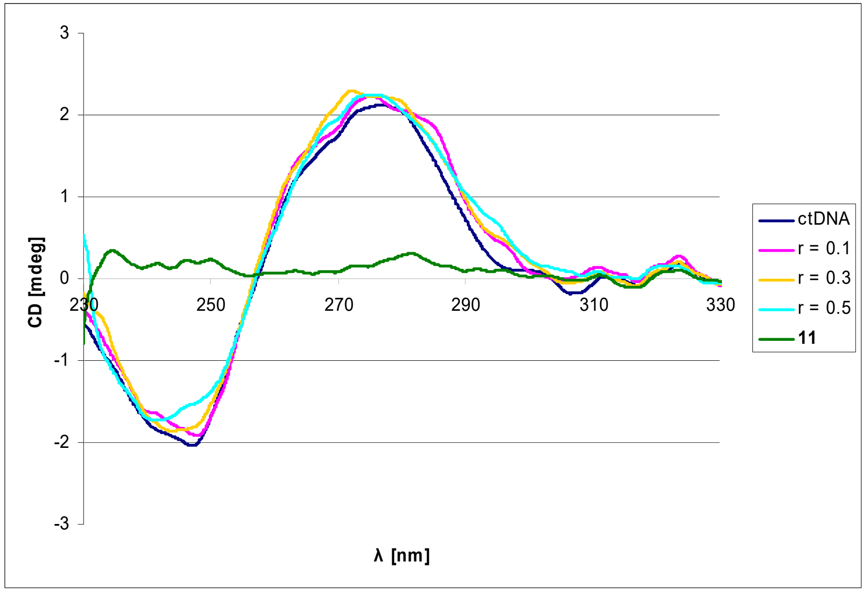

Figure S256. Changes in the CD spectrum of ct-DNA upon addition of **10** (c (ct-DNA) = 4×10^{-5} mol dm^{-3}) (left) and **11** (c (ct-DNA) = 4×10^{-5} mol dm^{-3}) (right) at different molar ratios r = [compound]/[ct-DNA], sodium cacodylate buffer (pH 7.0, 20 mM).

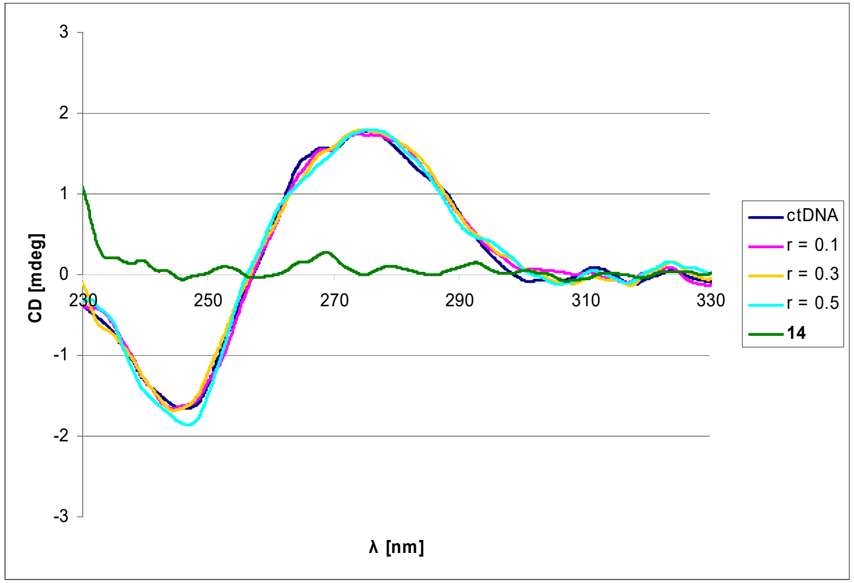

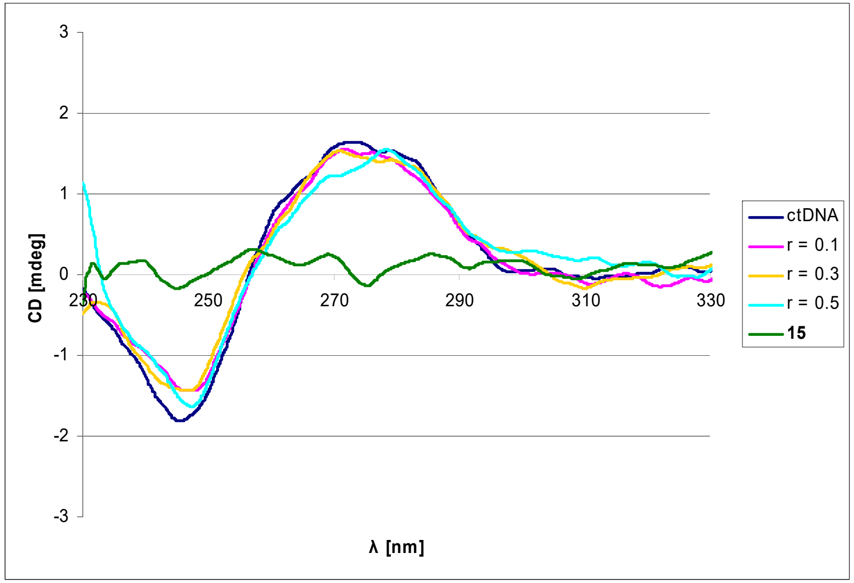

Figure S257. Changes in the CD spectrum of ct-DNA upon addition of **14** (c (ct-DNA) = 4 × 10^{-5} mol dm^{-3}) (left) and **15** (c (ct-DNA) = 4 × 10^{-5} mol dm^{-3}) (right) at different molar ratios r = [compound]/[ct-DNA], sodium cacodylate buffer (pH 7.0, 20 mM).

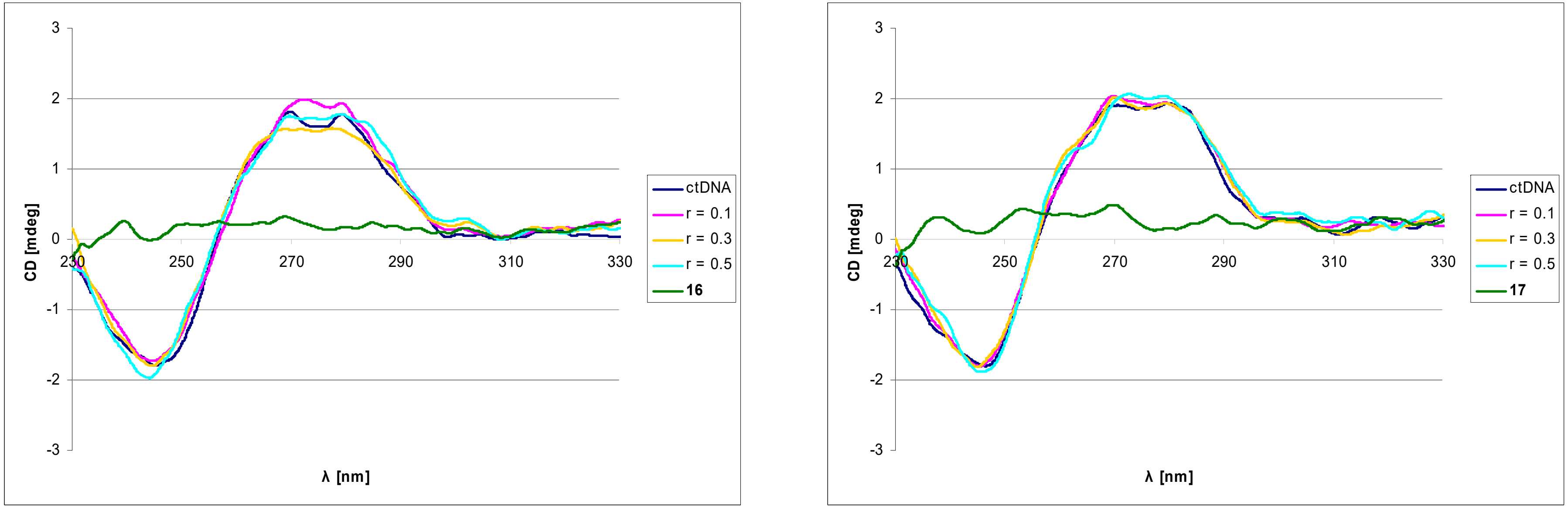

Figure S258. Changes in the CD spectrum of ct-DNA upon addition of **16** (c (ct-DNA) = 4 × 10^{-5} mol dm^{-3}) (left) and **17** (c (ct-DNA) = 4 × 10^{-5} mol dm^{-3}) (right) at different molar ratios r = [compound]/[ct-DNA], sodium cacodylate buffer (pH 7.0, 20 mM).

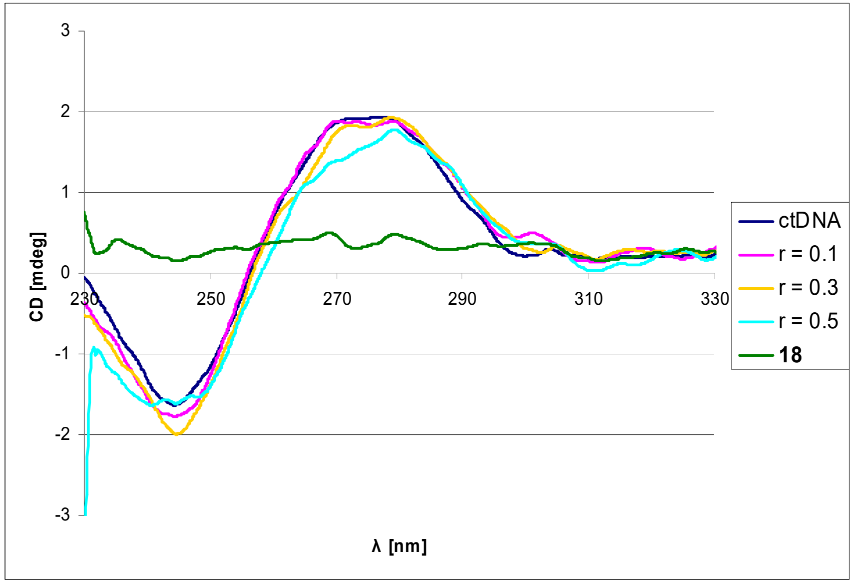

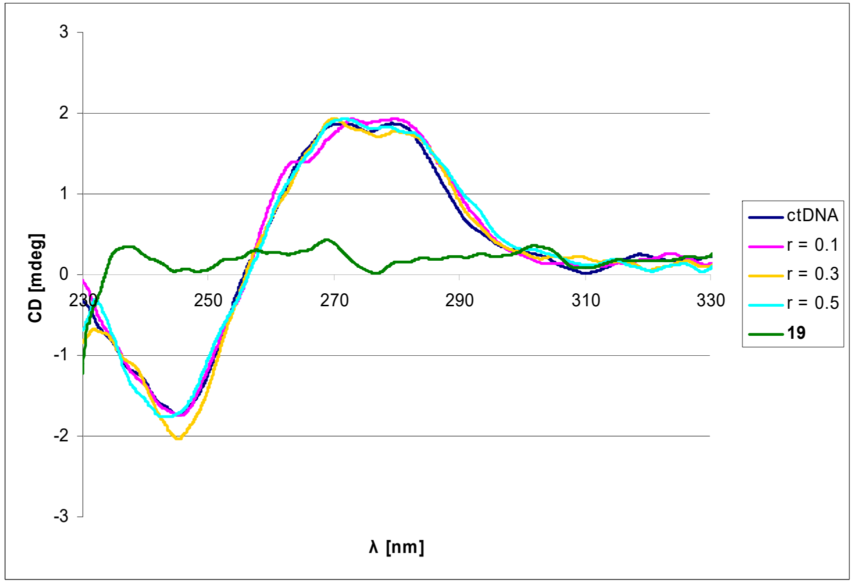

Figure S259. Changes in the CD spectrum of ct-DNA upon addition of **18** (c (ct-DNA) = 4 × 10^{-5} mol dm^{-3}) (left) and **19** (c (ct-DNA) = 4 × 10^{-5} mol dm^{-3}) (right) at different molar ratios r = [compound]/[ct-DNA], sodium cacodylate buffer (pH 7.0, 20 mM).

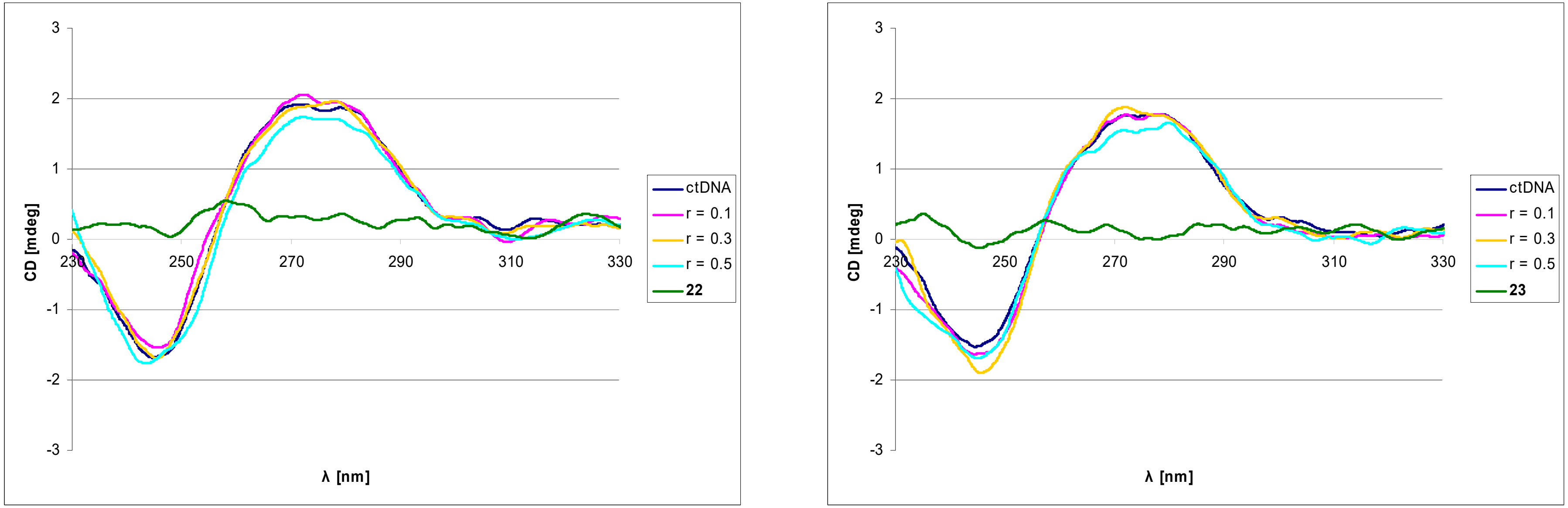

Figure S260. Changes in the CD spectrum of ct-DNA upon addition of **22** (c (ct-DNA) = 4 × 10^{-5} mol dm^{-3}) (left) and **23** (c (ct-DNA) = 4 × 10^{-5} mol dm^{-3}) (right) at different molar ratios r = [compound]/[ct-DNA], sodium cacodylate buffer (pH 7.0, 20 mM).

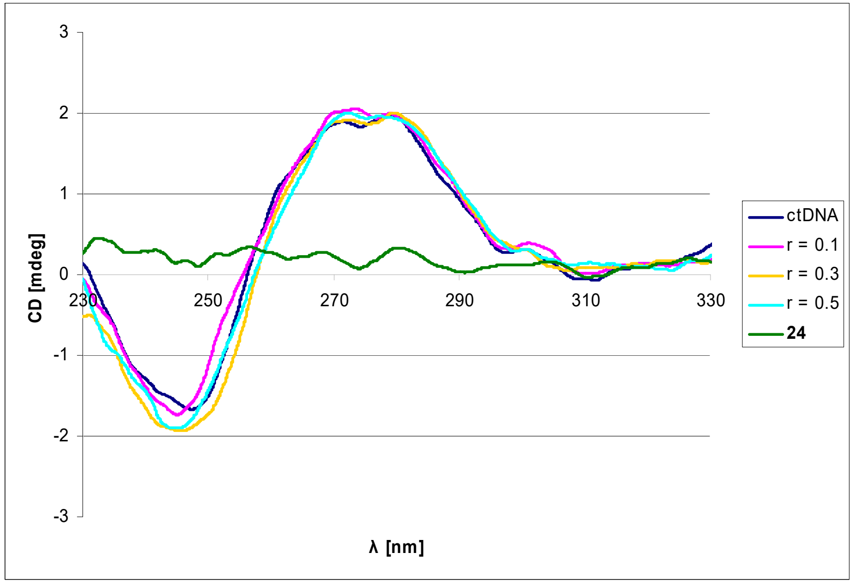

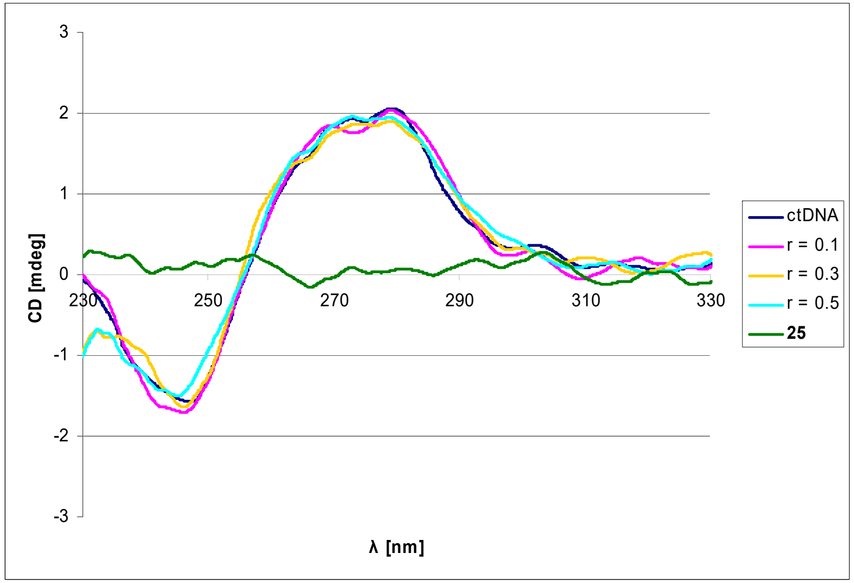

Figure S261. Changes in the CD spectrum of ct-DNA upon addition of **24** (c (ct-DNA) = 4×10^{-5} mol dm^{-3}) (left) and **25** (c (ct-DNA) = 4×10^{-5} mol dm^{-3}) (right) at different molar ratios r = [compound]/[ct-DNA], sodium cacodylate buffer (pH 7.0, 20 mM).

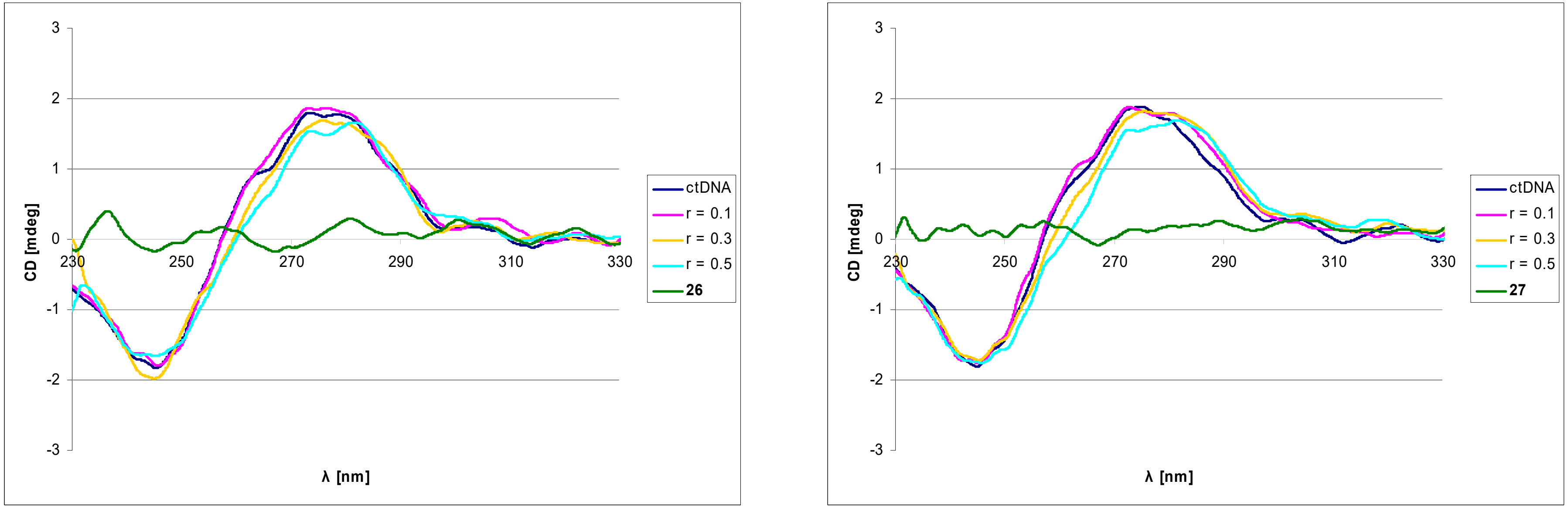

Figure S262. Changes in the CD spectrum of ct-DNA upon addition of **26** (c (ct-DNA) = 4 × 10^{-5} mol dm^{-3}) (left) and **27** (c (ct-DNA) = 4 × 10^{-5} mol dm^{-3}) (right) at different molar ratios r = [compound]/[ct-DNA], sodium cacodylate buffer (pH 7.0, 20 mM).

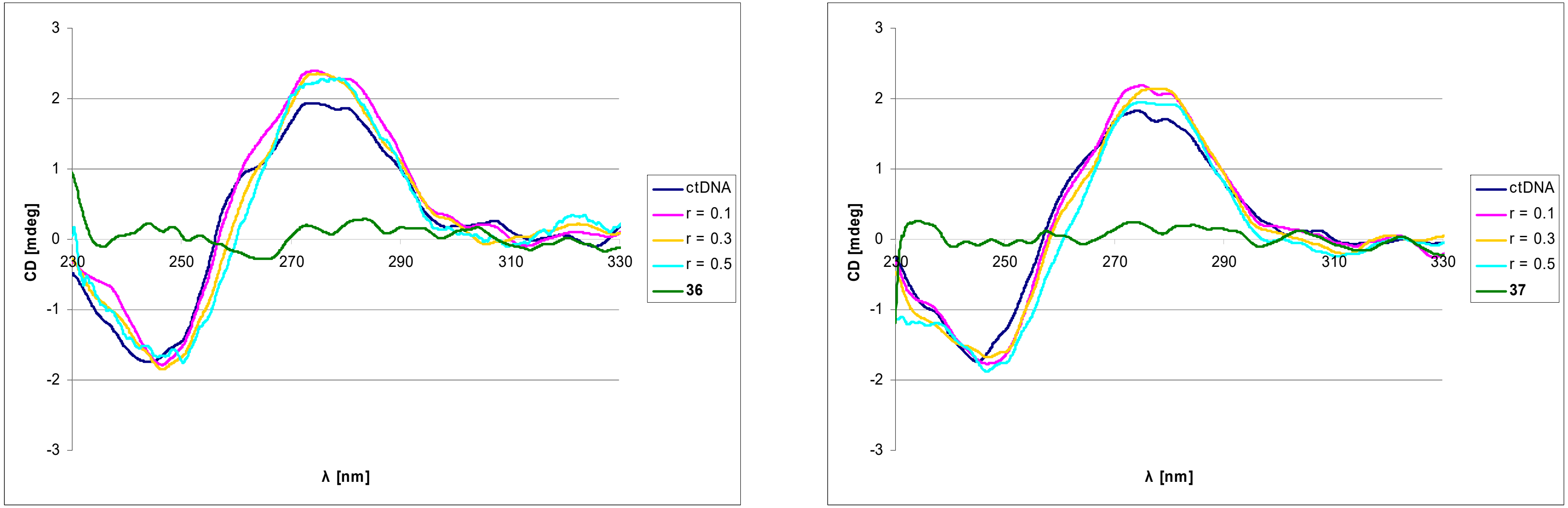

Figure S263. Changes in the CD spectrum of ct-DNA upon addition of **36** (c (ct-DNA) = 4 × 10^{-5} mol dm^{-3}) (left) and **37** (c (ct-DNA) = 4 × 10^{-5} mol dm^{-3}) (right) at different molar ratios r = [compound]/[ct-DNA], sodium cacodylate buffer (pH 7.0, 20 mM).

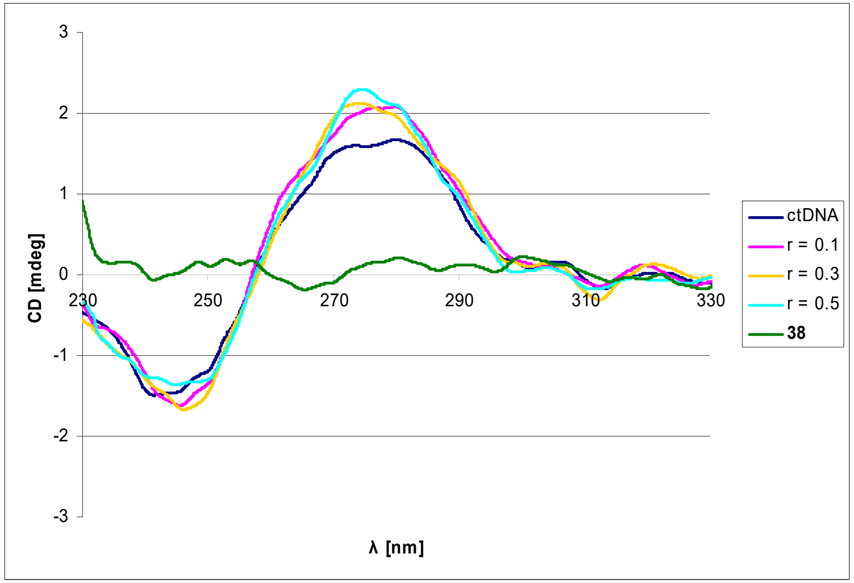

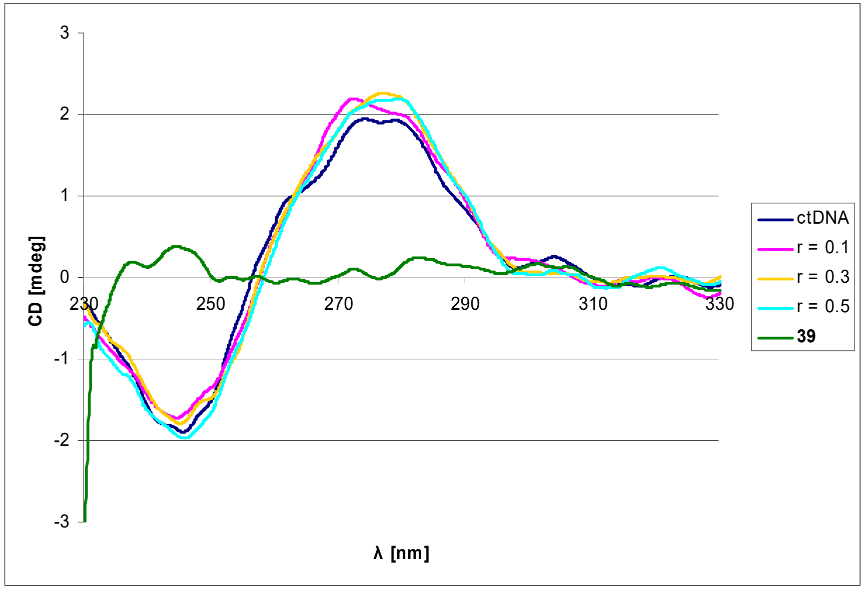

Figure S264. Changes in the CD spectrum of ct-DNA upon addition of **38** (c (ct-DNA) = 4 × 10^{-5} mol dm^{-3}) (left) and **39** (c (ct-DNA) = 4 × 10^{-5} mol dm^{-3}) (right) at different molar ratios r = [compound]/[ct-DNA], sodium cacodylate buffer (pH 7.0, 20 mM).

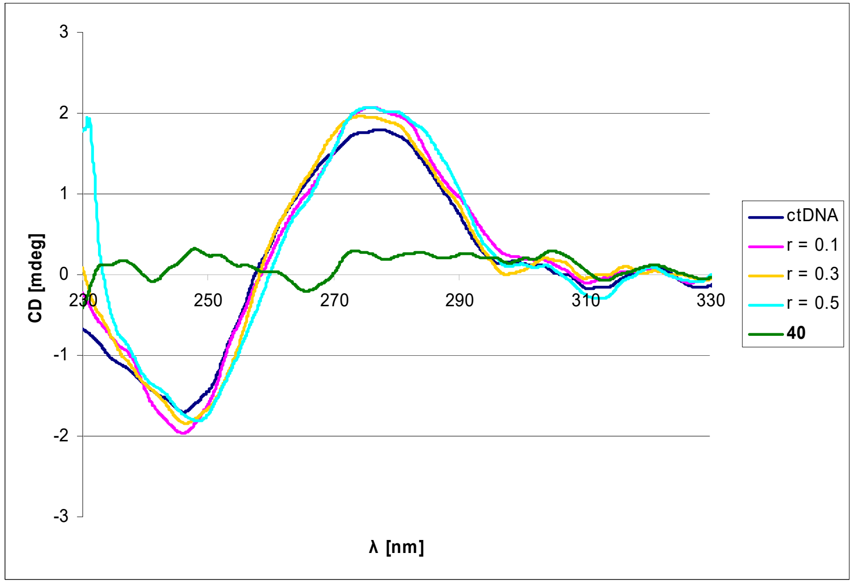

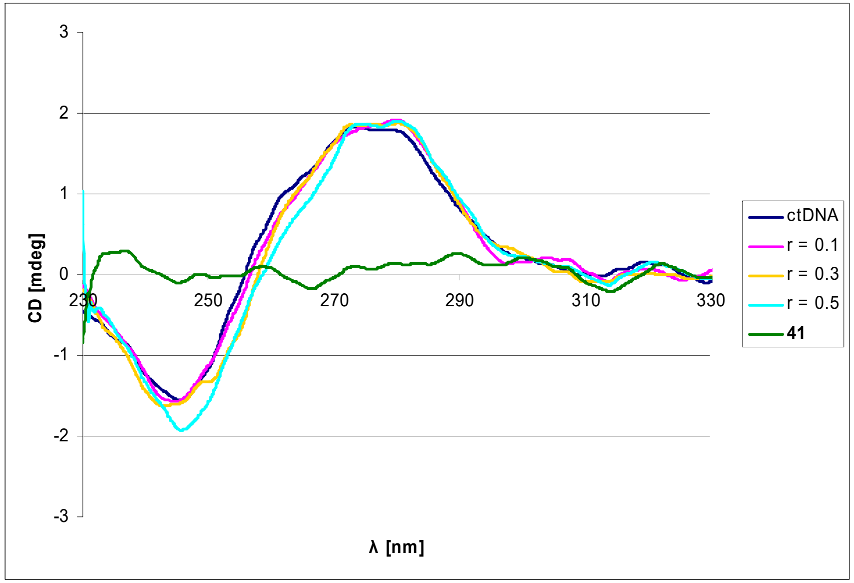

Figure S265. Changes in the CD spectrum of ct-DNA upon addition of **40** (c (ct-DNA) = 4×10^{-5} mol dm^{-3}) (left) and **41** (c (ct-DNA) = 4×10^{-5} mol dm^{-3}) (right) at different molar ratios r = [compound]/[ct-DNA], sodium cacodylate buffer (pH 7.0, 20 mM).

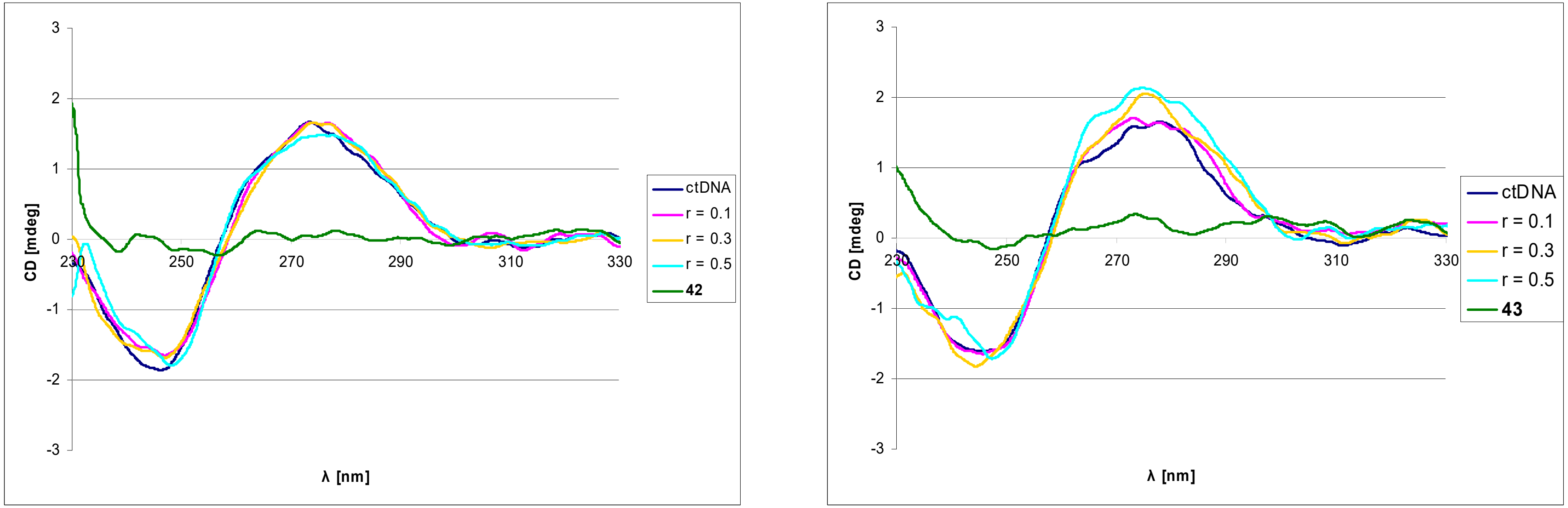

Figure S266. Changes in the CD spectrum of ct-DNA upon addition of **42** (c (ct-DNA) = 4 × 10^{-5} mol dm^{-3}) (left) and **43** (c (ct-DNA) = 4 × 10^{-5} mol dm^{-3}) (right) at different molar ratios r = [compound]/[ct-DNA], sodium cacodylate buffer (pH 7.0, 20 mM).

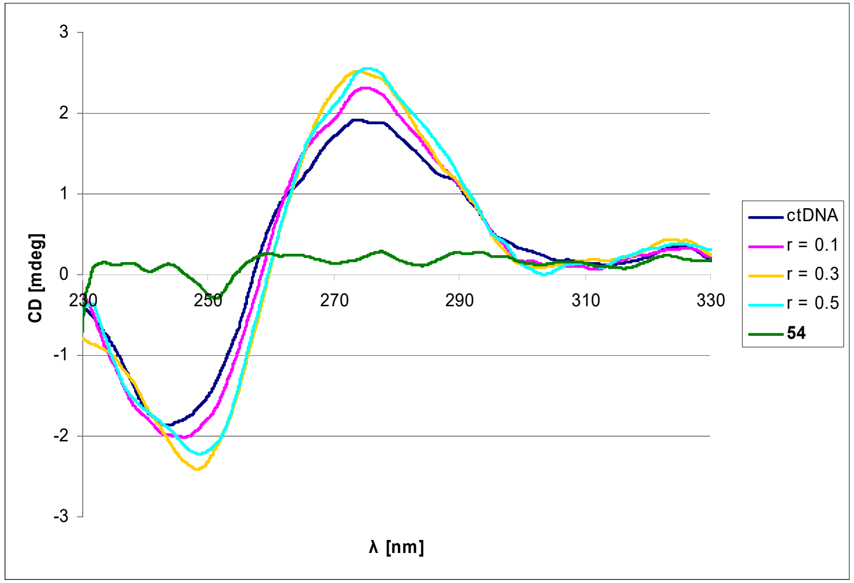

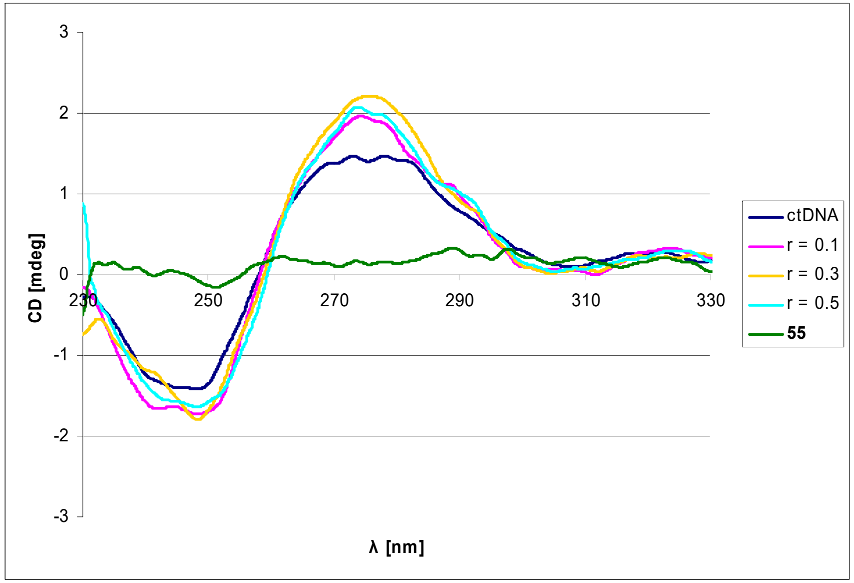

Figure S267. Changes in the CD spectrum of ct-DNA upon addition of **54** (c (ct-DNA) = 4 × 10^{-5} mol dm^{-3}) (left) and **55** (c (ct-DNA) = 4 × 10^{-5} mol dm^{-3}) (right) at different molar ratios r = [compound]/[ct-DNA], sodium cacodylate buffer (pH 7.0, 20 mM).

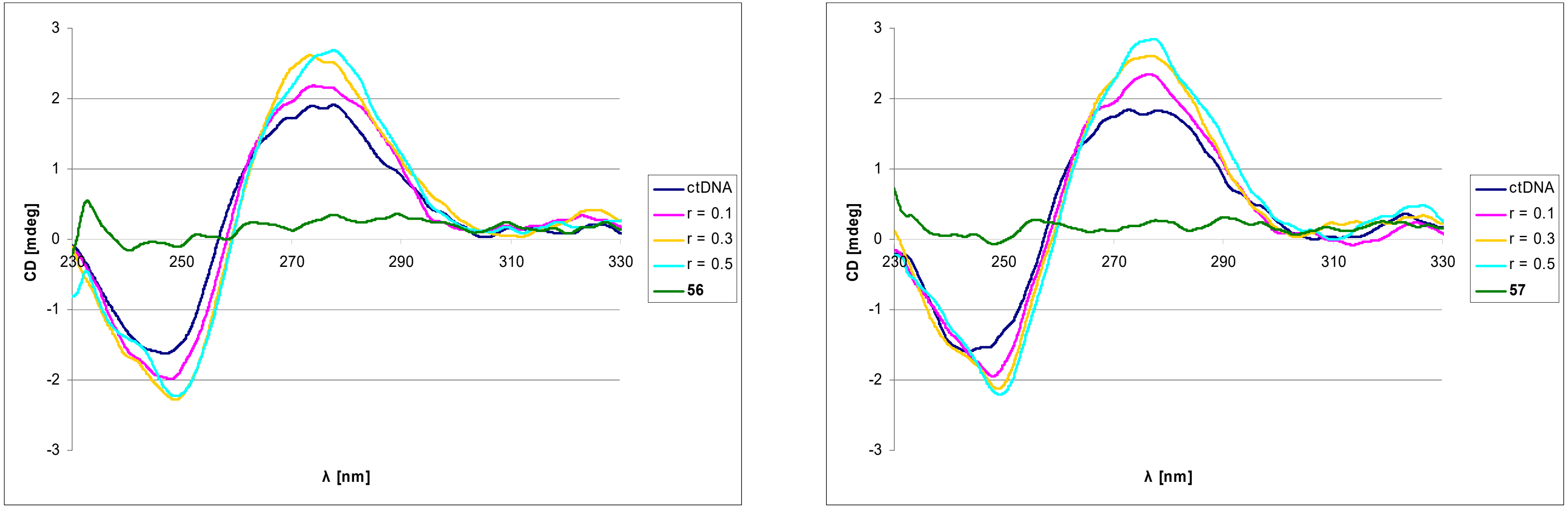

Figure S268. Changes in the CD spectrum of ct-DNA upon addition of **56** (c (ct-DNA) = 4 × 10^{-5} mol dm^{-3}) (left) and **57** (c (ct-DNA) = 4 × 10^{-5} mol dm^{-3}) (right) at different molar ratios r = [compound]/[ct-DNA], sodium cacodylate buffer (pH 7.0, 20 mM).

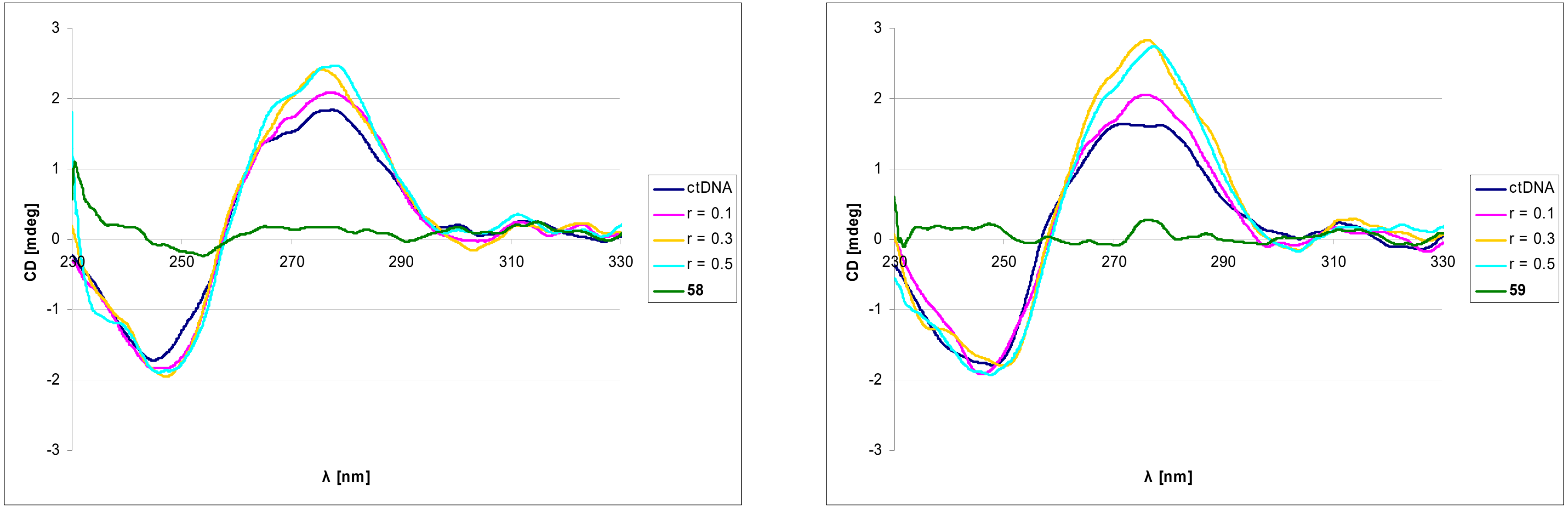

Figure S269. Changes in the CD spectrum of ct-DNA upon addition of **58** (c (ct-DNA) = 4 × 10^{-5} mol dm^{-3}) (left) and **59** (c (ct-DNA) = 4 × 10^{-5} mol dm^{-3}) (right) at different molar ratios r = [compound]/[ct-DNA], sodium cacodylate buffer (pH 7.0, 20 mM).

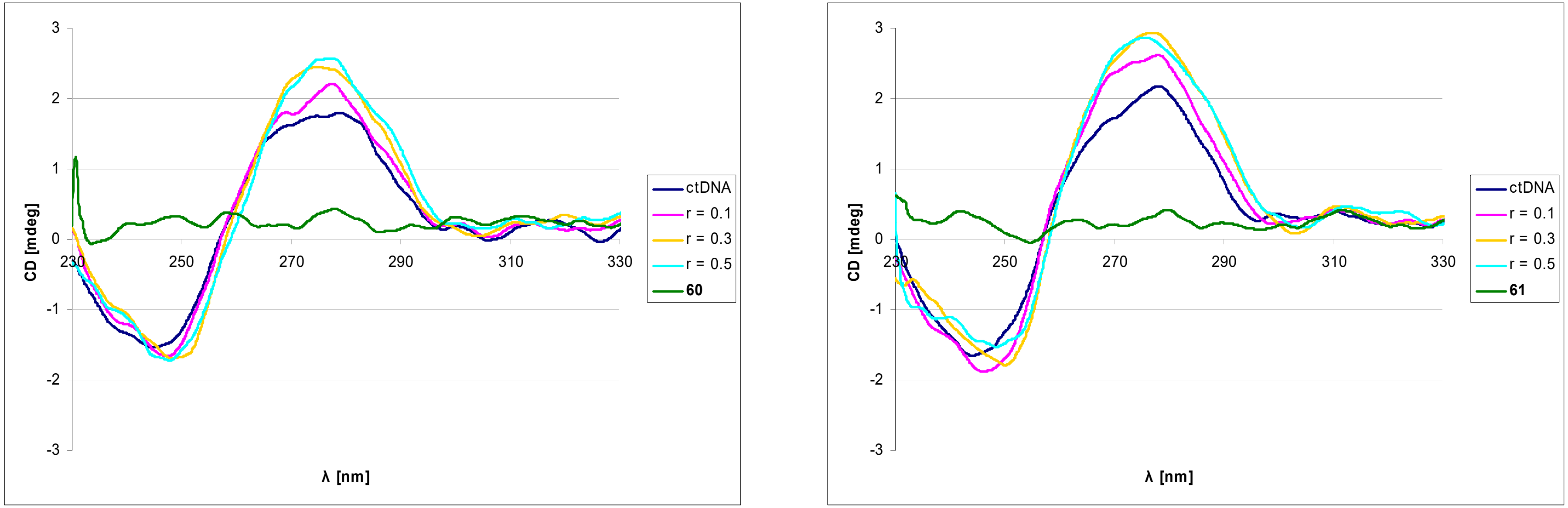

Figure S270. Changes in the CD spectrum of ct-DNA upon addition of **60** (c (ct-DNA) = 4 × 10^{-5} mol dm^{-3}) (left) and **61** (c (ct-DNA) = 4 × 10^{-5} mol dm^{-3}) (right) at different molar ratios r = [compound]/[ct-DNA], sodium cacodylate buffer (pH 7.0, 20 mM).

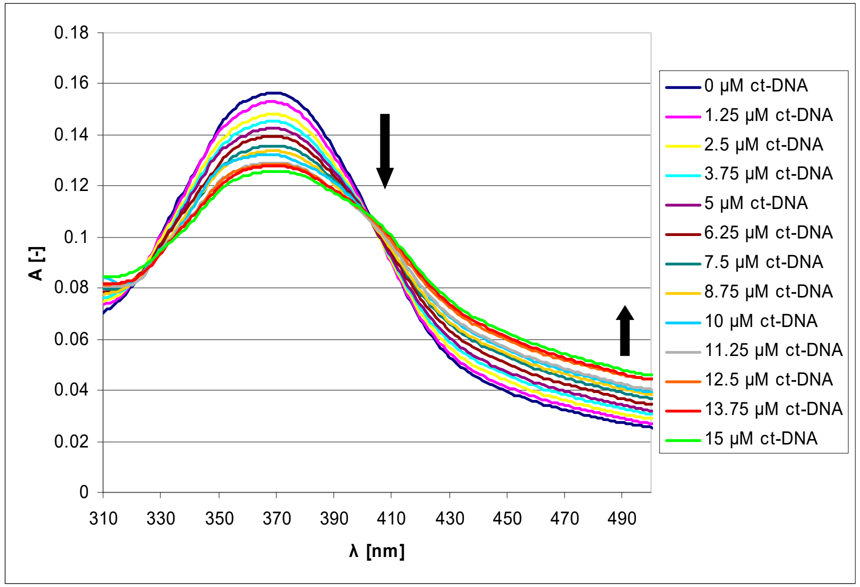

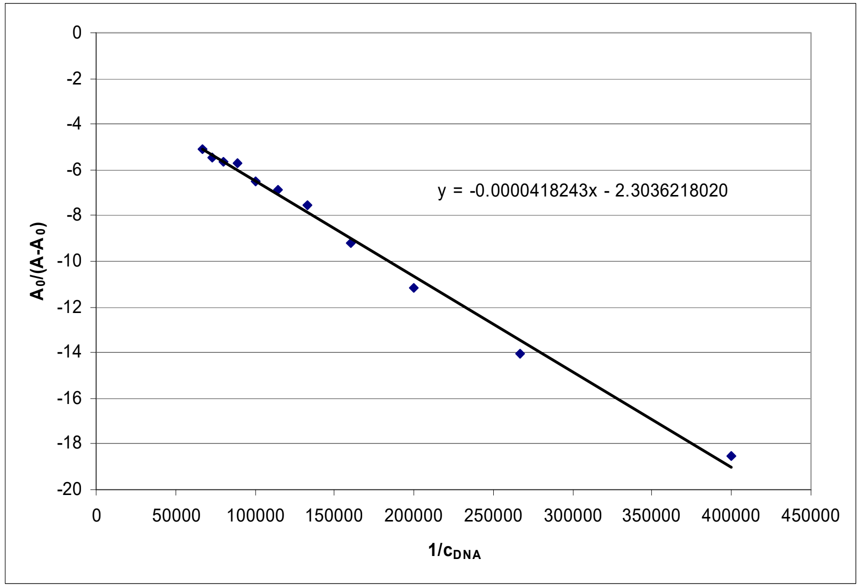

Figure S271. UV-vis absorption spectra of compound **6** (10 µM) in the presence of the increasing amount of cf-DNA (0-15 µM) (left). The plot of $A_0/A-A_0$ versus 1/[DNA] yielded the binding constant (right).

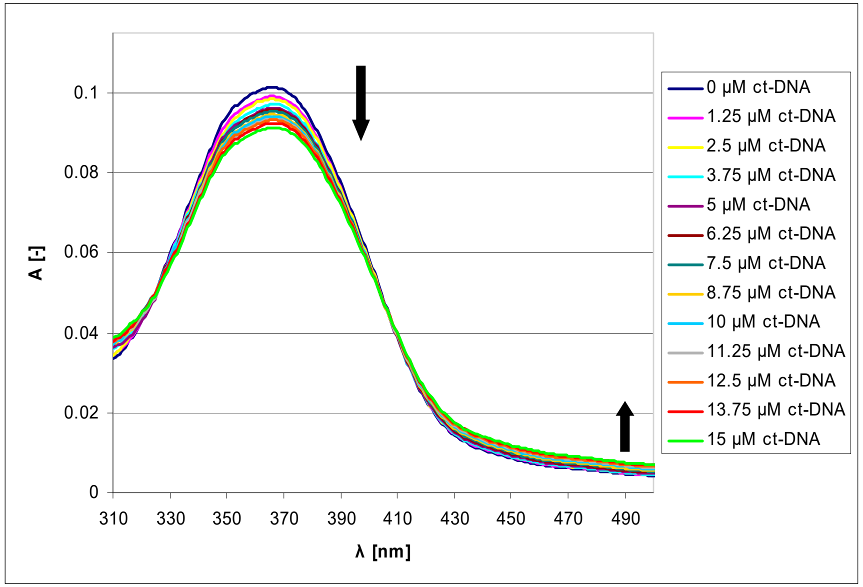

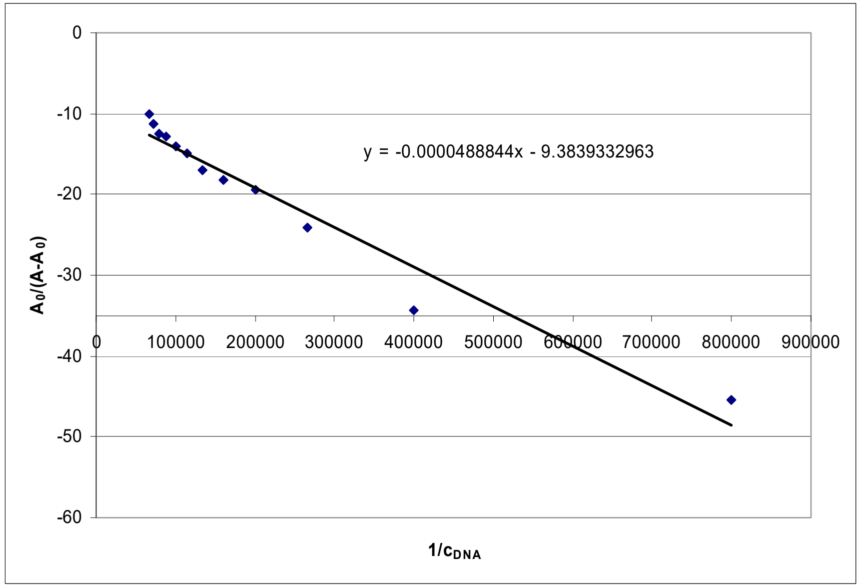

Figure S272. UV-vis absorption spectra of compound **7** (10 µM) in the presence of the increasing amount of cf-DNA (0-15 µM) (left). The plot of $A_0/A-A_0$ versus 1/[DNA] yielded the binding constant (right).

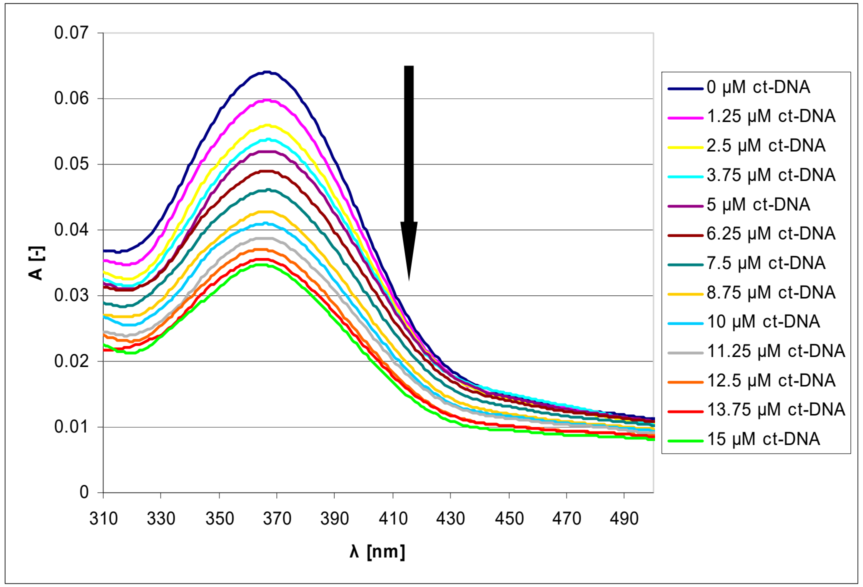

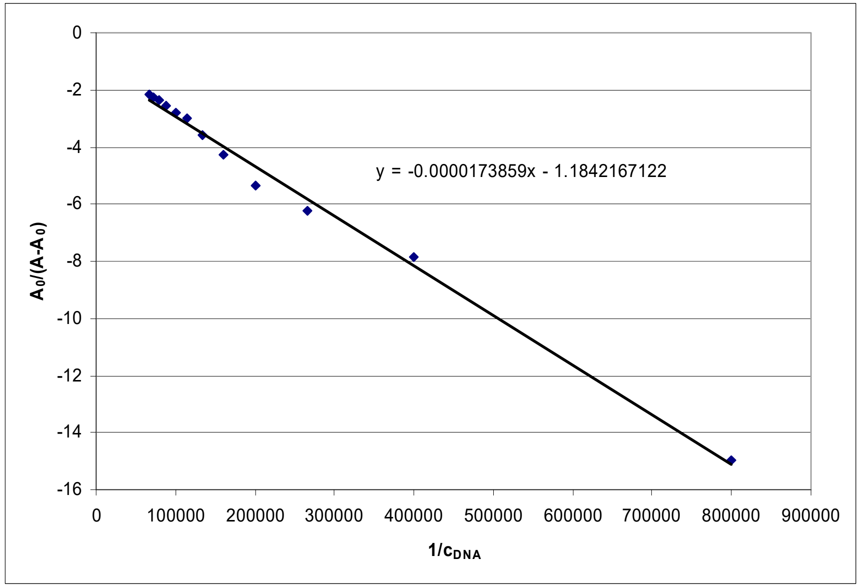

Figure S273. UV-vis absorption spectra of compound **8** (10 µM) in the presence of the increasing amount of cf-DNA (0-15 µM) (left). The plot of $A_0/A-A_0$ versus 1/[DNA] yielded the binding constant (right).

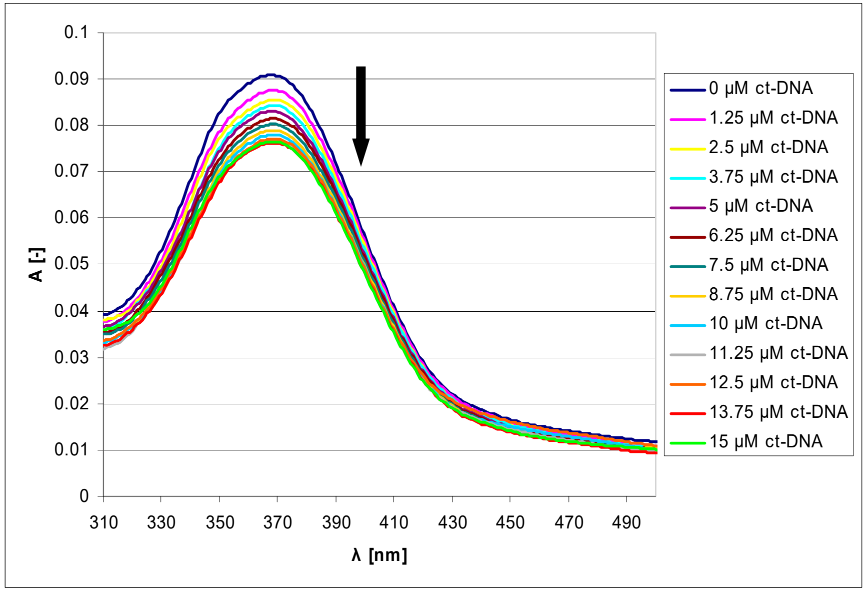

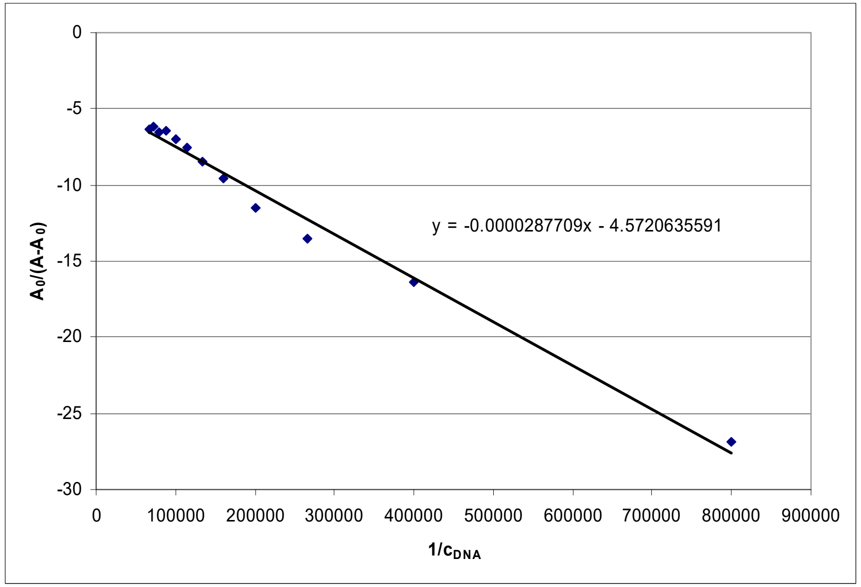

Figure S274. UV-vis absorption spectra of compound **9** (10 µM) in the presence of the increasing amount of cf-DNA (0-15 µM) (left). The plot of $A_0/A-A_0$ versus 1/[DNA] yielded the binding constant (right).

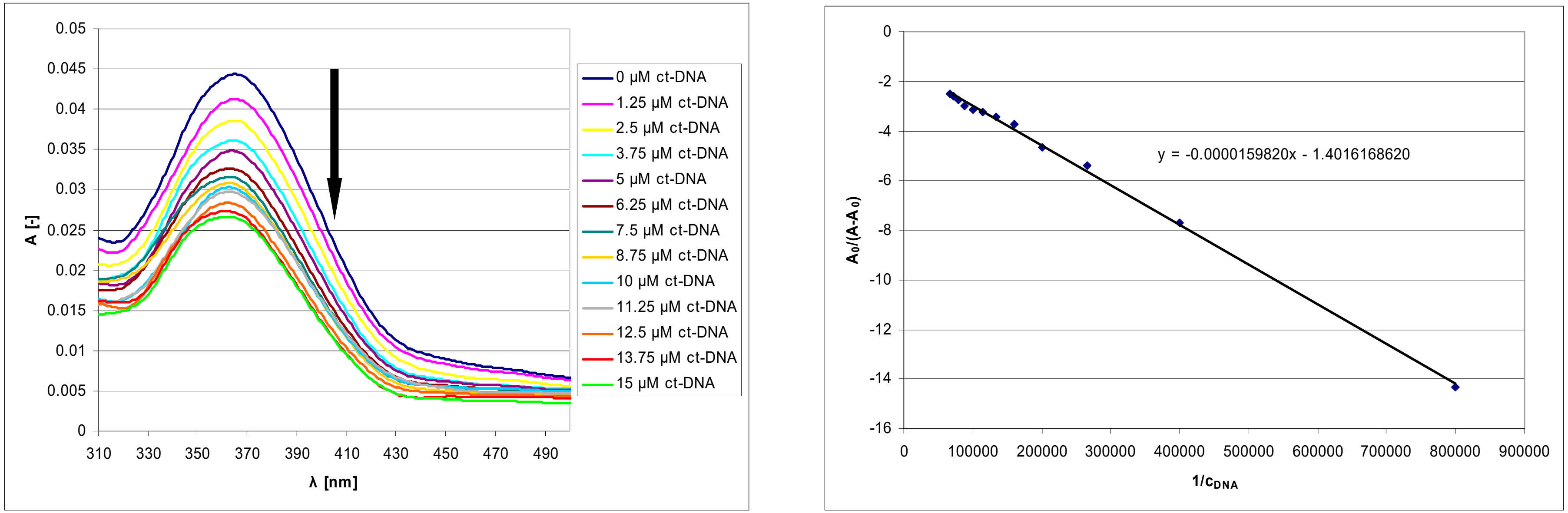

Figure S275. UV-vis absorption spectra of compound **10** (10 μM) in the presence of the increasing amount of cf-DNA (0-15 μM) (left). The plot of $A_0/A-A_0$ versus 1/[DNA] yielded the binding constant (right).

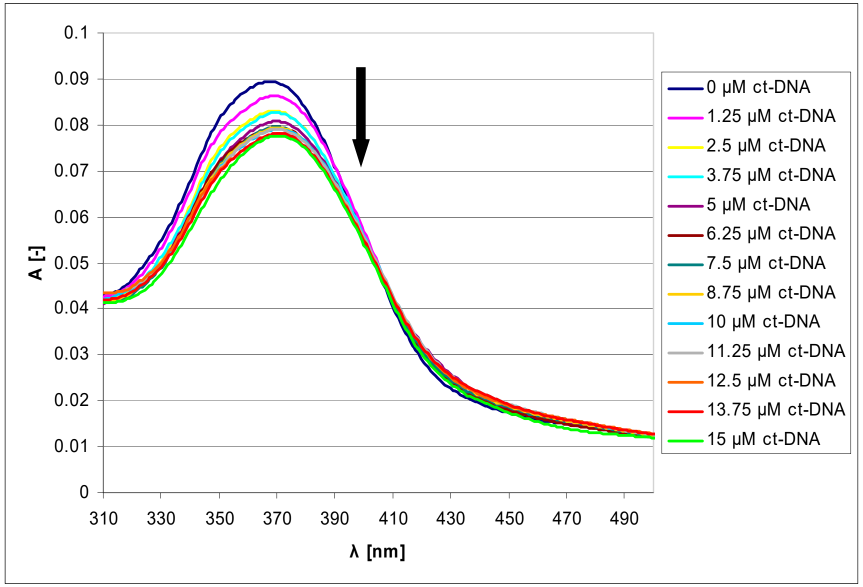

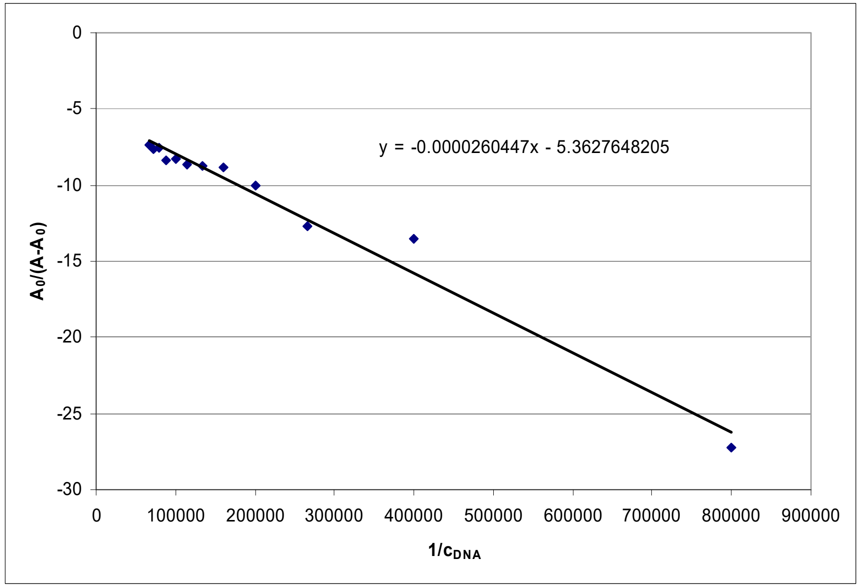

Figure S276. UV-vis absorption spectra of compound **11** (10 µM) in the presence of the increasing amount of cf-DNA (0-15 µM) (left). The plot of $A_0/A-A_0$ versus 1/[DNA] yielded the binding constant (right).

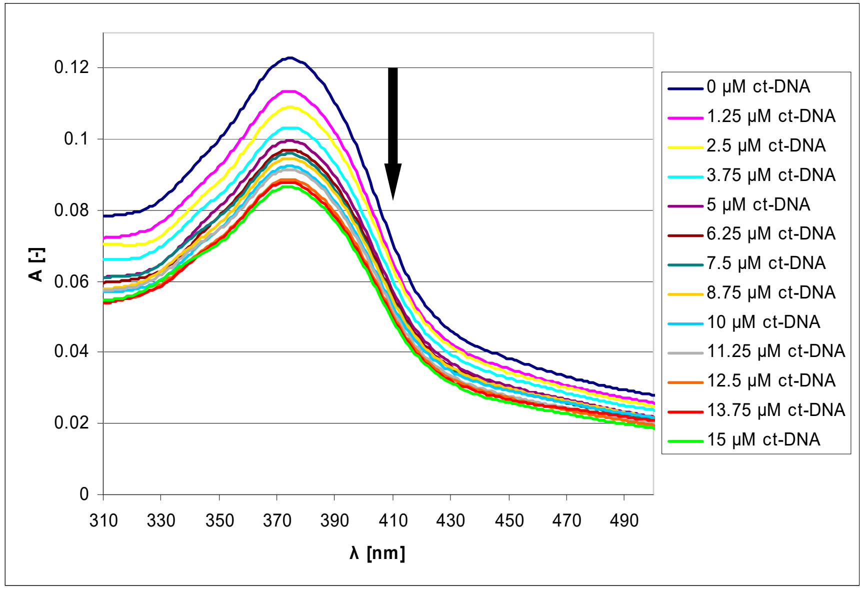

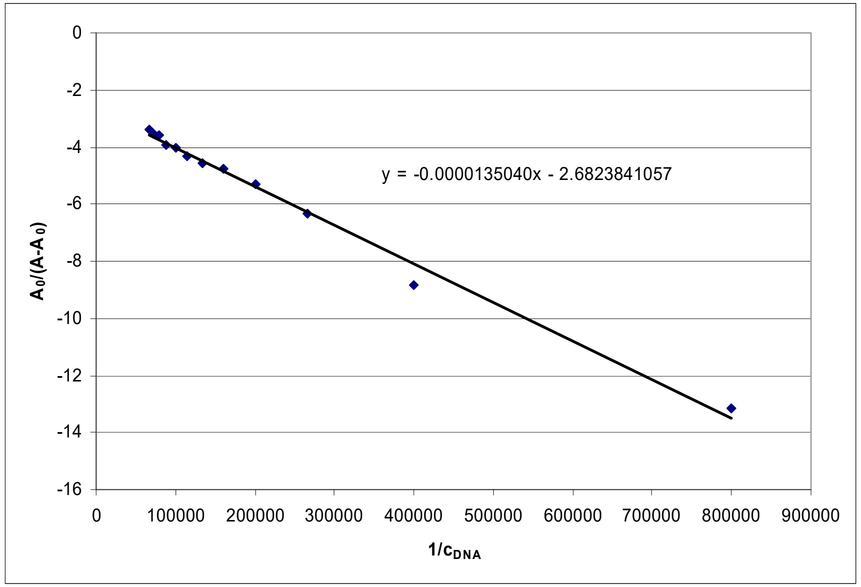

Figure S277. UV-vis absorption spectra of compound **16** (10 µM) in the presence of the increasing amount of cf-DNA (0-15 µM) (left). The plot of $A_0/A-A_0$ versus 1/[DNA] yielded the binding constant (right).

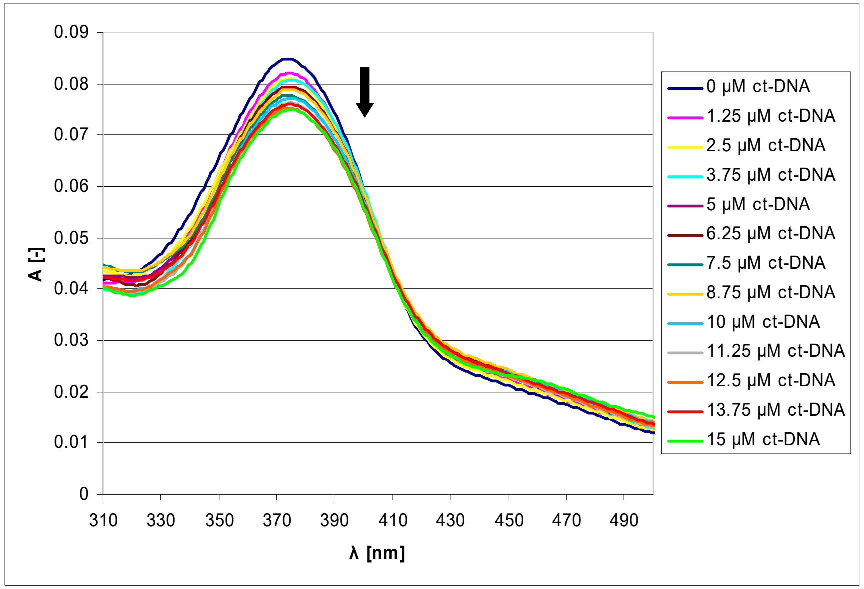

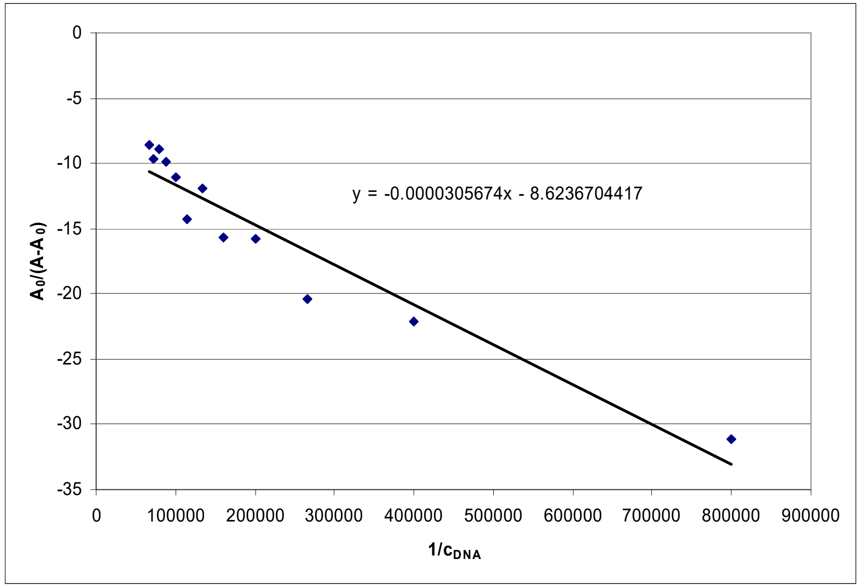

Figure S278. UV-vis absorption spectra of compound **17** (10 µM) in the presence of the increasing amount of cf-DNA (0-15 µM) (left). The plot of $A_0/A-A_0$ versus 1/[DNA] yielded the binding constant (right).

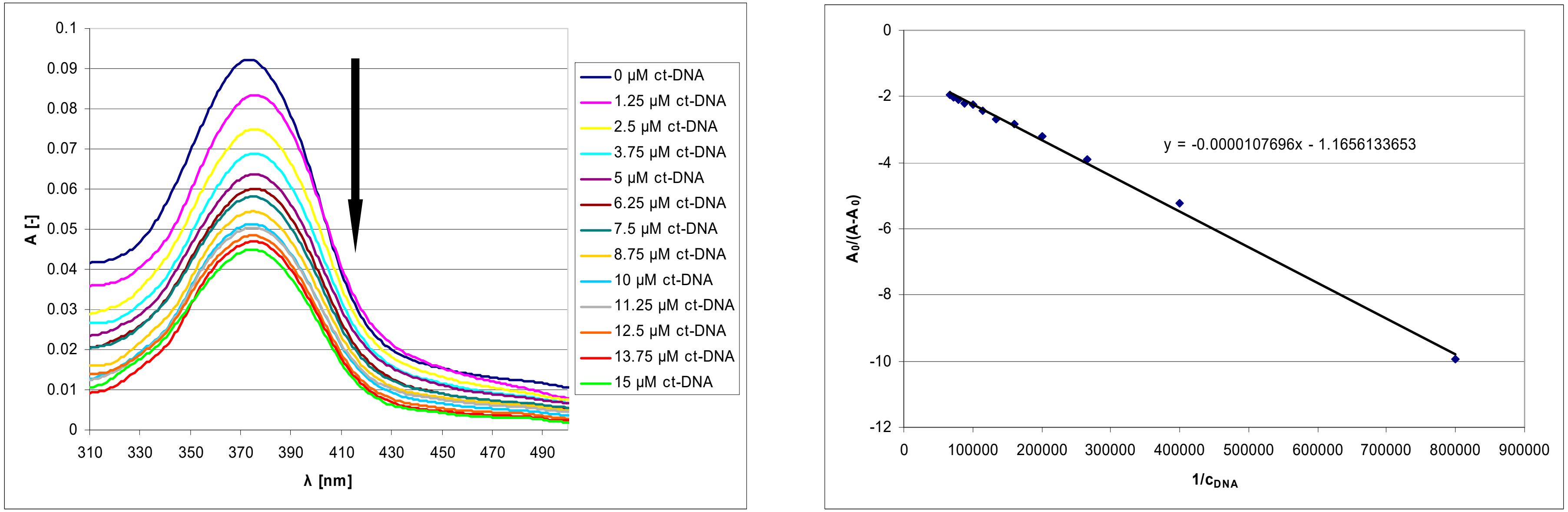

Figure S279. UV-vis absorption spectra of compound **18** (10 µM) in the presence of the increasing amount of cf-DNA (0-15 µM) (left). The plot of $A_0/A-A_0$ versus 1/[DNA] yielded the binding constant (right).

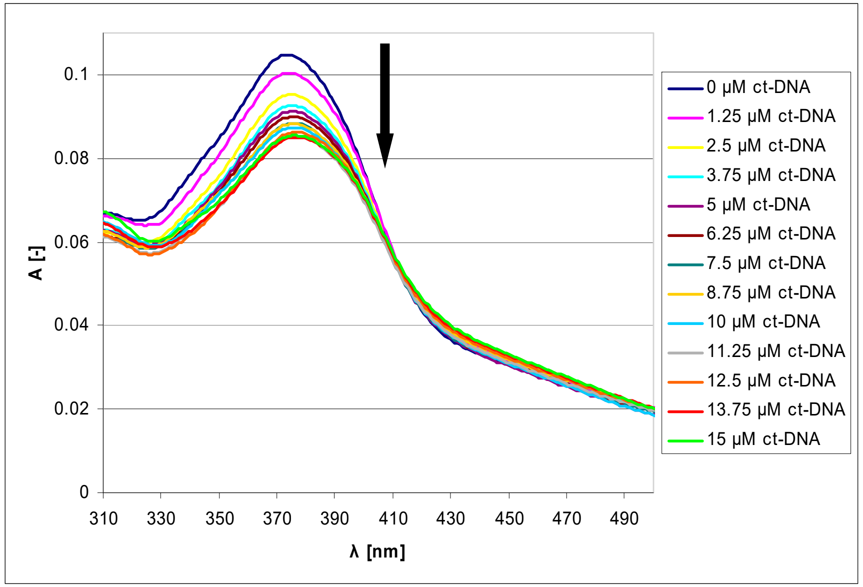

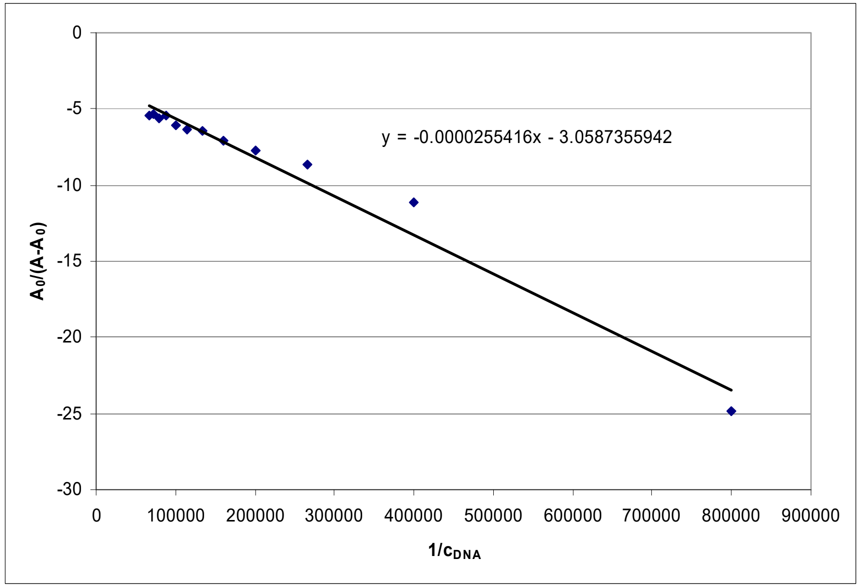

Figure S280. UV-vis absorption spectra of compound **19** (10 µM) in the presence of the increasing amount of cf-DNA (0-15 µM) (left). The plot of $A_0/A-A_0$ versus 1/[DNA] yielded the binding constant (right).

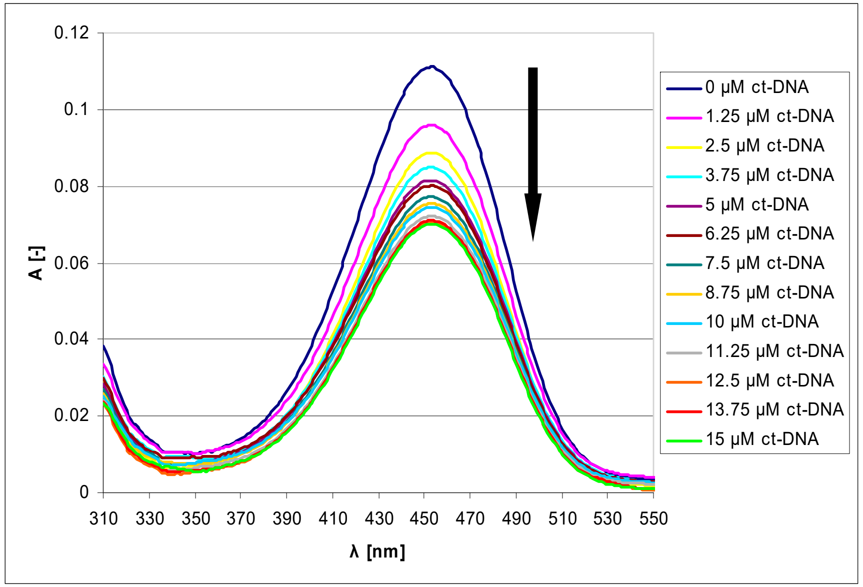

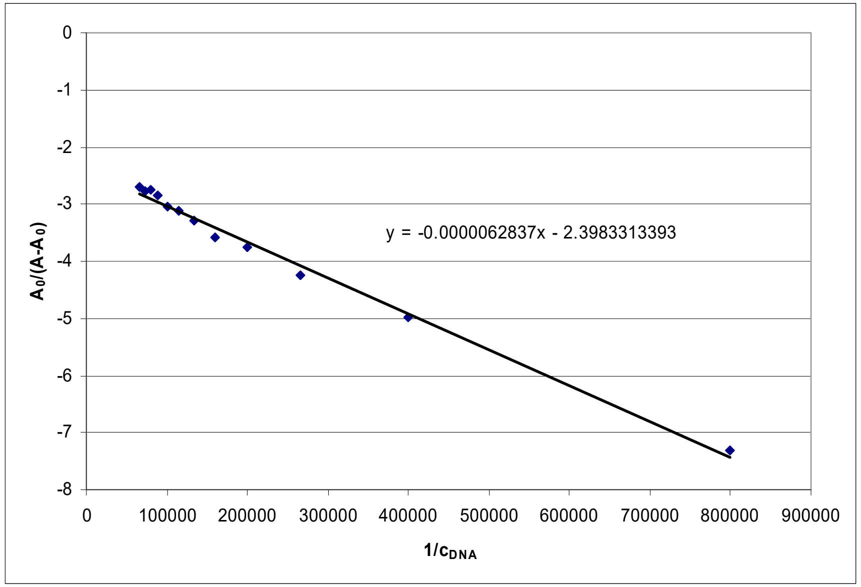

Figure S281. UV-vis absorption spectra of compound **36** (10 µM) in the presence of the increasing amount of cf-DNA (0-15 µM) (left). The plot of $A_0/A\text{-}A_0$ versus 1/[DNA] yielded the binding constant (right).

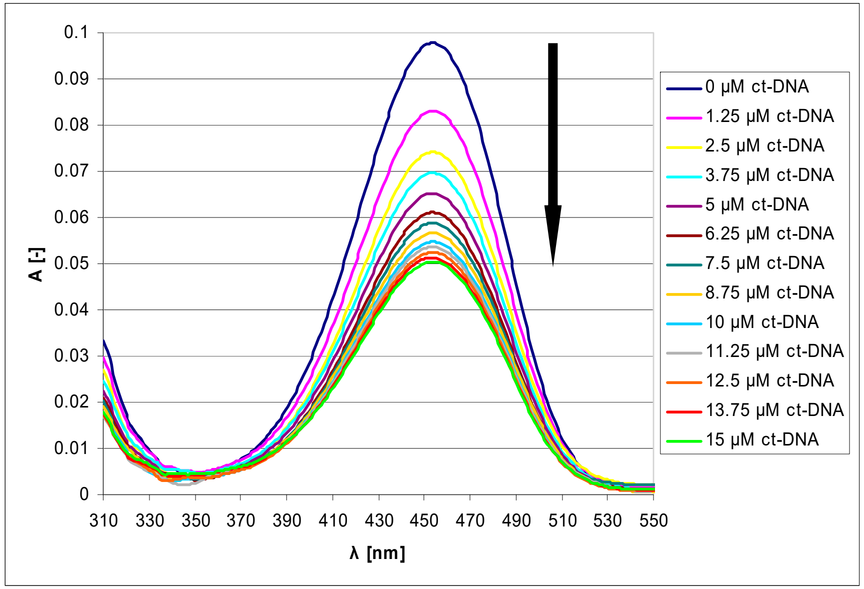

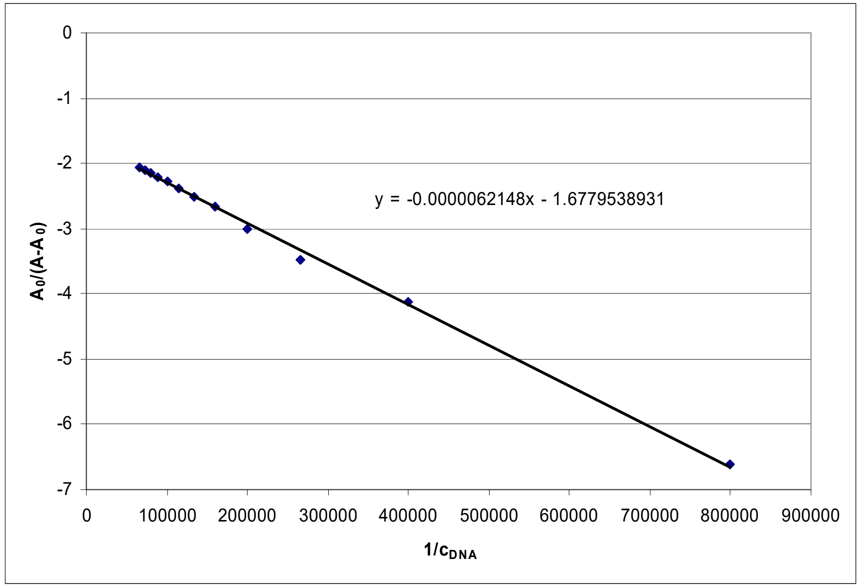

Figure S282. UV-vis absorption spectra of compound **37** (10 μM) in the presence of the increasing amount of cf-DNA (0-15 μM) (left). The plot of $A_0/A-A_0$ versus 1/[DNA] yielded the binding constant (right).

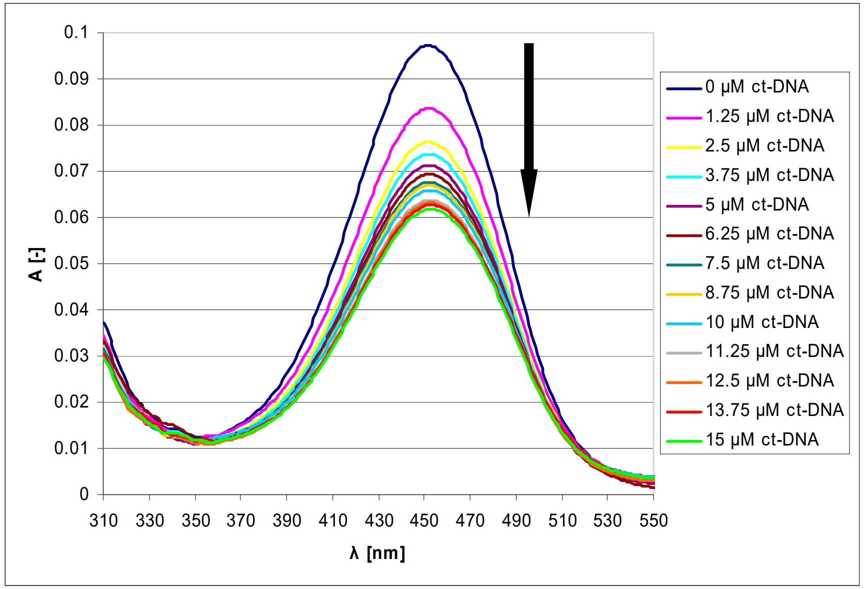

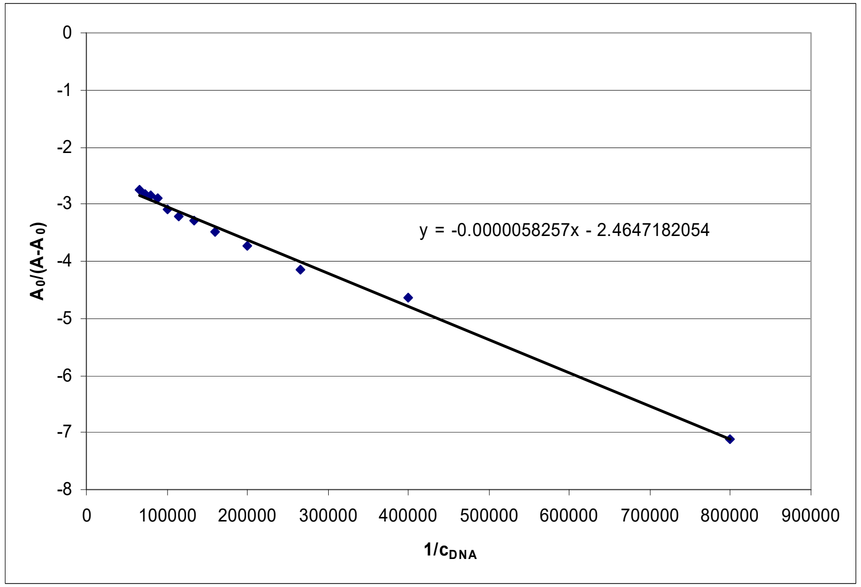

Figure S283. UV-vis absorption spectra of compound **38** (10 µM) in the presence of the increasing amount of cf-DNA (0-15 µM) (left). The plot of $A_0/A\text{-}A_0$ versus 1/[DNA] yielded the binding constant (right).

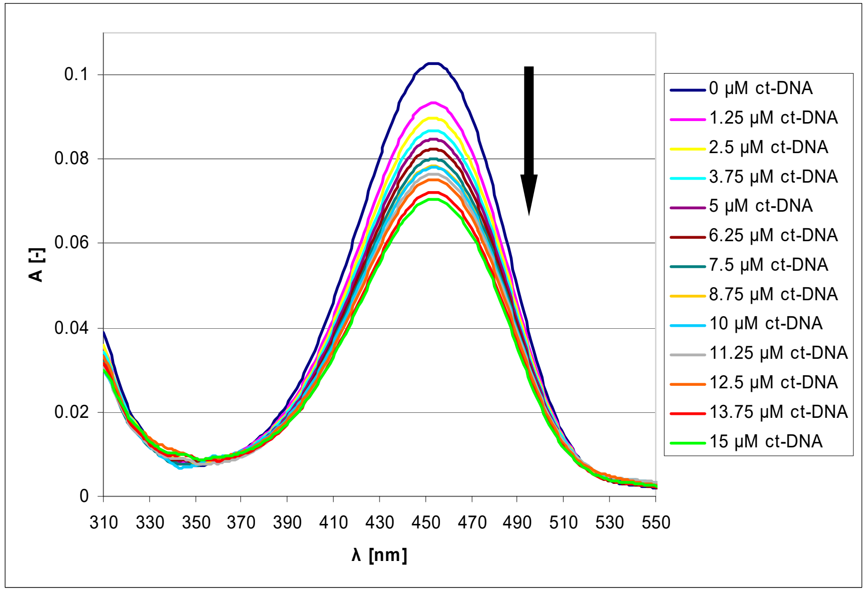

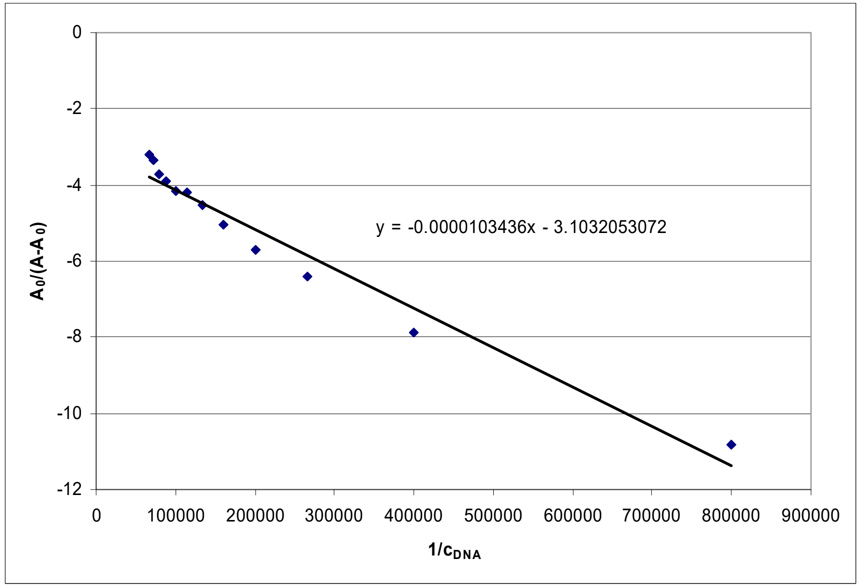

Figure S284. UV-vis absorption spectra of compound **39** (10 μM) in the presence of the increasing amount of cf-DNA (0-15 μM) (left). The plot of $A_0/A-A_0$ versus 1/[DNA] yielded the binding constant (right).

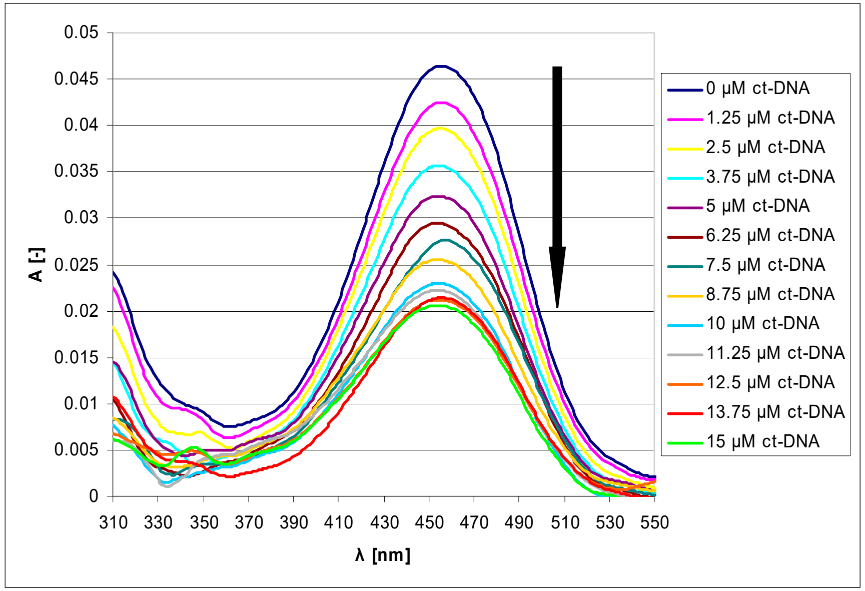

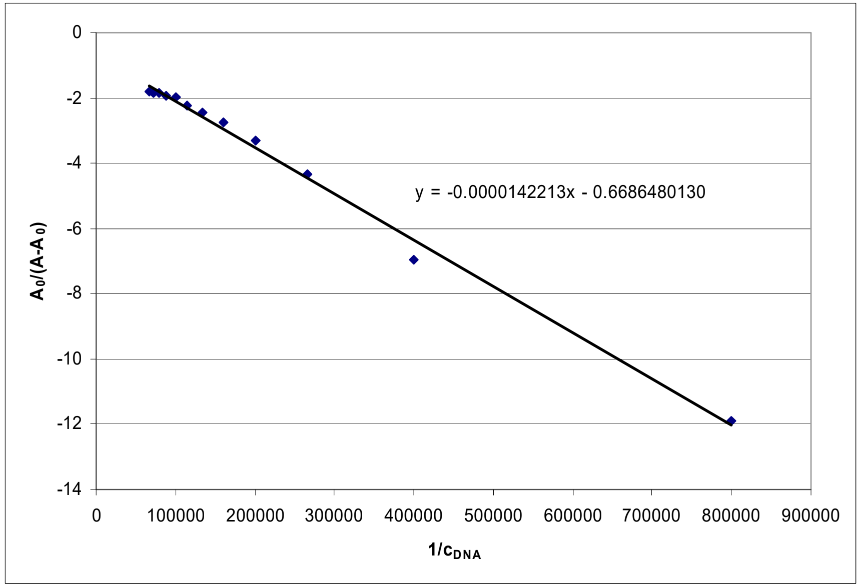

Figure S285. UV-vis absorption spectra of compound **40** (10 μM) in the presence of the increasing amount of cf-DNA (0-15 μM) (left). The plot of $A_0/A-A_0$ versus 1/[DNA] yielded the binding constant (right).

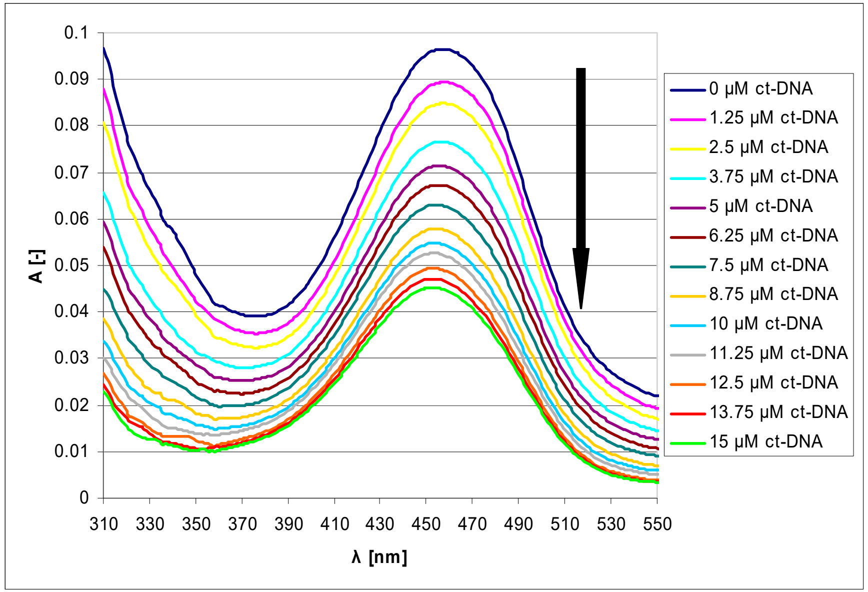

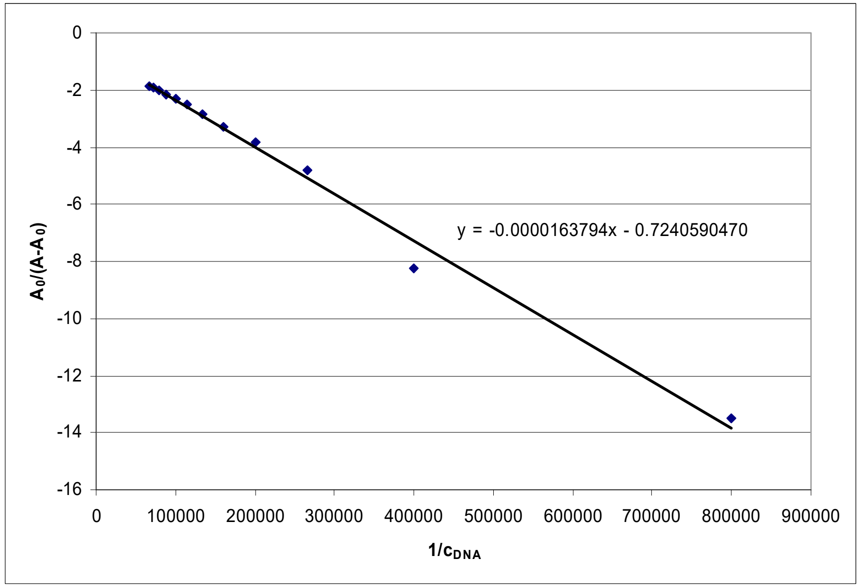

Figure S286. UV-vis absorption spectra of compound **41** (10 μM) in the presence of the increasing amount of cf-DNA (0-15 μM) (left). The plot of $A_0/A-A_0$ versus 1/[DNA] yielded the binding constant (right).

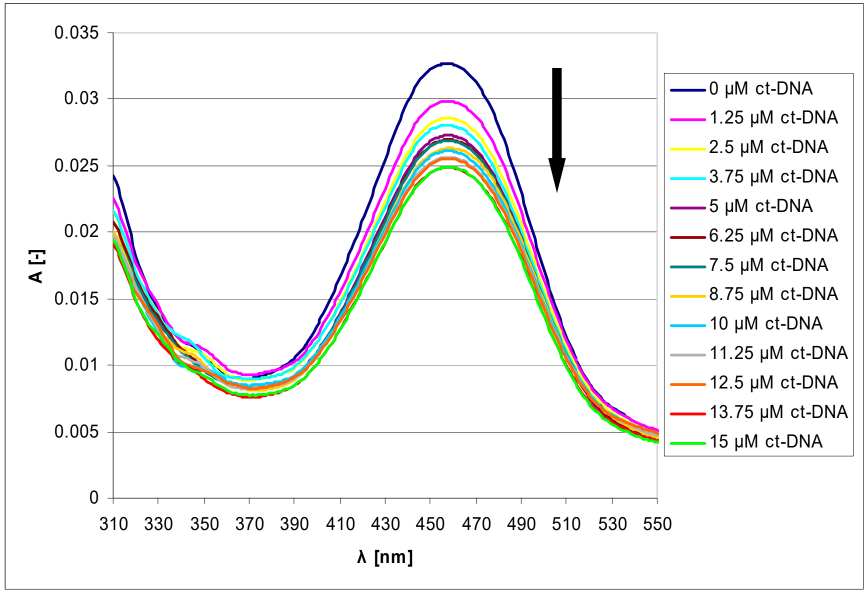

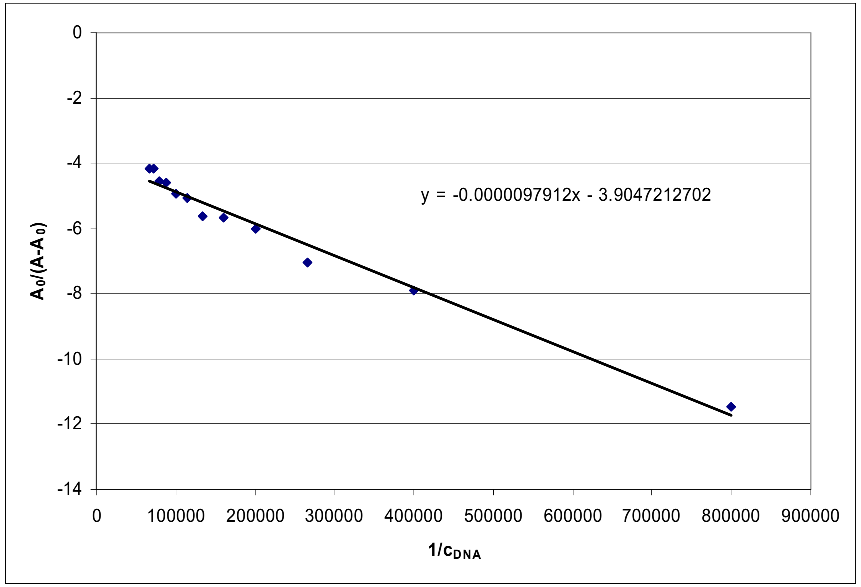

Figure S287. UV-vis absorption spectra of compound **42** (10 μM) in the presence of the increasing amount of cf-DNA (0-15 μM) (left). The plot of $A_0/A-A_0$ versus 1/[DNA] yielded the binding constant (right).

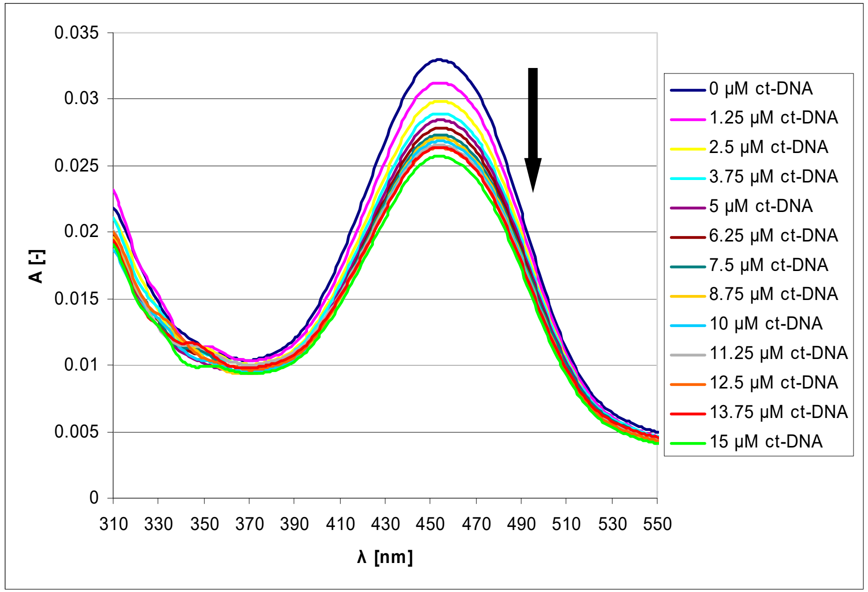

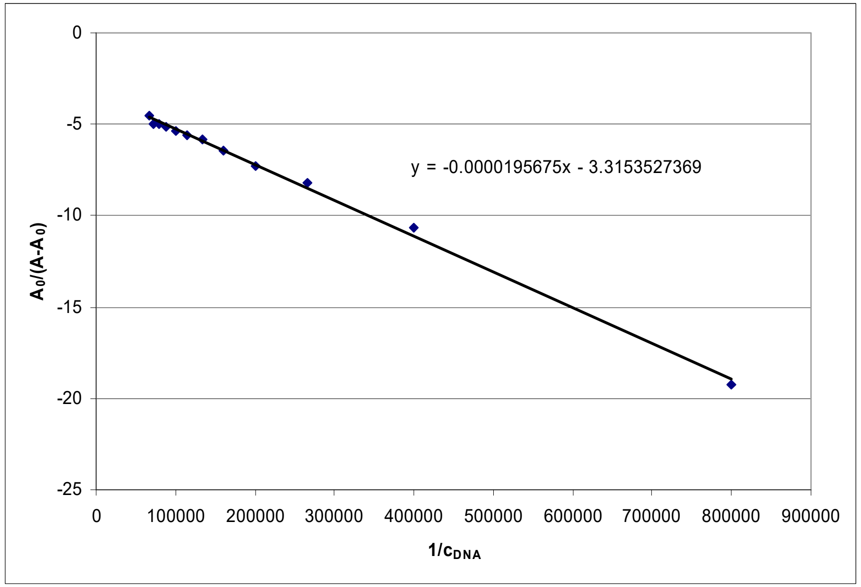

Figure S288. UV-vis absorption spectra of compound **43** (10 μM) in the presence of the increasing amount of cf-DNA (0-15 μM) (left). The plot of $A_0/A-A_0$ versus 1/[DNA] yielded the binding constant (right).

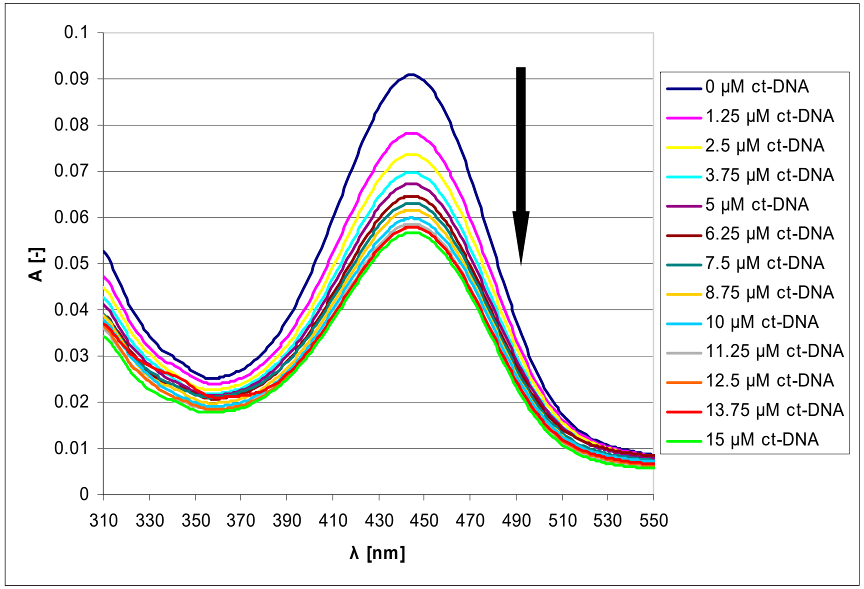

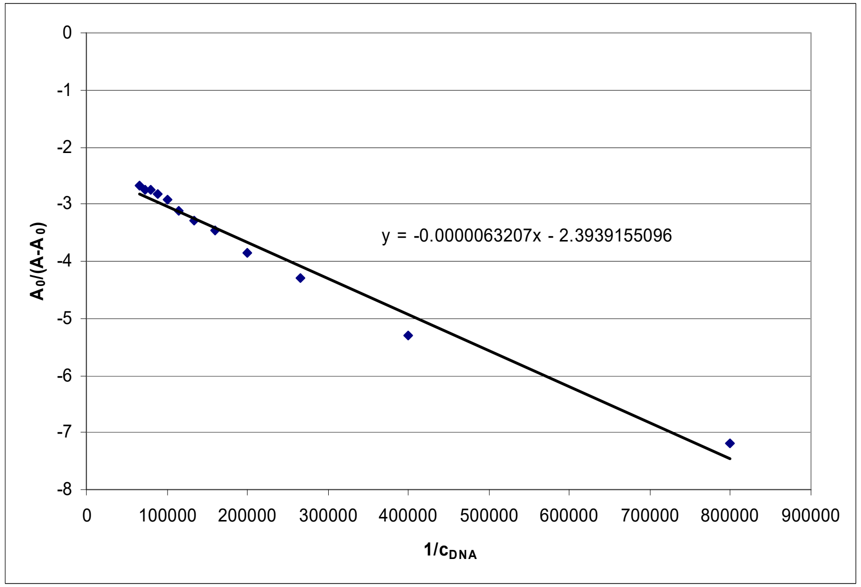

Figure S289. UV-vis absorption spectra of compound **54** (10 μM) in the presence of the increasing amount of cf-DNA (0-15 μM) (left). The plot of $A_0/A-A_0$ versus 1/[DNA] yielded the binding constant (right).

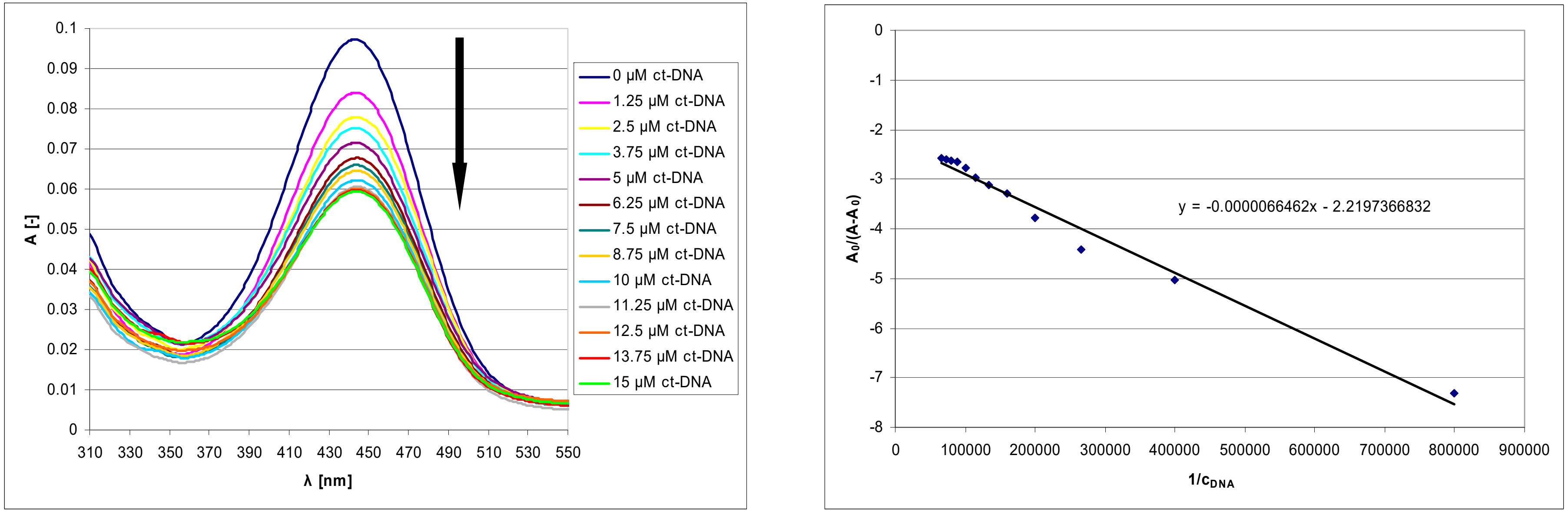

Figure S290. UV-vis absorption spectra of compound **55** (10 μM) in the presence of the increasing amount of cf-DNA (0-15 μM) (left). The plot of $A_0/A-A_0$ versus 1/[DNA] yielded the binding constant (right).

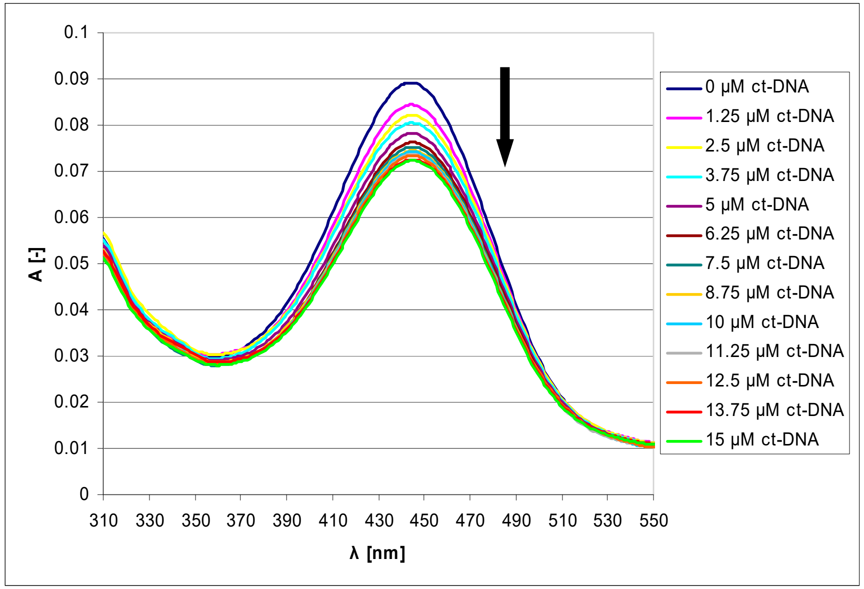

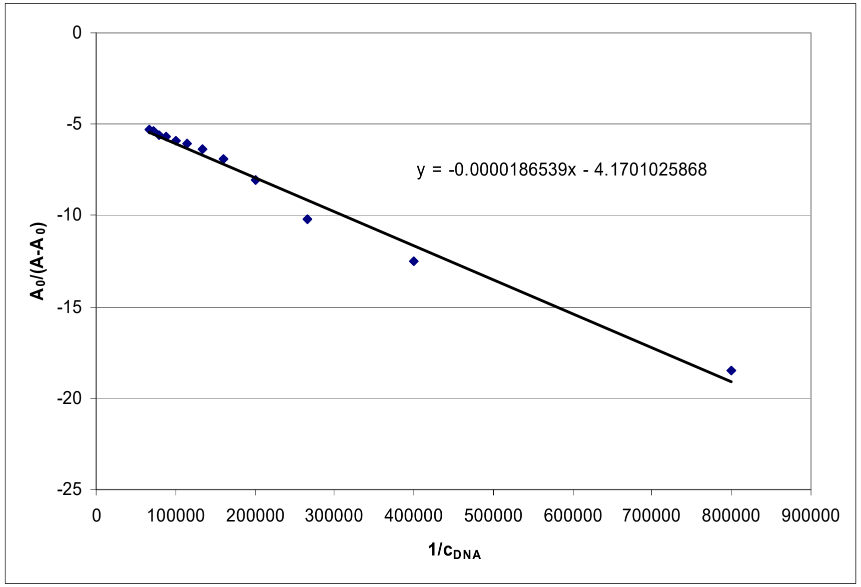

Figure S291. UV-vis absorption spectra of compound **56** (10 µM) in the presence of the increasing amount of cf-DNA (0-15 µM) (left). The plot of $A_0/A-A_0$ versus 1/[DNA] yielded the binding constant (right).

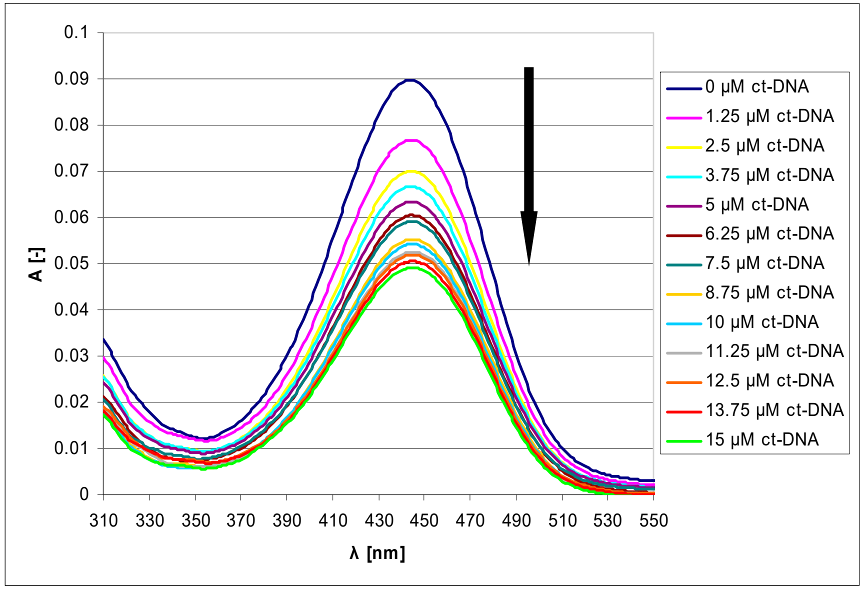

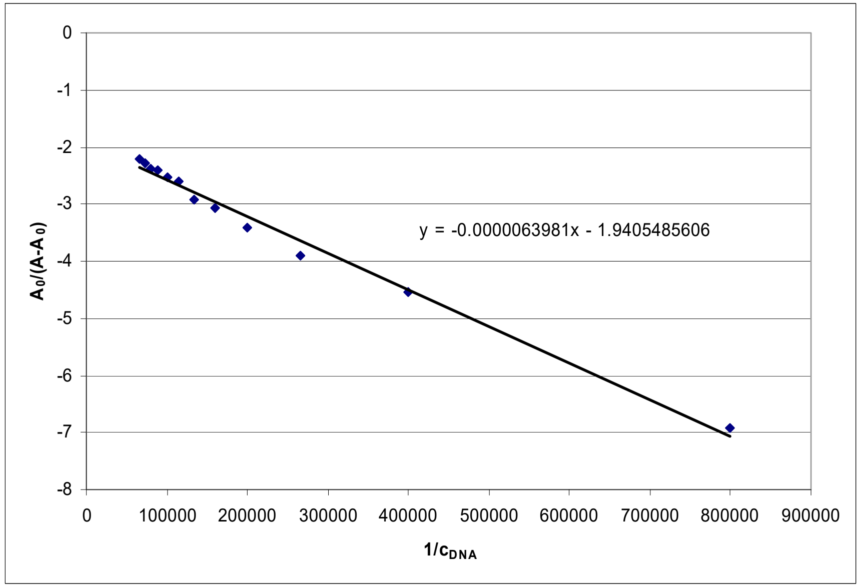

Figure S292. UV-vis absorption spectra of compound **57** (10 μM) in the presence of the increasing amount of cf-DNA (0-15 μM) (left). The plot of $A_0/A-A_0$ versus 1/[DNA] yielded the binding constant (right).

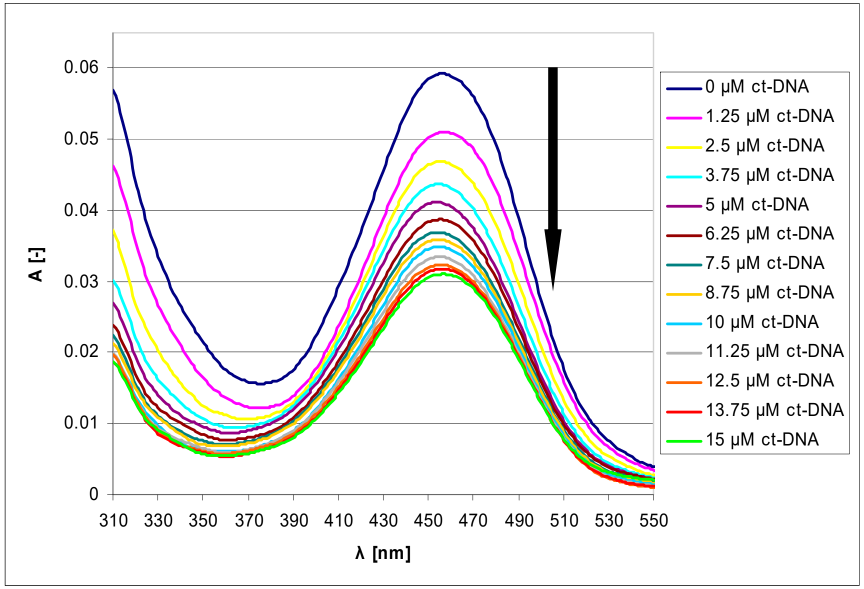

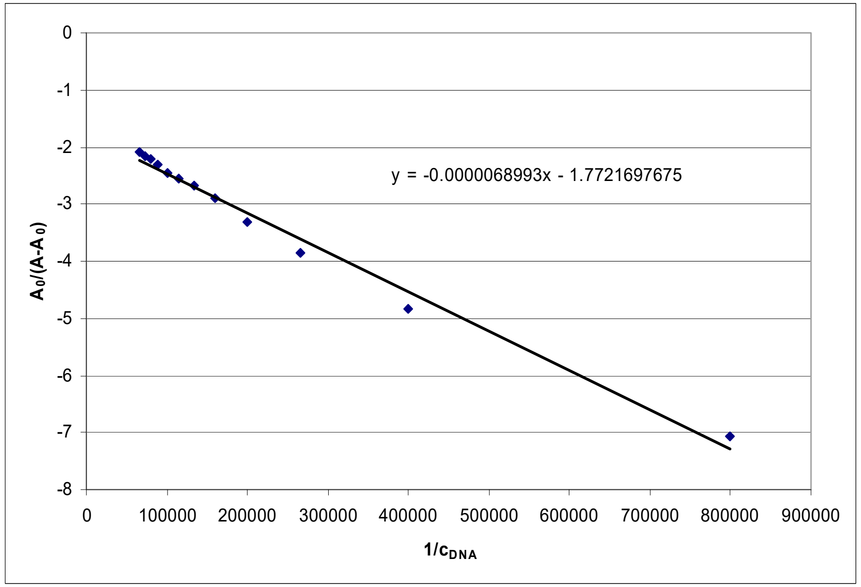

Figure S293. UV-vis absorption spectra of compound **58** (10 μM) in the presence of the increasing amount of cf-DNA (0-15 μM) (left). The plot of $A_0/A-A_0$ versus 1/[DNA] yielded the binding constant (right).

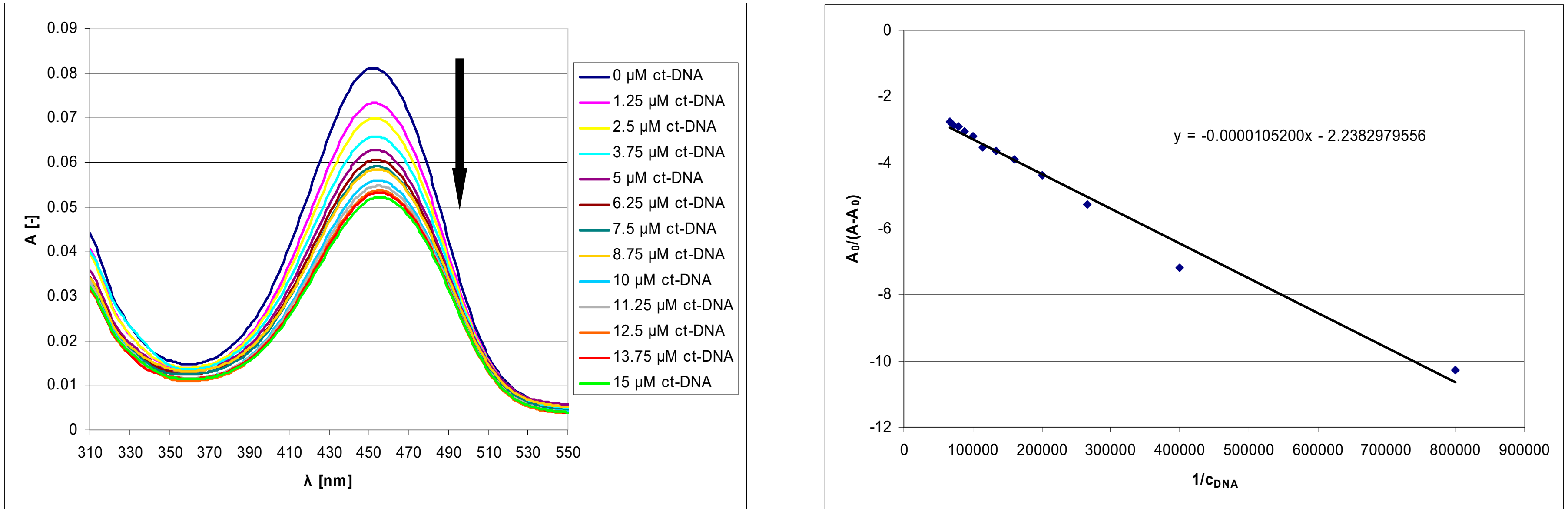

Figure S294. UV-vis absorption spectra of compound **59** (10 µM) in the presence of the increasing amount of cf-DNA (0-15 µM) (left). The plot of $A_0/A-A_0$ versus 1/[DNA] yielded the binding constant (right).

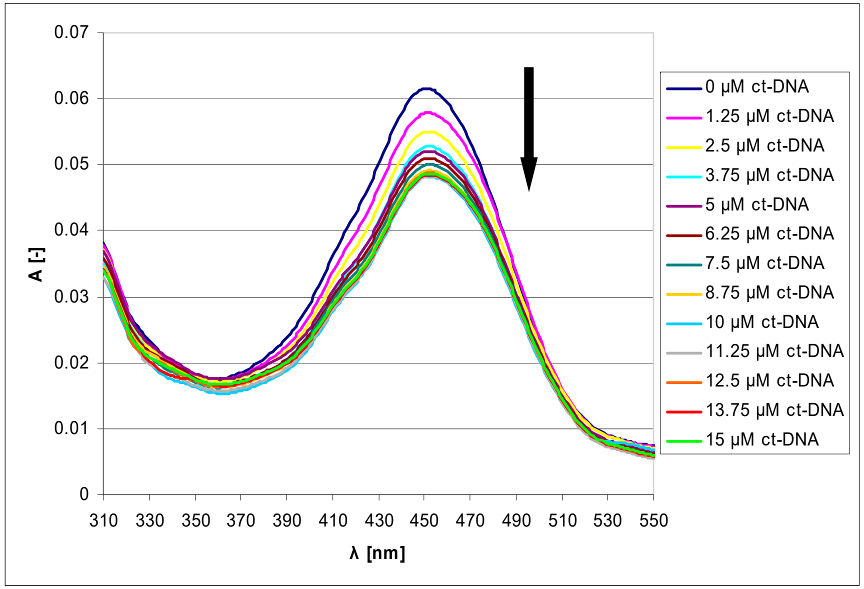

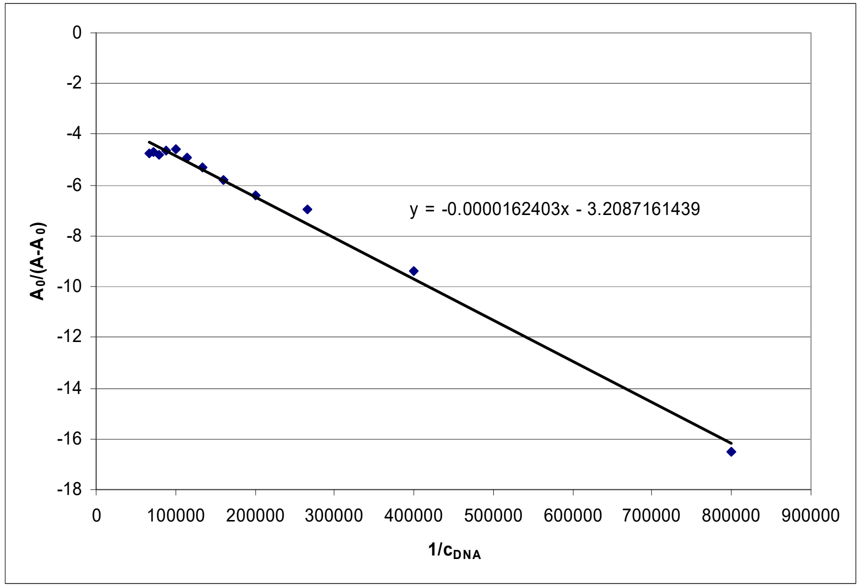

Figure S295. UV-vis absorption spectra of compound **60** (10 μM) in the presence of the increasing amount of cf-DNA (0-15 μM) (left). The plot of $A_0/A-A_0$ versus 1/[DNA] yielded the binding constant (right).

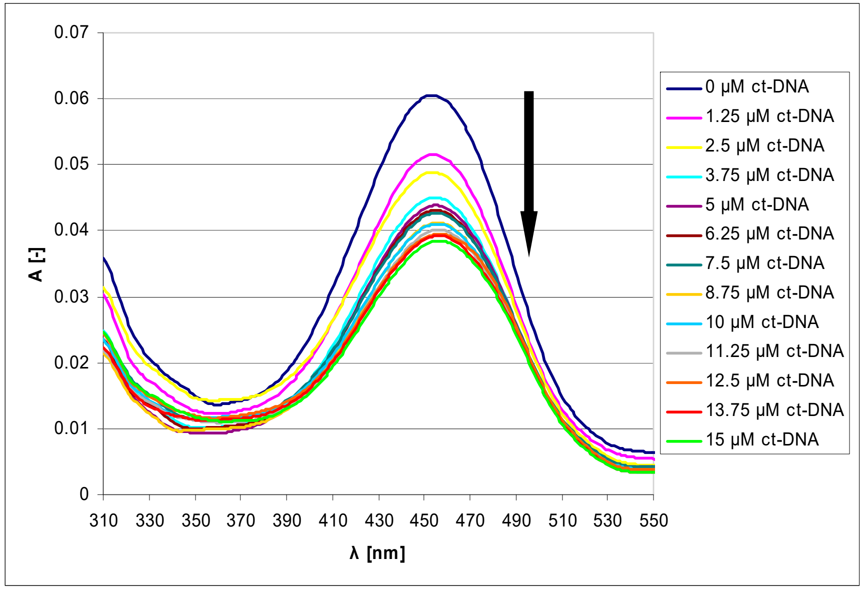

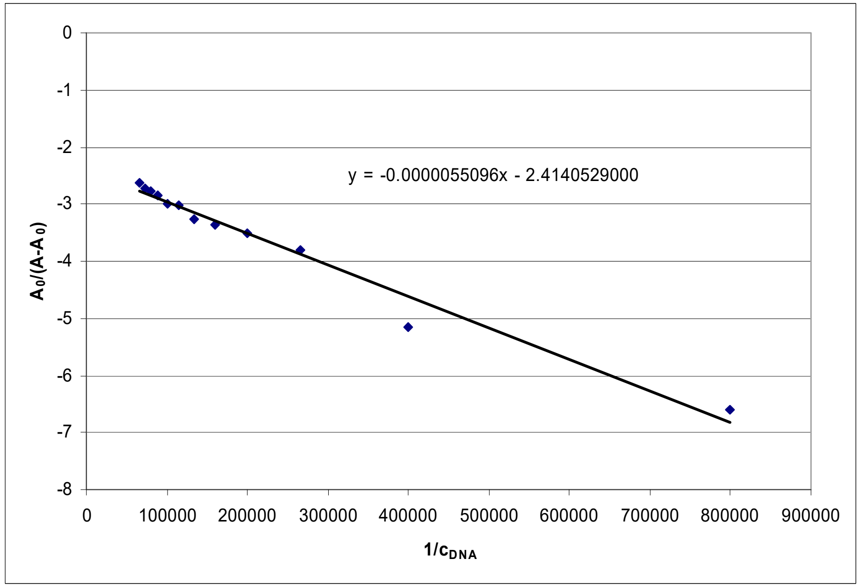

Figure S296. UV-vis absorption spectra of compound **61** (10 µM) in the presence of the increasing amount of cf-DNA (0-15 µM) (left). The plot of $A_0/A-A_0$ versus 1/[DNA] yielded the binding constant (right).

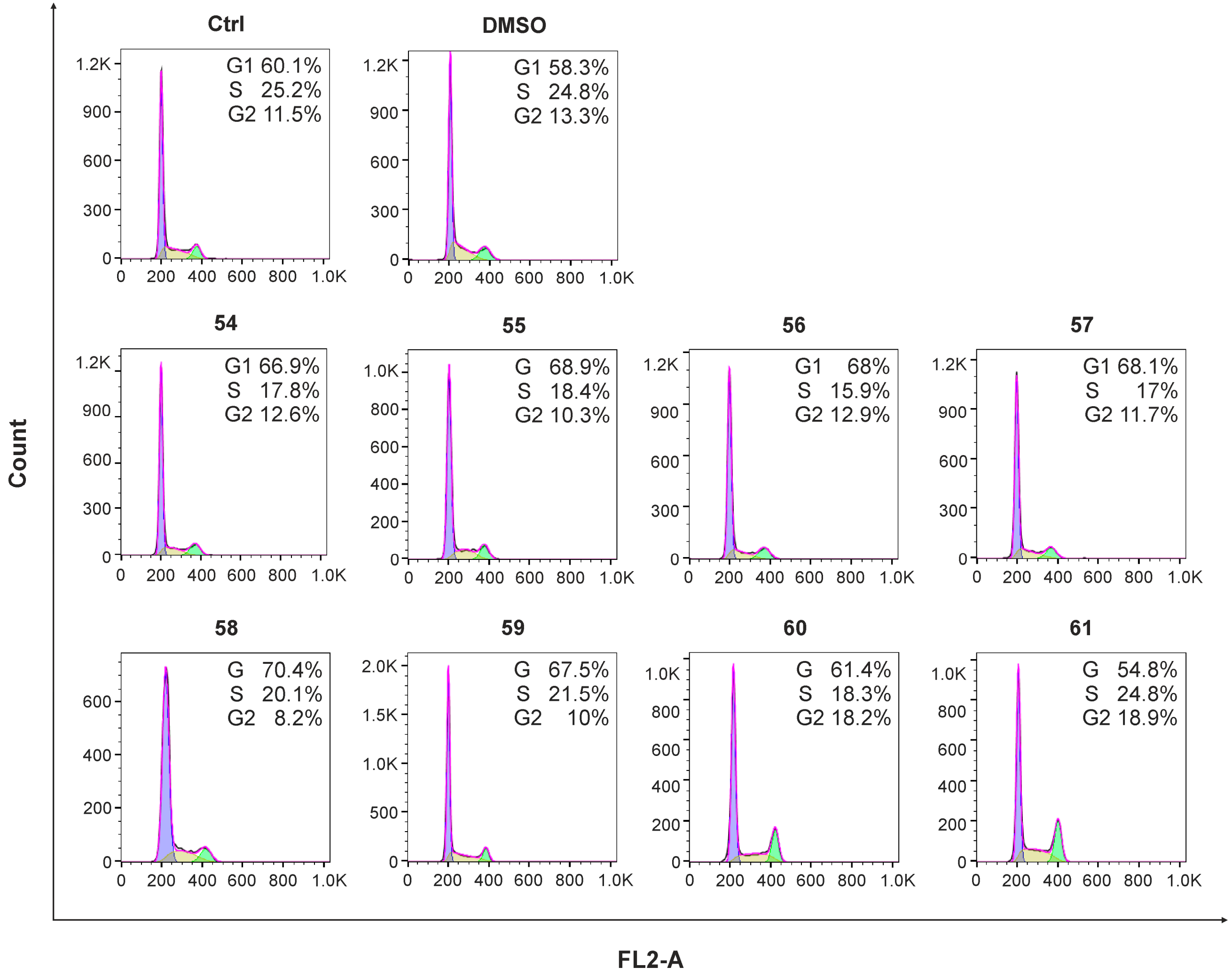

Figure S297. Influence of compound **54** (5.5 µM), **55** (3.4 µM), **56** (12 µM), **57** (3.1 µM), **58** (3.9 µM), **59** (5.4 µM), **60** (31 µM), and **61** (4.8 µM) on cell cycle distribution in HepG2 cells. Flow cytometry analysis of cells treated for 24 h with tested compounds. One representative experiment of three is shown.

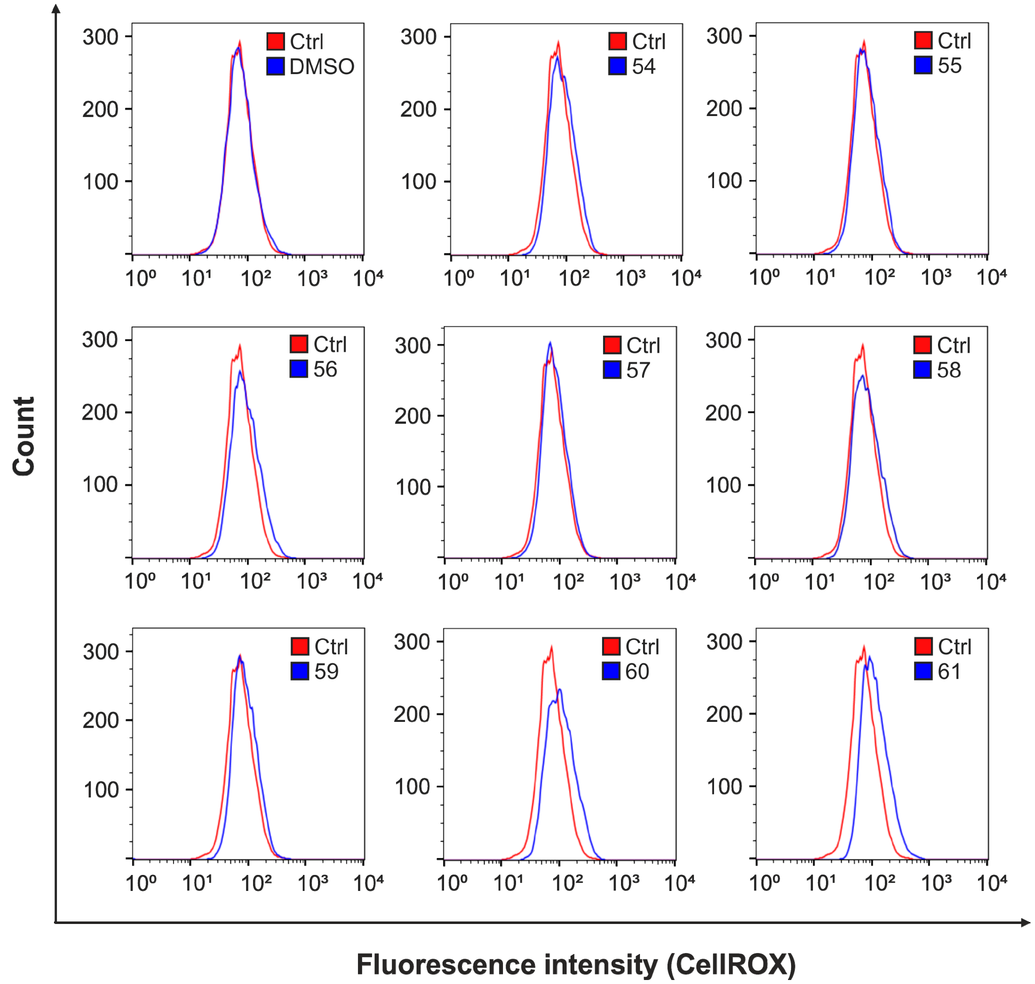

Figure S298. ROS production in HepG2 cells after 24 h incubation with compound **54** (5.5 µM), **55** (3.4 µM), **56** (12 µM), **57** (3.1 µM), **58** (3.9 µM), **59** (5.4 µM), **60** (31 µM), and **61** (4.8 µM). The oxidative stress was evaluated using CellROX Deep Red Reagent by flow cytometry. Fluorescence intensity shift is an indication of higher oxidative stress compared to control.

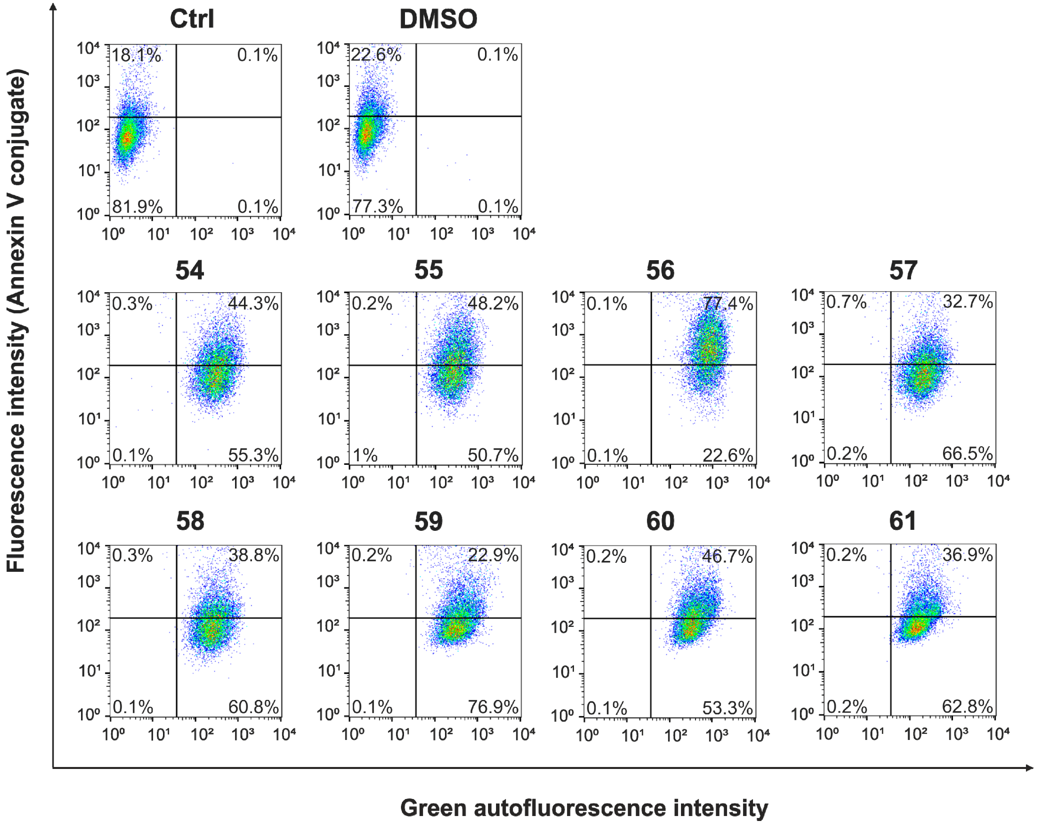

Figure S299. Flow cytometry analysis of apoptosis/necrosis in HepG2 cells after cell toxicity induction with compound **54** (5.5 μM), **55** (3.4 μM), **56** (12 μM), **57** (3.1 μM), **58** (3.9 μM), **59** (5.4 μM), **60** (31 μM), and **61** (4.8 μM). The chosen concentration of each compound corresponded to whole IC50 values. Apoptosis rate was analyzed by flow cytometry using Annexin V Alexa 647 conjugate. Intensive green autofluorescence signal of studied compounds was used as a marker of live cells with intact cell membranes. The more advanced apoptotic changes occur, the more permeable the cell membrane becomes, which leads to leakage of the compound, and less intensive green fluorescence is observed. The representative percentage distribution of live (lower left and lower right quadrants) and apoptotic cells (upper left and upper right quadrants) in the analyzed HepG2 population.

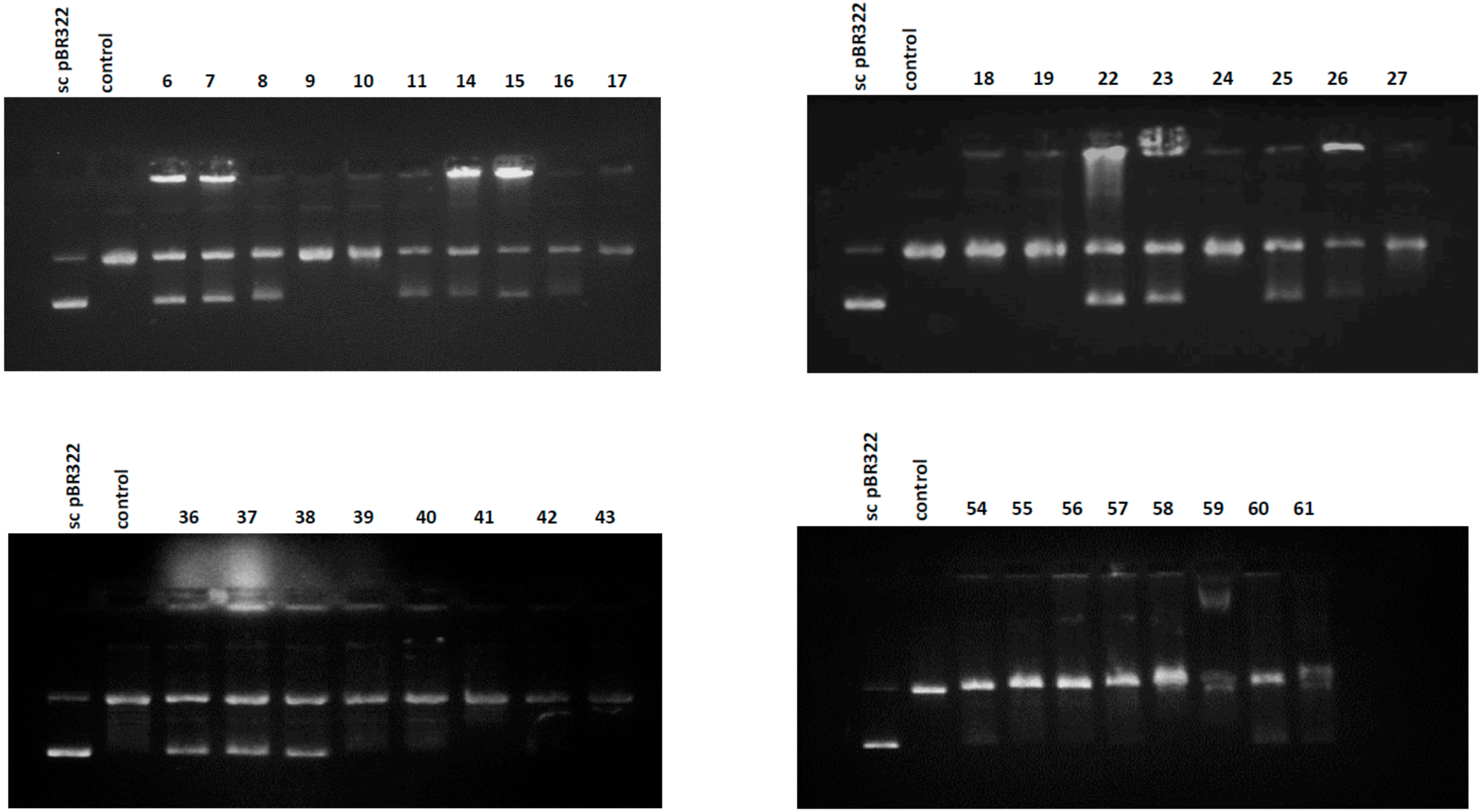

Figure S300. Human Topoisomerase IIα relaxation assay in the presence of modified with carborane cluster naphthalic anhydrides (**6**, **7**, **14**, **15**, **22**, **23**) and 1,8-naphthalimides (**8-11**, **16-19**, **24-27**, **36-43**, **54-61**), at a concentration of 100 μM. SC – supercoiled DNA.

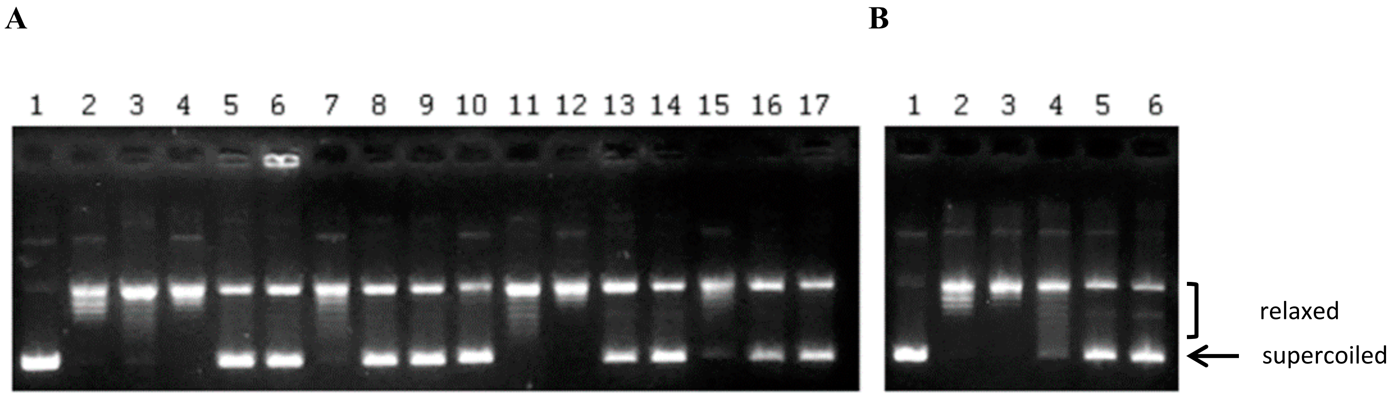

Figure S301. Inhibition of the relaxation activity of human topoisomerase IIα in the presence of compounds **6**, **8**, **36**, **38,** and mitonafide.
A: 1 – s.c. pBR, 2 – control hTopo II, 3 – compound **6** - 1 μM, 4 – compound **6** - 5 μM, 5 – compound **6** - 10 μM, 6 – compound **6** - 100 μM, 7 – compound **6** - 0.1 μM, 8 – compound **8** - 1 μM, 9 – compound **8** - 10 μM, 10 – compound **8** - 100 μM, 11 – compound **36** - 1 μM, 12 – compound **36** - 5 μM, 13 – compound **36** - 10 μM, 14 – compound **36** - 100 μM, 15 – compound **38** - 0.1 μM, 16 – compound **38** - 1 μM, 17 – compound **38** - 10 μM. B: 1 – s.c. pBR, 2 – control hTopo II, 3 – mitonafide - 0.1 μM, 4 – mitonafide - 1 μM, 5 – mitonafide - 10 μM, 6 – mitonafide - 100 μM.

Table S1. Crystallographic data.

Compound	**23**
Wavelength [Å]	1.54184
Temperature [K]	100
Space group	C2/c
Z	8
a [Å]	24.244(5)
b [Å]	6.8390(14)
c [Å]	25.864(5)
α [°]	90
β [°]	108.01(3)
γ [°]	90
R_{int}	0.0489
Resolution [Å]	0.983
% completeness	99.1
Independent reflections	2224
R/R(for F_o>4σ)	0.0569/0.0426
CSD code	2156512
